# Identification of novel microRNAs in the embryonic mouse brain using deep sequencing

**DOI:** 10.1007/s11010-023-04730-2

**Published:** 2023-04-15

**Authors:** Susanna Szakats, Alice McAtamney, Megan J. Wilson

**Affiliations:** Developmental Genomics Laboratory, Department of Anatomy, School of Biomedical Sciences, University of Otago, P.O. Box 56, Dunedin, 9054 New Zealand

**Keywords:** miRNA, Novel, Neurodevelopment, snoRNA, Y-RNA

## Abstract

Many advances in small RNA-seq technology and bioinformatics pipelines have been made recently, permitting the discovery of novel miRNAs in the embryonic day 15.5 (E15.5) mouse brain. We aimed to improve miRNA discovery in this tissue to expand our knowledge of the regulatory networks that underpin normal neurodevelopment, find new candidates for neurodevelopmental disorder aetiology, and deepen our understanding of non-coding RNA evolution. A high-quality small RNA-seq dataset of 458 M reads was generated. An unbiased miRNA discovery pipeline identified fifty putative novel miRNAs, six of which were selected for further validation. A combination of conservation analysis and target functional prediction was used to determine the authenticity of novel miRNA candidates. These findings demonstrate that miRNAs remain to be discovered, particularly if they have the features of other small RNA species.

**Supplementary Information:**

The online version contains supplementary material available at 10.1007/s11010-023-04730-2.

## Background

MicroRNAs (miRNAs) are a class of small non-coding RNAs (ncRNAs). Initially discovered in *Caenorhabditis elegans* in 1993, miRNAs are encoded by genome sequences and transcribed in the same manner as genes [42]. Canonical miRNA biogenesis involves a well-defined series of RNA processing steps, ultimately yielding a mature miRNA of ~ 20–25 nucleotides in length [5]. Complementary base pairing between the miRNA seed sequence, bases 2–8 from the 5′ end, and sequences within the 3′ UTR of the mRNA, enables the RNA Induced Silencing Complex (RISC) associated with the mature miRNA to prevent translation or degradation the mRNA [36]. Unique spatial and temporal expression profiles indicate the importance of miRNAs in developing tissues, including the brain. Neurodevelopment is disrupted when all miRNA activity is deleted via *Ago2* knockout [45, 54]. Furthermore, the disruption of specific miRNAs, including *miR-9*, *miR-124*, and *miR-132,* results in developmental brain defects and altered brain function in adults [28, 40, 52, 55, 58]. Thus, the regulation of gene expression by miRNAs is necessary for typical neurodevelopment. The importance of miRNAs in neurodevelopment has been noted by various researchers looking at the origin of neurodevelopmental disorders. Multiple studies have shown that miRNA expression profiles differ between individuals with neurodevelopmental and psychiatric disorders and matched control groups [1, 23, 24, 30], suggesting that miRNA dysregulation is a component of neurodiversity and disease aetiology.

**Fig. 1:**
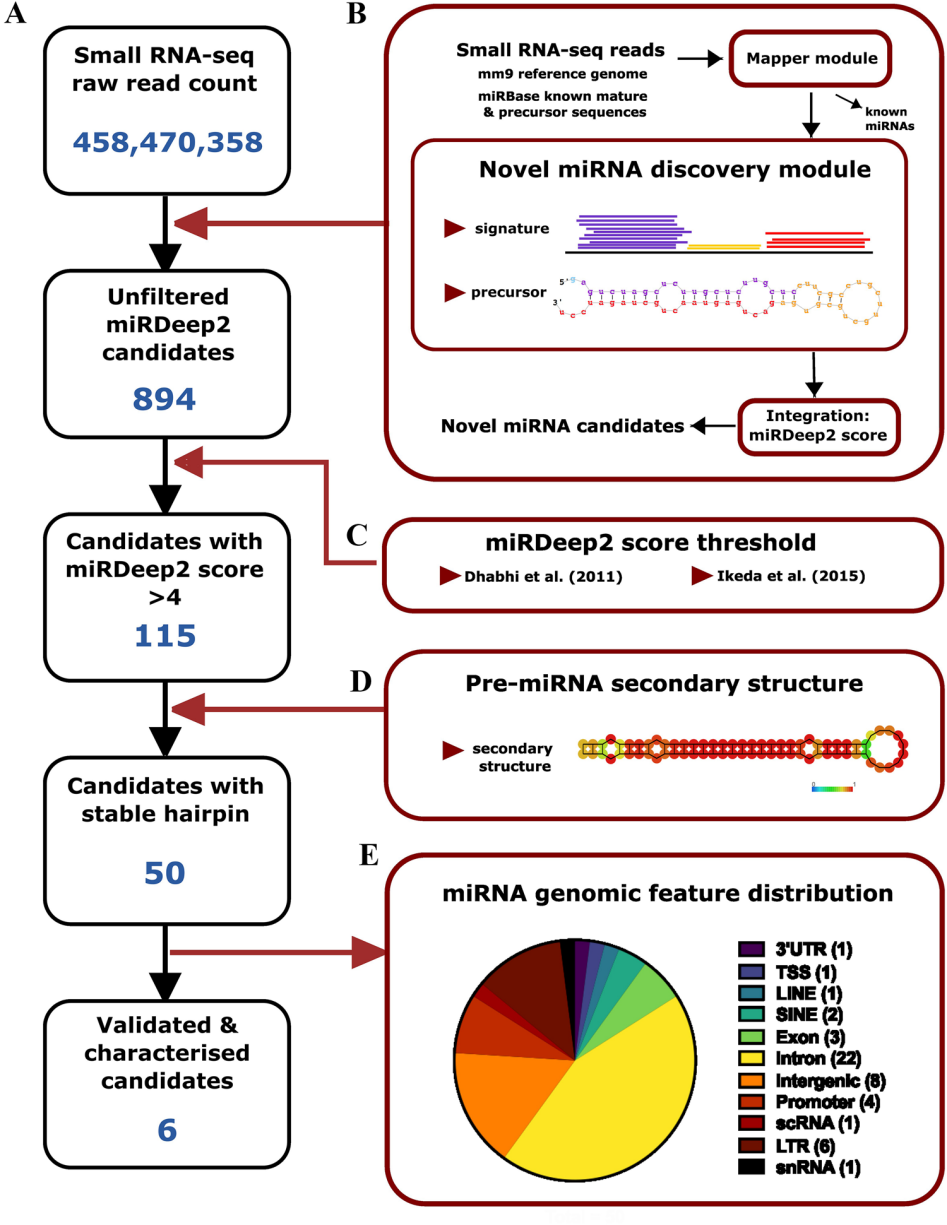
Novel miRNA discovery pipeline. **A** Flowchart showing the number of novel miRNAs at each step in the discovery pipeline. **B** Schematic summary of the miRDeep2 criteria. A simplified pipeline adapted from [21] was used—the input of trimmed and filtered small RNA-seq reads into the miRDeep2 mapper module along with the reference genome (mm9) and sequences of known mature and precursor mouse miRNAs from miRbase were detected. Sequence reads that mapped to mm9, but not known miRNA sequences, were submitted to the novel miRNA discovery module of miRDeep2. Two parameters determined novel miRNA discovery: (1) Characteristic read signature with low read counts for putative star (red) and loop (yellow) sequences compared to higher read counts for predicted mature sequences (red). (2) Formation of a stem-loop-stem RNA secondary structure by base pairing between the mature (purple) and star (red) sequences joined by the loop sequence (yellow). The main miRDeep2 algorithm integrates the results of these three analyses to generate a miRDeep2 score that predicts the likelihood of a particular sequence being a novel miRNA. **C** References used to guide miRDeep2 score threshold applied. **D** Thermal stability and low positional entropy of the predicted RNA secondary structure according to the RNAfold program. **E** Distribution of the candidate miRNAs. Pie chart indicates the number of novel miRNAs across 11 genomic features. *3′ UTR* 3′ Untranslated Region, *TSS* Transcription Start Site, *LINE* Long Interspersed Nuclear Element, *SINE* Short Interspersed Nuclear Element, *scRNA* small conditional RNA, *LTR* Long Terminal Repeat retrotransposon, *snRNA* small nuclear RNA

Initial miRNA discovery was achieved using cloning and gel-based methods [41], where small RNAs were isolated from total RNA libraries by size fractionation, used to create cDNA clones, and then subjected to Sanger sequencing to identify putative sequences of small RNAs. A Northern blot would subsequently confirm the expression of an RNA. These early experimental approaches to identify miRNAs were followed by computational prediction strategies in which genome data were scanned for putative miRNA features. Prediction is based on the presence of an open reading frame, proximity to a gene promoter, and predicted thermal stability of a pre-miRNA hairpin [7]. Methods based on high-throughput sequencing have been developed to overcome the limitations of early experimental and computer-based techniques. High-throughput sequencing of small RNAs is followed by specialized bioinformatics tools that determine the probability of a read being a genuine miRNA. Combining experimental and computational strategies at a high-throughput scale, sequencing, and bioinformatics approaches have greatly increased the ability of researchers to detect novel miRNAs [20, 62]. The software miRDeep2 identifies novel miRNAs from small RNA-seq data and reports the probability that each candidate is a genuine miRNA [21]. In brief, small RNA-seq reads were mapped to the reference genome and quantified in comparison to existing miRBase miRNAs. The thermal stability of the predicted pre-miRNA secondary structure was calculated, and a read signature consisting of read counts for the mature miRNA sequence, star sequence, and loop was generated. Each candidate novel miRNA is assigned a score based on these parameters, and the estimated probability of the reported miRNA is genuine. The miRDeep2 workflow is summarized in Fig. 1B. Ongoing novel miRNA discovery has yielded new candidate miRNAs from many cells, tissues, developmental stages, species, and pathologies. Since miRNAs often demonstrate unique spatiotemporal expression patterns, many have been discovered because they are not present in specific contexts [13]. However, specific expression dynamics do not negate their importance; all novel miRNAs contribute to the genetic networks underpinning development, homeostasis, and/or pathological processes. Therefore, it is vital to continue searching for these undiscovered components to improve our understanding of the processes to which they contribute and because they represent potential candidates for novel avenues of research into disease-causing mechanisms [44]. In addition to its biomedical utility, this research can further our understanding of miRNA evolution [6].

This research aimed to update novel miRNA discovery in the developing mouse brain, which has not been investigated in the past decade [44], and could benefit significantly from improvements made over this period in small RNA-seq technology and specialist bioinformatics tools such as miRDeep2. We have assessed the characteristics of six candidate novel miRNAs discovered by the highly stringent miRDeep2 criteria in the E15.5 mouse brain by combining bioinformatics, conservation, expression data and functional prediction; the miRNA attributes that further support those identified candidates are genuine novel miRNAs. E15.5 was selected as the preferred developmental time point for our samples because it is a critical time period for regional identity and brain neurodevelopment, and also because it is the same time point investigated by Ling et al. [44], allowing us to build on their findings. Conservation analysis revealed a mix of results, which can be broadly categorised as highly conserved miRNAs (*Novel_3*, *Novel_11*, *Novel_16*, and *Novel_17*), potentially conserved miRNA (*Novel_1*), and potentially species-specific miRNA (*Novel_2*). RT-qPCR confirmed the expression of *Novel_1*, *2*, *3*, *11*, *16*, and *17* in multiple tissues. Finally, pathway analysis of the predicted target genes revealed potential functions for each candidate miRNA, where many over-represented pathways were involved in neurological processes, the cell cycle, and proliferation. Together, these data support the authenticity of the six candidate novel miRNAs and further indicate the likely validity of other candidates among those discovered in the E15.5 mouse brain.

## Methods and materials

### Animal husbandry & tissue collection

Breeding and dissection of C57BL/6 wild-type mice was performed with the approval of the University of Otago Animal Ethics Committee to generate embryos at specific developmental time points. The mice were housed in standard conditions with ad libitum access to food and water. A mating pair, a dam and a stud, were housed together for up to four nights and checked each morning for a copulation plug. After the identification of a copulation plug or after four consecutive nights, the dam and stud were separated, and the developmental stage was considered embryonic day 0.5 (E0.5). The dams were culled by cervical dislocation to collect embryos at E15.5. Embryo dissections were performed in cold, sterile 70% PBS to collect whole brain tissue for RNA isolation and gonad tissue to determine the sex of each embryo by the presence (male) or absence (female) of testis cords. In addition to E15.5 brain tissue, E11.5 brains, E15.5 liver and gonads, and the adult spinal cord were collected for RNA extraction.

### RNA isolation & purification

Individual brains that had been sexed were kept in 70% sterile PBS on ice for immediate RNA isolation or stored in RNALater (Ambion) at − 20 °C. RNA was extracted using the Purelink RNA miniprep kit (Ambion) following the manufacturer’s instructions, including additional DNase treatment. Manual homogenization was performed with a sterile needle tip before being passed through an 18-gauge syringe and centrifuged for 2 min at 12,000×*g*. In the final step, RNA was eluted in 50 μL mqH_2_O and stored at − 20 °C. RNA was purified using ethanol precipitation. The RNA pellet was resuspended in 15 μL mqH_2_O. The concentration of each sample was measured by using a NanoDrop spectrophotometre. The purity of each sample was analysed using the 260/230 and 260/280 ratios. RNA samples with ratios of 1.8–2.2 were considered of sufficient quality for downstream application.

### Novel miRNA discovery

The miRDeep2 tool was used to identify novel miRNAs in a previously generated small RNA-seq dataset consisting of 3 male and 3 female replicates from the E15.5 mouse brain [59]. The mm9 genome was indexed as a reference genome, and mature and precursor miRNA sequences were obtained from the latest version of miRBase (v. 22; [26]. Fastq sequences were processed and quality controlled using FastQC [2], and then all six sample FASTA files (*n* = 3/sex) were concatenated to pool data and increase read depth. Fasta files were collapsed and run through a series of commands to map the sequences back to the reference genome. First, bwa aln was used to find the coordinates of the input reads and convert fasta files into sai format. Second, bwa samse generated sam alignments for reads from the sai input. Next, the samtools view was used to convert the SAM files into BAM files. Finally, the bam files were converted into the format required by miRDeep2.arf, by bwa_sam_converter. The main miRDeep2.pl script was run on the mapped arf file using the reference genome and known miRNA sequences from miRBase. This script generated a main report summarising novel miRNA discovery, details of every individual miRNA identified, including statistical parameters, and pdf files with RNA secondary structure information on each novel miRNA [21].

### Pre-miRNA conservation

UCSC Blat [37] was used to align pre-miRNA sequences to the mm9 genome; the same assembly was used to discover small RNAs, along with a range of genomes from other species. The genomes selected for comparison included rat (another rodent species), human and rhesus monkeys (primates), dogs and cows (placental mammals), and chickens (avian, vertebrate outgroup) from the Multiz alignment. Multiz aligns pairwise Blastz sequences to the reference genome and assigns each region a conservation score. Tracks of other vertebrate sequences in the genome browser provide a specific indication of which individual nucleotides change among vertebrate species [10]. The Phylo-P algorithm track was also used to measure conservation by testing whether each nucleotide in the sequence was substituted at a rate faster or slower than expected from neutral genetic drift [50]. The miRNAs were considered conserved among vertebrates if the average identity between the top four alignments was > 80%. Potential species-specific miRNAs, where pre-miRNA mapped to a poorly conserved region of other vertebrate genomes, were assessed in the UCSC genome browser by aligning all available mouse strains with a cactus analysis, an algorithm such as Multiz [49].

### miRNA RT-qPCR

We used the MystiCq microRNA cDNA Synthesis Kit (Sigma) to prepare cDNA. PolyA Tailing and cDNA synthesis reactions were carried out per the manufacturer’s instructions using 1 μg RNA as input, adding 0.5 μL of 5 nM *cel-miR-39* spike-in oligo to the PolyA Tailing reaction mix to act as an exogenous reference gene. Negative controls were generated using polyA-tailed RNA that did not contain reverse transcriptase during cDNA synthesis. Next, 1 μL of microRNA cDNA (diluted 1 in 2 in mqH_2_O) was added to 5 μL SYBR Green MasterMix (ThermoFisher), 0.75 μL each primer, and 2.5 μL mqH_2_O in a 96-well plate, where the two primers consist of (a) a miRNA-specific forward primer designed in IDT PrimerQuest (Table S1), and (b) a universal reverse primer provided in the MystiCq Kit. The primers were tested for their specificity and efficiency. Each sample was loaded in triplicates. The 96-well plate was loaded into a Viia7 PCR Machine (ThermoFisher) for the RT-qPCR reaction with the following thermal profile: 2 min at 50 °C, 2 min at 95 °C, 40 cycles of 15 s each at 95 °C, 60 °C and 72 °C, followed by a machine-programmed dissociation curve. Expression was normalised to the reference gene *cel-miR-39* using the *2*^*−ΔCt*^ method.

### miRNA seed sequence family analysis

To test whether novel miRNAs belong to existing miRNA families, the mature sequence of each novel miRNA was input into the miRBase BLASTn search function with no species filter [39]. This search function output is an *E*-value, which indicates whether sequence similarity is likely to arise because of true sequence homology rather than chance. An E-val < 1 is considered a genuine hit, whereas an increasing E-val suggests that sequence homology is due to chance. The *E*-value threshold for our analysis was set to 10, and an arbitrary but high threshold was set to identify any low-confidence hits that may have a conserved seed region.

### Functional prediction

Firstly, target genes for each candidate novel miRNA were ascertained from two databases with the functionality to enter customized sequences: TargetScan Custom (v.5.2) was used to predict target gene-based complementarity between the seed sequence [22], and miRDB custom prediction was also used, selecting “mouse” as the species [63]. Next, the list of predicted targets from each tool was compared using GeneVenn (http://genevenn.sourceforge.net/) to determine the target genes that were common to both prediction methods, which minimizes the inclusion of false positives. The target gene list for each novel miRNA was then searched using DAVID [16] to determine whether genes in that list were over-represented in any KEGG pathway [34].

## Results

### Novel miRNA discovery in the embryonic mouse brain

Six small RNA sequencing libraries generated from E15.5 mouse brain tissue (*n* = 3/sex) were pooled to improve read depth for novel miRNA discovery (GEO: GSE211816; [59]. The combined FASTA file contained 458,470,358 raw reads (Fig. 1A, which, according to the most stringent estimates, is sufficient for novel discoveries (Raabe et al; Sims et al. [62]. miRDeep2 analysis confirmed that this dataset was representative of the E15.5 mouse brain, identifying miRNAs that are highly expressed in the developing mouse brain (Supplementary File1miRDeep2output.pdfx).File 1) The novel discovery module of miRDeep2 predicted 894 candidate miRNAs, 115 reaching the previously determined miRDeep2 score threshold of 4 [17, 31]. The final filtering step required sequences to demonstrate a stable RNA secondary structure, as indicated by a significant randfold calculation. Randfold measures RNA folding using MFE (maximum free energy estimates). In the context of miRNA, randfold refers to the likelihood that the predicted pre-miRNA sequence can fold into a stable RNA secondary structure (Fig. 1D). For example, 50 of 115 candidates met the miRDeep score threshold and were likely to form a stable RNA hairpin (Fig. 1A).

To obtain more information about these 50 putative novel miRNAs, their genomic locations and the DNA strand from which they originated were determined. Of the top 50 novel miRNAs, 22 mapped to introns, 8 to intergenic regions, 6 to LTRs and 4 to promoters, 3 to exons, and 2 to SINEs. One each mapped to 3’UTR, TTS, LINE, scRNA and snRNA (Fig. 1E). This finding is consistent with most known miRNAs being either intronic or intergenic [7].

### Validation and spatiotemporal expression of novel miRNAs

**Fig. 2:**
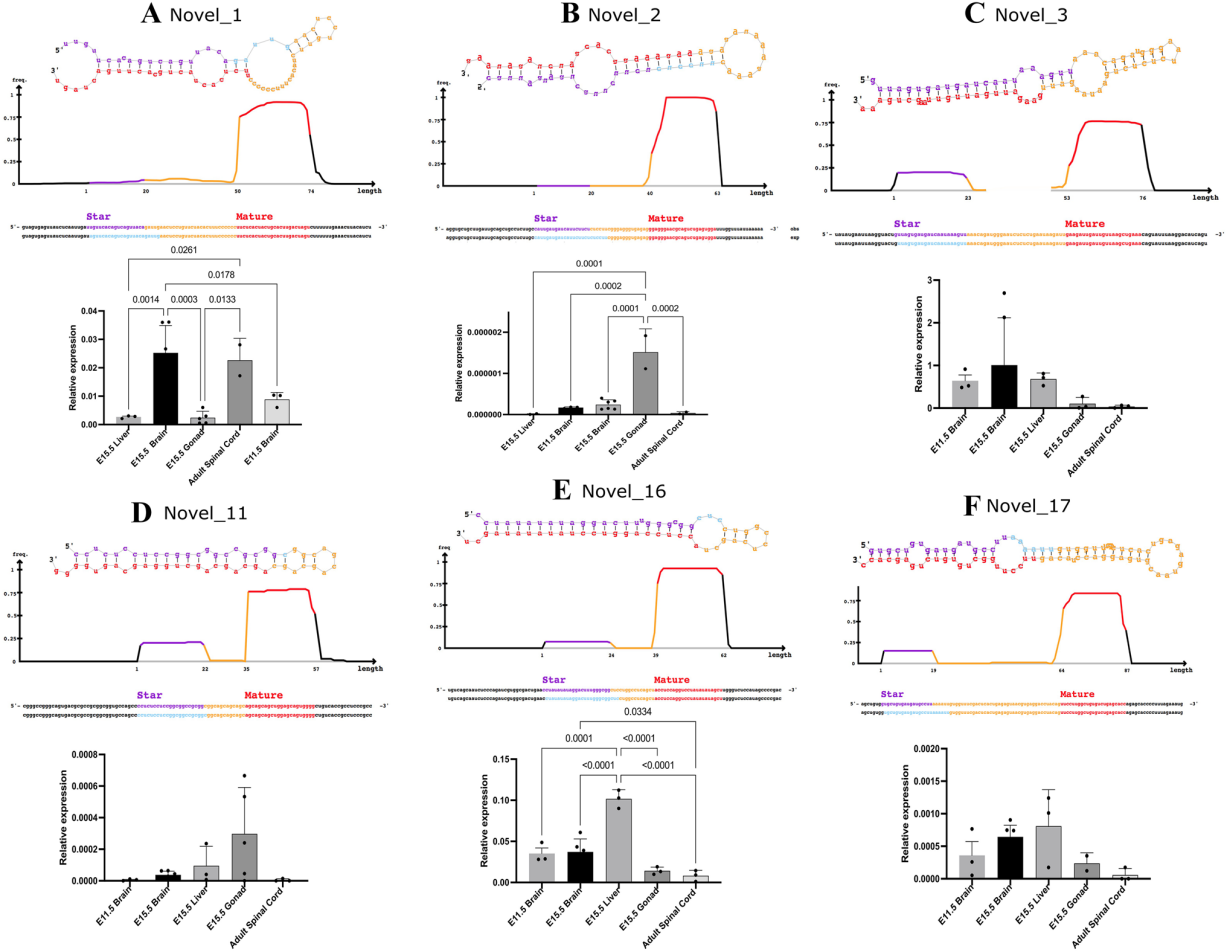
miRNA signature and RT-qPCR validation of the six candidate miRNAs. **A** Novel_1. **B** Novel_2. **C** Novel_3. **D** Novel_11. **E** Novel_16. **F** Novel_17. miRDeep2 hairpin and read signature, where colours indicate whether the sequence is predicted to be a star sequence (purple), loop (yellow), or mature (red). RT-qPCR relative to cel-miR-39 spike-in reference gene in seven tissues: E11.5 brain (*n* = 3), E15.5 brain (*n* = 6), adult spinal cord (*n* = 3), E15.5 liver (*n* = 3), and E15.5 gonads (*n* = 3). Bars indicate mean relative expression using SEM. Statistical significance was determined using one-way analysis of variance (ANOVA) with Tukey’s test for multiple comparisons

RT-qPCR validation was performed on six putative novel miRNAs, selected for the highest ranked miRDeep2 score, and based on a miRDeep2 score > 2 × 10^2^ and mapped to only one location in the mm9 genome (File S1, Fig. 2), allowing the design of specific primers. The expression of each miRNA was profiled in six different tissues.

*Novel_1* was the sequence with the highest miRDeep2 score (2800) and a significant randfold score in the small RNA-seq library that did not map to a known miRNA, with a high read count of > 53,000 reads for the mature miRNA (File S1). Expression analysis revealed *Novel_1* expression level varied with the tissue type tested (one-way ANOVA; *p* = 0.0001) (Fig. 2A). The highest relative expression was observed in the neuronal samples (E11.5 and E15.5 brain) and the adult spinal cord. In comparison, there was modest expression in the E15.5 liver and minimal amplification of *Novel_1* in the E15.5 gonads. In addition*, Novel_1* expression was significantly higher in the brain than in non-neural tissues (liver, *p* = 0.0014; gonad, *p* = 0.0003) and the E11.5 brain (Fig. 2A *p* = 0.012).

Of the novel miRNA candidates identified by miRDeep2, *Novel_2* demonstrated one of the highest read counts (> 18,000), in addition to a characteristic read distribution and pre-miRNA hairpin (Fig. 2B; File S1). *Novel_2* expression was detectable, although it varied greatly between biological replicates, and there was no significant difference in the means between tissues (one-way ANOVA; *p* = 0.29).

*Novel_3* was an abundant transcript with characteristic hairpin and miRNA signature characteristics (Fig. 2C). *Novel_3* miRNA expression was significantly different between tissues (one-way ANOVA; *p* < 0.0001). Its expression was significantly higher in the E15.5 liver than in all other tissues assayed (Fig. 2C; *p* < 0.0001). However, relative expression was modest in the brain at E11.5 and E15.5, and it was detectable in the adult spinal cord and E15.5 gonads (Fig. 2C).

*Novel_11* fits the criteria for miRDeep2 pre-miRNA hairpin formation and miRNA signatures (Fig. 2D). The coordinates for *Novel_11* in the mm9 genome are located within an intron of nucleolar protein 4-like (*8430427H17Rik*) gene. Amplification was detected across all assayed tissues for *Novel_11*, however, there was no significant differences in expression across tissues. Expression was higher in the gonad for two of the biological replicates (Fig. 2D), this was not consistent and was not dependent on gonadal sex (data not shown).

*Novel_16* demonstrated characteristic hairpin stability and read coverage signature of a mature miRNA (Fig. 2E). Alignment to mm9 shows that *Novel_16* is located near Empty Spiracles Homeobox 2 (*Emx2*), a gene that aids in determining the rostral-caudal patterning of the developing neocortex [9]. *Novel_16* was also enriched in E15.5 gonads compared to all other tissues (*p* < 0.0001). Modest *Novel_16* expression was detected in the assayed embryonic brain tissues, and there was a significant difference in expression based on tissue type (one-way ANOVA; *p* = 028) (Fig. 2E).

*Novel_17* maps to chromosome 17, within the intron of the *Wdr43* (WD40 repeat 43) gene, which codes for an RNA-binding protein [8]. It was predicted to form a stable pre-miRNA hairpin, with an miRDeep2 score of 240, and a typical miRNA read distribution with a modest read count of 403 for the mature miRNA (Fig. 2F; File S1). There were no statistically significant differences in expression between groups (Tukey’s multiple comparison test, *p* > 0.05). The E15.5 brain showed consistent expression of *Novel_17* between biological replicates, and the relative expression in other tissues was very variable (Fig. 2F).

### Predicting candidate miRNA function

A measure of miRNA functionality can be obtained by investigating the relationships between novel miRNA sequences and existing miRNAs and miRNA families. miRNA families are defined by similarities in structure and function, particularly in seed sequences, because they are derived from common ancestral sequences [33]. A novel miRNA that shares a seed sequence with an existing miRNA family indicates shared functionality and helps to validate putative novel miRNAs. Functional prediction was performed via pathway analysis of the predicted target genes to infer the possible function of each novel miRNA. The full results for target prediction and functional enrichment analysis can be found in Supplementary Data File S4.

**Fig. 3:**
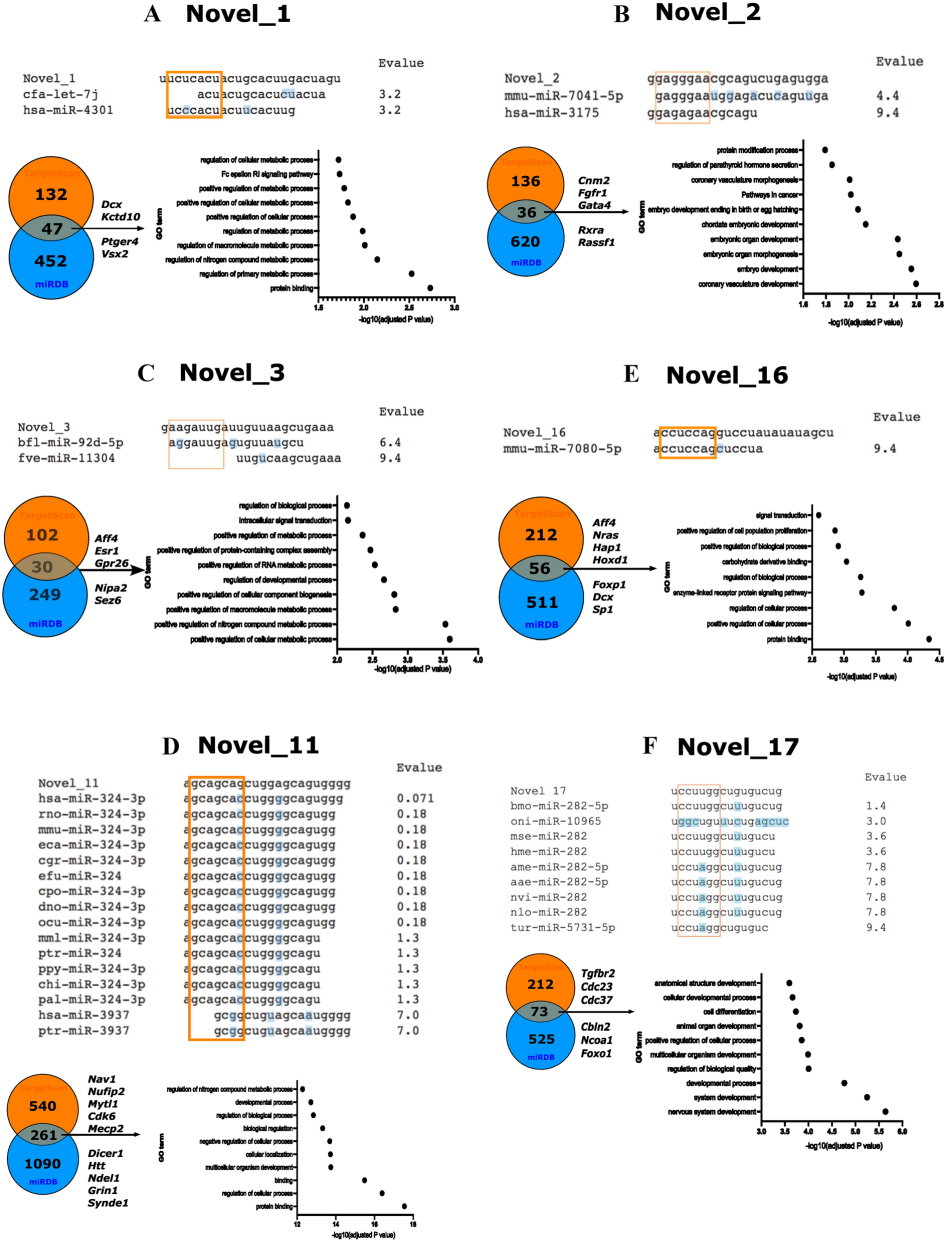
Functional prediction for six candidate miRNAs. **A** Novel_1. **B** Novel_2. **C** Novel_3. **D** Novel_11. **E** Novel_16. **F** Novel_17. The miRNA seed sequence family analysis identified annotated miRNAs in other species with similar seed sequences. They were ranked with the lowest E-values. Orange box = seed sequence. Blue highlighting = base-pair mismatch. Target gene identification from two tools (TargetScan and miRDB). The Venn diagram indicates the mRNAs identified by both tools, and the selection of target genes with neurodevelopmental roles is listed. gProfiler GO enrichment analysis for predicted target genes, the ten significantly enriched pathways by *p*-adjusted values

*Novel_1* seed sequence in miRBase found no hits (E-val < 1); therefore, it is not a member of any known miRNA family. The 47 *Novel_1* predicted mRNA targets were significantly associated with biological processes primarily related to metabolism and development (Fig. 3A; File S4). Three *Novel_1* predicted targets were linked to the ErbB signalling pathway (KEGG ID: 04012; *p* = 0.038). ErbB signalling is vital for forming neural circuitry and has been implicated in neuropsychiatric disorder pathology (Brinkman et al., Mei et al.). A particularly interesting target gene is *Dcx* (Doublecortin), which is required for normal migration of neurons in cortical development and mutated in the brain disorder lissencephaly [35]. As *Novel_1* appears to target genes and pathways critical to neurodevelopment, *Novel_1* expression may influence crucial processes in the embryonic mouse brain.

The *Novel_2* seed comparison identified two mammalian miRNAs with similar seed sequences. However, both had E-value BLAST score of > 1, suggesting the similarity could be due to chance rather than an evolutionary relationship. TargetScan and miRDB identified 36 overlapping target genes of *Novel_2* (Fig. 3B and File S4). GO analysis revealed an over-representation of terms related to vascular and embryonic development (Fig. 3B and File S4). Targets of note included cell-cycle-related *Cnnm2* (cyclin M2), growth factor receptor *Fgfr1* (fibroblast growth factor receptor 1), oncogene-related *Rassf1* (Ras association domain family member 1), and *Rxra* (Retinoid X Receptor A). These targets suggest that *Novel_2* may have a role in regulating cell growth and proliferation in the developing brain.

For *Novel_3*, seed sequence analysis revealed no evidence for shared similarity with known vertebrate miRNAs (Fig. 3C). Only 30 predicted target mRNAs overlapped between TargetScan and miRDB (Fig. 3C). However, GO analysis still found significant enrichment for metabolic processes, signal transduction and developmental processes (Fig. 3C, File S4). Some of these genes themselves are of interest in brain development specifically, *Nipa2* (non-imprinted in Prader-Willi/Angelman syndrome 2), *Sez6* (seizure-related 6 homologs), and *Aff4* (AF4/FMR2 family member 4) are involved in neurodevelopmental disorders with complex phenotypes [12, 32, 47, 48, 64]. These findings suggest that *Novel_3* could be linked to the genetic regulatory circuits that underlie neurodevelopment.

The *Novel_11* seed also showed high similarity to *miR-324-3p*, with significant hits (E-value < 1) in nine species (Fig. 3D). This suggests that *Novel_11* is a previously unidentified member of the *miR-324* family. The function of miR-324 function has been described in mouse brain development, with established functions in astrocyte-mediated synaptogenesis and subsequent effects on neuronal function and excitability [29, 57]. Functional prediction yielded a pool of 261 putative target genes, including several with notably brain-related functions: *Nav1* (neuron navigator 1), *Myt1l* (myelin transcription factor 1-like), *Syde1* (synapse defective 1 Rho GTPase homolog 1), *Htt* (Huntingtin) and *Ndel1* (nudE neurodevelopment protein 1 like 1). Other target genes of note are associated with neuronal physiology (*Grin1*, *Cacnb1*, *and Cacna1c*) and cell cycle regulation (*Cdk6*, *Cdca4*, *and Caprin1*). Finally, genes such as *Mecp2* (methyl CpG binding protein 2) and *Nufip2* (nuclear fragile X mental retardation protein interacting protein 2) are essential for normal neurodevelopment [4, 25]. GO analysis revealed many enriched terms among *Novel_11* target mRNAs. Many of the most significant hits related to development (“multicellular organism development”) included nervous system development (GO: 0007399) (Fig. 3D, File S4). Based on the target gene prediction, *Novel_11* may influence a range of genes involved in mouse brain development.

*Novel_16* is not a member of any known miRNA family, as the miRNA family analysis yielded no significant BLAST alignment (*E*-value < 1; Fig. 3E). Target prediction identified 56 overlapping potential mRNAs for *Novel_16*. Genes with roles in neurodevelopment were consistent among the target mRNAs (*Aff4*, *Nras*, *Hap1*, *Dcx*) (File S3). Overrepresented GO terms included “positive regulation of cell proliferation” and “vesicle transport along microtubules”. Thus, *Novel_16* has the potential to regulate processes essential for neurodevelopment and neuronal function.

For *Novel_17*, miRNA family analysis found hits with the seed sequences of 7/9 members of the *miR-282* family (Fig. 3F). This finding suggests that *Novel_17* diverged from an ancestor of the *miR-282* family or, based on the low E-val reported, that seed sequence similarity emerged by chance. We identified 73 predicted targets for *Novel_17* (Fig. 3F, File S3). The top significant GO term significantly overrepresented was “nervous system development (GO: 0007399); other terms were mostly related to developmental biology (Fig. 3F, File S4). Individual target genes reflect a potential role for *Novel_17* in developmental processes. Cell cycle functions (*Cdk6*, *Cdc27*, *Cdc23*), developmental pathways (*Tgfbr2*) and neurodevelopment-related genes (*Cbln2*, *Syt9*) all provide potential mechanistic bases for *Novel_17* to impact mouse brain development.

### Variable conservation among novel miRNAs

**Fig. 4:**
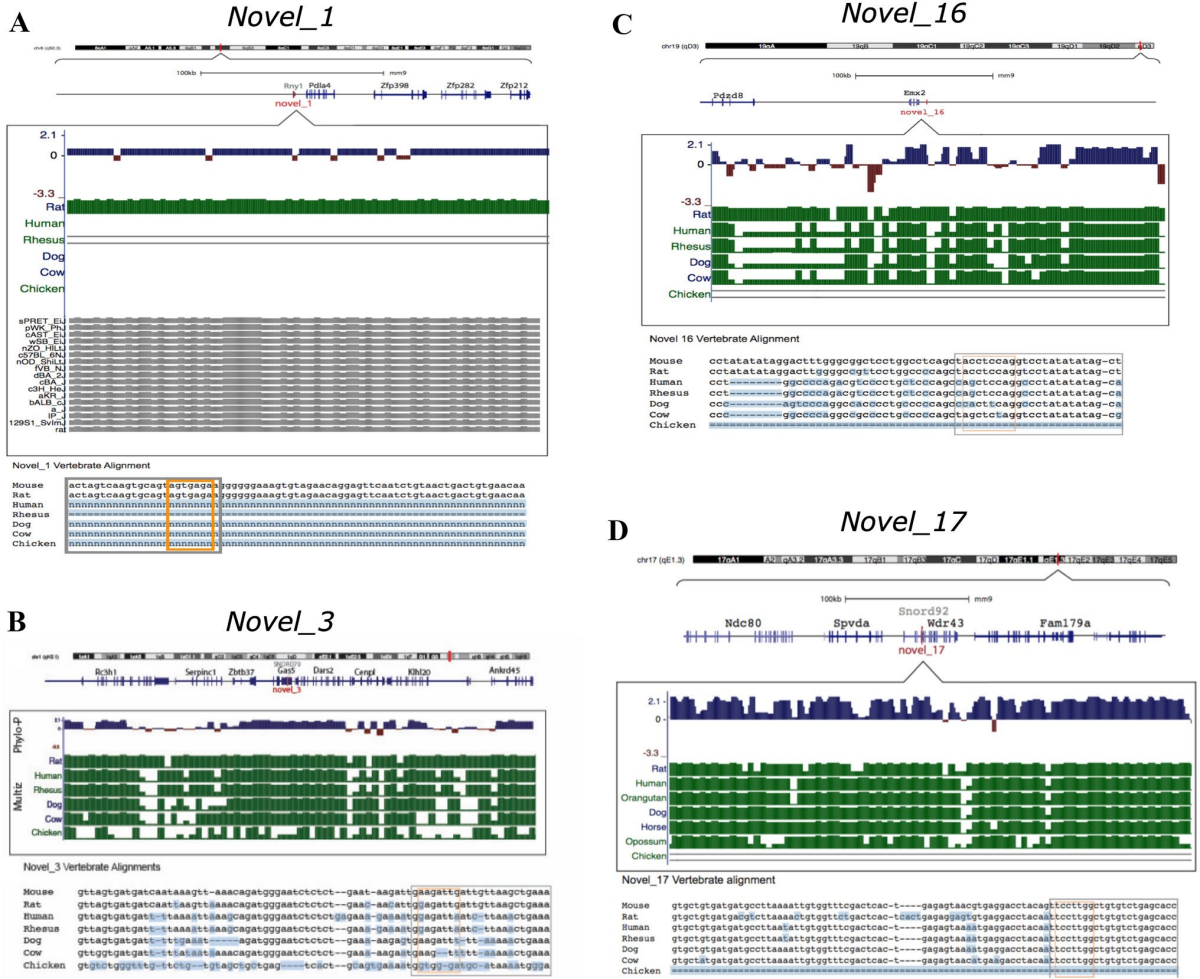
Example of PhyloP and MultiZ alignment for four novel miRNAs. **A** Pre-Novel_1 actus alignment on chromosome 6, with alignment to different mouse strains. **B** Alignment of pri-Novel_3 locus on chromosome 1. **C** Novel_16 on chromosome 19 near the Emx2 gene. **D** Novel_17 pre-miRNA was aligned to an intron within the Wdr43 gene on chromosome 17. Changes from mm9 are highlighted in blue. “=” indicates no sequence alignment at that position. Grey box = mature miRNA sequence. Orange box = seed sequence

Conservation analysis was performed for the six candidate miRNAs to determine whether each sequence was unique to mice or present in other species (Fig. 4).

**Fig. 5:**
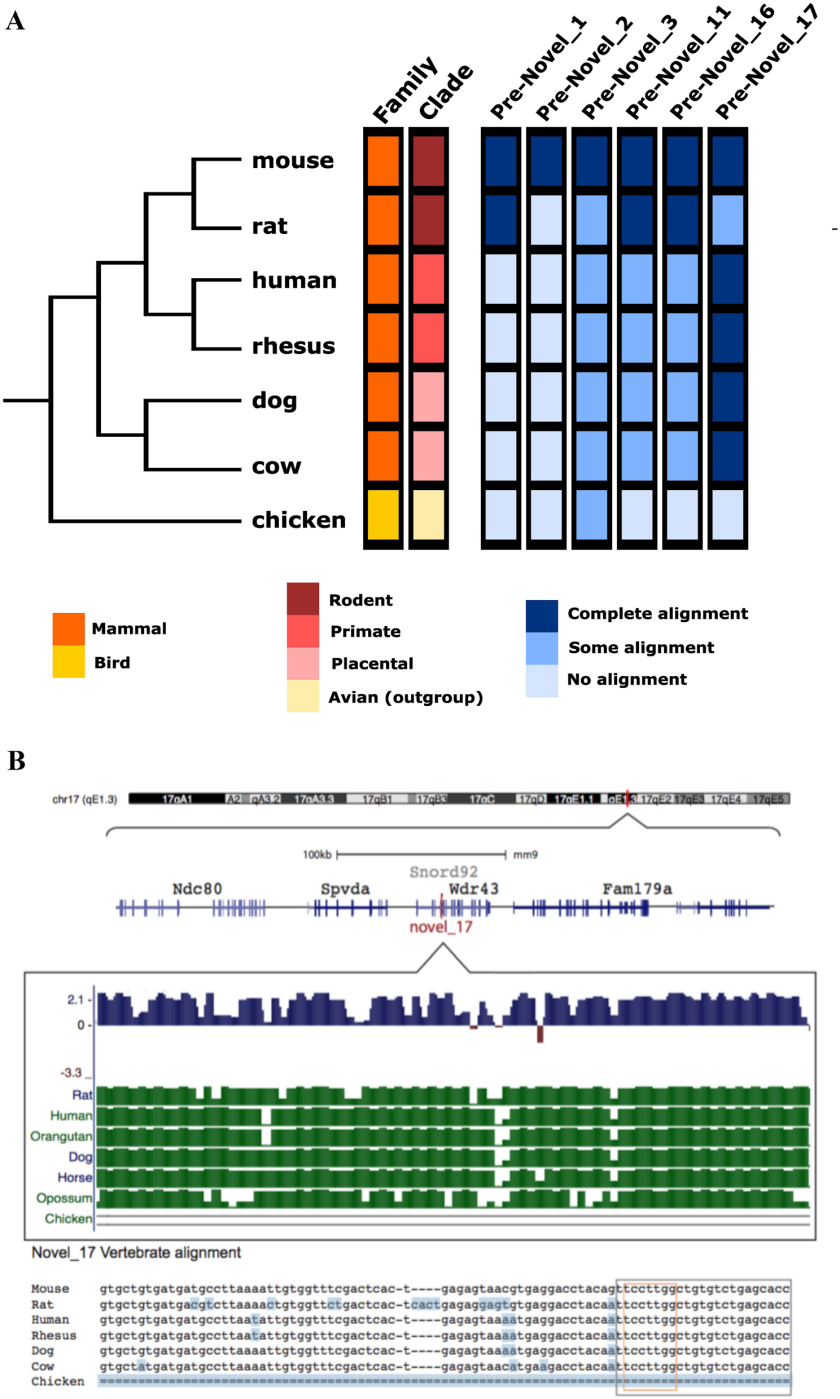
Conservation of novel miRNAs. **A** Summary of pre-miRNA sequence alignments across seven vertebrate species. The phylogenetic tree approximates relatedness, and red/orange/yellow indicates key species attributes and family groupings. Blue panels indicate the extent of base pair alignment of mouse pre-miRNAs in each species, where a darker blue colour indicates greater sequence homology. **B** Example of MultiZ and PhyloP alignments for pre-novel _17. Changes from mm9 are highlighted in blue. “=” indicates no sequence alignment at that position. Grey box = mature miRNA sequence. Orange box = seed sequence

The pre-*Novel_1* sequence has 100% identity in mouse and rat genomes but does not match the sequences of other vertebrate genomes. The phylo-P algorithm gives a primarily positive score throughout the sequence, which is likely driven by rat conservation. Conservation analyses suggested that *Novel_1* is probably a rodent-specific miRNA (Fig. 4A & 5A).

Although the pre*-Novel_2* sequence failed to align with any other vertebrate genomes, it aligned with > 99% identity with other mouse strains (Fig. S5). Therefore, *Novel_2* is potentially a recently emerged mouse-specific miRNA (Fig. 5A).

Pre-Novel *_3* is located within an intron of the lncRNA growth arrest-specific transcript 5 (*Gas5*) gene, a lncRNA tumour suppressor [19] (Fig. 4B). *Gas5* notably hosts several other small RNAs within its introns [56]. Pre-Novel *_3* identity was ~ 80% among all vertebrates and has a positive phylo-p score, indicating slower evolution than expected, which is consistent with sequence conservation. Furthermore, the mature miRNA and seed sequences are increasingly retained in closely related species, suggesting a functional miRNA in many mammals that may be a conserved aspect of the *Gas5* locus (Fig. 5A).

Pre*-Novel_11* had a positive phylo-p score, which indicates a slower evolution than expected. This is consistent with the sequence conservation and demonstrates approximately 87.5% identity among vertebrates (Fig. S5). While the seed sequence showed 100% conservation in rat, human, rhesus, and cow genomes, unique indels in the seed regions of both dog and avian outgroups likely rendered non-functional in those species (Fig. S5). Therefore, *Novel_11* may be a miRNA in mammalian species (Fig. 5A).

*Pre-Novel_16* is generally highly conserved among mammals, averaging ~ 80% identity, but no alignment to the avian genome. Conservation of the mature *Novel_16* sequence is also high among mammalian genomes (Fig. 4C). However, there was greater variability in the phylo-p score throughout the precursor sequence (Fig. 4C). This may be attributable to an 8 bp insertion in the predicted passenger strand of the primate sequence (Fig. 4C). These findings suggest that *Novel_16* is a functional miRNA in non-primate mammals (Fig. 5A).

Novel_17 conservation analysis indicated ~ 94% pre-miRNA sequence identity between all mammalian species and 100% seed sequence identity between all mammals, with no alignment with the avian genome (Fig. 4D & 5B). While the positive phylo-p score indicates slower evolution than expected, consistent with sequence conservation, there seems to be some divergence between rodent species: the rat sequence shows only 87.21% identity, making it the most different from mouse and other mammalian species (Fig. 4D & 5B). This suggests *that Novel_17* is likely a functional miRNA in mammals, but its sequence has diverged in the rat (Fig. 5A).

## Discussion

Using robust small RNA-seq datasets generated from the E15.5 mouse brain, an unbiased miRNA discovery strategy identified 50 putative novel miRNAs, six of which were further validated. In addition, a combination of conservation analysis and mRNA target prediction was used to determine the authenticity of the six miRNA candidates. These findings demonstrate that miRNAs tend to remain undiscovered if they are derived from other RNA (ncRNA).

### Conservation and function of candidate novel miRNAs

The novel miRNAs identified here were previously undiscovered components of the instruction manual for mouse brain development. Genomic networks that inform the processes of neurodevelopment are enormously complex. Given this overall complexity, exploring the role of any individual miRNA, especially one that has been discovered to date, could be dismissed as of little importance. However, subtle changes underscore much of the typical and pathological variations seen in the brain, often as additional components of networks that ensure robust phenotypes that buffer differences in gene expression or the environment [14, 51]. Together with the predicted conservation of several candidate novel miRNAs in humans (*Novel_3*, *Novel_11*, *Novel_17*), these novel miRNAs represent candidates whose functions are required for neurodevelopment and whose dysfunction contributes to neurodevelopmental disorders. In contrast, the other validated candidates did not appear to be conserved in humans, suggesting that these sequences are either non-functional or rodent-specific. However, their function is still worth studying to improve our understanding of the commonly used mouse model and for an evolutionary perspective on why they have not been retained in humans.

### Dual small RNA function

Three validated miRNAs map to known ncRNAs in the mouse genome: Novel_1 aligns with *Rny1*, Novel_3 to *Snord79*, and Novel_17 to *Snord_92*. One would initially assume these are false positives, mistakenly identified by the miRDeep2 algorithm due to shared features between different ncRNA classes, such as read length and stable secondary structure formation [11]. However, these candidates should not be discounted; multiple publications have previously encountered instances where a single ncRNA transcript may perform the function of a snoRNA, for example, in addition to generating a mature miRNA. Four pieces of evidence support this phenomenon, known as dual RNA function: (1) ~ 22nt sequences derived from snoRNAs associate with Ago proteins, meaning they guide downregulation of target mRNA via the RISC complex [18]; (2) snoRNA-derived fragments are Dicer-dependent, thereby sharing a critical step in biogenesis with pre-miRNAs [60]; and (3) reads derived from snoRNAs showed a high abundance of one stem, with lower abundance for its complementary stem and the loop [18]. This non-random degradation pattern is consistent with the typical miRNA signature but atypical for snoRNAs. Additionally, (4) small RNAs derived from snoRNA can silence target genes in luciferase reporter assays [11]. The possibility of dual RNA function might explain the three mentioned examples where a candidate novel miRNA overlaps with a known ncRNA.

Novel_3 and Novel_17 both mapped to snoRNAs. Whether they could function as novel miRNAs, in addition to their previously described function, depends on whether they can demonstrate any of the four characteristics outlined above. The data presented here cannot confirm the downregulation of any target genes or Dicer-dependence. The small terminal loop size observed for Novel_3 and Novel_17 compared to the canonical terminal loop size suggests Dicer-independent biogenesis of an atypical precursor via non-canonical processing [27]. In contrast, both candidate novel miRNAs demonstrated a typical miRNA signature consisting of an abundant mature sequence compared with low expression of star and loop sequences.

It has also been suggested that miRNAs can be derived from Y-RNAs, a group of small RNA that associate with Ro-proteins to form ribonucleoproteins (RNPs) [43]. This claim is based on structural similarities with miRNA precursors [61]. However, it has not been confirmed that small sequences derived from Y-RNAs downregulate target gene expression [46]. Without evidence that they function as miRNAs or show arm-specific stabilisation/degradation patterns expected of miRNAs [53], there remains uncertainty regarding whether functional miRNAs can be derived from Y-RNAs. Whether Novel_1 is a mature miRNA derived from Rny1 is conflicting. First, the predicted secondary structure of pre-Novel_1 was similar to that of typical Y-RNA features, including a bulged cytosine ([38]. In contrast, Novel_1 shows a miRNA read signature with the preferential stability of one stem strand. Further experiments are required to determine whether Novel_1 has a Y-RNA function via interaction with Ro-proteins, miRNA function, interaction with RISC components, downregulation of target genes, or both.

This highlights the possibility that novel miRNA candidates that map to known ncRNAs should not be immediately discarded as false positives. Although these claims are limited without further functional assays, up to three top candidates can demonstrate dual functions of small RNA. These examples provide an emerging body of evidence regarding the complex and diverse roles of ncRNAs. Indeed, excluding candidates on this basis presumably underlies why these RNAs have escaped discovery and continues to keep other miRNAs undiscovered.

### Limitations and future directions

A vital advantage of the novel miRNA discovery strategies utilized here is the consideration of multiple types of evidence (bioinformatics prediction, conservation, expression, and functional prediction) to predict whether a small RNA transcript is a candidate novel miRNA. However, to ensure that each candidate is a genuine novel miRNA, further experiments should be performed to distinguish it from other RNA species and determine their function; the former is particularly important given the potential for dual small RNA function. For example, in vitro knockdown by antagomiRs could help to reveal the role of each novel miRNA. Target pull-down methods can also be used to identify specific miRNA target interactions in vivo [3, 15].

In addition to confirming that each candidate carries out fundamental miRNA function and downregulation of target genes, we also wanted to distinguish these sequences from other ncRNA species, many of which share the characteristic features of miRNAs. This could be achieved by exploiting the characteristics that differ between each RNA species, yet the possibility of dual small RNA function complicates it. Transcripts that fit the criteria for more than one RNA category force our understanding of these categories to transcend the disparate classical grouping into a more fluid sense of complex RNA functions. A general approach requires experiments to confirm each type of predicted function. In addition to demonstrating the downregulation of target genes as per miRNA function, characteristics unique to snoRNAs/Y-RNAs/etc. must be confirmed. Such a challenging experimental design has so far resulted in very few efforts being made to tease apart dual RNA functions.

A notable strength of our novel miRNA discovery strategy is the intentional sample inclusion from E15.5 brains of both sexes. Other studies, including our own (Szakats et al., manuscript in review), have shown sex-based differences in miRNA expression in the developing mouse brain. To account for any novel miRNAs that may be differentially expressed between the sexes, we ensured that tissue samples from both sexes were included. Failure to report the sex of samples is a common flaw in many previous studies, as it may result in false negatives in the scenario where a miRNA is highly expressed in females but not males, yet only males are sampled in the study, or vice versa. Increased recognition of sex-biased genes and miRNA expression should improve the use of this strategy to include both sexes in the study design, resulting in novel discoveries that were previously missed.

## Conclusion

We have presented the first novel miRNA discovery experiment conducted in the embryonic mouse brain over the last decade. Improvements in bioinformatics discovery tools in the intervening years and the very high read depth that we obtained contributed to generating a highly powered dataset. We identified 50 and further validated six putative miRNAs from these data, which have not been previously reported. Bioinformatics prediction, sequence conservation, temporal expression, and functional prediction provide crucial evidence. Combining several types of evidence suggests that the ten candidate miRNAs investigated are genuine novel miRNAs. The discovery and successful validation of new miRNAs augments our current understanding of ncRNA regulation in mouse brain development and provides new avenues to explore this complex process. Moreover, we have provided compelling recent examples of dual small RNA function. This intriguing phenomenon has only been validated on a handful of occasions and, with the additional evidence presented here, may generate a thought-provoking discussion regarding the delineation of RNA species.

### Supplementary Information

Below is the link to the electronic supplementary material.

miRDeep2 output. Supplementary file1 (PDF 16839 KB)

miRDeep2 output and RNA secondary structure. Supplementary file2 (PDF 96 KB)

RT-qPCR primer sequences. Supplementary file3 (XLSX 267 KB)

Target gene prediction. Supplementary file4 (XLSX 71 KB)

gProfiler GO analysis for the target genes of six candidate miRNAs. Supplementary file5 (PDF 458 KB)

Conservation analysis for candidate miRNAs. Supplementary file6 (PDF 40 KB)

## Data Availability

Enquiries about data availability should be directed to the authors.
