# Supplementary material for: Identification of novel microRNAs in the embryonic mouse brain using deep sequencing
Source: Mol Cell Biochem. 2023 Apr 15;479(2):297–311. doi: 10.1007/s11010-023-04730-2 (PMC10890980; doi:10.1007/s11010-023-04730-2)
Supplement: Supplementary file 1 — miRDeep2 output. Supplementary file1 (PDF 16839 KB) [file 11010_2023_4730_MOESM1_ESM.pdf]

miRDeep2

miRDeep home

Parameters used

miRDeep2 version	2.0.0.8
Program call	/home/utilities/miniconda2/envs/mirdeep/bin/miRDeep2.pl collapsed_combined_files.fasta /scratch/susie/genome/mir_allchr.fa converted_mapout.arf mus_mature_conv.fa none mus_precurs_conv.fa -t mmu 2
Reads	collapsed_combined_files.fasta
Genome	/scratch/susie/genome/mir_allchr.fa
Mappings	converted_mapout.arf
Reference mature miRNAs	mus_mature_conv.fa
Other mature miRNAs	none
Species	mmu

Survey of miRDeep2 performance for score cut-offs -10 to 10

	novel miRNAs			known miRBase miRNAs				
miRDeep2 score	predicted by miRDeep2	estimated false positives	estimated true positives	in species	in data	detected by miRDeep2	estimated signal-to-noise	excision gearing
10	115	45 ± 7	70 ± 7 (61 ± 6%)	1915	1144	602 (53%)	8.5	28
9	115	45 ± 7	70 ± 7 (61 ± 6%)	1915	1144	602 (53%)	8.5	28
8	115	45 ± 7	70 ± 7 (61 ± 6%)	1915	1144	602 (53%)	8.5	28
7	115	45 ± 7	70 ± 7 (61 ± 6%)	1915	1144	602 (53%)	8.5	28
6	115	45 ± 7	70 ± 7 (61 ± 6%)	1915	1144	602 (53%)	8.4	28
5	115	45 ± 7	70 ± 7 (61 ± 6%)	1915	1144	602 (53%)	8.4	28
4	115	45 ± 7	70 ± 7 (60 ± 6%)	1915	1144	602 (53%)	8.4	28
3	131	47 ± 7	84 ± 7 (64 ± 5%)	1915	1144	604 (53%)	8.4	28
2	522	100 ± 9	422 ± 9 (81 ± 2%)	1915	1144	784 (69%)	7.7	28
1	795	574 ± 18	221 ± 18 (28 ± 2%)	1915	1144	845 (74%)	2.1	28
0	894	2587 ± 34	0 ± 0 (0 ± 0%)	1915	1144	848 (74%)	0.5	28
-1	1126	3310 ± 38	0 ± 0 (0 ± 0%)	1915	1144	851 (74%)	0.5	28
-2	2441	3938 ± 38	0 ± 0 (0 ± 0%)	1915	1144	912 (80%)	0.7	28
-3	3175	4281 ± 40	0 ± 0 (0 ± 0%)	1915	1144	929 (81%)	0.8	28
-4	3597	4494 ± 39	0 ± 0 (0 ± 0%)	1915	1144	937 (82%)	0.9	28
-5	3892	4638 ± 41	0 ± 0 (0 ± 0%)	1915	1144	937 (82%)	0.9	28
-6	4108	4746 ± 40	0 ± 0 (0 ± 0%)	1915	1144	937 (82%)	1	28
-7	4262	4834 ± 40	0 ± 0 (0 ± 0%)	1915	1144	938 (82%)	1	28
-8	4364	4917 ± 41	0 ± 0 (0 ± 0%)	1915	1144	940 (82%)	1	28
-9	4476	4966 ± 42	0 ± 0 (0 ± 0%)	1915	1144	940 (82%)	1	28
-10	4557	5017 ± 42	0 ± 0 (0 ± 0%)	1915	1144	940 (82%)	1	28

novel miRNAs predicted by miRDeep2

provisional id	miRDeep2 score	estimated probability that the miRNA candidate is a true positive	rfam alert	total read count	mature read count	loop read count	star read count	significant randfold p-value	miRBase miRNA	example miRBase miRNA with the same seed	UCSC browser	NCBI blastn	consensus mature sequence	consensus star sequence	consensus precursor sequence	precursor coordinate
gi|307603380|gb|CM000996.1|_29607	2.9e+4	0.61 ± 0.06		58821	58800	0	21	no				blast	ucccggguuucggcacca	gaguuggaauuuugaugggg	ucccggguuucggcaccaaagaauugaguuggaauuuugaugggg	gi|307603380|gb|CM000996.1|:7834169..7834
gi|307603377|gb|CM000999.1|_38372	2.8e+4	0.61 ± 0.06		55617	53812	1331	474	yes				blast	uucucacuacugcacuugacuagu	uuguucacagucaguuaca	uuguucacagucaguuacagauugaacuccuguucuacacuuuccccccuucucacuacugcacuugacuagu	gi|307603377|gb|CM000999.1|:47738072..477
gi|307603380|gb|CM000996.1|_30318	1.3e+4	0.61 ± 0.06	rRNA/tRNA	25652	16269	233	9150	no				blast	uuuaguaguaccgucccuuu	ugguuaguacucugcguu	ugguuaguacucugcguuguggccgcagcaaccucgguucgaauccgagucacggcauuuaguaguaccgucccuuu	gi|307603380|gb|CM000996.1|:96304268..963
gi|307603379|gb|CM000997.1|_31365	1.2e+4	0.61 ± 0.06		24193	23652	540	1	no				blast	ccagccugggcuacacauuuu	gugguggcgcacgcuggu	gugguggcgcacgcugguaggauuugcugaaguaggcagaggcaggaggaucacgaguucgaggccagccugggcuacacauuuu	gi|307603379|gb|CM000997.1|:82975190..829
gi|307603370|gb|CM001006.1|_9631	9.8e+3	0.61 ± 0.06		19402	19325	0	77	no				blast	uaaucugaagguccugagu	cucagagagggcagauguuuu	uaaucugaagguccugaguucgaaccucagagagggcagauguuuu	gi|307603370|gb|CM001006.1|:21418577..2141
gi|307603372|gb|CM001004.1|_3793	9.3e+3	0.61 ± 0.06		18365	18364	0	1	yes				blast	ggagggaacgcagucugagugga	cauugaugaucauucuucu	cauugaugaucauucuucucuccuucgggagggugagagggagggaacgcagucugagugga	gi|307603372|gb|CM001004.1|:95894144..958
gi|307603382|gb|CM000994.1|_22531	3.4e+3	0.61 ± 0.06		6706	5133	230	1343	yes				blast	gaagauugauuguuaagcugaaa	guuagugaugaucaauaaaguu	guuagugaugaucaauaaaguuaaacagaugggaaucucucugaauaagauugaagauugauuguuaagcugaaa	gi|307603382|gb|CM000994.1|:162967490..16
gi|307603370|gb|CM001006.1|_8630	3.3e+3	0.61 ± 0.06		6645	6615	0	30	no				blast	ccagccugggcuacacauuu	cuugggaggcagaggcaggcggau	ccagccugggcuacacauuugccgggcgguggugg cgcacaccuuugaucccagcacuugggaggcagaggcaggcggau	gi|307603370|gb|CM001006.1|:23359862..233
gi|307603373|gb|CM001003.1|_2350	1.2e+3	0.61 ± 0.06		2506	2501	0	5	no				blast	gucacgguggccgagugg	gcgcggccaaccauaguuuu	gcgcggccaaccauaguuuugggagucacgguggccgagugg	gi|307603373|gb|CM001003.1|:127895583..12
gi|307603378|gb|CM000998.1|_33984	1.0e+3	0.61 ± 0.06		2151	1844	0	307	no				blast	ggccaaggaugagaacucuaa	aaaggauuuaccugaggcc	ggccaaggaugagaacucuaaccugacuggauacgcugcugugaugg guuaaaggauuuaccugaggcc	gi|307603378|gb|CM000998.1|:23358028..233
gi|307603378|gb|CM000998.1|_35485	1.0e+3	0.61 ± 0.06		2138	1844	0	294	yes				blast	ggccaaggaugagaacucuaa	aaaggauuuaccugaggcc	ggccaaggaugagaacucuaaccugacaggauacgcugcugugaugg guuaaaggauuuaccugaggcc	gi|307603378|gb|CM000998.1|:23216809..232
gi|307603382|gb|CM000994.1|_22181	5.3e+2	0.61 ± 0.06		1045	994	0	51	yes				blast	uguggaaucccuucaaccuugugg	ucuugguuguacuuggguucuugc	ucuugguuguacuuggguucuugcucagcuguuucuguccugcuguggaaucccuucaaccuugugg	gi|307603382|gb|CM000994.1|:108098754..10
gi|307603371|gb|CM001005.1|_7228	5.1e+2	0.61 ± 0.06		1003	961	11	31	yes				blast	gaccgaugaugagaucugaugg	gaacagauaucugagguc	gaccgaugaugagaucugauggugucugaguguuucucgaugacaugaacagauaucugagguc	gi|307603371|gb|CM001005.1|:110918156..11
gi|307603367|gb|CM001009.1|_14934	4.7e+2	0.61 ± 0.06		941	873	0	68	no				blast	uguaguccuggcuguccugg	aggcuggccucgaacucag	uguaguccuggcuguccuggauuuuacucuggagucuaucuaggcuggccucgaacucag	gi|307603367|gb|CM001009.1|:44190480..441
gi|307603363|gb|CM001013.1|_49774	4.2e+2	0.61 ± 0.06		839	829	0	10	yes				blast	gacugaguaacugcuagauccu	agucuagcucuugcucuugcuc	agucuagcucuugcucuugcucuugcuccuuugccugcuugcugcgugagacugaguaacugcuagauccu	gi|307603363|gb|CM001013.1|:157617245..15
gi|307603372|gb|CM001004.1|_5652	4.2e+2	0.61 ± 0.06		839	829	0	10	yes				blast	gacugaguaacugcuagauccu	agucuagcucuugcucuugcuc	agucuagcucuugcucuugcuccuucgccugcuugcugcgugagacugaguaacugcuagauccu	gi|307603372|gb|CM001004.1|:99683400..996
gi|307603380|gb|CM000996.1|_28729	4.2e+2	0.61 ± 0.06		839	829	0	10	yes				blast	gacugaguaacugcuagauccu	agucuagcucuugcucuugcuc	agucuagcucuugcucuugcuccuucgccugcuugcugcgugagacugaguaacugcuagauccu	gi|307603380|gb|CM000996.1|:76696701..766
gi|307603381|gb|CM000995.1|_26193	4.2e+2	0.61 ± 0.06		839	829	0	10	yes				blast	gacugaguaacugcuagauccu	agucuagcucuugcucuugcuc	agucuagcucuugcucuugcuccuuugccugcuugcugcgugagacugaguaacugcuagauccu	gi|307603381|gb|CM000995.1|:158380708..15
gi|307603370|gb|CM001006.1|_9717	4.0e+2	0.61 ± 0.06		798	747	0	51	no				blast	augccgagguugugaguuc	gccucaccuggagcauuguuu	augccgagguugugaguucgagccucaccuggagcauuguuu	gi|307603370|gb|CM001006.1|:23362283..233
gi|307603381|gb|CM000995.1|_27908	3.6e+2	0.61 ± 0.06		721	571	1	149	yes				blast	agcagcagcuggagcagugggg	ccucuccuccggcggccgcgg	ccucuccuccggcggccgcggcggcagcagcagcagcagcagcuggagcagugggg	gi|307603381|gb|CM000995.1|:153270161..15
gi|307603370|gb|CM001006.1|_10046	3.3e+2	0.61 ± 0.06		664	478	0	186	yes				blast	uagguagaccaggcugaucu	uguagcccuggcuguccugga	uguagcccuggcuguccuggaacuugcuagguagaccaggcugaucu	gi|307603370|gb|CM001006.1|:48980298..489
gi|307603374|gb|CM001002.1|_46090	3.3e+2	0.61 ± 0.06		659	630	0	29	yes				blast	ugugucacugggguuaugcuuuga	aagccgggccuagugacucucu	ugugucacugggguuaugcuuugagguuucuaacucucaagccgggccuagugacucucu	gi|307603374|gb|CM001002.1|:74814549..748
gi|307603377|gb|CM000999.1|_38851	3.3e+2	0.61 ± 0.06		651	488	0	163	yes				blast	uggaaggaugccucuugcagaug	ucugcagaggcuuccuuggc	uggaaggaugccucuugcagauguagcagaggagguaucugcagaggcuuccuuggc	gi|307603377|gb|CM000999.1|:99134956..991
gi|307603380|gb|CM000996.1|_28790	2.6e+2	0.61 ± 0.06		520	462	0	58	yes				blast	aagcaucccucucccggucuc	gagcggguggcuggugugagc	aagcaucccucucccggucucgacgcaucccgggagcggguggcuggugugagc	gi|307603380|gb|CM000996.1|:88019907..880
gi|307603364|gb|CM001012.1|_20539	2.6e+2	0.61 ± 0.06		509	471	0	38	yes				blast	accuccagguccuauauauagcu	ccuauauauaggacuuugggcgg	ccuauauauaggacuuugggcggcuccuggccucagcuaccuccagguccuauauauagcu	gi|307603364|gb|CM001012.1|:59544738..595
gi|307603366|gb|CM001010.1|_17021	2.4e+2	0.61 ± 0.06		487	403	6	78	yes				blast	uuccuuggcugugucugagcacc	gugcugugaugaugccuu	gugcugugaugaugccuuaaaauugugguuucgacucacugagaguaacgugaggaccuacaguuccuuggcugugucugagcacc	gi|307603366|gb|CM001010.1|:71980620..719
gi|307603376|gb|CM001000.1|_40427	2.4e+2	0.61 ± 0.06		489	291	0	198	no				blast	uggugugcuacaguucucggaga	gccgaggcuagagucacauccuga	gccgaggcuagagucacauccugacauggugcuuguccuggugugcuacaguucucggaga	gi|307603376|gb|CM001000.1|:116664793..11
gi|307603375|gb|CM001001.1|_44120	2.2e+2	0.61 ± 0.06		448	438	0	10	yes				blast	uucagggauaaauggagucacaga	cugugacuccugagcucugu	cugugacuccugagcucuguuccccaugauagguucagggauaaauggagucacaga	gi|307603375|gb|CM001001.1|:22566566..225
gi|307603366|gb|CM001010.1|_16913	2.2e+2	0.61 ± 0.06		448	440	0	8	no				blast	ucuuguccccgcugcccacagc	ugcgggcgggcgcgggcc	ugcgggcgggcgcgggccgagguaccgugucucagccgcugucuuguccccgcugcccacagc	gi|307603366|gb|CM001010.1|:56752946..567
gi|307603382|gb|CM000994.1|_22963	2.2e+2	0.61 ± 0.06		437	386	0	51	yes				blast	uaccaccacacccugcuuugacu	uaaagccgggcgguggugg cgc	uaccaccacacccugcuuugacuguuacuauuaagauuuuaaguaaagccgggcgguggugg cgc	gi|307603382|gb|CM000994.1|:16641565..166
gi|307603365|gb|CM001011.1|_18583	2.2e+2	0.61 ± 0.06		435	336	0	99	yes				blast	uguguauuccugguuguucug	aggagggccuagaauucauaga	uguguauuccugguuguucuggaauuagcuguguaaaccaggagggccuagaauucauaga	gi|307603365|gb|CM001011.1|:37913958..379
gi|307603374|gb|CM001002.1|_46313	2.0e+2	0.61 ± 0.06		393	387	0	6	yes				blast	ugucccugucccgguacccagc	agggugg agggaaacggauagc	agggugg agggaaacggauagcagggaacuggcaggcugucccugucccgguacccagc	gi|307603374|gb|CM001002.1|:106051238..10
gi|307603371|gb|CM001005.1|_7851	1.9e+2	0.61 ± 0.06		398	367	0	31	no				blast	gaacugagugcuucugacag	cagagguacuagugucca	gaacugagugcuucugacagcaaaaggcagagguacuagugucca	gi|307603371|gb|CM001005.1|:55993599..559
gi|307603381|gb|CM000995.1|_24740	1.8e+2	0.61 ± 0.06		353	257	0	96	yes				blast	aggggaccauucuugugaagga	uucaaaaggaugggcccacagc	aggggaccauucuugugaaggaaaucagagugcaccuucaaaaggaugggcccacagc	gi|307603381|gb|CM000995.1|:22506610..225
gi|307603370|gb|CM001006.1|_8926	1.6e+2	0.61 ± 0.06		335	317	0	18	no				blast	cugcccugugguugcuggagg	uccaguggauacaaugggcuaga	cugcccugugguugcuggaggcuguugugcagguacgguucuccaguggauacaaugggcuaga	gi|307603370|gb|CM001006.1|:49779667..497
gi|307603382|gb|CM000994.1|_22291	1.6e+2	0.61 ± 0.06		330	319	0	11	no				blast	cagacgcgcggcgcuccgggagc	cggcgggggcgccgggcg	cggcgggggcgccgggcgccgcgggccugcuccgugcuccgcagacgcgcggcgcuccgggagc	gi|307603382|gb|CM000994.1|:134776972..13
gi|307603376|gb|CM001000.1|_39993	1.6e+2	0.61 ± 0.06		322	251	0	71	yes				blast	cacugacagcagcaucuccauga	uggagacuucccugucagagc	uggagacuucccugucagagcccgaaucggacguggcacugacagcagcaucuccauga	gi|307603376|gb|CM001000.1|:56216904..562
gi|307603379|gb|CM000997.1|_33626	1.6e+2	0.61 ± 0.06		326	280	13	33	no				blast	cugggcucgcugaagcguu	cgcggccgcgggcgccagagc	cgcggccgcgggcgccagagcgacggcggcggcugcggcucugggcucgcugaagcguu	gi|307603379|gb|CM000997.1|:142802533..14
gi|307603381|gb|CM000995.1|_27551	1.5e+2	0.61 ± 0.06		304	288	0	16	no				blast	uuuugagacaggcugaccugg	caggcuggccucgaacucacag	uuuugagacaggcugaccuggaacucccucuguaagccaggcuggccucgaacucacag	gi|307603381|gb|CM000995.1|:119583358..11
gi|307603373|gb|CM001003.1|_1860	1.4e+2	0.61 ± 0.06		297	266	0	31	no				blast	agcggcggcggcggcggc	cggggcgcaauggagcggaagag	agcggcggcggcggcggcaucggccacggcgggcggaaaagccggggcgcaauggagcggaagag	gi|307603373|gb|CM001003.1|:79861962..798
gi|307603378|gb|CM000998.1|_34190	1.4e+2	0.61 ± 0.06		293	273	0	20	no				blast	agggcugagaagggcgggccggu	gaggcuugcuugaacggg	agggcugagaagggcgggccggugccggacgagcggaagugggucuucgugaggcgguggaggcuugcuugaacggg	gi|307603378|gb|CM000998.1|:34679032..346
gi|307603377|gb|CM000999.1|_39312	1.4e+2	0.61 ± 0.06		296	288	0	8	no				blast	uauuucauugggccacucagcug	acugguagcucuaaaug	acugguagcucuaaaugaaaugagcugaauauuucauugggccacucagcug	gi|307603377|gb|CM000999.1|:144560116..14
gi|307603378|gb|CM000998.1|_34401	1.3e+2	0.61 ± 0.06		271	270	0	1	no				blast	agcccggggcugcgcgagacac	ucucagcggcccgcggggcgg	ucucagcggcccgcggggcgggcggcaguguccagcccggggcugcgcgagacac	gi|307603378|gb|CM000998.1|:74489270..744
gi|307603380|gb|CM000996.1|_30042	1.1e+2	0.61 ± 0.06		222	213	0	9	yes				blast	cuuggaugguacuggggcaggc	ucccccagccccacccaguga	ucccccagccccacccagugacucagagugucaggaacuuggaugguacuggggcaggc	gi|307603380|gb|CM000996.1|:83825935..838
gi|307603381|gb|CM000995.1|_28089	1.1e+2	0.61 ± 0.06		219	209	0	10	yes				blast	acaaguaccaaggggaagagaau	uucuccucucacuggugcgga	acaaguaccaaggggaagagaaugacaucauucuccucucacuggugcgga	gi|307603381|gb|CM000995.1|:166864371..16
gi|307603382|gb|CM000994.1|_22582	1.1e+2	0.61 ± 0.06		226	136	81	9	no				blast	gcccgcguacugugccucugc	cguggcuggucguggggc	gcccgcguacugugccucugcagccgcauuacuuugagccugccugcguggcuggucguggggc	gi|307603382|gb|CM000994.1|:167564834..16
gi|307603370|gb|CM001006.1|_9283	1.1e+2	0.61 ± 0.06		224	165	0	59	no				blast	acagggcgccgagagggg	ccaccggcgcuggaucug	ccaccggcgcuggaucugggcgcgcguuaggggggaagcugcaggcacagggcgccgagagggg	gi|307603370|gb|CM001006.1|:91510711..915
gi|307603372|gb|CM001004.1|_2636	1.1e+2	0.61 ± 0.06		222	209	0	13	no				blast	ucugagggugcaacuggucugcc	cauacuagaggcagcccagagu	ucugagggugcaacuggucugccugucugacuuuaccugcauacuagaggcagcccagagu	gi|307603372|gb|CM001004.1|:24016674..240
gi|307603374|gb|CM001002.1|_45948	1.0e+2	0.61 ± 0.06		214	204	0	10	no				blast	cgcgagggucuggcuccg	uaagccgcccucgcugcucagc	cgcgagggucuggcuccgcccuugaugccagcgcuaagccgcccucgcugcucagc	gi|307603374|gb|CM001002.1|:64188435..641
gi|307603381|gb|CM000995.1|_25619	1.0e+2	0.61 ± 0.06		205	199	0	6	no				blast	gcgcacgcucugauccucucuccc	gggggagggggcgggca	gggggagggggcgggcagcgcggccaagcccacccagcacacacaucgguggcugcggaggaccccgcgcacgcucugauccucucuccc	gi|307603381|gb|CM000995.1|:118199205..11
gi|307603369|gb|CM001007.1|_10703	9.6e+1	0.61 ± 0.06		194	127	38	29	no				blast	caccgugucccugcgcuccg	gggggcgggggggggggg	gggggcgggggggggggggggggcgaacgcgucggaagucagagagcaccgugucccugcgcuccg	gi|307603369|gb|CM001007.1|:4518418..4518
gi|307603376|gb|CM001000.1|_41405	9.5e+1	0.61 ± 0.06		183	169	0	14	yes				blast	ucugucccacugcugugaua	aucacagcagugggacaguaau	ucugucccacugcugugauaaaaccuucuguuuuaucacagcagugggacaguaau	gi|307603376|gb|CM001000.1|:35534757..355
gi|307603366|gb|CM001010.1|_16507	8.9e+1	0.61 ± 0.06		178	150	0	28	no				blast	cgcugcgugccgagaagg	gagcucgaggaccaggcgcgcgc	gagcucgaggaccaggcgcgcgcucugcccgagcugcaggagcaggugcgcgcgcugcgugccgagaagg	gi|307603366|gb|CM001010.1|:33954695..339
gi|307603378|gb|CM000998.1|_36624	8.5e+1	0.61 ± 0.06		167	148	0	19	yes				blast	cuguccuccaguguuagugcuc	ugacuaauccggagucacagcu	ugacuaauccggagucacagcuguguccuugcucuagcuguccuccaguguuagugcuc	gi|307603378|gb|CM000998.1|:139787979..13
gi|307603373|gb|CM001003.1|_831	8.1e+1	0.61 ± 0.06		170	139	0	31	no				blast	uuugauggcccugaaguaaga	cuuauuuaaggggaacguaug	cuuauuuaaggggaacguaugggugauaaugcauuugauggcccugaaguaaga	gi|307603373|gb|CM001003.1|:84488854..844
gi|307603375|gb|CM001001.1|_43932	8.0e+1	0.61 ± 0.06		154	125	0	29	yes				blast	agcucuguggaaguuggggugu	cucccagacuuccucaggcuuu	agcucuguggaaguuggggugugucucuccuuacccacucugcucccagacuuccucaggcuuu	gi|307603375|gb|CM001001.1|:127068796..12
gi|307603381|gb|CM000995.1|_27101	8.0e+1	0.61 ± 0.06		154	98	0	56	yes				blast	gaugccguggcacagaaugggaa	uccucugugcccaugguaacca	gaugccguggcacagaaugggaagccaggagagucuuccucugugcccaugguaacca	gi|307603381|gb|CM000995.1|:68052631..680
gi|307603382|gb|CM000994.1|_22770	7.4e+1	0.61 ± 0.06		149	135	0	14	no				blast	agggugaagcucgcgcgacg	uccgcugcugucacccggcucuc	agggugaagcucgcgcgacgccaggcaaaagcguccgcugcugucacccggcucuc	gi|307603382|gb|CM000994.1|:183772740..18
gi|307603381|gb|CM000995.1|_27412	6.9e+1	0.61 ± 0.06		135	117	0	18	yes				blast	gaauuguuccucuuaagucga	guacuugagagaggcaaagcuga	guacuugagagaggcaaagcugaaagguaucaaccagaauuguuccucuuaagucga	gi|307603381|gb|CM000995.1|:104522827..10
gi|307603376|gb|CM001000.1|_39410	6.6e+1	0.61 ± 0.06		137	95	31	11	no				blast	cauccugggccugaagauuga	aagcugggccuggccaaauc	cauccugggccugaagauugaggauuucuuggagaggcggcugcagacccaggucuuuaagcugggccuggccaaauc	gi|307603376|gb|CM001000.1|:3657401..3657
gi|307603374|gb|CM001002.1|_47406	6.4e+1	0.61 ± 0.06		132	88	0	44	no				blast	aguggcugugccucguccgcac	agcggcggggcccugacccucc	agcggcggggcccugacccucccaacggugucgcagaccggaguggcugugccucguccgcac	gi|307603374|gb|CM001002.1|:70269264..702
gi|307603380|gb|CM000996.1|_29957	6.3e+1	0.61 ± 0.06		122	64	0	58	yes				blast	auccgcgaggcuccugagagac	ucucaggagccucaggaaccaaaa	auccgcgaggcuccugagagacaacuuggucucucaggagccucaggaaccaaaa	gi|307603380|gb|CM000996.1|:67177815..671
gi|307603381|gb|CM000995.1|_26464	6.3e+1	0.61 ± 0.06		122	103	0	19	yes				blast	gugagugccugcugcccugagu	cucaaggucuagcagguagcaca	gugagugccugcugcccugaguugagagggaguccuuaacacagcucaaggucuagcagguagcaca	gi|307603381|gb|CM000995.1|:181114394..18
gi|307603373|gb|CM001003.1|_1388	6.3e+1	0.61 ± 0.06		133	132	0	1	no				blast	cagaggcuguuucugagc	gccagugacuuuggccuu	gccagugacuuuggccuuaggcuucugaaugaccagugcaugcagaggcuguuucugagc	gi|307603373|gb|CM001003.1|:28567927..285
gi|307603371|gb|CM001005.1|_6930	6.2e+1	0.61 ± 0.06		128	115	0	13	no				blast	cuuccuccuugacuggucauc	agcacccggagaaggagggcggac	cuuccuccuugacugggucaucuguucauuaccaaaagaagcacccggagaaggagggcggac	gi|307603371|gb|CM001005.1|:87443855..874
gi|307603366|gb|CM001010.1|_16566	5.9e+1	0.61 ± 0.06		121	105	0	16	no				blast	agggacuuaggucgggug	acgcugcgugagaaccugga	agggacuuaggucgggugugcuugggaugcgcugagcugugcacgcugcgugagaaccugga	gi|307603366|gb|CM001010.1|:35258659..352
gi|307603371|gb|CM001005.1|_6622	5.9e+1	0.61 ± 0.06		116	76	26	14	yes				blast	cacccuuuccugugcccuuuuaa	uaagaggcuaacacagaagg	cacccuuuccugugcccuuuuaagguugacacuguacuuuaagaggcuaacacagaagg	gi|307603371|gb|CM001005.1|:60235047..602
gi|307603382|gb|CM000994.1|_23610	5.4e+1	0.61 ± 0.06		104	85	0	19	yes				blast	uaacccuucugucucucucuaga	uagaggacaggcaggauggguc	uagaggacaggcaggauggucuccucucuaggagaguaacccuucugucucucucuaga	gi|307603382|gb|CM000994.1|:94957393..949
gi|307603379|gb|CM000997.1|_31069	5.4e+1	0.61 ± 0.06		103	77	0	26	yes				blast	ucccuucagcuucaggccucau	acuggccucaggcuguggugcc	acuggccucaggcuguggugcccaguuaccugcugguccc uucagcuucaggccucau	gi|307603379|gb|CM000997.1|:41263490..412
gi|307603378|gb|CM000998.1|_35503	5.3e+1	0.61 ± 0.06		109	107	0	2	no				blast	auggcggacucgguggcccg	aggccgcgggggagccc	auggcggacucgguggcccgcugaugaggaggccgcgggggagccc	gi|307603378|gb|CM000998.1|:23950941..239
gi|307603376|gb|CM001000.1|_41844	5.2e+1	0.61 ± 0.06		100	89	0	11	yes				blast	ugugcauggugcacucucucucag	gauggagggucaugcugcagcaug	gauggagggucaugcugcagcaugaaucaaauauaacaugugcauggugcacucucucucag	gi|307603376|gb|CM001000.1|:69314438..693
gi|307603372|gb|CM001004.1|_5333	5.0e+1	0.61 ± 0.06		104	58	0	46	no				blast	gaugacccugggcaguacagu	ugugcuuguccugguucaccu	gaugacccugggcaguacaguggugu ccagagaggcugugcuuguccugguucaccu	gi|307603372|gb|CM001004.1|:81754669..817
gi|307603364|gb|CM001012.1|_20748	4.9e+1	0.61 ± 0.06		93	62	0	31	yes				blast	aucugucgcugcagggcgugg	gcggacgcagcgacagug	aucugucgcugcagggcguggacgacgcggacgcagcgacagug	gi|307603364|gb|CM001012.1|:6117995..6118

<table>
<tr><td>gi|307603381|gb|CM000995.1|_26462</td><td>4.7e+1</td><td>0.61 ± 0.06</td><td></td><td>92</td><td>75</td><td>0</td><td>17</td><td>yes</td><td></td><td></td><td></td><td>blast</td><td>acuugccuguguccccauagaa</td><td>cucuggggagcagugcaagc</td><td>acuugccuguguccccauagaagggcuggaaucccuuucucuggggagcagugcaagc</td><td>gi|307603381|gb|CM000995.1|:181086426..181</td></tr>
<tr><td>gi|307603370|gb|CM001006.1|_9559</td><td>4.6e+1</td><td>0.61 ± 0.06</td><td></td><td>88</td><td>81</td><td>0</td><td>7</td><td>yes</td><td></td><td></td><td></td><td>blast</td><td>cacgccgcggcucacacacacagu</td><td>ugugucguguggcacgccgcgg</td><td>ugugucguguggcacgccgcggcucacacacacaguuagccaucacaacugugucguguggcacgccgcggcucacacacacagu</td><td>gi|307603370|gb|CM001006.1|:12295820..1229</td></tr>
<tr><td>gi|307603370|gb|CM001006.1|_9934</td><td>4.6e+1</td><td>0.61 ± 0.06</td><td></td><td>90</td><td>75</td><td>0</td><td>15</td><td>yes</td><td></td><td></td><td></td><td>blast</td><td>uaugcugggguagguacugucugg</td><td>guccaguacuccucagugaaac</td><td>guccaguacuccucagugaaacauacaugaugacauuguaugcugggguagguacugucugg</td><td>gi|307603370|gb|CM001006.1|:36174300..3617</td></tr>
<tr><td>gi|307603379|gb|CM000997.1|_32599</td><td>4.5e+1</td><td>0.61 ± 0.06</td><td></td><td>92</td><td>69</td><td>0</td><td>23</td><td>no</td><td></td><td></td><td></td><td>blast</td><td>ucgcgggcugccgagcuccagguc</td><td>ccgggucccggcagcugcggc</td><td>ucgcgggcugccgagcuccagguccugcgcucuccacgcguccgggucccggcagcugcggc</td><td>gi|307603379|gb|CM000997.1|:45421417..4542</td></tr>
<tr><td>gi|307603366|gb|CM001010.1|_16568</td><td>4.4e+1</td><td>0.61 ± 0.06</td><td></td><td>83</td><td>60</td><td>0</td><td>23</td><td>yes</td><td></td><td></td><td></td><td>blast</td><td>ugggcagcagcaaccacagcagg</td><td>uggacggggcugcugcccgggc</td><td>ugggcagcagcaaccacagcaggagaaagcagagccuguccuggacggggcugcugcccgggc</td><td>gi|307603366|gb|CM001010.1|:35260061..3526</td></tr>
<tr><td>gi|307603370|gb|CM001006.1|_8520</td><td>4.4e+1</td><td>0.61 ± 0.06</td><td></td><td>83</td><td>60</td><td>0</td><td>23</td><td>yes</td><td></td><td></td><td></td><td>blast</td><td>ugggcagcagcaaccacagcagg</td><td>uggacggggcugcugcccgggc</td><td>ugggcagcagcaaccacagcaggagaaagcagagccuguccuggacggggcugcugcccgggc</td><td>gi|307603370|gb|CM001006.1|:18041803..1804</td></tr>
<tr><td>gi|307603372|gb|CM001004.1|_5586</td><td>4.3e+1</td><td>0.61 ± 0.06</td><td></td><td>88</td><td>57</td><td>0</td><td>31</td><td>no</td><td></td><td></td><td></td><td>blast</td><td>agccccacgccucccccaugcag</td><td>uguggggcgugugugggcugcugg</td><td>uguggggcgugugugggcugcuggugcgcgcccccagccccacgccucccccaugcag</td><td>gi|307603372|gb|CM001004.1|:97435227..9743</td></tr>
<tr><td>gi|307603374|gb|CM001002.1|_48042</td><td>4.2e+1</td><td>0.61 ± 0.06</td><td></td><td>80</td><td>50</td><td>0</td><td>30</td><td>yes</td><td></td><td></td><td></td><td>blast</td><td>ucagaugugugcagaguccaaa</td><td>gagacccugcacgcauuggugg</td><td>gagacccugcacgcauugguggcgugucuguguccuguucagaugugugcagaguccaaa</td><td>gi|307603374|gb|CM001002.1|:122998724..122</td></tr>
<tr><td>gi|307603378|gb|CM000998.1|_34505</td><td>4.1e+1</td><td>0.61 ± 0.06</td><td></td><td>86</td><td>52</td><td>18</td><td>16</td><td>no</td><td></td><td></td><td></td><td>blast</td><td>ggcaaguagcgaagccggagaagu</td><td>uuccaguccggugagccug</td><td>uuccaguccggugagccugccgaguccuggccuuccacuuccgccccucagccggccgcggcaaguagcgaagccggagaagu</td><td>gi|307603378|gb|CM000998.1|:89149935..8915</td></tr>
<tr><td>gi|307603375|gb|CM001001.1|_44545</td><td>3.8e+1</td><td>0.61 ± 0.06</td><td></td><td>80</td><td>59</td><td>0</td><td>21</td><td>no</td><td></td><td></td><td></td><td>blast</td><td>ccggaccucaagcagccucacg</td><td>uggagcggccugagguccagcg</td><td>ccggaccucaagcagccucacgcggcuccaggguggagcggccugagguccagcg</td><td>gi|307603375|gb|CM001001.1|:74432570..7443</td></tr>
<tr><td>gi|307603372|gb|CM001004.1|_6106</td><td>3.7e+1</td><td>0.61 ± 0.06</td><td></td><td>80</td><td>65</td><td>0</td><td>15</td><td>no</td><td></td><td></td><td></td><td>blast</td><td>guugugcucugucugccucaca</td><td>ccuggcacucagggcaucccccu</td><td>ccuggcacucagggcaucccccuuucauagcugaccucaggguugugcucugucugccucaca</td><td>gi|307603372|gb|CM001004.1|:119016531..119</td></tr>
<tr><td>gi|307603370|gb|CM001006.1|_10399</td><td>3.7e+1</td><td>0.61 ± 0.06</td><td></td><td>80</td><td>62</td><td>0</td><td>18</td><td>no</td><td></td><td></td><td></td><td>blast</td><td>cccuuucggucuucgcgcucugc</td><td>aggucguguagaaagggg</td><td>cccuuucggucuucgcgcucugccgagcaaggucguguagaaagggg</td><td>gi|307603370|gb|CM001006.1|:86186348..8618</td></tr>
<tr><td>gi|307603363|gb|CM001013.1|_48408</td><td>3.7e+1</td><td>0.61 ± 0.06</td><td></td><td>71</td><td>32</td><td>0</td><td>39</td><td>yes</td><td></td><td></td><td></td><td>blast</td><td>uaagggggaucagggauccaga</td><td>cugagcacccuggcccccagau</td><td>uaagggggaucagggauccagagcccucucugagcacccuggcccccagau</td><td>gi|307603363|gb|CM001013.1|:70923925..7092</td></tr>
<tr><td>gi|307603378|gb|CM000998.1|_34420</td><td>3.6e+1</td><td>0.61 ± 0.06</td><td></td><td>76</td><td>49</td><td>0</td><td>27</td><td>no</td><td></td><td></td><td></td><td>blast</td><td>gggcgggcgcguucacgcggacgg</td><td>cgcucccgggacgccggccucuu</td><td>gggcgggcgcguucacgcggacggucucgcgcgcucccgggacgccggccucuu</td><td>gi|307603378|gb|CM000998.1|:75440712..7544</td></tr>
<tr><td>gi|307603376|gb|CM001000.1|_40651</td><td>3.6e+1</td><td>0.61 ± 0.06</td><td></td><td>80</td><td>31</td><td>34</td><td>15</td><td>no</td><td></td><td></td><td></td><td>blast</td><td>gcaguugaggaaugguacag</td><td>agaccaagacugaaguggacgug</td><td>agaccaagacugaaguggacgugccuaugugggcggggacuccaggaccgcgaguaucgugcaguugaggaaugguacag</td><td>gi|307603376|gb|CM001000.1|:134537316..134</td></tr>
<tr><td>gi|307603368|gb|CM001008.1|_13118</td><td>3.4e+1</td><td>0.61 ± 0.06</td><td></td><td>71</td><td>56</td><td>0</td><td>15</td><td>no</td><td></td><td></td><td></td><td>blast</td><td>gcccgggcagcgccagacgagug</td><td>uccgucccggugcccuccggcc</td><td>gcccgggcagcgccagacgaguggcccgcuccgucccggugcccuccggcc</td><td>gi|307603368|gb|CM001008.1|:85501940..8550</td></tr>
<tr><td>gi|307603382|gb|CM000994.1|_23549</td><td>3.3e+1</td><td>0.61 ± 0.06</td><td></td><td>64</td><td>57</td><td>0</td><td>7</td><td>yes</td><td></td><td></td><td></td><td>blast</td><td>cuuucgcuuccuucgcuuuggu</td><td>caaagcguuuuaagcggaaaug</td><td>cuuucgcuuccuucgcuuugguuucccaucaaagcguuuuaagcggaaaug</td><td>gi|307603382|gb|CM000994.1|:90171718..9017</td></tr>
<tr><td>gi|307603381|gb|CM000995.1|_27875</td><td>3.2e+1</td><td>0.61 ± 0.06</td><td></td><td>69</td><td>58</td><td>0</td><td>11</td><td>no</td><td></td><td></td><td></td><td>blast</td><td>ugucuguacaguucagguggga</td><td>ucaaccccaacuguccagucgc</td><td>ugucuguacaguucaggugggauguggaugaugguuucucaaccccaacuguccagucgc</td><td>gi|307603381|gb|CM000995.1|:151797021..151</td></tr>
<tr><td>gi|307603363|gb|CM001013.1|_49805</td><td>3.0e+1</td><td>0.61 ± 0.06</td><td></td><td>59</td><td>37</td><td>0</td><td>22</td><td>yes</td><td></td><td></td><td></td><td>blast</td><td>uggucuggcuagcagagaggg</td><td>cuugcauguuggugaaggaaaa</td><td>uggucuggcuagcagagagggaaaagaacuugcauguuggugaaggaaaa</td><td>gi|307603363|gb|CM001013.1|:163645191..163</td></tr>
<tr><td>gi|307603375|gb|CM001001.1|_44144</td><td>3.0e+1</td><td>0.61 ± 0.06</td><td></td><td>57</td><td>30</td><td>0</td><td>27</td><td>yes</td><td></td><td></td><td></td><td>blast</td><td>gugcucauaggccugucugca</td><td>ucugggcagguuccucugagc</td><td>ucugggcagguuccucugagcagcagugguuguucuaccugugcucauaggccugucugca</td><td>gi|307603375|gb|CM001001.1|:25965280..2596</td></tr>
<tr><td>gi|307603382|gb|CM000994.1|_21577</td><td>2.9e+1</td><td>0.61 ± 0.06</td><td></td><td>63</td><td>48</td><td>0</td><td>15</td><td>no</td><td></td><td></td><td></td><td>blast</td><td>cgcuuuacggccgcgggag</td><td>uccgcucgucugacuuaggca</td><td>uccgcucgucugacuuaggcacuguuuuuguuugcgacgcugucgcggaagggugcgcuuuacggccgcgggag</td><td>gi|307603382|gb|CM000994.1|:39592548..3959</td></tr>
<tr><td>gi|307603381|gb|CM000995.1|_25070</td><td>2.9e+1</td><td>0.61 ± 0.06</td><td></td><td>62</td><td>36</td><td>0</td><td>26</td><td>no</td><td></td><td></td><td></td><td>blast</td><td>uggccaggacacugcucucacgg</td><td>uggagaacggugcugggcagg</td><td>uggccaggacacugcucucacgguugauauuagagccuggagaacggugcugggcagg</td><td>gi|307603381|gb|CM000995.1|:35585740..3558</td></tr>
<tr><td>gi|307603382|gb|CM000994.1|_24094</td><td>2.8e+1</td><td>0.61 ± 0.06</td><td></td><td>61</td><td>55</td><td>0</td><td>6</td><td>no</td><td></td><td></td><td></td><td>blast</td><td>cucgcagccgcgauccggacag</td><td>uggggccgggagcggggagcgg</td><td>uggggccgggagcggggagcgguggcguggagguguccucgcgcucgcagccgcgauccggacag</td><td>gi|307603382|gb|CM000994.1|:167564594..167</td></tr>
<tr><td>gi|307603375|gb|CM001001.1|_44605</td><td>2.7e+1</td><td>0.61 ± 0.06</td><td></td><td>60</td><td>49</td><td>0</td><td>11</td><td>no</td><td></td><td></td><td></td><td>blast</td><td>aaggacuggaagggacug</td><td>agucgcugcugucagguucuc</td><td>aaggacuggaagggacugagcgcuuuuuccuuacauaagaaaggcgucgcagucgcugcugucagguucuc</td><td>gi|307603375|gb|CM001001.1|:81818575..8181</td></tr>
<tr><td>gi|307603380|gb|CM000996.1|_29928</td><td>2.7e+1</td><td>0.61 ± 0.06</td><td></td><td>58</td><td>35</td><td>0</td><td>23</td><td>no</td><td></td><td></td><td></td><td>blast</td><td>gccgucacugccaccacaggaa</td><td>cucuugggcaucguggcagcg</td><td>cucuugggcaucguggcagcgcgggguggggucacagaggagccgcacugccaagccgucacugccaccacaggaa</td><td>gi|307603380|gb|CM000996.1|:63709286..6370</td></tr>
<tr><td>gi|307603364|gb|CM001012.1|_20081</td><td>2.6e+1</td><td>0.61 ± 0.06</td><td></td><td>59</td><td>34</td><td>0</td><td>25</td><td>no</td><td></td><td></td><td></td><td>blast</td><td>ggacucugcauucuucag</td><td>gaguuacacggggcacagagga</td><td>gaguuacacggggcacagaggacaucauggacgaugcuggcgaggacucugcauucuucag</td><td>gi|307603364|gb|CM001012.1|:21872574..2187</td></tr>
<tr><td>gi|307603382|gb|CM000994.1|_23824</td><td>2.6e+1</td><td>0.61 ± 0.06</td><td></td><td>58</td><td>51</td><td>0</td><td>7</td><td>no</td><td></td><td></td><td></td><td>blast</td><td>ccugagcccucuucuccaucucc</td><td>ugguggaggaacuggggcgcugc</td><td>ugguggaggaacuggggcgcugcaugcuugaagacaguugccugagcccucuucuccaucucc</td><td>gi|307603382|gb|CM000994.1|:134425286..134</td></tr>
<tr><td>gi|307603382|gb|CM000994.1|_21896</td><td>2.5e+1</td><td>0.61 ± 0.06</td><td></td><td>54</td><td>31</td><td>0</td><td>23</td><td>no</td><td></td><td></td><td></td><td>blast</td><td>ggagucggugccuuuuaagcuggc</td><td>cgcggaagucuggcucggcug</td><td>ggagucggugccuuuuaagcuggcuagccguguucuucgcugguccgcggaagucuggcucggcug</td><td>gi|307603382|gb|CM000994.1|:74863628..7486</td></tr>
<tr><td>gi|307603370|gb|CM001006.1|_8436</td><td>2.4e+1</td><td>0.61 ± 0.06</td><td></td><td>54</td><td>28</td><td>17</td><td>9</td><td>no</td><td></td><td></td><td></td><td>blast</td><td>agaaggcgugcacaggaaauga</td><td>cucuucuaguguauugugg</td><td>agaaggcgugcacaggaaaugaggcuccucagucggaaccucuucuaguguauugugg</td><td>gi|307603370|gb|CM001006.1|:3610545..3610</td></tr>
<tr><td>gi|307603375|gb|CM001001.1|_44461</td><td>2.3e+1</td><td>0.61 ± 0.06</td><td></td><td>43</td><td>33</td><td>0</td><td>10</td><td>yes</td><td></td><td></td><td></td><td>blast</td><td>ugguucuuccuggggcuuccg</td><td>ugaagcuagggaagugccggu</td><td>ugguucuuccuggggcuuccguguagcucuauucugaagcuagggaagugccggu</td><td>gi|307603375|gb|CM001001.1|:71849971..7185</td></tr>
<tr><td>gi|307603379|gb|CM000997.1|_31593</td><td>2.3e+1</td><td>0.61 ± 0.06</td><td></td><td>43</td><td>42</td><td>0</td><td>1</td><td>yes</td><td></td><td></td><td></td><td>blast</td><td>aguggucagggccaagccagag</td><td>cagagccuggucaaggucacuccc</td><td>cagagccuggucaaggucacucccacccuggugaaggaguggucagggccaagccagag</td><td>gi|307603379|gb|CM000997.1|:109657964..109</td></tr>
<tr><td>gi|307603369|gb|CM001007.1|_11728</td><td>2.3e+1</td><td>0.61 ± 0.06</td><td></td><td>49</td><td>46</td><td>0</td><td>3</td><td>no</td><td></td><td></td><td></td><td>blast</td><td>cggcggugugggagcucg</td><td>aggggcggaccgcggcug</td><td>aggggcggaccgcggcugugcaggcggcggugugggagcucg</td><td>gi|307603369|gb|CM001007.1|:25065098..2506</td></tr>
<tr><td>gi|307603369|gb|CM001007.1|_12028</td><td>2.3e+1</td><td>0.61 ± 0.06</td><td></td><td>50</td><td>31</td><td>0</td><td>19</td><td>no</td><td></td><td></td><td></td><td>blast</td><td>cgggcugcgcuacgaagg</td><td>auggagugagccagagau</td><td>auggagugagccagagauccaacgggcugcgcuacgaagg</td><td>gi|307603369|gb|CM001007.1|:55732474..5573</td></tr>
<tr><td>gi|307603371|gb|CM001005.1|_8283</td><td>2.3e+1</td><td>0.61 ± 0.06</td><td></td><td>54</td><td>53</td><td>0</td><td>1</td><td>no</td><td></td><td></td><td></td><td>blast</td><td>uugcacuuuggaucucugcgcaga</td><td>agcgcuuagaauuccugg</td><td>uugcacuuuggaucucugcgcagauuguuaccagcgcuuagaauuccugg</td><td>gi|307603371|gb|CM001005.1|:105932929..105</td></tr>
<tr><td>gi|307603371|gb|CM001005.1|_7824</td><td>2.1e+1</td><td>0.61 ± 0.06</td><td></td><td>46</td><td>38</td><td>0</td><td>8</td><td>no</td><td></td><td></td><td></td><td>blast</td><td>gccgcgggcggacaaugcg</td><td>cagagcccgcucgcugccg</td><td>cagagcccgcucgcugccgcagccgccgcgggcggacaaugcg</td><td>gi|307603371|gb|CM001005.1|:54354233..5435</td></tr>
<tr><td>gi|307603364|gb|CM001012.1|_20342</td><td>2.1e+1</td><td>0.61 ± 0.06</td><td></td><td>46</td><td>34</td><td>0</td><td>12</td><td>no</td><td></td><td></td><td></td><td>blast</td><td>gacuuuuguaaacagccugugcgu</td><td>cgccaccuggcuggugaacagug</td><td>gacuuuuguaaacagccugugcguuagcaccuucuugcgauacgccguggugcucaacgccaccuggcuggugaacagug</td><td>gi|307603364|gb|CM001012.1|:44375773..4437</td></tr>
<tr><td>gi|307603375|gb|CM001001.1|_44641</td><td>2.0e+1</td><td>0.61 ± 0.06</td><td></td><td>47</td><td>33</td><td>0</td><td>14</td><td>no</td><td></td><td></td><td></td><td>blast</td><td>agcccaacggccgccggcucgagg</td><td>gucccgcugcugugcgcc</td><td>agcccaacggccgccggcucgaggugccuaaccgucccgcugcugugcgcc</td><td>gi|307603375|gb|CM001001.1|:86239327..8623</td></tr>
<tr><td>gi|307603369|gb|CM001007.1|_12450</td><td>2.0e+1</td><td>0.61 ± 0.06</td><td></td><td>41</td><td>36</td><td>0</td><td>5</td><td>yes</td><td></td><td></td><td></td><td>blast</td><td>uaugaccaagaaccuaggg</td><td>uuaggguuuucauugcuguaaa</td><td>uuaggguuuucauugcuguaaaaauacaguaugaccaagaaccuaggg</td><td>gi|307603369|gb|CM001007.1|:103665985..103</td></tr>
<tr><td>gi|307603364|gb|CM001012.1|_20479</td><td>1.9e+1</td><td>0.61 ± 0.06</td><td></td><td>43</td><td>34</td><td>0</td><td>9</td><td>no</td><td></td><td></td><td></td><td>blast</td><td>gagcgagcgggagcgagc</td><td>uccgcggccgccgccauuu</td><td>uccgcggccgccgccauuuuguuuggccacagggggagcgagcgggagcgagc</td><td>gi|307603364|gb|CM001012.1|:53674873..5367</td></tr>
<tr><td>gi|307603365|gb|CM001011.1|_18546</td><td>1.9e+1</td><td>0.61 ± 0.06</td><td></td><td>43</td><td>30</td><td>0</td><td>13</td><td>no</td><td></td><td></td><td></td><td>blast</td><td>uacagguggaggugcagggaau</td><td>cucccugugcccccuccucagc</td><td>uacagguggaggugcagggaauggaagggugggaguuccugaagcagaagguugcauccacacucccugugcccccuccucagc</td><td>gi|307603365|gb|CM001011.1|:36165647..3616</td></tr>
<tr><td>gi|307603379|gb|CM000997.1|_32562</td><td>1.8e+1</td><td>0.61 ± 0.06</td><td></td><td>42</td><td>31</td><td>0</td><td>11</td><td>no</td><td></td><td></td><td></td><td>blast</td><td>aggccugggccagagggga</td><td>ccccgggcugaggugggaggg</td><td>ccccgggcugaggugggagggcgcccgccgcggaaggacugggacaugggcaggcagggagcgaagcgaggccugggccagagggga</td><td>gi|307603379|gb|CM000997.1|:43644522..4364</td></tr>
<tr><td>gi|307603376|gb|CM001000.1|_41630</td><td>1.7e+1</td><td>0.61 ± 0.06</td><td></td><td>39</td><td>30</td><td>0</td><td>9</td><td>no</td><td></td><td></td><td></td><td>blast</td><td>gagauggccuugggcugggug</td><td>aucgcagcucuguggggcccucc</td><td>aucgcagcucuguggggcccuccucuagcucgccuucgccugcggcgcaggggccuuugggaaggggagauggccuugggcugggug</td><td>gi|307603376|gb|CM001000.1|:66483929..6648</td></tr>
<tr><td>gi|307603366|gb|CM001010.1|_16911</td><td>3.4</td><td>0.64 ± 0.05</td><td></td><td>37</td><td>28</td><td>0</td><td>9</td><td>yes</td><td></td><td></td><td></td><td>blast</td><td>uaagggggcugauggcagcgcu</td><td>cugccaucagcccccuuacugu</td><td>cugccaucagcccccuuacuguacugaaucucucaguaagggggcugauggcagcgcu</td><td>gi|307603366|gb|CM001010.1|:56655218..5665</td></tr>
<tr><td>gi|307603378|gb|CM000998.1|_34831</td><td>3.4</td><td>0.64 ± 0.05</td><td></td><td>38</td><td>38</td><td>0</td><td>0</td><td>yes</td><td></td><td></td><td></td><td>blast</td><td>ccccgccgcgccgccccgcgcu</td><td>gguggggauggggauggcgggggga</td><td>gguggggauggggauggcgggggaggggccccgccgcgccgccccgcgcu</td><td>gi|307603378|gb|CM000998.1|:116181633..116</td></tr>
<tr><td>gi|307603372|gb|CM001004.1|_2762</td><td>3.2</td><td>0.64 ± 0.05</td><td></td><td>161</td><td>161</td><td>0</td><td>0</td><td>yes</td><td></td><td></td><td></td><td>blast</td><td>cggccuuccccggcucgcucggu</td><td>cgccccgugggccgccuguggccuca</td><td>cggccuuccccggcucgcucggucccggccgccccuugcgggguugcugggccgccccgugggccgccuguggccuca</td><td>gi|307603372|gb|CM001004.1|:35583192..3558</td></tr>
<tr><td>gi|307603365|gb|CM001011.1|_18359</td><td>3.2</td><td>0.64 ± 0.05</td><td></td><td>98</td><td>98</td><td>0</td><td>0</td><td>yes</td><td></td><td></td><td></td><td>blast</td><td>cgccucgggcuccagcgcgg</td><td>gcgcgggcccggggcgcc</td><td>gcgcgggcccggggcgccaacagucguggcggcgccucgggcuccagcgcgg</td><td>gi|307603365|gb|CM001011.1|:15221651..1522</td></tr>
<tr><td>gi|307603372|gb|CM001004.1|_5855</td><td>3.2</td><td>0.64 ± 0.05</td><td></td><td>33</td><td>33</td><td>0</td><td>0</td><td>yes</td><td></td><td></td><td></td><td>blast</td><td>ugggggcgcggcgggucggg</td><td>cgaggacgccgcgggggacgcc</td><td>ugggggcgcggcgggucggggugauggugacgcugaguggcgcgucucggcccgaggacgccgcgggggacgcc</td><td>gi|307603372|gb|CM001004.1|:104330608..104</td></tr>
<tr><td>gi|307603375|gb|CM001001.1|_44698</td><td>3.1</td><td>0.64 ± 0.05</td><td></td><td>117</td><td>117</td><td>0</td><td>0</td><td>yes</td><td></td><td></td><td></td><td>blast</td><td>uggccgguccucgcucucuucc</td><td>agggugcggaggccggguguc</td><td>agggugcggaggccgggugucucggaggugauggccgguccucgcucucuucc</td><td>gi|307603375|gb|CM001001.1|:87447845..8744</td></tr>
<tr><td>gi|307603376|gb|CM001000.1|_40967</td><td>3.1</td><td>0.64 ± 0.05</td><td></td><td>219</td><td>219</td><td>0</td><td>0</td><td>yes</td><td></td><td></td><td></td><td>blast</td><td>cggcagcgggcccgggggcggcg</td><td>caguggggggcucgggcgggc</td><td>cggcagcgggcccgggggcggcggcggcugcugcggccgcccgugagcggcggcaggagcagcugcggcaguggggggcucgggcgggc</td><td>gi|307603376|gb|CM001000.1|:4453092..44531</td></tr>
<tr><td>gi|307603365|gb|CM001011.1|_18277</td><td>3.1</td><td>0.64 ± 0.05</td><td></td><td>250</td><td>226</td><td>24</td><td>0</td><td>yes</td><td></td><td></td><td></td><td>blast</td><td>gggaacgcgagcugacagcc</td><td>ccggccggggaggcggccucg</td><td>ccggccggggaggcggccucggcgcacaguaauggcagcgcugugagccggggaacgcgagcugacagcc</td><td>gi|307603365|gb|CM001011.1|:3383227..33832</td></tr>
<tr><td>gi|307603376|gb|CM001000.1|_42681</td><td>3.1</td><td>0.64 ± 0.05</td><td></td><td>35</td><td>35</td><td>0</td><td>0</td><td>yes</td><td></td><td></td><td></td><td>blast</td><td>gcugguacuuccuucucuggau</td><td>ccagagaaggaaguaccagccc</td><td>gcugguacuuccuucucuggauacauacucuauauccagagaaggaaguaccagccc</td><td>gi|307603376|gb|CM001000.1|:146032672..146</td></tr>
<tr><td>gi|307603371|gb|CM001005.1|_7090</td><td>3.0</td><td>0.64 ± 0.05</td><td></td><td>52</td><td>52</td><td>0</td><td>0</td><td>yes</td><td></td><td></td><td></td><td>blast</td><td>uuuggguuuaaacgugugugcca</td><td>gcacgccuuuaaucccaacac</td><td>uuuggguuuaaacgugugugccacgccgggcgugguggggcacgccuuuaaucccaacac</td><td>gi|307603371|gb|CM001005.1|:105991080..105</td></tr>
<tr><td>gi|307603381|gb|CM000995.1|_25375</td><td>3.0</td><td>0.64 ± 0.05</td><td></td><td>264</td><td>264</td><td>0</td><td>0</td><td>yes</td><td></td><td></td><td></td><td>blast</td><td>agacggcgcgagggggagcga</td><td>ccuccuccugccgaggcugu</td><td>ccuccuccugccgaggcugugcaccgcagacggcgcgagggggagcga</td><td>gi|307603381|gb|CM000995.1|:83652767..8365</td></tr>
<tr><td>gi|307603366|gb|CM001010.1|_18040</td><td>3.0</td><td>0.64 ± 0.05</td><td></td><td>33</td><td>33</td><td>0</td><td>0</td><td>yes</td><td></td><td></td><td></td><td>blast</td><td>uucgcgggagcugcuuggaggcu</td><td>ccugggagcggccggggaca</td><td>ccugggagcggccggggacagcgcgggccuguguguccgcggcgccgggguucgcgggagcugcuuggaggcu</td><td>gi|307603366|gb|CM001010.1|:66300434..6630</td></tr>
<tr><td>gi|307603376|gb|CM001000.1|_42727</td><td>3.0</td><td>0.64 ± 0.05</td><td></td><td>32</td><td>32</td><td>0</td><td>0</td><td>yes</td><td></td><td></td><td></td><td>blast</td><td>uggccgaggcggcagcugu</td><td>ggccgcagcggaaagc</td><td>uggccgaggcggcagcuguggcggccgcugccgcgguggcggcggccgccgcggccgcagcggaaagc</td><td>gi|307603376|gb|CM001000.1|:148513364..148</td></tr>
<tr><td>gi|307603379|gb|CM000997.1|_32348</td><td>3.0</td><td>0.64 ± 0.05</td><td></td><td>54</td><td>42</td><td>12</td><td>0</td><td>yes</td><td></td><td></td><td></td><td>blast</td><td>cuggcgggcgggcgggcg</td><td>cccgcccagcucgcccgcgcg</td><td>cccgcccagcucgcccgcgcgcgccgcgcccugcccucagugcgcgcggacgggcuggcgggcgggcgggcg</td><td>gi|307603379|gb|CM000997.1|:11118751..1111</td></tr>
<tr><td>gi|307603373|gb|CM001003.1|_953</td><td>3.0</td><td>0.64 ± 0.05</td><td></td><td>85</td><td>85</td><td>0</td><td>0</td><td>yes</td><td></td><td></td><td></td><td>blast</td><td>uaagucuagggcuccgccagc</td><td>uggggagcugggggcgcggc</td><td>uggggagcugggggcgcggcuucgaccagccggggggcggggcucgaggccuaagucuagggcuccgccagc</td><td>gi|307603373|gb|CM001003.1|:95026900..9502</td></tr>
<tr><td>gi|307603368|gb|CM001008.1|_13959</td><td>3.0</td><td>0.64 ± 0.05</td><td></td><td>35</td><td>35</td><td>0</td><td>0</td><td>yes</td><td></td><td></td><td></td><td>blast</td><td>uggcacaggggugucuagggacc</td><td>ucccacacacgccccuuugcccgc</td><td>uggcacaggggugucuagggaccgcgggaagucuugaggucccacacacgccccuuugcccgc</td><td>gi|307603368|gb|CM001008.1|:79659410..7965</td></tr>
<tr><td>gi|307603374|gb|CM001002.1|_47358</td><td>2.9</td><td>0.81 ± 0.02</td><td></td><td>30</td><td>29</td><td>1</td><td>0</td><td>yes</td><td></td><td></td><td></td><td>blast</td><td>ccuuuguuucgcgcggcccgac</td><td>cccggcgcgcggggcgaggga</td><td>cccggcgcgcggggcgaggggacgcggcgcggcgcggcgggcgcgguggcggcuccuuuguuucgcgcggcccgac</td><td>gi|307603374|gb|CM001002.1|:66440376..6644</td></tr>
<tr><td>gi|307603372|gb|CM001004.1|_5515</td><td>2.9</td><td>0.81 ± 0.02</td><td></td><td>34</td><td>34</td><td>0</td><td>0</td><td>yes</td><td></td><td></td><td></td><td>blast</td><td>auuuugucuucucggcuccucuc</td><td>gagugggccgagggggcuugg</td><td>auuuugucuucucggcuccucucuggaagaggcuuuucgagcuugagagugggccgagggggcuugg</td><td>gi|307603372|gb|CM001004.1|:94183182..9418</td></tr>
<tr><td>gi|307603363|gb|CM001013.1|_48086</td><td>2.9</td><td>0.81 ± 0.02</td><td></td><td>37</td><td>37</td><td>0</td><td>0</td><td>yes</td><td></td><td></td><td></td><td>blast</td><td>cucguuggggcagcauggcg</td><td>ccgcugcucugcgggcc</td><td>cucguuggggcagcauggcggggucgccgcugcucugcgggcc</td><td>gi|307603363|gb|CM001013.1|:7497042..74970</td></tr>
<tr><td>gi|307603375|gb|CM001001.1|_42831</td><td>2.9</td><td>0.81 ± 0.02</td><td></td><td>42</td><td>42</td><td>0</td><td>0</td><td>yes</td><td></td><td></td><td></td><td>blast</td><td>gccgcuggccgaggggg</td><td>cucggccggccccucucgcuc</td><td>cucggccggccccucucgcucccccgggagugccuggcggggccgcugggccgaggggg</td><td>gi|307603375|gb|CM001001.1|:3493143..34932</td></tr>
<tr><td>gi|307603380|gb|CM000996.1|_29549</td><td>2.9</td><td>0.81 ± 0.02</td><td></td><td>309</td><td>309</td><td>0</td><td>0</td><td>yes</td><td></td><td></td><td></td><td>blast</td><td>ccgcuguggcgcuuccugcg</td><td>cgggaaguggaggagcggga</td><td>cgggaaguggaggagcgggaggcggggacccaccuggaagcgccgcggcgccgcuguggcgcuuccugcg</td><td>gi|307603380|gb|CM000996.1|:152058950..152</td></tr>
<tr><td>gi|307603380|gb|CM000996.1|_28579</td><td>2.9</td><td>0.81 ± 0.02</td><td></td><td>36</td><td>36</td><td>0</td><td>0</td><td>yes</td><td></td><td></td><td></td><td>blast</td><td>gggcucggcguggcgcuggc</td><td>ccgcgcuuccgcaagugugg</td><td>ccgcgcuuccgcaaguguggagcugcgggauggcuacgcugcuacugcagcugcuggggcucggcguggcgcuggc</td><td>gi|307603380|gb|CM000996.1|:54539476..5453</td></tr>
<tr><td>gi|307603377|gb|CM000999.1|_38326</td><td>2.9</td><td>0.81 ± 0.02</td><td></td><td>75</td><td>49</td><td>26</td><td>0</td><td>yes</td><td></td><td></td><td></td><td>blast</td><td>cggcugccggcgcgggcu</td><td>cucguggcuccacccgcc</td><td>cucguggcuccacccgccauuguguaagugcgcggcugccggcgcgggcu</td><td>gi|307603377|gb|CM000999.1|:43616107..4361</td></tr>
<tr><td>gi|307603379|gb|CM000997.1|_33643</td><td>2.9</td><td>0.81 ± 0.02</td><td></td><td>59</td><td>59</td><td>0</td><td>0</td><td>yes</td><td></td><td></td><td></td><td>blast</td><td>gacgcgucccgggcaccggc</td><td>cggggggcgcggggccgg</td><td>gacgcgucccgggcaccggcggccgccggggggcgcggggccgg</td><td>gi|307603379|gb|CM000997.1|:146230754..146</td></tr>
<tr><td>gi|307603373|gb|CM001003.1|_2016</td><td>2.9</td><td>0.81 ± 0.02</td><td></td><td>37</td><td>37</td><td>0</td><td>0</td><td>yes</td><td></td><td></td><td></td><td>blast</td><td>cuagcggcggcugugggcugg</td><td>cgcccgccgccgccgcgcgcg</td><td>cgcccgccgccgccgcgcgcgccacucacccuagccgcgcgccacuagcggcggcugugggcugg</td><td>gi|307603373|gb|CM001003.1|:90292389..9029</td></tr>
<tr><td>gi|307603365|gb|CM001011.1|_18987</td><td>2.9</td><td>0.81 ± 0.02</td><td></td><td>33</td><td>33</td><td>0</td><td>0</td><td>yes</td><td></td><td></td><td></td><td>blast</td><td>cuccgcgccgcgggcgcucugc</td><td>agggcgcagcuggcgcggucgguc</td><td>agggcgcagcuggcgcggucggucuagagcagcgcucggcuccgcgccgcgggcgcucugc</td><td>gi|307603365|gb|CM001011.1|:83080260..8308</td></tr>
<tr><td>gi|307603366|gb|CM001010.1|_16450</td><td>2.9</td><td>0.81 ± 0.02</td><td></td><td>66</td><td>66</td><td>0</td><td>0</td><td>yes</td><td></td><td></td><td></td><td>blast</td><td>agugccggucgggccaugggcg</td><td>cucgcggcccgccggagcugg</td><td>agugccggucgggccaugggcgagcccgaccccuuggucucggggcagcucgcggcccgccggagcugg</td><td>gi|307603366|gb|CM001010.1|:29751897..2975</td></tr>
<tr><td>gi|307603379|gb|CM000997.1|_32412</td><td>2.9</td><td>0.81 ± 0.02</td><td></td><td>32</td><td>32</td><td>0</td><td>0</td><td>yes</td><td></td><td></td><td></td><td>blast</td><td>ugcgcuccucguccgacccuc</td><td>gggguggagaggggcggugc</td><td>gggguggagaggggcggugcuccaaguggagcugcgcuccucguccgacccuc</td><td>gi|307603379|gb|CM000997.1|:22418609..2241</td></tr>
<tr><td>gi|307603366|gb|CM001010.1|_18039</td><td>2.9</td><td>0.81 ± 0.02</td><td></td><td>33</td><td>33</td><td>0</td><td>0</td><td>yes</td><td></td><td></td><td></td><td>blast</td><td>uucgcgggagcugcuuggaggcu</td><td>gggccacggugagcgagcu</td><td>uucgcgggagcugcuuggaggcucggcggccgggaggaggccggggccacggugagcgagcu</td><td>gi|307603366|gb|CM001010.1|:66300395..6630</td></tr>
<tr><td>gi|307603369|gb|CM001007.1|_11712</td><td>2.8</td><td>0.81 ± 0.02</td><td></td><td>204</td><td>204</td><td>0</td><td>0</td><td>yes</td><td></td><td></td><td></td><td>blast</td><td>cggccguucgggcuccggc</td><td>ugggacggcccauguggaagca</td><td>ugggacggcccauguggaagcaggugagagccccacaccugcggccguucgggcuccggc</td><td>gi|307603369|gb|CM001007.1|:22667186..2266</td></tr>
<tr><td>gi|307603368|gb|CM001008.1|_12808</td><td>2.8</td><td>0.81 ± 0.02</td><td></td><td>114</td><td>114</td><td>0</td><td>0</td><td>yes</td><td></td><td></td><td></td><td>blast</td><td>ucuuuugcuagaugcugugcca</td><td>gcacgggugaucuagcagaagaug</td><td>ucuuuugcuagaugcugugccaaauccuggagauggcacgggugaucuagcagaagaug</td><td>gi|307603368|gb|CM001008.1|:58767452..5876</td></tr>
<tr><td>gi|307603382|gb|CM000994.1|_24064</td><td>2.8</td><td>0.81 ± 0.02</td><td></td><td>56</td><td>56</td><td>0</td><td>0</td><td>yes</td><td></td><td></td><td></td><td>blast</td><td>cacagcaaguguagacaggca</td><td>ccugucugugccugcuguaca</td><td>ccugucugugccugcuguacaggugagcggauguucugcacagcaaguguagacaggca</td><td>gi|307603382|gb|CM000994.1|:164153527..164</td></tr>
<tr><td>gi|307603381|gb|CM000995.1|_27878</td><td>2.8</td><td>0.81 ± 0.02</td><td></td><td>33</td><td>33</td><td>0</td><td>0</td><td>yes</td><td></td><td></td><td></td><td>blast</td><td>uggcacagaaggcucucugg</td><td>aggccugggcuggcugagcaaac</td><td>aggccugggcuggcugagcaaacgguggcacagaaggcucucugg</td><td>gi|307603381|gb|CM000995.1|:151882294..151</td></tr>
<tr><td>gi|307603363|gb|CM001013.1|_48524</td><td>2.8</td><td>0.81 ± 0.02</td><td></td><td>110</td><td>110</td><td>0</td><td>0</td><td>yes</td><td></td><td></td><td></td><td>blast</td><td>uuucgggcuccagacaucugucc</td><td>auaggugucugcccagcccuagag</td><td>uuucgggcuccagacaucuguccaccuuccuggccagaggauaggugucugcccagcccuagag</td><td>gi|307603363|gb|CM001013.1|:93044117..9304</td></tr>
<tr><td>gi|307603381|gb|CM000995.1|_24778</td><td>2.8</td><td>0.81 ± 0.02</td><td></td><td>49</td><td>49</td><td>0</td><td>0</td><td>yes</td><td></td><td></td><td></td><td>blast</td><td>agguugggcugcuugcugccucc</td><td>cggcagcaggcggucgccagg</td><td>agguugggcugcuugcugccucccgugugcagcggcggcagcaggcggucgccagg</td><td>gi|307603381|gb|CM000995.1|:25111071..2511</td></tr>
<tr><td>gi|307603376|gb|CM001000.1|_39538</td><td>2.8</td><td>0.81 ± 0.02</td><td></td><td>35</td><td>35</td><td>0</td><td>0</td><td>yes</td><td></td><td></td><td></td><td>blast</td><td>gccgccgcggucgcaucgg</td><td>aacccgacggcggcggccc</td><td>gccgccgcggucgcaucggccgccuccuccugccgcccucugggcucgggcacggcucccaacccgacggcggcggccc</td><td>gi|307603376|gb|CM001000.1|:17401719..1740</td></tr>
<tr><td>gi|307603366|gb|CM001010.1|_17042</td><td>2.8</td><td>0.81 ± 0.02</td><td></td><td>69</td><td>42</td><td>27</td><td>0</td><td>yes</td><td></td><td></td><td></td><td>blast</td><td>gccgggggcggcgggaug</td><td>ugagccgcugccuacccggcgc</td><td>gccgggggcggcgggaugcugcugcggaugcggagcugugugagccgcugccuacccggcgc</td><td>gi|307603366|gb|CM001010.1|:74154214..7415</td></tr>
<tr><td>gi|307603381|gb|CM000995.1|_25378</td><td>2.8</td><td>0.81 ± 0.02</td><td></td><td>29</td><td>29</td><td>0</td><td>0</td><td>yes</td><td></td><td></td><td></td><td>blast</td><td>aggccggaggugccuggggc</td><td>uccagguacgucucgguagcc</td><td>aggccggaggugccuggggcauccucuacuuugguggcuccagguacgucucgguagcc</td><td>gi|307603381|gb|CM000995.1|:83653091..8365</td></tr>
<tr><td>gi|307603373|gb|CM001003.1|_226</td><td>2.8</td><td>0.81 ± 0.02</td><td></td><td>87</td><td>59</td><td>0</td><td>28</td><td>yes</td><td></td><td></td><td></td><td>blast</td><td>gcgguggcggcggcggcg</td><td>gccgccgcugcugccagg</td><td>gccgccgcugcugccaggucgcugaguggaggggaggcgguggcgguggcggcggcggcg</td><td>gi|307603373|gb|CM001003.1|:39452217..3945</td></tr>
<tr><td>gi|307603378|gb|CM000998.1|_34523</td><td>2.8</td><td>0.81 ± 0.02</td><td></td><td>235</td><td>204</td><td>31</td><td>0</td><td>yes</td><td></td><td></td><td></td><td>blast</td><td>aggcggcgguggcggagg</td><td>gguguccgcggcuguuucc</td><td>gguguccgcggcuguuuccgggcucgggggggccggagaggcugcugaguaggaggcggcgguggcggagg</td><td>gi|307603378|gb|CM000998.1|:92566946..9256</td></tr>
<tr><td>gi|307603372|gb|CM001004.1|_5550</td><td>2.8</td><td>0.81 ± 0.02</td><td></td><td>222</td><td>222</td><td>0</td><td>0</td><td>yes</td><td></td><td></td><td></td><td>blast</td><td>cucgguggugcgggagcagcggc</td><td>cggagccagcgccacccggg</td><td>cggagccagcgccacccgggcuggggaagccgaggguggggacacucuggcccggcucucgguggugcgggagcagcggc</td><td>gi|307603372|gb|CM001004.1|:95926638..9592</td></tr>
<tr><td>gi|307603364|gb|CM001012.1|_19772</td><td>2.7</td><td>0.81 ± 0.02</td><td></td><td>45</td><td>35</td><td>10</td><td>0</td><td>yes</td><td></td><td></td><td></td><td>blast</td><td>aggcgguggugaggacccg</td><td>ugcucucgcugcgguaccucc</td><td>aggcgguggugaggacccggcgggugguagcucaggcccgcacccgcccugcucucgcugcgguaccucc</td><td>gi|307603364|gb|CM001012.1|:4625898..46259</td></tr>
<tr><td>gi|307603364|gb|CM001012.1|_20362</td><td>2.7</td><td>0.81 ± 0.02</td><td></td><td>57</td><td>57</td><td>0</td><td>0</td><td>yes</td><td></td><td></td><td></td><td>blast</td><td>gccgucccuagaguagagau</td><td>cuggcucuggagaggccc</td><td>gccgucccuagaguagagauggcggcgacggcagccgagguugcggccucuggcucuggagaggccc</td><td>gi|307603364|gb|CM001012.1|:44637349..4463</td></tr>
<tr><td>gi|307603369|gb|CM001007.1|_11068</td><td>2.7</td><td>0.81 ± 0.02</td><td></td><td>36</td><td>36</td><td>0</td><td>0</td><td>yes</td><td></td><td></td><td></td><td>blast</td><td>gcggucgcgcuaccuccg</td><td>caggugggcggcugccc</td><td>gcggucgcgcuaccuccggcccaggugggcggcugccc</td><td>gi|307603369|gb|CM001007.1|:51550761..5155</td></tr>
<tr><td>gi|307603373|gb|CM001003.1|_2164</td><td>2.7</td><td>0.81 ± 0.02</td><td></td><td>126</td><td>126</td><td>0</td><td>0</td><td>yes</td><td></td><td></td><td></td><td>blast</td><td>uccggcugggggguagcaguagc</td><td>ugcugcuccgcucggccuucaggc</td><td>ugcugcuccgcucggccuucaggcuuccagacgcuccggcugggggguagcaguagc</td><td>gi|307603373|gb|CM001003.1|:110943816..110</td></tr>
<tr><td>gi|307603381|gb|CM000995.1|_26887</td><td>2.7</td><td>0.81 ± 0.02</td><td></td><td>31</td><td>31</td><td>0</td><td>0</td><td>yes</td><td></td><td></td><td></td><td>blast</td><td>ggggcguggccucggacccuga</td><td>agggcccuucugcgccccac</td><td>ggggcguggccucggacccugagccucugauugggcggaggucucagggcccuucugcgccccac</td><td>gi|307603381|gb|CM000995.1|:35946937..3594</td></tr>
<tr><td>gi|307603379|gb|CM000997.1|_32874</td><td>2.7</td><td>0.81 ± 0.02</td><td></td><td>37</td><td>37</td><td>0</td><td>0</td><td>yes</td><td></td><td></td><td></td><td>blast</td><td>gguacggcccagcgcuagcg</td><td>uagcgccacccgugccug</td><td>uagcgccacccgugccugcgcgagcacuugguacggcccagcgcuagcg</td><td>gi|307603379|gb|CM000997.1|:83132360..8313</td></tr>
<tr><td>gi|307603375|gb|CM001001.1|_45058</td><td>2.7</td><td>0.81 ± 0.02</td><td></td><td>59</td><td>30</td><td>29</td><td>0</td><td>yes</td><td></td><td></td><td></td><td>blast</td><td>acugucugcgugggucggg</td><td>ugcgccuacgccagcua</td><td>ugcgccuacgccagcuacgcugggcgcucgcugggcacacgcugggcgagcaucuguuaacugucugcgugggucggg</td><td>gi|307603375|gb|CM001001.1|:124114147..124</td></tr>
<tr><td>gi|307603366|gb|CM001010.1|_16986</td><td>2.7</td><td>0.81 ± 0.02</td><td></td><td>36</td><td>36</td><td>0</td><td>0</td><td>yes</td><td></td><td></td><td></td><td>blast</td><td>gcggcggcgacaacaugc</td><td>acgggagccgcgcuc</td><td>gcggcggcgacaacaugcgcggcagcggcacgggagccgcgcuc</td><td>gi|307603366|gb|CM001010.1|:68046660..6804</td></tr>
<tr><td>gi|307603367|gb|CM001009.1|_14640</td><td>2.7</td><td>0.81 ± 0.02</td><td></td><td>32</td><td>32</td><td>0</td><td>0</td><td>yes</td><td></td><td></td><td></td><td>blast</td><td>ccgggcugguccgcacca</td><td>aagcggacagcuccggag</td><td>ccgggcugguccgcaccaccacuaccggcgncggcgugguggcugaagcggacagcuccggag</td><td>gi|307603367|gb|CM001009.1|:20620015..2062</td></tr>
<tr><td>gi|307603379|gb|CM000997.1|_33752</td><td>2.7</td><td>0.81 ± 0.02</td><td></td><td>54</td><td>54</td><td>0</td><td>0</td><td>yes</td><td></td><td></td><td></td><td>blast</td><td>accgguuggcuuggcagcg</td><td>cugagacagcuagcaggagg</td><td>cugagacagcuagcaggagggcgggggucucccggccuccugccaaccgguuggcuuggcagcg</td><td>gi|307603379|gb|CM000997.1|:150503479..150</td></tr>
<tr><td>gi|307603376|gb|CM001000.1|_41165</td><td>2.7</td><td>0.81 ± 0.02</td><td></td><td>32</td><td>32</td><td>0</td><td>0</td><td>yes</td><td></td><td></td><td></td><td>blast</td><td>gaagugaagcugggcccu</td><td>ggcucggcaaucuccu</td><td>gaagugaagcugggcccuacggcucggcaaucuccu</td><td>gi|307603376|gb|CM001000.1|:25372606..2537</td></tr>
<tr><td>gi|307603364|gb|CM001012.1|_20396</td><td>2.7</td><td>0.81 ± 0.02</td><td></td><td>55</td><td>54</td><td>0</td><td>1</td><td>yes</td><td></td><td></td><td></td><td>blast</td><td>gccagagcaggucgcagcug</td><td>cagcggcagacuugggcca</td><td>gccagagcaggucgcagcuggugaagcgccgcagccagcggcagacuugggcca</td><td>gi|307603364|gb|CM001012.1|:45734320..4573</td></tr>
<tr><td>gi|307603363|gb|CM001013.1|_48494</td><td>2.7</td><td>0.81 ± 0.02</td><td></td><td>52</td><td>52</td><td>0</td><td>0</td><td>yes</td><td></td><td></td><td></td><td>blast</td><td>guggcccaccgacaccgucgag</td><td>ccaccauguuugugggccgcgg</td><td>guggcccaccgacaccgucgagaggcgggaggcccgggccacccccccaccauguuugugggccgcgg</td><td>gi|307603363|gb|CM001013.1|:91368845..9136</td></tr>
<tr><td>gi|307603375|gb|CM001001.1|_43794</td><td>2.6</td><td>0.81 ± 0.02</td><td></td><td>99</td><td>99</td><td>0</td><td>0</td><td>yes</td><td></td><td></td><td></td><td>blast</td><td>ggaucccccugcuguggacugugc</td><td>acaguugcacaguugggggcagcca</td><td>ggaucccccugcuguggacugugcugcggagucuuguucacaguugcacaguugggggcagcca</td><td>gi|307603375|gb|CM001001.1|:119863696..119</td></tr>
<tr><td>gi|307603382|gb|CM000994.1|_23155</td><td>2.6</td><td>0.81 ± 0.02</td><td></td><td>153</td><td>153</td><td>0</td><td>0</td><td>yes</td><td></td><td></td><td></td><td>blast</td><td>acugacccgaaccgcugugcugu</td><td>agcacccaggguuaggguuagggu</td><td>acugacccgaaccgcugugcugugcuguccacagcacccaggguuaggguuagggu</td><td>gi|307603382|gb|CM000994.1|:43884117..4388</td></tr>
<tr><td>gi|307603372|gb|CM001004.1|_4039</td><td>2.6</td><td>0.81 ± 0.02</td><td></td><td>219</td><td>219</td><td>0</td><td>0</td><td>yes</td><td></td><td></td><td></td><td>blast</td><td>cgcccgcgagugugccugccc</td><td>uuccggcuuccggucucagggcccgcc</td><td>uuccggcuuccggucucagggcccgcccugcugaguagggcgcccgcgagugugccugccc</td><td>gi|307603372|gb|CM001004.1|:103559026..103</td></tr>
<tr><td>gi|307603375|gb|CM001001.1|_43316</td><td>2.6</td><td>0.81 ± 0.02</td><td></td><td>29</td><td>29</td><td>0</td><td>0</td><td>yes</td><td></td><td></td><td></td><td>blast</td><td>cgccauggcagcacccgc</td><td>gucgcuggcgcagcugg</td><td>cgccauggcagcacccgcgcccagucucaucucggucuucuccagcccgcaagagcugggcgcgucgcuggcgcagcugg</td><td>gi|307603375|gb|CM001001.1|:74116111..7411</td></tr>
<tr><td>gi|307603382|gb|CM000994.1|_22050</td><td>2.6</td><td>0.81 ± 0.02</td><td></td><td>28</td><td>28</td><td>0</td><td>0</td><td>yes</td><td></td><td></td><td></td><td>blast</td><td>ugcacagcagguggccgagcacu</td><td>ggccagccacugccugugcgug</td><td>ugcacagcagguggccgagcacucaccuggagcagagaguggugaggccagccacugccugugcgug</td><td>gi|307603382|gb|CM000994.1|:89688556..8968</td></tr>
<tr><td>gi|307603371|gb|CM001005.1|_7514</td><td>2.6</td><td>0.81 ± 0.02</td><td></td><td>149</td><td>149</td><td>0</td><td>0</td><td>yes</td><td></td><td></td><td></td><td>blast</td><td>aguaagucgccaccugggg</td><td>ccgguggcgcccugg</td><td>ccgguggcgcccugggcugucgccugcgacggcgacgucuguuccgcaguaagucgccaccugggg</td><td>gi|307603371|gb|CM001005.1|:4914031..49140</td></tr>
<tr><td>gi|307603379|gb|CM000997.1|_33797</td><td>2.6</td><td>0.81 ± 0.02</td><td></td><td>31</td><td>31</td><td>0</td><td>0</td><td>yes</td><td></td><td></td><td></td><td>blast</td><td>aggccaucuggggagcag</td><td>ucccucuaggcagccuuu</td><td>aggccaucuggggagcaguccuccgcgcgggcagggugggggcuucccucuaggcagccuuu</td><td>gi|307603379|gb|CM000997.1|:154323815..154</td></tr>
<tr><td>gi|307603371|gb|CM001005.1|_6886</td><td>2.6</td><td>0.81 ± 0.02</td><td></td><td>35</td><td>35</td><td>0</td><td>0</td><td>yes</td><td></td><td></td><td></td><td>blast</td><td>aggcccaaggccggagcugc</td><td>ggcggggggguggucugc</td><td>aggcccaaggccggagcugccggggcgggggcggggggguggucugc</td><td>gi|307603371|gb|CM001005.1|:85455489..8545</td></tr>
<tr><td>gi|307603367|gb|CM001009.1|_14839</td><td>2.6</td><td>0.81 ± 0.02</td><td></td><td>511</td><td>491</td><td>20</td><td>0</td><td>yes</td><td></td><td></td><td></td><td>blast</td><td>cucccaggggcgcgucgacggug</td><td>aggucgacgccuugauuc</td><td>aggucgacgccuugauucagccuuccgucucacggcgaguggcugacucccaggggcgcgucgacggug</td><td>gi|307603367|gb|CM001009.1|:36875199..3687</td></tr>
<tr><td>gi|307603375|gb|CM001001.1|_44884</td><td>2.6</td><td>0.81 ± 0.02</td><td></td><td>29</td><td>29</td><td>0</td><td>0</td><td>yes</td><td></td><td></td><td></td><td>blast</td><td>cacugccguggcggccugggcg</td><td>ucucaggcucgcccucgggccugcc</td><td>ucucaggcucgcccucgggccugcccacaguggagccccggggcacugccguggcggccugggcg</td><td>gi|307603375|gb|CM001001.1|:108129510..108</td></tr>
<tr><td>gi|307603365|gb|CM001011.1|_18617</td><td>2.6</td><td>0.81 ± 0.02</td><td></td><td>69</td><td>69</td><td>0</td><td>0</td><td>yes</td><td></td><td></td><td></td><td>blast</td><td>cugcugggcucuuggcacuccuuu</td><td>auccgggugcccagggcucagcacuc</td><td>auccgggugcccagggcucagcacucugagaauguggcugcugggcucuuggcacuccuuu</td><td>gi|307603365|gb|CM001011.1|:38675179..3867</td></tr>
<tr><td>gi|307603378|gb|CM000998.1|_36543</td><td>2.6</td><td>0.81 ± 0.02</td><td></td><td>33</td><td>33</td><td>0</td><td>0</td><td>yes</td><td></td><td></td><td></td><td>blast</td><td>gcugguuggaggggcgag</td><td>gguccucuacaggaa</td><td>gcugguuggaggggcgagcgcagcugguccucuacaggaa</td><td>gi|307603378|gb|CM000998.1|:136720098..136</td></tr>
<tr><td>gi|307603370|gb|CM001006.1|_9558</td><td>2.6</td><td>0.81 ± 0.02</td><td></td><td>81</td><td>81</td><td>0</td><td>0</td><td>yes</td><td></td><td></td><td></td><td>blast</td><td>cacgccgcggcucacacacacagu</td><td>ugugucguguggcacgccgcggcucaca</td><td>ugugucguguggcacgccgcggcucacacacacaguuagccaucacaacugugucguguggcacgccgcggcucacacacacagu</td><td>gi|307603370|gb|CM001006.1|:12295771..1229</td></tr>
<tr><td>gi|307603374|gb|CM001002.1|_45946</td><td>2.6</td><td>0.81 ± 0.02</td><td></td><td>33</td><td>33</td><td>0</td><td>0</td><td>yes</td><td></td><td></td><td></td><td>blast</td><td>uuguagcaggcagguccg</td><td>guccugccugugccggcc</td><td>uuguagcaggcagguccggagaggagcgccccugaggcugcagaggccgguccugccugugccggcc</td><td>gi|307603374|gb|CM001002.1|:64188263..6418</td></tr>
<tr><td>gi|307603379|gb|CM000997.1|_32890</td><td>2.6</td><td>0.81 ± 0.02</td><td></td><td>90</td><td>90</td><td>0</td><td>0</td><td>yes</td><td></td><td></td><td></td><td>blast</td><td>uggcgccuccacugucccgagu</td><td>ccgggccggcggaggcucgaccagc</td><td>ccgggccggcggaggcucgaccagcaaguucauugcuggcgccuccacugucccgagu</td><td>gi|307603379|gb|CM000997.1|:84530119..8453</td></tr>
<tr><td>gi|307603375|gb|CM001001.1|_44074</td><td>2.6</td><td>0.81 ± 0.02</td><td></td><td>29</td><td>29</td><td>0</td><td>0</td><td>yes</td><td></td><td></td><td></td><td>blast</td><td>guuguuagcacuguccugcagga</td><td>cugcaggaguuguuagcacugu</td><td>cugcaggaguuguuagcacuguccugcaggaguuguuagcacuguccugcaggaguuguuagcacuguccugcagga</td><td>gi|307603375|gb|CM001001.1|:12651799..1265</td></tr>
<tr><td>gi|307603378|gb|CM000998.1|_34572</td><td>2.6</td><td>0.81 ± 0.02</td><td></td><td>49</td><td>49</td><td>0</td><td>0</td><td>yes</td><td></td><td></td><td></td><td>blast</td><td>accggcggcgccuaucagg</td><td>ugaggguucgguuccucg</td><td>accggcggcgccuaucagguaccugaggcucacgccucggcgucugaggguucgguuccucg</td><td>gi|307603378|gb|CM000998.1|:100335406..100</td></tr>
<tr><td>gi|307603375|gb|CM001001.1|_43347</td><td>2.6</td><td>0.81 ± 0.02</td><td></td><td>259</td><td>259</td><td>0</td><td>0</td><td>yes</td><td></td><td></td><td></td><td>blast</td><td>cgcacccuucaaguccuggaac</td><td>cucgggacuggagggaugggcu</td><td>cucgggacuggagggaugggcuuccuaacggaagcgcacccuucaaguccuggaac</td><td>gi|307603375|gb|CM001001.1|:75072404..7507</td></tr>
<tr><td>gi|307603381|gb|CM000995.1|_26367</td><td>2.6</td><td>0.81 ± 0.02</td><td></td><td>135</td><td>120</td><td>0</td><td>15</td><td>yes</td><td></td><td></td><td></td><td>blast</td><td>ugccuggcucccugcccucu</td><td>acaggagcauagggcgcagg</td><td>acaggagcauagggcgcaggcagcuugggcgcugugaguggaucgcugccuggcucccugcccucu</td><td>gi|307603381|gb|CM000995.1|:173607905..173</td></tr>
<tr><td>gi|307603376|gb|CM001000.1|_42442</td><td>2.6</td><td>0.81 ± 0.02</td><td></td><td>30</td><td>30</td><td>0</td><td>0</td><td>yes</td><td></td><td></td><td></td><td>blast</td><td>gacuuccggaucgcggcugcg</td><td>aggccgcgacccggaaggaga</td><td>aggccgcgacccggaaggagaaggauuucgagggggcgggccagaaagucgaagcugacuuccggaucgcggcugcg</td><td>gi|307603376|gb|CM001000.1|:125999177..125</td></tr>
<tr><td>gi|307603379|gb|CM000997.1|_32617</td><td>2.6</td><td>0.81 ± 0.02</td><td></td><td>52</td><td>52</td><td>0</td><td>0</td><td>yes</td><td></td><td></td><td></td><td>blast</td><td>accgggcgggcgagcggc</td><td>uucuccacccgccgucc</td><td>accgggcgggcgagcggcucaaaguuugcuucuccacccgccgucc</td><td>gi|307603379|gb|CM000997.1|:46464430..4646</td></tr>
<tr><td>gi|307603373|gb|CM001003.1|_635</td><td>2.6</td><td>0.81 ± 0.02</td><td></td><td>47</td><td>47</td><td>0</td><td>0</td><td>yes</td><td></td><td></td><td></td><td>blast</td><td>ugcucagacccucuccuccacagu</td><td>uggggaggcagagggcugguggc</td><td>uggggaggcagagggcugguggcccugagcacacaugggcggugaggcugcccaaggaggcugcucagacccucuccuccacagu</td><td>gi|307603373|gb|CM001003.1|:79607879..7960</td></tr>
<tr><td>gi|307603381|gb|CM000995.1|_24932</td><td>2.6</td><td>0.81 ± 0.02</td><td></td><td>38</td><td>38</td><td>0</td><td>0</td><td>yes</td><td></td><td></td><td></td><td>blast</td><td>augguucugaaguuccggcucgg</td><td>gagcggagcuuaggccuugg</td><td>augguucugaaguuccggcucggcuccauagaguggagcggagcggagcuuaggccuugg</td><td>gi|307603381|gb|CM000995.1|:30248651..3024</td></tr>
<tr><td>gi|307603380|gb|CM000996.1|_28524</td><td>2.6</td><td>0.81 ± 0.02</td><td></td><td>153</td><td>153</td><td>0</td><td>0</td><td>yes</td><td></td><td></td><td></td><td>blast</td><td>cgccgcugcugcucaaagu</td><td>gcggagcgcggcggcggg</td><td>cgccgcugcugcucaaaguggcggagcgcggcggcggg</td><td>gi|307603380|gb|CM000996.1|:41368807..4136</td></tr>
<tr><td>gi|307603369|gb|CM001007.1|_10824</td><td>2.5</td><td>0.81 ± 0.02</td><td></td><td>34</td><td>34</td><td>0</td><td>0</td><td>yes</td><td></td><td></td><td></td><td>blast</td><td>agggcggacccugcaagag</td><td>gguguugggucuggaccacc</td><td>agggcggacccugcaagagcucaggaaccaaugugugaaagugguucuugagugguguugggucuggaccacc</td><td>gi|307603369|gb|CM001007.1|:21613540..2161</td></tr>
<tr><td>gi|307603372|gb|CM001004.1|_3411</td><td>2.5</td><td>0.81 ± 0.02</td><td></td><td>200</td><td>200</td><td>0</td><td>0</td><td>yes</td><td></td><td></td><td></td><td>blast</td><td>ugcacaccuggagcccagauaag</td><td>cuacugggcugcucugggcgagccgg</td><td>cuacugggcugcucugggcgagccggcugaugucaugugagaagccugcacaccuggagcccagauaag</td><td>gi|307603372|gb|CM001004.1|:77580153..7758</td></tr>
<tr><td>gi|307603379|gb|CM000997.1|_32362</td><td>2.5</td><td>0.81 ± 0.02</td><td></td><td>30</td><td>30</td><td>0</td><td>0</td><td>yes</td><td></td><td></td><td></td><td>blast</td><td>caaacuaaagucuauucugggu</td><td>ccagaauagacuauaguuuggc</td><td>ccagaauagacuauaguuuggccacucaggaagacuuuagguggccaaacuaaagucuauucugggu</td><td>gi|307603379|gb|CM000997.1|:11630218..1163</td></tr>
<tr><td>gi|307603372|gb|CM001004.1|_3039</td><td>2.5</td><td>0.81 ± 0.02</td><td></td><td>73</td><td>73</td><td>0</td><td>0</td><td>yes</td><td></td><td></td><td></td><td>blast</td><td>ugccugcucuucuguucucugga</td><td>cagggagcagagguggaggcugc</td><td>cagggagcagagguggaggcugcagucagcugccugcucuucuguucucugga</td><td>gi|307603372|gb|CM001004.1|:60062204..6006</td></tr>
<tr><td>gi|307603380|gb|CM000996.1|_29904</td><td>2.5</td><td>0.81 ± 0.02</td><td></td><td>42</td><td>42</td><td>0</td><td>0</td><td>yes</td><td></td><td></td><td></td><td>blast</td><td>uuccuaaagugucacuuggcaga</td><td>ugccaagugacacuuaggaaug</td><td>ugccaagugacacuuaggaauguuacuuuuucugacaaaugcacagaaaggaacauuccuaaagugucacuuggcaga</td><td>gi|307603380|gb|CM000996.1|:58379782..5837</td></tr>
<tr><td>gi|307603370|gb|CM001006.1|_10041</td><td>2.5</td><td>0.81 ± 0.02</td><td></td><td>34</td><td>34</td><td>0</td><td>0</td><td>yes</td><td></td><td></td><td></td><td>blast</td><td>uguccauggccugucuuca</td><td>gagacaagccacaggcaua</td><td>uguccauggccugucuucaccaauguguggucccugcuagguggagacaagccacaggcaua</td><td>gi|307603370|gb|CM001006.1|:48914133..4891</td></tr>
<tr><td>gi|307603366|gb|CM001010.1|_16437</td><td>2.5</td><td>0.81 ± 0.02</td><td></td><td>30</td><td>30</td><td>0</td><td>0</td><td>yes</td><td></td><td></td><td></td><td>blast</td><td>ucacaaccucaccccccaacccaga</td><td>ggggugggggugggguggggggacu</td><td>ggggugggggugggguggggggacugugcuagacagucacaaccucacccccaacccaga</td><td>gi|307603366|gb|CM001010.1|:29506030..2950</td></tr>
<tr><td>gi|307603381|gb|CM000995.1|_27877</td><td>2.5</td><td>0.81 ± 0.02</td><td></td><td>33</td><td>33</td><td>0</td><td>0</td><td>yes</td><td></td><td></td><td></td><td>blast</td><td>uggcacagaaggcucucugg</td><td>cgacagccugucuguccacu</td><td>uggcacagaaggcucucugggcacauagccgggccccgacagccugucuguccacu</td><td>gi|307603381|gb|CM000995.1|:151882258..151</td></tr>
<tr><td>gi|307603378|gb|CM000998.1|_34642</td><td>2.5</td><td>0.81 ± 0.02</td><td></td><td>78</td><td>78</td><td>0</td><td>0</td><td>yes</td><td></td><td></td><td></td><td>blast</td><td>uuaggcgaugggcaaccg</td><td>gcucgcccaucaccugcc</td><td>uuaggcgaugggcaaccgggugcacaggagcuccgugcgccgucgcucugcacaccuggcucgcccaucaccugcc</td><td>gi|307603378|gb|CM000998.1|:108297504..108</td></tr>
<tr><td>gi|307603376|gb|CM001000.1|_42677</td><td>2.5</td><td>0.81 ± 0.02</td><td></td><td>75</td><td>75</td><td>0</td><td>0</td><td>yes</td><td></td><td></td><td></td><td>blast</td><td>gccgcauggacagccugg</td><td>ggagcugccagcggcag</td><td>gccgcauggacagccuggcgucgggccgcuggagacggcggaggacggaggagcugccagcggcag</td><td>gi|307603376|gb|CM001000.1|:144602330..144</td></tr>
<tr><td>gi|307603382|gb|CM000994.1|_22043</td><td>2.5</td><td>0.81 ± 0.02</td><td></td><td>44</td><td>44</td><td>0</td><td>0</td><td>yes</td><td></td><td></td><td></td><td>blast</td><td>gugcccugugugggaagguga</td><td>aucuucccggaucgccu</td><td>aucuucccggaucgccuccgggugcccugugugggaagguga</td><td>gi|307603382|gb|CM000994.1|:89652622..8965</td></tr>
<tr><td>gi|307603378|gb|CM000998.1|_34958</td><td>2.5</td><td>0.81 ± 0.02</td><td></td><td>102</td><td>102</td><td>0</td><td>0</td><td>yes</td><td></td><td></td><td></td><td>blast</td><td>uacuaccaccuccuggacuuuu</td><td>aaggccaggagguguaguuca</td><td>uacuaccaccuccuggacuuuuggugcuuuuuaaaaaaaaggccaggagguguaguuca</td><td>gi|307603378|gb|CM000998.1|:123972646..123</td></tr>
<tr><td>gi|307603378|gb|CM000998.1|_34924</td><td>2.5</td><td>0.81 ± 0.02</td><td></td><td>39</td><td>39</td><td>0</td><td>0</td><td>yes</td><td></td><td></td><td></td><td>blast</td><td>aggcgacaggaagcgagg</td><td>ucgguuccugcgagaug</td><td>aggcgacaggaagcgaggcccgacucgcgcaggggcgaacaggccgcaaguuugggagugggucucgguuccugcgagaug</td><td>gi|307603378|gb|CM000998.1|:123229540..123</td></tr>
<tr><td>gi|307603366|gb|CM001010.1|_18001</td><td>2.5</td><td>0.81 ± 0.02</td><td></td><td>43</td><td>43</td><td>0</td><td>0</td><td>yes</td><td></td><td></td><td></td><td>blast</td><td>auccaccggcugacucuc</td><td>uagcuccgccgguggaugg</td><td>auccaccggcugacucucacagugagccccgcaggcuuggccucguugucuccccccauagcuccgccgguggaugg</td><td>gi|307603366|gb|CM001010.1|:57386877..5738</td></tr>
</table>

<table>
<tr><td></td><td></td><td></td><td></td><td></td><td></td><td></td><td></td><td></td><td></td><td></td><td></td><td></td><td></td><td></td><td></td><td></td></tr>
<tr><td>gi|307603373|gb|CM001003.1|_1920</td><td>2.5</td><td>0.81 ± 0.02</td><td></td><td>76</td><td>76</td><td>0</td><td>0</td><td>yes</td><td></td><td></td><td></td><td>blast</td><td>ucaucacccccucuguccuaca</td><td>ugggaggggagaguggggugaagggag</td><td>ugggaggggagagugggggugaagggagagagcggcucugcuugaggucuucaucacccccucuguccuaca</td><td>gi|307603373|gb|CM001003.1|:80809223..8080</td></tr>
<tr><td>gi|307603366|gb|CM001010.1|_16755</td><td>2.5</td><td>0.81 ± 0.02</td><td></td><td>39</td><td>39</td><td>0</td><td>0</td><td>yes</td><td></td><td></td><td></td><td>blast</td><td>aucuggaaagggaaagcgcggug</td><td>cucgugccugcucugucucuccuc</td><td>cucgugccugcucugucucucuccccccgcccuccacugggagggcagaaagaucuggaaagggaaagcgcggug</td><td>gi|307603366|gb|CM001010.1|:45709604..4570</td></tr>
<tr><td>gi|307603376|gb|CM001000.1|_39902</td><td>2.5</td><td>0.81 ± 0.02</td><td></td><td>75</td><td>75</td><td>0</td><td>0</td><td>yes</td><td></td><td></td><td></td><td>blast</td><td>agggaccauugggagccagagg</td><td>ucugccacccaguggccuug</td><td>agggaccauugggagccagaggguaaguccugaggccagacccccucugccacccaguggccuug</td><td>gi|307603376|gb|CM001000.1|:51698936..5169</td></tr>
<tr><td>gi|307603372|gb|CM001004.1|_2509</td><td>2.5</td><td>0.81 ± 0.02</td><td></td><td>31</td><td>31</td><td>0</td><td>0</td><td>yes</td><td></td><td></td><td></td><td>blast</td><td>uuucucugccuggaugcacuga</td><td>ggucauccaggcagagagagg</td><td>ggucauccaggcagagagaggugcugaacuccuugugugucugccuuucucugccuggaugcacuga</td><td>gi|307603372|gb|CM001004.1|:11722640..1172</td></tr>
<tr><td>gi|307603371|gb|CM001005.1|_7881</td><td>2.5</td><td>0.81 ± 0.02</td><td></td><td>222</td><td>222</td><td>0</td><td>0</td><td>yes</td><td></td><td></td><td></td><td>blast</td><td>gugucauggcugcugcugc</td><td>agcggcagcgagaggagccu</td><td>gugucauggcugcugcugcggagcuuaguuccucaagcggcagcgagaggagccu</td><td>gi|307603371|gb|CM001005.1|:57446737..5744</td></tr>
<tr><td>gi|307603381|gb|CM000995.1|_26663</td><td>2.5</td><td>0.81 ± 0.02</td><td></td><td>29</td><td>29</td><td>0</td><td>0</td><td>yes</td><td></td><td></td><td></td><td>blast</td><td>gacagcuagaggcucuuguucagg</td><td>agcauaggcgcguuagguguguc</td><td>gacagcuagaggcucuuguucaggagcugggcgcagaguucugcacagacagagcgcuccgcccagcauaggcgcguuaggugugcc</td><td>gi|307603381|gb|CM000995.1|:23176637..2317</td></tr>
<tr><td>gi|307603369|gb|CM001007.1|_12249</td><td>2.5</td><td>0.81 ± 0.02</td><td></td><td>206</td><td>206</td><td>0</td><td>0</td><td>yes</td><td></td><td></td><td></td><td>blast</td><td>uuggggacccugagcgcggcg</td><td>ccgcggugaguguugagg</td><td>uuggggacccugagcgcggcggcaacuuuccgagcugccgcggugagugugagg</td><td>gi|307603369|gb|CM001007.1|:70107299..7010</td></tr>
<tr><td>gi|307603380|gb|CM000996.1|_30401</td><td>2.5</td><td>0.81 ± 0.02</td><td></td><td>55</td><td>55</td><td>0</td><td>0</td><td>yes</td><td></td><td></td><td></td><td>blast</td><td>gguagcgucguuuccucucucg</td><td>agaggggacaagugacagcccg</td><td>gguagcgucguuuccucucucgcccgcucugagaggggacaagugacagcccg</td><td>gi|307603380|gb|CM000996.1|:104315706..104</td></tr>
<tr><td>gi|307603372|gb|CM001004.1|_4538</td><td>2.5</td><td>0.81 ± 0.02</td><td></td><td>36</td><td>36</td><td>0</td><td>0</td><td>yes</td><td></td><td></td><td></td><td>blast</td><td>aggacuaggggugucgcg</td><td>ccccaccccacuucccgc</td><td>ccccaccccacuucccgcccaagccuaggugcaggacuaggggugucgcg</td><td>gi|307603372|gb|CM001004.1|:18771562..1877</td></tr>
<tr><td>gi|307603382|gb|CM000994.1|_23821</td><td>2.5</td><td>0.81 ± 0.02</td><td></td><td>70</td><td>70</td><td>0</td><td>0</td><td>yes</td><td></td><td></td><td></td><td>blast</td><td>acaagugggcacugccaucucuc</td><td>gagucccaggcccagcaggaacaugggc</td><td>acaagugggcacugccaucucucaucucucugggaugagagucccaggcccagcaggaacaugggc</td><td>gi|307603382|gb|CM000994.1|:134411694..134</td></tr>
<tr><td>gi|307603380|gb|CM000996.1|_28320</td><td>2.5</td><td>0.81 ± 0.02</td><td></td><td>28</td><td>28</td><td>0</td><td>0</td><td>yes</td><td></td><td></td><td></td><td>blast</td><td>uucggagagccgggcuug</td><td>agucuggggcuccucucugucc</td><td>uucggagagccgggcuugugcaggaagcggguuccuccccauccucccagcacagucuggggcuccucucugucc</td><td>gi|307603380|gb|CM000996.1|:14534061..1453</td></tr>
<tr><td>gi|307603366|gb|CM001010.1|_17342</td><td>2.5</td><td>0.81 ± 0.02</td><td></td><td>30</td><td>30</td><td>0</td><td>0</td><td>yes</td><td></td><td></td><td></td><td>blast</td><td>cagcccugaacaucuccacaca</td><td>ggugagggaaagcagggaaggga</td><td>ggugagggaaagcagggaagggagaaucuuuaagauuccuccagcccugaacaucuccacaca</td><td>gi|307603366|gb|CM001010.1|:17965512..1796</td></tr>
<tr><td>gi|307603372|gb|CM001004.1|_3705</td><td>2.5</td><td>0.81 ± 0.02</td><td></td><td>599</td><td>599</td><td>0</td><td>0</td><td>yes</td><td></td><td></td><td></td><td>blast</td><td>ugcgugcagccagugugaaccac</td><td>ggucaccaggcugcaugcuua</td><td>ggucaccaggcugcaugcuuagcugcuguucucugcgugcagccagugugaaccac</td><td>gi|307603372|gb|CM001004.1|:93906544..9390</td></tr>
<tr><td>gi|307603372|gb|CM001004.1|_4398</td><td>2.5</td><td>0.81 ± 0.02</td><td></td><td>261</td><td>261</td><td>0</td><td>0</td><td>yes</td><td></td><td></td><td></td><td>blast</td><td>aggcgaaggcggcgguccgcg</td><td>cgagccgggguggggcgccgga</td><td>cgagccgggguggggcgccggagcaaaggaaacaaaaggcgaaggcgaaggcggcgguccgcg</td><td>gi|307603372|gb|CM001004.1|:3190064..31901</td></tr>
<tr><td>gi|307603375|gb|CM001001.1|_43930</td><td>2.5</td><td>0.81 ± 0.02</td><td></td><td>61</td><td>61</td><td>0</td><td>0</td><td>yes</td><td></td><td></td><td></td><td>blast</td><td>uccgaggagcuguccgcc</td><td>cggacacgcuccaggugggcggc</td><td>uccgaggagcuguccgcccagcgguucugaaauucacucugcggacacgcuccaggugggcggc</td><td>gi|307603375|gb|CM001001.1|:126958604..126</td></tr>
<tr><td>gi|307603364|gb|CM001012.1|_21305</td><td>2.5</td><td>0.81 ± 0.02</td><td></td><td>29</td><td>29</td><td>0</td><td>0</td><td>yes</td><td></td><td></td><td></td><td>blast</td><td>ucuuuucucuggcuggccu</td><td>gcuagccucuguagaguggaga</td><td>ucuuuucucuggcuggccucuguagaguggagacaggcuugugucuuuucucuggcuagccucuguagaguggaga</td><td>gi|307603364|gb|CM001012.1|:56891250..5689</td></tr>
<tr><td>gi|307603372|gb|CM001004.1|_4291</td><td>2.5</td><td>0.81 ± 0.02</td><td></td><td>108</td><td>108</td><td>0</td><td>0</td><td>yes</td><td></td><td></td><td></td><td>blast</td><td>uucauccaccagcccugccacu</td><td>aggcagggucucgugggguguugu</td><td>aggcagggucucgugggguguugugugcugucgcacggagcuucauccaccagcccugccacu</td><td>gi|307603372|gb|CM001004.1|:117592395..117</td></tr>
<tr><td>gi|307603376|gb|CM001000.1|_41568</td><td>2.5</td><td>0.81 ± 0.02</td><td></td><td>42</td><td>42</td><td>0</td><td>0</td><td>yes</td><td></td><td></td><td></td><td>blast</td><td>caucuggcccccuuccucua</td><td>gggugagggggcuuggaugcu</td><td>gggugagggggcuuggaugcugagguccgugggggacaggaaccucugccccuggcacacaucuggcccccuuccucua</td><td>gi|307603376|gb|CM001000.1|:53067669..5306</td></tr>
<tr><td>gi|307603376|gb|CM001000.1|_41528</td><td>2.5</td><td>0.81 ± 0.02</td><td></td><td>94</td><td>94</td><td>0</td><td>0</td><td>yes</td><td></td><td></td><td></td><td>blast</td><td>cccgguggucgcuccuucgcucc</td><td>cacgaaggauggcccaggggguc</td><td>cacgaaggauggcccagggggucgaacuaacagaagacccggugucgcuccuucgcucc</td><td>gi|307603376|gb|CM001000.1|:52471455..5247</td></tr>
<tr><td>gi|307603381|gb|CM000995.1|_24874</td><td>2.5</td><td>0.81 ± 0.02</td><td></td><td>205381</td><td>205381</td><td>0</td><td>0</td><td>yes</td><td></td><td></td><td></td><td>blast</td><td>aucucgcuggggccucca</td><td>ggggcccaaguguugagaa</td><td>aucucgcuggggccucccaauuguuggaccccaggggcccaaguguugagaa</td><td>gi|307603381|gb|CM000995.1|:28351452..2835</td></tr>
<tr><td>gi|307603378|gb|CM000998.1|_36389</td><td>2.5</td><td>0.81 ± 0.02</td><td></td><td>42</td><td>42</td><td>0</td><td>0</td><td>yes</td><td></td><td></td><td></td><td>blast</td><td>auggaggacgguggugagugug</td><td>cacgguccgcggccuccgcacugg</td><td>auggaggacgguggugagugugucaacagagcggcggggacacacgguccgcggccuccgcacugg</td><td>gi|307603378|gb|CM000998.1|:125028956..125</td></tr>
<tr><td>gi|307603367|gb|CM001009.1|_14899</td><td>2.5</td><td>0.81 ± 0.02</td><td></td><td>33</td><td>33</td><td>0</td><td>0</td><td>yes</td><td></td><td></td><td></td><td>blast</td><td>uucaaaauagccuguccaua</td><td>uggacaggcuauuuugaaug</td><td>uggacaggcuauuuugaaugcuaguaauggacauucaaaauagccuguccaua</td><td>gi|307603367|gb|CM001009.1|:42926983..4292</td></tr>
<tr><td>gi|307603374|gb|CM001002.1|_46233</td><td>2.5</td><td>0.81 ± 0.02</td><td></td><td>32</td><td>32</td><td>0</td><td>0</td><td>yes</td><td></td><td></td><td></td><td>blast</td><td>gccguccggguccgggaaag</td><td>aucccgggccuuggaggcac</td><td>aucccgggccuuggaggcacacauggccuggagugucaaacagucauccagacaggcuccggucgcgccguccggguccgggaaag</td><td>gi|307603374|gb|CM001002.1|:96790004..9679</td></tr>
<tr><td>gi|307603366|gb|CM001010.1|_16393</td><td>2.5</td><td>0.81 ± 0.02</td><td></td><td>212</td><td>212</td><td>0</td><td>0</td><td>yes</td><td></td><td></td><td></td><td>blast</td><td>cccggccgguuugaggggug</td><td>ccgccagggaguggggga</td><td>ccgccagggagugggggaggccgaggaggcggggggagaggggguggcguccccggccgguuugaggggug</td><td>gi|307603366|gb|CM001010.1|:28046251..2804</td></tr>
<tr><td>gi|307603382|gb|CM000994.1|_24020</td><td>2.5</td><td>0.81 ± 0.02</td><td></td><td>32</td><td>32</td><td>0</td><td>0</td><td>yes</td><td></td><td></td><td></td><td>blast</td><td>gggguuggagagcgcugc</td><td>aagcgacacuggaugucccccc</td><td>aagcgacacuggauguccccccccaccccgaucuccuaggcaaugggugggugcgggggguuggagagcgcugc</td><td>gi|307603382|gb|CM000994.1|:158134633..158</td></tr>
<tr><td>gi|307603364|gb|CM001012.1|_20447</td><td>2.5</td><td>0.81 ± 0.02</td><td></td><td>50</td><td>50</td><td>0</td><td>0</td><td>yes</td><td></td><td></td><td></td><td>blast</td><td>cugagcagcagggcugug</td><td>cgccggcugcgcaguc</td><td>cgccggcugcgcagucaaacccucuucugagcugucaguuccagccagaggcugagcagcagggcugug</td><td>gi|307603364|gb|CM001012.1|:47142964..4714</td></tr>
<tr><td>gi|307603377|gb|CM000999.1|_39291</td><td>2.5</td><td>0.81 ± 0.02</td><td></td><td>69</td><td>69</td><td>0</td><td>0</td><td>yes</td><td></td><td></td><td></td><td>blast</td><td>acgcaccagaggggcugcugc</td><td>agcggcgaggggccugc</td><td>acgcaccagaggggcugcugcuuauugcugccucccucugcagagaacaagggcagcggcgaggggccugc</td><td>gi|307603377|gb|CM000999.1|:142913054..142</td></tr>
<tr><td>gi|307603379|gb|CM000997.1|_32260</td><td>2.4</td><td>0.81 ± 0.02</td><td></td><td>276</td><td>276</td><td>0</td><td>0</td><td>yes</td><td></td><td></td><td></td><td>blast</td><td>gcucguggcguggcaccua</td><td>ggugccucuugccgcgacua</td><td>gcucguggcguggcaccuauacgcgcuucccuugcgaucgcgugcuuuuggggugccucuugccgcgacua</td><td>gi|307603379|gb|CM000997.1|:155068937..155</td></tr>
<tr><td>gi|307603378|gb|CM000998.1|_36095</td><td>2.4</td><td>0.81 ± 0.02</td><td></td><td>31</td><td>31</td><td>0</td><td>0</td><td>yes</td><td></td><td></td><td></td><td>blast</td><td>ucagggccggagcggacuagca</td><td>cuauuggcuccgcagcaccuggcc</td><td>ucagggccggagcggacuagcagagcuauuggcuccgcagcaccuggcc</td><td>gi|307603378|gb|CM000998.1|:109089551..109</td></tr>
<tr><td>gi|307603376|gb|CM001000.1|_41294</td><td>2.4</td><td>0.81 ± 0.02</td><td></td><td>29</td><td>29</td><td>0</td><td>0</td><td>yes</td><td></td><td></td><td></td><td>blast</td><td>gccgccggagccagugag</td><td>cgccccggcucccggugc</td><td>cgccccggcucccggugcccgucuccagcgccgccggagccagugag</td><td>gi|307603376|gb|CM001000.1|:29954536..2995</td></tr>
<tr><td>gi|307603375|gb|CM001001.1|_43002</td><td>2.4</td><td>0.81 ± 0.02</td><td></td><td>34</td><td>34</td><td>0</td><td>0</td><td>yes</td><td></td><td></td><td></td><td>blast</td><td>gcugaggcucggcacugugaga</td><td>uggugaaggugcagccucaguug</td><td>gcugaggcucggcacugugagaggccauggaaggcuacugguggaaggugcagccucaguug</td><td>gi|307603375|gb|CM001001.1|:25583058..2558</td></tr>
<tr><td>gi|307603376|gb|CM001000.1|_39793</td><td>2.4</td><td>0.81 ± 0.02</td><td></td><td>359</td><td>344</td><td>15</td><td>0</td><td>yes</td><td></td><td></td><td></td><td>blast</td><td>aggcaugggauguacggg</td><td>uuguucugguuucg</td><td>uuguucugguuucgccccuuuagugcucgccucacuaaaugcacagagcgggugaggcaugggauguacggg</td><td>gi|307603376|gb|CM001000.1|:31158814..3115</td></tr>
<tr><td>gi|307603364|gb|CM001012.1|_21242</td><td>2.4</td><td>0.81 ± 0.02</td><td></td><td>160</td><td>160</td><td>0</td><td>0</td><td>yes</td><td></td><td></td><td></td><td>blast</td><td>cucucugcacgagcaucga</td><td>ggugcuuggaagaggggu</td><td>ggugcuuggaagaggggguucuucuuaaagggccucucugcacgagcaucga</td><td>gi|307603364|gb|CM001012.1|:47126139..4712</td></tr>
<tr><td>gi|307603374|gb|CM001002.1|_47889</td><td>2.4</td><td>0.81 ± 0.02</td><td></td><td>30</td><td>30</td><td>0</td><td>0</td><td>yes</td><td></td><td></td><td></td><td>blast</td><td>gagcaaggacucugugguca</td><td>ggagcagggggccauggccag</td><td>gagcaaggacucugugguccacagcccagugggagcagggggccauggccag</td><td>gi|307603374|gb|CM001002.1|:109000481..109</td></tr>
<tr><td>gi|307603378|gb|CM000998.1|_36648</td><td>2.4</td><td>0.81 ± 0.02</td><td></td><td>32</td><td>32</td><td>0</td><td>0</td><td>yes</td><td></td><td></td><td></td><td>blast</td><td>aggacaagcggcagugcccucu</td><td>aggacacugcccacuuuccucu</td><td>aggacacugcccacuuuccucucgguuucauucucacccaggacaagcggcagugcccucu</td><td>gi|307603378|gb|CM000998.1|:141098503..141</td></tr>
<tr><td>gi|307603376|gb|CM001000.1|_42788</td><td>2.4</td><td>0.81 ± 0.02</td><td></td><td>35</td><td>35</td><td>0</td><td>0</td><td>yes</td><td></td><td></td><td></td><td>blast</td><td>cucgcagagcacgacgcgg</td><td>cauucgugcgcugcagcg</td><td>cauucgugcgcugcagcggcugcucgcagagcacgacgcgg</td><td>gi|307603376|gb|CM001000.1|:150154076..150</td></tr>
<tr><td>gi|307603379|gb|CM000997.1|_33389</td><td>2.4</td><td>0.81 ± 0.02</td><td></td><td>70</td><td>70</td><td>0</td><td>0</td><td>yes</td><td></td><td></td><td></td><td>blast</td><td>cgugguaggacugugagcc</td><td>cucugugugguggcuugcuggug</td><td>cgugguaggacugugagcccagcgccuggcuuggcaggucgggcuuucugcucuguguggcuugcuggug</td><td>gi|307603379|gb|CM000997.1|:131830139..131</td></tr>
<tr><td>gi|307603366|gb|CM001010.1|_17710</td><td>2.4</td><td>0.81 ± 0.02</td><td></td><td>114</td><td>114</td><td>0</td><td>0</td><td>yes</td><td></td><td></td><td></td><td>blast</td><td>cugccucucugaccucuucac</td><td>gaggaggcugggauggccgcc</td><td>gaggaggcugggauggccgccacuggcagagcuagcagagagcuuaccgugcugccucucugaccucuucac</td><td>gi|307603366|gb|CM001010.1|:35253374..3525</td></tr>
<tr><td>gi|307603381|gb|CM000995.1|_27716</td><td>2.4</td><td>0.81 ± 0.02</td><td></td><td>29</td><td>29</td><td>0</td><td>0</td><td>yes</td><td></td><td></td><td></td><td>blast</td><td>accgagcucacccaccug</td><td>ugcugggguuuggcgg</td><td>ugcugggguuuggcggccuuaggggcugcauugcagcuccgcgagcccaccgagcucacccaccug</td><td>gi|307603381|gb|CM000995.1|:130100456..130</td></tr>
<tr><td>gi|307603369|gb|CM001007.1|_10820</td><td>2.4</td><td>0.81 ± 0.02</td><td></td><td>38</td><td>38</td><td>0</td><td>0</td><td>yes</td><td></td><td></td><td></td><td>blast</td><td>ggagccuacugggaacgc</td><td>gacgcccagucuacgcggccgc</td><td>ggagccuacugggaacgcgugcaaaauggcgacgcccagucuacgcggccgc</td><td>gi|307603369|gb|CM001007.1|:21522269..2152</td></tr>
<tr><td>gi|307603378|gb|CM000998.1|_35183</td><td>2.4</td><td>0.81 ± 0.02</td><td></td><td>101</td><td>101</td><td>0</td><td>0</td><td>yes</td><td></td><td></td><td></td><td>blast</td><td>augcugcuagggaagcuguug</td><td>ucagcuucgguggcgcgg</td><td>augcugcuagggaagcuguugcucagcuucgguggcgcgg</td><td>gi|307603378|gb|CM000998.1|:139064116..139</td></tr>
<tr><td>gi|307603382|gb|CM000994.1|_21893</td><td>2.4</td><td>0.81 ± 0.02</td><td></td><td>40</td><td>40</td><td>0</td><td>0</td><td>yes</td><td></td><td></td><td></td><td>blast</td><td>ugagucggagaccggggccuccu</td><td>cccgcuuccgguucgccaccucgcc</td><td>cccgcuuccgguucgccaccucgccuuucucuggugagucggagaccggggccuccu</td><td>gi|307603382|gb|CM000994.1|:74708548..7470</td></tr>
<tr><td>gi|307603371|gb|CM001005.1|_8368</td><td>2.4</td><td>0.81 ± 0.02</td><td></td><td>1566</td><td>1566</td><td>0</td><td>0</td><td>yes</td><td></td><td></td><td></td><td>blast</td><td>uugaacugucaagaaccacugg</td><td>aguggucuaaacauuucacaa</td><td>aguggucuaaacauuucacaauugugcuacagaacuguugaacugucaagaaccacugg</td><td>gi|307603371|gb|CM001005.1|:113369097..113</td></tr>
<tr><td>gi|307603376|gb|CM001000.1|_41511</td><td>2.4</td><td>0.81 ± 0.02</td><td></td><td>54</td><td>29</td><td>0</td><td>25</td><td>yes</td><td></td><td></td><td></td><td>blast</td><td>aucccccaggccuguaccagg</td><td>acugguacaaggguugggagac</td><td>aucccccaggccuguaccaggucugaggcacagcacugguacaaggguugggagac</td><td>gi|307603376|gb|CM001000.1|:52377130..5237</td></tr>
<tr><td>gi|307603373|gb|CM001003.1|_1882</td><td>2.4</td><td>0.81 ± 0.02</td><td></td><td>128</td><td>128</td><td>0</td><td>0</td><td>yes</td><td></td><td></td><td></td><td>blast</td><td>uagcucucccuggccugcacagg</td><td>ugaggcagggcuaggagcuggcuggu</td><td>ugaggcagggcuaggagcuggcugcuguggaggugaccuagcucucccuggccugcacagg</td><td>gi|307603373|gb|CM001003.1|:80132146..8013</td></tr>
<tr><td>gi|307603376|gb|CM001000.1|_42392</td><td>2.4</td><td>0.81 ± 0.02</td><td></td><td>32</td><td>32</td><td>0</td><td>0</td><td>yes</td><td></td><td></td><td></td><td>blast</td><td>cggcguguagagggagcu</td><td>cccucggaggcuacgcuuuc</td><td>cccucggaggcuacgcuuucuucggcguguagagggagcu</td><td>gi|307603376|gb|CM001000.1|:123236639..123</td></tr>
<tr><td>gi|307603380|gb|CM000996.1|_29551</td><td>2.4</td><td>0.81 ± 0.02</td><td></td><td>31</td><td>31</td><td>0</td><td>0</td><td>yes</td><td></td><td></td><td></td><td>blast</td><td>uggugcccaugccuccuaguca</td><td>gguaguggggauggugcccaug</td><td>gguaguggggauggugcccaugccuccuagucaccugaccucugugugguaguggggauggugcccaugccuccuaguca</td><td>gi|307603380|gb|CM000996.1|:152388986..152</td></tr>
<tr><td>gi|307603364|gb|CM001012.1|_19891</td><td>2.4</td><td>0.81 ± 0.02</td><td></td><td>31</td><td>31</td><td>0</td><td>0</td><td>yes</td><td></td><td></td><td></td><td>blast</td><td>ugcagcgguggcaucgagaggagc</td><td>ucuucucugcccucugcuacuuc</td><td>ucuucucugcccucugcuacuucugcugaaggaggaggcuccauguuguccccucagcgaacugcagcgguggcaucgagaggagc</td><td>gi|307603364|gb|CM001012.1|:7569064..75691</td></tr>
<tr><td>gi|307603364|gb|CM001012.1|_20183</td><td>2.4</td><td>0.81 ± 0.02</td><td></td><td>134</td><td>134</td><td>0</td><td>0</td><td>yes</td><td></td><td></td><td></td><td>blast</td><td>gcugcgcgagcgggucugggcg</td><td>cccagacgcgcaccaucgaggacguuu</td><td>cccagacgcgcaccaucgaggacguuucgcaaagccaccaucgaggagcugcgcgagcgggucugggcg</td><td>gi|307603364|gb|CM001012.1|:30105299..3010</td></tr>
<tr><td>gi|307603363|gb|CM001013.1|_49783</td><td>2.4</td><td>0.81 ± 0.02</td><td></td><td>114</td><td>94</td><td>20</td><td>0</td><td>yes</td><td></td><td></td><td></td><td>blast</td><td>gcugcugccgucaccucaug</td><td>cgaggaagaggcggcgguacug</td><td>gcugcugccgucaccucauggcgacucggcuugaggagguaacgcgaggaagaggcggcgguacug</td><td>gi|307603363|gb|CM001013.1|:159267223..159</td></tr>
<tr><td>gi|307603376|gb|CM001000.1|_39441</td><td>2.4</td><td>0.81 ± 0.02</td><td></td><td>42</td><td>42</td><td>0</td><td>0</td><td>yes</td><td></td><td></td><td></td><td>blast</td><td>gcuagcgugucggggccu</td><td>gcgccugauacgcaggcgc</td><td>gcgccugauacgcaggcgcauuuguugcuagcgugucggggccu</td><td>gi|307603376|gb|CM001000.1|:4966879..49669</td></tr>
<tr><td>gi|307603375|gb|CM001001.1|_44920</td><td>2.4</td><td>0.81 ± 0.02</td><td></td><td>124</td><td>124</td><td>0</td><td>0</td><td>yes</td><td></td><td></td><td></td><td>blast</td><td>ucggcgaggcccggaggaca</td><td>uccuccggccacgcccagucc</td><td>uccuccggccacgcccaguccguccgcaggauggucggcgaggcccggaggaca</td><td>gi|307603375|gb|CM001001.1|:108939395..108</td></tr>
<tr><td>gi|307603376|gb|CM001000.1|_39553</td><td>2.4</td><td>0.81 ± 0.02</td><td></td><td>29</td><td>29</td><td>0</td><td>0</td><td>yes</td><td></td><td></td><td></td><td>blast</td><td>uugaaaggccacgcugag</td><td>gggcggaggccuuuaagca</td><td>gggcggaggccuuuaagcaccucgccauauugaaaggccacgcugag</td><td>gi|307603376|gb|CM001000.1|:17579321..1757</td></tr>
<tr><td>gi|307603372|gb|CM001004.1|_4485</td><td>2.4</td><td>0.81 ± 0.02</td><td></td><td>235</td><td>235</td><td>0</td><td>0</td><td>yes</td><td></td><td></td><td></td><td>blast</td><td>ccuguggacgguccugca</td><td>caggagcucuucccuacagaguc</td><td>ccuguggacgguccugcacagcucugccagcagggccgcaggagcucuucccuacagaguc</td><td>gi|307603372|gb|CM001004.1|:6462411..64624</td></tr>
<tr><td>gi|307603375|gb|CM001001.1|_44585</td><td>2.4</td><td>0.81 ± 0.02</td><td></td><td>33</td><td>33</td><td>0</td><td>0</td><td>yes</td><td></td><td></td><td></td><td>blast</td><td>uagugagccugugauagugga</td><td>caccucagcagggcucagag</td><td>uagugagccugugauaguggacacuuuugugugaaagugccaccucagcagggcucagag</td><td>gi|307603375|gb|CM001001.1|:80025078..8002</td></tr>
<tr><td>gi|307603364|gb|CM001012.1|_21283</td><td>2.4</td><td>0.81 ± 0.02</td><td></td><td>352</td><td>352</td><td>0</td><td>0</td><td>yes</td><td></td><td></td><td></td><td>blast</td><td>cuugcuggugcagggcugcucugg</td><td>ccccgagcucugcacgacaguaaagg</td><td>cuugcuggugcagggcugcucugggagaagcucucccaaacccagcggcuucgaccccgagcucugcacgacaguaaagg</td><td>gi|307603364|gb|CM001012.1|:53675711..5367</td></tr>
<tr><td>gi|307603374|gb|CM001002.1|_46520</td><td>2.4</td><td>0.81 ± 0.02</td><td></td><td>39</td><td>39</td><td>0</td><td>0</td><td>yes</td><td></td><td></td><td></td><td>blast</td><td>aaguuggagcaagaaggugcgug</td><td>cacacccagcuuuggcugu</td><td>aaguuggagcaagaaggugcgugcgggugccgaaaugcucagacccuccgaagcugaugggcaccugcucacacccagcuuuggcugu</td><td>gi|307603374|gb|CM001002.1|:118834702..118</td></tr>
<tr><td>gi|307603375|gb|CM001001.1|_44897</td><td>2.4</td><td>0.81 ± 0.02</td><td></td><td>184</td><td>184</td><td>0</td><td>0</td><td>yes</td><td></td><td></td><td></td><td>blast</td><td>uguaguuuuggcuauccuggua</td><td>ccaggccggccacaaacucaca</td><td>uguaguuuuggcuauccugguacuugcucuguagaccaggccggccacaaacucaca</td><td>gi|307603375|gb|CM001001.1|:108234969..108</td></tr>
<tr><td>gi|307603376|gb|CM001000.1|_40748</td><td>2.4</td><td>0.81 ± 0.02</td><td></td><td>63</td><td>63</td><td>0</td><td>0</td><td>yes</td><td></td><td></td><td></td><td>blast</td><td>uccgcggaaaagagccag</td><td>ggcucgugcgcggaau</td><td>uccgcggaaaagagccagggguuggcucgugcgcggaau</td><td>gi|307603376|gb|CM001000.1|:138506391..138</td></tr>
<tr><td>gi|307603372|gb|CM001004.1|_5560</td><td>2.4</td><td>0.81 ± 0.02</td><td></td><td>29</td><td>29</td><td>0</td><td>0</td><td>yes</td><td></td><td></td><td></td><td>blast</td><td>ucggaguguaucacagaaccua</td><td>aguucugucaugcacugacugcuc</td><td>aguucugucaugcacugacugcuccagagcccgagucggaguguaucacagaaccua</td><td>gi|307603372|gb|CM001004.1|:96711713..9671</td></tr>
<tr><td>gi|307603372|gb|CM001004.1|_5880</td><td>2.4</td><td>0.81 ± 0.02</td><td></td><td>233</td><td>233</td><td>0</td><td>0</td><td>yes</td><td></td><td></td><td></td><td>blast</td><td>cacaucccggaucuggaguau</td><td>ccuccggaggccgggagaggcc</td><td>ccuccggaggccgggagaggccugcgggcucgcggcacaucccggaucuggaguau</td><td>gi|307603372|gb|CM001004.1|:106134216..106</td></tr>
<tr><td>gi|307603370|gb|CM001006.1|_9802</td><td>2.4</td><td>0.81 ± 0.02</td><td></td><td>41</td><td>41</td><td>0</td><td>0</td><td>yes</td><td></td><td></td><td></td><td>blast</td><td>ugggcgagauaugugacaucacu</td><td>ugaugucacuuuucccgccgucc</td><td>ugaugucacuuuucccgccguccggcuuucggggcggggcugaugggcgagauaugugacaucacu</td><td>gi|307603370|gb|CM001006.1|:23853435..2385</td></tr>
<tr><td>gi|307603371|gb|CM001005.1|_6217</td><td>2.4</td><td>0.81 ± 0.02</td><td></td><td>31</td><td>31</td><td>0</td><td>0</td><td>yes</td><td></td><td></td><td></td><td>blast</td><td>cugcugugcacuguggug</td><td>cugcagaugcgcugcaccc</td><td>cugcagaugcgcugcacccgggugcugcugcuuaguaugugcagaccagcacaggcugcugugcacuguggug</td><td>gi|307603371|gb|CM001005.1|:3780506..37805</td></tr>
<tr><td>gi|307603378|gb|CM000998.1|_36135</td><td>2.4</td><td>0.81 ± 0.02</td><td></td><td>72</td><td>72</td><td>0</td><td>0</td><td>yes</td><td></td><td></td><td></td><td>blast</td><td>ucuagaagagcccgguggauccuu</td><td>ggaggccguguucuucuucuag</td><td>ucuagaagagcccgguggauccuucagcaagaggaggccguguucuucuucuag</td><td>gi|307603378|gb|CM000998.1|:111676100..111</td></tr>
<tr><td>gi|307603382|gb|CM000994.1|_22447</td><td>2.4</td><td>0.81 ± 0.02</td><td></td><td>121</td><td>121</td><td>0</td><td>0</td><td>yes</td><td></td><td></td><td></td><td>blast</td><td>cuugcaggguucuggaugga</td><td>uuuccagcugcgcugcccacc</td><td>uuuccagcugcgcugcccaccgccaggcagguaagaugagaaugccuguuucuugcaggucuggaugga</td><td>gi|307603382|gb|CM000994.1|:155500455..155</td></tr>
<tr><td>gi|307603380|gb|CM000996.1|_29571</td><td>2.4</td><td>0.81 ± 0.02</td><td></td><td>32</td><td>32</td><td>0</td><td>0</td><td>yes</td><td></td><td></td><td></td><td>blast</td><td>aggcucggcucugugggaugc</td><td>cucccagggaggccaggccccu</td><td>cucccagggaggccaggccccugaaccagagagaggcucggcucugugggaugc</td><td>gi|307603380|gb|CM000996.1|:154756673..154</td></tr>
<tr><td>gi|307603376|gb|CM001000.1|_41179</td><td>2.4</td><td>0.81 ± 0.02</td><td></td><td>45</td><td>45</td><td>0</td><td>0</td><td>yes</td><td></td><td></td><td></td><td>blast</td><td>ccccccgauggcuggcuu</td><td>gccggcccggcuaggggag</td><td>ccccccgauggcuggcuuucuuuuucuccacccagucccucacccccagugcccccccaggccggcccggcuaggggag</td><td>gi|307603376|gb|CM001000.1|:25857452..2585</td></tr>
<tr><td>gi|307603375|gb|CM001001.1|_43533</td><td>2.4</td><td>0.81 ± 0.02</td><td></td><td>34</td><td>34</td><td>0</td><td>0</td><td>yes</td><td></td><td></td><td></td><td>blast</td><td>cucugggcugagcugugga</td><td>ugggcugagcuguggaggg</td><td>cucugggcugagcuguggagggugcuggcagacucugggcugagcuguggagggugcuggcagacucugggcugagcuguggaggg</td><td>gi|307603375|gb|CM001001.1|:96482845..9648</td></tr>
<tr><td>gi|307603366|gb|CM001010.1|_16455</td><td>2.4</td><td>0.81 ± 0.02</td><td></td><td>42</td><td>42</td><td>0</td><td>0</td><td>yes</td><td></td><td></td><td></td><td>blast</td><td>gugugcugccuguggccu</td><td>ccugugggcucacacca</td><td>ccugugggcucacaccauagucucugugugugcugccuguggggcucacaccauagucucugugugugcugccuguggccu</td><td>gi|307603366|gb|CM001010.1|:30277240..3027</td></tr>
<tr><td>gi|307603376|gb|CM001000.1|_42197</td><td>2.4</td><td>0.81 ± 0.02</td><td></td><td>68</td><td>68</td><td>0</td><td>0</td><td>yes</td><td></td><td></td><td></td><td>blast</td><td>uggccuuugugucugcggauag</td><td>auccgcagacuucaaggcccagc</td><td>uggccuuugugucugcggauagaagcaagcggaaaauccgcagacuucaaggcccagc</td><td>gi|307603376|gb|CM001000.1|:106683702..106</td></tr>
<tr><td>gi|307603381|gb|CM000995.1|_26411</td><td>2.4</td><td>0.81 ± 0.02</td><td></td><td>812</td><td>346</td><td>466</td><td>0</td><td>yes</td><td></td><td></td><td></td><td>blast</td><td>gcugguccggcuguuggagc</td><td>gccaauggcugcgagcgc</td><td>gccaauggcugcgagcgccagaggccggggagcaggcuaggcgcugggugcuggucggcuguuggagc</td><td>gi|307603381|gb|CM000995.1|:179711567..179</td></tr>
<tr><td>gi|307603376|gb|CM001000.1|_42166</td><td>2.4</td><td>0.81 ± 0.02</td><td></td><td>29</td><td>29</td><td>0</td><td>0</td><td>yes</td><td></td><td></td><td></td><td>blast</td><td>ucucuccccgaaccgcccgaga</td><td>ucgggccggggaggcugagc</td><td>ucgggccggggaggcugagcgaggaaggagguccgcucucuccccgaaccgcccgaga</td><td>gi|307603376|gb|CM001000.1|:105987380..105</td></tr>
<tr><td>gi|307603381|gb|CM000995.1|_28071</td><td>2.4</td><td>0.81 ± 0.02</td><td></td><td>183</td><td>183</td><td>0</td><td>0</td><td>yes</td><td></td><td></td><td></td><td>blast</td><td>acaguucaggacugccuguggca</td><td>ccagagcgguucugggaaagcc</td><td>acaguucaggacugccuguggcagagagcagagcaagccagcuugcuucuccagagcgguucugggaaaggc</td><td>gi|307603381|gb|CM000995.1|:165709574..165</td></tr>
<tr><td>gi|307603368|gb|CM001008.1|_14295</td><td>2.4</td><td>0.81 ± 0.02</td><td></td><td>59</td><td>59</td><td>0</td><td>0</td><td>yes</td><td></td><td></td><td></td><td>blast</td><td>uggaacccggggccgccccgu</td><td>gcaaaggcuccgccuucugcc</td><td>uggaacccggggccgccccguucggaaauuucccucauuauucccgcuuccacccucccgagcaaaggcuccgccuucugcc</td><td>gi|307603368|gb|CM001008.1|:99304971..9930</td></tr>
<tr><td>gi|307603381|gb|CM000995.1|_26753</td><td>2.4</td><td>0.81 ± 0.02</td><td></td><td>31</td><td>31</td><td>0</td><td>0</td><td>yes</td><td></td><td></td><td></td><td>blast</td><td>cuccccaacaaagaaagc</td><td>uugcacuggggggggggggg</td><td>cuccccaacaaagaaagcagcagccacacugcagcuugcacuggggggggggggg</td><td>gi|307603381|gb|CM000995.1|:28553961..2855</td></tr>
<tr><td>gi|307603380|gb|CM000996.1|_29200</td><td>2.4</td><td>0.81 ± 0.02</td><td></td><td>122</td><td>122</td><td>0</td><td>0</td><td>yes</td><td></td><td></td><td></td><td>blast</td><td>augugugugugugauguguguauuu</td><td>augaugcugggcaauggaugu</td><td>augugugugugugauguguguauuuauauaugugugugugcacacgaaugugugugcgcacgcgcguguguaaaugaugcugggcaauggaugu</td><td>gi|307603380|gb|CM000996.1|:104036268..104</td></tr>
<tr><td>gi|307603372|gb|CM001004.1|_4778</td><td>2.4</td><td>0.81 ± 0.02</td><td></td><td>51</td><td>51</td><td>0</td><td>0</td><td>yes</td><td></td><td></td><td></td><td>blast</td><td>acacacugaccugaucgacucgcu</td><td>uggucuguccaguguggcu</td><td>uggucuguccaguguggcuggacacacugaccugaucgacucgcu</td><td>gi|307603372|gb|CM001004.1|:50071414..5007</td></tr>
<tr><td>gi|307603379|gb|CM000997.1|_32273</td><td>2.4</td><td>0.81 ± 0.02</td><td></td><td>141</td><td>141</td><td>0</td><td>0</td><td>yes</td><td></td><td></td><td></td><td>blast</td><td>uucaaaccucucuggcugccc</td><td>caggccaaggugggauuuuugaggg</td><td>caggccaaggugggauuuuugaggggguuagacagagccuucaaaccucucuggcugccc</td><td>gi|307603379|gb|CM000997.1|:155232913..155</td></tr>
<tr><td>gi|307603382|gb|CM000994.1|_23125</td><td>2.4</td><td>0.81 ± 0.02</td><td></td><td>47</td><td>47</td><td>0</td><td>0</td><td>yes</td><td></td><td></td><td></td><td>blast</td><td>auggugagcuuccugagcgcag</td><td>cugcucaguggcuucacugcga</td><td>auggugagcuuccugagcgcagggugcacucucugcucaguggcuucacugcga</td><td>gi|307603382|gb|CM000994.1|:39460931..3946</td></tr>
<tr><td>gi|307603365|gb|CM001011.1|_18430</td><td>2.3</td><td>0.81 ± 0.02</td><td></td><td>320</td><td>320</td><td>0</td><td>0</td><td>yes</td><td></td><td></td><td></td><td>blast</td><td>cgcugagauuccggguucccg</td><td>gcucucggucucuucuuuucgcc</td><td>cgcugagauuccggguucccgcagccgcgggcagcgggugccuguuagcuaccguggcucucggucucuucuuuucgcc</td><td>gi|307603365|gb|CM001011.1|:25912890..2591</td></tr>
<tr><td>gi|307603373|gb|CM001003.1|_543</td><td>2.3</td><td>0.81 ± 0.02</td><td></td><td>29</td><td>29</td><td>0</td><td>0</td><td>yes</td><td></td><td></td><td></td><td>blast</td><td>uugagcgcucucggaagggg</td><td>ccucacuuccagcgaugaug</td><td>ccucacuuccagcgaugaugcucuggacgcccccucccucuucagagucagagggcgugccaagcauugagcgcucucggaagggg</td><td>gi|307603373|gb|CM001003.1|:74708583..7470</td></tr>
<tr><td>gi|307603372|gb|CM001004.1|_6165</td><td>2.3</td><td>0.81 ± 0.02</td><td></td><td>34</td><td>34</td><td>0</td><td>0</td><td>yes</td><td></td><td></td><td></td><td>blast</td><td>cucgauccggguggugaa</td><td>cgggccgggauccggggu c</td><td>cucgauccggguggugaaggucgggccgggauccggggguc</td><td>gi|307603372|gb|CM001004.1|:120494156..120</td></tr>
<tr><td>gi|307603374|gb|CM001002.1|_46740</td><td>2.3</td><td>0.81 ± 0.02</td><td></td><td>177</td><td>177</td><td>0</td><td>0</td><td>yes</td><td></td><td></td><td></td><td>blast</td><td>ucacgugagcacguuggcaccc</td><td>acguuggcacugcucacgugagc</td><td>acguuggcacugcucacgugagcacguuggcaccgcucacgugagcacguuggcaccc</td><td>gi|307603374|gb|CM001002.1|:15782964..1578</td></tr>
<tr><td>gi|307603371|gb|CM001005.1|_7427</td><td>2.3</td><td>0.81 ± 0.02</td><td></td><td>29</td><td>29</td><td>0</td><td>0</td><td>yes</td><td></td><td></td><td></td><td>blast</td><td>cggccggagcugcugcaga</td><td>ugccuuguggcuccuacugag</td><td>ugccuuguggcuccuacugaguccacggccggagcugcugcaga</td><td>gi|307603371|gb|CM001005.1|:113983628..113</td></tr>
<tr><td>gi|307603363|gb|CM001013.1|_48543</td><td>2.3</td><td>0.81 ± 0.02</td><td></td><td>38</td><td>38</td><td>0</td><td>0</td><td>yes</td><td></td><td></td><td></td><td>blast</td><td>agggcauugugagggcug</td><td>ggaagaaaggagggcccuua</td><td>ggaagaaaggagggcccuuacccaggucagccacccaccugggugagggcauugugagggcug</td><td>gi|307603363|gb|CM001013.1|:96411906..9641</td></tr>
<tr><td>gi|307603366|gb|CM001010.1|_16821</td><td>2.3</td><td>0.81 ± 0.02</td><td></td><td>33</td><td>33</td><td>0</td><td>0</td><td>yes</td><td></td><td></td><td></td><td>blast</td><td>agcugucgugguagcgucugc</td><td>ccacgcugccgccacccccucc</td><td>ccacgcugccgccacccccucccagggccugucagccccuggagcugucgugguagcgucugc</td><td>gi|307603366|gb|CM001010.1|:48015769..4801</td></tr>
<tr><td>gi|307603381|gb|CM000995.1|_28184</td><td>2.3</td><td>0.81 ± 0.02</td><td></td><td>975</td><td>975</td><td>0</td><td>0</td><td>yes</td><td></td><td></td><td></td><td>blast</td><td>ucaagguccgcgugaacacgc</td><td>gcauucaccgcgugccuuaauu</td><td>gcauucaccgcgugccuuaauuguauagacauuaaaucaagguccgcgugaacacgc</td><td>gi|307603381|gb|CM000995.1|:180628748..180</td></tr>
<tr><td>gi|307603382|gb|CM000994.1|_24479</td><td>2.3</td><td>0.81 ± 0.02</td><td></td><td>36</td><td>36</td><td>0</td><td>0</td><td>yes</td><td></td><td></td><td></td><td>blast</td><td>uacuagggcgcugagauaccg</td><td>guauugagaagcgccgugcu</td><td>uacuagggcgcugagauaccgagaugcugaggugaaugaggcucugagguauugagaagcgccgugcu</td><td>gi|307603382|gb|CM000994.1|:194861280..194</td></tr>
<tr><td>gi|307603381|gb|CM000995.1|_27300</td><td>2.3</td><td>0.81 ± 0.02</td><td></td><td>198</td><td>198</td><td>0</td><td>0</td><td>yes</td><td></td><td></td><td></td><td>blast</td><td>ugcugcugcucugggccucucgca</td><td>ggaagaggcuggagcugccgcugc</td><td>ggaagaggcuggagcugccgcugcuucugcugcugcucugggccucucgca</td><td>gi|307603381|gb|CM000995.1|:92024346..9202</td></tr>
<tr><td>gi|307603380|gb|CM000996.1|_28953</td><td>2.3</td><td>0.81 ± 0.02</td><td></td><td>38</td><td>38</td><td>0</td><td>0</td><td>yes</td><td></td><td></td><td></td><td>blast</td><td>ccugaagccugucgcucca</td><td>ggagugucucaagcuucauuag</td><td>ggagugucucaagcuucauuagacaagcuguccucaccugaagccugucgcucca</td><td>gi|307603380|gb|CM000996.1|:94974184..9497</td></tr>
<tr><td>gi|307603372|gb|CM001004.1|_4281</td><td>2.3</td><td>0.81 ± 0.02</td><td></td><td>164</td><td>164</td><td>0</td><td>0</td><td>yes</td><td></td><td></td><td></td><td>blast</td><td>aucggacccuuguauucucuggc</td><td>uagagaccacaggggugcacugugg</td><td>uagagaccacaggggugcacugugguggauaccaccaucggacccuuguauucucuggc</td><td>gi|307603372|gb|CM001004.1|:117194259..117</td></tr>
<tr><td>gi|307603381|gb|CM000995.1|_25763</td><td>2.3</td><td>0.81 ± 0.02</td><td></td><td>455</td><td>455</td><td>0</td><td>0</td><td>yes</td><td></td><td></td><td></td><td>blast</td><td>uugccugggacucuggaaccgu</td><td>uguauccagcguccuaggugagg</td><td>uguauccagcguccuaggugagggaacugucuccuucuugccugggacucuggaaccgu</td><td>gi|307603381|gb|CM000995.1|:125685017..125</td></tr>
<tr><td>gi|307603370|gb|CM001006.1|_10106</td><td>2.3</td><td>0.81 ± 0.02</td><td></td><td>38</td><td>38</td><td>0</td><td>0</td><td>yes</td><td></td><td></td><td></td><td>blast</td><td>cgcuugguucccugugugc</td><td>cuaucugaagccaagcuug</td><td>cuaucugaagccaagcuugggcagugggcaggugggccugccaguugaccugcgcuugguucccugugugc</td><td>gi|307603370|gb|CM001006.1|:54784064..5478</td></tr>
<tr><td>gi|307603363|gb|CM001013.1|_48565</td><td>2.3</td><td>0.81 ± 0.02</td><td></td><td>161</td><td>161</td><td>0</td><td>0</td><td>yes</td><td></td><td></td><td></td><td>blast</td><td>acgggcugggcaagauggcgg</td><td>gccccuucccuccccaccu</td><td>gccccuucccuccccaccucgccuggcccugcaggcgccucaggcgguacgggcugggcaagauggcgg</td><td>gi|307603363|gb|CM001013.1|:98469537..9846</td></tr>
<tr><td>gi|307603382|gb|CM000994.1|_21353</td><td>2.3</td><td>0.81 ± 0.02</td><td></td><td>33</td><td>33</td><td>0</td><td>0</td><td>yes</td><td></td><td></td><td></td><td>blast</td><td>uggucguggagcuguccu</td><td>ugcagacccacugcugaccacc</td><td>ugcagacccacugcugaccaccgucuugcaaucugcuccaggcugcuggcagcuggagguggugucguggagcuguccu</td><td>gi|307603382|gb|CM000994.1|:3660835..36609</td></tr>
<tr><td>gi|307603375|gb|CM001001.1|_44095</td><td>2.3</td><td>0.81 ± 0.02</td><td></td><td>34</td><td>34</td><td>0</td><td>0</td><td>yes</td><td></td><td></td><td></td><td>blast</td><td>auccgaaaggguaccugggcg</td><td>uccagagaccuucggagcc</td><td>auccgaaaggguaccugggcgaggcuaagcccggauacgguccacguccagagaccuucggagcc</td><td>gi|307603375|gb|CM001001.1|:13870030..1387</td></tr>
<tr><td>gi|307603378|gb|CM000998.1|_36495</td><td>2.3</td><td>0.81 ± 0.02</td><td></td><td>30</td><td>30</td><td>0</td><td>0</td><td>yes</td><td></td><td></td><td></td><td>blast</td><td>uacggccggcaagaauggaugu</td><td>auccaaauugcugguggauaug</td><td>uacggccggcaagaauggauguaaaaauaucuugcugcuuacauccaaauugcugguggauaug</td><td>gi|307603378|gb|CM000998.1|:135421731..135</td></tr>
<tr><td>gi|307603369|gb|CM001007.1|_11141</td><td>2.3</td><td>0.81 ± 0.02</td><td></td><td>33</td><td>33</td><td>0</td><td>0</td><td>yes</td><td></td><td></td><td></td><td>blast</td><td>accuaccggguuuagagaau</td><td>acucaacaggaggugg</td><td>acucaacaggagguggggugguguuguugauugacaggcauauuucaccuaccggguuuagagaau</td><td>gi|307603369|gb|CM001007.1|:55512763..5551</td></tr>
<tr><td>gi|307603366|gb|CM001010.1|_16772</td><td>2.3</td><td>0.81 ± 0.02</td><td></td><td>673</td><td>673</td><td>0</td><td>0</td><td>yes</td><td></td><td></td><td></td><td>blast</td><td>gaguucgaggccagccuggg</td><td>caggacggccagggcuauacag</td><td>gaguucgaggccagccugggcuacaaaguuaguuccaggacggccagggcuauacag</td><td>gi|307603366|gb|CM001010.1|:46497084..4649</td></tr>
<tr><td>gi|307603378|gb|CM000998.1|_36544</td><td>2.3</td><td>0.81 ± 0.02</td><td></td><td>33</td><td>33</td><td>0</td><td>0</td><td>yes</td><td></td><td></td><td></td><td>blast</td><td>gcugguuggaggggcgag</td><td>cccuuuccccucauca</td><td>cccuuuccccucaucagacccuggcugguuggaggggcgag</td><td>gi|307603378|gb|CM000998.1|:136720120..136</td></tr>
<tr><td>gi|307603369|gb|CM001007.1|_12160</td><td>2.3</td><td>0.81 ± 0.02</td><td></td><td>265</td><td>265</td><td>0</td><td>0</td><td>yes</td><td></td><td></td><td></td><td>blast</td><td>ucagccacugugucccccuccu</td><td>uugggggcagagguggcacggu</td><td>uugggggcagagguggcacgguguuuguuguuaacucagccacugugucccccuccu</td><td>gi|307603369|gb|CM001007.1|:63769129..6376</td></tr>
<tr><td>gi|307603381|gb|CM000995.1|_26114</td><td>2.3</td><td>0.81 ± 0.02</td><td></td><td>34</td><td>34</td><td>0</td><td>0</td><td>yes</td><td></td><td></td><td></td><td>blast</td><td>ccauugagcagugucggc</td><td>ugaaggcugccaggug</td><td>ccauugagcagugucggcgagagggcuucagccagaagaugcaggagcagaagaaugaaggcugccaggug</td><td>gi|307603381|gb|CM000995.1|:155836906..155</td></tr>
<tr><td>gi|307603379|gb|CM000997.1|_32306</td><td>2.3</td><td>0.81 ± 0.02</td><td></td><td>37</td><td>37</td><td>0</td><td>0</td><td>yes</td><td></td><td></td><td></td><td>blast</td><td>acuuucuagauguucccggaag</td><td>ucugggaacaucuacaaaguga</td><td>ucugggaacaucuacaaagugaaguagaaagaaugcaagagcucacuuucuagauguucccggaag</td><td>gi|307603379|gb|CM000997.1|:4707342..47074</td></tr>
<tr><td>gi|307603382|gb|CM000994.1|_24412</td><td>2.3</td><td>0.81 ± 0.02</td><td></td><td>43</td><td>43</td><td>0</td><td>0</td><td>yes</td><td></td><td></td><td></td><td>blast</td><td>uggcagcagguuugcucggagcag</td><td>gcuccgggcagaaucgugauguuauc</td><td>gcuccgggcagaaucgugauguuaucugcuggcagcagguuugcucggagcag</td><td>gi|307603382|gb|CM000994.1|:188529835..188</td></tr>
<tr><td>gi|307603372|gb|CM001004.1|_3734</td><td>2.3</td><td>0.81 ± 0.02</td><td></td><td>28</td><td>28</td><td>0</td><td>0</td><td>yes</td><td></td><td></td><td></td><td>blast</td><td>cuaguaagacugccaccgcacugc</td><td>aggggcacuuggcagcuagcu</td><td>cuaguaagacugccaccgcacugcuagagggcggucggccauauacagagaauaugggcagucauucugggaggggcacuuggcagcuagcu</td><td>gi|307603372|gb|CM001004.1|:94276822..9427</td></tr>
<tr><td>gi|307603372|gb|CM001004.1|_4083</td><td>2.3</td><td>0.81 ± 0.02</td><td></td><td>80</td><td>80</td><td>0</td><td>0</td><td>yes</td><td></td><td></td><td></td><td>blast</td><td>uccugcccccuuucccuguaga</td><td>uaagggugga ugggggcagagcu</td><td>uaagggugga ugggggcagagcucggucugaguccugcccccuuucccuguaga</td><td>gi|307603372|gb|CM001004.1|:105208496..105</td></tr>
<tr><td>gi|307603363|gb|CM001013.1|_49128</td><td>2.3</td><td>0.81 ± 0.02</td><td></td><td>47</td><td>28</td><td>0</td><td>19</td><td>yes</td><td></td><td></td><td></td><td>blast</td><td>guugcggaggcacccgaagca</td><td>uuccugugcagcagcuccc</td><td>uuccugugcagcagcucccguugcggaggcacccgaagca</td><td>gi|307603363|gb|CM001013.1|:34529424..3452</td></tr>
<tr><td>gi|307603372|gb|CM001004.1|_5425</td><td>2.3</td><td>0.81 ± 0.02</td><td></td><td>74</td><td>40</td><td>34</td><td>0</td><td>yes</td><td></td><td></td><td></td><td>blast</td><td>uguguuaggacagggaua</td><td>uacuuguccagcauaua</td><td>uguguuaggacagggauacggacggcuggggauguagcucagcgucugaguacuuguccagcauaua</td><td>gi|307603372|gb|CM001004.1|:86688284..8668</td></tr>
<tr><td>gi|307603364|gb|CM001012.1|_20509</td><td>2.3</td><td>0.81 ± 0.02</td><td></td><td>45</td><td>45</td><td>0</td><td>0</td><td>yes</td><td></td><td></td><td></td><td>blast</td><td>gguuccuggaagcugaag</td><td>ccagcucccgccaggagcaaa</td><td>ccagcucccgccaggagcaaagcugaccgaaggcgcgcccagcugcugguuccuggaagcugaag</td><td>gi|307603364|gb|CM001012.1|:56669444..5666</td></tr>
<tr><td>gi|307603373|gb|CM001003.1|_2270</td><td>2.3</td><td>0.81 ± 0.02</td><td></td><td>53</td><td>53</td><td>0</td><td>0</td><td>yes</td><td></td><td></td><td></td><td>blast</td><td>ugacucaagcccagcucaca</td><td>ggggcuggggaauccuggucacc</td><td>ggggcuggggaauccuggucaccugagcaggagggugacucaagcccagcucaca</td><td>gi|307603373|gb|CM001003.1|:121483046..121</td></tr>
<tr><td>gi|307603366|gb|CM001010.1|_16902</td><td>2.3</td><td>0.81 ± 0.02</td><td></td><td>33</td><td>33</td><td>0</td><td>0</td><td>yes</td><td></td><td></td><td></td><td>blast</td><td>aaucuugucacucuagccaucu</td><td>cacucuggagucucaagaaggg</td><td>aaucuugucacucuagccaucuaggugggggucccaaccuggucacucuggagucucaagaaggg</td><td>gi|307603366|gb|CM001010.1|:56409277..5640</td></tr>
<tr><td>gi|307603374|gb|CM001002.1|_45830</td><td>2.3</td><td>0.81 ± 0.02</td><td></td><td>528</td><td>528</td><td>0</td><td>0</td><td>yes</td><td></td><td></td><td></td><td>blast</td><td>gugaguggagacucgguagaggua</td><td>ccucuccuguucuccaucuag</td><td>gugaguggagacucgguagagguagagguggggaguccugcccuugucggccucaauggccucuccuguucuccaucuag</td><td>gi|307603374|gb|CM001002.1|:56989722..5698</td></tr>
<tr><td>gi|307603367|gb|CM001009.1|_14854</td><td>2.3</td><td>0.81 ± 0.02</td><td></td><td>49</td><td>49</td><td>0</td><td>0</td><td>yes</td><td></td><td></td><td></td><td>blast</td><td>gugggguacgaugggugguca</td><td>accuccucggugccuagaag</td><td>gugggguacgaugggguggucagaagaaggggcuggggcccccucugcaccuccucggugccuagaag</td><td>gi|307603367|gb|CM001009.1|:37992352..3799</td></tr>
<tr><td>gi|307603370|gb|CM001006.1|_8480</td><td>2.3</td><td>0.81 ± 0.02</td><td></td><td>39</td><td>39</td><td>0</td><td>0</td><td>yes</td><td></td><td></td><td></td><td>blast</td><td>ucuuucccuugcuaccauugucu</td><td>acagggaagggaggggaagggc</td><td>ucuuucccuugcuaccauugucugccuauuaaaugcacagggaagggaggggaagggc</td><td>gi|307603370|gb|CM001006.1|:13731128..1373</td></tr>
<tr><td>gi|307603379|gb|CM000997.1|_33590</td><td>2.3</td><td>0.81 ± 0.02</td><td></td><td>498</td><td>498</td><td>0</td><td>0</td><td>yes</td><td></td><td></td><td></td><td>blast</td><td>guaucugcaacuucggagg</td><td>acugaagcggauacuc</td><td>acugaagcggauacuccagguaucugcaacuucggagg</td><td>gi|307603379|gb|CM000997.1|:140136284..140</td></tr>
<tr><td>gi|307603379|gb|CM000997.1|_31814</td><td>2.3</td><td>0.81 ± 0.02</td><td></td><td>37</td><td>37</td><td>0</td><td>0</td><td>yes</td><td></td><td></td><td></td><td>blast</td><td>uuuccagcuucucccacuucagcc</td><td>guggagagagagaagcugggccca</td><td>uuuccagcuucucccacuucagccuguucccacggcaccgggguuucuacuccuguacccucugcagguggagagagagaagcugggccca</td><td>gi|307603379|gb|CM000997.1|:128429362..128</td></tr>
<tr><td>gi|307603367|gb|CM001009.1|_15385</td><td>2.3</td><td>0.81 ± 0.02</td><td></td><td>151</td><td>151</td><td>0</td><td>0</td><td>yes</td><td></td><td></td><td></td><td>blast</td><td>auauccagggagagaggg</td><td>cgaaggccuccugugagc</td><td>auauccagggagagagggcgcucacgcccgaaggccuccugugagc</td><td>gi|307603367|gb|CM001009.1|:10447144..1044</td></tr>
<tr><td>gi|307603373|gb|CM001003.1|_1772</td><td>2.3</td><td>0.81 ± 0.02</td><td></td><td>36</td><td>36</td><td>0</td><td>0</td><td>yes</td><td></td><td></td><td></td><td>blast</td><td>guggcaggccuaccuggag</td><td>ugagggugggccagcugauaccc</td><td>ugagggugggccagcugauaccccaaguuuauggaacccuuuguccacuuagggggauccuggaguggcaggccuaccuggag</td><td>gi|307603373|gb|CM001003.1|:76534776..7653</td></tr>
<tr><td>gi|307603367|gb|CM001009.1|_15000</td><td>2.3</td><td>0.81 ± 0.02</td><td></td><td>61</td><td>61</td><td>0</td><td>0</td><td>yes</td><td></td><td></td><td></td><td>blast</td><td>uaugucacugggcauuggcuuuga</td><td>caagccaggcccagugucacucu</td><td>uaugucacugggcauuggcuuugagguuucaaaugcucaagccaggcccagugucacucu</td><td>gi|307603367|gb|CM001009.1|:57610898..5761</td></tr>
<tr><td>gi|307603381|gb|CM000995.1|_27486</td><td>2.3</td><td>0.81 ± 0.02</td><td></td><td>87</td><td>87</td><td>0</td><td>0</td><td>yes</td><td></td><td></td><td></td><td>blast</td><td>uuuccuccccagugcuucuaauu</td><td>aacgaggcucuggggaaggaaaga</td><td>aacgaggcucuggggaaggaaagaacggugcauuuggaaaaggugugucuuuccuccccagugcuucuaauu</td><td>gi|307603381|gb|CM000995.1|:115680512..115</td></tr>
<tr><td>gi|307603379|gb|CM000997.1|_33596</td><td>2.3</td><td>0.81 ± 0.02</td><td></td><td>968</td><td>968</td><td>0</td><td>0</td><td>yes</td><td></td><td></td><td></td><td>blast</td><td>ugccucugcucucccugacg</td><td>acaggguggggcaggcguaga</td><td>acaggguggggcaggcguagaccuuccugccucugcucucccugacg</td><td>gi|307603379|gb|CM000997.1|:140602630..140</td></tr>
<tr><td>gi|307603376|gb|CM001000.1|_39958</td><td>2.3</td><td>0.81 ± 0.02</td><td></td><td>64</td><td>64</td><td>0</td><td>0</td><td>yes</td><td></td><td></td><td></td><td>blast</td><td>agcauggcugcuugugacacuggu</td><td>cggugucuccaaggccaggcugc</td><td>agcauggcugcuugugacacugguaggacccgagccggugucuccaaggccaggcugc</td><td>gi|307603376|gb|CM001000.1|:53315326..5331</td></tr>
<tr><td>gi|307603372|gb|CM001004.1|_4773</td><td>2.3</td><td>0.81 ± 0.02</td><td></td><td>301</td><td>301</td><td>0</td><td>0</td><td>yes</td><td></td><td></td><td></td><td>blast</td><td>uguggcuuugaggaguugggaag</td><td>ucccaucuucuccgagccaugacu</td><td>uguggcuuugaggaguugggaagagguaacccacuucucucccaucuucuccgagccaugacu</td><td>gi|307603372|gb|CM001004.1|:50069470..5006</td></tr>
<tr><td>gi|307603378|gb|CM000998.1|_36612</td><td>2.3</td><td>0.81 ± 0.02</td><td></td><td>77</td><td>77</td><td>0</td><td>0</td><td>yes</td><td></td><td></td><td></td><td>blast</td><td>ugccuggggccucugcagcac</td><td>acugcagauucuccuggcaug</td><td>acugcagauucuccuggcauguaugcaccugccuggggccucugcagcac</td><td>gi|307603378|gb|CM000998.1|:139225557..139</td></tr>
<tr><td>gi|307603369|gb|CM001007.1|_11334</td><td>2.3</td><td>0.81 ± 0.02</td><td></td><td>49</td><td>49</td><td>0</td><td>0</td><td>yes</td><td></td><td></td><td></td><td>blast</td><td>aggccagcgccugugggg</td><td>cuccuggcuagcacucc</td><td>aggccagcgccuguggggcaaaagggcccggucuagcgcccuggccgcccuccuggcuagcacucc</td><td>gi|307603369|gb|CM001007.1|:67488153..6748</td></tr>
<tr><td>gi|307603369|gb|CM001007.1|_12149</td><td>2.3</td><td>0.81 ± 0.02</td><td></td><td>151</td><td>102</td><td>49</td><td>0</td><td>yes</td><td></td><td></td><td></td><td>blast</td><td>ucggagcuuugcugaaacug</td><td>gagagcgcggcgccgagg</td><td>ucggagcuuugcugaaacugcacaaaaaaucgagcuggggggguucccugguccccgauguuggggcggagagcgcggcgccgagg</td><td>gi|307603369|gb|CM001007.1|:62301079..6230</td></tr>
<tr><td>gi|307603366|gb|CM001010.1|_18010</td><td>2.3</td><td>0.81 ± 0.02</td><td></td><td>2561</td><td>2561</td><td>0</td><td>0</td><td>yes</td><td></td><td></td><td></td><td>blast</td><td>agguuauagaaauuucagacaaua</td><td>aauuuugaaguugguuugaggccuug</td><td>aauuuugaaguugguuugaggccuugcccugguucuuuccagggcaagguuauagaaauuucagacaaua</td><td>gi|307603366|gb|CM001010.1|:62806335..6280</td></tr>
<tr><td>gi|307603381|gb|CM000995.1|_27660</td><td>2.3</td><td>0.81 ± 0.02</td><td></td><td>79</td><td>79</td><td>0</td><td>0</td><td>yes</td><td></td><td></td><td></td><td>blast</td><td>ugcugagcauggacugaccucu</td><td>gggucaggguccaugcaugcaug</td><td>gggucaggguccaugcaugcaugccgugcugucugccaugcugagcauggacugaccucu</td><td>gi|307603381|gb|CM000995.1|:126714197..126</td></tr>
<tr><td>gi|307603376|gb|CM001000.1|_40003</td><td>2.3</td><td>0.81 ± 0.02</td><td></td><td>29</td><td>29</td><td>0</td><td>0</td><td>yes</td><td></td><td></td><td></td><td>blast</td><td>gucgcagcaaucggacagcuuaa</td><td>gggcugucccccagugucuc</td><td>gggcugucccccagugucuccagugccucuggugcuccagaugccucuggagucgcagcaaucggacagcuuaa</td><td>gi|307603376|gb|CM001000.1|:58766373..5876</td></tr>
<tr><td>gi|307603371|gb|CM001005.1|_6558</td><td>2.3</td><td>0.81 ± 0.02</td><td></td><td>32</td><td>32</td><td>0</td><td>0</td><td>yes</td><td></td><td></td><td></td><td>blast</td><td>auggcacuuuaucauagcaaca</td><td>uugcugggagccagugcccuaa</td><td>auggcacuuuaucauagcaacaguuuauauguuuguugcugggagccagugcccuaa</td><td>gi|307603371|gb|CM001005.1|:54631068..5463</td></tr>
<tr><td>gi|307603375|gb|CM001001.1|_44946</td><td>2.3</td><td>0.81 ± 0.02</td><td></td><td>64</td><td>44</td><td>0</td><td>20</td><td>yes</td><td></td><td></td><td></td><td>blast</td><td>cugcccucugcuuuccuuca</td><td>ggggguugggggguggggg</td><td>ggggguugggggguggggucagaugggaguuguaggacugaccucugcccucugcuuuccuuca</td><td>gi|307603375|gb|CM001001.1|:112084063..112</td></tr>
<tr><td>gi|307603368|gb|CM001008.1|_12952</td><td>2.3</td><td>0.81 ± 0.02</td><td></td><td>29</td><td>29</td><td>0</td><td>0</td><td>yes</td><td></td><td></td><td></td><td>blast</td><td>auugaggaugacggcugacc</td><td>ugagacugaacucuaucc</td><td>auugaggaugacggcugaccuuagacccuugcccaugcugggcaagcagucugggacugagacugaacucuaucc</td><td>gi|307603368|gb|CM001008.1|:78907111..7890</td></tr>
<tr><td>gi|307603375|gb|CM001001.1|_43534</td><td>2.3</td><td>0.81 ± 0.02</td><td></td><td>35</td><td>34</td><td>1</td><td>0</td><td>yes</td><td></td><td></td><td></td><td>blast</td><td>cucugggcugagcugugga</td><td>uaggcugagcuguggaggg</td><td>cucugggcugagcuguggagggugcugguggacucugggcugagccguggagggugcuggcggacucuaggcugagcuguggaggg</td><td>gi|307603375|gb|CM001001.1|:96482909..9648</td></tr>
<tr><td>gi|307603367|gb|CM001009.1|_15505</td><td>2.3</td><td>0.81 ± 0.02</td><td></td><td>31</td><td>31</td><td>0</td><td>0</td><td>yes</td><td></td><td></td><td></td><td>blast</td><td>gguguggccuucggccugcucu</td><td>aggggcugggagccgcccacac</td><td>gguguggccuucggccugcucuaugugcucaaucacucggaggggcugggagccgcccacac</td><td>gi|307603367|gb|CM001009.1|:18228160..1822</td></tr>
</table>

gi|307603375|gb|CM001001.1|_42883	2.3	0.81 ± 0.02		418	418	0	0	yes				blast	uaaugucugcaugagggucucuau	ggagacagcccauuuagacaugagug	ggagacagcccauuuagacaugaguguuccaaagucuuugcauaaugucugcaugagggucucuau	gi|307603375|gb|CM001001.1|:11732456..1173
gi|307603373|gb|CM001003.1|_612	2.3	0.81 ± 0.02		132	132	0	0	yes				blast	acccaggaagcuggggcaggaga	uccuaccucagucuccuugagcgc	acccaggaagcuggggcaggagagcgugguaauccuccuaccucagucuccuugagcgc	gi|307603373|gb|CM001003.1|:79281613..7928
gi|307603377|gb|CM000999.1|_38756	2.3	0.81 ± 0.02		36	36	0	0	yes				blast	gcccaggcugugcuucag	ggaaccuagcagggcuc	ggaaccuagcagggcucugaggcccaggcugugcuucag	gi|307603377|gb|CM000999.1|:89302331..8930
gi|307603372|gb|CM001004.1|_5974	2.3	0.81 ± 0.02		58	58	0	0	yes				blast	ucugucccacacacccucugacu	agggagaguggcugggaccuaguggagg	agggagaguggcugggaccuaguggaggaaccuuuccucugucccacacacccucugacu	gi|307603372|gb|CM001004.1|:113571273..113
gi|307603376|gb|CM001000.1|_40183	2.3	0.81 ± 0.02		83	83	0	0	yes				blast	uugacucuuccuuccugugcag	gagcaggaagaagagaaga	gagcaggaagaagagaagacaugcauccugaaaugucagucccucuugacucuuccuuccugugcag	gi|307603376|gb|CM001000.1|:87430830..8743
gi|307603377|gb|CM000999.1|_37316	2.3	0.81 ± 0.02		39	39	0	0	yes				blast	uguugggcucgagggcauacc	ugcuccuccugcccaacugu	uguugggcucgagggcauaccuaguaugugcuuccuguacuauggugcuccuccugcccaacugu	gi|307603377|gb|CM000999.1|:65851597..6585
gi|307603369|gb|CM001007.1|_11932	2.3	0.81 ± 0.02		44	44	0	0	yes				blast	cauccugccuacaccuccuaag	ugagaaguggaggcaggaaggc	ugagaaguggaggcaggaaggccuguguaguagcaccuugaaugucuagucauccugccuacaccuccuaag	gi|307603369|gb|CM001007.1|:51549055..5154
gi|307603364|gb|CM001012.1|_20631	2.3	0.81 ± 0.02		107	107	0	0	yes				blast	aaaguccugggguacgcugga	cagcgagcugcccagggcucu	aaaguccugggguacgcuggaggcuuuggagacaugagaugaugccagcgagcugcccagggcucu	gi|307603364|gb|CM001012.1|:5153882..51539
gi|307603376|gb|CM001000.1|_39732	2.3	0.81 ± 0.02		32	32	0	0	yes				blast	gaugugcaggccggcaggggcua	gcaggggcugccugugugugcauugg	gaugugcaggccggcaggggcuaguggcaggggcugccugugugugcauugg	gi|307603376|gb|CM001000.1|:29601289..2960
gi|307603372|gb|CM001004.1|_4380	2.3	0.81 ± 0.02		93	93	0	0	yes				blast	uuuggcccuaggcacugcucuugu	aggacagaccuggagcuguagc	uuuggcccuaggcacugcucuuguuuugcucaaagccacaggacagaccuggagcuguagc	gi|307603372|gb|CM001004.1|:121384709..121
gi|307603379|gb|CM000997.1|_30943	2.3	0.81 ± 0.02		49	49	0	0	yes				blast	aagcugcgggccggcgugaag	ucucccacccggaguuauuucc	ucucccacccggaguuauuuccggucucuggaaaccgaggcccggaagcugcgggccggcgugaag	gi|307603379|gb|CM000997.1|:19502184..1950
gi|307603378|gb|CM000998.1|_35344	2.3	0.81 ± 0.02		37	37	0	0	yes				blast	gagccacagaggaaccug	gcccuucuguggccua	gagccacagaggaaccugugcuggagagcaggccagugaggcagccuggagagcccuucuguggccua	gi|307603378|gb|CM000998.1|:147819031..147
gi|307603381|gb|CM000995.1|_26984	2.2	0.81 ± 0.02		40	40	0	0	yes				blast	aggcuguuggcguccgca	aggagggcaagaggaaag	aggagggcaagaggaaagggaggaggagauuuggguuuccaauccccaggcgaggcuguuggcguccgca	gi|307603381|gb|CM000995.1|:44973019..4497
gi|307603382|gb|CM000994.1|_22863	2.2	0.81 ± 0.02		40	40	0	0	yes				blast	ugcgugucagaaugaucagaug	ucuggucauuuagaccugcccg	ugcgugucagaaugaucagaggucuuauuagugaaaagucaucuggucauuuagaccugcccg	gi|307603382|gb|CM000994.1|:3634276..36343
gi|307603381|gb|CM000995.1|_25530	2.2	0.81 ± 0.02		156	156	0	0	yes				blast	ugugucauuggcugugggcuuuga	caagccaaggccaauggcucucu	ugugucauuggcugugggcuuugagguuucaagugaucaagccaaggccaauggcucucu	gi|307603381|gb|CM000995.1|:106264666..106
gi|307603376|gb|CM001000.1|_42811	2.2	0.81 ± 0.02		223	223	0	0	yes				blast	augcaaguguggauucug	cauuuuacucugcugcauag	augcaaguguggauucugcggcgcaggcucugagccugccccuaccgugccucauuuuacucugcugcauag	gi|307603376|gb|CM001000.1|:151015814..151
gi|307603380|gb|CM000996.1|_28815	2.2	0.81 ± 0.02		64	64	0	0	yes				blast	ccuguggcagcuucccuccuuu	aggaaggaggucacagccc	aggaaggaggucacagcccagaaaacuggccuguggcagcuucccuccuuu	gi|307603380|gb|CM000996.1|:88342702..8834
gi|307603377|gb|CM000999.1|_37196	2.2	0.81 ± 0.02		51	51	0	0	yes				blast	aaacccuaagacagucacugug	cugugaguugucuuaggauuucu	aaacccuaagacagucacuguguuaguuagguuacuauuacugugaguugucuuaggauuucu	gi|307603377|gb|CM000999.1|:48467145..4846
gi|307603366|gb|CM001010.1|_16549	2.2	0.81 ± 0.02		30	30	0	0	yes				blast	cuggguucacuggagag	uguccagaacucuccagag	uguccagaacucuccagagguuucuggguucacuggagag	gi|307603366|gb|CM001010.1|:35109272..3510
gi|307603377|gb|CM000999.1|_38792	2.2	0.81 ± 0.02		149	149	0	0	yes				blast	uuagaauguaaauagagaggaa	ccucucuauuuacauucuaaca	ccucucuauuuacauucuaacauuuccaaaaaccuuuggggguuagaauguaaauagagaggaa	gi|307603377|gb|CM000999.1|:92045756..9204
gi|307603371|gb|CM001005.1|_7269	2.2	0.81 ± 0.02		29	29	0	0	yes				blast	gugaugaguacugagcca	guuugggcuggauug	guuugggcuggauugcuauggcagugaugaguacugagcca	gi|307603371|gb|CM001005.1|:110956774..110
gi|307603381|gb|CM000995.1|_26152	2.2	0.81 ± 0.02		59	59	0	0	yes				blast	ccaagggggucccuggaacagcag	gguccuggcuccucugacca	ccaagggggucccuggaacagcagacaucugacugucuggucccuggcuccucugacca	gi|307603381|gb|CM000995.1|:157296901..157
gi|307603367|gb|CM001009.1|_14491	2.2	0.81 ± 0.02		62	46	0	16	yes				blast	cuccacacugugccugaccugu	ucuggcauuacuguagagcaugg	ucuggcauuacuguagagcauggucucagaugucugcuccacacugugccugaccugu	gi|307603367|gb|CM001009.1|:11744790..1174
gi|307603370|gb|CM001006.1|_10301	2.2	0.81 ± 0.02		2427	2386	0	41	yes				blast	acaucaacguggagcugagucu	agaaaaggcuccggguugac	acaucaacguggagcugagucuucuugggaagaagaagaaaaggcuccggguugac	gi|307603370|gb|CM001006.1|:72592614..7259
gi|307603377|gb|CM000999.1|_38712	2.2	0.81 ± 0.02		59	59	0	0	yes				blast	gcguagagacugaacggccgg	ugguugguucagucugggugaagcac	ugguugguucagucugggugaagcacauuaugcguagagacugaacggccgg	gi|307603377|gb|CM000999.1|:87788429..8778
gi|307603374|gb|CM001002.1|_45938	2.2	0.81 ± 0.02		35	35	0	0	yes				blast	uuggaaguggagcccgcg	cggggcgcgggaga	uuggaaguggagcccgcguugcaaaggcggcgcggggcgcgggaga	gi|307603374|gb|CM001002.1|:64129487..6412
gi|307603371|gb|CM001005.1|_6779	2.2	0.81 ± 0.02		50	50	0	0	yes				blast	ugacccuguguucuuccccag	ggugaagacagcagguauacg	ggugaagacagcagguauacgaagagugauuggaacgucagccccgcccuucugacccuguguucuuccccag	gi|307603371|gb|CM001005.1|:78015533..7801
gi|307603367|gb|CM001009.1|_15734	2.2	0.81 ± 0.02		31	31	0	0	yes				blast	cuggagcccugcgaggaccc	caccugggggcucuccaggc	caccugggggcucuccaggcucuguggggaggcagagccggaaaaccuccccacuuaguuccuggagcccugcgaggaccc	gi|307603367|gb|CM001009.1|:43802203..4380
gi|307603368|gb|CM001008.1|_14104	2.2	0.81 ± 0.02		153	153	0	0	yes				blast	acugugaaccuuagggcugca	cggccuaaaugaguuugcgcagacu	cggccuaaaugaguuugcgcagacucagggcgggcucucagccugucacugugaaccuuagggcugca	gi|307603368|gb|CM001008.1|:85464816..8546
gi|307603372|gb|CM001004.1|_3627	2.2	0.81 ± 0.02		24129	18499	1725	3905	yes				blast	ggagggaacgcagucugagugga	cuugacugcagcugcccucc	cuugacugcagcugcccuccugccauugaugaucguucuucccucuuugggagggugagagggagggaacgcagucugagugga	gi|307603372|gb|CM001004.1|:87275918..8727
gi|307603377|gb|CM000999.1|_38045	2.2	0.81 ± 0.02		29	29	0	0	yes				blast	cgaggugggacucgaaccc	gacucaaacccacgaucucgca	gacucaaacccacgaucucgcaacaaaacgcgaggugggacucgaaccc	gi|307603377|gb|CM000999.1|:148587910..148
gi|307603368|gb|CM001008.1|_13279	2.2	0.81 ± 0.02		51	51	0	0	yes				blast	gcgugcaggcuggagcggugug	ggcggcuggagcugggcgggcgu	ggcggcuggagcugggcgggcgugggggcgaggccaggcgugcaggcuggagcggugug	gi|307603368|gb|CM001008.1|:98462773..9846
gi|307603381|gb|CM000995.1|_28041	2.2	0.81 ± 0.02		32	32	0	0	yes				blast	gggcuugacaccaccuggu	cggguggggaaacugaggccucg	gggcuugacaccaccugguggccccggcugugcagcugagauuggguuucggguggggaaacugaggccucg	gi|307603381|gb|CM000995.1|:163427897..163
gi|307603372|gb|CM001004.1|_5676	2.2	0.81 ± 0.02		89	89	0	0	yes				blast	uaacugcuguuucugccucuga	agaggcacagggcagguaag	agaggcacagggcagguaagguagguuucauuguuccuccggcacccugguucgccuuaacugcuguuucugccucuga	gi|307603372|gb|CM001004.1|:100355396..100
gi|307603382|gb|CM000994.1|_23778	2.2	0.81 ± 0.02		29	29	0	0	yes				blast	agggacaggccugcagcu	cgccagcaggcugguacuugg	cgccagcaggcugguacuugggaagggacaggccugcagcu	gi|307603382|gb|CM000994.1|:129427861..129
gi|307603378|gb|CM000998.1|_34292	2.2	0.81 ± 0.02		30	30	0	0	yes				blast	accuagggcgcggacagau	aaguuccgcccucaggcag	accuagggcgcggacagaugcuaaguuccgcccucaggcag	gi|307603378|gb|CM000998.1|:45841823..4584
gi|307603365|gb|CM001011.1|_18998	2.2	0.81 ± 0.02		98	98	0	0	yes				blast	ucuggcucuugggaucuuucugu	agauaggucccugagcccugagg	ucuggcucuugggaucuuucugucuccucuucuacacagauaggucccugagcccugagg	gi|307603365|gb|CM001011.1|:83271832..8327
gi|307603371|gb|CM001005.1|_7328	2.2	0.81 ± 0.02		33	33	0	0	yes				blast	aguggucugggcuggucuuggaa	ccaagaugacccugcccucc	aguggucugggcuggucuuggaaauguccaagaugacccugcccucc	gi|307603371|gb|CM001005.1|:111741100..111
gi|307603379|gb|CM000997.1|_32699	2.2	0.81 ± 0.02		32	32	0	0	yes				blast	gaggcuguggauagaaggugcuaa	uccccuaguucucagcgucug	gaggcuguggauagaaggugcuaagcuuaugcuccccuaguucucagcgucug	gi|307603379|gb|CM000997.1|:57002625..5700
gi|307603372|gb|CM001004.1|_3631	2.2	0.81 ± 0.02		24129	18499	1725	3905	yes				blast	ggagggaacgcagucugagugga	cuugacugcagcugcccucc	cuugacugcagcugcccuccugccauugaugaucguucuucccucuuugggagggugagagggagggaacgcagucugagugga	gi|307603372|gb|CM001004.1|:87285000..8728
gi|307603372|gb|CM001004.1|_6052	2.2	0.81 ± 0.02		483	480	3	0	yes				blast	uccuccugugacuccuucuagg	ugggaggaugucagagcaga	ugggaggaugucagagcagaccacagacaugaggccucccucugugacuccuucuagg	gi|307603372|gb|CM001004.1|:116401448..116
gi|307603371|gb|CM001005.1|_8357	2.2	0.81 ± 0.02		56	35	21	0	yes				blast	gcagcucuguccagccuag	agagcaugacugaggcgg	gcagcucuguccagccuagauggcgaccugucuggcagguacuacgcgcucaagagcaugacugaggcgg	gi|307603371|gb|CM001005.1|:112909189..112
gi|307603370|gb|CM001006.1|_9557	2.2	0.81 ± 0.02		81	81	0	0	yes				blast	cacgccgcggcucacacacacagu	ugugucgugguggcacgccgcagcu	cacgccgcggcucacacacacaguuagccaucacaacugugucgugguggcacgccgcagcu	gi|307603370|gb|CM001006.1|:12295734..1229
gi|307603380|gb|CM000996.1|_28451	2.2	0.81 ± 0.02		117	117	0	0	yes				blast	ucucaccagcucuguggccuagg	uaggcugcaugcuggugaugga	uaggcugcaugcuggugauggacuucaagcugccucucucaccagcucuguggccuagg	gi|307603380|gb|CM000996.1|:34196714..3419
gi|307603382|gb|CM000994.1|_23152	2.2	0.81 ± 0.02		28	28	0	0	yes				blast	cugaucuuucucgggaga	agcugagaaaucugca	agcugagaaaucugcagacagguuuaacccuguccucacacuuggagggggccugcugcuuugcugcugaucuuucucgggaga	gi|307603382|gb|CM000994.1|:43358726..4335
gi|307603380|gb|CM000996.1|_28681	2.2	0.81 ± 0.02		40	40	0	0	yes				blast	gccggcgcugagcacaacc	ugcggcuccgguugccggcgu	ugcggcuccgguugccggcgugacuuugagcucuucccgcucugcugccggcgcugagcacaacc	gi|307603380|gb|CM000996.1|:67234048..6723
gi|307603379|gb|CM000997.1|_31985	2.2	0.81 ± 0.02		68	68	0	0	yes				blast	uggagaaaagcucugcugccc	gcagcauaagagcuguucuucugg	gcagcauaagagcuguucuucugguuucccuuggagaaaagcucugcugccc	gi|307603379|gb|CM000997.1|:135701118..135
gi|307603375|gb|CM001001.1|_43292	2.2	0.81 ± 0.02		36	36	0	0	yes				blast	gagccugcugugcauagg	ugugucagccuuggcugga	gagccugcugugcauaggccaaggugugugucagccuuggcugga	gi|307603375|gb|CM001001.1|:73226803..7322
gi|307603371|gb|CM001005.1|_8198	2.2	0.81 ± 0.02		328	328	0	0	yes				blast	ugaagcacuuguggcucacugga	aaagcugccauuuacgugcuugguc	aaagcugccauuuacgugcuuggucugggaccaggguucugguugaagcacuuguggcucacugga	gi|307603371|gb|CM001005.1|:99668704..9966
gi|307603370|gb|CM001006.1|_8461	2.2	0.81 ± 0.02		31	30	1	0	yes				blast	gaggacugugugcaugcaua	uccagcgcacaguucagcu	uccagcgcacaguucagcuguucgcuggcagaggccggcgucuauacagauuagaggacugugugcaugcaua	gi|307603370|gb|CM001006.1|:9762770..97628
gi|307603369|gb|CM001007.1|_11148	2.2	0.81 ± 0.02		34	34	0	0	yes				blast	acugcagcugccacccuug	agggggcagcgacaggag	acugcagcugccacccuugugcccaggagaugggaggggacaaguggagggggcagcgacaggag	gi|307603369|gb|CM001007.1|:55673136..5567
gi|307603382|gb|CM000994.1|_24238	2.2	0.81 ± 0.02		275	275	0	0	yes				blast	ugucucugcuccuacccuccaga	ggcggggaagggcagcaagaccggc	ggcggggaagggcagcaagaccggcaugagcauagaugcugucucugcuccuacccuccaga	gi|307603382|gb|CM000994.1|:174210216..174
gi|307603364|gb|CM001012.1|_20614	2.2	0.81 ± 0.02		43	43	0	0	yes				blast	gguggcuguuagcgcggagg	ugaguaucugggggugggcgg	ugaguaucugggggugggcgggguuaagagagcgggucugagggcuggcgccuguggcuguuagcgcggagg	gi|307603364|gb|CM001012.1|:4942808..49428
gi|307603381|gb|CM000995.1|_28050	2.2	0.81 ± 0.02		314	314	0	0	yes				blast	agguuaccggagggacacg	gcgccgucccggaaaccacu	gcgccgucccggaaaccacuccgagguuaccggagggacacg	gi|307603381|gb|CM000995.1|:164322930..164
gi|307603367|gb|CM001009.1|_15265	2.2	0.81 ± 0.02		85	85	0	0	yes				blast	agugucugugcgggaucgg	gagcgugugcgagggcgagug	gagcgugugcgagggcgagugugcgcgcggugagugugcgcgagugucugugcgggaucgg	gi|307603367|gb|CM001009.1|:94791608..9479
gi|307603369|gb|CM001007.1|_12501	2.2	0.81 ± 0.02		38	38	0	0	yes				blast	ggcaggagcugcggauuccacggc	cgugggguuuccagcagcgcucgccgu	ggcaggagcugcggauuccacggcggcagcagccuuuccgugggguuuccagcagcgcucgccgu	gi|307603369|gb|CM001007.1|:118295000..118
gi|307603371|gb|CM001005.1|_7846	2.2	0.81 ± 0.02		110407	110407	0	0	yes				blast	aucucgguggaaccucca	gugguccugggcagg	gugguccugggcaggggucucaaaucucgguggaaccucca	gi|307603371|gb|CM001005.1|:55875909..5587
gi|307603376|gb|CM001000.1|_42228	2.2	0.81 ± 0.02		30	30	0	0	yes				blast	gccgaugcuggagaacagggccug	ggaacuugggucuuggccu	ggaacuugggucuuggccuaggaugcugcagacccaggagaugggguagcguaggccgaugcuggagaacagggccug	gi|307603376|gb|CM001000.1|:108042853..108
gi|307603373|gb|CM001003.1|_1096	2.2	0.81 ± 0.02		138	138	0	0	yes				blast	ccugggagcugcaauaccucug	gagcugcgugguuccugggcc	ccugggagcugcaauaccucugcuggggaggcaucuccucagagcugcgugguuccugggcc	gi|307603373|gb|CM001003.1|:116697398..116
gi|307603380|gb|CM000996.1|_29866	2.2	0.81 ± 0.02		2395	2395	0	0	yes				blast	ucuguggucuguuuguccguc	aggagacacaguccuugcaggca	aggagacacaguccuugcaggcaguuccagcacgucugugggucuguuuguccguc	gi|307603380|gb|CM000996.1|:55691205..5569
gi|307603377|gb|CM000999.1|_37706	2.2	0.81 ± 0.02		38	38	0	0	yes				blast	gcugcccggggacuguaa	gcaagucccucuggcaccg	gcugcccggggacuguaaggccgcuauggcagcagcaagucccucuggcaccg	gi|307603377|gb|CM000999.1|:114459562..114
gi|307603373|gb|CM001003.1|_1985	2.2	0.81 ± 0.02		31	31	0	0	yes				blast	caguuggagagguggagcccca	gaggcugccuccugug	caguuggagagguggagccccaagggauuaggugggaggcugccuccugug	gi|307603373|gb|CM001003.1|:84366587..8436
gi|307603365|gb|CM001011.1|_18930	2.2	0.81 ± 0.02		45	45	0	0	yes				blast	agugugucacuguaagggcugacu	ugugcccacugugacacacagu	agugugucacuguaagggcugacuugaagucuccuaugcucaagcugugcccacugugacacacagu	gi|307603365|gb|CM001011.1|:76125929..7612
gi|307603375|gb|CM001001.1|_43220	2.2	0.81 ± 0.02		64	64	0	0	yes				blast	uuucagggcggcaacgug	caguugcccucaccccggaauc	uuucagggcggcaacgugcagcguccuuaccuugagccugugcaguugcccucaccccggaauc	gi|307603375|gb|CM001001.1|:63472050..6347
gi|307603376|gb|CM001000.1|_41997	2.2	0.81 ± 0.02		481	435	0	46	yes				blast	uucgguuaucuagcuuuauga	acagcuagauaaccaaagagaga	uucgguuaucuagcuuuaugacggcucugguggcacucauacagcuagauaaccaaagagaga	gi|307603376|gb|CM001000.1|:86650160..8665
gi|307603376|gb|CM001000.1|_42138	2.2	0.81 ± 0.02		52	52	0	0	yes				blast	gcgcucagccuuagcaaccucugc	cuaggcugaagggcauugcguac	cuaggcugaagggcauugcguacggcggugcagcacccaaaagcuucccugugcgcucagccuuagcaaccucugc	gi|307603376|gb|CM001000.1|:104548524..104
gi|307603377|gb|CM000999.1|_37445	2.2	0.81 ± 0.02		29	29	0	0	yes				blast	agcugggagcuccugaagg	aaggggagcuuggccuc	aaggggagcuuggccuccccaauucugugugugggcucaguguggaagagcugggagcuccugaagg	gi|307603377|gb|CM000999.1|:83751501..8375
gi|307603368|gb|CM001008.1|_14079	2.2	0.81 ± 0.02		57	57	0	0	yes				blast	gggcagaagugaggcucuu	gggccuguaggacccug	gggcagaagugaggcucuuucuggacccugaagaauuccuaggggagggaggggccuguaggacccug	gi|307603368|gb|CM001008.1|:84247820..8424
gi|307603368|gb|CM001008.1|_13825	2.2	0.81 ± 0.02		248	248	0	0	yes				blast	ugcccugugccugccuuccugag	caggagcugucacaggugcugg	ugcccugugccugccuuccugagccuucuggggcuaccucaggagcugucacaggugcugg	gi|307603368|gb|CM001008.1|:75727768..7572
gi|307603374|gb|CM001002.1|_46588	2.2	0.81 ± 0.02		52	52	0	0	yes				blast	ugugaacuugcucaggaca	agcugagggagccacagc	ugugaacuugcucaggacacaaggaggcugcagaggccauugaagcugagggagccacagc	gi|307603374|gb|CM001002.1|:120866685..120
gi|307603378|gb|CM000998.1|_35014	2.2	0.81 ± 0.02		279	243	0	36	yes				blast	aaacaaaccagaggcucaca	ugugaaucuccggcgcguuuaa	aaacaaaccagaggcucacacugaugauugucauagugugaaucuccggcgcguuuaa	gi|307603378|gb|CM000998.1|:126262052..126
gi|307603380|gb|CM000996.1|_30222	2.2	0.81 ± 0.02		30	30	0	0	yes				blast	cauuggugagguacuggaag	ucuaggccugaaaauggu	ucuaggccugaaaauggugucacaugaugccauuggugagguacuggaag	gi|307603380|gb|CM000996.1|:94809831..9480
gi|307603377|gb|CM000999.1|_36796	2.2	0.81 ± 0.02		28	28	0	0	yes				blast	caacacaaggagucugcauguc	caugcucagcuuuggauacgcg	caacacaaggagucugcaugucuaagguggagacaugcucagcuuuggauacgcg	gi|307603377|gb|CM000999.1|:4455805..44558
gi|307603382|gb|CM000994.1|_23299	2.2	0.81 ± 0.02		31	31	0	0	yes				blast	aguuuggauggcugugugca	caggagcccaaaacagc	aguuuggauggcugugugcacacugggaacacagcaaaaaucugcuguggcuuucucuccugcaggagcccaaaacagc	gi|307603382|gb|CM000994.1|:61202216..6120
gi|307603367|gb|CM001009.1|_14474	2.2	0.81 ± 0.02		38	38	0	0	yes				blast	uuagggagcuggcucaaaaauag	augagagccagcucucuuuca	uuagggagcuggcucaaaaauaggguccucugguugaucccaugagagccagcucucuuuca	gi|307603367|gb|CM001009.1|:11069206..1106
gi|307603377|gb|CM000999.1|_37956	2.2	0.81 ± 0.02		94	94	0	0	yes				blast	auccgggacgagcccccca	uccacucgaguccugggauga	auccgggacgagcccccacauguaaggcccccgaaacugggggagacuuccacucgaguccugggauga	gi|307603377|gb|CM000999.1|:136391532..136
gi|307603379|gb|CM000997.1|_31568	2.2	0.81 ± 0.02		166	166	0	0	yes				blast	agucagggggcugaguaugacc	ucauccaguccaucccccugucagg	agucagggggcugaguaugacccucagaggucauccaguccaucccccugucagg	gi|307603379|gb|CM000997.1|:107542563..107
gi|307603368|gb|CM001008.1|_13149	2.2	0.81 ± 0.02		63	63	0	0	yes				blast	ccuggcugucuguccuucagc	ucaaggacaggcagccugag	ucaaggacaggcagccugagacaggacagcagcaucugucuccuggcugucuguccuucagc	gi|307603368|gb|CM001008.1|:88352836..8835
gi|307603365|gb|CM001011.1|_18822	2.2	0.81 ± 0.02		34	34	0	0	yes				blast	gaaagccagguaacaggugcug	gguccugaaacuuggcuuugcc	gaaagccagguaacaggugcugucccugggucucagguccugaaacuuggcuuugcc	gi|307603365|gb|CM001011.1|:65144179..6514
gi|307603380|gb|CM000996.1|_29319	2.2	0.81 ± 0.02		29	29	0	0	yes				blast	cacuucuccagcgcugccuuc	aggcagugacuguucagauguc	aggcagugacuguucagauguccaguuucuuugggacacuucuccagcgcugccuuc	gi|307603380|gb|CM000996.1|:118137661..118
gi|307603372|gb|CM001004.1|_5339	2.2	0.81 ± 0.02		362	362	0	0	yes				blast	uugcgaguugggaagggu	ccucaccucgacuugcaucc	uugcgaguugggaaggguuagugucccucaccucgacuugcaucc	gi|307603372|gb|CM001004.1|:82704057..8270
gi|307603381|gb|CM000995.1|_25916	2.1	0.81 ± 0.02		132	132	0	0	yes				blast	ugugaacuuggggccccucugu	agaggauguccaaguucuguguugagc	ugugaacuuggggccccucuguuuuggugauuucugagaggauguccaaguucuguguugagc	gi|307603381|gb|CM000995.1|:136772870..136
gi|307603376|gb|CM001000.1|_41826	2.1	0.81 ± 0.02		2092	2092	0	0	yes				blast	ugauauagccaagcccgacugu	agucaggcugcuggcuauauuccagg	agucaggcugcuggcuauauuccaggacaagucugguccugauauagccaagcccgacugu	gi|307603376|gb|CM001000.1|:68895353..6889
gi|307603377|gb|CM000999.1|_37049	2.1	0.81 ± 0.02		32	32	0	0	yes				blast	ucaaacugaccuugaacucaca	ugaguucaaaguuagcuggguc	ugaguucaaaguuagcugggucuaugagaucaaacugaccuugaacucaca	gi|307603377|gb|CM000999.1|:38434311..3843
gi|307603378|gb|CM000998.1|_36078	2.1	0.81 ± 0.02		91	91	0	0	yes				blast	acaaacuagacacccggg	ggggugcuaguuaguuc	ggggugcuaguuaguucuugucaacuugacacaaacuagacacccggg	gi|307603378|gb|CM000998.1|:108861778..108
gi|307603381|gb|CM000995.1|_25471	2.1	0.81 ± 0.02		32	32	0	0	yes				blast	ugcaucuguggccccacuuga	ccgugggucuccggagauucaga	ccgugggucuccggagauucagagggcuuuuaucccccugcaucuguggccccacuuga	gi|307603381|gb|CM000995.1|:92756003..9275
gi|307603366|gb|CM001010.1|_18210	2.1	0.81 ± 0.02		41	41	0	0	yes				blast	agugagcucaggaggccuguaguc	cuuaagggcuuccugggguuggacuuc	cuuaagggcuuccugggguuggacuuccugcuucagugagcucaggaggccuguaguc	gi|307603366|gb|CM001010.1|:87775704..8777
gi|307603380|gb|CM000996.1|_28769	2.1	0.81 ± 0.02		63	63	0	0	yes				blast	cucagccucugaugucuccaccac	ggugggcaaaagaaggagugagug	ggugggcaaaagaaggagugaguguuggagggggugguuugggaggggcuagccucugguaccgucagccucugaugucuccaccac	gi|307603380|gb|CM000996.1|:87717884..8771
gi|307603367|gb|CM001009.1|_15252	2.1	0.81 ± 0.02		28	28	0	0	yes				blast	aucaguagcaccgagcaguga	acugccucggguguaaugc	aucaguagcaccgagcagugaggcugucacuccagaagaccauaggugaguauagcucaucacugccucggguguaaugc	gi|307603367|gb|CM001009.1|:94657108..9465
gi|307603376|gb|CM001000.1|_42632	2.1	0.81 ± 0.02		30	30	0	0	yes				blast	ggggcuggccagacuuuuca	gagaguuuuuggcccuuc	ggggcuggccagacuuuucaagagagguuuuuggcccuuc	gi|307603376|gb|CM001000.1|:139960560..139
gi|307603378|gb|CM000998.1|_35753	2.1	0.81 ± 0.02		155	155	0	0	yes				blast	ucagcugccgugguucugggcuuu	agccuaagcugaugcagcuggaa	ucagcugccgugguucugggcuuuugggaucuaucuguagagccuaagcugaugcagcuggaa	gi|307603378|gb|CM000998.1|:53548886..5354
gi|307603370|gb|CM001006.1|_9322	2.1	0.81 ± 0.02		120	120	0	0	yes				blast	auggaggacugagaagguggagca	ccuuccauugguuaagaccuccaaca	auggaggacugagaagguggagcaguucuguaccgugcugccuuccauugguuaagaccuccaaca	gi|307603370|gb|CM001006.1|:96100553..9610
gi|307603373|gb|CM001003.1|_1890	2.1	0.81 ± 0.02		41	41	0	0	yes				blast	uggugcacaggaacaggccugc	uauccugcucugcagcaccuua	uauccugcucugcagcaccuuaguuagcuguggugcacaggaacaggccugc	gi|307603373|gb|CM001003.1|:80257540..8025
gi|307603366|gb|CM001010.1|_16907	2.1	0.81 ± 0.02		428	428	0	0	yes				blast	guuggucugagcaucuuccagg	gggaggaugucaggaugcagacug	gggaggaugucaggaugcagacugucccuuuccagugccucaguuggucugagcaucuuccagg	gi|307603366|gb|CM001010.1|:56497995..5649
gi|307603368|gb|CM001008.1|_13373	2.1	0.81 ± 0.02		44	44	0	0	yes				blast	uccugagccugaugcccccc	gggaaugcacagacagcugc	gggaaugcacagacagcugcaaaggccuccugagccugaugcccccc	gi|307603368|gb|CM001008.1|:102045453..102
gi|307603370|gb|CM001006.1|_8784	2.1	0.81 ± 0.02		49	49	0	0	yes				blast	uuugugucugcugggcucuccccu	ggagacucuuagcaggcucaaucu	uuugugucugcugggcucuccccucacuucccaugggagacucuuagcaggcucaaucu	gi|307603370|gb|CM001006.1|:34241671..3424
gi|307603381|gb|CM000995.1|_26015	2.1	0.81 ± 0.02		37	31	0	6	yes				blast	ccgguucaggggaacgugugcgga	cucguuccucucugcgccuggc	cucguuccucucugcgccuggccugcccccaccgccgguucaggggaacgugugcgga	gi|307603381|gb|CM000995.1|:151907377..151
gi|307603374|gb|CM001002.1|_47733	2.1	0.81 ± 0.02		149	149	0	0	yes				blast	ccgaagcagcggggcugga	cagccuggcugcgguuaug	ccgaagcagcggggcuggaguggccuucucccucuuccagccuggcugcgguuaug	gi|307603374|gb|CM001002.1|:102326377..102
gi|307603369|gb|CM001007.1|_12351	2.1	0.81 ± 0.02		28	28	0	0	yes				blast	ccggagcagacugcagug	cagcaucucugcuacgcgug	ccggagcagacugcagugcaggcggcagccaccagggccaacaccucgcagcaucucugcuacgcgug	gi|307603369|gb|CM001007.1|:79701322..7970
gi|307603371|gb|CM001005.1|_7892	2.1	0.81 ± 0.02		41	41	0	0	yes				blast	ugggagguggaaugacua	gagguuccagacccuccgcag	gagguuccagacccuccgcagggcaugaaguggcuuuugagcccauuccaugccuugggagguggaaugacua	gi|307603371|gb|CM001005.1|:59346688..5934
gi|307603376|gb|CM001000.1|_40730	2.1	0.81 ± 0.02		67	67	0	0	yes				blast	uagcauagggcuugacuuua	aagguggcccugugcugcu	uagcauagggcuugacuuuagagaaaauucuucugguggccaaagaccagaauccagccccagucccugaagguggcccugugcugcu	gi|307603376|gb|CM001000.1|:137835383..137
gi|307603376|gb|CM001000.1|_42179	2.1	0.81 ± 0.02		155	155	0	0	yes				blast	agauugcuguugcuggua	cuagacuggcucugg	agauugcuguugcugguauaggauucuauccaccaacucugugacauuccuggacuagacuggcucugg	gi|307603376|gb|CM001000.1|:106529004..106
gi|307603373|gb|CM001003.1|_365	2.1	0.81 ± 0.02		42	42	0	0	yes				blast	uggccaaguugaacuaaaaa	gaagguucaaagcuuggccaua	gaagguucaaagcuuggccauaacuuggaagaguuguuggccaaguugaacuaaaaa	gi|307603373|gb|CM001003.1|:57321701..5732
gi|307603376|gb|CM001000.1|_40658	2.1	0.81 ± 0.02		29	29	0	0	yes				blast	uguuuggaggaagaaacugagg	uccuuucuccaacugca	uguuuggaggaagaaacugaggcuucugagguaauguuuuagucaccugagcguccuuucuccaacugca	gi|307603376|gb|CM001000.1|:134618494..134
gi|307603377|gb|CM000999.1|_37503	2.1	0.81 ± 0.02		33	33	0	0	yes				blast	cuggcuguccucugcaggcu	uuugcuuggcagcgacug	uuugcuuggcagcgacugcagcgucucaaaggcaccugcucucuggcuguccucugcaggcu	gi|307603377|gb|CM000999.1|:86438237..8643
gi|307603372|gb|CM001004.1|_5925	2.1	0.81 ± 0.02		29	29	0	0	yes				blast	gacuggcccacucggaucucugc	aggcauccugggcccagucca	gacuggcccacucggaucucugccagucuaccuacccgcaggcauccugggcccagucca	gi|307603372|gb|CM001004.1|:107605202..107
gi|307603377|gb|CM000999.1|_37241	2.1	0.81 ± 0.02		29	29	0	0	yes				blast	ucuccuugccugaccucugucu	acagagggagggugagggucc	acagagggagggugaggguccugugcaugauccagggucuccuugccugaccucugucu	gi|307603377|gb|CM000999.1|:52129768..5212
gi|307603372|gb|CM001004.1|_3040	2.1	0.81 ± 0.02		73	73	0	0	yes				blast	ugccugcucuucuguucucugga	caagagcaaaugagcaucucug	ugccugcucuucuguucucuggagaacaagaagcacuuuuucugucauccaagagcaaaugagcaucucug	gi|307603372|gb|CM001004.1|:60062234..6006
gi|307603379|gb|CM000997.1|_31137	2.1	0.81 ± 0.02		36	36	0	0	yes				blast	guguuagguaagccaagaucugg	ggaaguggcuuuggggcgggaccaac	ggaaguggcuuuggggcgggaccaacuguguucucuucugagcaagguguguuagguaagccaagaucugg	gi|307603379|gb|CM000997.1|:45310012..4531
gi|307603370|gb|CM001006.1|_9120	2.1	0.81 ± 0.02		2427	2386	0	41	yes				blast	acaucaacguggagcugagucu	agaaaaggcuccggguugac	acaucaacguggagcugagucuucuugggaagaagaagaaaaggcuccggguugac	gi|307603370|gb|CM001006.1|:66975134..6697
gi|307603379|gb|CM000997.1|_31534	2.1	0.81 ± 0.02		76	76	0	0	yes				blast	uccuucacuagcugagaccuga	aacaacucugcuaguggagaga	uccuucacuagcugagaccugagcuuggccagugugcugcaacaacucugcuaguggagaga	gi|307603379|gb|CM000997.1|:106019735..106
gi|307603380|gb|CM000996.1|_29652	2.1	0.81 ± 0.02		806	806	0	0	yes				blast	ucaagguccgcugugaacacgg	gcauucaccgcgugccuuaauu	gcauucaccgcgugccuuaauuguaugacauuaaaucaagguccgcugugaacacgg	gi|307603380|gb|CM000996.1|:17695684..1769
gi|307603371|gb|CM001005.1|_7659	2.1	0.81 ± 0.02		34	34	0	0	yes				blast	acagggcugcugcaagggaga	ucccuccuacagcugcccugucc	acagggcugcugcaagggagacaggcuuuguuagccuccauguuuucccuccuacagcugcccugucc	gi|307603371|gb|CM001005.1|:27824350..2782
gi|307603364|gb|CM001012.1|_20920	2.1	0.81 ± 0.02		64	64	0	0	yes				blast	uauagcgggaaagaauaccau	agcauucuuccccacuaauu	agcauucuuccccacuaauuacuucauuaguauagcgggaaagaauaccau	gi|307603364|gb|CM001012.1|:14558271..1455
gi|307603378|gb|CM000998.1|_36250	2.1	0.81 ± 0.02		31	31	0	0	yes				blast	acgcucuggcuuugcaggaagca	cuuuuugcauaggugcug	cuuuuugcauaggugcugggagucggcuccucacgcucuggcuuugcaggaagca	gi|307603378|gb|CM000998.1|:117538295..117
gi|307603373|gb|CM001003.1|_985	2.1	0.81 ± 0.02		32	32	0	0	yes				blast	ugcuuucccucugucucuagga	uauaggagacagagagaaaacggg	uauaggagacagagagaaaacgggacgucuccugcuuucccucugucucuagga	gi|307603373|gb|CM001003.1|:99605983..9960
gi|307603366|gb|CM001010.1|_17982	2.1	0.81 ± 0.02		32	32	0	0	yes				blast	aggcuagcuggacuggcagca	cagccaugugccagcuaagcaccc	cagccaugugccagcuaagcacccacaguuccaaggcuagcuggacuggcagca	gi|307603366|gb|CM001010.1|:57033257..5703
gi|307603377|gb|CM000999.1|_36867	2.1	0.81 ± 0.02		40	40	0	0	yes				blast	gaguuugggugaugcugccaca	uggcacgucucccaacuuccu	uggcacgucucccaacuuccuaggccgcugggaguuugggugaugcugccaca	gi|307603377|gb|CM000999.1|:13628261..1362
gi|307603381|gb|CM000995.1|_25424	2.1	0.81 ± 0.02		156	156	0	0	yes				blast	agauucgcucggagacgcgga	cgcgucaggaaucgcg	agauucgcucggagacgcggacagaacccguucugucgcgucaggaaucgcg	gi|307603381|gb|CM000995.1|:90894259..9089
gi|307603381|gb|CM000995.1|_27080	2.1	0.81 ± 0.02		34	32	0	2	yes				blast	ccaacauggcagaggcug	cagccucuggcaugcuggc	cagccucuggcaugcuggcuccuuccaagcagcuguuuuggguuugaaauuccaacauggcagaggcug	gi|307603381|gb|CM000995.1|:63936034..6393
gi|307603373|gb|CM001003.1|_724	2.1	0.81 ± 0.02		42	42	0	0	yes				blast	uuugguuccucugaccuuuugcug	gccaggucucuggagccuuugc	uuugguuccucugaccuuuugcugugacccuucuauauggccaggucucuggagccuuugc	gi|307603373|gb|CM001003.1|:80604649..8060
gi|307603381|gb|CM000995.1|_28078	2.1	0.81 ± 0.02		1131	1120	0	11	yes				blast	cucucugcagucacuguccagg	uucugccugccugagagaacuucc	uucugccugccugagagaacuucccacacagaaagacauggagucucucugcagucacuguccagg	gi|307603381|gb|CM000995.1|:165905991..165
gi|307603381|gb|CM000995.1|_25926	2.1	0.81 ± 0.02		31	31	0	0	yes				blast	aguggugggugugucagaa	cuacacacccccugccgguac	aguggugggugugucagaacuaaccuacacacccccugccgguac	gi|307603381|gb|CM000995.1|:138111853..138
gi|307603381|gb|CM000995.1|_27326	2.1	0.81 ± 0.02		228	228	0	0	yes				blast	agaaaucgggcagcucgag	cgggccugccccuccucccucc	cgggccugcccccuccucccucccucucugggguggccucccagauggugagaaaucgggcagcucgag	gi|307603381|gb|CM000995.1|:93017169..9301
gi|307603376|gb|CM001000.1|_42350	2.1	0.81 ± 0.02		252	252	0	0	yes				blast	aguggagcucuaagaguc	ugagcucugucgcuga	ugagcucugucgcugauuggacagcggcgaagucgcuccugcagaccaucgcgucuggccgcaguggagcucuaagaguc	gi|307603376|gb|CM001000.1|:118266198..118
gi|307603373|gb|CM001003.1|_483	2.1	0.81 ± 0.02		107	107	0	0	yes				blast	ugagauuaccugcuggcgguuca	ugcccagccacgggaaucuccua	ugcccagccacgggaaucuccuagagagaaaaugaucuaugagauuaccugcuggcgguuca	gi|307603373|gb|CM001003.1|:68430725..6843
gi|307603379|gb|CM000997.1|_31896	2.1	0.81 ± 0.02		907	901	0	6	yes				blast	uggcccuuaucgaagcugcagca	gucaaaaagguggcccagag	uggcccuuaucgaagcugcagcagcuucuacauagcugcugugucaaaaagguggcccagag	gi|307603379|gb|CM000997.1|:131865381..131
gi|307603376|gb|CM001000.1|_40674	2.1	0.81 ± 0.02		137	137	0	0	yes				blast	gagggaugagagcggcgacc	ggggugcuaagacaucccaacu	gagggaugagagcggcgaccugggcccagggccgaagcagcgcgaggcuaggggaguaggggugcuaagacaucccaacu	gi|307603376|gb|CM001000.1|:134889812..134
gi|307603369|gb|CM001007.1|_12220	2.1	0.81 ± 0.02		30	30	0	0	yes				blast	gcggcauguucugcccugugga	ggcuccggcagucccaagccgcgg	ggcuccggcagucccaagccgcggcagaaauucugcggcauguucugcccugugga	gi|307603369|gb|CM001007.1|:67529495..6752
gi|307603366|gb|CM001010.1|_16864	2.1	0.81 ± 0.02		265	265	0	0	yes				blast	acucagagguguggaaacag	ucuucuuuugcuucucuguga	acucagagguguggaaacagagguggaaacacauuccauccuuagcucuucuuuugcuucucuguga	gi|307603366|gb|CM001010.1|:54838295..5483
gi|307603366|gb|CM001010.1|_17980	2.0	0.81 ± 0.02		428	428	0	0	yes				blast	guuggucugagcaucuuccagg	gggaggaugucaggaugcagacug	gggaggaugucaggaugcagacugucccuuuccagugccucaguuggucugagcaucuuccagg	gi|307603366|gb|CM001010.1|:56889067..5688
gi|307603365|gb|CM001011.1|_19407	2.0	0.81 ± 0.02		48	48	0	0	yes				blast	cagcugcugcagggagcucaccc	guuugagagccuuccagcaguaagga	cagcugcugcagggagcucacccgcuagggcgugguggaguuugagagccuuccagcaguaagga	gi|307603365|gb|CM001011.1|:45427917..4542
gi|307603366|gb|CM001010.1|_18140	2.0	0.81 ± 0.02		494	494	0	0	yes				blast	uggccuguacucucacacuaa	agugugacauggcagucgccaug	agugugacauggcagucgccauggccuucccccaggacauggccuguacucucacacuaa	gi|307603366|gb|CM001010.1|:80594250..8059
gi|307603380|gb|CM000996.1|_28852	2.0	0.81 ± 0.02		103	103	0	0	yes				blast	gauuagaccucaggcagaauuga	aauucugccugaguuuggugcg	gauuagaccucaggcagaauugauacuggucaauucugccugaguuuggugcg	gi|307603380|gb|CM000996.1|:89000743..8900

gi|307603372|gb|CM001004.1|_4751	2.0	0.81 ± 0.02		270	270	0	0	yes				blast	acaacagggcagcagaga	ggagcugcacacgccucuugugc	acaacagggcagcagagacugggagcugcacacgccucuugugc	gi|307603372|gb|CM001004.1|:48651975..4865
gi|307603376|gb|CM001000.1|_39585	2.0	0.81 ± 0.02		115	115	0	0	yes				blast	ccaguccucccuucucuccacag	gggaggaggggggaggaccucag	gggaggaggggggaggaccucaguguuucuuccucaucuccaguccucccuucucuccacag	gi|307603376|gb|CM001000.1|:19933688..1993
gi|307603373|gb|CM001003.1|_807	2.0	0.81 ± 0.02		163	163	0	0	yes				blast	ugcccucaggaugugagccuau	ucgcucauauucugagggccaa	ucgcucauauucugagggccaaucauauguugaccuuugcccucaggaugugagccuau	gi|307603373|gb|CM001003.1|:82843530..8284
gi|307603371|gb|CM001005.1|_6824	2.0	0.81 ± 0.02		31	31	0	0	yes				blast	cccagugcuggcaggacu	ccuugcuguguggcc	cccagugcuggcaggacucuuuccuugcuguguggcc	gi|307603371|gb|CM001005.1|:81646113..8164
gi|307603368|gb|CM001008.1|_13471	2.0	0.81 ± 0.02		30	30	0	0	yes				blast	aagugcagaucacuggggcuuug	ucgcuccaguucugcucugcu	aagugcagaucacuggggcuuugcucaucgcuccaguucugcucugcu	gi|307603368|gb|CM001008.1|:12734883..1273
gi|307603363|gb|CM001013.1|_49101	2.0	0.81 ± 0.02		1782	1782	0	0	yes				blast	gucccuguucgggcgcca	gcguaagaaagggucuc	gucccuguucgggcgccaucugccacaggcccucuggucccugugccugcguaagaaagggucuc	gi|307603363|gb|CM001013.1|:27328432..2732
gi|307603374|gb|CM001002.1|_46681	2.0	0.81 ± 0.02		72	72	0	0	yes				blast	gugggugggaauaguauaacaau	uguuauaguauuccaccuuccc	gugggugggaauaguauaacaauagaucaugcauuguuauaguauuccaccuuccc	gi|307603374|gb|CM001002.1|:4455949..4456
gi|307603368|gb|CM001008.1|_13815	2.0	0.81 ± 0.02		37	37	0	0	yes				blast	gaaggaccgaggacaugggggg	uuccaucauauggguucugagg	gaaggaccgaggacaugggggggaguuaguucuuucucuuuccaucauauggguucugagg	gi|307603368|gb|CM001008.1|:75393366..7539
gi|307603378|gb|CM000998.1|_36369	2.0	0.81 ± 0.02		29	29	0	0	yes				blast	ugggaaaagacacuguca	aucugagguccuccccuc	ugggaaaagacacugucaccaagcccggugaucugagguccuccccuc	gi|307603378|gb|CM000998.1|:124529050..124
gi|307603380|gb|CM000996.1|_30546	2.0	0.81 ± 0.02		29	29	0	0	yes				blast	uugcugggacacacaugg	uggguggcucagcaggu	uggguggcucagcagguaaaggcauuggcugccaagccuaaugaccugcacuugauugcugggacacacaugg	gi|307603380|gb|CM000996.1|:119456862..119
gi|307603379|gb|CM000997.1|_31014	2.0	0.81 ± 0.02		28	28	0	0	yes				blast	ccagggugugu aggaacaugaa	uuugucugcaggccuggcc	ccagggugugu aggaacaugaacaucccuuugucugcaggccuggcc	gi|307603379|gb|CM000997.1|:33750849..3375
gi|307603372|gb|CM001004.1|_3623	2.0	0.81 ± 0.02		24243	18613	1725	3905	yes				blast	ggagggaacgcagucugagugga	cuugacugcagcugccucc	cuugacugcagcugccuccugccauugaugaucguucuucccuccuuugggagggugagagggagggaacgcagucugagugga	gi|307603372|gb|CM001004.1|:87262259..8726
gi|307603372|gb|CM001004.1|_5984	2.0	0.81 ± 0.02		40	40	0	0	yes				blast	uggacuuagggacagagca	cucuggacuugacugucccuc	cucuggacuugacuguccccuccaguggacuuagggacagagca	gi|307603372|gb|CM001004.1|:114657167..114
gi|307603379|gb|CM000997.1|_30895	2.0	0.81 ± 0.02		47	47	0	0	yes				blast	gcugaaggcugcauggug	ccuaguuaagccugaucuuuc	ccuaguuaagccugaucuuucuguuugugaaaagagcugagcagagcugaaggcugcauggug	gi|307603379|gb|CM000997.1|:11506782..1150
gi|307603376|gb|CM001000.1|_40393	2.0	0.81 ± 0.02		33	33	0	0	yes				blast	cugaguagagcccuccugug	cagcagggccuggcaaguc	cagcagggccuggcaagucacucugcaucaucugcagaagccaucaccacuacaggacugaguagagcccuccugug	gi|307603376|gb|CM001000.1|:108936982..108
gi|307603382|gb|CM000994.1|_21628	2.0	0.81 ± 0.02		30	30	0	0	yes				blast	cugggccuuggguaggagacuu	gacccaacaggaacagag	cugggccuuggguaggagacuugaggaugcagaucugguguugccuuccagcaccagaguuccgggacccaacaggaacagag	gi|307603382|gb|CM000994.1|:44176132..4417
gi|307603374|gb|CM001002.1|_47169	2.0	0.81 ± 0.02		31	31	0	0	yes				blast	gcuggagagguggcucagg	ugagcaccuacaucaggcga	gcuggagagguggcucagggaaagagcagcuccugcccuuacagaagcucccaucuugguucugagcaccuacaucaggcga	gi|307603374|gb|CM001002.1|:55070436..5507
gi|307603365|gb|CM001011.1|_18969	2.0	0.81 ± 0.02		150	143	7	0	yes				blast	cuguccguacgcccguag	gcgagguuuacaaguaaac	gcgagguuuacaaguaaacuaugggucgccgaagcuauaggucccgcuccgcagugcuguccguacgcccguag	gi|307603365|gb|CM001011.1|:80666853..8066
gi|307603365|gb|CM001011.1|_18895	2.0	0.81 ± 0.02		143	143	0	0	yes				blast	accgcaggaaggccggacgccc	gucaccgccuuccucgggac	gucaccgccuuccucgggaccgcuauuuccuccgccaccgcaggaaggccggacgccc	gi|307603365|gb|CM001011.1|:70632049..7063
gi|307603364|gb|CM001012.1|_21151	2.0	0.81 ± 0.02		37	37	0	0	yes				blast	gucuuccuggcgcauuuguu	caaaugggcccgacuc	gucuuccuggcgcauuuguuaaguguucaaaugggcccgacuc	gi|307603364|gb|CM001012.1|:44188559..4418
gi|307603379|gb|CM000997.1|_33512	2.0	0.81 ± 0.02		29	29	0	0	yes				blast	uuggagcggcaggggcca	gcccaggcuucagagc	gcccaggcuucagagcaguucaagugggggacgcccagcaaagcuuggagcggcaggggcca	gi|307603379|gb|CM000997.1|:135798829..135
gi|307603364|gb|CM001012.1|_20976	2.0	0.81 ± 0.02		33	33	0	0	yes				blast	ugcuugcaaccucucugggc	ccagagcggaugugaggaca	ccagagcggaugugaggacagaucgcuaccugugcuugcaaccucucugggc	gi|307603364|gb|CM001012.1|:24375699..2437
gi|307603371|gb|CM001005.1|_6985	2.0	0.81 ± 0.02		31	31	0	0	yes				blast	acaaaccagugagcuccaagu	auggagcucacugguuagcca	auggagcucacugguuagccagucuagacaaaccagugagcuccaagu	gi|307603371|gb|CM001005.1|:90578207..9057
gi|307603377|gb|CM000999.1|_39119	2.0	0.81 ± 0.02		32	32	0	0	yes				blast	gcagcauucaggucucuggguccu	guccuuaaguucauagucuguau	gcagcauucaggucucugggucccuggauguccuuggugcacacuccaaggacucccucguccuuaaguucauagucuguau	gi|307603377|gb|CM000999.1|:125115933..125
gi|307603377|gb|CM000999.1|_37165	2.0	0.81 ± 0.02		28	28	0	0	yes				blast	gggguggacccaguggcca	guggaccuggguaccccag	guggaccuggguaccccaguacuguccaacucuaguggacaguccagcucaucucaaggggugggacccaguggcca	gi|307603377|gb|CM000999.1|:47855531..4785
gi|307603374|gb|CM001002.1|_47426	2.0	0.81 ± 0.02		29	29	0	0	yes				blast	uagggcugugugcauggc	caugaguacccguagcccccca	caugaguacccguagccccccacccccacccccacccccaccccccaccggagggggauuggacuagggcugugugcauggc	gi|307603374|gb|CM001002.1|:72018241..7201
gi|307603376|gb|CM001000.1|_39437	2.0	0.81 ± 0.02		76	76	0	0	yes				blast	ucuucucuuuccagucaucagc	uggugccuggauuggaggaug	uggugccuggauuggaggaugagagaauauuuauuaucagaucgcucuucucuuuccagucaucagc	gi|307603376|gb|CM001000.1|:4869566..4869
gi|307603381|gb|CM000995.1|_26413	2.0	0.81 ± 0.02		243	243	0	0	yes				blast	cuuuguguaggacuucaga	ugguucuguacugau	ugguucuguacugaugaagccacagcgugaaagcuuggcuuuaucuuuguguaggacuucaga	gi|307603381|gb|CM000995.1|:179770032..179
gi|307603375|gb|CM001001.1|_43726	2.0	0.81 ± 0.02		411	301	110	0	yes				blast	gcuggaggaugaaguaagg	ucacauugcugacagugc	gcuggaggaugaaguaaggagugauccaucggcuaaguguuuucccacugugcugacacucacauugcugacagugc	gi|307603375|gb|CM001001.1|:112363199..112
gi|307603365|gb|CM001011.1|_18604	2.0	0.81 ± 0.02		69	69	0	0	yes				blast	accagaggaguaggagag	cuucugccccucauguuu	accagaggaguaggagagucugguuacuggugaacaggggucucuucugccccucauguuu	gi|307603365|gb|CM001011.1|:38415718..3841
gi|307603376|gb|CM001000.1|_42822	2.0	0.81 ± 0.02		34	34	0	0	yes				blast	ucgugacaagcagggcaucucu	ugauguccuccuugugagaca	ugauguccuccuugugagacaagcugucgugacaagcagggcaucucu	gi|307603376|gb|CM001000.1|:151775188..151
gi|307603378|gb|CM000998.1|_36548	2.0	0.81 ± 0.02		37	37	0	0	yes				blast	aggccaggcacucugaugg	cccagagucccugcacuga	cccagagucccugcacugaacucugcuggccuuacuccagcacaaggccaggcacucugaugg	gi|307603378|gb|CM000998.1|:136904451..136
gi|307603370|gb|CM001006.1|_10026	2.0	0.81 ± 0.02		44	44	0	0	yes				blast	augcuagaugggagaaucc	aagcugccaggcugc	aagcugccaggcugccaugcagaacugcucuugcaaacaggcaggcaugcuagaugggagaaucc	gi|307603370|gb|CM001006.1|:48372494..4837
gi|307603371|gb|CM001005.1|_8089	2.0	0.81 ± 0.02		33	33	0	0	yes				blast	gaagacuuacccugguguguc	agcacagggauaagguggcac	gaagacuuacccuggugugucccccagcacagggauaagguggcac	gi|307603371|gb|CM001005.1|:84888458..8488
gi|307603379|gb|CM000997.1|_32214	2.0	0.81 ± 0.02		30	30	0	0	yes				blast	cuguuugcuagcugggga	cucagcuacaggcaaaa	cuguuugcuagcuggggaaugugcuucucagcuacaggcaaaa	gi|307603379|gb|CM000997.1|:154282091..154
gi|307603377|gb|CM000999.1|_39261	2.0	0.81 ± 0.02		30	30	0	0	yes				blast	cuccagaggacacagagcug	gcucauauacguggacgugguuuu	cuccagaggacacagagcugugggggggugguggugugugaaaguucccaaggcucauauacguggacgugguuuu	gi|307603377|gb|CM000999.1|:136761856..136
gi|307603377|gb|CM000999.1|_38943	2.0	0.81 ± 0.02		39	39	0	0	yes				blast	gugagcgucuguccucagagug	cacacugcggacagaugaacag	gugagcgucuguccucagagugcugaggcacacacugcggacagaugaacag	gi|307603377|gb|CM000999.1|:115262952..115
gi|307603376|gb|CM001000.1|_40128	2.0	0.81 ± 0.02		55	55	0	0	yes				blast	cugguaggggucugaugcu	caugauauaccugccagua	cugguaggggucugaugcuugcccuccuuagaggcacagaagagcagcaugauauaccugccagua	gi|307603376|gb|CM001000.1|:83018191..8301
gi|307603377|gb|CM000999.1|_37175	2.0	0.81 ± 0.02		175	175	0	0	yes				blast	ucacaccugcggggcugagacga	cucuacagcucaaaggugaacggagc	cucuacagcucaaaggugaacggagcucaacaccuagugcucacaccugcggggcugagacga	gi|307603377|gb|CM000999.1|:47903218..4790
gi|307603372|gb|CM001004.1|_4325	2.0	0.81 ± 0.02		232	232	0	0	yes				blast	ucccaggcugagcaugcacuau	uguggaugcuccaccagggagg	uguggaugcuccaccagggagggcauucuucccauguaauaggccacccucccaggcugagcaugcacuau	gi|307603372|gb|CM001004.1|:119691385..119
gi|307603381|gb|CM000995.1|_24980	2.0	0.81 ± 0.02		71	71	0	0	yes				blast	ucucuaucccuggccugcucu	ccuagguuuggggauacagagc	ucucuaucccuggccugcucuccugugggggacuuggagccuagguuuggggauacagagc	gi|307603381|gb|CM000995.1|:32039915..3203
gi|307603378|gb|CM000998.1|_35194	2.0	0.81 ± 0.02		42	42	0	0	yes				blast	gcugacugggugugccug	ggaagaacggacaguca	gcugacugggugugccugugacuuccaggccgucuggucgcaggaagaacggacaguca	gi|307603378|gb|CM000998.1|:139233344..139
gi|307603381|gb|CM000995.1|_24542	2.0	0.81 ± 0.02		216	216	0	0	yes				blast	ugugaauuuguggccagccugg	aggacugccacggauacacaua	ugugaauuuguggccagccuggcuacaaagugaguccaggacugccacggauacacaua	gi|307603381|gb|CM000995.1|:9162418..9162
gi|307603381|gb|CM000995.1|_26595	2.0	0.81 ± 0.02		54	54	0	0	yes				blast	aaugcccaccauacucuguugc	agcagggucugggagcggccgguu	aaugcccaccauacucuguugccucaaaaugcagcagggucugggagcggccgguu	gi|307603381|gb|CM000995.1|:13975844..1397
gi|307603371|gb|CM001005.1|_6977	2.0	0.81 ± 0.02		44	44	0	0	yes				blast	uugcucugccaagguuugcuugc	aggcuugcuuuggcaugguguca	aggcuugcuuuggcauggugucauugcuucauuauguugcucugccaagguuugcuugc	gi|307603371|gb|CM001005.1|:90170428..9017
gi|307603378|gb|CM000998.1|_36508	2.0	0.81 ± 0.02		33	33	0	0	yes				blast	guucacuuucggcaacucugug	cacuugccccaaguuuuggcag	cacuugccccaaguuuuggcagcucguucacuuucggcaacucugug	gi|307603378|gb|CM000998.1|:135857285..135
gi|307603380|gb|CM000996.1|_29442	2.0	0.81 ± 0.02		30	30	0	0	yes				blast	cuaauuugaggggagcuucu	aaaucuccaucaccugggc	cuaauuugaggggagcuucuucgcgcugcucagcccucucugaggagcacgggaagaaaucuccaucaccugggc	gi|307603380|gb|CM000996.1|:135100872..135
gi|307603378|gb|CM000998.1|_34102	1.9	0.28 ± 0.02		87	87	0	0	yes				blast	cucacugggaaguggccuccgag	uucacagaucugccuuuacccaggagga	cucacugggaaguggccuccgagcaacaagguuuucagccuuuuaaaaccauguagcuuucacagaucugccuuuacccaggagga	gi|307603378|gb|CM000998.1|:31482914..3148
gi|307603374|gb|CM001002.1|_47522	1.9	0.28 ± 0.02		34	34	0	0	yes				blast	ccagucuaggaaagaggg	uuuucuuccuagauuagga	uuuucuuccuagauuaggaucuucaacuggcaugcucaggacugaucccagucuaggaaagaggg	gi|307603374|gb|CM001002.1|:78351097..7835
gi|307603381|gb|CM000995.1|_26865	1.9	0.28 ± 0.02		33	33	0	0	yes				blast	gcaguaggagccugagagugcu	uccucuggcugccaaagucucugcug	gcaguaggagccugagagugcucuguuuccccgaguccucuggcugccaaagucucugcug	gi|307603381|gb|CM000995.1|:34707886..3470
gi|307603371|gb|CM001005.1|_7146	1.9	0.28 ± 0.02		55	55	0	0	yes				blast	ccuccucagcaugucuucagaacu	uucuaggaugcuuuggcuugg	ccuccucagcaugucuucagaacucuggagacuguaucucagaguucuaggaugcuuuggcuugg	gi|307603371|gb|CM001005.1|:110079698..110
gi|307603372|gb|CM001004.1|_6161	1.9	0.28 ± 0.02		240	240	0	0	yes				blast	acucacugugcccucucuguc	cagagaguauguacgagugu	acucacugugcccucucugucccaggucugcaccuccugcaucccuaacagagaguauguacgagugu	gi|307603372|gb|CM001004.1|:120482154..120
gi|307603376|gb|CM001000.1|_40420	1.9	0.28 ± 0.02		30	30	0	0	yes				blast	uguucgagccuguuccaaggga	cgaaaacaguuuggaugag	uguucgagccuguuccaagggaugguaagugguccgaaaacaguuuggaugag	gi|307603376|gb|CM001000.1|:114656171..114
gi|307603374|gb|CM001002.1|_47085	1.9	0.28 ± 0.02		57	57	0	0	yes				blast	gacacugacugucugacugugg	gucucugauugucagugcag	gacacugacugucugacuguggacaaaucagccguccgucucugauugucagugcag	gi|307603374|gb|CM001002.1|:48550714..4855
gi|307603373|gb|CM001003.1|_2318	1.9	0.28 ± 0.02		32	32	0	0	yes				blast	caugauguauagaggauggg	auuccucaaugaauuugga	auuccucaaugaauuuggagggggaauccuggaaaugugccagaauccauccuccaugauguauagaggauggg	gi|307603373|gb|CM001003.1|:126619784..126
gi|307603376|gb|CM001000.1|_41109	1.9	0.28 ± 0.02		32	32	0	0	yes				blast	gcugucgggcuccugcga	acagaagccugacuggcgc	gcugucgggcuccugcgacugaaaucaaguuacagcaaguaacugccacagaagccugacuggcgc	gi|307603376|gb|CM001000.1|:19509911..1950
gi|307603378|gb|CM000998.1|_36262	1.9	0.28 ± 0.02		60	60	0	0	yes				blast	uuugcucuucucuccuuucagu	ugaaauggucagucaaagccgaagg	ugaaauggucagucaaagccgaaggugaggacugccacaaggcuguucucagccuuugcucuucucuccuuucagu	gi|307603378|gb|CM000998.1|:118651439..118
gi|307603371|gb|CM001005.1|_6703	1.9	0.28 ± 0.02		35	35	0	0	yes				blast	aggccccggugcacgcugug	cagaggcucccccucu	cagaggcucccccucugucuaaccguugguuagacaaaggccccggugcacgcugug	gi|307603371|gb|CM001005.1|:72634218..7263
gi|307603374|gb|CM001002.1|_47159	1.9	0.28 ± 0.02		258	258	0	0	yes				blast	agcaacucaggccagccacu	gggcaggucugaguucucu	agcaacucaggccagccacucaucugugcacugauaaaauaaagauguggcugcuguaacagaugggcaggucugaguucucu	gi|307603374|gb|CM001002.1|:53779145..5377
gi|307603375|gb|CM001001.1|_43539	1.9	0.28 ± 0.02		39	39	0	0	yes				blast	gcacagcucuggcagggg	ccugcucagccacgcga	ccugcucagccacgcgacucccaaacacacucaaaguuugcguagaaaaagcacagcucuggcagggg	gi|307603375|gb|CM001001.1|:96492463..9649
gi|307603374|gb|CM001002.1|_46047	1.9	0.28 ± 0.02		55	41	14	0	yes				blast	uccagucaggccgagccgcucc	agcggccucggcguacacagc	agcggccucggcguacacagcccgcgcuccucccucgccuccucccucuuucuccucccucagggcuccagucaggccgagccgcucc	gi|307603374|gb|CM001002.1|:72122740..7212
gi|307603377|gb|CM000999.1|_37238	1.9	0.28 ± 0.02		39	39	0	0	yes				blast	cuuggacacaguagugauga	cucauggauguaaaacgagcc	cuuggacacaguagugaugacagcaguucucauggauguaaaacgagcc	gi|307603377|gb|CM000999.1|:51538892..5153
gi|307603382|gb|CM000994.1|_22866	1.9	0.28 ± 0.02		36	36	0	0	yes				blast	auuuguggcuggcuuugauaacg	ugcaggaagccgcuccggaugu	ugcaggaagccgcuccggauguuugcuucuuuacuuguggcauuuggcuggcuuugauaacg	gi|307603382|gb|CM000994.1|:4486515..4486
gi|307603378|gb|CM000998.1|_36245	1.9	0.28 ± 0.02		29	29	0	0	yes				blast	gagcagcuggcuacccug	ggcacugccggugugcucaa	gagcagcuggcuacccugaggcagacacgauagggucucugcugcuguacaacacugcaccaggcacugccggugugcucaa	gi|307603378|gb|CM000998.1|:116907228..116
gi|307603380|gb|CM000996.1|_29407	1.9	0.28 ± 0.02		30	30	0	0	yes				blast	ugucaaaggccucuacaaga	uuugaaguuuuugucgau	uuugaaguuuuugucgaugcuccucugcaugucugugagcagagggaugucaaaggccucuacaaga	gi|307603380|gb|CM000996.1|:131246098..131
gi|307603363|gb|CM001013.1|_48594	1.9	0.28 ± 0.02		32	32	0	0	yes				blast	augacaacagucucagggcuga	aggccacucagcugaccuuugugcagc	augacaacagucucagggcugaaacacuuucaggccacucagcugaccuuugugcagc	gi|307603363|gb|CM001013.1|:99852481..9985
gi|307603380|gb|CM000996.1|_30684	1.9	0.28 ± 0.02		53	53	0	0	yes				blast	uauaaggacgagaagcucacugua	cagggauccuucucucucagcucu	cagggauccuucucucucagcucuaauauauaaggacgagaagcucacugua	gi|307603380|gb|CM000996.1|:135189299..135
gi|307603371|gb|CM001005.1|_6356	1.9	0.28 ± 0.02		70	70	0	0	yes				blast	aucccgaacgagcccca	cucacucaagucucgggaacuuu	aucccgaacgagcccccaaaugaaagaccccugaagggagacccucacucaagucucgggaacuuu	gi|307603371|gb|CM001005.1|:19724455..1972
gi|307603382|gb|CM000994.1|_24165	1.9	0.28 ± 0.02		29	29	0	0	yes				blast	auuucggugcaucaucuguuu	acagccaugcucugauggcu	auuucggugcaucaucuguuuccauaaguugggcacagccaugcucugauggcu	gi|307603382|gb|CM000994.1|:172216609..172
gi|307603379|gb|CM000997.1|_31807	1.9	0.28 ± 0.02		147	147	0	0	yes				blast	auccuggggagaaaguggca	ccuccccuccucccuguaaaucu	ccuccccuccucccuguaaaucuuucucuacugaugggaagauccuggggagaaaguggca	gi|307603379|gb|CM000997.1|:128119842..128
gi|307603381|gb|CM000995.1|_27289	1.9	0.28 ± 0.02		37	37	0	0	yes				blast	cugagcuucugccaucaug	uggugcaggcucaaggg	cugagcuucugccaucauggcgugagagccuggugcaggcucaaggg	gi|307603381|gb|CM000995.1|:91745166..9174
gi|307603376|gb|CM001000.1|_39427	1.9	0.28 ± 0.02		36	36	0	0	yes				blast	auggcugccugggcuccaccc	cuggaguccucggagaggcauua	cuggaguccucggagaggcauuagggacuggauccuucuuccccuaauggcugccugggcuccaccc	gi|307603376|gb|CM001000.1|:4692835..4692
gi|307603369|gb|CM001007.1|_11439	1.9	0.28 ± 0.02		33	33	0	0	yes				blast	uacuucucucgcgcugcuaccguu	caagagugguguucagagagagggaga	caagagugguguucagagagagggagagaaagcuggcaucuacuucucucgcgcugcuaccguu	gi|307603369|gb|CM001007.1|:98569261..9856
gi|307603368|gb|CM001008.1|_13789	1.9	0.28 ± 0.02		30	30	0	0	yes				blast	caucagcacugcuacccucaag	ugggguuuuauacagcagcacuggguu	caucagcacugcuacccucaagauggaggggcagacaucuuuaugcugggguuuuauacagcagcacuggguu	gi|307603368|gb|CM001008.1|:72638254..7263
gi|307603376|gb|CM001000.1|_40657	1.9	0.28 ± 0.02		29	29	0	0	yes				blast	uguuuggaggaagaaacugagg	gcaggcucacuccucuagagaca	gcaggcucacuccucuagagacagauuaguguuuggaggaagaaacugagg	gi|307603376|gb|CM001000.1|:134618465..134
gi|307603363|gb|CM001013.1|_48139	1.9	0.28 ± 0.02		189	189	0	0	yes				blast	auuagcuguuggcugugugagg	ucaccagccacaauuaaugc	ucaccagccacaauuaaugcugguuuuugaguuacuuguggcauuagcuguuggcugugugagg	gi|307603363|gb|CM001013.1|:12721510..1272
gi|307603368|gb|CM001008.1|_14013	1.9	0.28 ± 0.02		32	32	0	0	yes				blast	aauccucugcucuguaagcuuc	agcugauggagcaggggaucuc	aauccucugcucuguaagcuucucgaagucaaaauuagagucugagcugauggagcaggggaucuc	gi|307603368|gb|CM001008.1|:81685876..8168
gi|307603379|gb|CM000997.1|_30873	1.9	0.28 ± 0.02		35	35	0	0	yes				blast	gaaaacugugcagcagaggg	cucugcagaugcgcaggaauucau	cucugcagaugcgcaggaauucauaaugagaugaaaauacaaucaaaggaaaacugugcagcagaggg	gi|307603379|gb|CM000997.1|:9111950..9112
gi|307603370|gb|CM001006.1|_9645	1.9	0.28 ± 0.02		59	59	0	0	yes				blast	uucuugucccuccgucug	gacauacagggacucagacc	uucuugucccuccgucuguagugcucgauggcuuaggccaagguugcagcauuuucagacauacagggacucagacc	gi|307603370|gb|CM001006.1|:21825979..2182
gi|307603376|gb|CM001000.1|_42016	1.9	0.28 ± 0.02		45	45	0	0	yes				blast	uuuugugguagaucgagcuggcac	gccucuccuuucugcaguacaaggcc	gccucuccuuucugcaguacaaggccacagauuuugugguagaucgagcuggcac	gi|307603376|gb|CM001000.1|:87243762..8724
gi|307603381|gb|CM000995.1|_28125	1.9	0.28 ± 0.02		260	260	0	0	yes				blast	aggcgcacuggacaucug	gaucacaguggcgcccac	aggcgcacuggacaucugcaggccuccccuugccgaucacaguggcgcccac	gi|307603381|gb|CM000995.1|:170227065..170
gi|307603378|gb|CM000998.1|_34700	1.9	0.28 ± 0.02		37	37	0	0	yes				blast	ucaagugccgugguaugaaag	cucucaccauccgcacugagg	ucaagugccgugguaugaaagagacccauaugccuguguacugcagcugcgcaggggacuuuacucucaccauccgcacugagg	gi|307603378|gb|CM000998.1|:110765983..110
gi|307603368|gb|CM001008.1|_13982	1.9	0.28 ± 0.02		46	46	0	0	yes				blast	acugaguggacacccuggcaagu	uuccagguaggaaacucaguua	uuccagguaggaaacucaguuacagaacugcccugcugggagaaguccuggugauacugaguggacacccuggcaagu	gi|307603368|gb|CM001008.1|:80893355..8089
gi|307603376|gb|CM001000.1|_42715	1.9	0.28 ± 0.02		117	117	0	0	yes				blast	guacccaggguuuccagcug	gcuggaucccucaacac	guacccaggguuuccagcugcucuggccacucuaggcagcuggaucccucaacac	gi|307603376|gb|CM001000.1|:148247665..148
gi|307603369|gb|CM001007.1|_11357	1.9	0.28 ± 0.02		56	56	0	0	yes				blast	uucuuccuggaaaguuggaccg	guccaaccuuucugauuggcuggaagc	guccaaccuuucugauuggcuggaagccaaauucuggcuucuuccuggaaaguuggaccg	gi|307603369|gb|CM001007.1|:70760400..7076
gi|307603373|gb|CM001003.1|_2118	1.8	0.28 ± 0.02		239	239	0	0	yes				blast	gcugcaacugcgggcucggc	uaagccccagugcaaaca	uaagccccagugcaaacaagcugcaacugcgggcucggc	gi|307603373|gb|CM001003.1|:98725663..9872
gi|307603377|gb|CM000999.1|_37502	1.8	0.28 ± 0.02		93	93	0	0	yes				blast	gccaacccgcagccagag	cuuggccagacgggcucccug	gccaacccgcagccagagcaccccauagugauucuuggccagacgggcucccug	gi|307603377|gb|CM000999.1|:86437875..8643
gi|307603379|gb|CM000997.1|_32854	1.8	0.28 ± 0.02		60	60	0	0	yes				blast	aaagcugauucuggccugcaga	ugcuggccauuuuauacagcuguaa	ugcuggccauuuuauacagcuguaacugagugggagagauuaaagcugauucuggccugcaga	gi|307603379|gb|CM000997.1|:82039110..8203
gi|307603374|gb|CM001002.1|_46399	1.8	0.28 ± 0.02		36	36	0	0	yes				blast	ucuaaugcuucuguuccacag	guggauuugggcagagagacu	guggauuugggcagagagacugguuuggcuagucuaaugcuucuguuccacag	gi|307603374|gb|CM001002.1|:108415565..108
gi|307603376|gb|CM001000.1|_40207	1.8	0.28 ± 0.02		29	29	0	0	yes				blast	uauaacgcuggaggaagag	cuuaucuccagucgugcuagaca	cuuaucuccagucgugcuagacagagcagacagagccucuguauaacgcuggaggaagag	gi|307603376|gb|CM001000.1|:89228227..8922
gi|307603363|gb|CM001013.1|_48328	1.8	0.28 ± 0.02		319	319	0	0	yes				blast	ucccuguucaggcgccaau	ugugugcauggagcgggauc	ucccuguucaggcgccaauggcccacagacacucuggguccccugugugugcauggagcgggauc	gi|307603363|gb|CM001013.1|:52078554..5207
gi|307603378|gb|CM000998.1|_36454	1.8	0.28 ± 0.02		34	34	0	0	yes				blast	cuugcccuugggaagucuga	ggagccccuaaguuuuggau	ggagccccuaaguuuuggauacucuugcccuugggaagucuga	gi|307603378|gb|CM000998.1|:132161792..132
gi|307603372|gb|CM001004.1|_2849	1.8	0.28 ± 0.02		30	30	0	0	yes				blast	gagugucuccagaaguaaugaa	cauuacuucagggagaaaggaca	cauuacuucagggagaaaggacagcugacuggccuggacauccagguauaaaguccccagagugucuccagaaguaaugaa	gi|307603372|gb|CM001004.1|:49935000..4993
gi|307603380|gb|CM000996.1|_29921	1.8	0.28 ± 0.02		17201	17201	0	0	yes				blast	accccacuccugguacca	gggggugggggg	accccacuccugguaccaauaugucuuagucaggguuucuauuuauccagaaacaucaugaccaagaagcaaguuggggggugggggg	gi|307603380|gb|CM000996.1|:61433026..6143
gi|307603381|gb|CM000995.1|_28030	1.8	0.28 ± 0.02		35	35	0	0	yes				blast	agggaagagagcacugcu	uaggugauccuccccuc	uaggugauccuccccucaccccacaccauggugcauuugggaagggaagagagcacugcu	gi|307603381|gb|CM000995.1|:160815918..160
gi|307603380|gb|CM000996.1|_29298	1.8	0.28 ± 0.02		2725	2725	0	0	yes				blast	ucccuguucgggcgccaa	ggucugugaagaacgggauu	ucccuguucgggcgccaacugccacagacaaucuggguccugggucugugaagaacgggauu	gi|307603380|gb|CM000996.1|:114181655..114
gi|307603366|gb|CM001010.1|_16428	1.8	0.28 ± 0.02		318	318	0	0	yes				blast	cacugcuguccucuguguacaca	uguacgcacugcacugcuguccu	uguacgcacugcacugcuguccucuguguacacaaugcacugcuguccucuguguacacacugcacugcuguccucuguguacaca	gi|307603366|gb|CM001010.1|:29422996..2942
gi|307603364|gb|CM001012.1|_19966	1.8	0.28 ± 0.02		39	39	0	0	yes				blast	uuggaaaagaacgguggaa	cugcuuucuuccucgu	uuggaaaagaacgguggaaucucugcuuucuuccucgu	gi|307603364|gb|CM001012.1|:10176906..1017
gi|307603376|gb|CM001000.1|_42535	1.8	0.28 ± 0.02		43	43	0	0	yes				blast	cucuacgauuacaacccuaaccug	aguugggggguggucaggagga	cucuacgauuacaacccuaaccuggccacguacaugaaggugagaacagcagaagaguacuggggguugaguuggggggguggucaggagga	gi|307603376|gb|CM001000.1|:133701147..133
gi|307603375|gb|CM001001.1|_44150	1.8	0.28 ± 0.02		208	208	0	0	yes				blast	uaauuggacagggaaaaucuu	ccuuuucccuguccaaucaca	ccuuuucccuguccaaucacagaccucccucuauauuaauguaauuggacagggaaaaucuu	gi|307603375|gb|CM001001.1|:26184986..2618
gi|307603381|gb|CM000995.1|_26848	1.8	0.28 ± 0.02		39	39	0	0	yes				blast	uaugcacauguagucucuaua	uaggcucuauauacacaugua	uaggcucuauauacacauguaggcucuauauacacauguaggcucuauaugcacauguagucucuaua	gi|307603381|gb|CM000995.1|:33194732..3319
gi|307603382|gb|CM000994.1|_22067	1.8	0.28 ± 0.02		29	29	0	0	yes				blast	uuggaaaugaccugaagc	ucggguguuucugaag	ucggguguuucugaagguuuugugggcaugugcgcaguauggaggaauuuggaaaugaccugaagc	gi|307603382|gb|CM000994.1|:91527121..9152
gi|307603376|gb|CM001000.1|_40709	1.8	0.28 ± 0.02		56024	56024	0	0	yes				blast	agucgguagagcauggga	ccaaguagaugcuuccugu	ccaaguagaugcuuccugugcagucagaugggugugacaggauggcuugcacagucgguagagcauggga	gi|307603376|gb|CM001000.1|:135936047..135
gi|307603382|gb|CM000994.1|_23576	1.8	0.28 ± 0.02		35	35	0	0	yes				blast	uugaugaucuuccgccugcu	caggcucugggggaccuuuacuc	caggcucugggggaccuuuacucuuugaugaucuuccgccugcu	gi|307603382|gb|CM000994.1|:93287199..9328
gi|307603370|gb|CM001006.1|_9246	1.8	0.28 ± 0.02		292	272	20	0	yes				blast	acuucacucugcacuccg	aaguguuuggagugg	acuucacucugcacuccgaggcucagggagcaaguguuuggagugg	gi|307603370|gb|CM001006.1|:83872053..8387
gi|307603365|gb|CM001011.1|_18543	1.8	0.28 ± 0.02		31	31	0	0	yes				blast	aggggacagaagcgaagc	ugcaguccguccccucc	ugcaguccguccccuccuucugcucugccccagccucuccacagccgaggcuccaacaccugaggggacagaagcgaagc	gi|307603365|gb|CM001011.1|:36124693..3612
gi|307603373|gb|CM001003.1|_1926	1.8	0.28 ± 0.02		34	34	0	0	yes				blast	uccugguggagucagacaa	acgugacuuccaucauguucu	acgugacuuccaucauguucuuggugggcacuaagcgaguaugaccaaguccugguggagucagacaa	gi|307603373|gb|CM001003.1|:80994070..8099
gi|307603376|gb|CM001000.1|_42083	1.8	0.28 ± 0.02		34	34	0	0	yes				blast	uggugacauaaccugcugaggaa	cccagguuagcccacuggc	uggugacauaaccugcugaggaacucccacucccccugucauuaggauagugaggccccagguuagcccacuggc	gi|307603376|gb|CM001000.1|:91395627..9139
gi|307603378|gb|CM000998.1|_34044	1.8	0.28 ± 0.02		30	30	0	0	yes				blast	caugggaaugccaugucucuggua	cccuauggcauuucuacugcu	caugggaaugccaugucucugguacucugaccucaguuuuagcagugagcuuauuguacccuauggcauuucuacugcu	gi|307603378|gb|CM000998.1|:29781230..2978
gi|307603374|gb|CM001002.1|_47946	1.8	0.28 ± 0.02		31	31	0	0	yes				blast	guaagcagcaaaguagacagggc	cuuggaacccaacucugcaguuuacug	cuuggaacccaacucugcaguuuacugcuuuaaguaagcagcaaaguagacagggc	gi|307603374|gb|CM001002.1|:112135866..112
gi|307603381|gb|CM000995.1|_27854	1.8	0.28 ± 0.02		35	35	0	0	yes				blast	aucccgaggguuucugua	ccggaagcuuuccugcuag	ccggaagcuuuccugcuaguuccuuuaugaagcagauaaaucccgaggguuucugua	gi|307603381|gb|CM000995.1|:150188426..150
gi|307603377|gb|CM000999.1|_38868	1.8	0.28 ± 0.02		30	30	0	0	yes				blast	gagucuagcugacgccuuguc	gaaggcggcuagauaagg	gaaggcggcuagauaagguuuuacagagucuagcugacgccuuguc	gi|307603377|gb|CM000999.1|:100362981..100
gi|307603381|gb|CM000995.1|_26010	1.8	0.28 ± 0.02		53	53	0	0	yes				blast	ggccaugugagggcaguc	cuucccucccuggccu	ggccaugugagggcagucuaccccagccccuuaauggccaugugcuugggcagucuucccucccuggccu	gi|307603381|gb|CM000995.1|:151390632..151
gi|307603378|gb|CM000998.1|_34443	1.8	0.28 ± 0.02		29	29	0	0	yes				blast	ugguguuuugcgauuggag	gcaaagcaaugccacu	gcaaagcaaugccacuaagaaguaguugguguuuugcgauuggag	gi|307603378|gb|CM000998.1|:77116526..7711
gi|307603376|gb|CM001000.1|_40482	1.8	0.28 ± 0.02		43	43	0	0	yes				blast	ucccuggaguaggcugugg	acauccaaacuccagagauc	ucccuggaguaggcugugggucuugaacacagcacugaccacauccaaacuccagagauc	gi|307603376|gb|CM001000.1|:120001512..120
gi|307603381|gb|CM000995.1|_27594	1.8	0.28 ± 0.02		71	71	0	0	yes				blast	acagccuguggggacgcggc	caguugcgcagcugugu	caguugcgcagcuguguccagcacagccuguggggacgcggc	gi|307603381|gb|CM000995.1|:121299716..121
gi|307603375|gb|CM001001.1|_44038	1.8	0.28 ± 0.02		215	215	0	0	yes				blast	auggcuguggcuguggcug	gcuguggcuguggcugugg	gcuguggcuguggcuguggcugugacugugacuauggauguggauguggcuauggcuguggcuguggcug	gi|307603375|gb|CM001001.1|:11064664..1106
gi|307603381|gb|CM000995.1|_25105	1.8	0.28 ± 0.02		33	33	0	0	yes				blast	ugccauggcuguagcaguuguau	caggcugauacuagcuguggcucc	caggcugauacuagcuguggcuccagagaaugggauugugccauggcuguagcaguuguau	gi|307603381|gb|CM000995.1|:40188390..4018
gi|307603382|gb|CM000994.1|_22464	1.8	0.28 ± 0.02		13703	13703	0	0	yes				blast	ggggauguagcucagugg	caugagacuauaucucuag	ggggauguagcucagugguagagcccaugagacuauaucucuag	gi|307603382|gb|CM000994.1|:156831609..156
gi|307603368|gb|CM001008.1|_14394	1.8	0.28 ± 0.02		35	35	0	0	yes				blast	aggcaaaugcugagcaca	ugucagcaugggcucca	ugucagcaugggcucccacacagcagaggcaaaugcugagcaca	gi|307603368|gb|CM001008.1|:103148642..103
gi|307603369|gb|CM001007.1|_11815	1.8	0.28 ± 0.02		159	159	0	0	yes				blast	uugcaccugauuuguccuugcu	caaguaggagucagucuugcauca	uugcaccugauuuguccuugcuuggaaguguucaaaucgcaaguaggagucagucuugcauca	gi|307603369|gb|CM001007.1|:32005264..3200
gi|307603381|gb|CM000995.1|_26566	1.8	0.28 ± 0.02		50	50	0	0	yes				blast	auagcucaguugguuaga	aggccagcagggcugguc	aggccagcagggcuggucauauagcucaguugguuaga	gi|307603381|gb|CM000995.1|:11445436..1144
gi|307603372|gb|CM001004.1|_3055	1.8	0.28 ± 0.02		41	41	0	0	yes				blast	ccugggcuguucuguaggcc	ggaaaugaggcucauggug	ggaaaugaggcucaugguggaagccugggcuguucuguaggcc	gi|307603372|gb|CM001004.1|:60525325..6052
gi|307603379|gb|CM000997.1|_33681	1.8	0.28 ± 0.02		171	171	0	0	yes				blast	ugagcuuguggugugcuuugc	agaguucaccagugagcucagg	ugagcuuguggugugcuuugcaggagaacuuggaagcagaguucaccagugagcucagg	gi|307603379|gb|CM000997.1|:147991116..147
gi|307603375|gb|CM001001.1|_43675	1.8	0.28 ± 0.02		29	29	0	0	yes				blast	uuguguggcguagcaauuucca	gaggugcugggguggcacugag	gaggugcugggguggcacugagcaagcuggcuucuuuguguggcguagcaauuucca	gi|307603375|gb|CM001001.1|:109398221..109
gi|307603375|gb|CM001001.1|_44841	1.8	0.28 ± 0.02		501	501	0	0	yes				blast	aucugccuguguccgguuuugca	uaaugugugugagugugggguggaugu	aucugccuguguccgguuuugcagagcuuuagucuguaaugugugugagugugggguggaugu	gi|307603375|gb|CM001001.1|:101938159..101
gi|307603379|gb|CM000997.1|_33067	1.8	0.28 ± 0.02		31	31	0	0	yes				blast	aggcuacuggcucucagg	ccugagucagagucauuc	aggcuacuggcucucagggaaaagccuuuccuagcagccugagucagagucauuc	gi|307603379|gb|CM000997.1|:111117588..111
gi|307603372|gb|CM001004.1|_3890	1.8	0.28 ± 0.02		29	29	0	0	yes				blast	uaaccuggaugacacggaagug	cuaaugucaacagguaucu	uaaccuggaugacacggaaguggcucugcugcaggcugugcugcuaaugucaacagguaucu	gi|307603372|gb|CM001004.1|:98625045..9862
gi|307603371|gb|CM001005.1|_7990	1.8	0.28 ± 0.02		34	34	0	0	yes				blast	gcggaagcugugcugggcg	uccugcuaaggauguucguca	uccugcuaaggauguucgucagcaggcggaagcugugcugggcg	gi|307603371|gb|CM001005.1|:74382114..7438
gi|307603371|gb|CM001005.1|_6402	1.8	0.28 ± 0.02		33	33	0	0	yes				blast	ucucaagucaugggcuccug	ugagcugcaaggcaggcugggaag	ucucaagucaugggcuccugcugagcugcaaggcaggcugggaag	gi|307603371|gb|CM001005.1|:25750782..2575
gi|307603377|gb|CM000999.1|_36819	1.8	0.28 ± 0.02		131	131	0	0	yes				blast	ugaccagcacugacaaacugca	uuguuugccagugcugcaccaag	uuguuugccagugcugcaccaaguguuugaccagcacugacaaacugca	gi|307603377|gb|CM000999.1|:5110462..5110
gi|307603381|gb|CM000995.1|_25750	1.7	0.28 ± 0.02		29	29	0	0	yes				blast	gacuucaugucagaaagcaugug	cacugcuuucuaccguggcaggaug	gacuucaugucagaaagcauguguucacugcuuucuaccguggcaggaug	gi|307603381|gb|CM000995.1|:124948268..124
gi|307603373|gb|CM001003.1|_124	1.7	0.28 ± 0.02		40	40	0	0	yes				blast	agcucacugaccagguacugug	caguacuucucagacaugcgcuuu	agcucacugaccagguacuguguugagagacccucggcccacagugcaguacuucucagacaugcgcuuu	gi|307603373|gb|CM001003.1|:25242779..2524
gi|307603382|gb|CM000994.1|_23619	1.7	0.28 ± 0.02		38	38	0	0	yes				blast	guuuguagaggccugccu	uugggccaccccaggcag	guuuguagaggccugccucaauaaggacccacgauucguaaggccuauauccacuugggccaccccaggcag	gi|307603382|gb|CM000994.1|:95522322..9552

gi|307603381|gb|CM000995.1|_26288	1.7	0.28 ± 0.02		57	57	0	0	yes				blast	agcugcuggcuguuagua	guaacagccaguuaagaa	agcugcuggcuguuaguaagguaacagccaguuaagaa	gi|307603381|gb|CM000995.1|:166762919..166
gi|307603371|gb|CM001005.1|_7598	1.7	0.28 ± 0.02		32	32	0	0	yes				blast	ucguuccugggcuaccucugac	cugggguagucacaggagcagc	cuggguagucacaggagcagcugugaguuugaucgcucguuccugggcuaccucugac	gi|307603371|gb|CM001005.1|:16703660..1670
gi|307603367|gb|CM001009.1|_14777	1.7	0.28 ± 0.02		358	358	0	0	yes				blast	acuccuaugguuacugugug	cgcgggacaggaaaaa	cgcgggacaggaaaaauagaagcuggaaacaagcggcuuacaagcuguacuccuaugguuacugugug	gi|307603367|gb|CM001009.1|:32420255..3242
gi|307603378|gb|CM000998.1|_36034	1.7	0.28 ± 0.02		30	30	0	0	yes				blast	gugugagcagaugaaggua	cuuuggugcucacccc	cuuuggugcucaccccugguggugauauuucaccugaggaaaagguggaacgugugagcagaugaaggua	gi|307603378|gb|CM000998.1|:103340347..103
gi|307603366|gb|CM001010.1|_16427	1.7	0.28 ± 0.02		379	339	40	0	yes				blast	cacugcuguccucuguguacaca	uguacacacugcacugcuguccu	uguacacacugcacugcuguccucuguguacacacugcacugcuguccucuguguacgcacugcacugcuguccucuguguacaca	gi|307603366|gb|CM001010.1|:29422944..2942
gi|307603365|gb|CM001011.1|_19106	1.7	0.28 ± 0.02		37	37	0	0	yes				blast	cauuuagaagcuagaaca	ugcuucuggcuugcaaacugua	ugcuucuggcuugcaaacuguaggcagcagccaugaacccaggaauguugaugacauuuagaagcuagaaca	gi|307603365|gb|CM001011.1|:13085616..1308
gi|307603381|gb|CM000995.1|_28203	1.7	0.28 ± 0.02		28	28	0	0	yes				blast	gacaaugcugggaagacg	ucucaccaguucccauuccc	gacaaugcugggaagacgguaggucccugcucucucaccaguucccauuccc	gi|307603381|gb|CM000995.1|:181099275..181
gi|307603377|gb|CM000999.1|_36962	1.7	0.28 ± 0.02		249	249	0	0	yes				blast	uuauggcccuucgguaauucacu	ugaauucuaccagugccauacac	uuauggcccuucgguaauucacugacugagacuguucacagugaauucuaccagugccauacac	gi|307603377|gb|CM000999.1|:30119671..3011
gi|307603372|gb|CM001004.1|_5091	1.7	0.28 ± 0.02		31	31	0	0	yes				blast	ugauugcuuuagguucggcu	ccggucucugagcauccu	ugauugcuuuagguucggcuuacauuuuccggaagaggaaagcggacuccggucucugagcauccu	gi|307603372|gb|CM001004.1|:69392644..6939
gi|307603371|gb|CM001005.1|_7476	1.7	0.28 ± 0.02		32	32	0	0	yes				blast	uguccaguguagaucuaug	cagcuccuacuggauaug	uguccaguguagaucuauggccauuggagucuggcagcuccuacuggauaug	gi|307603371|gb|CM001005.1|:118820220..118
gi|307603369|gb|CM001007.1|_12486	1.7	0.28 ± 0.02		44	44	0	0	yes				blast	uugacuggcgucugggcua	gccuggauaguuuguggaua	gccuggauaguuuguggauauaauuauugacuggcgucugggcua	gi|307603369|gb|CM001007.1|:109312671..109
gi|307603373|gb|CM001003.1|_1667	1.7	0.28 ± 0.02		57	57	0	0	yes				blast	uguucucuucacuguggga	cuacaguuaaagggaccucucc	uguucucuucacugugggacgcagguuccugacagggaaucugcucagcauuucuacaguuaaagggaccucucc	gi|307603373|gb|CM001003.1|:61634807..6163
gi|307603373|gb|CM001003.1|_1852	1.7	0.28 ± 0.02		157	157	0	0	yes				blast	accugcuguggaccuagcc	ugagugucuagggucucagcccc	ugagugucuagggucucagccccucaucauuccaucccccucagaggaccugcuguggaccuagcc	gi|307603373|gb|CM001003.1|:79763394..7976
gi|307603364|gb|CM001012.1|_21004	1.7	0.28 ± 0.02		38	38	0	0	yes				blast	acgucccgugcuaauaggcu	acuauugcacggguccuaucu	acuauugcacggguccuaucuccugcagccugguguaagagacguccccgugcuaauaggcu	gi|307603364|gb|CM001012.1|:29101520..2910
gi|307603366|gb|CM001010.1|_17873	1.7	0.28 ± 0.02		39	39	0	0	yes				blast	uugcuucuuccuguguggua	cccacagugcugugucu	uugcuucuuccugugugguaauggcccacagugcugugucu	gi|307603366|gb|CM001010.1|:46936720..4693
gi|307603371|gb|CM001005.1|_8197	1.7	0.28 ± 0.02		328	328	0	0	yes				blast	ugaagcacuuguggcucacugga	cagugugcagagugauuucaac	ugaagcacuuguggcucacuggacugggggaaugaguguuagccauccagugugcagagugauuucaac	gi|307603371|gb|CM001005.1|:99668660..9966
gi|307603371|gb|CM001005.1|_8136	1.7	0.28 ± 0.02		36	36	0	0	yes				blast	gggaaggaacccuagagu	ucuaacagguggcuaauuuucuc	ucuaacagguggcuaauuuucucuuaugccuuauuucuggaggcacuguuagagggaaggaacccuagagu	gi|307603371|gb|CM001005.1|:88448269..8844
gi|307603377|gb|CM000999.1|_37919	1.7	0.28 ± 0.02		117	117	0	0	yes				blast	auccuggacgagcccccca	ugggcaugauguaggugua	ugggcaugauguaggugguauguaccucccaagggguauuuugaguccuuggggacccccucaaauccuggacgagcccccca	gi|307603377|gb|CM000999.1|:133504032..133
gi|307603378|gb|CM000998.1|_34542	1.7	0.28 ± 0.02		209	209	0	0	yes				blast	cccacggacgcauaggagag	uuccgugucuuauggac	cccacggacgcauaggagaguuugaacaaagacgaacuuccgugucuuauggac	gi|307603378|gb|CM000998.1|:93635587..9363
gi|307603376|gb|CM001000.1|_42067	1.7	0.28 ± 0.02		120	120	0	0	yes				blast	cuuuuggggucccacugcuguu	uggagggucccucagga	cuuuuggggucccacugcuguuccuuuaaaacgaacacgaaacugauugugucgugaacugggauggagggucccucagga	gi|307603376|gb|CM001000.1|:90013461..9001
gi|307603377|gb|CM000999.1|_38837	1.7	0.28 ± 0.02		44	44	0	0	yes				blast	uggaauugucagugacuugagg	ucuuuucucuguguuccagg	uggaauugucagugacuugaggcugacgguccuucuaaccgcugccgaagugaccgagccucuuuucucuguguuccagg	gi|307603377|gb|CM000999.1|:97478844..9747
gi|307603365|gb|CM001011.1|_19021	1.7	0.28 ± 0.02		93355	93355	0	0	yes				blast	agucgguagagcaugagac	cguagguuugagccccgauaugg	agucgguagagcaugagacucuuaaucucagggucguagguuugagccccgauaugg	gi|307603365|gb|CM001011.1|:3012623..30126
gi|307603364|gb|CM001012.1|_21172	1.7	0.28 ± 0.02		228	228	0	0	yes				blast	uugcucuggcccuuaggc	cucgagggcggagcaugaa	cucgagggcggagcaugaagcugauuggcuuaccuaggaaacaaaggggguuuugcucuggcccuuaggc	gi|307603364|gb|CM001012.1|:45132805..4513
gi|307603371|gb|CM001005.1|_7697	1.7	0.28 ± 0.02		29	29	0	0	yes				blast	gcgcaucagauggaguccug	gguuccauucaggaauguguug	gcgcaucagauggaguccuguugguuccauucaggaauguguug	gi|307603371|gb|CM001005.1|:30634166..3063
gi|307603366|gb|CM001010.1|_17170	1.7	0.28 ± 0.02		135	135	0	0	yes				blast	gggaacaaagagcgagagcuguag	gcgggucgugggcucugggcuccugu	gggaacaaagagcgagagcuguagaagcgggucgugggcucugggcuccugu	gi|307603366|gb|CM001010.1|:88012910..8801
gi|307603380|gb|CM000996.1|_30270	1.7	0.28 ± 0.02		28	28	0	0	yes				blast	caggcucuggguggugcaugcuu	ggaucugcauccaccucacaaaaagc	ggaucugcauccaccucacaaaaagcuugcaaaggugaaaucuucuaaagaucccaaggcaggccaggcucuggguggugcaugcuu	gi|307603380|gb|CM000996.1|:95869239..9586
gi|307603371|gb|CM001005.1|_7956	1.7	0.28 ± 0.02		33	33	0	0	yes				blast	acacccgagggcugccuuc	guguagcccucgcggucca	guguagcccucgcgguccagcacacccgagggcugccuuc	gi|307603371|gb|CM001005.1|:71088359..7108
gi|307603366|gb|CM001010.1|_16302	1.7	0.28 ± 0.02		1097	1030	0	67	yes				blast	acucucucacucugcaugguuaca	uucguacggagagagagaga	acucucucacucugcaugguuacacaagguuuuacccgugugacuuucguacggagagagagaga	gi|307603366|gb|CM001010.1|:24857716..2485
gi|307603364|gb|CM001012.1|_20326	1.7	0.28 ± 0.02		71	71	0	0	yes				blast	uagcauuguaggauaggguca	accugccaagggugcugug	uagcauuguaggauagggucagucacauggccaguuuuauguucccucugaccugccaagggugcugug	gi|307603364|gb|CM001012.1|:42265726..4226
gi|307603381|gb|CM000995.1|_25119	1.7	0.28 ± 0.02		117	117	0	0	yes				blast	guucaugagugcucucccacuua	ugggggagagaacacaagagugu	ugggggagagaacacaagaguguuacaaguucaugagugcucucccacuua	gi|307603381|gb|CM000995.1|:49155183..4915
gi|307603378|gb|CM000998.1|_34856	1.7	0.28 ± 0.02		29	29	0	0	yes				blast	caggagacagugcuguguguguu	gcgggagucacuagcugga	caggagacagugcugugugugugugguuagggcaggagcccugacuaaggcgggagucacuagcugga	gi|307603378|gb|CM000998.1|:118433047..118
gi|307603374|gb|CM001002.1|_46722	1.6	0.28 ± 0.02		36	36	0	0	yes				blast	gcggacucugguauccaugugua	ugugugugugucucugcug	ugugugugugucucugcugggggcaugugccgcacuucccagcggacucugguauccaugugua	gi|307603374|gb|CM001002.1|:14346603..1434
gi|307603364|gb|CM001012.1|_20299	1.6	0.28 ± 0.02		31	31	0	0	yes				blast	ugcugauuugauugccauuguga	acaagggccaaucucccucagcuga	acaagggccaaucucccucagcugaacccucaggcgguauuuugcugauuugauugccauuguga	gi|307603364|gb|CM001012.1|:40911425..4091
gi|307603382|gb|CM000994.1|_24465	1.6	0.28 ± 0.02		112	112	0	0	yes				blast	agaagggagcaugguccc	gauacuugcuuguga	agaagggagcauggucccccaagucucagugucccuuaucuucuagggauaucacugggauuaagugggauacuugcuuguga	gi|307603382|gb|CM000994.1|:194320858..194
gi|307603372|gb|CM001004.1|_5390	1.6	0.28 ± 0.02		38	38	0	0	yes				blast	aaggucugcagaauauaaggaga	uccaaauagugucuccagccccuga	uccaaauagugucuccagccccugauaucaccugccacguuagguugcggugcugguggggaccaaggucugcagaauauaaggaga	gi|307603372|gb|CM001004.1|:86020832..8602
gi|307603382|gb|CM000994.1|_21619	1.6	0.28 ± 0.02		56	56	0	0	yes				blast	aggagccuagcauagcugu	agcucaaggggaggcucaaag	agcucaaggggaggcucaaaggccugacacuauuacugguguuaugguguguuacagagaggagccuagcauagcugu	gi|307603382|gb|CM000994.1|:43655404..4365
gi|307603380|gb|CM000996.1|_29251	1.6	0.28 ± 0.02		157	157	0	0	yes				blast	acagcucucucucucucugaagu	ugcagagagaugcggggaggguua	ugcagagagaugcggggaggguuaaaggaaggaaucacguucaacuaacagcucucucucucucugaagu	gi|307603380|gb|CM000996.1|:108075138..108
gi|307603375|gb|CM001001.1|_44807	1.6	0.28 ± 0.02		46	46	0	0	yes				blast	auugaguagaguaggggc	gccugcugcuugagcc	auugaguagaguaggggcugaggggaaggcggcucaguuauugcggugccugcugcuugagcc	gi|307603375|gb|CM001001.1|:97848421..9784
gi|307603381|gb|CM000995.1|_25875	1.6	0.28 ± 0.02		51	51	0	0	yes				blast	uuguuucaucuccuggguuugu	aaacaccaggacugaaaacagcc	aaacaccaggacugaaaacagccuggagaggaaagggguuguuucaucuccuggguuugu	gi|307603381|gb|CM000995.1|:132536503..132
gi|307603368|gb|CM001008.1|_14174	1.6	0.28 ± 0.02		29	29	0	0	yes				blast	cugcuguagcugcugcugu	agaaucugacuagagcauug	agaaucugacuagagcauugcuguuugcugcuguugcugcuguagcugcugcugu	gi|307603368|gb|CM001008.1|:93937661..9393
gi|307603366|gb|CM001010.1|_16429	1.6	0.28 ± 0.02		40	40	0	0	yes				blast	acugcuguccucuguguacgc	guacacacugcacugc	guacacacugcacugcugucaucccucgguguacgcacugcacugcuguccucuguguacgcacggcacugcuguccucuguguacgc	gi|307603366|gb|CM001010.1|:29423578..2942
gi|307603365|gb|CM001011.1|_19389	1.6	0.28 ± 0.02		31	31	0	0	yes				blast	uguuguauuaugaugugu	auggcaguggugcuccauc	uguuguauuaugauguguguuaauuuccauggaggauggucacauaggcaguggugcuccauc	gi|307603365|gb|CM001011.1|:43483434..4348
gi|307603364|gb|CM001012.1|_20430	1.6	0.28 ± 0.02		34	34	0	0	yes				blast	cagagguagagagaaaucaaga	uggcauuuuucuccuucucucuc	cagagguagagagaaaucaagacuuggcauuuuucuccuucucucuc	gi|307603364|gb|CM001012.1|:46575505..4657
gi|307603380|gb|CM000996.1|_30100	1.6	0.28 ± 0.02		1060	369	0	691	yes				blast	uacagcuagauaaccaaagaua	uucgguuaucuagcuuuauga	uucgguuaucuagcuuuaugaagacuccacaccacucauacagcuagauaaccaaagaua	gi|307603380|gb|CM000996.1|:88019532..8801
gi|307603366|gb|CM001010.1|_16317	1.6	0.28 ± 0.02		43	43	0	0	yes				blast	uuaaggaccuagccacccagga	caguggguggcugcggcccauaauu	caguggguggcugcggcccauaauuaagguccucaaagagcaauuaaggaccuagccacccagga	gi|307603366|gb|CM001010.1|:25572084..2557
gi|307603376|gb|CM001000.1|_41364	1.6	0.28 ± 0.02		31	31	0	0	yes				blast	augauccccugccuaggc	gugggaagacugggaucuuuca	gugggaagacugggaucuuucagugugcugaugauccccugccuaggc	gi|307603376|gb|CM001000.1|:31629733..3162
gi|307603367|gb|CM001009.1|_15745	1.6	0.28 ± 0.02		44	44	0	0	yes				blast	ccggcacaacagaaacaugagu	ucaugugucucuuguguugauc	ccggcacaacagaaacaugagugguacauacagagucaugugucucuuguguugauc	gi|307603367|gb|CM001009.1|:44490468..4449
gi|307603365|gb|CM001011.1|_18585	1.6	0.28 ± 0.02		132	132	0	0	yes				blast	ugggucgcgauuuccucuucc	aagaggaaguagagaucaaagucagc	aagaggaaguagagaucaaagucagcgagcauaccaguccugggucgcgauuuccucuucc	gi|307603365|gb|CM001011.1|:37915997..3791
gi|307603381|gb|CM000995.1|_25526	1.6	0.28 ± 0.02		30	30	0	0	yes				blast	gugcuuaguucgguucca	cugcuggagugagccag	gugcuuaguucgguuccaggcugcuggagugagccag	gi|307603381|gb|CM000995.1|:105511454..105
gi|307603370|gb|CM001006.1|_10485	1.6	0.28 ± 0.02		33	33	0	0	yes				blast	acagagggacaaagggcacuu	guacccguggcucucuguga	acagagggacaaagggcacuucuugauuucagaguacccguggcucucuguga	gi|307603370|gb|CM001006.1|:98668638..9866
gi|307603371|gb|CM001005.1|_6417	1.6	0.28 ± 0.02		32	32	0	0	yes				blast	cucaccaggaacuguugaca	ucagccauucugugaggu	ucagccauucugugagguuuucuuacacaaggucugaugcucaccaggaacuguugaca	gi|307603371|gb|CM001005.1|:29341111..2934
gi|307603382|gb|CM000994.1|_22495	1.6	0.28 ± 0.02		42	42	0	0	yes				blast	aguggguugcacaaagga	cacugugcccucugc	cacugugcccucugcaggaggaaggcuucuuucucacacagccuuggagccucuuugcaguggguugcacaaagga	gi|307603382|gb|CM000994.1|:159009661..159
gi|307603375|gb|CM001001.1|_43536	1.6	0.28 ± 0.02		30	30	0	0	yes				blast	uugucugcccagcaugga	ccugacuucugacuugaccccu	uugucugcccagcauggacagcagcccccaacuccuguuguucucccugacuucugacuugaccccu	gi|307603375|gb|CM001001.1|:96484273..9648
gi|307603376|gb|CM001000.1|_41558	1.6	0.28 ± 0.02		36	36	0	0	yes				blast	guaagguagcagaggga	ccuuccacauacuaacuacgu	ccuuccacauacuaacuacguggcucagcaagucacggagacaagaccagcuuagcuaaguaagguagcagaggga	gi|307603376|gb|CM001000.1|:52936840..5293
gi|307603363|gb|CM001013.1|_49815	1.6	0.28 ± 0.02		226	172	0	54	yes				blast	uguguauuccugguuguucug	cuggccuugaacucacag	uguguauuccugguuguucuguagcaugcucuguagaacaggcuggccuugaacucacag	gi|307603363|gb|CM001013.1|:164856538..164
gi|307603376|gb|CM001000.1|_42629	1.6	0.28 ± 0.02		29	29	0	0	yes				blast	ugggugugcacugaucug	gcucugcucgcccgaa	ugggugugcacugaucuguaagugucacaaagcccagaccuucagcucugcucgcccgaa	gi|307603376|gb|CM001000.1|:139478981..139
gi|307603367|gb|CM001009.1|_14851	1.6	0.28 ± 0.02		38	38	0	0	yes				blast	uuggucacucaggaagaa	uuaccuggauucuguacaagg	uuaccuggauucuguacaaggcaaagccuuggucacucaggaagaa	gi|307603367|gb|CM001009.1|:37899588..3789
gi|307603365|gb|CM001011.1|_19655	1.6	0.28 ± 0.02		394	394	0	0	yes				blast	uuuugagacaggcugaccugg	aggcugguuggucuucaacu	uuuugagacaggcugaccugggauagacucauacagcccaggcugguuggucuucaacu	gi|307603365|gb|CM001011.1|:75853836..7585
gi|307603373|gb|CM001003.1|_1517	1.6	0.28 ± 0.02		657	657	0	0	yes				blast	agaucaucaucccagggcugga	caacuuaugagugaugauuugg	agaucaucaucccagggcuggauuugagaaccgacuguccaacuuaugagugaugauuugg	gi|307603373|gb|CM001003.1|:42730944..4273
gi|307603381|gb|CM000995.1|_25521	1.6	0.28 ± 0.02		40	40	0	0	yes				blast	acugcccaugcuggagagc	uuaaaauagcaaacaggag	uuaaaauagcaaacaggaguccccauuuuggggucuacugcccaugcuggagagc	gi|307603381|gb|CM000995.1|:104655921..104
gi|307603381|gb|CM000995.1|_25639	1.5	0.28 ± 0.02		92	92	0	0	yes				blast	ugccugugggcugauuccugaagu	ggcagugaaacagaccagaguugac	ggcagugaaacagaccagaguugacaaguuccuuuuauucugccuguggcugauuccugaagu	gi|307603381|gb|CM000995.1|:118642681..118
gi|307603366|gb|CM001010.1|_16137	1.5	0.28 ± 0.02		286	286	0	0	yes				blast	gucucucguacauggucuaga	ugggccauuguacauggagauga	ugggccauuguacauggagaugaaaugguuuugucucucguacauggucuaga	gi|307603366|gb|CM001010.1|:13115724..1311
gi|307603375|gb|CM001001.1|_42851	1.5	0.28 ± 0.02		43	43	0	0	yes				blast	ccaaccaauccagccauu	aggcugggccuuggca	aggcugggccuuggcaaccaguagggcgggaucauugugccucuggcccaccaaccaauccagccauu	gi|307603375|gb|CM001001.1|:4217547..42176
gi|307603369|gb|CM001007.1|_12170	1.5	0.28 ± 0.02		31	31	0	0	yes				blast	accuuggcucugauucuuca	cgaauguagaggaaggaggugg	cgaauguagaggaaggagguggcucaggcugaccuuggcucugauucuuca	gi|307603369|gb|CM001007.1|:65211933..6521
gi|307603374|gb|CM001002.1|_46086	1.5	0.28 ± 0.02		245	245	0	0	yes				blast	gcucaggaacccggcuug	cccugguagcccuccaaagcaa	gcucaggaacccggcuugcgcgcugcucuuaucaauucuaucgcgcucccugguagccuccaaagcaa	gi|307603374|gb|CM001002.1|:73816506..7381
gi|307603377|gb|CM000999.1|_38839	1.5	0.28 ± 0.02		80	80	0	0	yes				blast	ggagcaggaucaauguacaaugua	cauuguuuuacucccugcucaaa	ggagcaggaucaauguacaauguagcuguaaagugcuacauuguuuuacucccugcucaaa	gi|307603377|gb|CM000999.1|:97497501..9749
gi|307603372|gb|CM001004.1|_4946	1.5	0.28 ± 0.02		42	42	0	0	yes				blast	acagggucagauuaagug	cuuacugggccugucc	cuuacugggccuguccugcaguugaagggacaggucagauuaagug	gi|307603372|gb|CM001004.1|:60717049..6071
gi|307603373|gb|CM001003.1|_894	1.5	0.28 ± 0.02		70	70	0	0	yes				blast	uugagucggguagaaucugugg	agaggcuuuacccgaacacugu	uugagucggguagaaucuguggcuaacuuacaacccagaggcuuuacccgaacacugu	gi|307603373|gb|CM001003.1|:90215443..9021
gi|307603375|gb|CM001001.1|_44097	1.5	0.28 ± 0.02		242	242	0	0	yes				blast	cauaugagcagaccuccaguuu	acugucugucuguucauagcca	cauaugagcagaccuccaguuuaucauuuuuaaaaacugucugucuguucauagcca	gi|307603375|gb|CM001001.1|:13885878..1388
gi|307603380|gb|CM000996.1|_29960	1.5	0.28 ± 0.02		47	47	0	0	yes				blast	uugggugugaaguggaaacug	guaccugacgucccauccauug	guaccugacgucccauccauugccaucaagggagaauuggagguuuggggugugaaguggaaacug	gi|307603380|gb|CM000996.1|:67823264..6782
gi|307603380|gb|CM000996.1|_30138	1.5	0.28 ± 0.02		202433	202433	0	0	yes				blast	aucucgcuggggccucca	gagccccagaaugaaga	gagccccagaaugaagaugcagcuuuagaauuugggugcuaucucgcuggggccucca	gi|307603380|gb|CM000996.1|:88920531..8892
gi|307603372|gb|CM001004.1|_5025	1.5	0.28 ± 0.02		29	29	0	0	yes				blast	gacccuucaggacugaccugg	ugguuccuuuagggucuc	gacccuucaggacugaccuggggaaugguuccuuuagggucuc	gi|307603372|gb|CM001004.1|:68023392..6802
gi|307603372|gb|CM001004.1|_3498	1.5	0.28 ± 0.02		57	57	0	0	yes				blast	uggcgguugguguggagaacucug	uuguuuuuuccagcuucuuuccgccucu	uggcgguugguguggagaacucugcgagucucuuggguuuuuuguuuuuuguuuuuuccagcuucuuuccgccucu	gi|307603372|gb|CM001004.1|:80196853..8019
gi|307603382|gb|CM000994.1|_23924	1.5	0.28 ± 0.02		626	302	240	84	yes				blast	caggcagccggggaccgcugaau	ucggcuggagcagucccc	uccggcuggagcaguccccaacgcaaccuacaggcacuggaacuugcaggcagccggggaccgcugaau	gi|307603382|gb|CM000994.1|:140738963..140
gi|307603373|gb|CM001003.1|_2287	1.5	0.28 ± 0.02		38	38	0	0	yes				blast	aucaacagcaggacugccaaga	cuggcuuuccuguuggauggacu	aucaacagcaggacugccaagaacuacuggcuuuccuguuggauggacu	gi|307603373|gb|CM001003.1|:122568380..122
gi|307603382|gb|CM000994.1|_22432	1.5	0.28 ± 0.02		59	59	0	0	yes				blast	aucacacugggguguagcuuga	aaaggccgcccuccacagugacac	aucacacugggguguagcuugagcauauaugacucaaaggccgcccuccacagugacac	gi|307603382|gb|CM000994.1|:154241485..154
gi|307603374|gb|CM001002.1|_46261	1.5	0.28 ± 0.02		29	29	0	0	yes				blast	ugucugugaacaguggaaguagca	uggcuuccacucaucucguauaga	uggcuuccacucaucucguauagaaacucauuagucacuggaggugaagaugacaggaugucugugaacaguggaaguagca	gi|307603374|gb|CM001002.1|:100847558..100
gi|307603382|gb|CM000994.1|_22929	1.5	0.28 ± 0.02		188	188	0	0	yes				blast	gucagagcuugugcacug	uugccaucacagcuucuucca	gucagagcuugugcacuguuuggcucuuccaugccuuuucuugccaucacagcuucuucca	gi|307603382|gb|CM000994.1|:13335822..1333
gi|307603372|gb|CM001004.1|_5193	1.5	0.28 ± 0.02		76	76	0	0	yes				blast	gcgccagaggcuggggag	ucugaguguggcaggg	gcgccagaggcuggggaggcccucuguagagcaguguuuucugaguguggcaggg	gi|307603372|gb|CM001004.1|:73104657..7310
gi|307603380|gb|CM000996.1|_29640	1.5	0.28 ± 0.02		32	32	0	0	yes				blast	ggguucagaaauaaagug	uuuaugucuggcaguccau	uuuaugucuggcaguccauuuugaaugaacaugguucagaaauaaagug	gi|307603380|gb|CM000996.1|:16719336..1671
gi|307603379|gb|CM000997.1|_32777	1.5	0.28 ± 0.02		30	30	0	0	yes				blast	gguucuggagccagccauuga	cccggcugccuuuuaaaggcuug	gguucuggagccagccauugauuugauucccggcugccuuuuaaaggcuug	gi|307603379|gb|CM000997.1|:68613498..6861
gi|307603381|gb|CM000995.1|_27596	1.5	0.28 ± 0.02		463	463	0	0	yes				blast	agacuuggcacucagcaugaua	acgugcugaauuccaucccacugg	acgugcugaauuccaucccacuggcaucugaaaucccagacuuggcacucagcaugaua	gi|307603381|gb|CM000995.1|:121407670..121
gi|307603376|gb|CM001000.1|_40008	1.5	0.28 ± 0.02		264	264	0	0	yes				blast	aggggaaucugacugucua	gacaaugaauugguuccaaccuuc	aggggaaucugacugucuaaugacugacaaugaauugguuccaaccuuc	gi|307603376|gb|CM001000.1|:59083015..5908
gi|307603377|gb|CM000999.1|_38804	1.5	0.28 ± 0.02		31	31	0	0	yes				blast	uuaaaaaucugagaccugucugg	aguucuugaacauuaaca	aguucuugaacauuaacauuguggauggcaacguugacaucauaguguuaaaaaucugagaccugucugg	gi|307603377|gb|CM000999.1|:93789434..9378
gi|307603376|gb|CM001000.1|_39681	1.5	0.28 ± 0.02		31	31	0	0	yes				blast	agguagauaaccauugaag	uuaagccaaucuccccucu	uuaagccaaucucccucucccuagcaucaauugggagugcucaccuggaggaggguagauaaccauugaag	gi|307603376|gb|CM001000.1|:28139346..2813
gi|307603382|gb|CM000994.1|_23667	1.5	0.28 ± 0.02		56013	55861	152	0	yes				blast	agcuaccaucugugggauua	aucccgcccaggugauaucccu	agcuaccaucugugggauuagacuaaaugccucuaagucagaaucccgcccaggugauaucccu	gi|307603382|gb|CM000994.1|:102076663..102
gi|307603371|gb|CM001005.1|_7559	1.5	0.28 ± 0.02		29	29	0	0	yes				blast	acagaguucccccaaauaa	gacugggggaacucuguga	acagaguucccccaaauaaaauaagcuuccaugucauuaaugagacugggggaacucuguga	gi|307603371|gb|CM001005.1|:12293705..1229
gi|307603370|gb|CM001006.1|_9445	1.5	0.28 ± 0.02		186	186	0	0	yes				blast	ugauguggaccauccuggggug	ucccaggcuguguucacagacuu	ucccaggcuguguucacagacuuggagaaucucgcggccauuuuugccagugcaauacaugauguggaccauccuggggug	gi|307603370|gb|CM001006.1|:110729463..110
gi|307603372|gb|CM001004.1|_3819	1.5	0.28 ± 0.02		35	35	0	0	yes				blast	agagacaaaggggccgag	gggcgucuggagucucccu	gggcgucuggagucucccuuaagcucuguguaaauaaacugagggggaggcucaggagcaguggcugggagagacaaaggggccgag	gi|307603372|gb|CM001004.1|:96733801..9673
gi|307603372|gb|CM001004.1|_6174	1.5	0.28 ± 0.02		34	34	0	0	yes				blast	ugcuacaacuggaauacaca	uguauucccgaaucccggcacu	uguauucccgaaucccggcacugcucaacacccagcccaugcuacaacuggaauacaca	gi|307603372|gb|CM001004.1|:120675273..120
gi|307603363|gb|CM001013.1|_49023	1.5	0.28 ± 0.02		32	32	0	0	yes				blast	ugugaguuacaggcuggcuugg	aggacagccaggacuacacaaa	ugugaguuacaggcuggcuugguauacagagagaguucuaggacagccaggacuacacaaa	gi|307603363|gb|CM001013.1|:11777156..1177
gi|307603370|gb|CM001006.1|_10683	1.5	0.28 ± 0.02		171	171	0	0	yes				blast	cguuggagagauggcuca	aguucaauucccagccac	cguuggagagauggcucagcgauuaagagcaccgaaugcucuuccagaguccugaguucaauucccagccac	gi|307603370|gb|CM001006.1|:115289961..115
gi|307603366|gb|CM001010.1|_16611	1.5	0.28 ± 0.02		32	32	0	0	yes				blast	gugugugagacaagcagaacgcc	gguuaaugagcucacauacaa	gguuaaugagcucacauacaaaagaugguggaucuuguggcccagaagucccacuucuuugugugugagacaagcagaacgcc	gi|307603366|gb|CM001010.1|:36055031..3605
gi|307603376|gb|CM001000.1|_40993	1.4	0.28 ± 0.02		36	36	0	0	yes				blast	gaguaugccugccaggag	ccugguggccaggucc	gaguaugccugccaggagggcccuguaaugguacaaacugagaaaggcagguagaaccugguggccaggucc	gi|307603376|gb|CM001000.1|:6236353..62364
gi|307603373|gb|CM001003.1|_1265	1.4	0.28 ± 0.02		44	44	0	0	yes				blast	augggaugaggacuuugagc	ucucuucagauccacuu	augggaugaggacuuugagcacggugaguuuacaaucgcuucucuucagauccacuu	gi|307603373|gb|CM001003.1|:6628319..66283
gi|307603368|gb|CM001008.1|_12569	1.4	0.28 ± 0.02		30	30	0	0	yes				blast	aaucucugaauaugcagac	cugcacuugauuccuggcuugc	cugcacuugauuccuggcuugcuaaguaaucucugaauaugcagac	gi|307603368|gb|CM001008.1|:3981436..39814
gi|307603377|gb|CM000999.1|_37862	1.4	0.28 ± 0.02		540	540	0	0	yes				blast	cuuguccugugccuguaacug	cuggcaggacacacagugauuuccc	cuuguccugugccuguaacugcuacccugugcucaguccuaacaggcugucuggcaggacacacagugauuuccc	gi|307603377|gb|CM000999.1|:125071740..125
gi|307603372|gb|CM001004.1|_2626	1.4	0.28 ± 0.02		86	86	0	0	yes				blast	gguuaagagcacuggcugc	aaccaacucacaaccau	gguuaagagcacuggcugcucuuccagaggaaugaguuuugauucccaggauucacauggcaaccaacucacaaccau	gi|307603372|gb|CM001004.1|:23406625..2340
gi|307603374|gb|CM001002.1|_47722	1.4	0.28 ± 0.02		116	116	0	0	yes				blast	uacaaggaggcugcugac	cacugccuucuucccuu	cacugccuucuucccuucccccuucaucccuuuugcaaguaggaauuacaaggaggcugcugac	gi|307603374|gb|CM001002.1|:101811276..101
gi|307603380|gb|CM000996.1|_29546	1.4	0.28 ± 0.02		30	30	0	0	yes				blast	gaugcagggccucuaccgaaagc	cugagguuuaaggcuuccugcugaau	gaugcagggccucuaccgaaagccuaggcaggccugagguuuaaggcuuccugcugaau	gi|307603380|gb|CM000996.1|:151874902..151
gi|307603376|gb|CM001000.1|_40738	1.4	0.28 ± 0.02		42	42	0	0	yes				blast	acuggccuguuugagugag	caggauaggacaguca	acuggccuguuugagugagucuugucccacaucuggauugcugggacucaggauaggacaguca	gi|307603376|gb|CM001000.1|:137870487..137
gi|307603379|gb|CM000997.1|_33482	1.4	0.28 ± 0.02		77	77	0	0	yes				blast	uguguggccacuaagugcuuugag	cacuguacucccaccauacacc	cacuguacucccaccauacaccugagcuuccucucacuuucuguguggccacuaagugcuuugag	gi|307603379|gb|CM000997.1|:134366770..134
gi|307603381|gb|CM000995.1|_27718	1.4	0.28 ± 0.02		29	29	0	0	yes				blast	aaucucucucucucugugugga	ccagagagcagggaguggaa	ccagagagcagggaguggaagccaguccauucaucaaucucucucucugugugga	gi|307603381|gb|CM000995.1|:130106871..130
gi|307603378|gb|CM000998.1|_33961	1.4	0.28 ± 0.02		82	82	0	0	yes				blast	gauucccuggauccagauuugc	augucugguacccauggaaacca	augucugguacccauggaaaccagaaaagagugugguugggauucccuggauccagauuugc	gi|307603378|gb|CM000998.1|:22271733..2227
gi|307603372|gb|CM001004.1|_5355	1.4	0.28 ± 0.02		39	39	0	0	yes				blast	acugaugcacuuggcuuucuacu	uagauuccuuuggcaucagacu	acugaugcacuuggcuuucuacuucagaguacagguggguagauuccuuuggcaucagacu	gi|307603372|gb|CM001004.1|:83888504..8388
gi|307603381|gb|CM000995.1|_26400	1.4	0.28 ± 0.02		35	35	0	0	yes				blast	cuuggccaugauauagag	cccaagugcuaggau	cuuggccaugauauagaggugaagauggccuugaacuuuuaaccccccacuccaccucccaagugcuaggau	gi|307603381|gb|CM000995.1|:177965624..177
gi|307603382|gb|CM000994.1|_21531	1.4	0.28 ± 0.02		30	30	0	0	yes				blast	ucagucccaaagcacccugc	aggagugugccuuggggcugaaa	aggagugugccuuggggcugaaaggccaugcaaacacaacaaacuaauaacucagucccaaagcacccugc	gi|307603382|gb|CM000994.1|:36162944..3616
gi|307603366|gb|CM001010.1|_16032	1.4	0.28 ± 0.02		29	29	0	0	yes				blast	cauuggauguuauucugua	caagacaugaaauccaacagg	cauuggauguuauucuguaucuagcaucugcacaagacaugaaauccaacagg	gi|307603366|gb|CM001010.1|:3238756..32388
gi|307603377|gb|CM000999.1|_37696	1.4	0.28 ± 0.02		33	33	0	0	yes				blast	uucccacucacuucgguucucc	aggaggaggugaaggggaaga	uucccacucacuucgguucuccagaagcacagaggaggaggaggugaaggggaaga	gi|307603377|gb|CM000999.1|:113654398..113
gi|307603369|gb|CM001007.1|_12424	1.4	0.28 ± 0.02		31	31	0	0	yes				blast	ucuguacugucuuggugagcuaga	uauucacaauguuuagugcauuug	uauucacaauguuuagugcauuuguauuuggcaacuuugaagaaagugcucuacaucuguacugucuuggugagcuaga	gi|307603369|gb|CM001007.1|:99493808..9949
gi|307603368|gb|CM001008.1|_13331	1.4	0.28 ± 0.02		38	38	0	0	yes				blast	auugaggcuagccugggcu	cucaagguuagccuugguua	cucaagguuagccuugguuauacaguggaaauugaggcuagccugggcu	gi|307603368|gb|CM001008.1|:99790676..9979
gi|307603370|gb|CM001006.1|_10502	1.4	0.28 ± 0.02		33	33	0	0	yes				blast	agaauuucggcugucugaagaaa	uuaugaagcagccucugg	uuaugaagcagccucugguguugcaaaucagaauuucggcugucugaagaaa	gi|307603370|gb|CM001006.1|:100123617..100
gi|307603375|gb|CM001001.1|_44105	1.4	0.28 ± 0.02		29	29	0	0	yes				blast	gcucuucaugcagacugaauu	cuuggugugaugauaagggcug	gcucuucaugcagacugaauuaacugucuggaaggauucuagacagaaaggaaaucuuggugugaugauaagggcug	gi|307603375|gb|CM001001.1|:17324013..1732
gi|307603373|gb|CM001003.1|_1761	1.4	0.28 ± 0.02		53	53	0	0	yes				blast	gcugauaccugggauauaa	guaucccagcagucagcau	gcugauaccugggauauaacacuuggcauuugucagucauuuaguuucuuugagacagggucugacuguguaucccagcagucagcau	gi|307603373|gb|CM001003.1|:75336197..7533
gi|307603374|gb|CM001002.1|_47554	1.4	0.28 ± 0.02		81	50	31	0	yes				blast	agggcgaccgagagaagg	uccucucagucaccuucu	uccucucagucaccuucugagccacaaauagaaccagagggcgaccgagagaagg	gi|307603374|gb|CM001002.1|:83341475..8334
gi|307603376|gb|CM001000.1|_42395	1.4	0.28 ± 0.02		438	438	0	0	yes				blast	ugacuccuggcucacacuggca	ccagguggagcggggagaaaac	ugacuccuggcucacacuggcaaacuucuugggccuuccagguggagcggggagaaaac	gi|307603376|gb|CM001000.1|:123289646..123
gi|307603368|gb|CM001008.1|_13976	1.4	0.28 ± 0.02		30	30	0	0	yes				blast	aacuuugcaggaauggcu	caagcugauagaguuug	caagcugauagaguuugggauuccugcuuaggguggaaagccauauuaggcagucaagaacuuugcaggaauggcu	gi|307603368|gb|CM001008.1|:80465230..8046
gi|307603374|gb|CM001002.1|_48017	1.4	0.28 ± 0.02		35	35	0	0	yes				blast	uugagaaccgcgcauccgua	cgcaccgaugugugcagcagcc	uugagaaccgcgcauccguacacauguuccugcucgauacacacgcgaccuuuacaugugugcgcaccgaugugugcagcagcc	gi|307603374|gb|CM001002.1|:121629024..121
gi|307603371|gb|CM001005.1|_8185	1.4	0.28 ± 0.02		29	29	0	0	yes				blast	agaagagguuuugcacagcaga	agcauguaaagaaaucugaucagc	agaagagguuuugcacagcagacacagcauguaaagaaaucugaucagc	gi|307603371|gb|CM001005.1|:96935395..9693
gi|307603365|gb|CM001011.1|_18907	1.4	0.28 ± 0.02		28	28	0	0	yes				blast	gcugggcugagucuuaagg	uuauauagguccagucc	uuauauagguccagucccugcuaucaauggagcauugaagaaagcugggcugagucuuaagg	gi|307603365|gb|CM001011.1|:74960942..7496
gi|307603370|gb|CM001006.1|_9833	1.4	0.28 ± 0.02		42	42	0	0	yes				blast	cagucuaggggcuccuugugaa	ccauggaaauccugggcucug	cagucuaggggcuccuugugaaccaggaaaucccaugaaaugauccuuucuccuagguuccauggaaauccugggcucug	gi|307603370|gb|CM001006.1|:28953978..2895
gi|307603373|gb|CM001003.1|_1962	1.4	0.28 ± 0.02		82	82	0	0	yes				blast	ucccagguuucggcacca	guaaccguccuggguuu	guaaccguccuggguuuucugucccaggauuucugucccaggucuucucgucccagguuucggcacca	gi|307603373|gb|CM001003.1|:82402311..8240
gi|307603375|gb|CM001001.1|_44696	1.4	0.28 ± 0.02		720	720	0	0	yes				blast	ucuguguccuugucccugaagc	ugaagggcaaggaucucacuucug	ugaagggcaaggaucucacuucugaaaccugcucuguguccuugucccugaagc	gi|307603375|gb|CM001001.1|:87445130..8744
gi|307603380|gb|CM000996.1|_28923	1.4	0.28 ± 0.02		32	32	0	0	yes				blast	ugaggaggcccuggcagcu	cuccgcucccuucau	cuccgcucccuucaucccggaugcucugaggaggcccuggcagcu	gi|307603380|gb|CM000996.1|:94288669..9428
gi|307603378|gb|CM000998.1|_34530	1.4	0.28 ± 0.02		162	162	0	0	yes				blast	uggcuguugggagaccgc	uuuccucccaacauucagg	uggcuguugggagaccgcaagacugaagucccucagccucuucacugcgccuccagucuuuuccucccaacauucagg	gi|307603378|gb|CM000998.1|:93334598..9333
gi|307603365|gb|CM001011.1|_19597	1.3	0.28 ± 0.02		38	38	0	0	yes				blast	aggaugggcuauagagugag	cacucuaguacauccuca	aggaugggcuauagagugagacacucuaguacauccuca	gi|307603365|gb|CM001011.1|:67931011..6793
gi|307603378|gb|CM000998.1|_35574	1.3	0.28 ± 0.02		30	30	0	0	yes				blast	uguaugugugcucagugcugaa	uagcaccccagugucuacauauuuc	uagcaccccagugucuacauauuucucuaauguaugugugcucagugcugaa	gi|307603378|gb|CM000998.1|:31254453..3125
gi|307603372|gb|CM001004.1|_4476	1.3	0.28 ± 0.02		2561	2561	0	0	yes				blast	agguuauagaaauuucagacaaua	aauuuugaagugguuugaggccuug	aauuuugaagugguuugaggccuugcccugcccucuccagagcaagguuauagaaauuucagacaaua	gi|307603372|gb|CM001004.1|:6428035..64281
gi|307603365|gb|CM001011.1|_19598	1.3	0.28 ± 0.02		38	38	0	0	yes				blast	aggaugggcuauagagugag	cacucuaguacauccuca	aggaugggcuauagagugagacacucuaguacauccuca	gi|307603365|gb|CM001011.1|:67931011..6793
gi|307603367|gb|CM001009.1|_14976	1.3	0.28 ± 0.02		339	339	0	0	yes				blast	ugauccacaguugucuuaugacc	ucauggcaaguguggacccacu	ugauccacaguugucuuaugaccaucacaugugggucauggcaaguguggacccacu	gi|307603367|gb|CM001009.1|:52203572..5220
gi|307603379|gb|CM000997.1|_32442	1.3	0.28 ± 0.02		30	30	0	0	yes				blast	gauguguucugucucuugaacc	uggaggccagaguccauucu	uggaggccagaguccauucuuagguaauuuuaggauguguucugucucuugaacc	gi|307603379|gb|CM000997.1|:32897290..3289
gi|307603379|gb|CM000997.1|_31462	1.3	0.28 ± 0.02		28	28	0	0	yes				blast	gccuuggaguagacagacug	gucuuuuaauccaagugg	gccuuggaguagacagacuguuagucuuuuaauccaagugg	gi|307603379|gb|CM000997.1|:98860513..9886
gi|307603364|gb|CM001012.1|_21278	1.3	0.28 ± 0.02		158	158	0	0	yes				blast	gcagacugcuguugacag	gucaacauggagucugaca	gucaacauggagucugacaacccuccacucuuugagugacaccuacuaugugcagacugcuguugacag	gi|307603364|gb|CM001012.1|:53409922..5340
gi|307603374|gb|CM001002.1|_47557	1.3	0.28 ± 0.02		32	32	0	0	yes				blast	cuuugugguuagagagagcauu	uguucucuugaagggccaggggcu	cuuugugguuagagagagcauucccacuaaucauuguucucuugaagggccaggggcu	gi|307603374|gb|CM001002.1|:83499895..8349

gi|307603365|gb|CM001011.1|_18705	1.3	0.28 ± 0.02		34	34	0	0	yes				blast	aggggaugaaagaaugggg	ugacauuugauuucuccaac	ugacauuugauuucuccaacuaacuuaaggagggggaugaaagaaugggg	gi|307603365|gb|CM001011.1|:55150157..5515
gi|307603380|gb|CM000996.1|_29644	1.3	0.28 ± 0.02		34	34	0	0	yes				blast	gaaaggcuacuguagcagg	uggaaguguguuuuua	uggaaguguguuuuuaccucccucuuguuuguugaggcaggguagaaaggcuacuguagcagg	gi|307603380|gb|CM000996.1|:16833063..1683
gi|307603379|gb|CM000997.1|_31805	1.3	0.28 ± 0.02		34	34	0	0	yes				blast	uuaauuagauuaagccgugauu	ucaccccuuaaucuaauuaguu	ucaccccuuaaucuaauuaguuuggaagcuauuuuaaagagcuuaauuagauuaagccgugauu	gi|307603379|gb|CM000997.1|:127982904..127
gi|307603370|gb|CM001006.1|_10367	1.3	0.28 ± 0.02		33	33	0	0	yes				blast	cugcccgcuguggaugcuugga	uuuugcaagagcagcaaguguu	cugcccgcuguggaugcuuggaccuauauucaggucuuuugcaagagcagcaaguguu	gi|307603370|gb|CM001006.1|:81667141..8166
gi|307603375|gb|CM001001.1|_43492	1.3	0.28 ± 0.02		32	32	0	0	yes				blast	gugucaaugagccacugaag	uauaguggcaauuucuggcacag	gugucaaugagccacugaagcuauuauaguggcaauuucuggcacag	gi|307603375|gb|CM001001.1|:91653937..9165
gi|307603372|gb|CM001004.1|_3474	1.3	0.28 ± 0.02		39	39	0	0	yes				blast	agcuguucugguugcuauguccug	agauauucgaugugggguagauga	agcuguucugguugcuauguccuguuuccgccaccucugugaagaagcagauauucgaugugggguagauga	gi|307603372|gb|CM001004.1|:79254450..7925
gi|307603375|gb|CM001001.1|_44578	1.3	0.28 ± 0.02		412	412	0	0	yes				blast	uguagaaggccucucuguacu	cauggagaaggccuucgccuauaga	cauggagaaggccuucgccuauagaguugccagcucaucuccuguagaaggccucucuguacu	gi|307603375|gb|CM001001.1|:76781517..7678
gi|307603378|gb|CM000998.1|_36599	1.3	0.28 ± 0.02		37	37	0	0	yes				blast	auggcuauaagggaucug	uuuuccuccagucuaugu	auggcuauaagggaucugaacuugguccgcacgcgugugcaacaaguguuuuccuccagucuaugu	gi|307603378|gb|CM000998.1|:138714573..138
gi|307603381|gb|CM000995.1|_27523	1.3	0.28 ± 0.02		33	33	0	0	yes				blast	accgcacugcuacagaaac	uaaaaugguagagacagugg	accgcacugcuacagaaacgucgugacauuuucugacaucguaaaaugguagagacagugg	gi|307603381|gb|CM000995.1|:118283653..118
gi|307603374|gb|CM001002.1|_47247	1.3	0.28 ± 0.02		45	45	0	0	yes				blast	gagauacagagcaagcauggc	cagggcugguuuuguguagaaa	gagauacagagcaagcauggcugacagggcugguuuuguguagaaa	gi|307603374|gb|CM001002.1|:59498763..5949
gi|307603363|gb|CM001013.1|_48287	1.3	0.28 ± 0.02		29	29	0	0	yes				blast	ggaagaccuugggcuccga	guuccucuuggucuuccug	guuccucuuggucuuccugcuugggaacgaggaagacuaagucuuuuccuauccgagauucuagaggaggaagaccuugggcuccga	gi|307603363|gb|CM001013.1|:45316721..4531
gi|307603368|gb|CM001008.1|_14036	1.2	0.28 ± 0.02		50	50	0	0	yes				blast	acagggagggcaaaauuc	aaccagcuuucuuuucuggau	aaccagcuuucuuuucuggaucccucuacuguacacgugguacgauggaugacagggagggcaaaauuc	gi|307603368|gb|CM001008.1|:82086372..8208
gi|307603363|gb|CM001013.1|_48744	1.2	0.28 ± 0.02	rRNA/tRNA	13703	13703	0	0	yes				blast	ggggauguagcucagugg	ccugguucgauucccgg	ggggauguagcucagugguagagcacaugcuuugcauguaugggguccugguucgauucccgg	gi|307603363|gb|CM001013.1|:133088263..133
gi|307603379|gb|CM000997.1|_32797	1.2	0.28 ± 0.02		33	33	0	0	yes				blast	ugugucacugggggugggcuuugu	aaagcuaucaccacuccaguuucucucu	ugugucacugggggugggcuuugugguucaaaagcuaucaccacuccaguuucucucu	gi|307603379|gb|CM000997.1|:71673608..7167
gi|307603382|gb|CM000994.1|_22974	1.2	0.28 ± 0.02		254	254	0	0	yes				blast	cagcgugcucuacauagauagua	uuaaucgagcagagacaggcaaau	uuaaucgagcagagacaggcaaauccuuuuaacuucaaggucagcgugcucuacauagauagua	gi|307603382|gb|CM000994.1|:21380738..2138
gi|307603372|gb|CM001004.1|_5695	1.2	0.28 ± 0.02		39	39	0	0	yes				blast	acauggcuguuggacacucagg	uggcacccacacgcccauguuc	acauggcuguuggacacucaggcuaaaccauggcacccacacgcccauguuc	gi|307603372|gb|CM001004.1|:100775803..100
gi|307603370|gb|CM001006.1|_9627	1.2	0.28 ± 0.02		30	30	0	0	yes				blast	aaaauauuacuauggcugga	cagccaucaggugcuuugu	aaaauauuacuauggcuggaugucgccacuccagccaucaggugcuuugu	gi|307603370|gb|CM001006.1|:21314385..2131
gi|307603379|gb|CM000997.1|_30866	1.2	0.28 ± 0.02		30	30	0	0	yes				blast	agggagacuugguaauca	ucccccagcucucccugu	agggagacuugguaaucacucugcuuacuucccccagcucucccugu	gi|307603379|gb|CM000997.1|:7117557..71176
gi|307603373|gb|CM001003.1|_2240	1.2	0.28 ± 0.02		40	40	0	0	yes				blast	ccgaggaugugaggaauc	uucuccacgugucugggaa	uucuccacgugucugggaagggaaagggaaaaggaaucuuuuccuccgaggaugugaggaauc	gi|307603373|gb|CM001003.1|:118307069..118
gi|307603374|gb|CM001002.1|_47078	1.2	0.28 ± 0.02		202	202	0	0	yes				blast	agaagcuggguugagagggca	uauauuccacucaguguuguuga	uauauuccacucaguguuguugauuuauugcaugucccucacguuggaagacaugggugagguagaagcuggguugagagggca	gi|307603374|gb|CM001002.1|:48063757..4806
gi|307603371|gb|CM001005.1|_7717	1.2	0.28 ± 0.02		38	38	0	0	yes				blast	uugggguagcaguuagag	cuguuuccuggagu	uugggguagcaguuagaggggaguuggcuagaaccuuuccaacaggaaaagagcuuguaaaucucccuucuguuuccuggagu	gi|307603371|gb|CM001005.1|:35693020..3569
gi|307603378|gb|CM000998.1|_36318	1.2	0.28 ± 0.02		29	29	0	0	yes				blast	cacugugugauguggagga	cucugcaugacugag	cucugcaugacugagguuggggaaaagaaaaauagaaacaguccccaacucacugugugauguggagga	gi|307603378|gb|CM000998.1|:122904151..122
gi|307603373|gb|CM001003.1|_1349	1.2	0.28 ± 0.02		32	32	0	0	yes				blast	ugcuggucuugucagaaa	ucgacuugacccaaa	ugcuggucuugucagaaauugacugacauuguguuuuaacaagaaguggcucgacuugacccaaa	gi|307603373|gb|CM001003.1|:22424631..2242
gi|307603380|gb|CM000996.1|_28942	1.2	0.28 ± 0.02		250	250	0	0	yes				blast	aaugugucaggcuguuuuaaca	guaaaacagccuuuucacauaca	aaugugucaggcuguuuuaacaugccacauguaaaacagccuuuucacauaca	gi|307603380|gb|CM000996.1|:94787640..9478
gi|307603372|gb|CM001004.1|_4312	1.2	0.28 ± 0.02		196	196	0	0	yes				blast	cacaugaggggaggcugcu	cacccuccacucacauggc	cacaugaggggaggcugcucucacacccuccacucacauggc	gi|307603372|gb|CM001004.1|:119071171..119
gi|307603381|gb|CM000995.1|_24791	1.2	0.28 ± 0.02		36	36	0	0	yes				blast	ggccccugauucuaggua	ccccgaauccccuaggaggugau	ccccgaauccccuaggaggugaugucccuucagcccucccagccugggggcaccaggguguggccccugauucuaggua	gi|307603381|gb|CM000995.1|:25277155..2527
gi|307603368|gb|CM001008.1|_12662	1.2	0.28 ± 0.02		48	37	11	0	yes				blast	ugcugauuauggggguggauu	uccaucccuuucaucucagcucc	ugcugauuauggggguggauuccuggauauggcagucucuacauggucccaucccuuucaucucagcucc	gi|307603368|gb|CM001008.1|:28494408..2849
gi|307603363|gb|CM001013.1|_48930	1.2	0.28 ± 0.02		3617	3617	0	0	yes				blast	gaacgcagucugagugga	cacuuuucacugcucua	gaacgcagucugaguggauuucgucacacuuuucacugcucua	gi|307603363|gb|CM001013.1|:161522557..161
gi|307603380|gb|CM000996.1|_30215	1.2	0.28 ± 0.02		161	161	0	0	yes				blast	gagacugaaguggggaaagg	auuccaagccuuucuc	gagacugaaguggggaaaggagccaauuuauuccaagccuuucuc	gi|307603380|gb|CM000996.1|:94569326..9456
gi|307603374|gb|CM001002.1|_45735	1.2	0.28 ± 0.02		30	30	0	0	yes				blast	aaaaagagggggaaaggc	uguggucccccuucugcugaa	uguggucccccuucugcugaagccaagagagggccugcagaucacccacauaccaacucuagcaaaaagagggggaaaggc	gi|307603374|gb|CM001002.1|:45470480..4547
gi|307603375|gb|CM001001.1|_44840	1.2	0.28 ± 0.02		294	294	0	0	yes				blast	uaacucaugaaaacaagauucugg	aauccuguuuuuguguggguugcu	aauccuguuuuuguguggguugcuguguuuuugguauuaguaacucaugaaaacaagauucugg	gi|307603375|gb|CM001001.1|:101891407..101
gi|307603371|gb|CM001005.1|_6996	1.2	0.28 ± 0.02		28	28	0	0	yes				blast	aggacagugagugauucaua	agaguagaucagugucuuag	aggacagugagugauucauagagaguagaucagugucuuag	gi|307603371|gb|CM001005.1|:91073298..9107
gi|307603370|gb|CM001006.1|_9296	1.2	0.28 ± 0.02		204189	204189	0	0	yes				blast	aucucgcuggggccucca	aaggccuuaguauugagugug	aucucgcuggggccuccaauguaagacccuaaggccuuaguauugagugug	gi|307603370|gb|CM001006.1|:93321112..9332
gi|307603373|gb|CM001003.1|_1519	1.1	0.28 ± 0.02		356	356	0	0	yes				blast	uggaugugugaugagaugaggc	cucaccacuucacacauuaaag	uggaugugugaugagaugaggcugugcuuucaagggcucaccacuucacacauuaaag	gi|307603373|gb|CM001003.1|:42835743..4283
gi|307603381|gb|CM000995.1|_25781	1.1	0.28 ± 0.02		46	46	0	0	yes				blast	uugucuggccucagugugaga	ucagaggcccagggcacaua	ucagaggcccagggcacauauguagcaaaagacuguugucuggccucagugugaga	gi|307603381|gb|CM000995.1|:126999315..126
gi|307603374|gb|CM001002.1|_47730	1.1	0.28 ± 0.02		32	32	0	0	yes				blast	uaguggucuuuccuguuggaa	cugggaggagacacauugca	cugggaggagacacauugcaggugacacugagguaguggucuuuccuguuggaa	gi|307603374|gb|CM001002.1|:102233600..102
gi|307603363|gb|CM001013.1|_48634	1.1	0.28 ± 0.02		127	127	0	0	yes				blast	gagcgguuugcaguaaggagcug	gcuaaccuuucccuguuuuuuguuuuu	gagcgguuugcaguaaggagcugcggcagcccagagucugcucuuuuugggcuggcuaaccuuucccuguuuuuuguuuuu	gi|307603363|gb|CM001013.1|:104116113..104
gi|307603379|gb|CM000997.1|_32127	1.1	0.28 ± 0.02		35	35	0	0	yes				blast	gcugcuuggaccagagag	cucuggguugagacaggca	cucuggguugagacaggcacaucaugacacaaagcagcaugcuuaguguuggcagaacagcugcuuggaccagagag	gi|307603379|gb|CM000997.1|:148680295..148
gi|307603375|gb|CM001001.1|_42981	1.1	0.28 ± 0.02		1654	1654	0	0	yes				blast	uguaguccuggcuguccuggaa	uuauacuuagcuucccucuacaca	uuauacuuagcuucccucuacacagguuucucuguguaguccuggcuguccuggaa	gi|307603375|gb|CM001001.1|:23831474..2383
gi|307603366|gb|CM001010.1|_17326	1.1	0.28 ± 0.02		146	146	0	0	yes				blast	gauccuuccccagccaga	uggcaggggagggagagg	uggcaggggagggagaggaagaagcgggggaaggagacgucagauccuuccccagccaga	gi|307603366|gb|CM001010.1|:15843118..1584
gi|307603376|gb|CM001000.1|_40558	1.1	0.28 ± 0.02		28	28	0	0	yes				blast	aaagaaggagcagagaaca	gacuuugacuccuuggaaga	aaagaaggagcagagaacacugaguuuguggacuuugacuccuuggaaga	gi|307603376|gb|CM001000.1|:129743161..129
gi|307603372|gb|CM001004.1|_4796	1.1	0.28 ± 0.02		88	88	0	0	yes				blast	uaugaguagacacagggacua	guucuguguuaggacaucauuuu	guucuguguuaggacaucauuuuaggguuauugauaugaguagacacagggacua	gi|307603372|gb|CM001004.1|:50625481..5062
gi|307603371|gb|CM001005.1|_6721	1.0	0.28 ± 0.02		32	32	0	0	yes				blast	ugacuggcugggccugcugc	agcagcuuccuacacaguagga	agcagcuuccuacacaguaggagacugccacauuuuuuuccuaucaugacuggcugggccugcugc	gi|307603371|gb|CM001005.1|:73895422..7389
gi|307603371|gb|CM001005.1|_8287	1.0	0.28 ± 0.02		96	96	0	0	yes				blast	aucacacugggugugagcuuga	aagcugcccuccauggugacac	aucacacugggugugagcuugagauauaugagacuucaaagcugcccuccauggugacac	gi|307603371|gb|CM001005.1|:105983398..105
gi|307603365|gb|CM001011.1|_18691	1.0	0.28 ± 0.02		172	172	0	0	yes				blast	cuggaaacugacagaggagc	uuuuuuucuucaguuuuuacug	uuuuuuucuucaguuuuuacuguggccuacuuugagaacuguuucuggaaacugacagaggagc	gi|307603365|gb|CM001011.1|:51631682..5163
gi|307603373|gb|CM001003.1|_239	1.0	0.28 ± 0.02		15725	15509	0	216	yes				blast	aaguuucucugacuguguagag	cagggaucauuucuauagu	cagggaucauuucuauaguuuguuacuagagaaguuucucugacuguguagag	gi|307603373|gb|CM001003.1|:39978285..3997
gi|307603374|gb|CM001002.1|_47914	1.0	0.28 ± 0.02		29	29	0	0	yes				blast	agagacuuguguguccugcugaag	gaggcagaacacacagucacugc	gaggcagaacacacagucacugcugagaugacaagaggcucugagguaguguggaucagaaacaaaggcagagacuuguguguccugcugaag	gi|307603374|gb|CM001002.1|:110196516..110
gi|307603373|gb|CM001003.1|_1391	1.0	0.28 ± 0.02		32	32	0	0	yes				blast	uucucccuugacaacuua	aguccucagggaggugg	uucucccuugacaacuuacguagcaucuucaaacacuaugagaguuaguuagucccucagggaggugg	gi|307603373|gb|CM001003.1|:29855446..2985
gi|307603371|gb|CM001005.1|_7386	1.0	0.28 ± 0.02		1021	984	37	0	yes				blast	uugcaagcaacacucuguggcag	gacacaauuugagcuugcuaua	uugcaagcaacacucuguggcagaugaacaaaaccgucugacacaauuugagcuugcuaua	gi|307603371|gb|CM001005.1|:112779147..112
gi|307603366|gb|CM001010.1|_16637	1.0	0.28 ± 0.02		33	33	0	0	yes				blast	caugauuguauggugagcagg	ugaccuuagugugcauucugag	ugaccuuagugugcauucugaggcucaaacucaagagcucaugauuguauggugagcagg	gi|307603366|gb|CM001010.1|:37143947..3714
gi|307603364|gb|CM001012.1|_20885	1.0	0.28 ± 0.02		43	43	0	0	yes				blast	uggauuuauggaguggug	ccgcucacuuagaaguuug	uggauuuauggaguggugcguuugccuaucgucuggauacagacuucuuaaaauaugccgcucacuuagaaguuug	gi|307603364|gb|CM001012.1|:12080311..1208
gi|307603381|gb|CM000995.1|_27912	1.0	0.28 ± 0.02		33	33	0	0	yes				blast	guucuaacccgggauuugg	cuauccuuguuaaagcac	cuauccuuguuaaagcacaagcuuuauucgaggcccauguguucuaacccgggauuugg	gi|307603381|gb|CM000995.1|:153271712..153
gi|307603381|gb|CM000995.1|_26847	0.9	0.00 ± 0.00		39	39	0	0	yes				blast	uaugcacauguagucucuaua	uaggcucuauaugcacaugua	uaugcacauguagucucuauauauacauguaggcucuauauacacauaucagcucuauaugcacauguaggcucuauaugcacaugua	gi|307603381|gb|CM000995.1|:33194665..3319
gi|307603366|gb|CM001010.1|_17616	0.9	0.00 ± 0.00		47	47	0	0	yes				blast	cccaacucccuuagaacug	ggggaguaacagucacccugguu	ggggaguaacagucacccugguucgugaauuauccggacccaacucccuuagaacug	gi|307603366|gb|CM001010.1|:33102650..3310
gi|307603378|gb|CM000998.1|_34363	0.9	0.00 ± 0.00		138	138	0	0	yes				blast	acaagugacuccuggggccugca	aagagccuuggagggacaauguag	aagagccuuggagggacaauguagccacaccucacaagugacuccuggggccugca	gi|307603378|gb|CM000998.1|:66865064..6686
gi|307603378|gb|CM000998.1|_35303	0.9	0.00 ± 0.00	rRNA/tRNA	13583	13583	0	0	yes				blast	ggggauguagcucagugg	acugguuauucag	acugguuauucagugcuuuggugaauacacaauaauacuaaagggcuggggauguagcucagugg	gi|307603378|gb|CM000998.1|:145046661..145
gi|307603372|gb|CM001004.1|_4460	0.9	0.00 ± 0.00		61	61	0	0	yes				blast	gugcccaggacagcuguu	cuguccaccuacag	cuguccaccuacagacacucaguggauaccacccuuucauccacacugugcccaggacagcuguu	gi|307603372|gb|CM001004.1|:5870124..58701
gi|307603372|gb|CM001004.1|_5470	0.9	0.00 ± 0.00		30	30	0	0	yes				blast	cacaucuuguugccuguccga	aggcagagcagggaggugggug	aggcagagcagggagguggguggcuguagaauacgaugcacacaucuuguugccuguccga	gi|307603372|gb|CM001004.1|:88519439..8851
gi|307603366|gb|CM001010.1|_16815	0.9	0.00 ± 0.00		28	28	0	0	yes				blast	ccucugaggauaacaggca	acuccaguuccaggggau	acuccaguuccaggggaucggauacacucuucuggccucugaggauaacaggca	gi|307603366|gb|CM001010.1|:47956045..4795
gi|307603379|gb|CM000997.1|_32714	0.9	0.00 ± 0.00		29	29	0	0	yes				blast	auguguguacgugcauguagac	guggauguguguacacagauac	guggauguguguacacagauacacaugcauauguguguacgugcauguagac	gi|307603379|gb|CM000997.1|:58465382..5846
gi|307603375|gb|CM001001.1|_43349	0.9	0.00 ± 0.00		32	32	0	0	yes				blast	cuucguguaguguuguaaccuu	guuuagugcacuauacgaaagug	guuuagugcacuauacgaaaguggccucgggagaaaauaacuucguguaguguuguaaccuu	gi|307603375|gb|CM001001.1|:75073137..7507
gi|307603376|gb|CM001000.1|_41344	0.9	0.00 ± 0.00		7815	7811	0	4	yes				blast	cgaggacaauauauuaaaauggauu	uuaguaucugauauguccu	uuaguaucugauauguccucuguccgaggacaauauauuaaaauggauu	gi|307603376|gb|CM001000.1|:31141842..3114
gi|307603370|gb|CM001006.1|_9053	0.9	0.00 ± 0.00		28	28	0	0	yes				blast	guguaagaagcguaugggcccuu	ugguccucacguuucugucagcug	ugguccucacguuucugucagcugaucugccaguguaagaagcguaugggcccuu	gi|307603370|gb|CM001006.1|:59183316..5918
gi|307603368|gb|CM001008.1|_14114	0.9	0.00 ± 0.00		30	30	0	0	yes				blast	guacugggacacagcugaguuu	gccccagcugcagcccaguucucaa	gccccagcugcagcccaguucucaaaaaaaacauuguacugggacacagcugaguuu	gi|307603368|gb|CM001008.1|:88119603..8811
gi|307603378|gb|CM000998.1|_34201	0.9	0.00 ± 0.00		32	32	0	0	yes				blast	agaacuuacaggacauuaagacgg	gcucaugugccuuuuuucuuc	gcucaugugccuuuuuucuuccucuccucagauucgagagcagaacuuacaggacauuaagacgg	gi|307603378|gb|CM000998.1|:34962662..3496
gi|307603365|gb|CM001011.1|_19522	0.9	0.00 ± 0.00		30	30	0	0	yes				blast	accgaauugcacucagagc	ucugagugagucggggc	ucugagugagucggggccuuucucagguccacacucaccgaauugcacucagagc	gi|307603365|gb|CM001011.1|:60990349..6099
gi|307603381|gb|CM000995.1|_26481	0.9	0.00 ± 0.00		255	255	0	0	yes				blast	aaaggcagauacccagggcu	ucaauguuauguucuuuugc	ucaauguuauguucuuuugcugacaaaggcagauacccagggcu	gi|307603381|gb|CM000995.1|:181502051..181
gi|307603380|gb|CM000996.1|_28983	0.9	0.00 ± 0.00		41	41	0	0	yes				blast	auaaaaaaggaggaugga	caugcuccgcccguacau	caugcuccgcccguacaugcggcuccucgggugauaaaaaaggaggaugga	gi|307603380|gb|CM000996.1|:95686366..9568
gi|307603367|gb|CM001009.1|_14835	0.8	0.00 ± 0.00		30	30	0	0	yes				blast	guggguuucugaauuucuauuaga	ucuuggacagaaucaacucacua	ucuuggacagaaucaacucacuauuucuuuccagagugggguuucugaauuucuauuaga	gi|307603367|gb|CM001009.1|:36075912..3607
gi|307603379|gb|CM000997.1|_32940	0.8	0.00 ± 0.00		38	38	0	0	yes				blast	gaagugcauggcugucugua	aagcuguugugcaggucc	aagcuguugugcaggucccagcuauuccaugucuucauggcagaugagaagugcauggcugucugua	gi|307603379|gb|CM000997.1|:94132144..9413
gi|307603381|gb|CM000995.1|_27160	0.8	0.00 ± 0.00		28	28	0	0	yes				blast	gauuuagugaccugauugaagaug	uuuuuuucaggucuuaauucu	uuuuuuucaggucuuaauucugaaggacucuaccgagugucaggauuuagugaccugauugaagaug	gi|307603381|gb|CM000995.1|:73462192..7346
gi|307603380|gb|CM000996.1|_29596	0.8	0.00 ± 0.00		96	96	0	0	yes				blast	aucuugguggaaccucca	ggguuccugggguguca	ggguuccugggugucaugcagaucuugguggaaccucca	gi|307603380|gb|CM000996.1|:3339389..33394
gi|307603382|gb|CM000994.1|_23031	0.8	0.00 ± 0.00		30	30	0	0	yes				blast	cuacuugcucuggcuucuga	aggagaagaggccgucucc	cuacuugcucuggcuucugaauugggaucacaaugauacugaggcaagugaugugauuacucauucaggagaagaggccgucucc	gi|307603382|gb|CM000994.1|:30997653..3099
gi|307603378|gb|CM000998.1|_34224	0.8	0.00 ± 0.00		29	29	0	0	yes				blast	auuaugacucugaugcca	gcaguuccuaugagucauaugau	auuaugacucugaugccaugagcaguuccuaugagucauaugau	gi|307603378|gb|CM000998.1|:36290683..3629
gi|307603368|gb|CM001008.1|_12732	0.8	0.00 ± 0.00		47	47	0	0	yes				blast	ccugggacaagagcuguuagg	uaccaguguaugcugaguuccagggc	ccugggacaagagcuguuaggaucugcucaguauuagguuuuaccaguguaugcugaguuccagggc	gi|307603368|gb|CM001008.1|:39258772..3925
gi|307603372|gb|CM001004.1|_4459	0.8	0.00 ± 0.00		61	61	0	0	yes				blast	gugcccaggacagcuguu	caucugccacccauggacacag	gugcccaggacagcuguucccuacccauggacacagggguaaacaucugccacccauggacacag	gi|307603372|gb|CM001004.1|:5870078..58701
gi|307603381|gb|CM000995.1|_26855	0.8	0.00 ± 0.00		32	32	0	0	yes				blast	gucucauagagccagagc	cccagccuauccaggcug	gucucauagagccagagcuguacccacagcccagccuauccaggcug	gi|307603381|gb|CM000995.1|:33883030..3388
gi|307603364|gb|CM001012.1|_20493	0.8	0.00 ± 0.00		56	56	0	0	yes				blast	ugucugacuaucuaccauggu	caugguguacacuguccgacuau	caugguguacacuguccgacuaucuaccaugguauacacuguccgacuaucuaaccaugguguacacugucugacuaucuaccauggu	gi|307603364|gb|CM001012.1|:55603348..5560
gi|307603378|gb|CM000998.1|_35064	0.8	0.00 ± 0.00		36	36	0	0	yes				blast	aggaugaggagcccagca	ucacagcucccucuuguccuugu	aggaugaggagcccagcagggacacaguucugucacagcucccucuuguccuugu	gi|307603378|gb|CM000998.1|:130611255..130
gi|307603368|gb|CM001008.1|_13746	0.8	0.00 ± 0.00		32	32	0	0	yes				blast	aacauaauaaggacuaaguggaa	ccacauuccuugagcgguaug	ccacauuccuugagcgguaugaggaaacauaauaaggacuaaguggaa	gi|307603368|gb|CM001008.1|:65916560..6591
gi|307603371|gb|CM001005.1|_8086	0.7	0.00 ± 0.00		39	39	0	0	yes				blast	uccgucagacagaguuugc	agacuguuccugaugcuaa	agacuguuccugaugcuaaguccugacuauuguucuuuucgucaacagaagcugauuucucaggcucuccgucagacagaguuugc	gi|307603371|gb|CM001005.1|:84200546..8420
gi|307603381|gb|CM000995.1|_26592	0.7	0.00 ± 0.00		33	33	0	0	yes				blast	gggaccaggauuuaguguc	uacaugaaaaucucccucaucccug	uacaugaaaaucucccucaucccugaggaucuccuugucaggggcaaccagauccuggggaccaggauuuaguguc	gi|307603381|gb|CM000995.1|:13886906..1388
gi|307603374|gb|CM001002.1|_46741	0.7	0.00 ± 0.00		34	34	0	0	yes				blast	uacucacucuauggaucuggaa	cuagauccagaaagugguaag	uacucacucuauggaucuggaaacaaugaauuuuuucuagauccagaaagugguaag	gi|307603374|gb|CM001002.1|:15794934..1579
gi|307603376|gb|CM001000.1|_41039	0.7	0.00 ± 0.00		46	46	0	0	yes				blast	ugagcaguucugucgguaga	ucuggacacaacucucacc	ugagcaguucugucgguagaauuugucuuuuacagauucucuggacacaacucucacc	gi|307603376|gb|CM001000.1|:13562269..1356
gi|307603366|gb|CM001010.1|_16163	0.7	0.00 ± 0.00		119	119	0	0	yes				blast	ucccaagcugucaccuuccugucu	acaggaugcagcuugacugaugaug	acaggaugcagcuugacugaugaugcacucagcaucccaagcugucaccuuccugucu	gi|307603366|gb|CM001010.1|:15480822..1548
gi|307603380|gb|CM000996.1|_29681	0.7	0.00 ± 0.00		30	30	0	0	yes				blast	uuaggggguccacugcuuc	agcagugucuauauccugagg	uuaggggguccacugcuucauucuuccagccaaguaugcagcagugucuauauccugagg	gi|307603380|gb|CM000996.1|:22994859..2299
gi|307603370|gb|CM001006.1|_8837	0.7	0.00 ± 0.00		44	44	0	0	yes				blast	cuuugaguguauggcuggcuag	ggcuggccaguacugaagagcu	ggcuggccaguacugaagagcuggcuuuuaguguauggguuggcuuugaguguauggcuggcuag	gi|307603370|gb|CM001006.1|:42205163..4220
gi|307603369|gb|CM001007.1|_11632	0.7	0.00 ± 0.00		109425	109425	0	0	yes				blast	aucucgguggaaccucca	gggguuccuggguguca	gggguuccugggugucacacagaucucgguggaaccucca	gi|307603369|gb|CM001007.1|:14765259..1476
gi|307603365|gb|CM001011.1|_18817	0.7	0.00 ± 0.00		41	41	0	0	yes				blast	caacugagcuuuagcacuag	agacccaaggcuuuguuuaugcu	agacccaaggcuuuguuuaugcuaaguaagcacuuuagcaacugagcuuuagcacuag	gi|307603365|gb|CM001011.1|:65055372..6505
gi|307603372|gb|CM001004.1|_3439	0.7	0.00 ± 0.00		36	36	0	0	yes				blast	cugcucccagaauccuggu	ugucugucugugaggaguggcc	ugucugucugugaggaguggccucgcugcucccagaauccuggu	gi|307603372|gb|CM001004.1|:77873065..7787
gi|307603372|gb|CM001004.1|_5510	0.7	0.00 ± 0.00		42	42	0	0	yes				blast	acgugucccccagcugaaggc	cgucagcuagacucggac	cgucagcuagacucggacacucccaggcccuguuccacacagaugcugggugggcacgugucccccagcugaaggc	gi|307603372|gb|CM001004.1|:94107835..9410
gi|307603371|gb|CM001005.1|_8067	0.7	0.00 ± 0.00		37	37	0	0	yes				blast	guuggaguucugaagcagga	auuucucagggaaccaaccu	guuggaguucugaagcaggagguaacucauggcucucauguucucccagagagcaguaaguuauuucucagggaaccaaccu	gi|307603371|gb|CM001005.1|:81852607..8185
gi|307603379|gb|CM000997.1|_33690	0.7	0.00 ± 0.00		31	31	0	0	yes				blast	gaguuuggcuggacccuc	aucuccuacgcccaaggcuuua	aucuccuacgcccaaggcuuuaugcugcucagacaggcagccacugaguuuggcuggacccuc	gi|307603379|gb|CM000997.1|:148528022..148
gi|307603363|gb|CM001013.1|_49080	0.6	0.00 ± 0.00		34	34	0	0	yes				blast	aggucuaguuaagcacuga	aguguaggggccuaa	aguguaggggccuaaguuuaacagauuuaggucuaguuaagcacuga	gi|307603363|gb|CM001013.1|:20375283..2037
gi|307603380|gb|CM000996.1|_29577	0.6	0.00 ± 0.00		29	29	0	0	yes				blast	ugaacuugacuggagcccaga	ugggauaguggaggaauc	ugggauaguggaggaaucagguuucucuggagaauaugcaagaagaaauauauagaugaacuugacuggagcccaga	gi|307603380|gb|CM000996.1|:156315976..156
gi|307603381|gb|CM000995.1|_25348	0.6	0.00 ± 0.00		29	29	0	0	yes				blast	augcgcugagcucucggug	ccagguuuagaagauuug	augcgcugagcucucggugcuuuguucucuguagagcauacggcuaauucagaaccagguuuagaagauuug	gi|307603381|gb|CM000995.1|:78535232..7853
gi|307603373|gb|CM001003.1|_2332	0.6	0.00 ± 0.00		44	44	0	0	yes				blast	guuguugcggagccuugagg	uagccccgccagcuccac	uagccccgccagcuccacccagcuugcuuucucccugcaguuccagauccaaaacaggcuggaaguuguugcggagccuugagg	gi|307603373|gb|CM001003.1|:126951870..126
gi|307603363|gb|CM001013.1|_48136	0.6	0.00 ± 0.00		35	35	0	0	yes				blast	guauugaaccuucuguggaa	ucauggauuuggcucucagugcuc	guauugaaccuucuguggaaucuagcucacagugaugaugugccuguagauaucauggauuuggcucucagugcuc	gi|307603363|gb|CM001013.1|:12690784..1269
gi|307603382|gb|CM000994.1|_23603	0.6	0.00 ± 0.00		47	47	0	0	yes				blast	gguagggcuguaagguuca	ccccuucagccuggcccug	gguagggcuguaagguucauguggagcuaccuucuccuaucccccccuucagccuggcccug	gi|307603382|gb|CM000994.1|:94914567..9491
gi|307603376|gb|CM001000.1|_40259	0.6	0.00 ± 0.00		38	38	0	0	yes				blast	auguuuaaugggcucauuuucuug	ggaaaauuagcucuaccuuacaugg	ggaaaauuagcucuaccuuacaugggugcuucugggcacagaauguuuaaugggcucauuuucuug	gi|307603376|gb|CM001000.1|:97177414..9717
gi|307603372|gb|CM001004.1|_3333	0.6	0.00 ± 0.00		30	30	0	0	yes				blast	cggaaccugugaaggcgaa	gguccucagaugucugua	gguccucagaugucuguauugaacauggguggugagccggaacccuggcaacacggaaccugugaaggcgaa	gi|307603372|gb|CM001004.1|:74719383..7471
gi|307603368|gb|CM001008.1|_13056	0.6	0.00 ± 0.00	rRNA	15527	15525	2	0	yes				blast	gcuaagcagggucgggccuggu	ugggugcuguaugcuuaaaac	gcuaagcagggucgggccugguuaguauucggaugggagaccaacuggaauacugggugcuguaugcuuaaaac	gi|307603368|gb|CM001008.1|:82173132..8217
gi|307603377|gb|CM000999.1|_38970	0.6	0.00 ± 0.00		35	35	0	0	yes				blast	gugguaugcaugaaugagca	accuagcguacuaaaacugg	accuagcguacuaaaacuggguccauugaggccugcaagcauccucaaaguggcccgugguaugcaugaaugagca	gi|307603377|gb|CM000999.1|:116214964..116
gi|307603378|gb|CM000998.1|_36440	0.6	0.00 ± 0.00		862	862	0	0	yes				blast	ugugugugugugguaugugu	gggugggagcacaugggcc	ugugugugugugguauguguuagaggaugagggugggagcacaugggcc	gi|307603378|gb|CM000998.1|:131693965..131
gi|307603373|gb|CM001003.1|_2041	0.5	0.00 ± 0.00		30	30	0	0	yes				blast	auccugccuuguguucugcuaccc	auagcaaaugcagaggggaugc	auccugccuuguguucugcuacccccauuuuauugcggauagcaaaugcagaggggaugc	gi|307603373|gb|CM001003.1|:91599465..9159
gi|307603364|gb|CM001012.1|_20045	0.5	0.00 ± 0.00		219	219	0	0	yes				blast	cacucaguaagggauagua	cuagccucugccuuuguc	cuagccucugccuuugucccuuuuauuuguggggcacucaguaagggauagua	gi|307603364|gb|CM001012.1|:17690062..1769
gi|307603365|gb|CM001011.1|_19327	0.5	0.00 ± 0.00		34	34	0	0	yes				blast	aagaagucaagcgucugcugagua	cucguugggagauaauugcugaagau	aagaagucaagcgucugcugaguacagaugcagaagcugucaguacucguugggagauaauugcugaagau	gi|307603365|gb|CM001011.1|:37802605..3780
gi|307603365|gb|CM001011.1|_18839	0.5	0.00 ± 0.00		38	38	0	0	yes				blast	acagauacugggagaaaa	guuucccaauuagucc	guuucccaauuaguccaguuaaagcccacugguugcaagugguacagauacugggagaaaa	gi|307603365|gb|CM001011.1|:66173550..6617
gi|307603378|gb|CM000998.1|_35972	0.5	0.00 ± 0.00		31	31	0	0	yes				blast	uaugcugugugaaugggga	ccuuucuucuuuacaguauggc	ccuuucuucuuuacaguauggccacauaugcugugugaaugggga	gi|307603378|gb|CM000998.1|:99876392..9987
gi|307603381|gb|CM000995.1|_25397	0.5	0.00 ± 0.00		36	36	0	0	yes				blast	agucucuuaacuuccuua	gcggaaagggauggagccuug	gcggaaagggauggagccuugcaaacagcucagcuguucaacauuucugagguugagcuugcacaagucucuuaacuuccuua	gi|307603381|gb|CM000995.1|:84978085..8497
gi|307603378|gb|CM000998.1|_34487	0.5	0.00 ± 0.00		31	31	0	0	yes				blast	uuugggaaauugcugauu	uuguaaaacccagaau	uuguaaaacccagaauauuuugggaaauugcugauu	gi|307603378|gb|CM000998.1|:87256102..8725
gi|307603366|gb|CM001010.1|_17648	0.5	0.00 ± 0.00		29	29	0	0	yes				blast	augaauagccaccugagug	cucaaggauggugcuaguauac	cucaaggauggugcuaguauacacuagauuugauaguuaugaauagccaccugagug	gi|307603366|gb|CM001010.1|:33957772..3395
gi|307603378|gb|CM000998.1|_34468	0.5	0.00 ± 0.00		157	157	0	0	yes				blast	augcucugcucaauaauacaaga	uuguuaauguacagugcaagu	augcucugcucaauaauacaagaugcuccuucugaucuuguuaauguacagugcaagu	gi|307603378|gb|CM000998.1|:82095195..8209
gi|307603377|gb|CM000999.1|_38919	0.5	0.00 ± 0.00		29	29	0	0	yes				blast	gaaaccgaggcuaaagagauu	acucuuuacuuggaauuuc	gaaaccgaggcuaaagagauucuucucccccauucccgucuugcacaacacucuuuacuuggaauuuc	gi|307603377|gb|CM000999.1|:112995986..112
gi|307603380|gb|CM000996.1|_28927	0.4	0.00 ± 0.00		34	34	0	0	yes				blast	accaucugccagcacuguua	acagaauggugagguggaugg	acagaauggugagguggauggucacaccaucugccagcacuguua	gi|307603380|gb|CM000996.1|:94676233..9467
gi|307603379|gb|CM000997.1|_32967	0.4	0.00 ± 0.00		38	38	0	0	yes				blast	ugguguuggacagacuua	aguucguuaacagcaga	ugguguuggacagacuuaaaaaaucucaaguaguucguuaacagcaga	gi|307603379|gb|CM000997.1|:98472375..9847
gi|307603370|gb|CM001006.1|_9376	0.4	0.00 ± 0.00		41	41	0	0	yes				blast	agguaaugagcuagcaua	uucuaggucucaugugccuau	agguaaugagcuagcauaaaauuguguagcacggauaaaauucuaggucucaugugccuau	gi|307603370|gb|CM001006.1|:102003658..102
gi|307603372|gb|CM001004.1|_4738	0.4	0.00 ± 0.00		146	146	0	0	yes				blast	acaaagaggaaaggggag	uccuuauccucuuuaaaa	uccuuauccucuuuaaaagccuuguaguaaaaggcucagaaacagaacaaagaggaaaggggag	gi|307603372|gb|CM001004.1|:46862221..4686
gi|307603382|gb|CM000994.1|_22545	0.4	0.00 ± 0.00		35	35	0	0	yes				blast	cuugcuagucuggggagcucauuu	auaggcucacucagcuuuugcauuau	auaggcucacucagcuuuugcauuaucuagauucauaguugcagcuggagagucuuccuuucuugcuagucuggggagcucauuu	gi|307603382|gb|CM000994.1|:164172458..164
gi|307603369|gb|CM001007.1|_11111	0.4	0.00 ± 0.00		350	350	0	0	yes				blast	acugcucuucugaagguccuga	agggcugaagagauggcac	agggcugaagagauggcacaggugguuaagagcacugacugcucuucugaagguccuga	gi|307603369|gb|CM001007.1|:53807614..5380
gi|307603380|gb|CM000996.1|_29988	0.4	0.00 ± 0.00		109371	109371	0	0	yes				blast	aucucgguggaaccucca	ggguuccugggugu	ggguuccugggugucacacauaucucgguggaaccucca	gi|307603380|gb|CM000996.1|:69726102..6972
gi|307603376|gb|CM001000.1|_41624	0.4	0.00 ± 0.00		241	241	0	0	yes				blast	gaucaagaugaggucuuuaugcu	cacacaaaggcacaccugacuu	cacacaaaggcacaccugacuuuuuacaugcacacugaagaucaagaugaggucuuuaugcu	gi|307603376|gb|CM001000.1|:64661405..6466
gi|307603371|gb|CM001005.1|_7136	0.4	0.00 ± 0.00		37	37	0	0	yes				blast	uucagaauauuggggggug	ccucuuuuuuucugcacu	uucagaauauuggggggugguggaaacccucaccucuuuuuuucugcacu	gi|307603371|gb|CM001005.1|:109844067..109
gi|307603363|gb|CM001013.1|_48668	0.4	0.00 ± 0.00		446	446	0	0	yes				blast	ugaagagacaugagaggu	uuuucauuagcuucucaca	ugaagagacaugagaggucaccuaaagcagcuguuuuucauuagcuucucaca	gi|307603363|gb|CM001013.1|:114184577..114
gi|307603370|gb|CM001006.1|_10481	0.3	0.00 ± 0.00		17185	17185	0	0	yes				blast	accccacuccugguacca	guauuagucaggguuc	accccacuccugguaccaacuucuguauuagucaggguuc	gi|307603370|gb|CM001006.1|:98486693..9848
gi|307603372|gb|CM001004.1|_2750	0.3	0.00 ± 0.00		39	39	0	0	yes				blast	cuaauuuauucuucucuggguua	gccucugagacacuuguuagga	cuaauuuauucuucucuggguuaccguaauagccucugagacacuuguuagga	gi|307603372|gb|CM001004.1|:34231920..3423
gi|307603368|gb|CM001008.1|_13878	0.3	0.00 ± 0.00		30	30	0	0	yes				blast	uuaauuucugugacugcauuuuau	aagaugcaagcacagcaguaauc	aagaugcaagcacagcaguaaucuaagggagcauugauuaauuucugugacugcauuuuau	gi|307603368|gb|CM001008.1|:76975268..7697
gi|307603376|gb|CM001000.1|_39650	0.3	0.00 ± 0.00		39	39	0	0	yes				blast	augaacgaacagccaggg	guggcaggcauguucagug	augaacgaacagccagggagacuguggcaggcauguucagug	gi|307603376|gb|CM001000.1|:25793435..2579
gi|307603374|gb|CM001002.1|_45578	0.3	0.00 ± 0.00		33	33	0	0	yes				blast	cggacaauggagagcaaga	uuaccacuccuuugucccuu	cggacaauggagagcaagauggcugcugccuugcacuccagcgcugcagaugccaccagcaguucaaauuaccacuccuuugucccuu	gi|307603374|gb|CM001002.1|:32066723..3206
gi|307603380|gb|CM000996.1|_29719	0.3	0.00 ± 0.00		287	287	0	0	yes				blast	gggccuguagcucagugg	auuggcuggcuaua	gggccuguagcucaguggcagagccauuggcuggcuaua	gi|307603380|gb|CM000996.1|:30331830..3033
gi|307603368|gb|CM001008.1|_13059	0.3	0.00 ± 0.00		39	39	0	0	yes				blast	gacacucaucagcaagaac	ucuucuccgagguguucu	ucuucuccgagguguucuccauugugaucccuuucucuaugucgggacacucaucagcaagaac	gi|307603368|gb|CM001008.1|:82178340..8217
gi|307603365|gb|CM001011.1|_19124	0.2	0.00 ± 0.00		35	35	0	0	yes				blast	aagcagagcuuagcagug	uggcucaagcucauuc	uggcucaagcucauucuguuauuuauuuaaagucaauagaaagcagagcuuagcagug	gi|307603365|gb|CM001011.1|:15174743..1517
gi|307603378|gb|CM000998.1|_35978	0.2	0.00 ± 0.00		240	240	0	0	yes				blast	uuauaccuuguuuguacccgcca	auggguacaaauuaaggaauugca	auggguacaaauuaaggaauugcagcaaaaagaaauauuugccuacuaacucugacauuauaccuuguuuguacccgcca	gi|307603378|gb|CM000998.1|:100391224..100
gi|307603371|gb|CM001005.1|_6546	0.2	0.00 ± 0.00		29	29	0	0	yes				blast	uuuguggagcuaaucagga	gagaacuaacuccacaaagu	uuuguggagcuaaucaggagcagagaacuaacuccacaaagu	gi|307603371|gb|CM001005.1|:53634181..5363

gi|307603366|gb|CM001010.1|_16433	0.2	0.00 ± 0.00		33	33	0	0	yes				blast	aggcucggagacagacug	uucuucugacccguu	uucuucugacccguuuuuuccgcggaaaagcaacccuccuucccugcuuuuacuaggcucggagacagacug	gi|307603366|gb|CM001010.1|:29492957..2949
gi|307603380|gb|CM000996.1|_28486	0.1	0.00 ± 0.00		29	29	0	0	yes				blast	ggagaaugagaagccauggg	caugugaaauuacccuccca	ggagaaugagaagccaugggaugacagguaacauggaacuuuaaguucacccaugugaaauuacccuccca	gi|307603380|gb|CM000996.1|:36895448..3689
gi|307603367|gb|CM001009.1|_14710	0.1	0.00 ± 0.00		151	151	0	0	yes				blast	guaccaaaagcuagauuug	auucaggcuauuugguagaa	guaccaaaagcuagauuuguggggaagguuuauuaaauaaccuuaaccaauucaggcuauuugguagaa	gi|307603367|gb|CM001009.1|:23111689..2311
gi|307603381|gb|CM000995.1|_27243	0.1	0.00 ± 0.00		628	628	0	0	yes				blast	gaguucgaggccagccuggg	caggcuaaccugagcuaga	gaguucgaggccagccugggcuauauagggaguuccaggcuaaccugagcuaga	gi|307603381|gb|CM000995.1|:84707183..8470
gi|307603379|gb|CM000997.1|_31058	0.1	0.00 ± 0.00		35	35	0	0	yes				blast	acagugggugauguggcua	ggcgcuaaggaguuaucuggaa	acagugggugauguggcuaagaaagggcgcuaaggaguuaucuggaa	gi|307603379|gb|CM000997.1|:40824005..4082
gi|307603364|gb|CM001012.1|_20037	0.1	0.00 ± 0.00		28	28	0	0	yes				blast	agaccacugcuugccugaa	uagaaaggcaguuuu	uagaaaggcaguuuuagaagcuauuugugauccacuaaggaaauguuuuaucagcuagagaccacugcuugccugaa	gi|307603364|gb|CM001012.1|:16460367..1646
gi|307603363|gb|CM001013.1|_49287	0.1	0.00 ± 0.00		61	61	0	0	yes				blast	augagaccugacugagcaag	uacagcuuauuuacaaucauau	augagaccugacugagcaagcuaguacagguccugccaugguggggagccuuugcuaguuuacagcuuauuuacaaucauau	gi|307603363|gb|CM001013.1|:70473194..7047
gi|307603368|gb|CM001008.1|_13508	0	0.00 ± 0.00		40	40	0	0	yes				blast	aagaaagaagggaaauca	uaaauucuacuuucuuau	uaaauucuacuuucuuauaaaagaaagaagggaaauca	gi|307603368|gb|CM001008.1|:26903337..2690
gi|307603378|gb|CM000998.1|_35242	0	0.00 ± 0.00		32	32	0	0	yes				blast	ugagcuguggaaggcaag	ugcuuuccuagcugucuagcc	ugagcuguggaaggcaagccuugcuuuccuagcugucuagcc	gi|307603378|gb|CM000998.1|:141888593..141
gi|307603373|gb|CM001003.1|_125	0	0.00 ± 0.00		31	31	0	0	yes				blast	ugacggcagauuagauuua	aguuuaacuguauugaac	aguuuaacuguauugaacauucuucucauuugcuuuugacucuauguauaguuaugacggcagauuagauuua	gi|307603373|gb|CM001003.1|:25243176..2524
gi|307603381|gb|CM000995.1|_27069	0	0.00 ± 0.00		157	157	0	0	yes				blast	acauuugccugcaucugaa	cgaggagcaaguaaaggcug	acauuugccugcaucugaaccagacuuugcuacaaaacugcaaagccuuucgaggagcaaguaaaggcug	gi|307603381|gb|CM000995.1|:62577349..6257
gi|307603368|gb|CM001008.1|_13073	0	0.00 ± 0.00		29	29	0	0	yes				blast	uggucggacaugguacacagu	acggugccaaauccuuauaaccucc	acggugccaaauccuuauaaccuccucagcaccuugccaccuggucggacaugguacacagu	gi|307603368|gb|CM001008.1|:83027501..8302
gi|307603371|gb|CM001005.1|_8281	0	0.00 ± 0.00		29	29	0	0	yes				blast	uaacuaguuugaaucauacu	uaugaguuagacgcgcgauc	uaacuaguuugaaucauacugugauuuacccagagaccaaguccgaaaaaucaaaaguaugaguuagacgcgcgauc	gi|307603371|gb|CM001005.1|:105927533..105
gi|307603370|gb|CM001006.1|_8814	0	0.00 ± 0.00		30	30	0	0	yes				blast	gugaacuuggcugccucu	aggaaagcccuugacacag	gugaacuuggcugccucugcuaugaugcagaggaaagcccuugacacag	gi|307603370|gb|CM001006.1|:37986122..3798
gi|307603373|gb|CM001003.1|_888	0	0.00 ± 0.00		36	36	0	0	yes				blast	uugaagaucugugccauacg	cuugagcaagauuucagag	uugaagaucugugccauacgaucucagaccacuacuuagauaacuugagcaagauuucagag	gi|307603373|gb|CM001003.1|:89539792..8953
gi|307603378|gb|CM000998.1|_34914	0	0.00 ± 0.00		41	41	0	0	yes				blast	gccuuggagccuucugug	caggcagguuugcuuggcug	gccuuggagccuucuguguaguuugcaggcagguuugcuuggcug	gi|307603378|gb|CM000998.1|:122210759..122
gi|307603377|gb|CM000999.1|_37467	0	0.00 ± 0.00		31	31	0	0	yes				blast	gagaucagaggauaauuu	uuaaucuauugcuuuugg	uuaaucuauugcuuuuggguguuuguguguguauaugugccuucacauaugcacacuuuuauuuguggagaucagaggauaauuu	gi|307603377|gb|CM000999.1|:85547802..8554

mature miRBase miRNAs detected by miRDeep2

tag id	miRDeep2 score	estimated probability that the miRNA is a true positive	rfam alert	predicted mature seq. in accordance with miRBase mature seq.	total read count	mature read count	loop read count	star read count	significant randfold p-value	mature miRBase miRNA	example miRBase miRNA with the same seed	UCSC browser	NCBI blastn	consensus mature sequence	consensus star sequence	consensus precursor sequence	precurso
gi|307603380|gb|CM000996.1|_28788	2.0e+7	0.61 ± 0.06		TRUE	39579646	33824661	2871	5752114	yes	mmu-miR-9-5p			blast	ucuuugguuaucuagcuguauga	auaaagcuagauaaccgaaagu	ucuuugguuaucuagcuguaugagugguguggagucuucauaaagcuagauaaccgaaagu	gi|307603380|gb|CM00099
gi|307603370|gb|CM001006.1|_9247	2.0e+7	0.61 ± 0.06		TRUE	39576775	33824661	0	5752114	yes	mmu-miR-9-5p			blast	ucuuugguuaucuagcuguauga	auaaagcuagauaaccgaaagu	ucuuugguuaucuagcuguaugaguguauuggucuucauaaagcuagauaaccgaaagu	gi|307603370|gb|CM00100
gi|307603376|gb|CM001000.1|_40156	2.0e+7	0.61 ± 0.06		TRUE	39432323	33817751	0	5614572	yes	mmu-miR-9-5p			blast	ucuuugguuaucuagcuguauga	auaaagcuagauaaccgaaagu	ucuuugguuaucuagcuguaugagugccacagagccgucauaaagcuagauaaccgaaagu	gi|307603376|gb|CM00100
gi|307603374|gb|CM001002.1|_45668	9.4e+6	0.61 ± 0.06		TRUE	18449090	18302095	16	146979	yes	mmu-miR-125b-5p			blast	ucccugagacccuaacuuguga	acggguuaggcucuugggagcu	ucccugagacccuaacuugugauguuuaccguuuaaauccacggguuaggcucuugggagcu	gi|307603374|gb|CM00100
gi|307603367|gb|CM001009.1|_15090	9.2e+6	0.61 ± 0.06		TRUE	18229776	18170660	0	59116	yes	mmu-miR-125b-5p			blast	ucccugagacccuaacuuguga	acaagucagguucuugggacc	ucccugagacccuaacuugugagguuuuuaguaacaucacaagucagguucuugggacc	gi|307603367|gb|CM00100
gi|307603369|gb|CM001007.1|_11513	4.0e+6	0.61 ± 0.06		STAR	8009983	8007628	32	2323	yes	mmu-miR-92a-1-5p			blast	uauugcacuugucccggccugu	agguugggauuugucgcaaugcu	agguugggauuugucgcaaugcugugguuucucuguaugguauugcacuugucccggccugu	gi|307603369|gb|CM00100
gi|307603381|gb|CM000995.1|_25100	4.0e+6	0.61 ± 0.06		TRUE	7925912	7788518	76	137318	yes	mmu-miR-181a-5p			blast	aacauucaacgcugucggugagu	accaccgaccguugacuguacc	aacauucaacgcugucggugaguuugggauucaaaaacaaaaaaaccaccgaccguugacuguacc	gi|307603381|gb|CM00099
gi|307603382|gb|CM000994.1|_22378	3.9e+6	0.61 ± 0.06		TRUE	7829283	7788478	0	40805	yes	mmu-miR-181a-5p			blast	aacauucaacgcugucggugagu	accaucgaccguugauuguacc	aacauucaacgcugucggugaguuugggauuucaaauaaaaaccaucgaccguugauuguacc	gi|307603382|gb|CM00099
gi|307603367|gb|CM001009.1|_15086	2.9e+6	0.61 ± 0.06		TRUE	5823305	5823098	0	207	yes	mmu-miR-99a-5p			blast	aacccguagauccgaucuugug	caagcucguuucuaugggucugu	aacccguagauccgaucuuguggugaaguggaccgcgcaagcucguuucuaugggucugu	gi|307603367|gb|CM00100
gi|307603367|gb|CM001009.1|_15088	2.9e+6	0.61 ± 0.06		TRUE	5782735	5775081	3349	4305	yes	mmu-let-7c-5p			blast	ugagguaguagguuguaugguu	cuguacaaccuucuagcuuucc	ugagguaguagguuguaugguuuagaguuacacccugggaguuaacuguacaaccuucuagcuuucc	gi|307603367|gb|CM00100
gi|307603373|gb|CM001003.1|_1148	2.4e+6	0.61 ± 0.06		TRUE	4810238	4806477	0	3761	yes	mmu-miR-26a-5p			blast	uucaaguaauccaggauaggcu	ccuguucuugauuacuuguuuc	uucaaguaauccaggauaggcugugucgucccaugaggccuguucuugauuacuuguuuc	gi|307603373|gb|CM00100
gi|307603374|gb|CM001002.1|_46522	2.4e+6	0.61 ± 0.06		TRUE	4807405	4807083	0	322	yes	mmu-miR-26a-5p			blast	uucaaguaauccaggauaggcu	ccuauucuugguuacuugcacg	uucaaguaauccaggauaggcugugcaggucccaggggccuauucuugguuacuugcacg	gi|307603374|gb|CM00100
gi|307603370|gb|CM001006.1|_10035	2.4e+6	0.61 ± 0.06		TRUE	4743776	4735505	190	8081	yes	mmu-let-7a-5p			blast	ugagguaguagguuguauaguu	cuauacaaucuacugucuuucc	ugagguaguagguuguauaguuuuagggucacacccaccacugggagauaacuauacaaucuacugucuuucc	gi|307603370|gb|CM00100
gi|307603374|gb|CM001002.1|_45664	2.4e+6	0.61 ± 0.06		TRUE	4728081	4727124	464	493	yes	mmu-let-7a-5p			blast	ugagguaguagguuguauaguu	cuguacagccuccuagcuuucc	ugagguaguagguuguauaguuuagaguuacaucaagggagauaacuguacagccuccuagcuuucc	gi|307603374|gb|CM00100
gi|307603373|gb|CM001003.1|_2275	2.3e+6	0.61 ± 0.06		TRUE	4591161	4586401	0	4760	yes	mmu-let-7i-5p			blast	ugagguaguaguuugugcuguu	cugcgcaagcuacugccuugcu	ugagguaguaguuugugcuguuggucggguugugacauugcccgcuguggagauaacugcgcaagcuacugccuugcu	gi|307603373|gb|CM00100
gi|307603374|gb|CM001002.1|_46320	2.3e+6	0.61 ± 0.06		TRUE	4545702	4545527	0	175	yes	mmu-let-7g-5p			blast	ugagguaguaguuuguacaguu	cuguacaggccacugccuugcc	ugagguaguaguuuguacaguuugagggucuaugauaccacccgguacaggagauaacuguacaggccacugccuugcc	gi|307603374|gb|CM00100
gi|307603368|gb|CM001008.1|_13771	2.2e+6	0.61 ± 0.06		TRUE	4429813	4429316	0	497	yes	mmu-miR-30d-5p			blast	uguaaacauccccgacuggaagcu	cuuucagucagauguuugcugc	uguaaacauccccgacuggaagcuguaagccacagccaagcuuucagucagauguuugcugc	gi|307603368|gb|CM00100
gi|307603382|gb|CM000994.1|_21458	2.0e+6	0.61 ± 0.06		TRUE	4036719	3892267	0	144452	yes	mmu-miR-30a-5p			blast	uguaaacauccucgacuggaagcu	cuuucagucggauguuugcagc	uguaaacauccucgacuggaagcugugaagccacaaaugggcuuucagucggauguuugcagc	gi|307603382|gb|CM00099
gi|307603363|gb|CM001013.1|_48838	1.7e+6	0.61 ± 0.06		TRUE	3392433	3392061	86	286	yes	mmu-let-7f-5p			blast	ugagguaguagauuguauaguu	cuauacagucuacugucuuucc	ugagguaguagauuguauaguuuuagggucauaccccaucuuggagauaacuauacagucuacugucuuucc	gi|307603363|gb|CM00101
gi|307603366|gb|CM001010.1|_16196	1.7e+6	0.61 ± 0.06		TRUE	3381688	3371554	0	10134	yes	mmu-miR-125a-5p			blast	ucccugagacccuuuaaccugug	acaggugagguucuugggagcc	ucccugagacccuuuaaccugugaggacguccagggucacaggugagguucuugggagcc	gi|307603366|gb|CM00101
gi|307603370|gb|CM001006.1|_10033	1.6e+6	0.61 ± 0.06		TRUE	3308259	3307571	0	688	yes	mmu-let-7f-5p			blast	ugagguaguagauuguauaguu	cuauacaaucuauugccuuccc	ugagguaguagauuguauaguugugggguagugauuuuacccuguuuaggagauaacuauacaaucuauugccuuccc	gi|307603370|gb|CM00100
gi|307603381|gb|CM000995.1|_25857	1.3e+6	0.61 ± 0.06		STAR	2647646	2647539	0	107	yes	mmu-miR-103-2-5p			blast	agcagcauuguacagggcuauga	agcuucuuuacagugcugccuugu	agcuucuuuacagugcugccuuguagcauucaggucaagcagcauuguacagggcuauga	gi|307603381|gb|CM00099
gi|307603378|gb|CM000998.1|_36592	1.2e+6	0.61 ± 0.06		STAR	2390241	2380029	0	10212	no	mmu-miR-25-5p			blast	cauugcacuugucucggucuga	aggcggagacuugggcaauugcu	aggcggagacuugggcaauugcuggacgcugcccugggcauugcacuugucucggucuga	gi|307603378|gb|CM00099
gi|307603370|gb|CM001006.1|_10185	1.1e+6	0.61 ± 0.06		TRUE	2319377	2312397	0	6980	yes	mmu-miR-7a-5p			blast	uggaagacuagugauuuuguuguu	caacaaaucacagucugccaua	uggaagacuagugauuuuguuguuuuuagauaacuaaaacgacaacaaaucacagucugccaua	gi|307603370|gb|CM00100
gi|307603380|gb|CM000996.1|_28696	1.1e+6	0.61 ± 0.06		TRUE	2201113	2200337	0	776	yes	mmu-miR-16-5p			blast	uagcagcacguaaauauuggcg	accaauauuauugugcugcuuu	uagcagcacguaaauauuggcguagugaaauaaauauuaaacaccaauauuauugugcugcuuu	gi|307603380|gb|CM00099
gi|307603369|gb|CM001007.1|_12141	1.1e+6	0.61 ± 0.06		TRUE	2199252	2198035	66	1151	yes	mmu-miR-16-5p			blast	uagcagcacguaaauauuggcg	ccaguauugacugugcugcugaa	uagcagcacguaaauauuggcguaagauucugaaauuaccuccaguauugacugugcugcugaa	gi|307603369|gb|CM00100
gi|307603382|gb|CM000994.1|_22380	1.0e+6	0.61 ± 0.06		TRUE	2132650	2127050	0	5600	yes	mmu-miR-181b-5p			blast	aacauucauugcugucgguggu	cucacugaacaaugaaugcaa	aacauucauugcugucgguggguugaacuguguagaaaagcucacugaacaaugaaugcaa	gi|307603382|gb|CM00099
gi|307603374|gb|CM001002.1|_45662	9.3e+5	0.61 ± 0.06		TRUE	1826509	1826027	0	482	yes	mmu-miR-100-5p			blast	aacccguagauccgaacuugug	acaagcuugugucuauagguau	aacccguagauccgaacuugugcugauucugcacacaagcuugugucuauagguau	gi|307603374|gb|CM00100
gi|307603382|gb|CM000994.1|_22541	9.2e+5	0.61 ± 0.06		STAR	1810785	1601002	0	209783	yes	mmu-miR-199a-5p			blast	acaguagucugcacauugguu	cccaguguucagacuaccuguuc	cccaguguucagacuaccuguucaggacaaugccguuguacaguagucugcacauugguu	gi|307603382|gb|CM00099
gi|307603374|gb|CM001002.1|_46805	9.1e+5	0.61 ± 0.06		STAR	1803046	1593217	0	209829	no	mmu-miR-199a-5p			blast	acaguagucugcacauugguu	cccaguguucagacuaccuguuc	cccaguguucagacuaccuguucaggaggcuggacauguacaguagucugcacauugguu	gi|307603374|gb|CM00100
gi|307603369|gb|CM001007.1|_11275	8.5e+5	0.61 ± 0.06		STAR	1679665	1677631	0	2034	yes	mmu-miR-124-5p			blast	uaaggcacgcggugaaugc	uguucacagcggaccuugauuu	uguucacagcggaccuugauuuaaauguccauacaauuaaggcacgcggugaaugc	gi|307603369|gb|CM00100
gi|307603380|gb|CM000996.1|_28345	8.5e+5	0.61 ± 0.06		STAR	1679665	1677631	0	2034	yes	mmu-miR-124-5p			blast	uaaggcacgcggugaaugc	uguucacagcggaccuugauuu	uguucacagcggaccuugauuuaaugucauacaauuaaggcacgcggugaaugc	gi|307603380|gb|CM00099
gi|307603381|gb|CM000995.1|_26449	8.5e+5	0.61 ± 0.06		STAR	1679665	1677631	0	2034	no	mmu-miR-124-5p			blast	uaaggcacgcggugaaugc	uguucacagcggaccuugauuu	uguucacagcggaccuugauuuaaugucuauacaauuaaggcacgcggugaaugc	gi|307603381|gb|CM00099
gi|307603381|gb|CM000995.1|_25003	8.1e+5	0.61 ± 0.06		STAR	1599632	1593217	7	6408	yes	mmu-miR-199b-5p			blast	acaguagucugcacauugguu	cccaguguuuagacuaccuguuc	cccaguguuuagacuaccuguucaggacucccaaauguacaguagucugcacauugguu	gi|307603381|gb|CM00099
gi|307603382|gb|CM000994.1|_21460	8.0e+5	0.61 ± 0.06		TRUE	1577023	1551171	0	25852	yes	mmu-miR-30c-5p			blast	uguaaacauccuacacucucagcu	cugggagaaggcuguuuacucu	uguaaacauccuacacucucagcugugaaaaguaagaaagcugggagaaggcuguuuacucu	gi|307603382|gb|CM00099
gi|307603380|gb|CM000996.1|_30146	7.5e+5	0.61 ± 0.06		STAR	1481882	1476620	18	5244	yes	mmu-miR-92b-5p			blast	uauugcacucgucccggccucc	agggacgggacguggugcagugu	agggacgggacguggugcaguguuguuucuuuccccgccaauauugcacucgucccggccucc	gi|307603380|gb|CM00099
gi|307603382|gb|CM000994.1|_21886	7.0e+5	0.61 ± 0.06		TRUE	1384585	1382589	0	1996	yes	mmu-miR-26b-5p			blast	uucaaguaauucaggauagguu	ccuguucuccauuacuuggcuc	uucaaguaauucaggauagguuguggugcugaccagccuguucuccauuacuuggcuc	gi|307603382|gb|CM00099
gi|307603382|gb|CM000994.1|_22239	6.8e+5	0.61 ± 0.06		STAR	1350465	1347008	0	3457	yes	mmu-miR-128-1-5p			blast	ucacagugaaccggucucuuu	cggggccguagcacugucugaga	cggggccguagcacugucugagagguuuacauuucucacagugaaccggucucuuu	gi|307603382|gb|CM00099
gi|307603378|gb|CM000998.1|_36593	5.7e+5	0.61 ± 0.06		TRUE	1121623	1101771	0	19852	yes	mmu-miR-93-5p			blast	caaagugcuguucgugcagguag	acugcugagcuagcacuucccga	caaagugcuguucgugcagguaguguaauuaccugaccuacugcugagcuagcacuucccga	gi|307603378|gb|CM00099
gi|307603379|gb|CM000997.1|_33197	5.6e+5	0.61 ± 0.06		TRUE	1105314	981185	174	123955	yes	mmu-miR-30e-5p			blast	uguaaacauccuugacuggaagcu	cuuucagucggauguuuacagc	uguaaacauccuugacuggaagcuguaagguguugagaggagcuuucagucggauguuuacagc	gi|307603379|gb|CM00099
gi|307603374|gb|CM001002.1|_47944	5.4e+5	0.61 ± 0.06		STAR	1071962	1067572	0	4390	yes	mmu-miR-128-2-5p			blast	ucacagugaaccggucucuuu	gggggccgaugcacuguaagaga	gggggccgaugcacuguaagagagugaguagcaggucucacagugaaccggucucuuu	gi|307603374|gb|CM00100
gi|307603377|gb|CM000999.1|_36976	5.0e+5	0.61 ± 0.06		TRUE	999478	756854	0	242624	yes	mmu-miR-335-5p			blast	ucaagagcaauaacgaaaaaug	uuuuucauuauugcuccugacc	ucaagagcaauaacgaaaaauguuuguuuuucguaaaccguuuuucauuauugcuccugacc	gi|307603377|gb|CM00099
gi|307603363|gb|CM001013.1|_48767	4.9e+5	0.61 ± 0.06		STAR	967292	967245	0	47	yes	mmu-miR-652-5p			blast	aauggcgccacuaggguugugc	caacccuaggagggggugccauuc	caacccuaggagggggugccauucacauagaguaauaugaauggcgccacuaggguugugc	gi|307603363|gb|CM00101
gi|307603371|gb|CM001005.1|_7287	4.8e+5	0.61 ± 0.06		STAR	960634	959566	0	1068	yes	mmu-miR-487b-5p			blast	aaucguacagggucauccacuu	gugguuaucccuguccucuucg	gugguuaucccuguccucuucgcuucacucaugccgaaucguacagggucauccacuu	gi|307603371|gb|CM00100
gi|307603368|gb|CM001008.1|_13799	4.7e+5	0.61 ± 0.06		TRUE	940937	538071	0	402866	yes	mmu-miR-151-5p			blast	ucgaggagcucacagucuagu	cuagacugaggcuccuugagg	ucgaggagcucacagucuaguauguccuccccuacuagacugaggcuccuugagg	gi|307603368|gb|CM00100
gi|307603370|gb|CM001006.1|_10031	4.4e+5	0.61 ± 0.06		TRUE	864361	721130	0	143231	yes	mmu-let-7d-5p			blast	agagguaguagguugcauaguu	cuauacgaccugcugccuuucu	agagguaguagguugcauaguuuuagggcagagauuuugcccacaaggaguuaacuauacgaccugcugccuuucu	gi|307603370|gb|CM00100
gi|307603381|gb|CM000995.1|_24819	4.3e+5	0.61 ± 0.06		STAR	856029	822569	0	33460	yes	mmu-miR-126a-5p			blast	ucguaccgugaguaauaaugcg	cauuauuacuuuugguacgcg	cauuauuacuuuugguacgcgcugugacacuucaaacucguaccgugaguaauaaugcg	gi|307603381|gb|CM00099
gi|307603366|gb|CM001010.1|_16194	4.2e+5	0.61 ± 0.06		TRUE	826648	817295	19	9334	yes	mmu-let-7e-5p			blast	ugagguaggagguuguauaguu	cuauacggccuccuagcuuucc	ugagguaggagguuguauaguugaggaagacacccgaggagaucacuauacggccuccuagcuuucc	gi|307603366|gb|CM00101
gi|307603365|gb|CM001011.1|_19544	4.0e+5	0.61 ± 0.06		STAR	800084	798794	0	1290	yes	mmu-miR-143-5p			blast	ugagaugaagcacuguagcuc	ggugcagugcugcaucucuggu	ggugcagugcugcaucucugguggcaguugggagucugagaugaagcacuguagcuc	gi|307603365|gb|CM00101
gi|307603371|gb|CM001005.1|_7173	4.0e+5	0.61 ± 0.06		STAR	788639	768147	0	20492	no	mmu-miR-433-5p			blast	aucaugaugggcuccucggugu	uacggugagccugucauuauuc	uacggugagccugucauuauucagagaggcuagauccucuguguugagaaggaucaugaugggcuccucggugu	gi|307603371|gb|CM00100
gi|307603371|gb|CM001005.1|_7246	4.0e+5	0.61 ± 0.06		TRUE	785726	671675	0	114051	no	mmu-miR-411-5p			blast	uaguagaccguauagcguacg	uauguaacacgguccacuaac	uaguagaccguauagcguacgcuuuaucugugacguauguaacacgguccacuaac	gi|307603371|gb|CM00100
gi|307603375|gb|CM001001.1|_43425	3.9e+5	0.61 ± 0.06		STAR	778621	767992	0	10629	yes	mmu-miR-24-2-5p			blast	uggcucaguucagcaggaacag	gugccuacugagcugaaacagu	gugccuacugagcugaaacaguugauuccagugcacuggcucaguucagcaggaacag	gi|307603375|gb|CM00100
gi|307603371|gb|CM001005.1|_7310	3.9e+5	0.61 ± 0.06		TRUE	772869	768160	0	4709	yes	mmu-miR-541-5p			blast	aagggauucugauguuggucaca	uggcgaacacagaauccauacu	aagggauucugauguuggucacacuccaagaguuuuaaaaugaguggcgaacacagaauccauacu	gi|307603371|gb|CM00100
gi|307603370|gb|CM001006.1|_9095	3.9e+5	0.61 ± 0.06		STAR	769276	767992	0	1284	no	mmu-miR-24-1-5p			blast	uggcucaguucagcaggaacag	ggugccuacugagcugauauca	ggugccuacugagcugauaucaguucucauuucacacacuggcucaguucagcaggaacag	gi|307603370|gb|CM00100
gi|307603380|gb|CM000996.1|_28694	3.9e+5	0.61 ± 0.06		TRUE	768000	756967	0	11033	yes	mmu-miR-15b-5p			blast	uagcagcacaucaugguuuaca	cgaaucauuauuugcugcucuag	uagcagcacaucaugguuuacauacuacagucaagaugcgaaucauuauuugcugcucuag	gi|307603380|gb|CM00099
gi|307603370|gb|CM001006.1|_9093	3.7e+5	0.61 ± 0.06		STAR	733609	729450	14	4145	yes	mmu-miR-27b-5p			blast	uucacaguggcuaaguucugc	agagcuuagcugauuggugaaca	agagcuuagcugauuggugaacagugauugguuuccguuuguucacaguggcuaaguucugc	gi|307603370|gb|CM00100
gi|307603375|gb|CM001001.1|_44655	3.6e+5	0.61 ± 0.06		TRUE	714114	710646	312	3156	yes	mmu-miR-181d-5p			blast	aacauucauuguugucggugggu	ccaccgggggaugaauguca	aacauucauuguugucgguggguuugugaggaggcagccagacccaccgggggaugaauguca	gi|307603375|gb|CM00100
gi|307603372|gb|CM001004.1|_3806	3.6e+5	0.61 ± 0.06		TRUE	713374	704948	30	8396	yes	mmu-miR-10a-5p			blast	uacccuguagauccgaauuugu	caaauucguaucuaggggaau	uacccuguagauccgaauuuguguaaggaauuuugugucacaaauucguaucuaggggaau	gi|307603372|gb|CM00100
gi|307603375|gb|CM001001.1|_43689	3.6e+5	0.61 ± 0.06		STAR	710746	685325	48	25373	yes	mmu-miR-140-5p			blast	accacaggguagaaccacggac	cagugguuuuacccuaugguag	cagugguuuuacccuaugguagguuacgucaugcuguucuaccacaggguagaaccacggac	gi|307603375|gb|CM00100
gi|307603371|gb|CM001005.1|_7244	3.4e+5	0.61 ± 0.06		TRUE	685046	637050	0	47996	yes	mmu-miR-379-5p			blast	ugguagacuauggaacguagg	uauguaacaugguccacuaac	ugguagacuauggaacguaggcguuauguuuuugaccuauguaacaugguccacuaac	gi|307603371|gb|CM00100
gi|307603371|gb|CM001005.1|_7311	3.4e+5	0.61 ± 0.06		STAR	681745	581575	0	100170	yes	mmu-miR-409-5p			blast	gaauguugcucggugaaccccu	agguuacccgagcaacuuugcauc	agguuacccgagcaacuuugcaucuggaggacgaauguugcucggugaaccccu	gi|307603371|gb|CM00100
gi|307603382|gb|CM000994.1|_22110	3.2e+5	0.61 ± 0.06		TRUE	642833	641467	245	1121	yes	mmu-miR-149-5p			blast	ucuggcuccgugucuucacuccc	gagggagggacgggggcggugc	ucuggcuccgugucuucacucccguguuuguccgaggagggagggagggacgggggcggugc	gi|307603382|gb|CM00099
gi|307603371|gb|CM001005.1|_7296	3.2e+5	0.61 ± 0.06		TRUE	632798	527244	0	105554	yes	mmu-miR-382-5p			blast	gaaguuguucgugguggauucg	aaucauucacggacaacacuuu	gaaguuguucgugguggauucgcuuuacuugugacgaaucauucacggacaacacuuu	gi|307603371|gb|CM00100
gi|307603371|gb|CM001005.1|_7189	3.1e+5	0.61 ± 0.06		STAR	620533	605908	0	14625	yes	mmu-miR-370-5p			blast	gccugcugggguggaaccuggu	caggucacgucucugcaguuacac	caggucacgucucugcaguuacacagcucaugagugccugcugggguggaaccuggu	gi|307603371|gb|CM00100
gi|307603371|gb|CM001005.1|_7253	3.1e+5	0.61 ± 0.06		STAR	618286	615426	0	2860	no	mmu-miR-323-5p			blast	cacauuacacggucgaccucu	aggugguccguggcgcguucg	aggugguccguggcgcguucgcuucauuuauggcgcacauuacacggucgaccucu	gi|307603371|gb|CM00100
gi|307603374|gb|CM001002.1|_46410	3.1e+5	0.61 ± 0.06		TRUE	610831	607570	0	3261	yes	mmu-miR-191-5p			blast	caacggaaucccaaaagcagcu	gcugcacuuggauuucguuccc	caacggaaucccaaaagcagcuguuguuguccagagcauuccagcugcacuuggauuucguuccc	gi|307603374|gb|CM00100
gi|307603368|gb|CM001008.1|_13409	3.0e+5	0.61 ± 0.06		STAR	589011	587468	0	1543	yes	mmu-miR-148b-5p			blast	ucagugcaucacagaacuuugu	gaaguucuguuauacacucaggc	gaaguucuguuauacacucaggcuguggcucugaaagucagugcaucacagaacuuugu	gi|307603368|gb|CM00100
gi|307603371|gb|CM001005.1|_7137	2.9e+5	0.61 ± 0.06		STAR	569466	553084	534	15848	yes	mmu-miR-342-5p			blast	ucucacacagaaaucgcacccguc	aggggugcuaucugugauugagg	aggggugcuaucugugauugagggacaugguuaauggaauugucucacacagaaaucgcacccguc	gi|307603371|gb|CM00100
gi|307603381|gb|CM000995.1|_25322	2.8e+5	0.61 ± 0.06		TRUE	558183	553061	0	5122	yes	mmu-miR-10b-5p			blast	uacccuguagaaccgaauuugu	cagauucgauucuaggggaau	uacccuguagaaccgaauuugugugguacccacauagucacagauucgauucuaggggaau	gi|307603381|gb|CM00099
gi|307603374|gb|CM001002.1|_46315	2.6e+5	0.61 ± 0.06		TRUE	519646	470821	0	48825	yes	mmu-miR-135a-5p			blast	uauggcuuuuuauuccuauguga	auauagggauuggagccguggc	uauggcuuuuuauuccuaugugauucuauugcucgcucauauagggauuggagccguggc	gi|307603374|gb|CM00100
gi|307603373|gb|CM001003.1|_2037	2.6e+5	0.61 ± 0.06		TRUE	518159	470604	0	47555	yes	mmu-miR-135a-5p			blast	uauggcuuuuuauuccuauguga	uguagggauggaagccaugaaa	uauggcuuuuuauuccuaugugaucguaauaaagucucauguagggauggaagccaugaaa	gi|307603373|gb|CM00100
gi|307603372|gb|CM001004.1|_2846	2.5e+5	0.61 ± 0.06		TRUE	502812	388779	953	113080	yes	mmu-miR-340-5p			blast	uuauaaagcaaugagacugauu	uccgucucaguuacuuuauagcc	uuauaaagcaaugagacugauugucauaugcguuguggauccgucucaguuacuuuauagcc	gi|307603372|gb|CM00100
gi|307603377|gb|CM000999.1|_38185	2.5e+5	0.61 ± 0.06		TRUE	499404	496745	0	2659	yes	mmu-miR-183-5p			blast	uauggcacugguagaauucacu	ugaauuaccgaagggccauaa	uauggcacugguagaauucacugugaacagucucagucagugaauuaccgaagggccauaa	gi|307603377|gb|CM00099
gi|307603372|gb|CM001004.1|_5250	2.4e+5	0.61 ± 0.06		STAR	490342	404528	0	85814	yes	mmu-miR-423-5p			blast	agcucggucugaggccccucagu	ugaggggcagagagcgagacuuu	ugaggggcagagagcgagacuuuucuauuuuccaaaagcucggucugaggccccucagu	gi|307603372|gb|CM00100
gi|307603368|gb|CM001008.1|_13124	2.4e+5	0.61 ± 0.06		TRUE	480719	476005	5	4709	yes	mmu-let-7b-5p			blast	ugagguaguagguugugugguu	cuauacaaccuacugccuuccc	ugagguaguagguugugugguuucagggcagugaugauugccccugaaggauaacuauacaaccuacugccuuccc	gi|307603368|gb|CM00100
gi|307603370|gb|CM001006.1|_9091	2.2e+5	0.61 ± 0.06		STAR	441918	440358	0	1560	no	mmu-miR-23b-5p			blast	aucacauugccagggauuaccacg	ggguuccuggcaugcugauuu	ggguuccuggcaugcugauuugugacuugagauuaaaaucacauugccagggauuaccacg	gi|307603370|gb|CM00100
gi|307603372|gb|CM001004.1|_5403	2.0e+5	0.61 ± 0.06		TRUE	397469	397324	0	145	yes	mmu-miR-21a-5p			blast	uagcuuaucagacugauguugac	caacagcagucgaugggcuguc	uagcuuaucagacugauguugacuguugaaucucauggcaacagcagucgaugggcuguc	gi|307603372|gb|CM00100
gi|307603363|gb|CM001013.1|_48840	1.9e+5	0.61 ± 0.06		TRUE	391560	389790	0	1770	yes	mmu-miR-98-5p			blast	ugagguaguaaguuguauuguu	cuauacaacuuacuacuuuccu	ugagguaguaaguuguauuguuguggggguagggauuuuaggccccaguaagaagauaacuauacaacuuacuacuuuccu	gi|307603363|gb|CM00101
gi|307603367|gb|CM001009.1|_15463	1.9e+5	0.61 ± 0.06		TRUE	388513	321488	0	67025	no	mmu-miR-130b-5p			blast	acucuuucccuguugcacuacu	cagugcaaugaugaaagggcau	acucuuucccuguugcacuacuguggggccucugggaagcagugcaaugaugaaagggcau	gi|307603367|gb|CM00100
gi|307603371|gb|CM001005.1|_7267	1.8e+5	0.61 ± 0.06		STAR	372138	365505	0	6633	yes	mmu-miR-543-5p			blast	aaacauucgcggugcacuucuu	aaguugcccgcguguuuuucg	aaguugcccgcguguuuuucgcuuuaauaugugacgaaacauucgcggugcacuucuu	gi|307603371|gb|CM00100
gi|307603382|gb|CM000994.1|_21447	1.8e+5	0.61 ± 0.06		STAR	363248	363209	0	39	yes	mmu-miR-206-5p			blast	uggaauguaaggaagugugugg	acaugcuucuuuauauccucaua	acaugcuucuuuauauccucauagauaucucagcacuauggaauguaaggaagugugugg	gi|307603382|gb|CM00099
gi|307603371|gb|CM001005.1|_7315	1.6e+5	0.61 ± 0.06		STAR	319227	267655	0	51572	yes	mmu-miR-369-5p			blast	aauaauacaugguugaucuuu	agaucgaccguguuauauucgc	agaucgaccguguuauauucgcuuggcugacuucgaauaauacaugguugaucuuu	gi|307603371|gb|CM00100
gi|307603381|gb|CM000995.1|_28151	1.6e+5	0.61 ± 0.06		TRUE	316805	316383	0	422	yes	mmu-miR-298-5p			blast	ggcagaggagggcuguucuucc	aggaacuagccuucucucagcuua	ggcagaggagggcuguucuucccuugaguuuuaugacugggaggaacuagccuucucucagcuua	gi|307603381|gb|CM00099
gi|307603382|gb|CM000994.1|_24487	1.5e+5	0.61 ± 0.06		TRUE	311030	310623	0	407	yes	mmu-miR-205-5p			blast	uccuucauuccaccggagucugu	gauuucaguggagugaagcuca	uccuucauuccaccggagucugucuuaugccaaccagauuucaguggagugaagcuca	gi|307603382|gb|CM00099
gi|307603363|gb|CM001013.1|_49180	1.4e+5	0.61 ± 0.06		STAR	286540	286539	0	1	yes	mmu-miR-92a-2-5p			blast	uauugcacuugucccggccuga	agguggggauugguggcauuacu	agguggggauugguggcauuacuuguguuagauauaaaguauugcacuugucccggccuga	gi|307603363|gb|CM00101
gi|307603366|gb|CM001010.1|_16896	1.4e+5	0.61 ± 0.06		TRUE	279493	278832	0	661	yes	mmu-miR-7b-5p			blast	uggaagacuugugauuuuguuguu	caacaagucacagccagccuca	uggaagacuugugauuuuguuguucugauaugauaugacaacaagucacagccagccuca	gi|307603366|gb|CM00101
gi|307603363|gb|CM001013.1|_48968	1.4e+5	0.61 ± 0.06		STAR	275070	268505	0	6565	yes	mmu-miR-501-5p			blast	aaugcacccgggcaaggauuugg	aauccuuugucccugggugaaa	aauccuuugucccugggugaaaaugcuauuuguaugcaaugcacccgggcaaggauuugg	gi|307603363|gb|CM00101
gi|307603380|gb|CM000996.1|_29582	1.3e+5	0.61 ± 0.06		TRUE	271430	270885	0	545	yes	mmu-miR-186-5p			blast	caaagaauucuccuuuugggcu	gcccuaaggugaauuuuuuggg	caaagaauucuccuuuugggcuuucucauuuuauuuuaagcccuaaggugaauuuuuuggg	gi|307603380|gb|CM00099
gi|307603379|gb|CM000997.1|_33826	1.3e+5	0.61 ± 0.06		STAR	270403	264408	0	5995	yes	mmu-miR-200b-5p			blast	uaauacugccugguaaugaugac	caucuuacugggcagcauugga	caucuuacugggcagcauuggauagugucugaucucuaauacugccugguaaugaugac	gi|307603379|gb|CM00099
gi|307603378|gb|CM000998.1|_36596	1.3e+5	0.61 ± 0.06		STAR	270308	175544	0	94764	yes	mmu-miR-106b-5p			blast	ccgcacuguggguacuugcug	uaaagugcugacagugcagaua	uaaagugcugacagugcagauaguggucccucucugugcuaccgcacuguggguacuugcug	gi|307603378|gb|CM00099
gi|307603363|gb|CM001013.1|_49206	1.3e+5	0.61 ± 0.06		STAR	268232	255991	26	12215	yes	mmu-miR-322-5p			blast	aaacaugaagcgcugcaaca	cagcagcaauucauguuuugga	cagcagcaauucauguuuuggaguauugccaagguucaaaacaugaagcgcugcaaca	gi|307603363|gb|CM00101
gi|307603375|gb|CM001001.1|_43421	1.3e+5	0.61 ± 0.06		STAR	266974	266635	0	339	no	mmu-miR-23a-5p			blast	aucacauugccagggauuucca	gggguuccuggggaugggauuu	gggguuccuggggaugggauuugaugccagucacaaaucacauugccagggauuucca	gi|307603375|gb|CM00100
gi|307603363|gb|CM001013.1|_49563	1.3e+5	0.61 ± 0.06		TRUE	262981	220760	0	42221	yes	mmu-miR-361-5p			blast	uuaucagaaucuccaggggguac	cccccaggugugauucugauuugu	uuaucagaaucuccaggggguacuuaguauuugaaaagucccccaggugugauucugauuugu	gi|307603363|gb|CM00101
gi|307603375|gb|CM001001.1|_42989	1.3e+5	0.61 ± 0.06		TRUE	262851	261378	0	1473	yes	mmu-miR-486b-5p			blast	uccuguacugagcugccccgagg	cggggcagcucaguacaggaug	uccuguacugagcugccccgaggucccuucacugugcucagcucggggcagcucaguacaggaug	gi|307603375|gb|CM00100
gi|307603375|gb|CM001001.1|_44133	1.3e+5	0.61 ± 0.06		TRUE	261549	261197	0	352	yes	mmu-miR-486b-5p			blast	uccuguacugagcugccccgagc	cggggcagcucaguacagga	uccuguacugagcugccccgagcugagcacagugaaggaccucggggcagcucaguacagga	gi|307603375|gb|CM00100
gi|307603382|gb|CM000994.1|_22278	1.3e+5	0.61 ± 0.06		TRUE	261512	255833	26	5653	yes	mmu-miR-135b-5p			blast	uauggcuuuucauuccuauguga	auguagggcuaaaagccaugggc	uauggcuuuucauuccuaugugauugcugcuccgaacucauguagggcuaaaagccaugggc	gi|307603382|gb|CM00099
gi|307603363|gb|CM001013.1|_49201	1.2e+5	0.61 ± 0.06		TRUE	239545	235567	28	3950	yes	mmu-miR-351-5p			blast	ucccugaggagcccuuugagccu	ggucaagaggcgccugggaac	ucccugaggagcccuuugagccuggagugaaaaaaaaaaacaggucaagaggcgccugggaac	gi|307603363|gb|CM00101
gi|307603371|gb|CM001005.1|_7301	1.2e+5	0.61 ± 0.06		STAR	237039	200348	0	36691	yes	mmu-miR-485-5p			blast	gucauacacggcucuccucucu	agaggcuggccgugaugaauucg	agaggcuggccgugaugaauucgauucaucuaaacgagucauacacggcucuccucucu	gi|307603371|gb|CM00100
gi|307603363|gb|CM001013.1|_48973	1.0e+5	0.61 ± 0.06		TRUE	201831	181147	0	20684	yes	mmu-miR-532-5p			blast	caugccuugaguguaggaccgu	ccucccacacccaaggcuugca	caugccuugaguguaggaccguugacaucuuaauuacccucccacacccaaggcuugca	gi|307603363|gb|CM00101
gi|307603365|gb|CM001011.1|_19536	1.0e+5	0.61 ± 0.06		STAR	196167	194130	0	2037	yes	mmu-miR-378a-5p			blast	acuggacuuggagucagaaggc	cuccugacuccagguccugugu	cuccugacuccagguccuguguguuaccucgaaauagcacuggacuuggagucagaaggc	gi|307603365|gb|CM00101
gi|307603375|gb|CM001001.1|_44657	9.6e+4	0.61 ± 0.06		STAR	189273	153099	0	36174	yes	mmu-miR-181c-5p			blast	aacauucaaccugucggugaguu	accaucgaccguugaguggacc	aacauucaaccugucggugaguuugggcagcucagacaaaccaucgaccguugaguggacc	gi|307603375|gb|CM00100
gi|307603371|gb|CM001005.1|_7317	9.6e+4	0.61 ± 0.06		STAR	188618	186406	0	2212	yes	mmu-miR-410-5p			blast	aauauaacacagauggccugu	agguugucugugaugaguucg	agguugucugugaugaguucgcuuuuauuaaugacgaauauaacacagauggccugu	gi|307603371|gb|CM00100
gi|307603371|gb|CM001005.1|_7164	9.6e+4	0.61 ± 0.06		TRUE	188448	188205	0	243	yes	mmu-miR-337-5p			blast	cggcgucaugcaggaguugauu	ucagcuccuauaugaugccuuu	cggcgucaugcaggaguugauuugcacagccauucagcuccuauaugaugccuuu	gi|307603371|gb|CM00100
gi|307603377|gb|CM000999.1|_38424	8.9e+4	0.61 ± 0.06		STAR	176319	173582	0	2737	yes	mmu-miR-148a-5p			blast	ucagugcacuacagaacuuugu	aaaguucugagacacuccgacu	aaaguucugagacacuccgacucugaguaugauagaagucagugcacuacagaacuuugu	gi|307603377|gb|CM00099

gi|307603381|gb|CM000995.1|_27339	8.8e+4	0.61 ± 0.06		TRUE	173757	166236	0	7521	yes	mmu-miR-129-5p			blast	cuuuuugcggucugggcuugc	aagcccuuaccccaaaaagcau	cuuuuugcggucugggcuugcuguacauaacucaauagccggaagcccuuaccccaaaaagcau	gi|307603381|gb|CM0009
gi|307603377|gb|CM000999.1|_38181	8.7e+4	0.61 ± 0.06		TRUE	171948	170935	277	736	yes	mmu-miR-182-5p			blast	uuuggcaaugguagaacucacacc	gugguucuagacuugccaacua	uuuggcaaugguagaacucacaccgguaagguaaugggacccgguggucuagacuugccaacua	gi|307603377|gb|CM0009
gi|307603377|gb|CM000999.1|_36938	8.6e+4	0.61 ± 0.06		TRUE	170187	166173	17	3997	yes	mmu-miR-129-5p			blast	cuuuuugcggucugggcuugc	aagcccuuaccccaaaaaguau	cuuuuugcggucugggcuugcuguucucucgacaguagucaggaagcccuuaccccaaaaaguau	gi|307603377|gb|CM0009
gi|307603371|gb|CM001005.1|_7271	8.6e+4	0.61 ± 0.06		STAR	168920	168450	0	470	yes	mmu-miR-495-5p			blast	aaacaaacauggugcacuucuu	gaaguugcccauguuauuuuucg	gaaguugcccauguuauuuuucgcuuuuauuugugacgaaacaaacauggugcacuucuu	gi|307603371|gb|CM0010
gi|307603379|gb|CM000997.1|_32986	8.2e+4	0.61 ± 0.06		STAR	162424	162397	0	27	yes	mmu-miR-101a-5p			blast	guacaguacugugauaacugaa	ucaguuaucacagugcugaugc	ucaguuaucacagugcugaugcuguccauucuaaagguacaguacugugauaacugaa	gi|307603379|gb|CM0009
gi|307603371|gb|CM001005.1|_7298	7.7e+4	0.61 ± 0.06		TRUE	151391	150412	12	967	no	mmu-miR-134-5p			blast	ugugacugguugaccagagggg	cugugggccaccuagucacc	ugugacugguugaccagaggggcgugcacucuguucacccugugggccaccuagucacc	gi|307603371|gb|CM0010
gi|307603381|gb|CM000995.1|_28149	7.5e+4	0.61 ± 0.06		TRUE	147536	119148	0	28388	yes	mmu-miR-296-5p			blast	agggcccccccucaauccugu	aggguuggguggaggcucucc	agggcccccccucaauccuguugugcucgcuucagaggguuggguggaggcucucc	gi|307603381|gb|CM0009
gi|307603371|gb|CM001005.1|_7415	7.3e+4	0.61 ± 0.06		STAR	144676	144214	0	462	yes	mmu-miR-203-5p			blast	ugaaauguuuaggaccacuag	agugguucuugacaguuca	agugguucuugacaguucaacaguucuguagcacaauugugaaauguuuaggaccacuag	gi|307603371|gb|CM0010
gi|307603381|gb|CM000995.1|_25611	7.1e+4	0.61 ± 0.06		STAR	140949	106266	0	34683	yes	mmu-miR-674-5p			blast	cacagcucccaucucagaaca	gcacugagaugggaggugguguaa	gcacugagaugggaggugguguaaggcucagguaugcacagcucccaucucagaaca	gi|307603381|gb|CM0009
gi|307603368|gb|CM001008.1|_13769	7.1e+4	0.61 ± 0.06		TRUE	139660	137904	0	1756	yes	mmu-miR-30b-5p			blast	uguaaacauccuacacucagcu	cugggauguggauguuuacguc	uguaaacauccuacacucagcugucauacaugcguuggcugggauguggauguuuacguc	gi|307603368|gb|CM0010
gi|307603363|gb|CM001013.1|_49517	6.9e+4	0.61 ± 0.06		TRUE	135523	88487	0	47036	yes	mmu-miR-384-5p			blast	uguaaacaauuccuaggcaaugu	auuccuagaaauuguucacaau	uguaaacaauuccuaggcaauguguauaauguugguaagucauuccuagaaauuguucacaau	gi|307603363|gb|CM00101
gi|307603363|gb|CM001013.1|_48547	6.8e+4	0.61 ± 0.06		STAR	134854	132113	0	2741	yes	mmu-miR-676-5p			blast	ccguccugagguuguugagcu	acucuacaaccuuaggacuugc	acucuacaaccuuaggacuugcagaauuaaaggaaugccguccugagguuguugagcu	gi|307603363|gb|CM00101
gi|307603372|gb|CM001004.1|_3349	6.2e+4	0.61 ± 0.06		STAR	122060	118008	0	4052	yes	mmu-miR-22-5p			blast	aagcugccaguugaagaacugu	aguucuucaguggcaagcuuua	aguucuucaguggcaagcuuuauguccugacccagcuaaagcugccaguugaagaacugu	gi|307603372|gb|CM0010
gi|307603367|gb|CM001009.1|_14724	6.1e+4	0.61 ± 0.06		TRUE	120320	65006	0	55314	yes	mmu-miR-28a-5p			blast	aaggagcucacagucuauuga	cacuagauugugagcugcugga	aaggagcucacagucuauugaguugccuuucugauucucccacuagauugugagcugcugga	gi|307603367|gb|CM0010
gi|307603374|gb|CM001002.1|_46412	6.1e+4	0.61 ± 0.06		TRUE	120135	116619	0	3516	yes	mmu-miR-425-5p			blast	aaugacacgaucacucccguuga	aucgggaaugucguguccgccc	aaugacacgaucacucccguugaguggggcacccaagaagccaucgggaaugucguguccgccc	gi|307603374|gb|CM0010
gi|307603363|gb|CM001013.1|_48080	6.0e+4	0.61 ± 0.06		TRUE	118558	117639	0	919	yes	mmu-miR-1198-5p			blast	uaugguguuccuggcuggcuugg	aagcuagccucuaacucauggc	uaugguguuccuggcuggcuugguacucauguguagcccaagcuagccucuaacucauggc	gi|307603363|gb|CM00101
gi|307603382|gb|CM000994.1|_22505	6.0e+4	0.61 ± 0.06		STAR	117762	116233	0	1529	yes	mmu-miR-488-5p			blast	uugaaaggcuguuucuugguc	cccagauaauagcacucucaaac	cccagauaauagcacucucaaacaaguuuccauguuguuugaaaggcuguuucuugguc	gi|307603382|gb|CM0009
gi|307603371|gb|CM001005.1|_7277	5.9e+4	0.61 ± 0.06		STAR	117348	106212	0	11136	yes	mmu-miR-376b-5p			blast	aucauagaggaacauccacuu	guggauauuccuucuaugguua	guggauauuccuucuaugguuacgugcuuccuggauaaucauagaggaacauccacuu	gi|307603371|gb|CM0010
gi|307603376|gb|CM001000.1|_40392	5.7e+4	0.61 ± 0.06		TRUE	112991	107970	0	5021	yes	mmu-miR-139-5p			blast	ucuacagugcacgugucuccagu	uggagacgcggcccuguuggagu	ucuacagugcacgugucuccagugugggcucggaggcuggagacgcggcccuguuggagu	gi|307603376|gb|CM0010
gi|307603381|gb|CM000995.1|_26431	5.7e+4	0.61 ± 0.06		STAR	111839	111223	73	543	yes	mmu-miR-133a-5p			blast	uugguccccuucaaccagcugu	agcugguaaaauggaaccaaau	agcugguaaaauggaaccaaaucagcuguuggauggauuugguccccuucaaccagcugu	gi|307603381|gb|CM0009
gi|307603365|gb|CM001011.1|_19090	5.6e+4	0.61 ± 0.06		STAR	111766	111223	0	543	yes	mmu-miR-133a-5p			blast	uugguccccuucaaccagcugu	agcugguaaaauggaaccaaau	agcugguaaaauggaaccaaaucgccucuucaauggauuugguccccuucaaccagcugu	gi|307603365|gb|CM00101
gi|307603363|gb|CM001013.1|_48800	5.6e+4	0.61 ± 0.06		TRUE	110958	107454	0	3504	yes	mmu-miR-1298-5p			blast	uucauucggcuguccagaugua	caucugggcaacugauugaacu	uucauucggcuguccagauguacccaaguacccuguuauuuggcaauacauacaucugggcaacugauugaacu	gi|307603363|gb|CM00101
gi|307603369|gb|CM001007.1|_11510	5.5e+4	0.61 ± 0.06		TRUE	108963	103350	0	5613	yes	mmu-miR-20a-5p			blast	uaaagugcuuauagugcagguag	acugcauuacgagcacuuaaagu	uaaagugcuuauagugcagguagugugugagccaucuacugcauuacgagcacuuaaagu	gi|307603369|gb|CM0010
gi|307603371|gb|CM001005.1|_7156	5.1e+4	0.61 ± 0.06		TRUE	100383	54335	0	46048	yes	mmu-miR-770-5p			blast	agcaccacgugucugggccacgu	cgugggccugacguggagcugg	agcaccacgugucugggccacgugagcaacgccacgugggccugacguggagcugg	gi|307603371|gb|CM0010
gi|307603371|gb|CM001005.1|_7281	4.9e+4	0.61 ± 0.06		STAR	96797	96696	0	101	yes	mmu-miR-300-5p			blast	uaugcaagggcaagcucucuuc	aagagagguuauccuuugugugu	aagagagguuauccuuugugugguuugcuuuacgcgaaaugaauaugcaagggcaagcucucuuc	gi|307603371|gb|CM0010
gi|307603379|gb|CM000997.1|_31428	4.8e+4	0.61 ± 0.06		TRUE	95671	87053	0	8618	yes	mmu-miR-872-5p			blast	aagguuacuuguuaguucagg	ugaacuauugcaguagccuccu	aagguuacuuguuaguucaggaccucauuacuuucugccugaacuauugcaguagccuccu	gi|307603379|gb|CM0009
gi|307603381|gb|CM000995.1|_26770	4.5e+4	0.61 ± 0.06		STAR	89990	87004	1376	1610	yes	mmu-miR-219a-5p			blast	agaauuguggcuggacaucugu	ugauuguccaaacgcaauucuugu	ugauuguccaaacgcaauucuuguacgagucugcggccaaccgagaauuguggcuggacaucugu	gi|307603381|gb|CM0009
gi|307603373|gb|CM001003.1|_2070	4.4e+4	0.61 ± 0.06		STAR	86866	80617	0	6249	yes	mmu-miR-331-5p			blast	gccccugggccuauccuagaa	cuagguauggucccagggauc	cuagguauggucccagggaucccagaucaaaccaggccccugggccuauccuagaa	gi|307603373|gb|CM0010
gi|307603376|gb|CM001000.1|_40306	4.3e+4	0.61 ± 0.06		TRUE	85038	47381	1921	35736	yes	mmu-miR-708-5p			blast	aaggagcuuacaaucuagcuggg	caacuagacugugagcuucuag	aaggagcuuacaaucuagcugggggguagaugacuugcacuugaacacaacuagacugugagcuucuag	gi|307603376|gb|CM0010
gi|307603369|gb|CM001007.1|_11504	4.3e+4	0.61 ± 0.06		TRUE	84785	74513	0	10272	yes	mmu-miR-17-5p			blast	caaagugcuuacagugcagguag	acugcagugagggcacuuguagc	caaagugcuuacagugcagguagugaugugugcaucuacugcagugagggcacuuguagc	gi|307603369|gb|CM0010
gi|307603363|gb|CM001013.1|_49524	4.3e+4	0.61 ± 0.06		STAR	84676	65949	0	18727	yes	mmu-miR-325-5p			blast	uuuauugagcaccuccuauca	ccuaguaggugcucaguaagu	ccuaguaggugcucaguaaguguuugugacauaauucguuuauugagcaccuccuauca	gi|307603363|gb|CM00101
gi|307603382|gb|CM000994.1|_22543	4.3e+4	0.61 ± 0.06		STAR	84654	70566	74	14014	yes	mmu-miR-214-5p			blast	acagcaggcacagacaggcagu	ugccugucuacacuugcugugc	ugccugucuacacuugcugugcagaacauccgcucaccuguacagcaggcacagacaggcagu	gi|307603382|gb|CM0009
gi|307603363|gb|CM001013.1|_49068	4.3e+4	0.61 ± 0.06		STAR	84480	82458	0	2022	yes	mmu-miR-221-5p			blast	agcuacauugucugcuggguuuc	accuggcauacaauguagauuucu	accuggcauacaauguagauuucuguguuuguuaggcaacagcuacauugucugcuggguuuc	gi|307603363|gb|CM00101
gi|307603364|gb|CM001012.1|_20424	4.2e+4	0.61 ± 0.06		TRUE	83986	83386	0	600	yes	mmu-miR-146b-5p			blast	ugagaacugaauuccauaggcugu	gcccuagggacucaguucuggu	ugagaacugaauuccauaggcugugagcucuagcagacgcccuagggacucaguucuggu	gi|307603364|gb|CM00101
gi|307603371|gb|CM001005.1|_7285	4.2e+4	0.61 ± 0.06		STAR	83523	82619	48	856	yes	mmu-miR-381-5p			blast	uauacaagggcaagcucucugu	agcgagguugcccuuuguauauu	agcgagguugcccuuuguauauucgguuuauugacauggaauauacaagggcaagcucucugu	gi|307603371|gb|CM0010
gi|307603372|gb|CM001004.1|_3817	3.8e+4	0.61 ± 0.06		STAR	76408	76086	0	322	yes	mmu-miR-152-5p			blast	ucagugcaugacagaacuugg	agguucugugauacacuccgacu	agguucugugauacacuccgacucgggcucuggagcagucagugcaugacagaacuugg	gi|307603372|gb|CM0010
gi|307603371|gb|CM001005.1|_7259	3.8e+4	0.61 ± 0.06		STAR	75938	75846	0	92	yes	mmu-miR-494-5p			blast	ugaaacauacacgggaaaccucu	agguuguccguguugucuucuc	agguuguccguguugucuucucuuuauuuaugaugaaacauacacgggaaaccucu	gi|307603371|gb|CM0010
gi|307603363|gb|CM001013.1|_49505	3.5e+4	0.61 ± 0.06		TRUE	68815	66146	0	2669	yes	mmu-miR-672-5p			blast	ugagguugguguacugugugugа	acacacagucacuaucuucga	ugagguugguguacugugugugaguauacauauuuaucacacacagucacuaucuucga	gi|307603363|gb|CM00101
gi|307603372|gb|CM001004.1|_3341	3.3e+4	0.61 ± 0.06		STAR	64888	60066	269	4553	yes	mmu-miR-132-5p			blast	uaacagucuacagccauggucg	accguggcuuucgauuguuacu	accguggcuuucgauuguuacuguggggaaccggagguaacagucuacagccauggucg	gi|307603372|gb|CM0010
gi|307603364|gb|CM001012.1|_19844	3.2e+4	0.61 ± 0.06		TRUE	63177	62480	0	697	yes	mmu-miR-194-5p			blast	uguaacagcaacuccauguggaa	ccaguggggcugcuguuaucug	uguaacagcaacuccauguggaagugcccacugguuccaguggggcugcuguuaucug	gi|307603364|gb|CM00101
gi|307603371|gb|CM001005.1|_7273	3.2e+4	0.61 ± 0.06		STAR	62817	62156	0	661	yes	mmu-miR-667-5p			blast	ugacaccugccacccagcccaagg	cggugcugguggagcagugagc	cggugcugguggagcagugagcacgccauacauuauaucugugacaccugccacccagcccaagg	gi|307603371|gb|CM0010
gi|307603369|gb|CM001007.1|_11255	3.1e+4	0.61 ± 0.06		STAR	60904	59877	0	1027	yes	mmu-miR-598-5p			blast	uacgucaucgucgucaucguuau	gcggugaugccgauggugcgagc	gcggugaugccgauggugcgagcugaaaaugggcugcuacgucaucgucgucaucguuau	gi|307603369|gb|CM0010
gi|307603363|gb|CM001013.1|_49279	3.0e+4	0.61 ± 0.06		TRUE	60388	60343	0	45	yes	mmu-miR-224-5p			blast	uaagucacuagugguuccguuu	aauggugcccuagugacuaca	uaagucacuagugguuccguuuagugagguuuugugcauuguuucaaaauggugcccuagugacuaca	gi|307603363|gb|CM00101
gi|307603371|gb|CM001005.1|_7280	3.0e+4	0.61 ± 0.06		TRUE	59098	56681	0	2417	yes	mmu-miR-376a-5p			blast	gguagauucuccuucuaugagu	aucguagaggaaaauccacg	gguagauucuccuucuaugaguacaauauuaaugacuaaucguagaggaaaauccacg	gi|307603371|gb|CM0010
gi|307603376|gb|CM001000.1|_40198	2.8e+4	0.61 ± 0.06		TRUE	56521	56110	8	403	yes	mmu-miR-1839-5p			blast	aagguagauagaacaggucuug	agaccuacuuaucuaccaacagc	aagguagauagaacaggucuuguuugcaaaauaaauucaagaccuacuuaucuaccaacagc	gi|307603376|gb|CM0010
gi|307603371|gb|CM001005.1|_7257	2.6e+4	0.61 ± 0.06		STAR	52565	39763	0	12802	yes	mmu-miR-329-5p			blast	aacacacccagcuaaccuuuuu	agagguuuucuggucucuguuuc	agagguuuucuggucucuguuucuuugaugagaaugaaacacacccagcuaaccuuuuu	gi|307603371|gb|CM0010
gi|307603371|gb|CM001005.1|_7249	2.6e+4	0.61 ± 0.06		STAR	51998	41954	0	10044	yes	mmu-miR-380-5p			blast	uauguaguauggccacaucu	augguugaccauagaacaugcg	augguugaccauagaacaugcgcuacuucugugucguauguaguaugguccacaucu	gi|307603371|gb|CM0010
gi|307603363|gb|CM001013.1|_49497	2.6e+4	0.61 ± 0.06		TRUE	51633	50328	0	1305	yes	mmu-miR-374b-5p			blast	auauaauacaaccugcuaagu	cuuagcagguuguauuaucauu	auauaauacaaccugcuaaguguucuagcacuuagcagguuguauuaucauu	gi|307603363|gb|CM00101
gi|307603367|gb|CM001009.1|_15517	2.5e+4	0.61 ± 0.06		TRUE	49774	47846	0	1928	yes	mmu-miR-185-5p			blast	uggagagaaaggcaguuccuga	aggggcuggcuuuccucuggu	uggagagaaaggcaguuccugauggccccucccaggggcuggcuuuccucuggu	gi|307603367|gb|CM0010
gi|307603381|gb|CM000995.1|_27242	2.5e+4	0.61 ± 0.06		STAR	49209	48479	20	710	yes	mmu-miR-130a-5p			blast	cagugcaauguuaaaagggca	gcucuuuucacauugugcuacu	gcucuuuucacauugugcuacugucuaacguguaccgagcagugcaauguuaaaagggca	gi|307603381|gb|CM0009
gi|307603376|gb|CM001000.1|_39469	2.4e+4	0.61 ± 0.06		STAR	48630	48616	0	14	yes	mmu-miR-3099-5p			blast	uaggcuagagagagguuggg	ccagcuuccuuccagcccuug	ccagcuuccuuccagcccuugauguuuuaguacgcacauaggcuagagagagguuggg	gi|307603376|gb|CM0010
gi|307603368|gb|CM001008.1|_13403	2.2e+4	0.61 ± 0.06		STAR	44951	44861	0	90	yes	mmu-miR-615-5p			blast	uccgagccuggguucucccucu	gggggucccggugcucggaucu	gggggucccggugcucggaucucgagggugcuuauuguucggucсgagccuggguucucccucu	gi|307603368|gb|CM0010
gi|307603371|gb|CM001005.1|_7162	2.2e+4	0.61 ± 0.06		TRUE	44779	42405	0	2374	yes	mmu-miR-493-5p			blast	uuguacaugguaggcuuucauu	ugaagguccuacugugugccagg	uuguacaugguaggcuuucauucauuuuuugcacauucggugaagguccuacugugugccagg	gi|307603371|gb|CM0010
gi|307603363|gb|CM001013.1|_49203	2.2e+4	0.61 ± 0.06		TRUE	44431	30811	15	13605	yes	mmu-miR-503-5p			blast	uagcagcgggaacaguacugcag	gaguauuguuuccacugccugg	uagcagcgggaacaguacugcagugagugguuuggugcccuggaguauuguuuccacugccugg	gi|307603363|gb|CM00101
gi|307603371|gb|CM001005.1|_7266	2.1e+4	0.61 ± 0.06		STAR	42222	24224	0	17998	yes	mmu-miR-666-5p			blast	ggcugcagcgugaucgccugcuca	agcgggcacagcugugagagc	agcgggcacagcugugagagccccccuagguacagcggggcugcagcgugaucgccugcuca	gi|307603371|gb|CM0010
gi|307603372|gb|CM001004.1|_2652	2.0e+4	0.61 ± 0.06		TRUE	40078	39865	0	213	yes	mmu-miR-216b-5p			blast	aaaucucugcaggcaaauguga	acacuuaccuguagagauucuu	aaaucucugcaggcaaaugugaugucacugaagaaaccacacacuuaccuguagagauucuu	gi|307603372|gb|CM0010
gi|307603365|gb|CM001011.1|_19541	2.0e+4	0.61 ± 0.06		TRUE	39909	29785	372	9752	yes	mmu-miR-145a-5p			blast	guccaguuuucccaggaaucccu	auuccuggaaauacuguucuu	guccaguuuucccaggaaucccuuggaugcuaagauggggauuccuggaaauacuguucuu	gi|307603365|gb|CM00101
gi|307603363|gb|CM001013.1|_48803	1.9e+4	0.61 ± 0.06		STAR	38824	37065	0	1759	yes	mmu-miR-448-5p			blast	uugcauauguaggaugucccau	gaacauccugcauagugcugcc	gaacauccugcauagugcugccaggaaaucccuacuucauacuaagggggcuggcugguugcauauguaggaugucccau	gi|307603363|gb|CM00101
gi|307603375|gb|CM001001.1|_44277	1.9e+4	0.61 ± 0.06		TRUE	38627	38439	40	148	yes	mmu-miR-383-5p			blast	agaucagaaggugacuguggcu	ccacagcacugccuggucagaa	agaucagaaggugacuguggcuuuggggugguauuuaaucagccacagcacugccuggucagaa	gi|307603375|gb|CM0010
gi|307603363|gb|CM001013.1|_49192	1.9e+4	0.61 ± 0.06		STAR	38586	37007	0	1579	yes	mmu-miR-450b-5p			blast	auugggaacauuuugcaugcau	uuuugcaguauguuccugaauac	uuuugcaguauguuccugaauacauacauaagcguauugggaacauuuugcaugcau	gi|307603363|gb|CM00101
gi|307603363|gb|CM001013.1|_49239	1.9e+4	0.61 ± 0.06		TRUE	37716	37440	0	276	yes	mmu-miR-504-5p			blast	gacccuggucugcacucuauc	agggagagcagggcagguuucc	gacccuggucugcacucuaucuguaaacuuacugaagggagagcagggcagguuucc	gi|307603363|gb|CM00101
gi|307603371|gb|CM001005.1|_7303	1.8e+4	0.61 ± 0.06		STAR	36859	34782	0	2077	yes	mmu-miR-154-5p			blast	aaucauacacgguugaccuauu	uagguuauccguguugccuucg	uagguuauccguguugccuucgcuuuauuugugacgaaucauacacgguugaccuauu	gi|307603371|gb|CM0010
gi|307603371|gb|CM001005.1|_7299	1.8e+4	0.61 ± 0.06		STAR	36513	35990	0	523	yes	mmu-miR-668-5p			blast	ugucacucggcucggcccacuacc	guaagugugccucgggugagca	guaagugugccucgggugagcaugcacuuaauguagguguaugucacucggcucggcccacuacc	gi|307603371|gb|CM0010
gi|307603380|gb|CM000996.1|_30586	1.8e+4	0.61 ± 0.06		STAR	36419	36400	0	19	yes	mmu-miR-760-5p			blast	cggcucuggggucugugggggagc	ccccucaggccaccagagcccgga	ccccucaggccaccagagcccggauaccucagaaaucggcucugggucuguggggagc	gi|307603380|gb|CM0009
gi|307603372|gb|CM001004.1|_2656	1.7e+4	0.61 ± 0.06		TRUE	35090	34949	0	141	yes	mmu-miR-217-5p			blast	uacugcaucaggaacugacugg	caucaguuccuaaugcauugccu	uacugcaucaggaacugacuggauaagacuuaaccccaucaguuccuaaugcauugccu	gi|307603372|gb|CM0010
gi|307603367|gb|CM001009.1|_15727	1.6e+4	0.61 ± 0.06		TRUE	32742	31885	13	844	yes	mmu-miR-6540-5p			blast	cuaaggcaggcagacuucagugu	ucugaagcuugcuuaccucca	cuaaggcaggcagacuucaguguugcccauuucucaguucugaagcuugcuuaccucca	gi|307603367|gb|CM0010
gi|307603376|gb|CM001000.1|_42782	1.6e+4	0.61 ± 0.06		STAR	32633	31208	0	1425	yes	mmu-miR-483-5p			blast	cacuccuccccucccgucuugu	aagacgggagaagagaagggag	aagacgggagaagagaagggagugguuuuuggggugccucacuccuccccucccgucuugu	gi|307603376|gb|CM0010
gi|307603371|gb|CM001005.1|_7165	1.6e+4	0.61 ± 0.06		STAR	31728	31420	0	308	yes	mmu-miR-540-5p			blast	aggucagaggucgauccuggc	caaggucacccucugacu	caaggucacccucugacucuguggccaagguagacaggucagaggucgauccuggc	gi|307603371|gb|CM0010
gi|307603369|gb|CM001007.1|_11505	1.5e+4	0.61 ± 0.06		STAR	31247	15893	27	15327	no	mmu-miR-18a-5p			blast	acugcccuaagugcuccuucugg	uaaggugcaucuagugcagauagu	uaaggugcaucuagugcagauagugaagaugacuagcaucuacugcccuaagugcuccuucugg	gi|307603369|gb|CM0010
gi|307603372|gb|CM001004.1|_6117	1.5e+4	0.61 ± 0.06		TRUE	30392	29973	0	419	yes	mmu-miR-338-5p			blast	aacaauauccuggugcugagu	uccagcaucagugauuuuguu	aacaauauccuggugcugaguggugcacagugacuccagcaucagugauuuuguu	gi|307603372|gb|CM0010
gi|307603363|gb|CM001013.1|_49178	1.4e+4	0.61 ± 0.06		STAR	29258	23437	0	5821	yes	mmu-miR-363-5p			blast	aauugcacgguauccaucugu	cagguggaacacgaugcaauuu	cagguggaacacgaugcaauuuuugguuggguguaaaggaggaaaauugcacgguauccaucugu	gi|307603363|gb|CM00101
gi|307603371|gb|CM001005.1|_7275	1.4e+4	0.61 ± 0.06		STAR	28732	27918	0	814	yes	mmu-miR-376c-5p			blast	aacauagaggaaauuucacgu	gguggauauuccuucuauguuuau	gguggauauuccuucuauguuuaugcuuuuugugauuaaacauagaggaaauuucacgu	gi|307603371|gb|CM0010
gi|307603369|gb|CM001007.1|_12143	1.4e+4	0.61 ± 0.06		TRUE	28057	27929	0	128	yes	mmu-miR-15a-5p			blast	uagcagcacauaaugguuugug	caggccauacugugcugccuca	uagcagcacauaaugguuugugguugaaaggugcaggccauacugugcugccuca	gi|307603369|gb|CM0010
gi|307603370|gb|CM001006.1|_10164	1.3e+4	0.61 ± 0.06		STAR	26372	26273	0	99	no	mmu-miR-874-5p			blast	cugcccuggcccgagggaccgacu	cggccccacgcaccagggguaaga	cggccccacgcaccaggguaagagagacucacuuccugcccuggcccgagggaccgacu	gi|307603370|gb|CM0010
gi|307603369|gb|CM001007.1|_11511	1.3e+4	0.61 ± 0.06		STAR	26199	26164	0	35	yes	mmu-miR-19b-1-5p			blast	ugugcaaauccaugcaaaacuga	aguuuugcagguuugcauccagc	aguuuugcagguuugcauccagcuguauaauauucugcugugcaaauccaugcaaaacuga	gi|307603369|gb|CM0010
gi|307603379|gb|CM000997.1|_33824	1.3e+4	0.61 ± 0.06		STAR	26061	23318	0	2743	yes	mmu-miR-200a-5p			blast	uaacacugucugguaacgaugu	caucuuaccggacagugcugga	caucuuaccggacagugcuggauuucuuggcuugacucuaacacugucugguaacgaugu	gi|307603379|gb|CM0009
gi|307603366|gb|CM001010.1|_17656	1.2e+4	0.61 ± 0.06		STAR	25279	24497	69	713	yes	mmu-miR-219a-5p			blast	agaguugcgucuggacgucccg	ugauuguccaaacgcaauucucg	ugauuguccaaacgcaauucucgagucucuggcuccggccgagaguugcgucuggacgucccg	gi|307603366|gb|CM00101
gi|307603371|gb|CM001005.1|_7263	1.2e+4	0.61 ± 0.06		STAR	25189	23246	0	1943	yes	mmu-miR-1193-5p			blast	uaggucacccguuuuacuauc	ugguagaccggugacguacacu	ugguagaccggugacguacacuucauuuaugcuguaggucacccguuuuacuauc	gi|307603371|gb|CM0010
gi|307603379|gb|CM000997.1|_33500	1.2e+4	0.61 ± 0.06		STAR	24790	24184	11	595	no	mmu-miR-700-5p			blast	cacgcgggaaccgaguccacc	uaaggcuccuuccugugcuugcag	uaaggcuccuuccugugcuugcaggggagaaauacgaacugcacgcgggaaccgaguccacc	gi|307603379|gb|CM0009
gi|307603363|gb|CM001013.1|_49248	1.2e+4	0.61 ± 0.06		STAR	23628	19224	0	4404	yes	mmu-miR-505-5p			blast	cgucaacacuugcugguuuucucu	gggagccaggaaguauugauguu	gggagccaggaaguauugauguuucugccagguuagcgucaacacuugcugguuuucucu	gi|307603363|gb|CM00101
gi|307603378|gb|CM000998.1|_36629	1.1e+4	0.61 ± 0.06		TRUE	23056	15879	0	7177	yes	mmu-miR-339-5p			blast	ucccuguccuccaggagcucacg	agcgccucggcgacagagccg	ucccuguccuccaggagcucacguaugccugccugugagcgccucggcgacagagccg	gi|307603378|gb|CM0009
gi|307603375|gb|CM001001.1|_43423	1.1e+4	0.61 ± 0.06		STAR	22023	20646	0	1377	no	mmu-miR-27a-5p			blast	uucacaguggcuaaguuccg	agggcuuagcugcuugugagca	agggcuuagcugcuugugagcaaggucсacagcaaagucguguucacaguggcuaaguuccg	gi|307603375|gb|CM0010
gi|307603371|gb|CM001005.1|_7262	1.0e+4	0.61 ± 0.06		TRUE	20197	17388	0	2809	yes	mmu-miR-679-5p			blast	uuggacugugaggugacucuugg	caagguccuccucacaguagc	uuggacugugaggugacucuuggugugugaggcuuuucagcaagguccuccucacaguagc	gi|307603371|gb|CM0010
gi|307603370|gb|CM001006.1|_10237	1.0e+4	0.61 ± 0.06		TRUE	20173	16871	0	3302	yes	mmu-miR-3074-5p			blast	guuccugcugaacugagccagu	gauaucagcucaguaggcaccgg	guuccugcugaacugagccagugugugaaaugagaacugauaucagcucaguaggcaccgg	gi|307603370|gb|CM0010
gi|307603374|gb|CM001002.1|_47129	9.9e+3	0.61 ± 0.06		TRUE	19489	18576	0	913	yes	mmu-miR-34c-5p			blast	aggcaguguaguuagcugauugc	aaucacuaaccacacagccagg	aggcaguguaguuagcugauugcuaauaguaccaaucacuaaccacacagccagg	gi|307603374|gb|CM0010
gi|307603372|gb|CM001004.1|_3598	9.3e+3	0.61 ± 0.06		TRUE	18301	14027	0	4274	yes	mmu-miR-301a-5p			blast	gcucugacuuuauugcacuacu	cagugcaauaguauugucaaagc	gcucugacuuuauugcacuacuguacuuuacagcgagcagugcaauaguauugucaaagc	gi|307603372|gb|CM0010
gi|307603376|gb|CM001000.1|_41820	9.2e+3	0.61 ± 0.06		STAR	18112	18031	0	81	yes	mmu-miR-344d-3-5p			blast	gauauaaccacugccagacuga	agucaggcuagugguuauacucc	agucaggcuagugguuauacuccaggacuuaucugguccuggauauaaccacugccagacuga	gi|307603376|gb|CM0010
gi|307603380|gb|CM000996.1|_29317	8.9e+3	0.61 ± 0.06		STAR	17616	16193	0	1423	yes	mmu-miR-137-5p			blast	uuauugcuuaagaauacgcguag	acgguauucuuggguggauaaua	acgguauucuuggguggauaauacggauuacguuguuauugcuuaagaauacgcguag	gi|307603380|gb|CM0009
gi|307603370|gb|CM001006.1|_9476	8.9e+3	0.61 ± 0.06		TRUE	17581	16750	417	414	yes	mmu-miR-449a-5p			blast	uggcaguguauuguuagcuggu	cagcuaacaaugcgacugcucuc	uggcaguguauuguuagcugguugaguaugugagcggcaccagcuaacaaugcgacugcucuc	gi|307603370|gb|CM0010
gi|307603371|gb|CM001005.1|_7248	8.7e+3	0.61 ± 0.06		TRUE	17211	6924	0	10287	yes	mmu-miR-299a-5p			blast	ugguuuaccgucccacauacau	uauguggggacgguaaaccgcuu	ugguuuaccgucccacauacauuuugaguauguaugugggacgguaaaccgcuu	gi|307603371|gb|CM0010
gi|307603376|gb|CM001000.1|_39912	8.6e+3	0.61 ± 0.06		TRUE	16895	16550	0	345	yes	mmu-miR-150-5p			blast	ucucccaacccuuguaccagug	ccugguacaggccugggggau	ucucccaacccuuguaccagugcugugccucagacccugguacaggccugggggau	gi|307603376|gb|CM0010
gi|307603375|gb|CM001001.1|_44661	8.5e+3	0.61 ± 0.06		TRUE	16835	16806	0	29	yes	mmu-miR-3074-5p			blast	guuccugcugaacugagccagu	uguuucagcucaguaggcacggg	guuccugcugaacugagccagugcacuggaaucaacuguuucagcucaguaggcacggg	gi|307603375|gb|CM0010
gi|307603371|gb|CM001005.1|_8325	8.5e+3	0.61 ± 0.06		TRUE	16836	16573	0	263	no	mmu-miR-1247-5p			blast	acccgucccguucgucccсgga	cgggaacgucgagacugga	acccgucccguucguccccggacguugcucucugccccgggaacgucgagacugga	gi|307603371|gb|CM0010
gi|307603379|gb|CM000997.1|_32925	8.1e+3	0.61 ± 0.06		TRUE	15937	15881	0	56	yes	mmu-miR-31-5p			blast	aggcaagaugcuggcauagcugu	ugcuaugccaacauauugccauc	aggcaagaugcuggcauagcuguugaacugagaaccugcuaugccaacauauugccauc	gi|307603379|gb|CM0009
gi|307603363|gb|CM001013.1|_49194	8.0e+3	0.61 ± 0.06		TRUE	15790	12308	0	3482	yes	mmu-miR-450a-5p			blast	uuuugcgauguguuccuaauau	auuggggaacauuuugcauaaau	uuuugcgauguguuccuaauaugugcuauaauuauauuggggaacauuuugcauaaau	gi|307603363|gb|CM00101
gi|307603371|gb|CM001005.1|_8049	7.7e+3	0.61 ± 0.06		TRUE	15229	11597	24	3608	no	mmu-miR-1843a-5p			blast	uauggaggucucugucugacu	ucugaucguucaccuccauaca	uauggaggucucugucugacuuagaaugcuggcugagucugaucguucaccuccauaca	gi|307603371|gb|CM0010
gi|307603363|gb|CM001013.1|_48969	7.7e+3	0.61 ± 0.06		TRUE	15160	10576	0	4584	yes	mmu-miR-362-5p			blast	aauccuuggaaccuaggugugaau	aacacaccuguucaaggaaucaa	aauccuuggaaccuaggugugaaugcugcuucagugcaacacaccuguucaaggaaucaa	gi|307603363|gb|CM00101
gi|307603371|gb|CM001005.1|_7305	7.6e+3	0.61 ± 0.06		STAR	15030	14574	0	456	yes	mmu-miR-496a-5p			blast	aguauuacauggccaaucucc	gguugcccaugguguguucauu	gguugcccaugguguguucauuuuauuuaugaugaguauuacauggccaaucucc	gi|307603371|gb|CM0010
gi|307603363|gb|CM001013.1|_49198	7.5e+3	0.61 ± 0.06		STAR	14710	12571	0	2139	yes	mmu-miR-542-5p			blast	ugugacagaauugauaacugaaag	cucggggaucaucaugucacg	cucggggaucaucaugucacgagauaccacugugcccuugugacagaauugauaacugaaag	gi|307603363|gb|CM00101
gi|307603372|gb|CM001004.1|_3489	7.1e+3	0.61 ± 0.06		STAR	14068	13611	0	457	yes	mmu-miR-365-2-5p			blast	uaaugccccuaaaaauccuuau	agggacuuucaggggcagcugug	agggacuuucaggggcagcugugguuuccugacucagucauaaugccccuaaaaauccuuau	gi|307603372|gb|CM0010
gi|307603367|gb|CM001009.1|_14511	6.9e+3	0.61 ± 0.06		STAR	13633	13611	0	22	yes	mmu-miR-365-1-5p			blast	uaaugccccuaaaaauccuuau	agggacuuuggggggcagaugug	agggacuuuggggggcagaugugguuuccauuccgcuaucauaaugccccuaaaaauccuuau	gi|307603367|gb|CM0010
gi|307603381|gb|CM000995.1|_24598	6.6e+3	0.61 ± 0.06		TRUE	13047	12482	0	565	yes	mmu-miR-467b-5p			blast	uaagugccugcauguauaugcg	auauacauacacacaccuacac	uaagugccugcauguauaugcguguauauuuuaugcauauacauacacacaccuacac	gi|307603381|gb|CM0009
gi|307603381|gb|CM000995.1|_24580	6.6e+3	0.61 ± 0.06		TRUE	13047	12482	0	565	yes	mmu-miR-467b-5p			blast	uaagugccugcauguauaugcg	auauacauacacacaccuacac	uaagugccugcauguauaugcguguauauuuuaugcauauacauacacacaccuacac	gi|307603381|gb|CM0009
gi|307603381|gb|CM000995.1|_24594	6.6e+3	0.61 ± 0.06		TRUE	13047	12482	0	565	yes	mmu-miR-467b-5p			blast	uaagugccugcauguauaugcg	auauacauacacacaccuacac	uaagugccugcauguauaugcguguauauuuuaugcauauacauacacacaccuacac	gi|307603381|gb|CM0009
gi|307603381|gb|CM000995.1|_24564	6.6e+3	0.61 ± 0.06		TRUE	13047	12482	0	565	yes	mmu-miR-467b-5p			blast	uaagugccugcauguauaugcg	auauacauacacacaccuacac	uaagugccugcauguauaugcguguauauuuuaugcauauacauacacacaccuacac	gi|307603381|gb|CM0009
gi|307603381|gb|CM000995.1|_24610	6.6e+3	0.61 ± 0.06		TRUE	13047	12482	0	565	yes	mmu-miR-467b-5p			blast	uaagugccugcauguauaugcg	auauacauacacacaccuacac	uaagugccugcauguauaugcguguauauuuuaugcauauacauacacacaccuacac	gi|307603381|gb|CM0009
gi|307603381|gb|CM000995.1|_24585	6.6e+3	0.61 ± 0.06		TRUE	13047	12482	0	565	yes	mmu-miR-467b-5p			blast	uaagugccugcauguauaugcg	auauacauacacacaccuacac	uaagugccugcauguauaugcguguauauuuuaugcauauacauacacacaccuacac	gi|307603381|gb|CM0009
gi|307603381|gb|CM000995.1|_24616	6.6e+3	0.61 ± 0.06		TRUE	13047	12482	0	565	yes	mmu-miR-467b-5p			blast	uaagugccugcauguauaugcg	auauacauacacacaccuacac	uaagugccugcauguauaugcguguauauuuuaugcauauacauacacacaccuacac	gi|307603381|gb|CM0009
gi|307603381|gb|CM000995.1|_24604	6.6e+3	0.61 ± 0.06		TRUE	13047	12482	0	565	yes	mmu-miR-467b-5p			blast	uaagugccugcauguauaugcg	auauacauacacacaccuacac	uaagugccugcauguauaugcguguauauuuuaugcauauacauacacacaccuacac	gi|307603381|gb|CM0009
gi|307603381|gb|CM000995.1|_24588	6.6e+3	0.61 ± 0.06		TRUE	13047	12482	0	565	yes	mmu-miR-467b-5p			blast	uaagugccugcauguauaugcg	auauacauacacacaccuacac	uaagugccugcauguauaugcguguauauuuuaugcauauacauacacacaccuacac	gi|307603381|gb|CM0009
gi|307603381|gb|CM000995.1|_24574	6.6e+3	0.61 ± 0.06		TRUE	13047	12482	0	565	yes	mmu-miR-467b-5p			blast	uaagugccugcauguauaugcg	auauacauacacacaccuacac	uaagugccugcauguauaugcguguauauuuuaugcauauacauacacacaccuacac	gi|307603381|gb|CM0009
gi|307603364|gb|CM001012.1|_19846	6.5e+3	0.61 ± 0.06		TRUE	12839	12819	0	20	yes	mmu-miR-192-5p			blast	cugaccuaugaauugacagcca	ggcugccaauuccauaggucaca	cugaccuaugaauugacagccaguacucuuuucucuccucuggcugccaauuccauaggucaca	gi|307603364|gb|CM00101
gi|307603372|gb|CM001004.1|_3340	6.5e+3	0.61 ± 0.06		TRUE	12823	8839	0	3984	no	mmu-miR-212-5p			blast	accuuggcucuagacugcuuacu	uaacagucuccagucacggccac	accuuggcucuagacugcuuacugcccgggccgccuucaguaacagucuccagucacggccac	gi|307603372|gb|CM0010
gi|307603363|gb|CM001013.1|_49195	6.3e+3	0.61 ± 0.06		TRUE	12359	12308	0	51	yes	mmu-miR-450a-5p			blast	uuuugcgauguguuccuaauau	auuggggaugcuuugcauucau	uuuugcgauguguuccuaauauguacuaugaguauauuggggaugcuuugcauucau	gi|307603363|gb|CM00101
gi|307603382|gb|CM000994.1|_24273	6.1e+3	0.61 ± 0.06		TRUE	12037	8776	0	3261	yes	mmu-miR-350-5p			blast	aaagugcaugcgcuuugggaca	uucacaaagcccauacacuuucac	aaagugcaugcgcuuugggacagugaggaaaauaauguucacaaagcccauacacuuucac	gi|307603382|gb|CM0009
gi|307603372|gb|CM001004.1|_3248	5.8e+3	0.61 ± 0.06		TRUE	11539	10648	0	891	yes	mmu-miR-195a-5p			blast	uagcagcacagaaauauuggca	ccaauauuggcugugcugcucca	uagcagcacagaaauauuggcauggggaagugagucugccaauauuggcugugcugcucca	gi|307603372|gb|CM0010
gi|307603371|gb|CM001005.1|_7469	5.8e+3	0.61 ± 0.06		NA	11391	8616	0	2775	yes	mmu-miR-153-5p			blast	uugcauagucacaaaagugaucau	ucauuuuugugacguugcagcu	ucauuuuugugacguugcagcuaguaauaugagcccaguugcauagucacaaaagugaucau	gi|307603371|gb|CM0010
gi|307603378|gb|CM000998.1|_33997	5.7e+3	0.61 ± 0.06		STAR	11199	7438	0	3761	yes	mmu-miR-671-5p			blast	uccgguucucagggcuccacc	aggaagcccuggaggggcuggagg	aggaagcccuggaggggcuggagguggauguuuuccucgguucucagggcuccacc	gi|307603378|gb|CM0009
gi|307603381|gb|CM000995.1|_24622	5.5e+3	0.61 ± 0.06		TRUE	10938	10937	0	1	yes	mmu-miR-467e-5p			blast	auaagugugagcauguauaugu	auauacauacacacaccuau	auaagugugagcauguauaugugugauauauuuuaugcauauacauacacacaccuau	gi|307603381|gb|CM0009
gi|307603375|gb|CM001001.1|_43551	5.5e+3	0.61 ± 0.06		TRUE	10792	6711	128	3953	yes	mmu-miR-138-5p			blast	agcugguguugugaaucaggccg	gcuauuucacgacaccagggu	agcugguguugugaaucaggccgacgagcagcgcauccucuuacccggcuauuucacgacaccagggu	gi|307603375|gb|CM0010
gi|307603374|gb|CM001002.1|_46626	5.4e+3	0.61 ± 0.06		TRUE	10770	9038	0	1732	yes	mmu-miR-138-5p			blast	agcugguguugugaaucaggccg	gcuacuucacaacaccagggc	agcugguguugugaaucaggccguuggccaaucagagaacggcuacuucacaacaccagggc	gi|307603374|gb|CM0010
gi|307603371|gb|CM001005.1|_7319	5.0e+3	0.61 ± 0.06		STAR	9925	9835	0	90	yes	mmu-miR-3072-5p			blast	ugcccccuccaggaagccuucu	agggaccccgagggagggcagg	agggaccccgagggagggcaggcugccccaggccсugcccccuccaggaagccuucu	gi|307603371|gb|CM0010
gi|307603381|gb|CM000995.1|_24569	5.0e+3	0.61 ± 0.06		STAR	9836	9595	0	241	yes	mmu-miR-466c-5p			blast	auacauacacgcacacauaaga	ugaugugugugugcauguacauau	ugaugugugugugcauguacauaugugaauaugauauacauauacauacacgcacacauaaga	gi|307603381|gb|CM0009
gi|307603381|gb|CM000995.1|_24590	5.0e+3	0.61 ± 0.06		STAR	9836	9595	0	241	yes	mmu-miR-466c-5p			blast	auacauacacgcacacauaaga	ugaugugugugugcauguacauau	ugaugugugugugcauguacauaugugaauaugauauacauauacauacacgcacacauaaga	gi|307603381|gb|CM0009
gi|307603381|gb|CM000995.1|_24596	5.0e+3	0.61 ± 0.06		STAR	9827	9595	0	232	yes	mmu-miR-466b-5p			blast	auacauacacgcacacauaaga	ugaugugugguguacauguaca	ugaugugugguguacauguacaugugugaauaugauauacauauacauacacgcacacauaaga	gi|307603381|gb|CM0009
gi|307603381|gb|CM000995.1|_24611	5.0e+3	0.61 ± 0.06		STAR	9827	9595	0	232	yes	mmu-miR-466o-5p			blast	auacauacacgcacacauaaga	ugaugugugguguacauguaca	ugaugugugguguacauguacaugugugaauaugauauacauauacauacacgcacacauaaga	gi|307603381|gb|CM0009
gi|307603381|gb|CM000995.1|_24605	5.0e+3	0.61 ± 0.06		STAR	9827	9595	0	232	yes	mmu-miR-466o-5p			blast	auacauacacgcacacauaaga	ugaugugugguguacauguaca	ugaugugugguguacauguacaugucugaauaugauauacauauacauacacgcacacauaaga	gi|307603381|gb|CM0009
gi|307603381|gb|CM000995.1|_24599	5.0e+3	0.61 ± 0.06		STAR	9827	9595	0	232	yes	mmu-miR-466o-5p			blast	auacauacacgcacacauaaga	ugaugugugguguacauguaca	ugaugugugguguacauguacaugugugaauaugauauacauauacauacacgcacacauaaga	gi|307603381|gb|CM0009
gi|307603381|gb|CM000995.1|_24625	4.9e+3	0.61 ± 0.06		STAR	9643	9595	0	48	yes	mmu-miR-466p-5p			blast	auacauacacgcacacauaaga	uaugugugugguguacauguacau	uaugugugugguguacauguacauaugugaauaugaauccauauacauacacgcacacauaaga	gi|307603381|gb|CM0009
gi|307603377|gb|CM000999.1|_38183	4.8e+3	0.61 ± 0.06		TRUE	9442	9320	0	122	yes	mmu-miR-96-5p			blast	uuuggcacuagcacauuuuugcu	caaucauguguagugccaauau	uuuggcacuagcacauuuuugcuugugucuccgcgugagcaaucauguguagugccaauau	gi|307603377|gb|CM0009
gi|307603381|gb|CM000995.1|_24614	4.4e+3	0.61 ± 0.06		TRUE	8748	6604	0	2144	yes	mmu-miR-669p-5p			blast	aguugugugugcauguucaugucu	acauaacauacacacacacguau	aguugugugugcauguucaugucuauauuugaauauacauaacauacacacacacguau	gi|307603381|gb|CM0009
gi|307603381|gb|CM000995.1|_24592	4.4e+3	0.61 ± 0.06		TRUE	8748	6604	0	2144	yes	mmu-miR-669a-5p			blast	aguugugugugcauguucaugucu	acauaacauacacacacacguau	aguugugugugcauguucaugucuauauuugaauauacauaacauacacacacacguau	gi|307603381|gb|CM0009
gi|307603381|gb|CM000995.1|_24636	4.4e+3	0.61 ± 0.06		TRUE	8748	6604	0	2144	yes	mmu-miR-669p-5p			blast	aguugugugugcauguucaugucu	acauaacauacacacacacguau	aguugugugugcauguucaugucuauauuugaauauacauaacauacacacacacguau	gi|307603381|gb|CM0009
gi|307603381|gb|CM000995.1|_24577	4.4e+3	0.61 ± 0.06		TRUE	8748	6604	0	2144	yes	mmu-miR-669a-5p			blast	aguugugugugcauguucaugucu	acauaacauacacacacacguau	aguugugugugcauguucaugucuauauuugaauauacauaacauacacacacacguau	gi|307603381|gb|CM0009

<table>
<tr><td>gi|307603381|gb|CM000995.1|_24607</td><td>4.4e+3</td><td>0.61 ± 0.06</td><td></td><td>TRUE</td><td>8748</td><td>6604</td><td>0</td><td>2144</td><td>yes</td><td>mmu-miR-669p-5p</td><td></td><td></td><td>blast</td><td>aguugugugugcauguucaugucu</td><td>acauaacauacacacacacguau</td><td>aguugugugugcauguucaugucuauauuugaauauacauaacauacacacacacguau</td><td>gi|307603381|gb|CM00099</td></tr>
<tr><td>gi|307603381|gb|CM000995.1|_24620</td><td>4.4e+3</td><td>0.61 ± 0.06</td><td></td><td>TRUE</td><td>8748</td><td>6604</td><td>0</td><td>2144</td><td>yes</td><td>mmu-miR-669p-5p</td><td></td><td></td><td>blast</td><td>aguugugugugcauguucaugucu</td><td>acauaacauacacacacacguau</td><td>aguugugugugcauguucaugucuauauuugaauauacauaacauacacacacacguau</td><td>gi|307603381|gb|CM00099</td></tr>
<tr><td>gi|307603381|gb|CM000995.1|_24630</td><td>4.4e+3</td><td>0.61 ± 0.06</td><td></td><td>TRUE</td><td>8748</td><td>6604</td><td>0</td><td>2144</td><td>yes</td><td>mmu-miR-669p-5p</td><td></td><td></td><td>blast</td><td>aguugugugugcauguucaugucu</td><td>acauaacauacacacacacguau</td><td>aguugugugugcauguucaugucuauauuugaauauacauaacauacacacacacguau</td><td>gi|307603381|gb|CM00099</td></tr>
<tr><td>gi|307603381|gb|CM000995.1|_24583</td><td>4.4e+3</td><td>0.61 ± 0.06</td><td></td><td>TRUE</td><td>8748</td><td>6604</td><td>0</td><td>2144</td><td>yes</td><td>mmu-miR-669a-5p</td><td></td><td></td><td>blast</td><td>aguugugugugcauguucaugucu</td><td>acauaacauacacacacacguau</td><td>aguugugugugcauguucaugucuauauuugaauauacauaacauacacacacacguau</td><td>gi|307603381|gb|CM00099</td></tr>
<tr><td>gi|307603381|gb|CM000995.1|_24624</td><td>4.2e+3</td><td>0.61 ± 0.06</td><td></td><td>TRUE</td><td>8374</td><td>7809</td><td>0</td><td>565</td><td>yes</td><td>mmu-miR-467d-5p</td><td></td><td></td><td>blast</td><td>auaagugcgcgcauguauaugc</td><td>auauacauacacacaccuacac</td><td>auaagugcgcgcauguauaugcguguauauuuuaugcauauacauacacacaccuacac</td><td>gi|307603381|gb|CM00099</td></tr>
<tr><td>gi|307603378|gb|CM000998.1|_34349</td><td>4.2e+3</td><td>0.61 ± 0.06</td><td></td><td>STAR</td><td>8240</td><td>5691</td><td>0</td><td>2549</td><td>yes</td><td>mmu-miR-574-5p</td><td></td><td></td><td>blast</td><td>cacgcucaugcacacacccaca</td><td>ugagugugugugugugagugugu</td><td>ugagugugugugugugagugugugucgcuccaaguccacgcucaugcacacacccaca</td><td>gi|307603378|gb|CM00099</td></tr>
<tr><td>gi|307603379|gb|CM000997.1|_32471</td><td>3.8e+3</td><td>0.61 ± 0.06</td><td></td><td>TRUE</td><td>7494</td><td>5645</td><td>0</td><td>1849</td><td>yes</td><td>mmu-miR-873a-5p</td><td></td><td></td><td>blast</td><td>ugcaggaacuugugagucucc</td><td>gagacugacaaguucccggga</td><td>ugcaggaacuugugagucuccuauugaaaauagacaggagacugacaaguucccggga</td><td>gi|307603379|gb|CM00099</td></tr>
<tr><td>gi|307603373|gb|CM001003.1|_2043</td><td>3.5e+3</td><td>0.61 ± 0.06</td><td></td><td>TRUE</td><td>7019</td><td>6684</td><td>0</td><td>335</td><td>yes</td><td>mmu-miR-1251-5p</td><td></td><td></td><td>blast</td><td>acucuagcugccaaaggcgc</td><td>cgcuuugcucagccaguguaga</td><td>acucuagcugccaaaggcgcuucuccuucugaacagagcgcuuugcucagccaguguaga</td><td>gi|307603373|gb|CM00100</td></tr>
<tr><td>gi|307603381|gb|CM000995.1|_24617</td><td>3.5e+3</td><td>0.61 ± 0.06</td><td></td><td>STAR</td><td>6958</td><td>6726</td><td>0</td><td>232</td><td>yes</td><td>mmu-miR-466o-5p</td><td></td><td></td><td>blast</td><td>auacauacacgcacacauaaga</td><td>ugauguguguguacauguaca</td><td>ugauguguguguacauguacaugugugaauaugauauacaaauacauacacgcacacauaaga</td><td>gi|307603381|gb|CM00099</td></tr>
<tr><td>gi|307603381|gb|CM000995.1|_24602</td><td>3.4e+3</td><td>0.61 ± 0.06</td><td></td><td>TRUE</td><td>6765</td><td>6604</td><td>0</td><td>161</td><td>yes</td><td>mmu-miR-669a-5p</td><td></td><td></td><td>blast</td><td>aguugugugugcauguucaugucu</td><td>acauaacauacacacacacacgu</td><td>aguugugugugcauguucaugucuauauuugaauauacauaacauacacacacacacgu</td><td>gi|307603381|gb|CM00099</td></tr>
<tr><td>gi|307603374|gb|CM001002.1|_47132</td><td>3.1e+3</td><td>0.61 ± 0.06</td><td></td><td>STAR</td><td>6231</td><td>6156</td><td>0</td><td>75</td><td>yes</td><td>mmu-miR-34b-5p</td><td></td><td></td><td>blast</td><td>aaucacuaacuccacugccauc</td><td>aggcaguguaauuagcugauugu</td><td>aggcaguguaauuagcugauuguagugcggugcugacaaucacuaacuccacugccauc</td><td>gi|307603374|gb|CM00100</td></tr>
<tr><td>gi|307603376|gb|CM001000.1|_41838</td><td>3.0e+3</td><td>0.61 ± 0.06</td><td></td><td>STAR</td><td>5984</td><td>5657</td><td>0</td><td>327</td><td>yes</td><td>mmu-miR-344f-5p</td><td></td><td></td><td>blast</td><td>cucuagccaggaccugacuaca</td><td>agucagucuccuggcuggaguc</td><td>agucagucuccuggcuggaguccagcucuaagcugguuccaggcucuagccaggaccugacuaca</td><td>gi|307603376|gb|CM00100</td></tr>
<tr><td>gi|307603370|gb|CM001006.1|_9439</td><td>2.9e+3</td><td>0.61 ± 0.06</td><td></td><td>STAR</td><td>5860</td><td>4911</td><td>0</td><td>949</td><td>yes</td><td>mmu-miR-582-5p</td><td></td><td></td><td>blast</td><td>aaccuguugaacaacugaaccc</td><td>uacaguuguucaaccaguuacu</td><td>uacaguuguucaaccaguuacuaaucuaacuaauuguaaccuguugaacaacugaaccc</td><td>gi|307603370|gb|CM00100</td></tr>
<tr><td>gi|307603373|gb|CM001003.1|_584</td><td>2.7e+3</td><td>0.61 ± 0.06</td><td></td><td>TRUE</td><td>5457</td><td>5405</td><td>0</td><td>52</td><td>yes</td><td>mmu-miR-1930-5p</td><td></td><td></td><td>blast</td><td>accuccauaguaccugcagcgu</td><td>ggugcaguuacuguggcugugg</td><td>accuccauaguaccugcagcgugcuugacuuucucuauggugcaguuacuguggcugugg</td><td>gi|307603373|gb|CM00100</td></tr>
<tr><td>gi|307603377|gb|CM000999.1|_38153</td><td>2.6e+3</td><td>0.61 ± 0.06</td><td></td><td>TRUE</td><td>5245</td><td>5128</td><td>0</td><td>117</td><td>yes</td><td>mmu-miR-592-5p</td><td></td><td></td><td>blast</td><td>uugugucaauaugcgaugaugu</td><td>ucaucacgguggugacgcaacau</td><td>uugugucaauaugcgaugauguguugugauggcacagcgucaucacgguggugacgcaacau</td><td>gi|307603377|gb|CM00099</td></tr>
<tr><td>gi|307603372|gb|CM001004.1|_2654</td><td>2.6e+3</td><td>0.61 ± 0.06</td><td></td><td>TRUE</td><td>5122</td><td>3523</td><td>0</td><td>1599</td><td>yes</td><td>mmu-miR-216a-5p</td><td></td><td></td><td>blast</td><td>uaaucucagcuggcaacuguga</td><td>cacaguggucucugggauuaug</td><td>uaaucucagcuggcaacugugagaugucccuaucauuccucacaguggucucugggauuaug</td><td>gi|307603372|gb|CM00100</td></tr>
<tr><td>gi|307603367|gb|CM001009.1|_14509</td><td>2.4e+3</td><td>0.61 ± 0.06</td><td></td><td>STAR</td><td>4882</td><td>4794</td><td>0</td><td>88</td><td>yes</td><td>mmu-miR-193b-5p</td><td></td><td></td><td>blast</td><td>aacuggcccacaaagucccgcu</td><td>cggggguuuugagggcgagauga</td><td>cggggguuuugagggcgagaugaguuuguguuuuauccaacuggcccacaaagucccgcu</td><td>gi|307603367|gb|CM00100</td></tr>
<tr><td>gi|307603371|gb|CM001005.1|_8148</td><td>1.9e+3</td><td>0.61 ± 0.06</td><td></td><td>STAR</td><td>3780</td><td>3253</td><td>0</td><td>527</td><td>yes</td><td>mmu-miR-3068-5p</td><td></td><td></td><td>blast</td><td>ggugaauugcaguacuccaaca</td><td>uuggaguucaugcaaguucuaacc</td><td>uuggaguucaugcaaguucuaaccagcuuacucaguagcuggugaauugcaguacuccaaca</td><td>gi|307603371|gb|CM00100</td></tr>
<tr><td>gi|307603372|gb|CM001004.1|_3444</td><td>1.8e+3</td><td>0.61 ± 0.06</td><td></td><td>TRUE</td><td>3659</td><td>2883</td><td>0</td><td>776</td><td>yes</td><td>mmu-miR-144-5p</td><td></td><td></td><td>blast</td><td>ggauaucaucauauacuguaagu</td><td>uacaguauagaugauguacu</td><td>ggauaucaucauauacuguaaguuugugaugagacacuacaguauagaugauguacu</td><td>gi|307603372|gb|CM00100</td></tr>
<tr><td>gi|307603379|gb|CM000997.1|_32692</td><td>1.8e+3</td><td>0.61 ± 0.06</td><td></td><td>STAR</td><td>3620</td><td>2326</td><td>32</td><td>1262</td><td>yes</td><td>mmu-miR-32-5p</td><td></td><td></td><td>blast</td><td>caauuuagugugugugauauu</td><td>uauugcacauuacuaaguugc</td><td>uauugcacauuacuaaguugcauguugucacggccucaaugcaauuuagugugugugauauu</td><td>gi|307603379|gb|CM00099</td></tr>
<tr><td>gi|307603363|gb|CM001013.1|_48797</td><td>1.8e+3</td><td>0.61 ± 0.06</td><td></td><td>STAR</td><td>3536</td><td>2071</td><td>0</td><td>1465</td><td>yes</td><td>mmu-miR-1264-5p</td><td></td><td></td><td>blast</td><td>caaaucuuauuugagcaccugu</td><td>agguccucaauaaguauuuguu</td><td>agguccucaauaaguauuuguugaaaaaauaaaugaagcaacaaaucuuauuugagcaccugu</td><td>gi|307603363|gb|CM00101</td></tr>
<tr><td>gi|307603373|gb|CM001003.1|_754</td><td>1.7e+3</td><td>0.61 ± 0.06</td><td></td><td>TRUE</td><td>3370</td><td>1412</td><td>0</td><td>1958</td><td>yes</td><td>mmu-miR-3057-5p</td><td></td><td></td><td>blast</td><td>auuggagcugagauucugcgggau</td><td>ucccacaggcccagcucauagc</td><td>auuggagcugagauucugcgggauggauuaggaguauauuguccucccacaggcccagcucauagc</td><td>gi|307603373|gb|CM00100</td></tr>
<tr><td>gi|307603376|gb|CM001000.1|_42724</td><td>1.7e+3</td><td>0.61 ± 0.06</td><td></td><td>STAR</td><td>3333</td><td>2912</td><td>0</td><td>421</td><td>yes</td><td>mmu-miR-210-5p</td><td></td><td></td><td>blast</td><td>cugugcgugugacagcggcugau</td><td>agccacugcccaccgcacacugc</td><td>agccacugcccaccgcacacugcguugcuccggacccacugugcgugugacagcggcugau</td><td>gi|307603376|gb|CM00100</td></tr>
<tr><td>gi|307603363|gb|CM001013.1|_48966</td><td>1.5e+3</td><td>0.61 ± 0.06</td><td></td><td>STAR</td><td>3043</td><td>2915</td><td>0</td><td>128</td><td>yes</td><td>mmu-miR-500-5p</td><td></td><td></td><td>blast</td><td>aaugcaccugggcaaggguuca</td><td>aauccuugcuaucugggugcuuag</td><td>aauccuugcuaucugggugcuuagugcuaucucaaugcaaugcaccugggcaaggguuca</td><td>gi|307603363|gb|CM00101</td></tr>
<tr><td>gi|307603367|gb|CM001009.1|_15748</td><td>1.3e+3</td><td>0.61 ± 0.06</td><td></td><td>STAR</td><td>2620</td><td>2589</td><td>0</td><td>31</td><td>yes</td><td>mmu-miR-3081-5p</td><td></td><td></td><td>blast</td><td>uugcgcuccgaucucugagcugg</td><td>agacuggagcuuggagcggugagc</td><td>agacuggagcuuggagcggugagcuggacucgggcuugcgcuccgaucucugagcugg</td><td>gi|307603367|gb|CM00100</td></tr>
<tr><td>gi|307603363|gb|CM001013.1|_49183</td><td>1.2e+3</td><td>0.61 ± 0.06</td><td></td><td>TRUE</td><td>2469</td><td>2431</td><td>0</td><td>38</td><td>yes</td><td>mmu-miR-20b-5p</td><td></td><td></td><td>blast</td><td>caaagugcucauagugcagguag</td><td>acugcagugugagcacuucuagu</td><td>caaagugcucauagugcagguaguuuuuauaccacucuacugcagugugagcacuucuagu</td><td>gi|307603363|gb|CM00101</td></tr>
<tr><td>gi|307603381|gb|CM000995.1|_25452</td><td>1.2e+3</td><td>0.61 ± 0.06</td><td></td><td>TRUE</td><td>2387</td><td>1827</td><td>0</td><td>560</td><td>yes</td><td>mmu-miR-1955-5p</td><td></td><td></td><td>blast</td><td>agucccaggaugcacugcagcuu</td><td>gagcauugcaugcugggacaug</td><td>agucccaggaugcacugcagcuuuuucuuaacuucagauaagagcauugcaugcugggacaug</td><td>gi|307603381|gb|CM00099</td></tr>
<tr><td>gi|307603363|gb|CM001013.1|_49281</td><td>1.1e+3</td><td>0.61 ± 0.06</td><td></td><td>TRUE</td><td>2233</td><td>2146</td><td>0</td><td>87</td><td>yes</td><td>mmu-miR-452-5p</td><td></td><td></td><td>blast</td><td>aacuguuugcagaggaaacugaga</td><td>ucagucucaucugcaaagagg</td><td>aacuguuugcagaggaaacugagacuuuauaacuaugucucagucucaucugcaaagagg</td><td>gi|307603363|gb|CM00101</td></tr>
<tr><td>gi|307603381|gb|CM000995.1|_24554</td><td>9.9e+2</td><td>0.61 ± 0.06</td><td></td><td>TRUE</td><td>1943</td><td>1923</td><td>0</td><td>20</td><td>yes</td><td>mmu-miR-669l-5p</td><td></td><td></td><td>blast</td><td>aguugugugugcauguauaugu</td><td>auauacauacacacccauauau</td><td>aguugugugugcauguauauguguguauaugaauauacauauacauacacacccauauau</td><td>gi|307603381|gb|CM00099</td></tr>
<tr><td>gi|307603379|gb|CM000997.1|_33206</td><td>8.7e+2</td><td>0.61 ± 0.06</td><td></td><td>STAR</td><td>1727</td><td>1675</td><td>0</td><td>52</td><td>no</td><td>mmu-miR-7015-5p</td><td></td><td></td><td>blast</td><td>ucucacuguccucugcacuagg</td><td>ucugugcaguaggcugugggu</td><td>ucugugcaguaggcuguggguuccaugcugacugacuuucucacuguccucugcacuagg</td><td>gi|307603379|gb|CM00099</td></tr>
<tr><td>gi|307603377|gb|CM000999.1|_37021</td><td>7.7e+2</td><td>0.61 ± 0.06</td><td></td><td>STAR</td><td>1510</td><td>755</td><td>0</td><td>755</td><td>yes</td><td>mmu-miR-490-5p</td><td></td><td></td><td>blast</td><td>caaccuggaggacuccaugcug</td><td>ccauggaucuccagguggguca</td><td>ccauggaucuccaggugggucacguuuagauauacaccaaccuggaggacuccaugcug</td><td>gi|307603377|gb|CM00099</td></tr>
<tr><td>gi|307603374|gb|CM001002.1|_47377</td><td>7.2e+2</td><td>0.61 ± 0.06</td><td></td><td>TRUE</td><td>1418</td><td>1128</td><td>0</td><td>290</td><td>yes</td><td>mmu-miR-190a-5p</td><td></td><td></td><td>blast</td><td>ugauauguuugauauauuaggu</td><td>acuauauaucaagcauauuccua</td><td>ugauauguuugauauauuagguuguuauuuaaauccaacuauauaucaagcauauuccua</td><td>gi|307603374|gb|CM00100</td></tr>
<tr><td>gi|307603368|gb|CM001008.1|_14051</td><td>6.9e+2</td><td>0.61 ± 0.06</td><td></td><td>TRUE</td><td>1370</td><td>1341</td><td>0</td><td>29</td><td>no</td><td>mmu-miR-3080-5p</td><td></td><td></td><td>blast</td><td>ugaagcgccuguucuuggauc</td><td>uccucgggcaaagcgcuugaca</td><td>ugaagcgccuguucuuggaucguggcccuguagucauccucgggcaaagcgcuugaca</td><td>gi|307603368|gb|CM00100</td></tr>
<tr><td>gi|307603375|gb|CM001001.1|_43302</td><td>6.5e+2</td><td>0.61 ± 0.06</td><td></td><td>TRUE</td><td>1284</td><td>1242</td><td>0</td><td>42</td><td>yes</td><td>mmu-miR-1969</td><td></td><td></td><td>blast</td><td>aagauggagacuuuaacaugggu</td><td>uccaugcugcccuccauuucu</td><td>aagauggagacuuuaacaugggugcucuuaaaaucuguuaacauccaugcugcccuccauuucu</td><td>gi|307603375|gb|CM00100</td></tr>
<tr><td>gi|307603367|gb|CM001009.1|_14800</td><td>6.4e+2</td><td>0.61 ± 0.06</td><td></td><td>TRUE</td><td>1271</td><td>1245</td><td>0</td><td>26</td><td>yes</td><td>mmu-miR-1947-5p</td><td></td><td></td><td>blast</td><td>aggacgagcuagcugagugcugc</td><td>gcacugagcuagcucucccucc</td><td>aggacgagcuagcugagugcugcagacacucuaagagcacugagcuagcucucccucc</td><td>gi|307603367|gb|CM00100</td></tr>
<tr><td>gi|307603378|gb|CM000998.1|_34710</td><td>6.4e+2</td><td>0.61 ± 0.06</td><td></td><td>TRUE</td><td>1258</td><td>874</td><td>0</td><td>384</td><td>yes</td><td>mmu-miR-701-5p</td><td></td><td></td><td>blast</td><td>uuagccgcugaaauagaugg</td><td>uaucuauuaaagaggcuagcc</td><td>uuagccgcugaaauagauggauuguauagaugagguucuaucuauuaaagaggcuagcc</td><td>gi|307603378|gb|CM00099</td></tr>
<tr><td>gi|307603380|gb|CM000996.1|_28412</td><td>6.1e+2</td><td>0.61 ± 0.06</td><td></td><td>STAR</td><td>1209</td><td>611</td><td>36</td><td>562</td><td>yes</td><td>mmu-miR-551b-5p</td><td></td><td></td><td>blast</td><td>gcgacccauacuugguuucagu</td><td>gaaaucaagcuugggugagaccu</td><td>gaaaucaagcuuggggugagaccuggugcagaacaggaaggcgacccauacuugguuucagu</td><td>gi|307603380|gb|CM00099</td></tr>
<tr><td>gi|307603381|gb|CM000995.1|_27341</td><td>5.7e+2</td><td>0.61 ± 0.06</td><td></td><td>TRUE</td><td>1126</td><td>1098</td><td>0</td><td>28</td><td>yes</td><td>mmu-miR-670-5p</td><td></td><td></td><td>blast</td><td>aucccugaguguauguggugaacc</td><td>uuuccucauauccauucaggagu</td><td>aucccugaguguauguggugaaccugaacuugcccugguuuccucauauccauucaggagu</td><td>gi|307603381|gb|CM00099</td></tr>
<tr><td>gi|307603371|gb|CM001005.1|_7188</td><td>5.4e+2</td><td>0.61 ± 0.06</td><td></td><td>TRUE</td><td>1069</td><td>994</td><td>0</td><td>75</td><td>no</td><td>mmu-miR-1188-5p</td><td></td><td></td><td>blast</td><td>uggugugagguugggccagga</td><td>cgaggcuccccaccacacccugc</td><td>uggugugagguugggccaggaugaaacccaaggcucuccgaggcuccccaccacacccugc</td><td>gi|307603371|gb|CM00100</td></tr>
<tr><td>gi|307603381|gb|CM000995.1|_24695</td><td>5.1e+2</td><td>0.61 ± 0.06</td><td></td><td>STAR</td><td>998</td><td>955</td><td>0</td><td>43</td><td>yes</td><td>mmu-miR-7655-5p</td><td></td><td></td><td>blast</td><td>uuuaugacgcuccgugggccugc</td><td>cggcccacggagcuccuaaagacu</td><td>cggcccacggagcuccuaaagacucgcccugccggguuuaugacgcuccgugggccugc</td><td>gi|307603381|gb|CM00099</td></tr>
<tr><td>gi|307603363|gb|CM001013.1|_48795</td><td>4.0e+2</td><td>0.61 ± 0.06</td><td></td><td>STAR</td><td>788</td><td>775</td><td>0</td><td>13</td><td>yes</td><td>mmu-miR-1912-5p</td><td></td><td></td><td>blast</td><td>cacagaacaugcagugagaacu</td><td>ugcucauugcaugggcugugua</td><td>ugcucauugcaugggcuguguagauuuuuuauuuaauacacagaacaugcagugagaacu</td><td>gi|307603363|gb|CM00101</td></tr>
<tr><td>gi|307603368|gb|CM001008.1|_14140</td><td>4.0e+2</td><td>0.61 ± 0.06</td><td></td><td>STAR</td><td>793</td><td>792</td><td>0</td><td>1</td><td>no</td><td>mmu-miR-6958-5p</td><td></td><td></td><td>blast</td><td>ucagcugccucuugccaucacag</td><td>aggugggagaagaggcug</td><td>aggugggagaagaggcugcugugggugccccaaguccgcuagcucagcuccucagcugccucuugccaucacag</td><td>gi|307603368|gb|CM00100</td></tr>
<tr><td>gi|307603376|gb|CM001000.1|_42766</td><td>3.9e+2</td><td>0.61 ± 0.06</td><td></td><td>STAR</td><td>764</td><td>414</td><td>0</td><td>350</td><td>yes</td><td>mmu-miR-675-5p</td><td></td><td></td><td>blast</td><td>uguaugcccuaaccgcucagu</td><td>uggugcggaaagggcccacagugg</td><td>uggugcggaaagggcccacaguggacuugguacacuguaugcccuaaccgcucagu</td><td>gi|307603376|gb|CM00100</td></tr>
<tr><td>gi|307603363|gb|CM001013.1|_48971</td><td>3.8e+2</td><td>0.61 ± 0.06</td><td></td><td>TRUE</td><td>743</td><td>667</td><td>14</td><td>62</td><td>yes</td><td>mmu-miR-188-5p</td><td></td><td></td><td>blast</td><td>caucccuugcaugguggaggg</td><td>cucccacaugcaggguuugc</td><td>caucccuugcaugguggagggugagcucucugaaaaccccucccacaugcaggguuugc</td><td>gi|307603363|gb|CM00101</td></tr>
<tr><td>gi|307603381|gb|CM000995.1|_24676</td><td>3.7e+2</td><td>0.61 ± 0.06</td><td></td><td>TRUE</td><td>734</td><td>542</td><td>0</td><td>192</td><td>yes</td><td>mmu-miR-511-5p</td><td></td><td></td><td>blast</td><td>caugccuuuugcucugcacuca</td><td>aauguguagcaaaagacaggaug</td><td>caugccuuuugcucugcacucaguaaauaauaauuugugaauguguagcaaaagacaggaug</td><td>gi|307603381|gb|CM00099</td></tr>
<tr><td>gi|307603379|gb|CM000997.1|_31404</td><td>3.4e+2</td><td>0.61 ± 0.06</td><td></td><td>TRUE</td><td>668</td><td>510</td><td>0</td><td>158</td><td>yes</td><td>mmu-miR-491-5p</td><td></td><td></td><td>blast</td><td>aguggggaacccuuccaugagg</td><td>cuuaugcaagauucccuucuac</td><td>aguggggaacccuuccaugaggaguagaacacuccuuaugcaagauucccuucuac</td><td>gi|307603379|gb|CM00099</td></tr>
<tr><td>gi|307603365|gb|CM001011.1|_18340</td><td>3.2e+2</td><td>0.61 ± 0.06</td><td></td><td>TRUE</td><td>638</td><td>375</td><td>0</td><td>263</td><td>yes</td><td>mmu-miR-1948-5p</td><td></td><td></td><td>blast</td><td>auaugaguauucugccuaaau</td><td>uuuaggcagagcacucguacag</td><td>auaugaguauucugccuaaauguggugguacggauacauuuaggcagagcacucguacag</td><td>gi|307603365|gb|CM00101</td></tr>
<tr><td>gi|307603371|gb|CM001005.1|_8324</td><td>3.2e+2</td><td>0.61 ± 0.06</td><td></td><td>STAR</td><td>630</td><td>429</td><td>0</td><td>201</td><td>yes</td><td>mmu-miR-299b-5p</td><td></td><td></td><td>blast</td><td>guauguggacgguaaaccau</td><td>cgguuuaccgucccacauacaua</td><td>cgguuuaccgucccacauacauacucaaaauguauguggacgguaaaccau</td><td>gi|307603371|gb|CM00100</td></tr>
<tr><td>gi|307603376|gb|CM001000.1|_39384</td><td>3.1e+2</td><td>0.61 ± 0.06</td><td></td><td>TRUE</td><td>610</td><td>535</td><td>0</td><td>75</td><td>yes</td><td>mmu-miR-292a-5p</td><td></td><td></td><td>blast</td><td>acucaaacugggggcucuuuu</td><td>aaagugccgccagguuuugagu</td><td>acucaaacugggggcucuuuugauuuucaucggaagaaaagugccgccagguuuugagu</td><td>gi|307603376|gb|CM00100</td></tr>
<tr><td>gi|307603381|gb|CM000995.1|_24557</td><td>2.9e+2</td><td>0.61 ± 0.06</td><td></td><td>NA</td><td>575</td><td>483</td><td>56</td><td>36</td><td>yes</td><td>mmu-miR-297a-5p</td><td></td><td></td><td>blast</td><td>auauacauacgcacauacccau</td><td>guguaugugccuguguguau</td><td>guguaugugccuguguguauauguaugugugugcaugugcauguguguauaugaguauacauauacauacgcacauacccau</td><td>gi|307603381|gb|CM00099</td></tr>
<tr><td>gi|307603379|gb|CM000997.1|_32712</td><td>2.8e+2</td><td>0.61 ± 0.06</td><td></td><td>STAR</td><td>558</td><td>500</td><td>0</td><td>58</td><td>yes</td><td>mmu-miR-3095-5p</td><td></td><td></td><td>blast</td><td>uggacacuggagagagagcuuuu</td><td>caagcuuucucaucugugacacu</td><td>caagcuuucucaucugugacacucgagaugguuucagggaguggacacuggagagagagcuuuu</td><td>gi|307603379|gb|CM00099</td></tr>
<tr><td>gi|307603366|gb|CM001010.1|_17403</td><td>2.7e+2</td><td>0.61 ± 0.06</td><td></td><td>STAR</td><td>553</td><td>397</td><td>0</td><td>156</td><td>no</td><td>mmu-miR-5134-5p</td><td></td><td></td><td>blast</td><td>acgggugcccucuuucugcagg</td><td>uggcagaaagggcagcuguga</td><td>uggcagaaagggcagcugugaugcuuguccuaguuuucacgggugcccucuuucugcagg</td><td>gi|307603366|gb|CM00101</td></tr>
<tr><td>gi|307603381|gb|CM000995.1|_24770</td><td>2.5e+2</td><td>0.61 ± 0.06</td><td></td><td>TRUE</td><td>494</td><td>297</td><td>0</td><td>197</td><td>yes</td><td>mmu-miR-7664-5p</td><td></td><td></td><td>blast</td><td>caguuuaccugucuaugagaug</td><td>ucucuuaggcugguuaacuaau</td><td>caguuuaccugucuaugagauggaaguacuugguaucuaucucuuaggcugguuaacuaau</td><td>gi|307603381|gb|CM00099</td></tr>
<tr><td>gi|307603381|gb|CM000995.1|_26921</td><td>2.3e+2</td><td>0.61 ± 0.06</td><td></td><td>STAR</td><td>464</td><td>291</td><td>0</td><td>173</td><td>yes</td><td>mmu-miR-5128</td><td></td><td></td><td>blast</td><td>ggucuuucuagcuccuguuuu</td><td>aauuggggcuggcgagauggcuu</td><td>ggucuuucuagcuccuguuuuacuaauuuaaaaagugcaauuggggcuggcgagauggcuu</td><td>gi|307603381|gb|CM00099</td></tr>
<tr><td>gi|307603373|gb|CM001003.1|_2091</td><td>2.3e+2</td><td>0.61 ± 0.06</td><td></td><td>TRUE</td><td>457</td><td>388</td><td>0</td><td>69</td><td>yes</td><td>mmu-miR-3058-5p</td><td></td><td></td><td>blast</td><td>ucagccacggcuuaccuggaag</td><td>uuccugucagccgugggugccu</td><td>ucagccacggcuuaccuggaagauugaaugcuacuuggcuuucuuccugucagccgugggugccu</td><td>gi|307603373|gb|CM00100</td></tr>
<tr><td>gi|307603372|gb|CM001004.1|_2895</td><td>2.3e+2</td><td>0.61 ± 0.06</td><td></td><td>STAR</td><td>452</td><td>295</td><td>0</td><td>157</td><td>yes</td><td>mmu-miR-3061-5p</td><td></td><td></td><td>blast</td><td>cuaccuuugauaguccacugcc</td><td>caguggccgugaaagguagc</td><td>caguggccgugaaagguagccugguggucccuuugagcuaaggcuaccuuugauaguccacugcc</td><td>gi|307603372|gb|CM00100</td></tr>
<tr><td>gi|307603366|gb|CM001010.1|_16577</td><td>2.1e+2</td><td>0.61 ± 0.06</td><td></td><td>STAR</td><td>424</td><td>389</td><td>0</td><td>35</td><td>no</td><td>mmu-miR-6975-5p</td><td></td><td></td><td>blast</td><td>ucucuccuuucuccuccuagu</td><td>gcuggggagaaaggggguuuggcac</td><td>gcuggggagaaaggggguuuggcacggugcaaaaaugucucuccuuucuccuccuagu</td><td>gi|307603366|gb|CM00101</td></tr>
<tr><td>gi|307603375|gb|CM001001.1|_42907</td><td>2.0e+2</td><td>0.61 ± 0.06</td><td></td><td>TRUE</td><td>412</td><td>411</td><td>0</td><td>1</td><td>no</td><td>mmu-miR-9768-5p</td><td></td><td></td><td>blast</td><td>uggcacucggaggacggcacugu</td><td>cugccgccgccgcugccg</td><td>cugccgccgccgcugccgcagcagccgcagccuguggcacucggaggacggcacugu</td><td>gi|307603375|gb|CM00100</td></tr>
<tr><td>gi|307603375|gb|CM001001.1|_44021</td><td>2.0e+2</td><td>0.61 ± 0.06</td><td></td><td>TRUE</td><td>392</td><td>376</td><td>0</td><td>16</td><td>yes</td><td>mmu-miR-7654-5p</td><td></td><td></td><td>blast</td><td>ggagucgcgcucccgcucgcg</td><td>cgagcgggagcgcgccucguc</td><td>ggagucgcgcucccgcucgcgcuccaccaacgcggcggcaucccggcgcgagcgggagcgcgccucguc</td><td>gi|307603375|gb|CM00100</td></tr>
<tr><td>gi|307603378|gb|CM000998.1|_33900</td><td>1.8e+2</td><td>0.61 ± 0.06</td><td></td><td>TRUE</td><td>367</td><td>352</td><td>0</td><td>15</td><td>yes</td><td>mmu-miR-879-5p</td><td></td><td></td><td>blast</td><td>agaggcuuauagcucuaagcc</td><td>cuuauggcuucaagcuuucgga</td><td>agaggcuuauagcucuaagccuuggaugaaagaggcuuauggcuucaagcuuucgga</td><td>gi|307603378|gb|CM00099</td></tr>
<tr><td>gi|307603377|gb|CM000999.1|_37720</td><td>1.8e+2</td><td>0.61 ± 0.06</td><td></td><td>TRUE</td><td>352</td><td>313</td><td>0</td><td>39</td><td>yes</td><td>mmu-miR-7660-5p</td><td></td><td></td><td>blast</td><td>cuuugcccuggcuucagaucu</td><td>aucuguaagugaugggcaaagua</td><td>cuuugcccuggcuucagaucucggcaaccaucacuaacuagaucuguaagugaugggcaaagua</td><td>gi|307603377|gb|CM00099</td></tr>
<tr><td>gi|307603368|gb|CM001008.1|_13355</td><td>1.7e+2</td><td>0.61 ± 0.06</td><td></td><td>STAR</td><td>346</td><td>325</td><td>0</td><td>21</td><td>no</td><td>mmu-miR-6962-5p</td><td></td><td></td><td>blast</td><td>uaucugcccuucccuguccuaua</td><td>aaaggaccugguggggagaucuc</td><td>aaaggaccugguggggagaucucacaucacuguugauaucugcccuucccuguccuaua</td><td>gi|307603368|gb|CM00100</td></tr>
<tr><td>gi|307603376|gb|CM001000.1|_39389</td><td>1.7e+2</td><td>0.61 ± 0.06</td><td></td><td>STAR</td><td>337</td><td>328</td><td>0</td><td>9</td><td>yes</td><td>mmu-miR-295-5p</td><td></td><td></td><td>blast</td><td>aagugcuacuacuuuugagucu</td><td>acucaaaugugggcacacuucu</td><td>acucaaaugugggcacacuucuggacuguacauagaaagugcuacuacuuuugagucu</td><td>gi|307603376|gb|CM00100</td></tr>
<tr><td>gi|307603372|gb|CM001004.1|_4570</td><td>1.6e+2</td><td>0.61 ± 0.06</td><td></td><td>STAR</td><td>331</td><td>240</td><td>0</td><td>91</td><td>yes</td><td>mmu-miR-1933-5p</td><td></td><td></td><td>blast</td><td>ccaggaccaucagugugacuauu</td><td>cagucaugguguucggucuuagu</td><td>cagucaugguguucggucuuaguuugugaccaguuuaguuuaaccaggaccaucagugugacuauu</td><td>gi|307603372|gb|CM00100</td></tr>
<tr><td>gi|307603378|gb|CM000998.1|_34215</td><td>1.6e+2</td><td>0.61 ± 0.06</td><td></td><td>TRUE</td><td>323</td><td>253</td><td>0</td><td>70</td><td>yes</td><td>mmu-miR-3097-5p</td><td></td><td></td><td>blast</td><td>acacaggugggaagugugugucca</td><td>cucagaccuuucuaccugucag</td><td>acacaggugggaagugugugucccagugggugugucuacagaucugucagaccuuucuaccugucag</td><td>gi|307603378|gb|CM00099</td></tr>
<tr><td>gi|307603376|gb|CM001000.1|_41225</td><td>1.5e+2</td><td>0.61 ± 0.06</td><td></td><td>TRUE</td><td>304</td><td>256</td><td>0</td><td>48</td><td>yes</td><td>mmu-miR-3101-5p</td><td></td><td></td><td>blast</td><td>gguaccauugacuaaagcuag</td><td>agcuuugguggauggucuuugc</td><td>gguaccauugacuaaagcuaguacuauaggcuugccucuagcuuugguggauggucuuugc</td><td>gi|307603376|gb|CM00100</td></tr>
<tr><td>gi|307603363|gb|CM001013.1|_48794</td><td>1.5e+2</td><td>0.61 ± 0.06</td><td></td><td>TRUE</td><td>303</td><td>289</td><td>0</td><td>14</td><td>yes</td><td>mmu-miR-764-5p</td><td></td><td></td><td>blast</td><td>ggugcucacauguccuccucca</td><td>gaggaggccauaguggcaacuguc</td><td>ggugcucacauguccuccuccaugcuuauaaaauacauggaggaggccauaguggcaacuguc</td><td>gi|307603363|gb|CM00101</td></tr>
<tr><td>gi|307603379|gb|CM000997.1|_33841</td><td>1.5e+2</td><td>0.61 ± 0.06</td><td></td><td>TRUE</td><td>296</td><td>201</td><td>0</td><td>95</td><td>yes</td><td>mmu-miR-7658-5p</td><td></td><td></td><td>blast</td><td>uguggcguggcggugcugga</td><td>accaccuucccucacccacgcug</td><td>uguggcguggcggugcuggagcugcucacuucccaccaccuucccucacccacgcug</td><td>gi|307603379|gb|CM00099</td></tr>
<tr><td>gi|307603373|gb|CM001003.1|_1903</td><td>1.5e+2</td><td>0.61 ± 0.06</td><td></td><td>TRUE</td><td>292</td><td>270</td><td>0</td><td>22</td><td>yes</td><td>mmu-miR-5615-5p</td><td></td><td></td><td>blast</td><td>cuugguuguuuucugagacaga</td><td>ugucucagaaaacaaccaaggac</td><td>cuugguuguuuucugagacagaaaagagucucauuuucugucucagaaaacaaccaaggac</td><td>gi|307603373|gb|CM00100</td></tr>
<tr><td>gi|307603382|gb|CM000994.1|_23890</td><td>1.5e+2</td><td>0.61 ± 0.06</td><td></td><td>STAR</td><td>291</td><td>238</td><td>19</td><td>34</td><td>yes</td><td>mmu-miR-1231-5p</td><td></td><td></td><td>blast</td><td>ugcccugucuguucugcccacag</td><td>cugggcagagcugcaggagaga</td><td>cugggcagagcugcaggagagaagggcagggccggacaucugcccacccugcccaccugcccugucuguucugcccacag</td><td>gi|307603382|gb|CM00099</td></tr>
<tr><td>gi|307603376|gb|CM001000.1|_39385</td><td>1.4e+2</td><td>0.61 ± 0.06</td><td></td><td>STAR</td><td>290</td><td>244</td><td>0</td><td>46</td><td>yes</td><td>mmu-miR-293-5p</td><td></td><td></td><td>blast</td><td>agugccgcagaguuuguagugu</td><td>acucaaacugugugacauuuu</td><td>acucaaacugugugacauuuuguucuuuguaagaagugccgcagaguuuguagugu</td><td>gi|307603376|gb|CM00100</td></tr>
<tr><td>gi|307603373|gb|CM001003.1|_1353</td><td>1.4e+2</td><td>0.61 ± 0.06</td><td></td><td>TRUE</td><td>279</td><td>234</td><td>0</td><td>45</td><td>yes</td><td>mmu-miR-7663-5p</td><td></td><td></td><td>blast</td><td>ggcugcuuggugaucauccacugu</td><td>aguguggaucauaaggagcucc</td><td>ggcugcuuggugaucauccacugucguggggacuucuucacaguguggaucauaaggagcucc</td><td>gi|307603373|gb|CM00100</td></tr>
<tr><td>gi|307603368|gb|CM001008.1|_14145</td><td>1.3e+2</td><td>0.61 ± 0.06</td><td></td><td>TRUE</td><td>267</td><td>253</td><td>0</td><td>14</td><td>yes</td><td>mmu-miR-6959-5p</td><td></td><td></td><td>blast</td><td>ugggaaaccugugucgggcuguga</td><td>cugcaccgaccugcucucccagg</td><td>ugggaaaccugugucgggcugugaguguuuucugggaccugauacucacugcaccgaccugcucucccagg</td><td>gi|307603368|gb|CM00100</td></tr>
<tr><td>gi|307603368|gb|CM001008.1|_13925</td><td>1.3e+2</td><td>0.61 ± 0.06</td><td></td><td>TRUE</td><td>262</td><td>203</td><td>0</td><td>59</td><td>yes</td><td>mmu-miR-1943-5p</td><td></td><td></td><td>blast</td><td>aagggaggaucugggcaccugga</td><td>caggugccagcuccucccuuc</td><td>aagggaggaucugggcaccuggaccagcucucccugcaggugccagcuccucccuuc</td><td>gi|307603368|gb|CM00100</td></tr>
<tr><td>gi|307603376|gb|CM001000.1|_39760</td><td>1.3e+2</td><td>0.61 ± 0.06</td><td></td><td>TRUE</td><td>258</td><td>227</td><td>0</td><td>31</td><td>yes</td><td>mmu-miR-7668-5p</td><td></td><td></td><td>blast</td><td>ucugaauaaccugcucucugu</td><td>cggugcaggggauucagaggaga</td><td>ucugaauaaccugcucucuguggaagaaaccaugcggugcaggggauucagaggaga</td><td>gi|307603376|gb|CM00100</td></tr>
<tr><td>gi|307603381|gb|CM000995.1|_24638</td><td>1.2e+2</td><td>0.61 ± 0.06</td><td></td><td>TRUE</td><td>239</td><td>224</td><td>0</td><td>15</td><td>yes</td><td>mmu-miR-669b-5p</td><td></td><td></td><td>blast</td><td>aguuuugugugcaugugcaugug</td><td>auauacauccacacaaacauau</td><td>aguuuugugugcaugugcauguguguauaugaauagacauauacauccacacaaacauau</td><td>gi|307603381|gb|CM00099</td></tr>
<tr><td>gi|307603374|gb|CM001002.1|_47403</td><td>1.1e+2</td><td>0.61 ± 0.06</td><td></td><td>TRUE</td><td>222</td><td>134</td><td>0</td><td>88</td><td>yes</td><td>mmu-miR-5626-5p</td><td></td><td></td><td>blast</td><td>gccccaucgauuaacugcuucc</td><td>cagcaguugagugaugugacac</td><td>gccccaucgauuaacugcuuccuguucucagacccggcagcaguugagugaugugacac</td><td>gi|307603374|gb|CM00100</td></tr>
<tr><td>gi|307603382|gb|CM000994.1|_24052</td><td>1.1e+2</td><td>0.61 ± 0.06</td><td></td><td>TRUE</td><td>214</td><td>196</td><td>0</td><td>18</td><td>yes</td><td>mmu-miR-1927</td><td></td><td></td><td>blast</td><td>gaccucuggauguuaggacuga</td><td>gaucccuagaaaccagaguugc</td><td>gaucccuagaaaccagaguugcaaguggcugugacugaccucuggauguuaggacuga</td><td>gi|307603382|gb|CM00099</td></tr>
<tr><td>gi|307603365|gb|CM001011.1|_18766</td><td>9.3e+1</td><td>0.61 ± 0.06</td><td></td><td>TRUE</td><td>188</td><td>139</td><td>0</td><td>49</td><td>no</td><td>mmu-miR-7220-5p</td><td></td><td></td><td>blast</td><td>ggugagcucuugguaccuugg</td><td>ccaggaacaaggucucaguccga</td><td>ggugagcucuugguaccuuggcgacaagaaggugagcccaggaacaaggucucaguccga</td><td>gi|307603365|gb|CM00101</td></tr>
<tr><td>gi|307603375|gb|CM001001.1|_43857</td><td>9.0e+1</td><td>0.61 ± 0.06</td><td></td><td>STAR</td><td>174</td><td>159</td><td>0</td><td>15</td><td>yes</td><td>mmu-miR-7237-5p</td><td></td><td></td><td>blast</td><td>cauccuguugagcuuaccgagc</td><td>augguagcuugugacaggacugg</td><td>augguagcuugugacaggacuggcccuugggagagcaaagccauccuguugagcuuaccgagc</td><td>gi|307603375|gb|CM00100</td></tr>
<tr><td>gi|307603375|gb|CM001001.1|_43643</td><td>8.7e+1</td><td>0.61 ± 0.06</td><td></td><td>STAR</td><td>179</td><td>161</td><td>0</td><td>18</td><td>no</td><td>mmu-miR-1966-5p</td><td></td><td></td><td>blast</td><td>uuucugacucaacucucccuuagc</td><td>aagggagcuggcucaggagaga</td><td>aagggagcuggcucaggagagaguccugagauuuaggcucuuucugacucaacucucccuuagc</td><td>gi|307603375|gb|CM00100</td></tr>
<tr><td>gi|307603378|gb|CM000998.1|_36117</td><td>7.0e+1</td><td>0.61 ± 0.06</td><td></td><td>TRUE</td><td>145</td><td>131</td><td>0</td><td>14</td><td>no</td><td>mmu-miR-7026-5p</td><td></td><td></td><td>blast</td><td>uucugagaccaugggguauaug</td><td>ugugcuuucuggucuuggcuuagg</td><td>uucugagaccaugggguauaugcagugguucugguaacaugugcuuucuggucuuggcuuagg</td><td>gi|307603378|gb|CM00099</td></tr>
<tr><td>gi|307603366|gb|CM001010.1|_16324</td><td>6.6e+1</td><td>0.61 ± 0.06</td><td></td><td>TRUE</td><td>127</td><td>116</td><td>0</td><td>11</td><td>yes</td><td>mmu-miR-6966-5p</td><td></td><td></td><td>blast</td><td>ccucggggauguggacagcaca</td><td>ugcugugugcuuccucugcag</td><td>ccucggggauguggacagcacagccaucucagaucaaugagagcaugugcugugugcuuccucugcag</td><td>gi|307603366|gb|CM00101</td></tr>
<tr><td>gi|307603375|gb|CM001001.1|_43445</td><td>6.5e+1</td><td>0.61 ± 0.06</td><td></td><td>STAR</td><td>134</td><td>64</td><td>0</td><td>70</td><td>no</td><td>mmu-miR-7069-5p</td><td></td><td></td><td>blast</td><td>ccugcccugccaaccccuaca</td><td>uuugggggccugggagggugaca</td><td>uuugggggccugggagggugacacugaggucaucagccugcccugccaaccccuaca</td><td>gi|307603375|gb|CM00100</td></tr>
<tr><td>gi|307603382|gb|CM000994.1|_22124</td><td>5.9e+1</td><td>0.61 ± 0.06</td><td></td><td>TRUE</td><td>123</td><td>111</td><td>0</td><td>12</td><td>no</td><td>mmu-miR-6902-5p</td><td></td><td></td><td>blast</td><td>uguucccucacauugucacaugaag</td><td>ccaugugaugugugguucagu</td><td>uguucccucacauugucacaugaaguagaugugaauuugcaccaugugaugugugguucagu</td><td>gi|307603382|gb|CM00099</td></tr>
<tr><td>gi|307603363|gb|CM001013.1|_49259</td><td>5.4e+1</td><td>0.61 ± 0.06</td><td></td><td>TRUE</td><td>106</td><td>48</td><td>0</td><td>58</td><td>yes</td><td>mmu-miR-465c-5p</td><td></td><td></td><td>blast</td><td>uauuuagaauggcgcugaucug</td><td>ugaucagggccuuucuaaguag</td><td>uauuuagaauggcgcugaucugaugaauaaaaaaaaaaugaucagggccuuucuaaguag</td><td>gi|307603363|gb|CM00101</td></tr>
<tr><td>gi|307603366|gb|CM001010.1|_17481</td><td>5.4e+1</td><td>0.61 ± 0.06</td><td></td><td>TRUE</td><td>105</td><td>91</td><td>0</td><td>14</td><td>yes</td><td>mmu-miR-3083-5p</td><td></td><td></td><td>blast</td><td>uaaaggcugggaauauuuca</td><td>aacauucccagccuuuacu</td><td>uaaaggcugggaauauuucagagauauauauguauaucuccgaaacauucccagccuuuacu</td><td>gi|307603366|gb|CM00101</td></tr>
<tr><td>gi|307603368|gb|CM001008.1|_13965</td><td>5.4e+1</td><td>0.61 ± 0.06</td><td></td><td>TRUE</td><td>103</td><td>68</td><td>0</td><td>35</td><td>yes</td><td>mmu-miR-7213-5p</td><td></td><td></td><td>blast</td><td>acuggucucuugagguagcc</td><td>uaccucaagagagccagucuga</td><td>acuggucucuugagguagccugaaaggaagggggcuaccucaagagagccagucuga</td><td>gi|307603368|gb|CM00100</td></tr>
<tr><td>gi|307603376|gb|CM001000.1|_39382</td><td>5.4e+1</td><td>0.61 ± 0.06</td><td></td><td>TRUE</td><td>103</td><td>91</td><td>0</td><td>12</td><td>yes</td><td>mmu-miR-291a-5p</td><td></td><td></td><td>blast</td><td>caucaaaguggaggcccucucu</td><td>aaagugcuuccacuuugugugcc</td><td>caucaaaguggaggcccucucuugagccugaaugagaaagugcuuccacuuugugugcc</td><td>gi|307603376|gb|CM00100</td></tr>
<tr><td>gi|307603364|gb|CM001012.1|_20597</td><td>4.5e+1</td><td>0.61 ± 0.06</td><td></td><td>TRUE</td><td>87</td><td>79</td><td>0</td><td>8</td><td>yes</td><td>mmu-miR-6986-5p</td><td></td><td></td><td>blast</td><td>gaggggagcuagguagaaagcca</td><td>guuuucaccuucccucccagc</td><td>gaggggagcuagguagaaagccagaggcauaugugguuuucaccuucccucccagc</td><td>gi|307603364|gb|CM00101</td></tr>
<tr><td>gi|307603380|gb|CM000996.1|_28898</td><td>4.3e+1</td><td>0.61 ± 0.06</td><td></td><td>TRUE</td><td>90</td><td>54</td><td>0</td><td>36</td><td>no</td><td>mmu-miR-7012-5p</td><td></td><td></td><td>blast</td><td>aaggagaggaguuggcagggacu</td><td>ugaccuguggcuccucucccag</td><td>aaggagaggaguuggcagggacuagcuaugagcauccugaccuguggcuccucucccag</td><td>gi|307603380|gb|CM00099</td></tr>
<tr><td>gi|307603371|gb|CM001005.1|_7759</td><td>4.1e+1</td><td>0.61 ± 0.06</td><td></td><td>TRUE</td><td>77</td><td>46</td><td>0</td><td>31</td><td>yes</td><td>mmu-miR-1938</td><td></td><td></td><td>blast</td><td>cggugggacuuguaguucgguc</td><td>cugaacugcaguucccaucaugc</td><td>cggugggacuuguaguucggucuucgcuucggacgcccaagucugcauagcgcgugguacggcugaacugcaguucccaucaugc</td><td>gi|307603371|gb|CM00100</td></tr>
<tr><td>gi|307603376|gb|CM001000.1|_39387</td><td>4.1e+1</td><td>0.61 ± 0.06</td><td></td><td>STAR</td><td>79</td><td>69</td><td>0</td><td>10</td><td>yes</td><td>mmu-miR-294-5p</td><td></td><td></td><td>blast</td><td>aaagugcuucccuuuugugugu</td><td>acucaaaauggaggcccuaucu</td><td>acucaaaauggaggcccuaucuaagcuuuuaaguggaaagugcuucccuuuugugugu</td><td>gi|307603376|gb|CM00100</td></tr>
<tr><td>gi|307603372|gb|CM001004.1|_6164</td><td>3.0</td><td>0.64 ± 0.05</td><td></td><td>STAR</td><td>181</td><td>181</td><td>0</td><td>0</td><td>yes</td><td>mmu-miR-6936-5p</td><td></td><td></td><td>blast</td><td>cggcgcgcggcgcucacc</td><td>ugagccacugccgugcccgc</td><td>ugagccacugccgugcccgcgcucgcgccggcgcgcggcgcucacc</td><td>gi|307603372|gb|CM00100</td></tr>
<tr><td>gi|307603368|gb|CM001008.1|_13843</td><td>2.8</td><td>0.81 ± 0.02</td><td></td><td>TRUE</td><td>294</td><td>294</td><td>0</td><td>0</td><td>yes</td><td>mmu-miR-3079-5p</td><td></td><td></td><td>blast</td><td>uuugaucugaugagcuaagcugga</td><td>caggcucaucagagaaagu</td><td>uuugaucugaugagcuaagcuggaguucccauucaccuggcuccaggcucaucagagaaagu</td><td>gi|307603368|gb|CM00100</td></tr>
<tr><td>gi|307603381|gb|CM000995.1|_24639</td><td>2.7</td><td>0.81 ± 0.02</td><td></td><td>STAR</td><td>238</td><td>238</td><td>0</td><td>0</td><td>yes</td><td>mmu-miR-466d-5p</td><td></td><td></td><td>blast</td><td>uauacaugagagcauacauaga</td><td>ugugugugcguacauguacaug</td><td>ugugugugcguacauguacauguguguauaugaauaugcauauacaugagagcauacauaga</td><td>gi|307603381|gb|CM00099</td></tr>
<tr><td>gi|307603381|gb|CM000995.1|_26104</td><td>2.7</td><td>0.81 ± 0.02</td><td></td><td>TRUE</td><td>2651</td><td>2651</td><td>0</td><td>0</td><td>yes</td><td>mmu-miR-499-5p</td><td></td><td></td><td>blast</td><td>uuaagacuugcagugauguuu</td><td>acaucacagcaagucugugcu</td><td>uuaagacuugcagugauguuuagcuccucugcaugugaacauacacagcaagucugugcu</td><td>gi|307603381|gb|CM00099</td></tr>
<tr><td>gi|307603376|gb|CM001000.1|_41832</td><td>2.7</td><td>0.81 ± 0.02</td><td></td><td>STAR</td><td>160933</td><td>160684</td><td>0</td><td>249</td><td>yes</td><td>mmu-miR-344-5p</td><td></td><td></td><td>blast</td><td>ugaucuagccaaagccugacugu</td><td>ucaggcuccuggcuagauucca</td><td>ucaggcuccuggcuagauuccagguaccagcugguaccugaucuagccaaagccugacugu</td><td>gi|307603376|gb|CM00100</td></tr>
<tr><td>gi|307603369|gb|CM001007.1|_10990</td><td>2.7</td><td>0.81 ± 0.02</td><td></td><td>TRUE</td><td>38280</td><td>38280</td><td>0</td><td>0</td><td>yes</td><td>mmu-miR-346-5p</td><td></td><td></td><td>blast</td><td>ugucugcccgagugccugccucu</td><td>aggcaggggcugggccugcagc</td><td>ugucugcccgagugccugccucucuguugcucugaaggaggcaggggcugggccugcagc</td><td>gi|307603369|gb|CM00100</td></tr>
<tr><td>gi|307603381|gb|CM000995.1|_24633</td><td>2.7</td><td>0.81 ± 0.02</td><td></td><td>STAR</td><td>3712</td><td>3712</td><td>0</td><td>0</td><td>yes</td><td>mmu-miR-466d-5p</td><td></td><td></td><td>blast</td><td>uauacauacacgcacacauaaga</td><td>ugugugugcguacauguacaug</td><td>ugugugugcguacauguacauguguguauaugaauaugaauacauauacauacacgcacacauaaga</td><td>gi|307603381|gb|CM00099</td></tr>
<tr><td>gi|307603377|gb|CM000999.1|_39080</td><td>2.7</td><td>0.81 ± 0.02</td><td></td><td>STAR</td><td>119428</td><td>119416</td><td>12</td><td>0</td><td>yes</td><td>mmu-miR-200c-5p</td><td></td><td></td><td>blast</td><td>uaauacugccggguaaugaugga</td><td>cgucuuacccagcaguguuugg</td><td>cgucuuacccagcaguguuuggggugcuugguugggagucucuaauacugccggguaaugaugga</td><td>gi|307603377|gb|CM00099</td></tr>
<tr><td>gi|307603364|gb|CM001012.1|_20490</td><td>2.7</td><td>0.81 ± 0.02</td><td></td><td>TRUE</td><td>75</td><td>75</td><td>0</td><td>0</td><td>yes</td><td>mmu-miR-6715-5p</td><td></td><td></td><td>blast</td><td>acaggcaccacagguuugagc</td><td>ccaaaccaggcgugccugugg</td><td>acaggcaccacagguuugagcauuuugauugaauugccaaaccaggcgugccugugg</td><td>gi|307603364|gb|CM00101</td></tr>
<tr><td>gi|307603366|gb|CM001010.1|_16520</td><td>2.7</td><td>0.81 ± 0.02</td><td></td><td>TRUE</td><td>677</td><td>677</td><td>0</td><td>0</td><td>yes</td><td>mmu-miR-219c-5p</td><td></td><td></td><td>blast</td><td>ggacguccagacgcaacucucg</td><td>agaauugcguuuggacaaucagg</td><td>ggacguccagacgcaacucucggccggagccagagacucgagaauugcguuuggacaaucagg</td><td>gi|307603366|gb|CM00101</td></tr>
<tr><td>gi|307603363|gb|CM001013.1|_49070</td><td>2.6</td><td>0.81 ± 0.02</td><td></td><td>STAR</td><td>44902</td><td>44537</td><td>0</td><td>365</td><td>yes</td><td>mmu-miR-222-5p</td><td></td><td></td><td>blast</td><td>agcuacaucuggcuacugggucu</td><td>ucaguagccaguguagauccugu</td><td>ucaguagccaguguagauccuguuuugguaaucagcagcuacaucuggcuacugggucu</td><td>gi|307603363|gb|CM00101</td></tr>
<tr><td>gi|307603375|gb|CM001001.1|_43829</td><td>2.6</td><td>0.81 ± 0.02</td><td></td><td>STAR</td><td>199</td><td>199</td><td>0</td><td>0</td><td>yes</td><td>mmu-miR-7687-5p</td><td></td><td></td><td>blast</td><td>ccacucagucgccccgccaca</td><td>aggcggggaaccugaggcgcaggcu</td><td>aggcggggaaccugaggcgcaggcugugucugacucagccacucagucgccccgccaca</td><td>gi|307603375|gb|CM00100</td></tr>
<tr><td>gi|307603375|gb|CM001001.1|_44872</td><td>2.6</td><td>0.81 ± 0.02</td><td></td><td>STAR</td><td>128091</td><td>128069</td><td>22</td><td>0</td><td>yes</td><td>mmu-miR-328-5p</td><td></td><td></td><td>blast</td><td>cuggcccucucugcccuuccgu</td><td>aggggggcaggaggggcucaggg</td><td>agggggcaggaggggcucagggagaaaguaucuacagccccuggcccucucugcccuuccgu</td><td>gi|307603375|gb|CM00100</td></tr>
<tr><td>gi|307603372|gb|CM001004.1|_3220</td><td>2.6</td><td>0.81 ± 0.02</td><td></td><td>TRUE</td><td>303</td><td>303</td><td>0</td><td>0</td><td>yes</td><td>mmu-miR-1934-5p</td><td></td><td></td><td>blast</td><td>ucugguccccugcuucguccuc</td><td>ggaugacgguggggcugguga</td><td>ucugguccccugcuucguccucuguccagauguuaagaggaugacgguggggcugguga</td><td>gi|307603372|gb|CM00100</td></tr>
<tr><td>gi|307603374|gb|CM001002.1|_46782</td><td>2.6</td><td>0.81 ± 0.02</td><td></td><td>STAR</td><td>102</td><td>102</td><td>0</td><td>0</td><td>yes</td><td>mmu-miR-7082-5p</td><td></td><td></td><td>blast</td><td>gcccccuugcugucugucucc</td><td>gguacgggcaggaggaggggaggcac</td><td>gguacgggcaggaggagggggaggcacaggcuggcaacccuggcucugggaggccacugaccuggccccccuugcugucugucucc</td><td>gi|307603374|gb|CM00100</td></tr>
<tr><td>gi|307603369|gb|CM001007.1|_11359</td><td>2.6</td><td>0.81 ± 0.02</td><td></td><td>STAR</td><td>302363</td><td>302363</td><td>0</td><td>0</td><td>yes</td><td>mmu-miR-320-5p</td><td></td><td></td><td>blast</td><td>aaaagcuggguugagagggcga</td><td>gccuucucuucccgguucuucc</td><td>gccuucucuucccgguucuucccggagucgggaaaagcuggguugagagggcga</td><td>gi|307603369|gb|CM00100</td></tr>
<tr><td>gi|307603372|gb|CM001004.1|_3246</td><td>2.6</td><td>0.81 ± 0.02</td><td></td><td>TRUE</td><td>4720</td><td>4720</td><td>0</td><td>0</td><td>yes</td><td>mmu-miR-497a-5p</td><td></td><td></td><td>blast</td><td>cagcagcacacugugguuugua</td><td>caaaccacacuguggguguuaga</td><td>cagcagcacacugugguuuguacggcacuguggccacguccaaaccacacuguggguguuaga</td><td>gi|307603372|gb|CM00100</td></tr>
<tr><td>gi|307603372|gb|CM001004.1|_5135</td><td>2.6</td><td>0.81 ± 0.02</td><td></td><td>TRUE</td><td>49</td><td>49</td><td>0</td><td>0</td><td>yes</td><td>mmu-miR-497b</td><td></td><td></td><td>blast</td><td>accacagugugguuuggacgugg</td><td>ccguacaaaccacagugugcugc</td><td>accacagugugguuuggacguggccacagugccguacaaaccacagugugcugc</td><td>gi|307603372|gb|CM00100</td></tr>
<tr><td>gi|307603368|gb|CM001008.1|_14094</td><td>2.6</td><td>0.81 ± 0.02</td><td></td><td>STAR</td><td>20618</td><td>20618</td><td>0</td><td>0</td><td>yes</td><td>mmu-miR-1249-5p</td><td></td><td></td><td>blast</td><td>acgcccuucccccccuucuuca</td><td>gaggagggaggggaugggccaaguuc</td><td>gaggagggagggggaugggccaaguucccuaugcccuggaacggcccuuccccccccuucuuca</td><td>gi|307603368|gb|CM00100</td></tr>
<tr><td>gi|307603376|gb|CM001000.1|_41834</td><td>2.6</td><td>0.81 ± 0.02</td><td></td><td>STAR</td><td>160933</td><td>160684</td><td>0</td><td>249</td><td>yes</td><td>mmu-miR-344-5p</td><td></td><td></td><td>blast</td><td>ugaucuagccaaagccugacugu</td><td>ucaggcuccuggcuagauucca</td><td>ucaggcuccuggcuagauuccagguaccaacugguaccugaucuagccaaagccugacugu</td><td>gi|307603376|gb|CM00100</td></tr>
<tr><td>gi|307603376|gb|CM001000.1|_40066</td><td>2.6</td><td>0.81 ± 0.02</td><td></td><td>TRUE</td><td>3419</td><td>3419</td><td>0</td><td>0</td><td>yes</td><td>mmu-miR-211-5p</td><td></td><td></td><td>blast</td><td>uucccuuugucauccuuugccu</td><td>gcaaggacagcaaagggggc</td><td>uucccuuugucauccuuugccuaggccucugagugaggcaaggacagcaaagggggc</td><td>gi|307603376|gb|CM00100</td></tr>
<tr><td>gi|307603381|gb|CM000995.1|_26052</td><td>2.5</td><td>0.81 ± 0.02</td><td></td><td>TRUE</td><td>89</td><td>89</td><td>0</td><td>0</td><td>yes</td><td>mmu-miR-5622-5p</td><td></td><td></td><td>blast</td><td>uucaccacacccagcuuaaaga</td><td>uuagcugguaguggguggugc</td><td>uucaccacacccagcuuaaagagaaguuuaaaaaauaauucuuagcugguaguggguggugc</td><td>gi|307603381|gb|CM00099</td></tr>
<tr><td>gi|307603382|gb|CM000994.1|_21449</td><td>2.5</td><td>0.81 ± 0.02</td><td></td><td>STAR</td><td>3925</td><td>3925</td><td>0</td><td>0</td><td>yes</td><td>mmu-miR-133b-5p</td><td></td><td></td><td>blast</td><td>uuugguccccuucaaccagcua</td><td>gcuggucaaacggaaccaaguc</td><td>gcuggucaaacggaaccaagucgucuucuugagagguuuugguccccuucaaccagcua</td><td>gi|307603382|gb|CM00099</td></tr>
<tr><td>gi|307603376|gb|CM001000.1|_39751</td><td>2.5</td><td>0.81 ± 0.02</td><td></td><td>STAR</td><td>5056</td><td>5056</td><td>0</td><td>0</td><td>yes</td><td>mmu-miR-1964-5p</td><td></td><td></td><td>blast</td><td>ccgacuucugggcuccggcuuu</td><td>agcuggagcacaaaagccggug</td><td>agcuggagcacaaaagccggugccggugugaugccuacaccgacuucugggcuccggcuuu</td><td>gi|307603376|gb|CM00100</td></tr>
<tr><td>gi|307603368|gb|CM001008.1|_13364</td><td>2.5</td><td>0.81 ± 0.02</td><td></td><td>TRUE</td><td>182</td><td>157</td><td>0</td><td>25</td><td>yes</td><td>mmu-miR-1941-5p</td><td></td><td></td><td>blast</td><td>agggagaugcugguacagagg</td><td>caucuuagcaguaucucccauc</td><td>agggagaugcugguacagaggcuuggggguuuugagggcaucuuagcaguaucucccauc</td><td>gi|307603368|gb|CM00100</td></tr>
<tr><td>gi|307603375|gb|CM001001.1|_44081</td><td>2.5</td><td>0.81 ± 0.02</td><td></td><td>TRUE</td><td>2781</td><td>2447</td><td>0</td><td>334</td><td>yes</td><td>mmu-miR-1968-5p</td><td></td><td></td><td>blast</td><td>ugcagcuguuaaggaugguggacu</td><td>accacccucugcagcuguuaagg</td><td>ugcagcuguuaaggaugguggacuauuguuagggccaccacccucugcagcuguuaagg</td><td>gi|307603375|gb|CM00100</td></tr>
<tr><td>gi|307603365|gb|CM001011.1|_19334</td><td>2.5</td><td>0.81 ± 0.02</td><td></td><td>STAR</td><td>751</td><td>751</td><td>0</td><td>0</td><td>yes</td><td>mmu-miR-6979-5p</td><td></td><td></td><td>blast</td><td>uugugucugucuggcucccagu</td><td>ugggggggaggcgcagagacugagc</td><td>ugggggggaggcgcagagacugagcugcucagugcgcgcuugugucugucuggcucccagu</td><td>gi|307603365|gb|CM00101</td></tr>
<tr><td>gi|307603376|gb|CM001000.1|_39478</td><td>2.5</td><td>0.81 ± 0.02</td><td></td><td>TRUE</td><td>218</td><td>218</td><td>0</td><td>0</td><td>yes</td><td>mmu-miR-5620-5p</td><td></td><td></td><td>blast</td><td>cacgaggcaggggcuuugacu</td><td>ucaucccccugccucacaca</td><td>cacgaggcaggggcuuugacugugacaaaaucacucacagucauccccccugccucacaca</td><td>gi|307603376|gb|CM00100</td></tr>
<tr><td>gi|307603376|gb|CM001000.1|_40359</td><td>2.5</td><td>0.81 ± 0.02</td><td></td><td>STAR</td><td>42908</td><td>42908</td><td>0</td><td>0</td><td>yes</td><td>mmu-miR-326-5p</td><td></td><td></td><td>blast</td><td>ccucuggcccuuccuccagu</td><td>ugggggcagggccuuugugaaggcg</td><td>ugggggcagggccuuugugaaggcggguuaugcucagaucgccucuggcccuuccuccagu</td><td>gi|307603376|gb|CM00100</td></tr>
<tr><td>gi|307603382|gb|CM000994.1|_23050</td><td>2.5</td><td>0.81 ± 0.02</td><td></td><td>TRUE</td><td>99</td><td>99</td><td>0</td><td>0</td><td>yes</td><td>mmu-miR-5103</td><td></td><td></td><td>blast</td><td>ucauccgggauccccugaggc</td><td>cuuuggggacccuaggaucugggc</td><td>cuuuggggacccuaggaucugggcauaacauuuggggggcucauccgggauccccugaggc</td><td>gi|307603382|gb|CM00099</td></tr>
<tr><td>gi|307603379|gb|CM000997.1|_32499</td><td>2.5</td><td>0.81 ± 0.02</td><td></td><td>TRUE</td><td>710</td><td>710</td><td>0</td><td>0</td><td>yes</td><td>mmu-miR-5123</td><td></td><td></td><td>blast</td><td>guguagauccauaugccauggug</td><td>ccaugguguguagauccauaugc</td><td>ccaugguguguagauccauaugccaugguguguagacccauaugccaugguguguagauccauaugccauggug</td><td>gi|307603379|gb|CM00099</td></tr>
<tr><td>gi|307603376|gb|CM001000.1|_39399</td><td>2.5</td><td>0.81 ± 0.02</td><td></td><td>STAR</td><td>204</td><td>204</td><td>0</td><td>0</td><td>yes</td><td>mmu-miR-3572-5p</td><td></td><td></td><td>blast</td><td>uacacuuguccuucuuuccccagg</td><td>uggggaacagggcaaggugga</td><td>uggggaacagggcaagguggacaguagcugacagccuguuuacacuuguccuucuuuccccagg</td><td>gi|307603376|gb|CM00100</td></tr>
<tr><td>gi|307603367|gb|CM001009.1|_15414</td><td>2.5</td><td>0.81 ± 0.02</td><td></td><td>TRUE</td><td>542</td><td>542</td><td>0</td><td>0</td><td>yes</td><td>mmu-miR-1945</td><td></td><td></td><td>blast</td><td>ucuucgcggguacugucggacu</td><td>ucccgucagcccccgagaaaacc</td><td>ucccgucagcccccgagaaaaccgcacgggucuucgcggguacugucggacu</td><td>gi|307603367|gb|CM00100</td></tr>
<tr><td>gi|307603379|gb|CM000997.1|_32500</td><td>2.5</td><td>0.81 ± 0.02</td><td></td><td>TRUE</td><td>710</td><td>710</td><td>0</td><td>0</td><td>yes</td><td>mmu-miR-5123</td><td></td><td></td><td>blast</td><td>guguagauccauaugccauggug</td><td>ccaugguguguagacucauaugc</td><td>ccaugguguguagacucauaugccaugguguguagacccauaugccaugguguguagauccauaugccauggug</td><td>gi|307603379|gb|CM00099</td></tr>
<tr><td>gi|307603368|gb|CM001008.1|_13402</td><td>2.5</td><td>0.81 ± 0.02</td><td></td><td>TRUE</td><td>133</td><td>133</td><td>0</td><td>0</td><td>yes</td><td>mmu-miR-196a-5p</td><td></td><td></td><td>blast</td><td>uagguaguuucauguuguuggg</td><td>cggcaacaagaaacugccugag</td><td>uagguaguuucauguuguugggauugaguuuugaacucggcaacaagaaacugccugag</td><td>gi|307603368|gb|CM00100</td></tr>
<tr><td>gi|307603379|gb|CM000997.1|_32137</td><td>2.4</td><td>0.81 ± 0.02</td><td></td><td>STAR</td><td>78</td><td>78</td><td>0</td><td>0</td><td>yes</td><td>mmu-miR-5616-5p</td><td></td><td></td><td>blast</td><td>acuugugaugaggugagacaga</td><td>cguuuccucucaucacaaguug</td><td>cguuuccucucaucacaaguugagaauacacagacagaucaacuugugaugaggugagacaga</td><td>gi|307603379|gb|CM00099</td></tr>
<tr><td>gi|307603381|gb|CM000995.1|_24552</td><td>2.4</td><td>0.81 ± 0.02</td><td></td><td>TRUE</td><td>1630</td><td>1630</td><td>0</td><td>0</td><td>yes</td><td>mmu-miR-669d-5p</td><td></td><td></td><td>blast</td><td>acuugugugugcauguauaugu</td><td>gcaugcaugcacacacacaaau</td><td>acuugugugugcauguauauguguguauaugaauauacauauacauacacccauauacacgcaugcaugcacacacacaaau</td><td>gi|307603381|gb|CM00099</td></tr>
<tr><td>gi|307603377|gb|CM000999.1|_37869</td><td>2.4</td><td>0.81 ± 0.02</td><td></td><td>STAR</td><td>285</td><td>285</td><td>0</td><td>0</td><td>yes</td><td>mmu-miR-3098-5p</td><td></td><td></td><td>blast</td><td>uucugcugccugccuuuagg</td><td>uaacagcaggaguaggagc</td><td>uaacagcaggaguaggagcucugauuauccccugcuucugcugccugccuuuagg</td><td>gi|307603377|gb|CM00099</td></tr>
<tr><td>gi|307603372|gb|CM001004.1|_3488</td><td>2.4</td><td>0.81 ± 0.02</td><td></td><td>TRUE</td><td>1810</td><td>1810</td><td>0</td><td>0</td><td>yes</td><td>mmu-miR-193a-5p</td><td></td><td></td><td>blast</td><td>ugggucuuugcgggcaagauga</td><td>aacuggccuacaaagucccagu</td><td>ugggucuuugcgggcaagaugagagugucaguucaacuggccuacaaagucccagu</td><td>gi|307603372|gb|CM00100</td></tr>
<tr><td>gi|307603371|gb|CM001005.1|_7144</td><td>2.4</td><td>0.81 ± 0.02</td><td></td><td>STAR</td><td>41558</td><td>23377</td><td>65</td><td>18116</td><td>yes</td><td>mmu-miR-345-5p</td><td></td><td></td><td>blast</td><td>cccugaacuaggggucuggaga</td><td>ugaccccuaguccagugcuugu</td><td>ugaccccuaguccagugcuugugguggcuacuggcccugaacuaggggucuggaga</td><td>gi|307603371|gb|CM00100</td></tr>
<tr><td>gi|307603371|gb|CM001005.1|_7172</td><td>2.4</td><td>0.81 ± 0.02</td><td></td><td>TRUE</td><td>311524</td><td>311524</td><td>0</td><td>0</td><td>yes</td><td>mmu-miR-431-5p</td><td></td><td></td><td>blast</td><td>ugucuugcaggccgucaugca</td><td>cacugacgguaacguugcaggucguc</td><td>ugucuugcaggccgucaugcaggccacacugacgguaacguugcaggucguc</td><td>gi|307603371|gb|CM00100</td></tr>
</table>

gi|307603373|gb|CM001003.1|_2077	2.4	0.81 ± 0.02		TRUE	132	132	0	0	yes	mmu-miR-7211-5p			blast	ucuuucccucugccacuccaccg	guggagugacuguagggaggaug	ucuuucccucugccacuccaccgcugggagcuugugucuguggagugacuguagggaggaug	gi|307603373|gb|CM00100
gi|307603363|gb|CM001013.1|_49258	2.4	0.81 ± 0.02		STAR	162	162	0	0	yes	mmu-miR-871-5p			blast	ugacuggcaccauucuggauaau	uauucagauuagugccagucaug	uauucagauuagugccagucaugugaaauacauaugacuggcaccauucuggauaau	gi|307603363|gb|CM00101
gi|307603376|gb|CM001000.1|_40919	2.4	0.81 ± 0.02		STAR	133	133	0	0	yes	mmu-miR-3105-5p			blast	acugcuuaugagcuugcacucc	agagcaagcccguaagcagcgu	agagcaagcccguaagcagcguaaguuacugaggaacacugcuuaugagcuugcacucc	gi|307603376|gb|CM00100
gi|307603368|gb|CM001008.1|_12877	2.4	0.81 ± 0.02		STAR	35	35	0	0	yes	mmu-miR-6953-5p			blast	cugacccagccucuaugucc	ggcaggggcagggauucaagug	ggcaggggcagggauucaagugcccucaggggacacugacccagccucuaugucc	gi|307603368|gb|CM00100
gi|307603365|gb|CM001011.1|_18828	2.4	0.81 ± 0.02		TRUE	1750	1750	0	0	yes	mmu-miR-122-5p			blast	uggagugugacaaugguguuug	aacgccauuaucacacuaaaua	uggagugugacaaugguguuugugucaaaccaucaaacgccauuaucacacuaaaua	gi|307603365|gb|CM00101
gi|307603371|gb|CM001005.1|_8268	2.4	0.81 ± 0.02		TRUE	165	165	0	0	yes	mmu-miR-1936			blast	uaacugaccugcugugaacuggc	cagucacacagaggacuuuagu	cagucacacagaggacuuuagucugagaaauugaaacuaacugaccugcugugaacuggc	gi|307603371|gb|CM00100
gi|307603376|gb|CM001000.1|_39677	2.4	0.81 ± 0.02		TRUE	2558	2558	0	0	yes	mmu-miR-1191a			blast	cagucuuacuauguagcccuag	agggcuacauagugagaaccugcc	agggcuacauagugagaaccugccucaaaagcaaaacagcagucuuacuauguagcccuag	gi|307603376|gb|CM00100
gi|307603363|gb|CM001013.1|_49182	2.4	0.81 ± 0.02		STAR	26662	26662	0	0	yes	mmu-miR-19b-2-5p			blast	ugugcaaauccaugcaaaacuga	aguuuugcagauuugcaguucagc	aguuuugcagauuugcaguucagcguauaugugaauauauggcugugcaaauccaugcaaaacuga	gi|307603363|gb|CM00101
gi|307603382|gb|CM000994.1|_22786	2.4	0.81 ± 0.02		TRUE	36379	36379	0	0	yes	mmu-miR-194-5p			blast	uguaacagcaacuccaugugga	caguggagcugcuguuacuuc	uguaacagcaacuccauguggacugugcucggauuccaguggagcugcuguuacuuc	gi|307603382|gb|CM00099
gi|307603373|gb|CM001003.1|_1322	2.4	0.81 ± 0.02		TRUE	106	70	36	0	yes	mmu-miR-466i-5p			blast	ugugugugugugugugugugu	auauauauacauacauauaua	ugugugugugugugugugugugcgugcgcguguauguauguauauauauacauacauauaua	gi|307603373|gb|CM00100
gi|307603375|gb|CM001001.1|_44662	2.4	0.81 ± 0.02		TRUE	16806	16806	0	0	yes	mmu-miR-3074-5p			blast	guuccugcugaacugagccagu	uugcucacggcagacauucugcag	uugcucacggcagacauucugcaggguugccagcuccuagggggcuggacuccuguuccugcugaacugagccagu	gi|307603375|gb|CM00100
gi|307603376|gb|CM001000.1|_41836	2.4	0.81 ± 0.02		STAR	370	370	0	0	yes	mmu-miR-344g-5p			blast	uagccaggggcuugacugcaag	ugccagucaggcuccuggcagga	ugccagucaggcuccuggcaggaguccugcucucagcugguuccaggcucuagccaggggcuugacugcaag	gi|307603376|gb|CM00100
gi|307603376|gb|CM001000.1|_42695	2.4	0.81 ± 0.02		TRUE	31	31	0	0	yes	mmu-miR-202-5p			blast	uuccuaugcauauacuucuuu	agagguauagcgcaugggaaga	uuccuaugcauauacuucuuuguggaucuggucuaaagagguauagcgcaugggaaga	gi|307603376|gb|CM00100
gi|307603380|gb|CM000996.1|_28670	2.4	0.81 ± 0.02		TRUE	36	36	0	0	yes	mmu-miR-8120			blast	ugugccuugcaacuuccgagcc	ccggcuaaggagcagggcacccc	ugugccuugcaacuuccgagccggaaacuuccuggccggcuaaggagcagggcacccc	gi|307603380|gb|CM00099
gi|307603373|gb|CM001003.1|_1225	2.4	0.81 ± 0.02		TRUE	95	95	0	0	yes	mmu-miR-8105			blast	ggcgacuggcgagacccgaag	ucggggcucgccagccgacuc	ucggggcucgccagccgacucccuaaagcggaggcgacuggcgagacccgaag	gi|307603373|gb|CM00100
gi|307603379|gb|CM000997.1|_32158	2.4	0.81 ± 0.02		TRUE	3497	3497	0	0	yes	mmu-miR-34a-5p			blast	uggcagugucuuagcugguuguu	caaucagcaaguauacugcccua	uggcagugucuuagcugguuguugugaguauuagcuaaggaagcaaucagcaaguauacugcccua	gi|307603379|gb|CM00099
gi|307603368|gb|CM001008.1|_13000	2.3	0.81 ± 0.02		TRUE	661	661	0	0	yes	mmu-miR-5113			blast	gacagaggaggagagagauccugu	agggucacucccuccucucugcu	gacagaggaggagagagauccuguuuaacucacugcaacaccaggucacucccuccucucugcu	gi|307603368|gb|CM00100
gi|307603372|gb|CM001004.1|_2758	2.3	0.81 ± 0.02		TRUE	903268	903198	70	0	yes	mmu-miR-218-5p			blast	uugugcuugaucuaaccaugug	caugguucugucaagcaccgcg	uugugcuugaucuaaccaugugguggaacgaugggaaacggaacaugguucugucaagcaccgcg	gi|307603372|gb|CM00100
gi|307603380|gb|CM000996.1|_29377	2.3	0.81 ± 0.02		STAR	933	933	0	0	yes	mmu-miR-302d-5p			blast	uaagugcuuccauguuugagugu	acuuuaacauggaggcacuugcu	acuuuaacauggaggcacuugcugugcauuuaaaaaauaagugcuuccauguuugagugu	gi|307603380|gb|CM00099
gi|307603364|gb|CM001012.1|_20525	2.3	0.81 ± 0.02		STAR	63	63	0	0	yes	mmu-miR-5623-5p			blast	ucugguucuggcuuucagcugu	agcuggcaagucagaaugcaggug	agcuggcaagucagaaugcaggugugaauggacaugggggucugguucuggcuuucagcugu	gi|307603364|gb|CM00101
gi|307603372|gb|CM001004.1|_4716	2.3	0.81 ± 0.02		TRUE	41743	41743	0	0	yes	mmu-miR-146a-5p			blast	ugagaacugaauuccauggguu	ccugggaggaaggccucauc	ccugggaggaaggccucaucuggagagucugugguauccccagcucugagaacugaauuccauggguu	gi|307603372|gb|CM00100
gi|307603371|gb|CM001005.1|_8322	2.3	0.81 ± 0.02		STAR	409	409	0	0	yes	mmu-miR-3544-5p			blast	acuccugcaugacgccguu	aggcaucauauaggagcug	aggcaucauauaggagcugaauggcugugcaaucaacuccugcaugacgccguu	gi|307603371|gb|CM00100
gi|307603365|gb|CM001011.1|_19092	2.3	0.81 ± 0.02		STAR	91991	91991	0	0	yes	mmu-miR-1a-2-5p			blast	uggaauguaaagaaguauguau	acauacuucuuuauguacccaua	acauacuucuuuauguacccauaugaacauucagugcuauggaauguaaagaaguauguau	gi|307603365|gb|CM00101
gi|307603381|gb|CM000995.1|_24628	2.3	0.81 ± 0.02		TRUE	14924	14924	0	0	yes	mmu-miR-669c-5p			blast	auaguuguguguggauguguguau	acacacacacacacaaguaaac	auaguuguguguggauguguguauuuugcauauaaauaacauacacacacacacacaaguaaac	gi|307603381|gb|CM00099
gi|307603371|gb|CM001005.1|_7167	2.3	0.81 ± 0.02		STAR	18993	18993	0	0	yes	mmu-miR-665-5p			blast	accaggaggcugaggucccuuaca	ugaggggccucugccucuauccaggauua	ugaggggccucugccucuauccaggauuauguuuuuaugaccaggaggcugaggucccuuaca	gi|307603371|gb|CM00100
gi|307603381|gb|CM000995.1|_24545	2.3	0.81 ± 0.02		TRUE	857	576	0	281	yes	mmu-miR-669f-5p			blast	aguugugugugcaugugcaugugu	auauacauacacacacacguau	aguugugugugcaugugcauguguguauaugaauauacauauacauacacacacacguau	gi|307603381|gb|CM00099
gi|307603381|gb|CM000995.1|_24556	2.2	0.81 ± 0.02		TRUE	1630	1630	0	0	yes	mmu-miR-669d-5p			blast	acuugugugugcauguauaugu	auauacauacacacccauauac	acuugugugugcauguauaugugucuauaugaauauacauauacauacacacccauauac	gi|307603381|gb|CM00099
gi|307603364|gb|CM001012.1|_21052	2.2	0.81 ± 0.02		STAR	56279	56253	0	26	yes	mmu-miR-107-5p			blast	agcagcauuguacagggcuauca	agcuucuuuacaguguugccuugu	agcuucuuuacaguguugccuuguggcauggaguucaagcagcauuguacagggcuauca	gi|307603364|gb|CM00101
gi|307603369|gb|CM001007.1|_10866	2.2	0.81 ± 0.02		TRUE	95	95	0	0	yes	mmu-miR-3075-5p			blast	ugucugggagcagccaagga	cuuggccuuccuagguguc	ugucugggagcagccaaggacaaguuaccucuugucuucuauccuuggccuuccuagguguc	gi|307603369|gb|CM00100
gi|307603371|gb|CM001005.1|_7255	2.2	0.81 ± 0.02		STAR	17505	16086	0	1419	yes	mmu-miR-758-5p			blast	uuugugaccugguccacuaaccc	ugguugaccagagagcacacg	ugguugaccagagagcacacgcuauauuugugccguuugugaccugguccacuaaccc	gi|307603371|gb|CM00100
gi|307603378|gb|CM000998.1|_36353	2.2	0.81 ± 0.02		TRUE	73	73	0	0	yes	mmu-miR-7647-5p			blast	aacuaccaccuccuggccuuuu	aaguccaggaggugguaguaca	aacuaccaccuccuggccuuuuuuuuuaaaaagcaccaaaaguccaggaggugguaguaca	gi|307603378|gb|CM00099
gi|307603378|gb|CM000998.1|_34425	2.2	0.81 ± 0.02		STAR	66	66	0	0	yes	mmu-miR-7025-5p			blast	cagcacaacuccagcucacca	gugagcugaagcugguggcuccc	gugagcugaagcugguggcucccagugaguuucucaguaggcagcacaacuccagcucacca	gi|307603378|gb|CM00099
gi|307603376|gb|CM001000.1|_41828	2.2	0.81 ± 0.02		STAR	590	590	0	0	yes	mmu-miR-344b-5p			blast	cauuuagccaaagccugacugu	agucaggcuccuggcuaaaguuc	agucaggcuccuggcuaaaguucagcuucagcugcauuuagccaaagccugacugu	gi|307603376|gb|CM00100
gi|307603364|gb|CM001012.1|_21134	2.2	0.81 ± 0.02		STAR	1974	1974	0	0	yes	mmu-miR-3085-5p			blast	ucuggcugcuauggcccccucc	aaggugccauuccgagggccaagagu	aaggugccauuccgagggccaagagucugaauaugagucacucuggcugcuauggcccccucc	gi|307603364|gb|CM00101
gi|307603376|gb|CM001000.1|_41168	2.2	0.81 ± 0.02		STAR	153	153	0	0	yes	mmu-miR-7046-5p			blast	ugaaccacccauccccccuacagg	uguagggugaggcugggagccagg	uguagggugaggcugggagccaggcgaaagagcacagccugaaccacccaucccccuacagg	gi|307603376|gb|CM00100
gi|307603376|gb|CM001000.1|_41822	2.2	0.81 ± 0.02		STAR	1314	1306	0	8	yes	mmu-miR-344e-5p			blast	gauauaaccaaagccugacuau	caggcuucuggcuauauuccagga	caggcuucuggcuauauuccaggacuuaccugguccuggauauaaccaaagccugacuau	gi|307603376|gb|CM00100
gi|307603378|gb|CM000998.1|_34605	2.2	0.81 ± 0.02		STAR	52	52	0	0	yes	mmu-miR-5619-5p			blast	acgaaaagcucauuggugauu	ucacuaauggcauuuuuguug	ucacuaauggcauuuuuguugguuguuaccagaacaacgaaaagcucauuggugauu	gi|307603378|gb|CM00099
gi|307603372|gb|CM001004.1|_5542	2.2	0.81 ± 0.02		STAR	72	72	0	0	yes	mmu-miR-3063-5p			blast	ugaggaauccugaucucucgcc	cuggagaucagguuugcacacu	cuggagaucagguuugcacacugucuugagaacaauguuccagugaggaauccugaucucucgcc	gi|307603372|gb|CM00100
gi|307603365|gb|CM001011.1|_18339	2.2	0.81 ± 0.02		TRUE	375	375	0	0	yes	mmu-miR-1948-5p			blast	auaugagauucugccuaaau	uuaggcagaauucucaccuua	uuaggcagaauucucaccuuacuauaugagauucugccuaaau	gi|307603365|gb|CM00101
gi|307603372|gb|CM001004.1|_6196	2.2	0.81 ± 0.02		STAR	46	46	0	0	yes	mmu-miR-7236-5p			blast	uuggaccgcacagauuuagacc	ccuaaaacugugugguccaaugcc	ccuaaaacugugugguccaaugcccauuuaacuaccagguuggaccgcacagauuuagacc	gi|307603372|gb|CM00100
gi|307603376|gb|CM001000.1|_41830	2.2	0.81 ± 0.02		STAR	962	918	0	44	yes	mmu-miR-344c-5p			blast	ugaucuagucaaagccugacagu	ucaggcugcuggcuagaguacagc	ucaggcugcuggcuagaguacagcuaucagcugguaccugaucuagucaaagccugacagu	gi|307603376|gb|CM00100
gi|307603372|gb|CM001004.1|_4715	2.2	0.81 ± 0.02		TRUE	41743	41743	0	0	yes	mmu-miR-146a-5p			blast	ugagaacugaauuccauggguu	ccugugaaauucaguucuucag	ugagaacugaauuccauggguuauaucaaugucagaccugugaaauucaguucuucag	gi|307603372|gb|CM00100
gi|307603381|gb|CM000995.1|_24565	2.1	0.81 ± 0.02		STAR	9663	9595	0	68	yes	mmu-miR-466b-5p			blast	auacauacacgcacacauaaga	ugaugugugugguacauguaca	ugaugugugugguacauguacauaugugaauaugauauacauauacauacacgcacacauaaga	gi|307603381|gb|CM00099
gi|307603381|gb|CM000995.1|_24560	2.1	0.81 ± 0.02		TRUE	232	232	0	0	yes	mmu-miR-466b-5p			blast	ugaugugugugguacauguaca	cacacauaagacacauaugagc	ugaugugugugguacauguacaugugugaauaugauauacauacaugcacacauaagacacauaugagc	gi|307603381|gb|CM00099
gi|307603381|gb|CM000995.1|_24642	2.1	0.81 ± 0.02		TRUE	4857	2738	0	2119	yes	mmu-miR-669o-5p			blast	uaguugugugugcauguuuaugu	acauaacauacacacacacguau	uaguugugugugcauguuuaugugugguauuugaauauacauaacauacacacacacguau	gi|307603381|gb|CM00099
gi|307603379|gb|CM000997.1|_33822	2.1	0.81 ± 0.02		STAR	41389	41389	0	0	yes	mmu-miR-429-5p			blast	uaauacugucugguaaugccgu	gucuuaccagacaugguuaga	gucuuaccagacaugguuagaucuggaugcaucugucuaauacugucugguaaugccgu	gi|307603379|gb|CM00099
gi|307603381|gb|CM000995.1|_24581	2.1	0.81 ± 0.02		STAR	9663	9595	0	68	yes	mmu-miR-466o-5p			blast	auacauacacgcacacauaaga	ugaugugugugguacauguaca	ugaugugugugguacauguacaugugugaauaugauauacauauacauacacgcacacauaaga	gi|307603381|gb|CM00099
gi|307603372|gb|CM001004.1|_5035	2.1	0.81 ± 0.02		TRUE	1650	1650	0	0	yes	mmu-miR-3062-5p			blast	ggagaauguaguguuaccguga	acggggacacacuuucuccuu	ggagaauguaguguuaccgugagccugucagcaaauaccacucacggggacacacuuucuccuu	gi|307603372|gb|CM00100
gi|307603370|gb|CM001006.1|_9474	2.1	0.81 ± 0.02		TRUE	231	208	0	23	yes	mmu-miR-449c-5p			blast	aggcagugcauugcuagcuggcu	aguugcuagcugcacuacccuc	aggcagugcauugcuagcuggcuguuagaacuuuaucccaacaguugcuagcugcacuacccuc	gi|307603370|gb|CM00100
gi|307603381|gb|CM000995.1|_24575	2.1	0.81 ± 0.02		STAR	9636	9595	0	41	yes	mmu-miR-466c-5p			blast	auacauacacgcacacauaaga	ugaugugugugugcauguaca	ugaugugugugugcauguacauaugugaauaugauauacauauacauacacgcacacauaaga	gi|307603381|gb|CM00099
gi|307603364|gb|CM001012.1|_20090	2.1	0.81 ± 0.02		TRUE	259171	258041	0	1130	yes	mmu-miR-204-5p			blast	uucccuuugucauccuaugccuga	gcugggaaggcaaagggacgu	uucccuuugucauccuaugccugagaauauaugaaggaggcugggaaggcaaagggacgu	gi|307603364|gb|CM00101
gi|307603372|gb|CM001004.1|_2960	2.1	0.81 ± 0.02		TRUE	273	202	0	71	yes	mmu-miR-7652-5p			blast	uaagggcacagggauuucagg	ucuggcuuuccuguggcuucug	uaagggcacagggauuucaggcauggugccaccaugucuggcuuuccuguggcuucug	gi|307603372|gb|CM00100
gi|307603381|gb|CM000995.1|_24567	2.1	0.81 ± 0.02		STAR	9663	9595	0	68	yes	mmu-miR-466o-5p			blast	auacauacacgcacacauaaga	ugaugugugugguacauguaca	ugaugugugugguacauguacauaugugaauaugauauacauauacauacacgcacacauaaga	gi|307603381|gb|CM00099
gi|307603379|gb|CM000997.1|_31305	2.0	0.81 ± 0.02		STAR	60204	58216	0	1988	yes	mmu-miR-455-5p			blast	gcaguccacgggcauauacacu	uaugugccuuuggacuacaucgu	uaugugccuuuggacuacaucgugaacgcagcaccaugcaguccacgggcauauacacu	gi|307603379|gb|CM00099
gi|307603374|gb|CM001002.1|_46774	2.0	0.81 ± 0.02		STAR	457	438	19	0	yes	mmu-miR-7081-5p			blast	uuccccugguauccucucaca	agaggagggugcucggccggggauua	agaggagggugcucggccggggauuauaaaucugugguaaaguaguauuccccugguauccucucaca	gi|307603374|gb|CM00100
gi|307603363|gb|CM001013.1|_49496	2.0	0.81 ± 0.02		STAR	36045	36045	0	0	yes	mmu-miR-421-5p			blast	aucaacagacauuaauugggcgc	ccucauuaaauguuuguugaauga	ccucauuaaauguuuguugaaugaaaaaaugaaucaucaacagacauuaauugggcgc	gi|307603363|gb|CM00101
gi|307603380|gb|CM000996.1|_28890	2.0	0.81 ± 0.02		TRUE	3524	3524	0	0	yes	mmu-miR-190b-5p			blast	ugauauguuugauauuggguug	acugaaugucaagcauacucuca	ugauauguuugauauuggguuguuaaauuaugaaccaacugaaugucaagcauacucuca	gi|307603380|gb|CM00099
gi|307603381|gb|CM000995.1|_24550	2.0	0.81 ± 0.02		TRUE	168	168	0	0	yes	mmu-miR-669b-5p			blast	aguuuugugugcaugugcaugug	cauauacauacacacaaacauau	aguuuugugugcaugugcaugugugucuauuaauguacauauacauacacacaaacauau	gi|307603381|gb|CM00099
gi|307603371|gb|CM001005.1|_7177	2.0	0.81 ± 0.02		STAR	929790	883382	0	46408	yes	mmu-miR-127-5p			blast	ucggauccgucugagcuuggcu	gaagcucagagggcucugauu	gaagcucagagggcucugauucagaaagaucaucggauccgucugagcuuggcu	gi|307603371|gb|CM00100
gi|307603376|gb|CM001000.1|_41146	2.0	0.81 ± 0.02		STAR	1074	1033	0	41	yes	mmu-miR-3470a			blast	aaaucuaccugccucugccu	uuguagaccaggcuggcc	uuguagaccaggcuggccucaaacucagaaaucuaccugccucugccu	gi|307603376|gb|CM00100
gi|307603367|gb|CM001009.1|_15130	2.0	0.81 ± 0.02		TRUE	10651	10651	0	0	yes	mmu-miR-155-5p			blast	uuaaugcuaauugugauagggguu	cuccuaccuguuagcauuaaca	uuaaugcuaauugugauaggggguuuuggccucugacugacuccuaccuguuagcauuaaca	gi|307603367|gb|CM00100
gi|307603375|gb|CM001001.1|_43710	2.0	0.81 ± 0.02		TRUE	99	61	0	38	yes	mmu-miR-3108-5p			blast	gucucuaaagcuagacguuccgg	cgucuaggucuagagucucaag	gucucuaaagcuagacguuccggagaauuuuuuuauuucggcgucuaggucuagagucucaag	gi|307603375|gb|CM00100
gi|307603371|gb|CM001005.1|_7159	2.0	0.81 ± 0.02		TRUE	54000	53984	16	0	yes	mmu-miR-673-5p			blast	cucacagcucugguccuugga	caugcaagagcugugggag	caugcaagagcugugggaggucuggcuuuggagccugaggggcucacagcucugguccuugga	gi|307603371|gb|CM00100
gi|307603375|gb|CM001001.1|_43426	2.0	0.81 ± 0.02		TRUE	767992	767992	0	0	yes	mmu-miR-24-3p			blast	uggcucaguucagcaggaacag	aacccugcagaaugucugccgug	uggcucaguucagcaggaacaggaguccagcccccuaggagcuggcaacccugcagaaugucugccgug	gi|307603375|gb|CM00100
gi|307603381|gb|CM000995.1|_24548	2.0	0.81 ± 0.02		TRUE	234	228	2	4	yes	mmu-miR-669e-5p			blast	ugucuugugugugcauguucauu	cacacucaugcacacacaca	ugucuugugugugcauguucauuuguguauaugaauaugaauauacacacuuacacacucaugcacacacacaca	gi|307603381|gb|CM00099
gi|307603376|gb|CM001000.1|_41816	2.0	0.81 ± 0.02		STAR	18031	18031	0	0	yes	mmu-miR-344d-2-5p			blast	gauauaaccacugccagacuga	agucugguugcuggcuauauucca	agucugguugcuggcuauauuccaggacuuaucugguccuggauauaaccacugccagacuga	gi|307603376|gb|CM00100
gi|307603380|gb|CM000996.1|_29375	2.0	0.81 ± 0.02		STAR	731	586	0	145	yes	mmu-miR-302a-5p			blast	aagugcuuccauguuuugguga	uaaacgugguuguacuugcuuu	uaaacgugguuguacuugcuuuagaccuaagaaaguaagugcuuccauguuuugguga	gi|307603380|gb|CM00099
gi|307603374|gb|CM001002.1|_46017	2.0	0.81 ± 0.02		STAR	32	32	0	0	yes	mmu-miR-3109-5p			blast	uagggccaucucauccagau	auggaugcgaugguucccaugc	auggaugcgaugguucccaugcuguucugaggcagagcuagggccaucucauccagau	gi|307603374|gb|CM00100
gi|307603377|gb|CM000999.1|_38206	1.9	0.28 ± 0.02		STAR	22918	22891	0	27	yes	mmu-miR-29a-5p			blast	uagcaccaucugaaaucgguua	ugauuucuuuugguguucagag	ugauuucuuuugguguucagagucaauagaauuuucuagcaccaucugaaaucgguua	gi|307603377|gb|CM00099
gi|307603376|gb|CM001000.1|_41818	1.9	0.28 ± 0.02		TRUE	18031	18031	0	0	yes	mmu-miR-344d-3p			blast	gauauaaccacugccagacuga	agucaggcugcuggugauucucca	agucaggcugcuggugauucuccaggacuuaucugguccuggauauaaccacugccagacuga	gi|307603376|gb|CM00100
gi|307603371|gb|CM001005.1|_7179	1.9	0.28 ± 0.02		STAR	1298646	1177038	0	121608	yes	mmu-miR-434-5p			blast	uuugaaccaucacucgacuccu	gcucgacucaugguuugaacca	gcucgacucaugguuugaaccauuacuuaauucgugguuugaaccaucacucgacuccu	gi|307603371|gb|CM00100
gi|307603372|gb|CM001004.1|_2763	1.9	0.28 ± 0.02		STAR	2646429	2646429	0	0	yes	mmu-miR-103-1-5p			blast	agcagcauuguacagggcuauga	ucggcuucuuuacagugcugccuug	ucggcuucuuuacagugcugccuuguugcauaggaucaagcagcauuguacagggcuauga	gi|307603372|gb|CM00100
gi|307603382|gb|CM000994.1|_22368	1.9	0.28 ± 0.02		TRUE	595	595	0	0	yes	mmu-miR-7667-5p			blast	gagccaucucucuagccccuga	ucuuucuagagaggcagcuuucuccc	gagccaucucucuagccccugaauuuuuuucuuucuagagaggcagcuuucuccc	gi|307603382|gb|CM00099
gi|307603366|gb|CM001010.1|_17448	1.9	0.28 ± 0.02		NA	2226	2226	0	0	yes	mmu-miR-3082-5p			blast	cacauggcacucaacucugcag	gcaugacaugagugcccuggug	gcaugacaugagugcccugguggacagagugugugugucuguggagacaggaguugcccacacauggcacucaacucugcag	gi|307603366|gb|CM00101
gi|307603363|gb|CM001013.1|_48792	1.9	0.28 ± 0.02		TRUE	423	423	0	0	yes	mmu-miR-3552			blast	aggcugcaggcccacuucccu	ggaguggggaaaguggcccua	aggcugcaggcccacuucccuuggcagauuuuuccauuucagggaguggggaaaguggcccua	gi|307603363|gb|CM00101
gi|307603373|gb|CM001003.1|_988	1.9	0.28 ± 0.02		TRUE	10357	10237	0	120	yes	mmu-miR-3059-5p			blast	uuuccucucugccccauaggu	ccucuagggaagagaagguuggg	uuuccucucugccccauaggguguagcucuaacuacccucuagggaagagaagguuggg	gi|307603373|gb|CM00100
gi|307603382|gb|CM000994.1|_22858	1.9	0.28 ± 0.02		TRUE	1711	772	0	939	yes	mmu-miR-29c-5p			blast	ugaccgauuucuccugguguuc	uagcaccauuugaaaucgguuau	ugaccgauuucuccugguguucagagucuguuuuugucuagcaccauuugaaaucgguuau	gi|307603382|gb|CM00099
gi|307603377|gb|CM000999.1|_38374	1.9	0.28 ± 0.02		TRUE	1366	1366	0	0	yes	mmu-miR-704			blast	agacaugugcucugcuccuagg	ugggagcuagaggauguggucagcugc	ugggagcuagaggauguggucagcugcaaggaucuuccucagacaugugcucugcuccuagg	gi|307603377|gb|CM00099
gi|307603378|gb|CM000998.1|_34310	1.9	0.28 ± 0.02		TRUE	857582	857520	32	30	yes	mmu-miR-218-5p			blast	uugugcuugaucuaaccaugug	aacaugguuccgucaagcac	uugugcuugaucuaaccaugugcuugcgagguaugagaaaaacaugguuccgucaagcac	gi|307603378|gb|CM00099
gi|307603372|gb|CM001004.1|_4262	1.8	0.28 ± 0.02		TRUE	652	520	0	132	yes	mmu-miR-6516-5p			blast	auuugcaguaacagguguggaca	auguaugauacugcaaacaggagc	auuugcaguaacagguguggacaucuagcagcaguuugguggucauguaugauacugcaaacaggagc	gi|307603372|gb|CM00100
gi|307603373|gb|CM001003.1|_119	1.8	0.28 ± 0.02		STAR	32	32	0	0	yes	mmu-miR-6905-5p			blast	uaaauaaccuuaucugcccgau	ugggcagguuggguugaaugau	ugggcagguuggguugaaugauuugaucaugucacuuaaauaaccuuaucugcccgau	gi|307603373|gb|CM00100
gi|307603371|gb|CM001005.1|_7290	1.8	0.28 ± 0.02		TRUE	21169	21169	0	0	yes	mmu-miR-539-5p			blast	ggagaaauuauccuugguguguu	cauacaaggauaauuucuuuuu	ggagaaauuauccuugguguguuggcucuuuuggaugaaucauacaaggauaauuucuuuuu	gi|307603371|gb|CM00100
gi|307603365|gb|CM001011.1|_18511	1.8	0.28 ± 0.02		TRUE	979	979	0	0	yes	mmu-miR-1949			blast	uauaccaggaugucagcauaguu	cugguuggcauucugggccuggu	cugguuggcauucugggccugguucaugccaccucuguguuuacuauaccaggaugucagcauaguu	gi|307603365|gb|CM00101
gi|307603378|gb|CM000998.1|_36511	1.8	0.28 ± 0.02		TRUE	55	55	0	0	yes	mmu-miR-7033-5p			blast	uccaggagucugaggggc	cuccauucucuugggca	uccaggagucugaggggcaggucgucuccagcaucuugggcuuggccuaaccugcccuccauucucuugggca	gi|307603378|gb|CM00099
gi|307603381|gb|CM000995.1|_26429	1.8	0.28 ± 0.02		STAR	91991	91991	0	0	yes	mmu-miR-1a-1-5p			blast	uggaauguaaagaaguauguau	acauacuucuuuauaugcccaua	acauacuucuuuauaugcccauaugaaccugcuaagcuauggaauguaaagaaguauguau	gi|307603381|gb|CM00099
gi|307603382|gb|CM000994.1|_22461	1.8	0.28 ± 0.02		TRUE	5580	5471	0	109	yes	mmu-miR-8114			blast	ucacccaucuccucuccgccucg	gagaaugggaguagagcagagu	gagaaugggaguagagcagagugucugaacagcacgcucacccaucuccucuccgccucg	gi|307603382|gb|CM00099
gi|307603376|gb|CM001000.1|_41824	1.8	0.28 ± 0.02		STAR	290	282	0	8	yes	mmu-miR-344h-5p			blast	gguauaaccaaagcccgacugu	caggcuucuggcuauauuccagga	caggcuucuggcuauauuccaggacauaccugguccugguauaaccaaagcccgacugu	gi|307603376|gb|CM00100
gi|307603382|gb|CM000994.1|_22788	1.7	0.28 ± 0.02		TRUE	32	32	0	0	yes	mmu-miR-215-5p			blast	augaccuaugauuugacagac	cugucauucuguaggccaaua	augaccuaugauuugacagaccgugcagcuguguaugucugcauucuguaggccaaua	gi|307603382|gb|CM00099
gi|307603367|gb|CM001009.1|_15222	1.7	0.28 ± 0.02		TRUE	221	221	0	0	yes	mmu-miR-802-5p			blast	ucaguaacaaagauucauccuug	acacggagagucuuugucacucagu	ucaguaacaaagauucauccuuggugucaaucaucaacacggagagucuuugucacucagu	gi|307603367|gb|CM00100
gi|307603377|gb|CM000999.1|_37639	1.7	0.28 ± 0.02		STAR	107	107	0	0	yes	mmu-miR-7661-5p			blast	uugacucccaguuucucucugc	agaaagaaaccuggaguuuaaa	agaaagaaaccuggaguuuaaaguagcucuuguaaucccuugacucccaguuucucucugc	gi|307603377|gb|CM00099
gi|307603371|gb|CM001005.1|_7243	1.7	0.28 ± 0.02		TRUE	637465	637050	0	415	yes	mmu-miR-379-5p			blast	ugguagacuauggaacguagg	uuccaugguuccugaagaga	uuccaugguuccugaagagaugguagacuauggaacguagg	gi|307603371|gb|CM00100
gi|307603382|gb|CM000994.1|_22855	1.7	0.28 ± 0.02		STAR	616	313	0	303	yes	mmu-miR-29b-2-5p			blast	cuagcaccauuugaaaucag	cugguuucacaugguggcuuagau	cugguuucacaugguggcuuagauuuuuccaucuuuguaucuagcaccauuugaaaucag	gi|307603382|gb|CM00099
gi|307603363|gb|CM001013.1|_49187	1.7	0.28 ± 0.02		TRUE	1251	1228	0	23	yes	mmu-miR-106a-5p			blast	caaagugcuaacagugcagguagc	uacugcagugccagcacuucu	caaagugcuaacagugcagguaguuuuugaguucuacugcagugccagcacuucu	gi|307603363|gb|CM00101
gi|307603377|gb|CM000999.1|_38061	1.7	0.28 ± 0.02		TRUE	1393	1393	0	0	yes	mmu-miR-653-5p			blast	uugaaacaaucucuacugaacc	uucacuggaguuuguuucagua	uugaaacaaucucuacugaaccaagcuccaaagcgaguucacuggaguuuguuucagua	gi|307603377|gb|CM00099
gi|307603374|gb|CM001002.1|_47626	1.7	0.28 ± 0.02		STAR	1144	1144	0	0	yes	mmu-miR-184-5p			blast	uggacggagaacugauaagggu	ccuuaucacuuuuccagccagc	ccuuaucacuuuuccagccagcuuugugacucuaaguguuggacggagaacugauaagggu	gi|307603374|gb|CM00100
gi|307603372|gb|CM001004.1|_2442	1.6	0.28 ± 0.02		TRUE	184	184	0	0	yes	mmu-miR-6918-5p			blast	ugcugaggacgggauuagguuc	gccugugcccuucucacagccuaca	ugcugaggacgggauuagguucucugaagaaugagccugugcccuucucacagccuaca	gi|307603372|gb|CM00100
gi|307603382|gb|CM000994.1|_22367	1.6	0.28 ± 0.02		TRUE	595	595	0	0	yes	mmu-miR-7667-5p			blast	gagccaucucucuagccccuga	aggugcugggaacugagcucug	aggugcugggaacugagcucugguccucugaacaggaggaucucaaccauucucaaccagggagccaucucucuagccccuga	gi|307603382|gb|CM00099
gi|307603367|gb|CM001009.1|_16027	1.6	0.28 ± 0.02		TRUE	49	49	0	0	yes	mmu-miR-6964-5p			blast	uuggggugugagacuggaaa	ucuugucuuccacucuaggu	uuggggugugagacuggaaacaucaccauuguucuuucuugucuuccacucuaggu	gi|307603367|gb|CM00100
gi|307603380|gb|CM000996.1|_29373	1.6	0.28 ± 0.02		STAR	143	69	0	74	yes	mmu-miR-302c-5p			blast	aagugcuuccauguuucagugg	uuuaacaugggguuaccugcug	uuuaacaugggguuaccugcuguguuaaacaaaagcaagugcuuccauguuucagugg	gi|307603380|gb|CM00099
gi|307603381|gb|CM000995.1|_24572	1.5	0.28 ± 0.02		STAR	56	55	0	1	yes	mmu-miR-669h-3p			blast	uuugcaugugugauaguugugu	auauacacacaugcacacac	uuugcaugugugauaguuguguggcauauucacuucucuauaugaauaugcauauacacacaugcacacac	gi|307603381|gb|CM00099
gi|307603365|gb|CM001011.1|_18332	1.4	0.28 ± 0.02		TRUE	114	95	0	19	yes	mmu-miR-1b-5p			blast	uacauacuucuuuacauucca	ugguacauaaagaaguaugug	uacauacuucuuuacauuccauagcacugaauguucauaugguacauaaagaaguaugug	gi|307603365|gb|CM00101
gi|307603375|gb|CM001001.1|_43797	1.4	0.28 ± 0.02		STAR	232	232	0	0	yes	mmu-miR-7077-5p			blast	ccuuccauggcuccuggcag	gccaaggucccauggcaggcu	gccaaggucccauggcaggcuauucccugacacaccuuccauggcuccuggcag	gi|307603375|gb|CM00100
gi|307603381|gb|CM000995.1|_25102	1.4	0.28 ± 0.02		TRUE	2133585	2132064	0	1521	yes	mmu-miR-181b-5p			blast	aacauucauugcugucggugggu	acucacugaucaaugaaugca	aacauucauugcugucgguggguuugaaugucaaccaacucacugaucaaugaaugca	gi|307603381|gb|CM00099
gi|307603381|gb|CM000995.1|_24908	1.2	0.28 ± 0.02		TRUE	174	140	0	34	yes	mmu-miR-6997-5p			blast	uaacaggcuggagaggugcaga	cucaaaccuuacccuccuguuucc	uaacaggcuggagaggugcagaacagagucucuggagucucucaaaccuuacccuccuguuucc	gi|307603381|gb|CM00099
gi|307603380|gb|CM000996.1|_29371	1.2	0.28 ± 0.02		STAR	899	899	0	0	yes	mmu-miR-302b-5p			blast	uaagugcuuccauguuuuagu	uuuaacaugggaaugcuuucugucu	uuuaacaugggaaugcuuucugucucaucgaagaguaagugcuuccauguuuuagu	gi|307603380|gb|CM00099
gi|307603379|gb|CM000997.1|_33444	1.1	0.28 ± 0.02		STAR	75	75	0	0	yes	mmu-miR-7227-5p			blast	ucugaaaagggucaucggcagaca	acucugaugacuuuuucauuga	acucugaugacuuuuucauugaaaaaauacuugcuauuucucugaaaagggucaucggcagaca	gi|307603379|gb|CM00099
gi|307603380|gb|CM000996.1|_29676	1.0	0.28 ± 0.02		STAR	75	75	0	0	yes	mmu-miR-7007-5p			blast	cccauccacguuucuucuguag	ucagaagaggcaguggaggag	ucagaagaggcaguggaggagagguacacagcacauucugaucccauccacguuucuucuguag	gi|307603380|gb|CM00099
gi|307603382|gb|CM000994.1|_22857	0.9	0.00 ± 0.00		TRUE	772	772	0	0	yes	mmu-miR-29c-5p			blast	ugaccgauuucuccugguguuc	acaccaccagucgguccau	acaccaccagucgguccaucucuuacacaggcugaccgauuucuccugguguuc	gi|307603382|gb|CM00099
gi|307603376|gb|CM001000.1|_42730	0.7	0.00 ± 0.00		TRUE	519	519	0	0	yes	mmu-miR-669f-5p			blast	aguugugugugcaugugcaugugu	acacacacacacacacacacacc	acacacacacacacacacacaccacagcauuaaaaaaacaaaacaacaaaaaacccaguugugugugcaugugcaugugu	gi|307603376|gb|CM00100
gi|307603382|gb|CM000994.1|_22377	0.6	0.00 ± 0.00		TRUE	7788497	7788478	19	0	yes	mmu-miR-181a-5p			blast	aacauucaacgcugucggugagu	ucccacagauacuguuua	ucccacagauacuguuuaaauucagcacaucucugccucacagguuguucagugaacauucaacgcugucggugagu	gi|307603382|gb|CM00099
gi|307603371|gb|CM001005.1|_6475	0	0.00 ± 0.00		TRUE	12834	12834	0	0	yes	mmu-miR-5099			blast	gcuuagaucgauguggugcuc	gaaauuacauugauuuaagaag	gaaauuacauugauuuaagaagagugguaaugggcuuagaucgauguggugcuc	gi|307603371|gb|CM00100
gi|307603371|gb|CM001005.1|_6753	0	0.00 ± 0.00		TRUE	81	81	0	0	yes	mmu-miR-5101			blast	uuuuguuuguuuugcugaugca	uaucaguuacaaacaaaaac	uaucaguuacaaacaaaaaccguuuuguaagacuuguuguuuuuguuuguuuugcugaugca	gi|307603371|gb|CM00100
gi|307603377|gb|CM000999.1|_37822	-0.7	0.00 ± 0.00		TRUE	122	122	0	0	yes	mmu-miR-7231-5p			blast	uuggggaacacuggggcauac	uugcuucuuuguuuccccagaa	uuggggaacacuggggcauacccaaugcucagaccuaacuugcuucuuuguuuccccagaa	gi|307603377|gb|CM00099
gi|307603368|gb|CM001008.1|_13190	-0.9	0.00 ± 0.00		TRUE	237	237	0	0	no	mmu-miR-7648-3p			blast	agggcuggcccgggacgcggcu	cccgcccgcccgcccgccaugg	agggcuggcccgggacgcggcugcccgcccgcccgcccgcccgccaugg	gi|307603368|gb|CM00100
gi|307603382|gb|CM000994.1|_23396	-1.1	0.00 ± 0.00		STAR	42174	42174	0	0	no	mmu-miR-375-5p			blast	uuuguucguucggcucgcguga	gcgacgagccccucgcacaaacc	gcgacgagccccucgcacaaaccggaccugagcguuuuguucguucggcucgcguga	gi|307603382|gb|CM00099
gi|307603374|gb|CM001002.1|_47273	-1.1	0.00 ± 0.00		NA	251	230	21	0	no	mmu-miR-5133			blast	gccgagaggcuggagcugc	cgcuucaguuuuccucagggcuc	gccgagaggcuggagcugcggcagcgcaggccugggccgccgugucucugccgcucgcuucaguuuuccucagggcuc	gi|307603374|gb|CM00100
gi|307603371|gb|CM001005.1|_7653	-1.2	0.00 ± 0.00		TRUE	305	305	0	0	no	mmu-miR-6538			blast	cgggcuccggggcggcga	uaugcuccgggauuccgcu	cgggcuccggggcggcgaugggggacuaugcuccgggauuccgcu	gi|307603371|gb|CM00100
gi|307603377|gb|CM000999.1|_37855	-1.2	0.00 ± 0.00		NA	60	60	0	0	no	mmu-miR-7045-5p			blast	ucuccccccucccccgcacagu	agggcaggggggcggaaagagc	agggcaggggggcggaaagagcggguggggggaggggacugcgcgucacucacuguucuccccccucccccgcacagu	gi|307603377|gb|CM00099
gi|307603366|gb|CM001010.1|_17690	-1.3	0.00 ± 0.00		TRUE	349	349	0	0	no	mmu-miR-6970-3p			blast	ucacgccacccacccugugcuca	aguucagggcugggggcagaga	aguucagggcugggggcagagacugggccugugcgaggucucacgccacccacccugugcuca	gi|307603366|gb|CM00101
gi|307603371|gb|CM001005.1|_7160	-1.3	0.00 ± 0.00		TRUE	60043	53984	0	6059	no	mmu-miR-673-5p			blast	cucacagcucugguccuugga	uccggggcugaguucugugcacc	cucacagcucugguccuuggagcuccagagaaaauguuguccggggcugaguucugugcacc	gi|307603371|gb|CM00100
gi|307603365|gb|CM001011.1|_19188	-1.3	0.00 ± 0.00		TRUE	3758	1708	0	2050	no	mmu-miR-187-5p			blast	gcuacaacacaggacccgggcgc	ucgugucuuguguugcagccg	gcuacaacacaggacccgggcgcugcucugaccccucgugucuuguguugcagccg	gi|307603365|gb|CM00101
gi|307603374|gb|CM001002.1|_47067	-1.4	0.00 ± 0.00		TRUE	41	41	0	0	no	mmu-miR-7087-5p			blast	aggcagguguggagcugg	ggcucucuccccugccaga	aggcagguguggagcuggucucugcagagcagacucaguaggcugcuggcucucuccccugccaga	gi|307603374|gb|CM00100
gi|307603375|gb|CM001001.1|_44562	-1.4	0.00 ± 0.00		STAR	1324	1276	48	0	no	mmu-miR-7068-5p			blast	ucacccuggacugacucucagg	ugaggcucaguaugggguuggggg	ugaggcucaguaugggguggggggugucgucgccugcccgacugaccacccacucacccuggacugacucucagg	gi|307603375|gb|CM00100
gi|307603379|gb|CM000997.1|_33555	-1.4	0.00 ± 0.00		TRUE	1549	1549	0	0	no	mmu-miR-7019-3p			blast	ucaccuuggccgccucucugcaga	ucagacuuuggcugcugccuggcugacc	ucaccuuggccgccucucugcagauggcugucccuggcuagugaucucagacuuuggcugcugccuggcugacc	gi|307603379|gb|CM00099
gi|307603381|gb|CM000995.1|_26200	-1.4	0.00 ± 0.00		TRUE	242	242	0	0	no	mmu-miR-3474			blast	cccugggaggagacgguggauuc	aucugcgucuuguuccagguucagcu	cccugggaggagacgguggauucuuuagagugcaucugcgucuuguuccagguucagcu	gi|307603381|gb|CM00099
gi|307603372|gb|CM001004.1|_5778	-1.4	0.00 ± 0.00		TRUE	254	254	0	0	no	mmu-miR-6930-5p			blast	aagggggugcuugagagguuugg	aggacccuguggagcggccucccagc	aggacccuguggagcggccucccagccacccugugaguacccuagucauggcugguuagggaagggggugcuugagagguuugg	gi|307603372|gb|CM00100
gi|307603368|gb|CM001008.1|_13926	-1.4	0.00 ± 0.00		TRUE	203	203	0	0	no	mmu-miR-1943-5p			blast	aagggaggaucuggcaccugga	cacugugcuugauauuccugaaa	cacugugcuugauauuccugaaagcagaaaccccuggggagccucuggcugggaagggaggaucuggcaccugga	gi|307603368|gb|CM00100
gi|307603372|gb|CM001004.1|_3057	-1.5	0.00 ± 0.00		TRUE	247	247	0	0	no	mmu-miR-5100			blast	ucgaaucccagcggugccucu	aggacuugggaacugaag	aggacuugggaacugaagagcagaguucgaaucccagcggugccucu	gi|307603372|gb|CM00100
gi|307603367|gb|CM001009.1|_15466	-1.5	0.00 ± 0.00		STAR	11174	7998	0	3176	no	mmu-miR-301b-5p			blast	cagugcaaugguauuugcaaagc	gcucugacuagguugcacuacu	gcucugacuagguugcacuacugugcugugagaagcagugcaaugguauuugcaaagc	gi|307603367|gb|CM00100
gi|307603379|gb|CM000997.1|_33164	-1.5	0.00 ± 0.00		STAR	514	514	0	0	no	mmu-miR-7226-5p			blast	ugacacagccauucucugagcagu	ggccagggaaguugauugugugaaggg	ggccagggaaguugauugugugaagggagcaggacuccugacacagccauucucugagcagu	gi|307603379|gb|CM00099
gi|307603376|gb|CM001000.1|_39573	-1.5	0.00 ± 0.00		STAR	65705	46069	0	19636	no	mmu-miR-330-5p			blast	gcaaagcacagggccugcagaga	ucucugggccugugucuuagg	ucucugggccugugucuuaggcucuucaagauccaacgagcaaagcacagggccugcagaga	gi|307603376|gb|CM00100
gi|307603379|gb|CM000997.1|_33556	-1.5	0.00 ± 0.00		STAR	1549	1549	0	0	no	mmu-miR-7019-5p			blast	ucaccuuggccgccucucugcaga	ugggaggggcugucaggaguacaggc	ugggaggggcugucaggaguacaggccgacugcaccgcucaccuuggccgccucucugcaga	gi|307603379|gb|CM00099
gi|307603366|gb|CM001010.1|_17958	-1.5	0.00 ± 0.00		NA	2556	2556	0	0	no	mmu-miR-6977-5p			blast	aaggcguugccugacccugacc	ucacgggugaggcgcugugg	ucacgggugaggcgcuguggcacggcuuggccccgaggcuuccaaggcguugccugacccugacc	gi|307603366|gb|CM00101
gi|307603366|gb|CM001010.1|_17694	-1.5	0.00 ± 0.00		STAR	349	349	0	0	no	mmu-miR-6970-5p			blast	ucacgccacccacccugugcuca	aguucaggcugggagcagaga	aguucaggcugggagcagagacugggccugugcgaggucucacgccacccacccugugcuca	gi|307603366|gb|CM00101

gi|307603364|gb|CM001012.1|_19851	-1.5	0.00 ± 0.00		STAR	2122	2122	0	0	no	mmu-miR-6989-5p			blast	uguccacugucucuggucccagc	uggggacagaggcacaggaugcu	uggggacagaggcacaggaugcuggcugcugaccaccuguccacugucucuggucccagc	gi|307603364|gb|CM00101
gi|307603371|gb|CM001005.1|_7169	-1.6	0.00 ± 0.00		STAR	183	170	0	13	no	mmu-miR-3070-5p			blast	uggugcuauggucagggguaga	agccccugaccuugaaccuggga	agccccugaccuugaaccugggauccuccccaugaucuucuggugcuauggucagggguaga	gi|307603371|gb|CM00100
gi|307603371|gb|CM001005.1|_7314	-1.6	0.00 ± 0.00		TRUE	88892	70038	0	18854	no	mmu-miR-412-5p			blast	uggucgaccagcuggaaaguaau	guacuucaccugguccacuagc	uggucgaccagcuggaaaguaauuguuucuaauguacuucaccugguccacuagc	gi|307603371|gb|CM00100
gi|307603376|gb|CM001000.1|_42513	-1.7	0.00 ± 0.00		TRUE	29	29	0	0	no	mmu-miR-7058-5p			blast	uagaggggaggugaccgugga	ucguuccuuccuuucuucc	uagaggggaggugaccguggaaguugugugcccuuuaucaacuucucguuccuuccuuucuucc	gi|307603376|gb|CM00100
gi|307603378|gb|CM000998.1|_34227	-1.7	0.00 ± 0.00		STAR	195	195	0	0	no	mmu-miR-7689-5p			blast	uuagagccagacugccuggguuu	accgaggcagccuggcuuagugg	accgaggcagccuggcuuaguggaaaaggaacaggcuuuagagccagacugccuggguuu	gi|307603378|gb|CM00099
gi|307603370|gb|CM001006.1|_9475	-1.7	0.00 ± 0.00		TRUE	16750	16750	0	0	no	mmu-miR-449a-5p			blast	uggcaguguauuguuagcuggu	uuguuaaugaggugcgcugucuug	uuguuaaugaggugcgcugucuugccugugugugauggcuuggcaguguauuguuagcuggu	gi|307603370|gb|CM00100
gi|307603381|gb|CM000995.1|_27310	-1.7	0.00 ± 0.00		NA	63	53	10	0	no	mmu-miR-7000-5p			blast	cacccaccugccuguccuccag	agaggacaguugggaagag	agaggacaguugggaagaggaggggacagguaucuggacagagguucugcccucagcacccaccugccuguccuccag	gi|307603381|gb|CM00099
gi|307603368|gb|CM001008.1|_13838	-1.7	0.00 ± 0.00		STAR	571	571	0	0	no	mmu-miR-6952-5p			blast	ucucugacucugccucccacaga	ggugggcggugaaacagagagc	ggugggcggugaaacagagagcuuagcaugggucucugacucugccucccacaga	gi|307603368|gb|CM00100
gi|307603373|gb|CM001003.1|_1800	-1.7	0.00 ± 0.00		STAR	452	452	0	0	no	mmu-miR-6907-5p			blast	aacgugcguuucuuuuguguccu	gacacagggaggcucgaugg	gacacagggaggcucgaugggguuggcugucuucuuacuaacgugcguuucuuuuguguccu	gi|307603373|gb|CM00100
gi|307603372|gb|CM001004.1|_2991	-1.7	0.00 ± 0.00		STAR	12405	12218	0	187	no	mmu-miR-709			blast	ccagccugggcuacacauuua	gaaggaggcagaggcagga	gaaggaggcagaggcaggaggaucaugaguucgaggccagccugggcuacacauuua	gi|307603372|gb|CM00100
gi|307603373|gb|CM001003.1|_705	-1.8	0.00 ± 0.00		STAR	1219	1219	0	0	no	mmu-miR-1982-5p			blast	ucacccuauguucucccacag	gcuugggagggucuggggaggga	gcuugggagggucuggggaggggaguucuggcaccccaaccucucacccuauguucucccacag	gi|307603373|gb|CM00100
gi|307603368|gb|CM001008.1|_13800	-1.8	0.00 ± 0.00		TRUE	538104	538071	33	0	no	mmu-miR-151-5p			blast	ucgaggagcucacagucuagu	uggacugcucagguccugggca	uggacugcucagguccugggcauggagcacagaugauggagcgcuuuccugcccucgaggagcucacagucuagu	gi|307603368|gb|CM00100
gi|307603382|gb|CM000994.1|_23318	-1.8	0.00 ± 0.00		STAR	330	330	0	0	no	mmu-miR-6899-5p			blast	uuguccuucugugucuucugcagg	ggcagcagaacgcagcgggcauga	ggcagcagaacgcagcgggcaugagauccuuucugaccauuguccuucugugucuucugcagg	gi|307603382|gb|CM00099
gi|307603382|gb|CM000994.1|_24387	-1.8	0.00 ± 0.00		TRUE	39586	23014	0	16572	no	mmu-miR-1981-5p			blast	guaaaggcugggcuuagacgugg	caucuaacccuggccuuugaca	guaaaggcugggcuuagacguggccuuuggguguggaaugcacuuccguuuguaaccgccaucuaacccuggccuuugaca	gi|307603382|gb|CM00099
gi|307603366|gb|CM001010.1|_17765	-1.8	0.00 ± 0.00		TRUE	8120	6645	0	1475	no	mmu-miR-877-5p			blast	guagaggagauggcgcaggggaca	uguccucuucucccuccucccagg	guagaggagauggcgcaggggacacaagguaggccuugcgggucuguggacccuuggacauguguccucuucucccuccucccagg	gi|307603366|gb|CM00101
gi|307603365|gb|CM001011.1|_19333	-1.9	0.00 ± 0.00		TRUE	751	751	0	0	no	mmu-miR-6979-3p			blast	uugugucugucuggcucccagu	gaagaagcagauugcggacguggagc	uugugucugucuggcucccagucucugcugagaaccugcagaagagcuuagaucagaugaagaagcagauugcggacguggagc	gi|307603365|gb|CM00101
gi|307603381|gb|CM000995.1|_24951	-1.9	0.00 ± 0.00		STAR	581	555	0	26	no	mmu-miR-3089-5p			blast	agcaucugcugauccugagcugu	ugaguucagggacagcgugucu	ugaguucagggacagcgugucucaguuucucugucugagcaucugcugauccugagcugu	gi|307603381|gb|CM00099
gi|307603376|gb|CM001000.1|_41226	-1.9	0.00 ± 0.00		TRUE	256	256	0	0	no	mmu-miR-3101-5p			blast	gguaccauugacuaaagcuag	ggugugggucaguggggcagc	ggugugggucaguggggcagcaggugggguagguagcagguaccauugacuaaagcuag	gi|307603376|gb|CM00100
gi|307603381|gb|CM000995.1|_24969	-1.9	0.00 ± 0.00		TRUE	33	33	0	0	no	mmu-miR-6998-5p			blast	ccugggcagagggcaaagugacu	ugacuugccucccauaguggcu	ugacuugccucccauaguggcuucccugggugggccugggcagagggcaaagugacu	gi|307603381|gb|CM00099
gi|307603372|gb|CM001004.1|_3242	-1.9	0.00 ± 0.00		STAR	53678	28211	0	25467	no	mmu-miR-324-5p			blast	acugccccaggugcugcuggg	cgcauccccuagggcauuggugu	cgcauccccuagggcauugguguaaagcuggagacccacugccccaggugcugcuggg	gi|307603372|gb|CM00100
gi|307603372|gb|CM001004.1|_3826	-1.9	0.00 ± 0.00		TRUE	127	127	0	0	no	mmu-miR-8103			blast	ucuccuguucucuguucucccu	caggagcaaagggcaugacagg	ucuccuguucucuguucucccucugacugcaggagcaaagggcaugacagg	gi|307603372|gb|CM00100
gi|307603376|gb|CM001000.1|_41506	-2.0	0.00 ± 0.00		STAR	263	241	0	22	no	mmu-miR-7054-5p			blast	uccaacuuggccccuuccag	gaaggugguugggcugagu	gaaggugguugggcugaguacucacagaacagaguccaacuuggccccuuccag	gi|307603376|gb|CM00100
gi|307603366|gb|CM001010.1|_16195	-2.0	0.00 ± 0.00		TRUE	3386424	3371554	14870	0	no	mmu-miR-125a-5p			blast	ucccugagacccuuuaaccugug	uggggaaaaggguuuuucuggucc	uggggaaaaggguuuuucugguccaccauagcuacacugccggccucuggguccugagacccuuuaaccugug	gi|307603366|gb|CM00101
gi|307603366|gb|CM001010.1|_16277	-2.1	0.00 ± 0.00		TRUE	513	513	0	0	no	mmu-miR-5125			blast	uucugccugggauuuccuugu	aaggagagccccaugccuuugg	aaggagagccccaugccuuuggacaugcuguuccuucugccugggauuuccuugu	gi|307603366|gb|CM00101
gi|307603371|gb|CM001005.1|_6796	-2.1	0.00 ± 0.00		STAR	24594	24123	0	471	no	mmu-miR-709			blast	ccagccugggcuacacauuuu	gaaggaggcagaggcagga	gaaggaggcagaggcaggaggaucacaaguucgaggccagccugggcuacacauuuu	gi|307603371|gb|CM00100
gi|307603381|gb|CM000995.1|_27340	-2.1	0.00 ± 0.00		TRUE	166633	166236	0	397	no	mmu-miR-129-5p			blast	cuuuuugcggucugggcuugc	guagacugccuuucgcgaau	guagacugccuuucgcgaaucuuuuugcggucugggcuugc	gi|307603381|gb|CM00099
gi|307603371|gb|CM001005.1|_8383	-2.1	0.00 ± 0.00		TRUE	705	705	0	0	no	mmu-miR-6939-3p			blast	ccucacuugacccgcugcagg	uguggcagauggacucaagaggca	ccucacuugacccgcugcagggaagaaugggccaccgccauucagacuguggcagauggacucaagaggca	gi|307603371|gb|CM00100
gi|307603371|gb|CM001005.1|_6336	-2.1	0.00 ± 0.00		TRUE	1481	1265	0	216	no	mmu-miR-3066-5p			blast	uugguugcuguagauuaaguagu	uuaauagaccgcaaccugca	uugguugcuguagauuaaguaguuuaauaugaugcugcacuuaauagaccgcaaccugca	gi|307603371|gb|CM00100
gi|307603376|gb|CM001000.1|_40881	-2.1	0.00 ± 0.00		STAR	1308	1308	0	0	no	mmu-miR-3104-5p			blast	gacgcucugcuuugcucccccaga	uagggggcaggagccggagcccucug	uagggggcaggagccggagcccucugcugaacugacagacgcucugcuuugcucccccaga	gi|307603376|gb|CM00100
gi|307603372|gb|CM001004.1|_3804	-2.2	0.00 ± 0.00		TRUE	133	133	0	0	no	mmu-miR-196a-5p			blast	uagguaguuucauguuguuggg	caacgacaucaaaccaccugau	uagguaguuucauguuguugggccuggcuuucugaacacaacgacaucaaaccaccugau	gi|307603372|gb|CM00100
gi|307603366|gb|CM001010.1|_16193	-2.2	0.00 ± 0.00		TRUE	817295	817295	0	0	no	mmu-let-7e-5p			blast	ugagguaggagguuguauaguu	cugucaaucugcccaccugccgcgcc	cugucaaucugcccaccugccgcgcccccgggcugagguaggagguuguauaguu	gi|307603366|gb|CM00101
gi|307603368|gb|CM001008.1|_13966	-2.2	0.00 ± 0.00		TRUE	68	68	0	0	no	mmu-miR-7213-5p			blast	acugggucucuugagguagcc	gaaucucagcucccccagaucu	gaaucucagcucccccagaucucuacccugcaaggaaaacuucguucaucucagacugggucucuugagguagcc	gi|307603368|gb|CM00100
gi|307603379|gb|CM000997.1|_33842	-2.2	0.00 ± 0.00		TRUE	201	201	0	0	no	mmu-miR-7658-5p			blast	ugugggcguggcggugcugga	cugcauguucacaagacgcagc	cugcauguucacaagacgcagcagugugggcguggcggugcugga	gi|307603379|gb|CM00099
gi|307603373|gb|CM001003.1|_913	-2.3	0.00 ± 0.00		TRUE	88	88	0	0	no	mmu-miR-1931			blast	ugcaagggcuggugcgauggcu	cuguugcagagccuuugaauu	cuguugcagagccuuugaauugaagcugcuuuaaaauuccaaugcaagggcuggugcgauggcu	gi|307603373|gb|CM00100
gi|307603376|gb|CM001000.1|_39752	-2.4	0.00 ± 0.00		TRUE	5056	5056	0	0	no	mmu-miR-1964-3p			blast	ccgacuucugggcuccggcuuu	aggagguagccucggcu	ccgacuucugggcuccggcuuuggaaggaaggagguagccucggcu	gi|307603376|gb|CM00100
gi|307603382|gb|CM000994.1|_22425	-2.4	0.00 ± 0.00		STAR	914	914	0	0	no	mmu-miR-7682-5p			blast	uguggguuggguuggcuuugg	uaggcacgacucuugccacaga	uaggcacgacucuugccacagaaacugagugcauuuguccuguggguuggguuggcuuugg	gi|307603382|gb|CM00099
gi|307603372|gb|CM001004.1|_2764	-2.4	0.00 ± 0.00		TRUE	2646429	2646429	0	0	no	mmu-miR-103-3p			blast	agcagcauuguacagggcuauga	augcgguuugagugauaugugcugcuug	agcagcauuguacagggcuaugaaggcauugagacuuguucuucaugcgguuugagugauaugugcugcuug	gi|307603372|gb|CM00100
gi|307603363|gb|CM001013.1|_48281	-2.4	0.00 ± 0.00		NA	6779	6617	162	0	no	mmu-miR-709			blast	ccagccugggcuacacauuu	uuguggugcugcaugcuggua	uuguggugcugcaugcugguaggauuuggugaaggaggcagaggcaggaggaucacaaauucgaggccagccugggcuacacauuu	gi|307603363|gb|CM00101
gi|307603374|gb|CM001002.1|_46358	-2.5	0.00 ± 0.00		STAR	2505	2505	0	0	no	mmu-miR-466i-5p			blast	cacagcaggcacagacagga	gugugugugugugugugug	cacagcaggcacagacaggacuuaagaacaaaagguuuauuuguuuggcuuguguacgugugugugugugugugug	gi|307603374|gb|CM00100
gi|307603366|gb|CM001010.1|_16448	-2.6	0.00 ± 0.00		TRUE	28	28	0	0	no	mmu-miR-7667-3p			blast	agggacugcagagauggcacug	gcuccagucucagugagacccugu	agggacugcagagauggcacuggggagggaggugccuagccuaaucuugagcuccagucucagugagacccugu	gi|307603366|gb|CM00101
gi|307603379|gb|CM000997.1|_33370	-2.6	0.00 ± 0.00		NA	16327	15405	922	0	no	mmu-miR-709			blast	ccagccugggcuacacauuua	aguggugcucacgcuggua	aguggugcucacgcugguaggauuugcugaaggaggcagaggcaggaggaucacgaguuugaggccagccugggcuacacauuua	gi|307603379|gb|CM00099
gi|307603369|gb|CM001007.1|_10929	-2.6	0.00 ± 0.00		STAR	479	479	0	0	no	mmu-miR-3076-5p			blast	cgcacucuggucuucccuugcag	acaggggaagcucagugccag	acaggggaagcucagugccagccuuucucuuagccgcacucuggucuucccuugcag	gi|307603369|gb|CM00100
gi|307603368|gb|CM001008.1|_13121	-2.7	0.00 ± 0.00		TRUE	5777842	5777842	0	0	no	mmu-let-7c-5p			blast	ugagguaguagguuguaugguu	ccacaacauccagcucuacg	ccacaacauccagcucuacgccaagacugacugacggccuuuggggugagguaguagguuguaugguu	gi|307603368|gb|CM00100
gi|307603369|gb|CM001007.1|_11999	-2.7	0.00 ± 0.00		TRUE	676	676	0	0	no	mmu-miR-6948-3p			blast	uguccuuccugucugaccaca	uguucagaaggaggcaga	uguccuuccugucugaccacagauuguucagaaggaggcaga	gi|307603369|gb|CM00100
gi|307603379|gb|CM000997.1|_33342	-2.8	0.00 ± 0.00		STAR	389	345	0	44	no	mmu-miR-7016-5p			blast	accugccuccugcucuccccagaa	agggaggggagcgagaguaggcca	agggaggggagcgagaguaggccacuauggacaugcugagccugaccugccuccugcucuccccagaa	gi|307603379|gb|CM00099
gi|307603376|gb|CM001000.1|_39759	-2.9	0.00 ± 0.00		TRUE	227	227	0	0	no	mmu-miR-7668-5p			blast	ucugaauaaccugcucucugu	uaagaggagguucuucuggac	uaagaggagguucuucuggacacggggucaugaguucuguaacacugagaaaccuaguugccacucugaauaaccugcucucugu	gi|307603376|gb|CM00100
gi|307603366|gb|CM001010.1|_17689	-2.9	0.00 ± 0.00		TRUE	349	349	0	0	no	mmu-miR-6970-3p			blast	ucacgccacccacccugugcuca	aguggaggccaagaaagagaggaugaga	ucacgccacccacccugugcucagguacuaugcugcaguggaggccaagaaagagaggaugaga	gi|307603366|gb|CM00101
gi|307603382|gb|CM000994.1|_22787	-2.9	0.00 ± 0.00		TRUE	32	32	0	0	no	mmu-miR-215-5p			blast	augaccuaugauuugacagac	cucucagcaucaacgguguaca	cucucagcaucaacgguguacaggagaaugaccuaugauuugacagac	gi|307603382|gb|CM00099
gi|307603364|gb|CM001012.1|_20421	-3.0	0.00 ± 0.00		TRUE	83386	83386	0	0	no	mmu-miR-146b-5p			blast	ugagaacugaauuccauaggcugu	agcccacagaagagagcucugcagg	agcccacagaagagagcucugcagggcaggcagcauccagacugagagaacuuuggccaccuggcucugagaacugaauuccauaggcugu	gi|307603364|gb|CM00101
gi|307603378|gb|CM000998.1|_34309	-3.0	0.00 ± 0.00		TRUE	857520	857520	0	0	no	mmu-miR-218-5p			blast	uugugcuugaucuaaccaugug	agugguugguguagugauaaug	agugguugguguagugauaauggagcgagauuuucuguugugcuugaucuaaccaugug	gi|307603378|gb|CM00099
gi|307603370|gb|CM001006.1|_9676	-3.0	0.00 ± 0.00		TRUE	631550	631481	69	0	no	mmu-miR-1983			blast	cucaccuggagcauguuuucu	aguauaugugcgggugaugc	aguauaugugcgggugaugccgagguugugaguucgagccucaccuggagcauguuuucu	gi|307603370|gb|CM00100
gi|307603382|gb|CM000994.1|_22426	-3.1	0.00 ± 0.00		TRUE	914	914	0	0	no	mmu-miR-7682-3p			blast	uguggguuggguuggcuuugg	aaacuauuugccuuaucacaag	uguggguuggguuggcuuugguuggcuuuggcauagcuacgaaacuauuugccuuaucacaag	gi|307603382|gb|CM00099
gi|307603381|gb|CM000995.1|_24941	-3.1	0.00 ± 0.00		NA	44542	44417	125	0	no	mmu-miR-709			blast	ccagccugggcuacacauuuu	agugguggcacacacuggua	agugguggcacacacugguaggauuugcuggaggaggcagaggcaggaggaucacgaguucaaggccagccugggcuacacauuuu	gi|307603381|gb|CM00099
gi|307603372|gb|CM001004.1|_5897	-3.1	0.00 ± 0.00		TRUE	1910	1542	0	368	no	mmu-miR-3064-5p			blast	ucuggcuguuguggugugcaa	uugccacacugcaacaccuuaca	ucuggcuguuguggugugcaaaacuccguacauugcuauuuugccacacugcaacaccuuaca	gi|307603372|gb|CM00100
gi|307603364|gb|CM001012.1|_21046	-3.2	0.00 ± 0.00		NA	65833	64496	1337	0	no	mmu-miR-709			blast	ccagccugggcuacacauuuuu	uuguggugcacacgcuggua	uuguggugcacacgcugguaggauuugcugaaggaggcagaggcaggaggaucacgaguucgaggccagccugggcuacacauuuuu	gi|307603364|gb|CM00101
gi|307603374|gb|CM001002.1|_47130	-3.5	0.00 ± 0.00		TRUE	18576	18576	0	0	no	mmu-miR-34c-5p			blast	aggcaguguaguuagcugauugc	accagcuaaagacugcaugcuuuu	accagcuaaagacugcaugcuuuuuucugagucuaguuacuaggcaguguaguuagcugauugc	gi|307603374|gb|CM00100
gi|307603373|gb|CM001003.1|_753	-3.5	0.00 ± 0.00		TRUE	1412	1412	0	0	no	mmu-miR-3057-5p			blast	auuggagcugagauucugcgggau	cuccagagcaucuaagaacccuuga	cuccagagcaucuaagaacccuugacuuagaaaccuugagguguccugaaggugugugcauuggagcugagauucugcgggau	gi|307603373|gb|CM00100
gi|307603371|gb|CM001005.1|_7251	-3.5	0.00 ± 0.00		STAR	404	380	0	24	no	mmu-miR-1197-5p			blast	uaggacacauggucuacuucu	cgguugaccaugguguguacg	cgguugaccaugguguguacgcuuuauuuaugacguaggacacauggucuacuucu	gi|307603371|gb|CM00100
gi|307603379|gb|CM000997.1|_32013	-3.6	0.00 ± 0.00		TRUE	42	42	0	0	no	mmu-miR-6399			blast	uugcaaugaugguauucugagg	ucagauaccauuguuugauc	ucagauaccauuguuugauccuugaaccuuuuagagguaaagcuguugcaaugaugguauucugagg	gi|307603379|gb|CM00099
gi|307603382|gb|CM000994.1|_21506	-3.6	0.00 ± 0.00		TRUE	165	165	0	0	no	mmu-miR-6896-5p			blast	cuuuguaggcgacgagaacccg	cucucucucaccuacaaacaggu	cuuuguaggcgacgagaacccgugccugguguguaauaaucacuguuucucucucucaccuacaaacaggu	gi|307603382|gb|CM00099
gi|307603381|gb|CM000995.1|_27241	-3.7	0.00 ± 0.00		TRUE	48479	48479	0	0	no	mmu-miR-130a-3p			blast	cagugcaauguuaaaagggca	ccuccucagcaucacugca	cagugcaauguuaaaagggcaucggccuuguaguacuacccagugccggcagccuccucagcaucacugca	gi|307603381|gb|CM00099
gi|307603367|gb|CM001009.1|_15776	-3.9	0.00 ± 0.00		NA	39146	37809	1337	0	no	mmu-miR-709			blast	ccagccugggcuacacauuuu	agugguggcgcacgcgggua	agugguggcgcacgcggguaggauuugcugaaggaggcagaggcaggaggaucacgaguucgaggccagccugggcuacacauuuu	gi|307603367|gb|CM00100
gi|307603372|gb|CM001004.1|_5404	-4.0	0.00 ± 0.00		TRUE	397324	397324	0	0	no	mmu-miR-21a-5p			blast	uagcuuaucagacugauguugac	cgugacaucgcauggcugua	cgugacaucgcauggcuguaccaccuugucggauagcuuaucagacugauguugac	gi|307603372|gb|CM00100
gi|307603381|gb|CM000995.1|_26971	-4.0	0.00 ± 0.00		TRUE	103	103	0	0	no	mmu-miR-5129-3p			blast	aaugugccugugcaucucuuccca	ggagacagaugaagaggacaagcuuca	aaugugccugugcaucucuucccacagguggugaacuaugacaacguaguggacgcaggcucggagacagaugaagaggacaagcuuca	gi|307603381|gb|CM00099
gi|307603371|gb|CM001005.1|_7286	-4.0	0.00 ± 0.00		TRUE	82619	82619	0	0	no	mmu-miR-381-3p			blast	uauacaagggcaagcucucugu	cgagggaugugguaugugugug	uauacaagggcaagcucucugugaguaucaaaccuuaucuuggaucccguccacacucagcgagggaugugguaugugugug	gi|307603371|gb|CM00100
gi|307603370|gb|CM001006.1|_10036	-4.6	0.00 ± 0.00		TRUE	4735562	4735505	57	0	no	mmu-let-7a-5p			blast	ugagguaguagguuguauaguu	cugugauuacuuucaacauucacc	cugugauuacuuucaacauucacccuggauguucucuucacugugggaugagguaguagguuguauaguu	gi|307603370|gb|CM00100
gi|307603370|gb|CM001006.1|_9096	-4.8	0.00 ± 0.00		TRUE	767992	767992	0	0	no	mmu-miR-24-3p			blast	uggcucaguucagcaggaacag	guaagugccugaggcagg	uggcucaguucagcaggaacaggagucgagcccuugagcaaaaagccuacccaucuguaagugccugaggcagg	gi|307603370|gb|CM00100
gi|307603369|gb|CM001007.1|_12144	-5.1	0.00 ± 0.00		TRUE	27929	27929	0	0	no	mmu-miR-15a-5p			blast	uagcagcacauaaugguuugug	uuacuauugaggugcuagg	uuacuauugaggugcuaggaguuuucaaaaccaacccuuggaguaaaguagcagcacauaaugguuugug	gi|307603369|gb|CM00100
gi|307603367|gb|CM001009.1|_14825	-5.1	0.00 ± 0.00		NA	45339	44417	922	0	no	mmu-miR-709			blast	ccagccugggcuacacauuuu	agugguggugcacacuggua	agugguggugcacacugguaggauuugcugaaggaggcagaggcaggaggaucacgaguuugaggccagccugggcuacacauuuu	gi|307603367|gb|CM00100
gi|307603369|gb|CM001007.1|_11503	-5.1	0.00 ± 0.00		TRUE	74513	74513	0	0	no	mmu-miR-17-5p			blast	caaagugcuuacagugcagguag	ccuugggacugaagcugugac	ccuugggacugaagcugugaccagucagaauaaugucaaagugcuuacagugcagguag	gi|307603369|gb|CM00100
gi|307603374|gb|CM001002.1|_47378	-5.3	0.00 ± 0.00		TRUE	1128	1128	0	0	no	mmu-miR-190a-5p			blast	ugauauguuugauauauuagguu	ccauauucucaagcucagucaaa	ccauauucucaagcucagucaaaccuggaugccuuuucugcaggccucugugugauauguuugauauauuagguu	gi|307603374|gb|CM00100
gi|307603366|gb|CM001010.1|_17284	-5.8	0.00 ± 0.00		NA	12589	12539	50	0	no	mmu-miR-709			blast	ccugggcuacacauuuuu	gaaugugcugaaggaggca	gaaugugcugaaggaggcagaggcaggaggaucacgagaucgaggcaaaccugggcuacacauuuuu	gi|307603366|gb|CM00101
gi|307603368|gb|CM001008.1|_13401	-6.3	0.00 ± 0.00		TRUE	133	133	0	0	no	mmu-miR-196a-5p			blast	uagguaguuucauguuguuggg	cugcuugcucagcugaucugug	cugcuugcucagcugaucuguggcuuagguaguuucauguuguuggg	gi|307603368|gb|CM00100
gi|307603370|gb|CM001006.1|_10032	-6.3	0.00 ± 0.00		TRUE	722792	721130	1662	0	no	mmu-let-7d-5p			blast	agagguaguagguugcauaguu	gugugugaagugcaucugc	gugugugaagugcaucugccaaguagaagaccagcaagaauaaaaauggguuccuaggaagagguaguagguugcauaguu	gi|307603370|gb|CM00100
gi|307603372|gb|CM001004.1|_2475	-6.4	0.00 ± 0.00		TRUE	620	620	0	0	no	mmu-miR-7651-5p			blast	aaguggaaagccagccugagau	uugaggcuaguaugguaaucuacaccu	uugaggcuaguaugguaaucuacaccuguaaucccagagcucaggaucuuaaguggaaagccagccugagau	gi|307603372|gb|CM00100
gi|307603379|gb|CM000997.1|_32472	-7.2	0.00 ± 0.00		TRUE	5645	5645	0	0	no	mmu-miR-873a-5p			blast	ugcaggaacuugugagucucc	aacaacgagcaccagcuua	aacaacgagcaccagcuuaaaaucaguuugugugcauuugcaggaacuugugagucucc	gi|307603379|gb|CM00099
gi|307603381|gb|CM000995.1|_24645	-7.4	0.00 ± 0.00		TRUE	420	420	0	0	no	mmu-miR-669h-5p			blast	augcaugggguauaguugagugc	auucaugugaugcccaccaaaa	auucaugugaugcccaccaaaaauaugcaugggguauaguugagugc	gi|307603381|gb|CM00099
gi|307603378|gb|CM000998.1|_34779	-8.0	0.00 ± 0.00		STAR	102890	97757	2	5131	no	mmu-miR-6240			blast	ugugauuucugcccagugcucuga	aagcaucgcgaaggcccacg	aagcaucgcgaaggcccacggcggguguugacucgaugugauuucugcccagugcucuga	gi|307603378|gb|CM00099
gi|307603382|gb|CM000994.1|_22379	-8.5	0.00 ± 0.00		TRUE	2127050	2127050	0	0	no	mmu-miR-181b-5p			blast	aacauucauugcugucggugggu	ccgccucuucagagacuauguuua	ccgccucuucagagacuauguuuacaaaggucacaaucaacauucauugcugucggugggu	gi|307603382|gb|CM00099
gi|307603382|gb|CM000994.1|_23744	-1.0e+1	0.00 ± 0.00		TRUE	121	79	42	0	no	mmu-miR-466i-5p			blast	ugugugugugugugugugu	aucuggauacaauagacgau	ugugugugugugugugugugugguuucauggaacucugugcucuuucugugcucacaucuggauacaauagacgau	gi|307603382|gb|CM00099
gi|307603380|gb|CM000996.1|_29498	-1.3e+1	0.00 ± 0.00		TRUE	192629	192629	0	0	no	mmu-let-7j			blast	ugagguauuaguuugugcuguua	ccuugcucagauuaaaagccuggag	ugagguauuaguuugugcuguuauaugaaucgaauaauaucccuugcucagauuaaaagccuggag	gi|307603380|gb|CM00099
gi|307603368|gb|CM001008.1|_13686	-2.0e+1	0.00 ± 0.00		TRUE	3302	3302	0	0	no	mmu-miR-28c			blast	gaggagcucacagucuauugaa	caaagacaaaugagauaugaucccua	caaagacaaaugagauaugaucccuacccuugaggagcucacagucuauugaa	gi|307603368|gb|CM00100
gi|307603374|gb|CM001002.1|_45667	-2.4e+1	0.00 ± 0.00		TRUE	18304760	18302095	2665	0	no	mmu-miR-125b-5p			blast	ucccugagacccuaacuuguga	gcuuguuuugucucaagaaagaau	gcuuguuuugucucaagaaagaaugaaggaaucguguugcgcuccccucagucccugagacccuaacuuguga	gi|307603374|gb|CM00100
gi|307603363|gb|CM001013.1|_48798	-2.7e+1	0.00 ± 0.00		TRUE	2071	2071	0	0	no	mmu-miR-1264-3p			blast	caaaucuuauuugagcaccugu	uugugaucuuauaugggagagaau	caaaucuuauuugagcaccuguuauguggaggauaaaaaacuacaaaccaaacagagcuugugaucuuauaugggagagaau	gi|307603363|gb|CM00101

mature miRBase miRNAs not detected by miRDeep2

miRBase precursor id	total read count	mature read count(s)	star read count	remaining reads	UCSC browser	NCBI blastn	miRBase mature sequence(s)	miRBase star sequence(s)	miRBase precursor sequence
mmu-mir-99b	5262984	5262984	0	0	-	blast	caagcucgugucuguggguccg<br>cacccguagaaccgaccuugcg	-	ggcacccacccguagaaccgaccuugcggggccuucgccgcacacaagcucgugucugugggguccguguc
mmu-mir-3968	517609	517609	0	0	-	blast	cgaaucccacuccagacacca	-	acgccgagcguguggugguaggauccguguagcaccgucuguguguuguugugccacacagagaacuugagcgaaucccacuccagacaccaugaguagg
mmu-mir-484	341068	341068	0	0	-	blast	ucaggcucaguccccucccgau	-	cucgucaggcucaguccccucccgauaaaccucaaaauagggucuuaccuagggggcuggcggcgua
mmu-mir-101b	61473	61036	0	437	-	blast	guacaguacugugauagcu<br>ucgguuaucaugguaccgaugcu	-	aucugagacugaacugcccuuuuucgguuaucaugguaccgaugcuguagcucugaaagguacaguacugugauagcugaagaauggcggugccauc
mmu-mir-744	47260	47185	0	75	-	blast	cuguugccacuaaccucaaccu<br>ugcggggcuagggcuaacagca	-	ggcugggcaaggugcggggcuagggcuaacagcagucuuacugacgguuuccuggaaaccacacacaugcuguugccacuaaccucaaccuuacucgguc
mmu-mir-136	54343	47643	0	6700	-	blast	aucaucgucucaaaugagucuu<br>acuccauuuguuuugaugaugg	-	gaggacuccauuuguuuugaugauggauucuuaagcuccaucaucgucucaaaugagucuuc
mmu-mir-1306	24753	24553	0	200	-	blast	caccaccuccccugcaaacgucc<br>acguuggcucugguggugaug	-	cagucuccaccaccuccccugcaaacguccagugaugcagagguaauggacguuggcucugguggugauggacaguccg
mmu-mir-341	16126	16062	0	64	-	blast	ucggucgaucggucggucggu<br>cggucggccgaucgcucgguc	-	aaaaugaugaugucaguuggccggucggccgaucgcucggucugucagucagucggucggucgaucggucggucggucagucggcuuccugucuuc
mmu-mir-6944	12473	12290	0	183	-	blast	gugagagcggggggaguggcaag<br>uaacucuucccuugugcccucag	-	gugagagcggggggaguggcaagugagacggaugggcggggcuggcucagacugcuagccccgcccagcucucuaacucuucccuugugcccucag
mmu-mir-467c	12042	12013	0	29	-	blast	uaagugcgugcauguauaugug<br>auauacauacacacaccuauac	-	ccuuugugcauaagugcgugcauguauaugugugugauauauuuuaugcauauacauacacacaccuauacacacaugcacacagacaugcgagaauggc
mmu-mir-3475	10328	10298	0	30	-	blast	ucuggaggcacaugguuugaa<br>aaaucauguacccccacagagg	-	caaaucauguacccccacagaggguccagaugguucuuaguucuucuggaggcacaugguuugaa
mmu-mir-3078	13058	13058	0	0	-	blast	uugcuggggguagucuuuagg<br>caaagccuagacugcagcuaccu	-	acuggcagacugccccaaagccuagacugcagcuaccuuguauugugcuuggggguugcuggggguagucuuuaggggaagcuguuugu
mmu-mir-378c	10040	10040	0	0	-	blast	acuggacuuggagucagaagc	-	uauacuggacuuggagucagaagccuuggguuugaggacugggcuagccucucauuaguugggguguguuaaacccaacauguagucuaucugaucugga
mmu-mir-690	19663	8948	0	10715	-	blast	aaaggcuaggcucacaaccaaa	-	uguguuuuuguggagcuaauuggcuguauuaaagugcuaguaagaaacauucuccuccagcuggagagauggcucagcuguuaaaggcuaggcucacaaccaaaauaua
mmu-mir-3093	10888	10836	0	52	-	blast	uguggacaccgugggagguugg<br>cgcaccccgcggagcucacacu	-	cgagcucggcucugccgcaccccgcggagcucacacucgaccuuccagagaagggugugggacaccgugggagguuggggaggccucg
mmu-mir-377	12550	6420	0	6130	-	blast	agagguugcccuuggugaauuc<br>aucacacaaaggcaacuuuugu	-	ugagcagagguugcccuuggugaauucgcuuuauuugauguugaaucacacaaaggcaacuuuuguuug
mmu-mir-451a	5145	5131	0	14	-	blast	aaaccguuaccauuacugaguu	-	cuugggaauggcgaggaaaccguuaccauuacugaguuuaguaaugguaacgguucucuugcugcucccaca
mmu-mir-3102	6215	6215	0	0	-	blast	gugaguggccagggugggggcug<br>gagcaccccauuggcuacccaca<br>gggugugcaggcaggagagcc<br>cucuacucccugccccagcca	-	gugaguggccagggugggggcugggugguggugcaggcaggagagccauugaucuauugugggcucuacucccugccccagccagagcaccccauuggcuacccacag
mmu-mir-223	4919	4910	0	9	-	blast	cguguauuugacaagcugaguug<br>ugucaguuugucaaauacccca	-	ucuggccaucugcagugucacgcuccguguauuugacaagcugaguuggacacucugugugguagagugucaguuugucaaauaccccaaguguggcucaugccuaucag
mmu-mir-19a	4175	4142	0	33	-	blast	ugugcaaaucuaugcaaaacuga<br>uaguuuugcauaguugcacuac	-	gcagcccucuguuaguuuugcauaguugcacuacaagaagaauguaguugugcaaaucuaugcaaaacugauggugggccugc
mmu-mir-3963	2801	2801	0	0	-	blast	uguaucccacuucugacac	-	ugggauguaucccacuucugacacuggugauauuguuuaauaucuuacagguuuuagguggaauauuuucc
mmu-mir-6238	2674	2674	0	0	-	blast	uuauuagucaguggaggaaaug	-	cugaaccccuuaagcacucaaguuauugcccuccuaucugaccugagugaacuaggcuauuucuaaagaccuuuauuagucaguggaggaaaugaagggagccaagccaggugugaaaauauucccgau

mmu-mir-142a	2815	2554	0	261	-	blast	uguaguguuuccuacuuuaugga<br>cauaaaguagaaagcacuacu	-	acccauaaaguagaaagcacuacuaacagcacuggagggguguaguguuuccuacuuuauggaug
mmu-mir-664	2690	2690	0	0	-	blast	cuggcuggggaaaaugacugg<br>uauucauuuacuccccagccua	-	cuggcuggggaaaaugacuggauagaaaacauuauucuauucauuuacuccccagccuacacaugaaag
mmu-mir-8112	2733	1892	0	841	-	blast	ucuccgccaccuccaccgca	-	cuccgccuccucccccccucccaccccgccugcucugcggcggaggcggcggcggcagcggugucucugccucuccgccaccuccaccgcagccucugacccccggccggcguuguuggcggggaggg
mmu-mir-7224	2500	1907	0	593	-	blast	uccacugagaggaccacccac<br>ggguaggccccucagugaaga	-	ggguaggccccucagugaagauuucaggacacuuagaucuccacugagaggaccacccac
mmu-mir-7688	1621	1621	0	0	-	blast	ucuggcuuaugccuagcuuuc<br>uagcugggcaugaucugaugagc	-	uagcugggcaugaucugaugagcucacagccaggcucuggcuuaugccuagcuuuc
mmu-mir-935	1550	1529	0	21	-	blast	cccaguuaccgcuuccgcuaccgc	-	ggcggcggcgcgggcggcaguggcgggagcggccccucggccauccuccgucugcccaguuaccgcuuccgcuaccgccgccgcucccgcu
mmu-mir-3060	1471	1471	0	0	-	blast	ccauagcacagaagcacuccca<br>gggagugcuucgugcuucug	-	gggcgugggggugggagugcuucgugcuucugguugaagagaaaaugaccauagcacagaagcacucccaccagcuuugucu
mmu-mir-30c-1	1552450	1552450	0	0	-	blast	cugggagaggguuguuuacucc<br>uguaaacauccuacacucucagc	-	accauguuguagugugugugaaacauccuacacucucagcugugagcucaagguggcugggagaggguuguuuacuccuucugccaugga
mmu-mir-2137	930	930	0	0	-	blast	gccggcgggagccccagggag	-	guaguauaccuccuuccugcugccuuguuggcuugccggcgggagccccagggaguagagcauugc
mmu-mir-297c	2492	2492	0	0	-	blast	uauacauacacacauacccaua<br>uauacauacacacauacccaua<br>uauacauacacacauacccaua<br>auguaugugugcauguacaugu	-	cugugugauauguaugugugcauguacauguguguauaugaauauacauauacauacacacauacccauacaaacaugcauacaaacacacaaaaau
mmu-mir-1194	754	748	0	6	-	blast	gaaugaguaacugcuagauccu	-	acaccuucugcuggagacaauauaaggacauuggaagaagggagucuagcucuugcccuuugccugcuugcugcgugagaaugaguaacugcuagauccuuggacuuccauucacagcug
mmu-mir-466g	737	737	0	0	-	blast	auacagacacaugcacacaca	-	uguguuuucgugugugugcauguggauguauguauaugauuuugcauauacagacacaugcacacacaugcggcacacac
mmu-mir-5121	698	698	0	0	-	blast	agcuugugaugagacaucucc	-	ggugaguuggugaugguggugacauguaggacagggUaggcucuggcagcuugugaugagacaucucccacucau
mmu-mir-18b	1156	1156	0	0	-	blast	uaaggugcaucuagugcuguuag<br>uacugcccuaaaugccccuucu	-	ucucuuguguuaaggugcaucuagugcuguuagugaagcagcuuacaaucuacugcccuaaaugccccuucucgcacaggcua
mmu-mir-30f	676	676	0	0	-	blast	guaaacauccgacugaaagcuc	-	uggcagccugccgcuguaaacauccgacugaaagcuccucucaacaccuuacagcuuccagucaaggauguuuacaguagcaaagacugccc
mmu-mir-3535	977	615	0	362	-	blast	uggauaugaugacugauuaccugaga	-	uuuggggcugucuuuauguuguugcugagaaaaugacaacuggauaugaugacugauuaccugagaaauaauugaugaaaucucaagaaaauuccucuagauagucaaguucugauccagcuaugucaacucaaagcagcaaccuug
mmu-mir-677	642	642	0	0	-	blast	gaagccagaugccguuccugagaagg<br>uucagugaugauuagcuucuga	-	gagccuucagugaugauuagcuucugacuuucguucuucugaauuugcugaagccagaugccguuccugagaaggga
mmu-mir-7021	541	541	0	0	-	blast	ucucugugcuucugcuccuuag<br>auaggaguugauuacagagcaa	-	gagucauaggaguugauuacagagcaagcgcugugucuucaucucugugcuucugcuccuuag
mmu-mir-378d	529	529	0	0	-	blast	acuggccuuggagucagaaggu	-	agaugcugggauagcacuggccuuggagucagaagguuugugauuugguggcagcucugccauccaauaucccugugauuuuuagggugucaguuucccuuuaagcaccg
mmu-mir-3084-2	711	700	0	11	-	blast	uucugccagucuccuucagac<br>guugaagguuaauuagcagagu	-	caaauaguugaagguuaauuagcagaguaacugaauauaguaauucugccagucuccuucagacaaaaa
mmu-mir-1224	487	487	0	0	-	blast	ccccaccucuucucuccucag<br>gugaggacuggggagguggag	-	gugaggacuggggagguggaggguagcaucauuagagccagagcucugucucagcucccucucccccaccucuucucuccucag
mmu-mir-3067	497	497	0	0	-	blast	aguucucaggcccgcugugguguu<br>ccaagcggcugcccugggagagg	-	gccccagcuuccccaguucucaggcccgcuguggugugaaguuagguucaccaagcggcugcccugggagagggggaggugugc
mmu-mir-3470b	757	472	0	285	-	blast	ucacucuguagaccaggcugg	-	ggauucucuguguagccuuggcuguccuggcacucacucuguagaccaggcuggccuugaacucaaaaauucaccugccucugccuccccgagugcugggacuaaaggcaugcgcuaccaaugcccg
mmu-mir-33	530	530	0	0	-	blast	caauguuuccacagugcaucac<br>gugcauuguaguugcauugca	-	cuguggugcauuguaguugcauugcauguucuggcaauaccugugcaauguuuccacagugcaucacgg
mmu-mir-7073	374	357	0	17	-	blast	auagccuuuuccuccccaacag<br>ugggggaauugaaagacuauga	-	cuuucugggggaauugaaagacuaugacuuaacaauuaacugauagccuuuuccuccccaacag
mmu-mir-7090	373	373	0	0	-	blast	ucucagaguugggacaccaugag<br>uuugaugucugacuuugcag	-	aguucucagaguugggacaccaugagggcuaacaggccugccuuugaugucugacuuugcag
mmu-mir-702	583	583	0	0	-	blast	ugcccacccuuuaccccgcucc<br>gugaguggggugguuggcaug	-	cgggacaaggugaguggggugguuggcauggguugcccauggggacucgacgcugugcccacagccuccugauguccuccucacgcaugcccacccuuuaccccgcucc
mmu-mir-6392	343	343	0	0	-	blast	ucuggcgcccugguccugucagc<br>gacgcagguuugccggguccucu	-	uucuuccugacccgcucuggcgcccugguccugucagcugggucaucccugagcagcagggaacgacgcagguuugccggguccucugguccccugagccgcagaaggu
mmu-mir-6911	379	379	0	0	-	blast	ugggcugagugggucuguggcau<br>ucaaucacacucuguccacag	-	uggagugggcugagugggucuguggcaucuacccccacaaagccugccucaaucacacucuguccacag
mmu-mir-141	413	370	0	43	-	blast	caucuuccagugcaguguugga<br>uaacacugucugguaaagaugg	-	ggguccaucuuccagugcaguguuggaugguugaaguaugaagcuccuaacacugucugguaaagauggccc
mmu-mir-5126	310	310	0	0	-	blast	gcgggcggggccgggggcgggg	-	gggaccggacgccuccugcagcugcgggagcccgugguuccccgggcaaccgcgggcggggccgggggcggggccca
mmu-mir-6941	315	314	0	1	-	blast	cuaacucggcugcucccaaag<br>cugggggacugacggguagcu	-	gcaccccugggggacugacggguagcugcugccgccagcuaacucggcugcucccaaag
mmu-mir-3473a	282	282	0	0	-	blast	uggagagauggcucagca	-	ccuguugagccaucucaccagcccagaaaauauuuuuaaaaagugaauugcuuaagcuggagagauggcucagcagu
mmu-mir-3069	347	317	0	30	-	blast	uuggacacuaaguacugccaca<br>uuggcagucaagauauuguuuagc	-	cuuggcagucaagauauuguuuagcagacggagcgguuucuguuggacacuaaguacugccacaa
mmu-mir-6904	281	281	0	0	-	blast	ucugaucucuuaccccag<br>uccuggggguuagaguugagugg	-	uccuuuccuggggguuagaguugaguggucguagccuguugcuucugaucucuuaccccagacaagcc
mmu-mir-544	835	327	0	508	-	blast	ucuuguuaaaaagcagagucu<br>auucugcauuuuuagcaagcuc	-	caccuagggaucuuguuaaaaagcagagucugauugaggggccaagauucugcauuuuuagcaagcucucaagugaug
mmu-mir-8111	334	252	0	82	-	blast	accgggcaugguaguguacac	-	ccaccuaccucugccucaugagugcugggauuaaaggcgugcaccaccacgcccggcgagauacuaauuuuuauacaccgggcaugguaguguacaccuuugaucauagcacucagagaaacugaggcaggugggcc
mmu-mir-1957a	344	244	0	100	-	blast	cagugguagagcauaugac	-	cuggaaaguagcucagugguagagcauaugaccagcaugugcaaggcccugaguuuauccccagca
mmu-mir-1187	239	239	0	0	-	blast	uaugugugugugauaugugugua	-	ucauauaguauuaaaccgaacuguuuuccuuauagacacaauuuauuuuuacacacacacacacacauauauauauauauguguguguguaugugugugaaauaacaauguuaaaauagua
mmu-mir-6996	239	239	0	0	-	blast	cggugucucuggucacucugcag<br>ugcacaggacagagcacaguc	-	ugggcugcacaggacagagcacagucagcaucauccgacggugucucuggucacucugcag
mmu-mir-5132	238	238	0	0	-	blast	cugauguuccccaucccgcag<br>gcguggggugguggacucagg	-	caggccggcgugggguggugggacucaggcuucuggagcugaagccugauguuccccaucccgcagcggccu
mmu-mir-5119	233	233	0	0	-	blast	caucucauccuggggcugg	-	cagggcuggccuauggggacagauacuauaagucaucucauccuggggcugggucugc
mmu-mir-3970	228	228	0	0	-	blast	gagguuguaguuugugcuuu	-	cuaggaauuugagguuguaguuugugcuuucuaucuuguagagauuucaauacucaguaugaagcagaaaccagcaucacccuuuuguuu
mmu-mir-3962	227	227	0	0	-	blast	agguaguaguuuguacauuu	-	uggguugagaaauguacucugccacguaggagcuagguaguaguuuguacauuucuuacucccu
mmu-mir-7018	225	225	0	0	-	blast	ucacccugcugccggcuugcag<br>gugagcagacagggaguggugggg	-	gugagcagacagggaguggugggggaagucuucuccugggcucccugagcccacacguaacccucacccugcugccggcuugcag
mmu-mir-5621	224	224	0	0	-	blast	aggagguccuggggccgcccuga<br>ugggcccuccagaccucaugc	-	aggagguccuggggccgcccugaaggcagaacaugagaauucugggcccuccagaccucaugc
mmu-mir-7079	213	213	0	0	-	blast	ugacuucuguucucuuuccag<br>agggcugaggcagugaguccug	-	cccggagggcugaggcagugaguccugugcacagguccuguaggcuccugacuucuguucucuuuccag
mmu-mir-3103	388	379	0	9	-	blast	ggagggaggaucugcuguuag<br>uaaccucugauccuucccacag	-	aggagggaggaucugcuguuagaaagucucugguuugugaccucuaaccucugauccuucccacagg
mmu-mir-378b	209	209	0	0	-	blast	cuggacuuggagucagaaga	-	uguccugggcuaucccaguccaggcaguacccucaggaggcagguacgauggauagagagggaacccuggacuuggagucagaagaccugag
mmu-mir-6239	199	199	0	0	-	blast	uagcguuggaucacucggug	-	uaaagcggugauguuucuggcccugggguguuagcguuggaucacucaguguuguggguguagcguuggaucacucggugcuguggcuuccaugguuccgaacag
mmu-mir-3094	226	226	0	0	-	blast	ccuuuaaauuguguccucaag<br>uguuggggacauuuuuaaagc	-	uuguuggggacauuuuuaaagcauggauugguuauugauuuguccuuuaaauuguguccucaag
mmu-mir-1291	197	197	0	0	-	blast	auggcucuuacugaagacuagcaguu	-	agaaucaagggaugggagguuacccauggcucuuacugaagacuagcaguuauacacuguagcuguuguuucaagcagacgccuagagaacugucuuccagugguuuauuagcuac
mmu-mir-6412	193	193	0	0	-	blast	ucgaaaccauccucagcuacua	-	cuaguuagcuugaugugguacugcacuccuauaaucccagggucuuggaggucuaggcaggaugauuaggaguucgaaaccauccucagcuacuaau
mmu-mir-3960	263	193	0	70	-	blast	ggcggcggcggaggcggggg	-	gccacggcuuccugcgcccccgaucggggccgccaacagcgcuggcggcggcggcggaggcgggggcaguggc
mmu-mir-6940	189	189	0	0	-	blast	agggcuggcuggaagguuuuucg<br>uuaccuuccgugcuugcccgcag	-	agaggcagggcuggcuggaagguuuuucgaggcuggcucagcaccccuuaccuuccgugcuugcccgcag
mmu-mir-7092	232	232	0	0	-	blast	aaaggcaaacucacagaaucauu<br>uguucuuuguuuuguuugccuuag	-	ugucuaaaggcaaacucacagaaucauuuucugaaaaaauaaaauguucuuuguuuuguuugccuuag
mmu-mir-5122	268	182	0	86	-	blast	ccgcgggacccggggcugug	-	guccgcuaccgcgggacccggggcugugucaggucuggaaguggagcuggagcugaccgaaggagcugccgcugggccggggguggaaca
mmu-mir-297b	2667	2667	0	0	-	blast	uauacauacacacauacccaua<br>uauacauacacacauacccaua<br>uauacauacacacauacccaua<br>auguaugugugcaugaacaugu	-	ucuauuugcuugugugugauauguaugugugcaugaacauguguauaugaauauacauauacauacacacauacccauacaaacaugcauacaaacacacagaaaaugga
mmu-mir-1195	194	173	0	21	-	blast	ugaguucgaggccagccugcuca	-	uagaggcaggcagaucucugugaguucgaggccagccugcucaacagaaugaguucuagggcagccaggaacacacagagaaacccugucucaagaag
mmu-mir-207	155	155	0	0	-	blast	gcuucuccuggcucuccucccuc	-	aaggcaggggugaggggcugcgggaggagccgggcggaggcugcggcuugcgcuucuccuggcucuccucccucucuuu
mmu-mir-7093	154	154	0	0	-	blast	caggaugacagaaggaaaaccu<br>uuuccaucugucauccugcag	-	gugggcaggaugacagaaggaaaaccuaucagauaguuuguucauaauccuucucucucaacagcacuuaguguccauguuuuccaucugucauccugcag
mmu-mir-7066	283	171	0	112	-	blast	uggguugggaaaugaguagagg<br>ucuacccauugccugccucccag	-	cccugugggguugggaaaugaguagaggcugaccucugaccucuacccauugccugccucccag
mmu-mir-7023	173	173	0	0	-	blast	augggggagcugggauugggcgu<br>ucacccugucugcgccccucag	-	uuggaaugggggagcugggauugggcguggagcuuggaggccucacccugucugcgccccucag
mmu-mir-7083	145	145	0	0	-	blast	ucggggcuggacaagcagaga<br>ucugcucuccuggaccccag	-	gagggucggggcuggacaagcagagacaugugcucaucccucugcucuccuggaccccag
mmu-mir-3473b	135	135	0	0	-	blast	gggcuggagagauggcucag	-	cugagccaucucuccagcccaagauuagguuuuaagggcuggagagauggcucag
mmu-mir-6418	132	132	0	0	-	blast	ucaggggaagggaagagaugc<br>acugcaaccuccuuucuccagg	-	agccagaagaggcaagaacacggucaggggaagggaagagaugcacuguccucuaaaaugccugacugcaaccuccuuucuccaggguaccugucaugcugccgcuuccuggacg
mmu-mir-6945	130	130	0	0	-	blast	uccugggaggggcagccccugg<br>ucugagcucugcccuucccau	-	cacguccugggaggggcagccccuggugcccacagaaccagagguucugagcucugcccuucccauag
mmu-mir-6960	124	124	0	0	-	blast	agccaaccaccucucuccucag<br>caggaugagaagaguuuggcug	-	cugggcaggaugagaagaguuuggcuggaagcccugcucagccaaccaccucucuccucag
mmu-mir-6990	122	122	0	0	-	blast	agcccugccucuuccuggcag<br>cccagggugagucagggcucu	-	gcuugcccagggugagucagggcucuccugucaucugggcccccuccgggcucugggaggccgccacuggagcccugccucuuccuggcag
mmu-mir-3110	108	108	0	0	-	blast	gcacuccaucggaggcagacac<br>uucugccuccccugaaggcuc	-	uguggggcuguguucugccuccccugaaggcucuguugucacucugggcacuccaucggaggcagacacucaaccccaca
mmu-mir-3086	126	126	0	0	-	blast	uagauuguaggcccauugga<br>cccaaugagccuacagucuaag	-	cugccauccuacacuuagauuguaggcccauuggaaggguuagcgucuugaccccaaugagccuacagucuaagugugggauggcgg

mmu-mir-3087	105	105	0	0	-	blast	uaacucacugucauguccuca<br>cagggcagggcaagaguugag	-	acagggcagggcaagaguugagacuuauggcuccuaacucacugucauguccucag
mmu-mir-466f-3	243	243	0	0	-	blast	acgugugugugcaugugcaugu<br>ugugugcaugugcauguguguau<br>uacgugugugugcaugugcaug<br>ugugugcaugugcauguguguau<br>cauacacacacacauacacac	-	caugugugguuuacgugugugugcaugugcaugugugguauaugaauauauguauacauacacacacauacacacgcacgcaacacacacacac
mmu-mir-7115	108	108	0	0	-	blast	acuuggucccugcccccacag<br>ucgggggguuguggugccgaauugu	-	gucgggggguuguggugccgaauugucugugucacccaaagccacuugguccccugcccccacag
mmu-mir-8098	95	95	0	0	-	blast	gcuauggucgcuggcucccaa	-	gccggcuugcggcuagugugggcggcgguguugguuuuggaagccagagcgggcacauucccuggacaggcggcagggcuauggucgcuggcucccaaaggcccgcuccgcuucgcagcccgauccugagcgcaagcc
mmu-mir-7679	147	147	0	0	-	blast	aggcuggcaccaggaucccu<br>caggaucccaggguaccaaccagc	-	aggcuggcaccaggaucccugauuugagauucuuacugucaggaucccaggguaccaaccagc
mmu-mir-6947	85	85	0	0	-	blast	gcagccucuuucccuggcauca<br>cauccaguggagaggcucgug	-	gggcucauccaguggagaggcucgugcacgcuccuacugacagcagccucuuucccuggcaucag
mmu-mir-3100	84	84	0	0	-	blast	uugggaacggggugucuuuggga<br>cugugacacacccgcucccag	-	uugggaacggggugucuuugggagucuugggggcuaccaaaccccugugacacacccgcucccag
mmu-mir-466m	196	195	0	1	-	blast	ugugugcaugugcauguguguau<br>ugugugcaugugcauguguguau<br>uacauacacacauacacacgca	-	ugugugugguucuugugugugcaugugcaugugugguauaugaauauacauauacauacacacauacacacgcacguacacacaca
mmu-mir-7061	96	96	0	0	-	blast	uauggaaauaggaggugauuuu<br>uaucccuuuuauuuccuuuuag	-	gaauuuauggaaauaggaggugauuuugagagaaaguugacugaaauaucccuuuuauuuccuuuuag
mmu-mir-3473e	80	80	0	0	-	blast	gggcuggagagauggcucgua	-	cucugccuucaagagacugacacugggcuggagagauggcucguaaggaagaacacuggaugcuuucucagaggacccaaggucaauuuccaguacccacaaggcagaca
mmu-mir-6915	132	132	0	0	-	blast	caacuuccuuccucgugcccag<br>aggucagagcagauggagguucgu	-	aagacaggucagagcagauggagguucgugguauuucucacccucaccaacuuccuuccucgugcccag
mmu-mir-6967-2	131	113	0	18	-	blast	ucaucuuuaucucuccccag<br>cagggagggauaaagaccagu	-	aaaggcagggagggauaaagaccagucacuauggaaacucaucuuuaucucuccccag
mmu-mir-7042	79	79	0	0	-	blast	ugucccuuuguuuucucucag<br>uagagacagcagaagggccac	-	agaauuagagacagcagaagggccacuaauuagcuguugucccuuuguuuucucucag
mmu-mir-290a	78	78	0	0	-	blast	aaagugccgccuaguuuuaagccc<br>acucaaacuauggggggcacuuu	-	cucaucuugcgguacucaaacuauggggggcacuuuuuuuuuucuuuaaaaagugccgccuaguuuuaagccccgccgguugag
mmu-mir-7043	77	77	0	0	-	blast	ugugaaagcagagaggcauuuuu<br>acugugccucucuguuuucag	-	cugccugugaaagcagagaggcauuuuucucugucgcagcuacuugaguacugugccucucuguuuucag
mmu-mir-6991	88	88	0	0	-	blast	gcagcccuuuguucuucuccag<br>uggagaacagggacugggccug	-	agcaguggagaacagggacugggccugauugauggcacucgcuccaaggcagcccuuuguucuucuccag
mmu-mir-7240	74	74	0	0	-	blast	cgccguugguccucuccaaca<br>uuggagaggaccgccgucgga	-	uuggagaggaccgccgucggacuccuccugcccugcccgccguugguccucuccaaca
mmu-mir-8117	71	71	0	0	-	blast	gcucguguggaacagaagggg	-	gggauuccugugugggcccuugcaggguguugggcgagacuccgcuggcaagguagccuggggcucguguggaacagaaggggcuugugccccaggucaggcccgggu
mmu-mir-7669	118	118	0	0	-	blast	aguaccaccauacacagcuuuug<br>gagcugggugugguggcacaugc	-	aguaccaccauacacagcuuuuguuguuguaagacgagcugggugugguggcacaugc
mmu-mir-7036b	88	88	0	0	-	blast	acgcggggguucucuguggaagc<br>uuccugguggggccucgggggucc	-	acgcgggguucucuguggaagcggcuugcucggcucugagcuuccugguggggccucgggggucc
mmu-mir-7063	86	86	0	0	-	blast	ugcucucugccccucuuaag<br>augagaggugcaggcugagcaca	-	uccccaugagaggugcaggcugagcacaaggcagcacaugggacagaugagauagugugacuccccauuugugcucucugccccucuuaag
mmu-mir-6380	66	66	0	0	-	blast	uguaagugcuuuuaacugcugagc	-	agcuucugguguuguaagcaacuauguggauacugggaaucagaccugggucccucuguaagugcuuuuaacugcugagcc
mmu-mir-6537	66	66	0	0	-	blast	uuaucagguggaagaacucagcu<br>gugaguuucucccacuggugac	-	uguccgcuucgccuuagagaagaguuuccuagcggugaguuucucccacuggugaccaggaagguuaucagguggaagaacucagcucaccaacccucucugccuguaga
mmu-mir-8092	81	65	0	16	-	blast	gccaucugggacucgcaggca	-	gcagcgcggcgcucggcuuuccggcaguacccggauggaggggguaauggccgccaucugggacucgcaggcaaugggggaccacggca
mmu-mir-1929	65	65	0	0	-	blast	uucuaggacuuuauagagcagag<br>cagcucauggagaccuaggugg	-	uuagaacugggaggucuucuaggacuuuauagagcagagguuugcagccugugacacucagcucauggagaccuagguggccuuggguucuga
mmu-mir-7117	63	63	0	0	-	blast	ucugggggcucagcugaggaua<br>uguccuucucuuuccccag	-	ugugaucugggggcucagcugaggauaagcccgccuugucaccgcuguccuucucuuuccccag
mmu-mir-6998	96	96	0	0	-	blast	agagcugcucugugcccacaca<br>cugggcagagggcaaagugacu	-	ugggccugggcagagggcaaagugacucggugacacgggugagagcugcucugugcccacacag
mmu-mir-1953	63	63	0	0	-	blast	ugggaaaguucucaggcuucug	-	ggugcugccuggucuugggaaaguucucaggcuucugagaauuugcaggagcagaaggcugugagguuccccucuuccuggcagcccua
mmu-mir-7024	73	73	0	0	-	blast	uugggggaugggguugcuuggc<br>ccagcagucccugcuccuacag	-	augggguugggggaugggguugcuuggcccuaggcaccugcccagcagucccugcuccuacag
mmu-mir-3473g	62	62	0	0	-	blast	caaaagugaggcuggggaga	-	cucugcaaguggagcaagugcucaccaccgagccaucucuccaaccuucauauuuguauuuguuagacaagaaguacaaaagugaggcuggggagaggguuugcagugugguauugcucuucuaaaggaccacaguu
mmu-mir-6932	86	86	0	0	-	blast	uuguaggugaaguagacuuggc<br>ucuguucugcuuccucugcag	-	agucuuuguaggugaaguagacuuggcugacagaugcucuguucugcuuccucugcag
mmu-mir-6994	75	75	0	0	-	blast	aacgaucuucuccgucuuugc<br>acaaaggugggugaagccgguga	-	cugucacaaaggugggugaagccggugacgauugcuaguucuucugugucaacgaucuucuccgucuuugcag
mmu-mir-3077	59	59	0	0	-	blast	cugcggacgggugggcgggcaggcc<br>cugacucccugcuucuccgcag	-	cugcggacgggugggcgggcaggccaaauggcgcgcccgaggcugacucccugcuucuccgcag
mmu-mir-712	608	59	0	549	-	blast	cuccuucacccgggcgguacc<br>ugcgagucacccccggguguug	-	ucuccgcuucuccuucacccgggcgguacccgcuccggcgccggcccgcgggacgccgcggcguccgugcgccgaugcgagucacccccggguguugcgaguucgggga
mmu-mir-6951	58	58	0	0	-	blast	cuuuuuucuucacaaauacag<br>uuguauuugugugauuaaagu	-	uuucauuguauuugugugauuaaaguauuagaaguauugaaauucugaguuugcuuuuuucuucacaaauacag
mmu-mir-669k	55	55	0	0	-	blast	uaugcauauacacgcaugcaa<br>ugugcauguguguauaguugugugc	-	ucuuaugcccaccaaaaaugugcaugugugguauaguugugugcauauucacuucucuauaugaauaugcauauacacgcaugcaaacacauucauaggaauggccagagcuauaucucaguuaggag
mmu-mir-7013	76	76	0	0	-	blast	uaugaagaggccaguguuguag<br>ccacacuuacuguugccucuuccu	-	uucgguaugaagaggccaguguuguagcuugagauccagccuaagccacacuuacuguugccucuuccucag
mmu-mir-3091	50	50	0	0	-	blast	caugggucugguugggcccgc<br>cgggccugaccagucucaagac	-	ugcuugcgggccaugggucugguugggcccgcagacucggagugcgggccugaccagucucaagaccugcguggua
mmu-mir-6901	49	49	0	0	-	blast	ugcaggaaugcagaggaccu<br>gaccuucuguguucuugcag	-	cuaagugcaggaaugcagaggaccucguggugaaaggaccuucuguguucuugcagcucccu
mmu-mir-7118	49	49	0	0	-	blast	uggggaaggcgggagagggaac<br>uaacucuacguccucccucaca	-	gggucuggggaaggcgggagagggaacagaacaacagcuaacucuacguccucccucccacag
mmu-mir-547	49	49	0	0	-	blast	cuugguacaucuuugagugag<br>cacuugaggauguaccaccca	-	gugugauguaucacuugaggauguaccacccauuuaacaggaaacaugcuugguacaucuuugagugagauaacacac
mmu-mir-7032	59	49	0	10	-	blast	auccucucgguaccgcccugca<br>cccaggguggucccccgagagggugggu	-	cgugggcccaggguggucccccgagagggugguccccacaugggcaaaccaggggguccauccucucgguaccgcccugcag
mmu-mir-6900	49	49	0	0	-	blast	ugccaggagaagccuagagccguc<br>uggugaugggcucucuuguag	-	ucaugugccaggagaagccuagagccgucuggacucugaugguggaugggcucucuuguag
mmu-mir-881	48	48	0	0	-	blast	aacugugucuuuucugaauaga<br>cagagagauaacagucacaucu	-	ugcaguacaauauucagagagauaacagucacaucuuuucuaaaguaacugugucuuuucugaauagaguaauguuca
mmu-mir-7014	48	48	0	0	-	blast	ccucuuucauaccaaccacag<br>uugggugcuguggaagggacag	-	aggcuuugggugcuguggaagggacagagacccugagcucuuccucuuucauaccaaccacag
mmu-mir-1668	47	47	0	0	-	blast	caaaggccuccuucucugcag	-	acacggguaaggggcuggccugcaguggggcugggagggcaggccuaggugagcucagccucaaaggccuccuucucugcaggcucucagguccucagaaucaggga
mmu-mir-6942	82	82	0	0	-	blast	uaggaggaggggaacauuugc<br>uuaugguucucuccaccucuca	-	gggccuaggaggaggggaacauuugcaacuauaguggcuuaugguucucuccaccucucag
mmu-mir-7666	105	45	0	60	-	blast	guagcccgggcggugcuucccc<br>gaugcagcgcacgggcgag	-	guagcccgggcggugcuuccccgagagucgccgugggaugcagcgcacgggcgag
mmu-mir-688	43	43	0	0	-	blast	ucgcaggcgacuacuuauuc	-	gaaagggcagugaagaaaaguaggggcuugcuuggggagagcaccucgcaggcgacuacuuauuccagcucgccc
mmu-let-7k	42	42	0	0	-	blast	ugagguaggagguugugug	-	uagccacagcccuaacccuagccugagguaggagguugugugcaagcucaccacuaaccuauaguacacagagagccuuuaucccacaacacaacacaaaacaauacuuc
mmu-mir-3070-1	54	54	0	0	-	blast	agccccugaccuugaaccuggga<br>uggugcuaccgucaggggguaga	-	gugcugagugaggcugagccccugaccuugaaccugggauccuauccaugaucuccuggugcuaccgucaggggguagauuccuugucau
mmu-mir-5124a	101	41	0	60	-	blast	gguccagugacuaagagcau	-	uagaaaggugggguccagugacuaagagcauuugagcacuuguucuugcagaggacccaaguucaauuucuag
mmu-mir-7059	41	41	0	0	-	blast	accggggacugcggcuuucagu<br>uguuugcuguuuguccucccag	-	aaaccaccggggacugcggcuuucaguuaaaggucacuguuugcuguuuguccucccag
mmu-mir-3473d	130	39	0	91	-	blast	ccacugagccacuuuccagcccuu	-	ugugcuuaaccacugagccacuuuccagcccuuugaaaacuuauuugagggcuggagagguggcucaguggcuaagagcac
mmu-mir-7659	38	38	0	0	-	blast	aggagucagcuugucgguucaug<br>aggagccggcuccagauuccug	-	aggagccggcuccagauuccuguguagcugccuccaggagucagcuugucgguucaug
mmu-mir-7089	65	65	0	0	-	blast	ccgugggaccuacagacuccuggg<br>ucagugucugcugucuccacag	-	cugggccgugggaccuacagacuccuggguguggcuuagcaggugcucagugucugcugucuccacag
mmu-mir-7656	37	37	0	0	-	blast	ugugggaucagauagccuguua<br>acaggcugucugaucccacggu	-	ugugggaucagauagccuguuaguguuauccuacacugguacaggcugucugaucccacggu
mmu-mir-7070	37	37	0	0	-	blast	caggcucuccuucccuccag<br>gugaggggagcugaggcagga	-	gugaggggagcugaggcaggaggccaagcaggcuaggaggugaggaaccucaaggauaacuaacuccaggcucuccuucccuccag
mmu-mir-6922	36	36	0	0	-	blast	uguucugcucacccuucucacag<br>ugugggaggggacuguagagagg	-	auuggugugggaggggacuguagagaggagggugccuaaccucuguucugcucacccuucucacag
mmu-mir-3113	37	37	0	0	-	blast	guccuggcccugguccggguccc<br>uccuggcccugguccugguc	-	ggcccuggcccggguccuggcccugguccggguccuggcccugguccuggcccugguccugguccuggcccuggcc
mmu-mir-201	35	35	0	0	-	blast	ugaacagugccuuucuguguagg<br>uacucaguaaggcauuguucuu	-	uaccuuacucaguaaggcauuguucuucuauauuaauaaaugaacagugccuuucuguguagggua
mmu-mir-6917	34	34	0	0	-	blast	gucacuucucuucccaccacag<br>uguggaaggggaguugugccg	-	gaagguguggaaggggaguugugccgcagucuucagccugucacuucucuucccaccacag
mmu-mir-6910	33	33	0	0	-	blast	ucaugguuuccacccuuugucc<br>ugggguagggcaccagugggca	-	gcaccugggggguagggcaccagugggcagagugucccugcugcucaugguuuccacccuuuguccag
mmu-mir-147	52	52	0	0	-	blast	gugugcggaaaugcuucugcua<br>uggaaacauuucugcacaaacuag	-	uaugaaucuaguggaaacauuucugcacaaacuagauguugaugccagugugcggaaaugcuucugcuacauuuguagg
mmu-mir-696	73	31	0	42	-	blast	gcgugugcuugcuguggg	-	ggucugucgcggugccugaagcugucccccgagccacgcuuccugcuuucccgggcuugcugcuugcgugugcuugcugugggcagcuu
mmu-mir-7219	31	31	0	0	-	blast	uauccggguuucuaacacacu	-	uguguuagagcucaggguugagaucaugugaucuauccggguuucuaacacacu

							uguguuagagcucaggguugaga		
mmu-mir-29b-1	344	344	0	0	-	blast	uagcaccauuugaaaucaguguu<br>gcugguuucauaugguguuua	-	aggaagcugguuucauaugguggguuuagauuuaaauagugauugucuagcaccauuugaaaucaguguucu
mmu-mir-6906	30	30	0	0	-	blast	gaauuccggucuccucuccag<br>auggaugggcagacggacagacag	-	gauggaugggcagacggacagacagcagagccuaccugaauuccggucuccucuccag
mmu-mir-196b	29	29	0	0	-	blast	ucgacagcacgacacugccuuc<br>uagguaguuuccuguuguuggg	-	aacuggucggugauuuagguaguuuccuguuguugggauccaccuuucucucgacagcacgacacugccuucauuacuucaguug
mmu-mir-1951	28	28	0	0	-	blast	guaguggagacuggugugggcua	-	uggguggggguuuagaaaccaccucucuucacuuacuucugaaaguuaaaagguaguggagacuggugugggcuaaaccucuugccuccc
mmu-mir-7243	27	27	0	0	-	blast	ucagaacagcugacagcagu<br>ugcugucagcuguucugagu	-	ugcugucagcuguucugagugcuaaaagcaacucagaacagcugacagcagu
mmu-mir-7116	43	43	0	0	-	blast	ugaagacaucaggaaaaaaaa<br>uuuuuuuccuuugccuucucag	-	aacagugaagacaucaggaaaaaaaaaauaagaaaugauaaaccuuuuuuuuuuuccuuugccuucucag
mmu-mir-1191b	26	26	0	0	-	blast	agacucacuauguagcccaagc<br>ucaggcuacagagcgagaaccu	-	ucaggcuacagagcgagaaccugcuucaaaaacaccagacucacuauguagcccaagc
mmu-mir-7065	26	26	0	0	-	blast	uugguuuuccuguuugcacag<br>aguguagcagguaaaccagga	-	cauggaguguagcagguaaaccaggaaucucucucaggucuugguuuuccuguuugcacag
mmu-mir-6971	26	26	0	0	-	blast	acagccucugcuucuucucag<br>ugggggaggguguagaggcu	-	ugggguggggggagggugagaggcugggcuggcagacucaucacagccucugcuucuucucag
mmu-mir-6950	26	26	0	0	-	blast	uccuggaguggaggacagucaga<br>cucugucuugauccucuccag	-	cucuguccuggaguggaggacagucagaaaggcaguuguagcauucucugucuugauccucuccagguuguca
mmu-mir-3569	26	26	0	0	-	blast	ucagucugcgcuccucuccagc<br>ucggaggagagcagaccggug	-	ucggaggagagcagaccgguggucgaaauccggaccucagucugcgcuccucuccagc
mmu-mir-7071	38	38	0	0	-	blast	cccgggagcgaggugcugcagcg<br>cugacgucucgguucccgcag	-	gggggcccgggagcgaggugcugcagcgcgcgugcucacuacgcgacucugacgucucgguucccgcag
mmu-mir-5709	25	25	0	0	-	blast	aaagucuuaagggugugguauug<br>auacgcacacuuaagacuuuag	-	aaguugauaggccggcaauacgcacacuuaagacuuuagaagcucucagacuaaagucuuaagggugugguauugcaggccuagcaaugc
mmu-mir-6985	39	25	0	14	-	blast	cucacagccaccuccucacag<br>uacugaggggguggcugcuaugu	-	guguauacugaggggguggcugcuaugucacucaaagcugacucacagccaccuccucacag
mmu-mir-7004	25	25	0	0	-	blast	uuccguggaugaggccugugccgu<br>cccacucccuccuucacgcag	-	ugaccuuccguggaugaggccugugccgugacucucagugacacccacucccuccuucacgcag
mmu-mir-706	25	25	0	0	-	blast	agagaaacccugucucaaaaaa	-	agagaaacccugucucaaaaaacaaaaaaacaaacaaaauuacuuuuaguagguuuauuuuguuuuuugagauggcuuuuuuuu
mmu-mir-465a	199	199	0	0	-	blast	gaucagggccuuucuaaguaga<br>gaucagggccuuucuaaguaga<br>uauuuagaauggcacugauguga<br>gaucagggccuuucuaaguaga	-	gcccuauuuagaauggcacugaugugauaaaauaaaaaauugaucagggccuuucuaaguagaguaaggcuuac
mmu-mir-6925	43	43	0	0	-	blast	ugaggaagagaaggcuuggacu<br>ucccuuuuaucccucag	-	aagccugaggaagagaaggcuuggacugacucauccugauuaccgucccccuuuuaucccucagcaaucaa
mmu-mir-219b	25	25	0	0	-	blast	agauguccagccacaauucucg<br>agaauugcguuuggacaaucagu	-	agauguccagccacaauucucgguuggccgcagacucguacaagaauugcguuuggacaaucagu
mmu-mir-6928	24	24	0	0	-	blast	aggggauuugcauggacugcacu<br>aagcuguuccuguacucuccag	-	uuccaaggggauuugcauggacugcacuaagacacuucaguguuccuaaagcuguuccuguacucuccag
mmu-mir-6923	24	24	0	0	-	blast	gugagggcaggaggauuggggugu<br>acacucccuccuccucccccag	-	gugagggcaggaggauuggggugugagggguccaccccagacgcacuguaugacacucccuccuccucccccag
mmu-mir-8094	24	24	0	0	-	blast	aacugaaggacaacgagaaga	-	cacggguugcagcaguacuacgugaaacugaaggacaacgagaagaaccggaagcucuuugaucuucucgauguccucgaguucaaccaggucaguaucgagggucuguguaggcga
mmu-mir-6955	24	24	0	0	-	blast	acaccugucuccuuugcccaca<br>agggccucaggacucaggagguga	-	aagggagggccucaggacucaggaggugacccuaacacuuuggagagucucacaccugucuccuuugcccacag
mmu-mir-7003	23	23	0	0	-	blast	uguggggagaagcugcggcag<br>ccccggguuuuccccacag	-	ucaggugugggggagaagcugcggcagccgagagcugcgugucagaggccccggguuuuccccacag
mmu-mir-3090	23	23	0	0	-	blast	ucccaggugacacccugacuca<br>gucuggguggggccugagauc	-	uuccugucccagguggucuggguggggccugagauccugaauuucuaaugagcucccaggugacacccugacucacugugccagagga
mmu-mir-7017	23	23	0	0	-	blast	agaggguugugagacuagggcugu<br>acccugcuccucucccuccag	-	gucccagaggguugugagacuagggcugugcuuccugccuaacccugcuccucucccuccag
mmu-mir-3112	23	23	0	0	-	blast	caguuugucucuucuuugucu<br>acauagaaaaggcagucugca	-	cuaaaugaacagugacauagaaaaggcagucugcauuuauucuggagcugcaguuugucucuucuuugucucugucaguugg
mmu-mir-344i	58	22	0	36	-	blast	aagucaggcuccuggcugga	-	gggauuuugcaagucaggcuccuggcuggaguccagcucucagcuggguccaggcucuagccagggucugacuacaugaugcucagua
mmu-mir-741	22	22	0	0	-	blast	ugagagaugccauucuauguaga<br>uacguagauugguaccuaucaug	-	uugaucuacguagauugguaccuaucauguaaaucauguaagcaugagagaugccauucuauguagauuaa
mmu-mir-6963	22	22	0	0	-	blast	ggugggauggauggcagaaccugg<br>ugccucuugccuccaucccacag	-	gggaaggugggauggauggcagaaccuggagauucuugucaaacugcuugccucuugccuccaucccacag
mmu-mir-878	22	22	0	0	-	blast	gcaugacaccacacuggguaga<br>uaucuaguuggaugucaagaca	-	ugcaaugcuuuaucuaguuggaugucaagacacgugaaacuuaagugcaugacaccacacuggguagaggagggcuca
mmu-mir-7049	22	22	0	0	-	blast	ugcagccccugucugccucag<br>ucugaaggcagucagguaggcgg	-	gugugucugaaggcagucagguaggcggcaccucugaccugcagccccugucugccucag
mmu-mir-6914	36	36	0	0	-	blast	uccuggggugguggugggccacaga<br>ucuguccaccucuucccag	-	cugacuccuggggugguggugggccacagaaacgaggucuuuaacucuguccaccucuucccaggcgcgg
mmu-mir-7038	33	33	0	0	-	blast	uguagaaggaagggcugcugug<br>cacugcuccugccuucuuacag	-	ugcgguguagaaggaagggcugcugugcuugacaagucugucauuuucacacugcuccugccuucuuacag
mmu-mir-703	229	21	0	208	-	blast	aaaaccuucagaaggaaagaa	-	agacugguuaaugauaacaaugcaucauaaaaccuucagaaggaaagaaugauguggaccauuuuuuuuuguguguggcaguuuuaaguuauuaguuuucaaaaucagu
mmu-mir-7047	33	33	0	0	-	blast	ugagggaggagggcugggucuga<br>ucacccucacccuuccuccuucc	-	uggucugagggaggagggcugggucugaagaguugguucucacccucacccuuccuccuuccag
mmu-mir-6539	21	21	0	0	-	blast	gcacagugaugaacucugagggcu	-	acacccucccccaggacaguaaagcacagugaugaacucugagggcuuuucacuucccucucggaguugcugguugcucacugggucugugaaugugaauccaugugggca
mmu-mir-6938	21	21	0	0	-	blast	uuggagcuagcuccagagagagu<br>ucaucuggggcugucuccuuag	-	aguguuuggagcuagcuccagagagagugaagucaugcuugucaucuggggcugucuccuuag
mmu-mir-7057	21	21	0	0	-	blast	uaauuugaugucuuucuccacc<br>caggagaggacucaaguu	-	gccugcaggagaggacucaaguuggaaaacguucuaauuugaugucuuucuccaccag
mmu-mir-7085	34	34	0	0	-	blast	caguggggggcaggccagcagga<br>uagcuggccucuccccaccuuc	-	uggugcaguggggggcaggccagcaggagcagagcccaaguccuagcuggccucuccccaccuucag
mmu-mir-28b	20	20	0	0	-	blast	aggagcucacaaucuauuuag	-	gacuauagaaaaggagcucacaaucuauuuaguggcaggcgcacacugccagaaugugugaggcaucuuaauagugugcu
mmu-mir-6995	20	20	0	0	-	blast	cugggaguagaagggggaaacca<br>ugugucccccuuccucucacag	-	cagaccugggaguagaagggggaaaccagaaaugagggcuuuaaugauggugugucccccuuccucucacag
mmu-mir-7648	46	37	0	9	-	blast	agggcugggcccgggacgcgg<br>ccgcguuccgggcucggcgc	-	ccgcguuccgggcucggcgcuccgggcugcccagggcugggcccgggacgcgg
mmu-mir-7075	19	19	0	0	-	blast	caaccaugucuucuuucccag<br>cggggaggaggacaugguuuu	-	ggccgcggggaggaggacaugguuuugggggggcaugacuuggggguccggggcucgcggagccaaccaugucuucuuucccag
mmu-mir-6968	18	18	0	0	-	blast	acaagcgcugucucccccuccag<br>agggggcgagggggcccugugg	-	agggggcgagggggcccugugguuucaagaaggcugucugagggccacaagcgcugucucccccuccag
mmu-mir-5617	18	18	0	0	-	blast	caggcggccucagcucucacu<br>guaagugagggcaagccuucugg	-	guaagugagggcaagccuucuggguuccuaccaucccaggcggccucagcucucacu
mmu-mir-6937	18	18	0	0	-	blast	ucacagguucccccuugcacuag<br>uagcuguaagggcugggucugugu	-	uagcuguaagggcugggucugugugaucggucuccaggaaacucacagguucccccuugcacuag
mmu-mir-8106	37	18	0	19	-	blast	ugacucuguacauggcauuuau	-	gaagacagucuuguuggaaaagcagcuuuacaaagugaaacauaaaucccaugugcagagucacugaguagcugaggugacucuguacauggcauuuauguuuuacauuucugugauuucucaucgagcagcugaggug
mmu-mir-7062	18	18	0	0	-	blast	acuaacuucuccuggccccacag<br>uggaggccagcuuguggagugucu	-	uaggauggaggccagcuuguggagugucuaugaccuaaaccagacuaacuucuccuggccccacag
mmu-mir-6988	18	18	0	0	-	blast	ggggugggagagcugaggccca<br>ugaccucuguaucuccugccag	-	aucuggggugggagagcugaggcccagguagcucagccugaccucuguaucuccugccag
mmu-mir-7002	17	17	0	0	-	blast	uuggcuucggggaguacgugg<br>uugugcuuccccuugccag	-	ucuuuggcuucggggaguacguggaaaaugagcgcuuugugcuuccccuugccag
mmu-mir-1946a	16	16	0	0	-	blast	agccgggcaguggguggcacacacuuuu	-	aacuuaaagaaaucuauagccgggcaguggguggcacacacuuuuaaucccaacacuugugaggcacaggcagguggauuucugaucaaaggcaugcgccaccacucucggcuuucucccucuccuuuuuuguuaa
mmu-mir-470	16	16	0	0	-	blast	aaccaguaccuuucugagaaga<br>uucuuggacuggcacuggugagu	-	cagugcucuucuuggacuggcacuggugaguuaaacuaaauacaaccaguaccuuucugagaagaguaaagcuca
mmu-mir-466f-4	32	32	0	0	-	blast	uacgugugugugcaugugcaug<br>acgugugugugcaugugcaugu	-	caucugcaucugugugucuacgugugugugcaugugcaugugugguaucuguaugugagugugugcaugcaugugugugcaucuauaucugugugucuacuugugugugaaugugcauau
mmu-mir-3065	16	16	0	0	-	blast	ucagcaccaggauauuguugggg<br>ucaacaaaaucacugaugcugg	-	gcagcugcccucuucaacaaaaucacugaugcuggagucacugugcacccacucagcaccaggauauuguuggggaggacggccugu
mmu-mir-6912	16	16	0	0	-	blast	cuaccugagggucuccuuccugcag<br>uacagggagggugcucaggcag	-	ugaaguacagggagggugcucaggcaggcaucuggcuguagcaggcuaccugagggucuccuuccugcag
mmu-mir-6913	15	15	0	0	-	blast	ucucuacugauuugucuccucag<br>ugggagaccagucagguguugg	-	ugaccugggagaccagucagguguugggcaucaagcaagcucccucucuacugauuugucuccucag
mmu-mir-8102	36	15	0	21	-	blast	ucacgcgggggaacgaggaaga	-	gguaccgcccguccccgggcgcuugcagcugccccgcccacuccuccucucccggccugaagggaacccagccggcuucacgcgggggaacgaggaagacuggggauggacagccucgaggcacucggcaagaagggcu
mmu-mir-692-3	46	15	0	31	-	blast	aucucuuugagcgccucacuc	-	gguggcagggccacaaccagcgcagacuggcgcgccccagggaucucugggcgaguaucucuuugagcgccucacucucaagcacgacuaggaggccucugugccuucc
mmu-mir-6898	15	15	0	0	-	blast	uguaaggggagaugcagggagc<br>aagucucuuuguaccuucucu	-	aucccuguaaggggagaugcagggagccaugccgaaugaugcuuggauucaagucucuuuguaccuucucuacccag
mmu-mir-466i	973	441	0	67	-	blast	auacacacacacauacacacua<br>ugugugugugugugugugug	-	agagaucaugucagugaacugccacauuuuugauauauauuauguauguaugugugugugugugugugugugugugugguauauauauacacacacacauacacacuacauguaugcaacauauauac
mmu-mir-1960	14	14	0	0	-	blast	ccagugcuguuagaagagggcu	-	cagagugagcagccagugcuguuagaagagggcugagagcagggagagcucucuucugcagcacugacuuuuccucug
mmu-mir-7039	14	14	0	0	-	blast	ccuguucugcccucccuuccag<br>agagggauggagagcaucgcgu	-	ucguuagagggauggagagcaucgcguguuugagucuugugacacuuccauaauguuuccacgccuguucugcccucccuuccag
mmu-mir-208a	14	14	0	0	-	blast	auaagacgagcaaaaagcuugu<br>gagcuuuuggcccggguuauac	-	uuccuuugacgggugagcuuuuggcccggguuauaccugacacucacguauaagacgagcaaaaagcuuguuggucagaggag
mmu-mir-3620	14	14	0	0	-	blast	cugcucagccccgcccacccca<br>cugugggcugggcugggaagca	-	cugugggcugggcugggaagcagcuuucaucccagccuccugcucagccccgcccacccca

mmu-mir-7029	13	13	0	0	-	blast	uguggcucugugugucagauc<br>ucuggacgcucggcugccagcgc	-	guaagucuggacgcucggcugccagcgcccugagcuguggcucuguguguccagaucagcu
mmu-mir-5110	13	13	0	0	-	blast	ggaggagguagaggguguggaauu	-	agcggaggagguagaggguguggaauuugaugucaucucugccacuacuggacagggcuacguuguucuagccacuugcccugagu
mmu-mir-5624	13	13	0	0	-	blast	uauuguaaacucugccuu<br>uuaaggcagaguuuacaauagg	-	uauuguaaacucugccuuaauuuaguuagguuauucuaauuaaggcagaguuuacaauagg
mmu-mir-1199	12	12	0	0	-	blast	ucugagucccggucgcgcgg<br>ugcggccggugcucagucggc	-	agccugcgccggagccggggucugagucccggucgcgcgggcgaggaacucauugaguugcgcgugcggccggugcucagucggcccggcuccgguacuccgcugccgcgcgcccugga
mmu-mir-698	12	12	0	0	-	blast	cauucucguuuccuucccu<br>uguggguggacagggauguu	-	ucaucucugcccucacuguaagggaggguggugggcuaugugggugggacagggauguucaguugcuaaagacauucucguuuccuucccucagugucccccagaugguga
mmu-mir-6965	12	12	0	0	-	blast	agagggaggcuauugggcaggaga<br>ucugugcucggucucgcuccag	-	gcgggagagggaggcuauugggcaggagaugaggaagaaccuucugugcucggucucgcuccag
mmu-mir-6909	12	12	0	0	-	blast	ugccuuccccggccucccacag<br>cagggggcuggaaugggcacu	-	gauaacagggggcuggaaugggcacugcggagaaccaugugccuuccccggccucccacag
mmu-mir-7653	12	12	0	0	-	blast	ccccuacuguuccaccccgccu<br>uaaggggcggacagacagacgg	-	uaaggggcggacagacagacggcaggaagggucagcaugccccuacuguuccaccccgccu
mmu-mir-7674	11	11	0	0	-	blast	ucccauucucaccgcacccag<br>ugaggugugggcagcaugaggacu	-	ugaggugugggcagcaugaggacuagggccaugacaacccugucccauucucaccgcacccag
mmu-mir-465b-2	185	185	0	0	-	blast	gaucagggccuuucuaaguaga<br>uauuuagaauggugcugaucug<br>gaucagggccuuucuaaguaga<br>gaucagggccuuucuaaguaga	-	ugcaaugcccuauuuagaauggugcugaucugaugaaauaaaaaauugaucagggccuuucuaaguagaguaaggcuua
mmu-mir-7067	21	21	0	0	-	blast	ugcagggggugguugcacacgga<br>ugauugcuucccuccccacag	-	agggcugcagggggugguugcacacggacagucucuggacacugauugcuucccuccccacag
mmu-mir-743b	11	11	0	0	-	blast	uguucagacugguguccauca<br>gaaagacaucaugcugaauaga	-	ugcagugcuguguucagacugguguccaucaugugaaauauuugugaaagacaucaugcugaauagaguaaggccca
mmu-mir-7041	10	10	0	0	-	blast	ugguucucuucuucccucag<br>agagggaauggagacucaguuga	-	cucugagagggaauggagacucaguugaccucugguucucuucuucccucag
mmu-mir-6903	10	10	0	0	-	blast	uugggaaaagcaguacuuacca<br>ugugugucuguuuuucccag	-	cccuuuugggaaaagcaguacuuaccacaugucuucagguggcacgcguguguucacugucgugugugucuguuuucccagguauca
mmu-mir-1895	10	10	0	0	-	blast	cccccgaggaggacgaggagga	-	cccacaccuuuuccucuucuucuuggccggguuggcuccgccagcuccccccgaggaggacgaggaggaggaggaauga
mmu-mir-7027	9	9	0	0	-	blast	uggaaaggaagaaacagcagagc<br>ucggguuucucuccucuuccag	-	uccccuggaaaggaagaaacagcagagcagcugggguccccuacaugaagcucggguuucucuccucuuccag
mmu-mir-8118	17	9	0	8	-	blast	gacaaacaugacuaugcugaca	-	acaagcuaaaacaaguccccauuccugugucggcauagucauguuugugcuuugauaaacaacagggaguaggacaaacaugacuaugcugacacuagaaugcuugcagggaccagaggaaagcaucag
mmu-mir-6769b	9	9	0	0	-	blast	ccuggugggugggggaagagc<br>caucuucccugucccacccag	-	ugcuccuggugggugggggaagagccuaguucacaucaggcaucuucccugucccacccag
mmu-mir-1893	32	8	0	24	-	blast	ggcgcgggcgcuggacgccucg	-	ccagagcggagagguaucugcugcgccugagaugaguaaacugucguuucgggcgcgggcgcuggacgccucgaagccgcugc
mmu-mir-3088	8	8	0	0	-	blast	gcuaugcaguuguucaugagcu<br>uucaugagcagcugcaaaggugu	-	cagugccugcuaugcaguuguucaugagcucuggcuccgaagguucaugagcagcugcaaagguguugcugcug
mmu-mir-5114	7	7	0	0	-	blast	acuggagacggaagcugcaaga	-	acuggagacggaagcugcaagagcggcugagugugggggucuugcaguuccuguuccagaag
mmu-mir-467h	7	7	0	0	-	blast	auaagugugugcauguauaugu	-	uuuucacuuuuguuuuucucauguauauguguaugugugcgguguacauaauuauaucuaugugugcauguauguggacauaagugugugcauguauaugugugugugugguguacauaau
mmu-mir-7010	7	7	0	0	-	blast	agguucuccuuuccuuugcag<br>cggaaaggauugggaaacuuga	-	guuucggaaaggauugggaaacuugacuaaugcagguaacagguucuccuuuccuuugcagg
mmu-mir-767	7	7	0	0	-	blast	ugcaccaugguugucugagca	-	agaauccauauagguuuuuacucaugcaccaugguugucugagcacauaaacaugcuugucugcucauacccuaugguuccugaagaggaaucuuaacugucuacugc
mmu-mir-3471-2	6	6	0	0	-	blast	ugagauccaacuguaaggcauu	-	agaaaugccuccaugagauccaacuguaaggcauuuucccaauuagugauaaaggggaaaaggccccuuguggguggggacaaucucugggcugguagucuug
mmu-mir-7022	6	6	0	0	-	blast	acaagccugaccucugcccca<br>ccuggcaaggaagggcucuguuu	-	accuggcaaggaagggcucuguuuagcaugcuggcaauacaagccugaccucugcccccag
mmu-mir-6481	6	6	0	0	-	blast	cacugaaaaugcuuagaug	-	uugcccaaacuaaacuaauaggauuuaccuauuaacaaacucagguaauguaguguuaaaaagcagagcacugaaaaugcuuagaugggcacuuaucccauaaaaauaaa
mmu-mir-7078	4	4	0	0	-	blast	ugugggugguaggagacgcu<br>uacuuuuuuuaucauccacag	-	cagggugugggugguaggagacgcuggcugguauuugaccagguacuuuuuuuaucauccacag
mmu-mir-7235	4	4	0	0	-	blast	ggagggaggggucugggc<br>ucugacuucuugcuucucuccuc	-	ggagggaggggucugggcagaagugaagcuggcucugacuucuugcuucucuccuc
mmu-mir-1971	4	4	0	0	-	blast	guaaaggcugggcugaga	-	gcaucauuugguaaaggcugggcugagaggacaucuuuguuccauggacccaguagauaggcagcuucugcuuccaggaacaacuugucuguuuuucagucuaucu
mmu-mir-3966	3	3	0	0	-	blast	agcugccagcuguagaacugu	-	aacacaggaaaagacagcugccagcuguagaacuguuuuuaauuaaaacuguguucaucaugcuugcugcagaguuuucuuauuu
mmu-mir-669n	3	3	0	0	-	blast	auuugugugugugaugugugu	-	uacauuugugugugugaugugugugugaaauguaugugcaucccacagcacaugug
mmu-mir-1954	106	3	0	103	-	blast	acugcagagugagacccuguu	-	gaggucagccagaacugcagagugagacccuguuguuuguuuguuuguuugagacgggguuucucuguguagcccuggcuauccua
mmu-mir-8108	3	3	0	0	-	blast	ucuggggaggagcguaguuaca	-	acucaugcauguguucucuacauucugccgcuuuuccccugaggcauauccagauaagacccucuggggaggagcguaguuacagaaagaaauguaggcucuuauuuug
mmu-mir-7091	3	3	0	0	-	blast	aguggcuucugucgucucuag<br>guaggggugauagauuccauggc	-	guaggggugauagauuccauggcagaggggauggugcaaaugccuuuuagcaagacgcaguggcuucugucgucucuag
mmu-mir-6974	2	2	0	0	-	blast	ucuccacucucuucugucccag<br>gugaggcagcaagagauugggggu	-	gugaggcagcaagagauugggggugcugggguggccuagcuaacucagaguccuagaguccucuccacucucuucugucccag
mmu-mir-5710	2	2	0	0	-	blast	ucuugggacauaguguaaggca	-	agagucuugggacauaguguaaggcaguggucuggcuagucccacuaacuggcucuuugucccucggca
mmu-mir-467g	2	2	0	0	-	blast	uauacauacacacacauauau	-	gucuugugcaugcauauauauauaugugugugugugguauauauauauacauauauauacauacauacacauguauauguauauauacauacacacacauauauauauucauguauauauaua
mmu-mir-3547	1	1	0	0	-	blast	gugggaagaggggugggggcccggga<br>ugagcaccaccccucucucag	-	gugggaagaggggugggggcccgggacuguacccaugugaggacuauucuugaguccugcucuuguucugagcaccaccccucucucag
mmu-mir-3969	1	1	0	0	-	blast	cccuaaaguagaaaucacua	-	aaauggauuuccccuaaaguagaaaucacuauagaguugcaugguuacagaggacuagaauuuagauuagcuacuuacagggcuucagaucu
mmu-mir-195b	1	1	0	0	-	blast	uagcagcacagaaauaguagaa	-	agaaaggacuuaaauagcagcacagaaauaguagaaaacacagcccaacagcuuuggcuugaauuacugcucuccuguaacugauauagucuuuuuc
mmu-mir-1961	1	1	0	0	-	blast	ugagguaguaguuagaa	-	gcuagccauaucugaaguucaacacgcccucccccucuuccuauacgucuauggaggauuuaccuccauauaccuugagagaugagguaguaguuagaacuuucauggagcaa
mmu-mir-6395	1	1	0	0	-	blast	cuggcccucucugcccuguuua	-	agcuggcccucucugcccuguuuaaagagauuugucuuuucucagauagauagaugucuuuauauaguccagacaugagucuucugucagca
mmu-mir-682	82	0	0	82	-	blast	cugcagucacagugaagucug	-	gccgucggcaucuggcacugugguuccugcaugaaaacaguggccggcggggccuggaccuacaacaccaccucugcagucacagugaagucugcc
mmu-mir-7238	226	0	0	226	-	blast	cuguccuuuguguuc<br>augcggaugggacgcggc	-	augcggaugggacgcggcagcagcucgccuguccuuuguguuc
mmu-mir-714	403	0	0	403	-	blast	cgacgagggccggucggucgc	-	cgacgagggccggucggucgcccugcgguugguucugugugugguuuggguc uugcgcuggggggaggcggggucgaccgcucgcggggguuggcgcggucgcccggcgccg
mmu-mir-467f	58	0	0	58	-	blast	auauacacacacacaccuaca	-	acggcuacauagagaaauccuguacccagaaaucgaacgggggugggugugugugugguagguguguguguguauguauguauauacacacacaccuacacacagcuuuuuuggaauuu
mmu-mir-6364	19	0	0	19	-	blast	ucgauccccagcacuccuguga	-	cuauagggcuggggaauagcuuagucagcaaagagcuuguuucacaagcaugaggacaugaguucgauccccagcacuccugugaa
mmu-mir-6931	12	0	0	12	-	blast	ugggguggggaguggggacu<br>cccaaccucuucauaccccag	-	gucacuggggugggggaguggggacuucugaaucacuagaggcucuggaaugacccaaccucuucauaccccag
mmu-mir-669j	39	0	0	39	-	blast	ugcauauacucacaugcaaaca	-	agcaugcgauucaugugauguccaccaagaaugugcaugugugauaguuguguacauauucacuuuuguauaugaauaugcauauacucacaugcaaacauacucccacgaauggcccga
mmu-mir-6236	90666	0	0	90666	-	blast	gccgucgccggcagucagg	-	acaacucaccugccgaaucaacuagcccugaaaauggauggcgcuggagcgugggggaccauacccagccgucgccggcagucaggggugggggguggggguggagaaagaaaaaaaaaucaauaa
mmu-mir-882	12	0	0	12	-	blast	aggagagaguuagcgcauuagu	-	cagcaguaccaggagagaguuagcgcauuagugcaauaguuaguccugauuucuggguuuuucuaaggcugcucuu
mmu-mir-8095	44	0	0	44	-	blast	aaaggauucugcugucuguccc	-	cguaauggcuguggggcaugcugagcugaggagaagaaaggauucugcugucugucccaugguuauuggagaugagauguaggccugugucaccccagacaguucuacuuuguagcccugguuggucagg
mmu-mir-1946b	22	0	0	22	-	blast	gccgggcaguggugggcacaugcuuuu	-	gggauuaaaggcgugcgccaccacugcccagcuuucagagugaauuugaggcuaucuggcuauaugagacugcuucacagguaagaaaggggaaagugccgggcaguggugggcacaugcuuuuaaucccag
mmu-mir-7035	2	0	0	2	-	blast	ggcugggagaguggcucagaa<br>ucugagccgcugucccugcag	-	ugaggggcugggagaguggcucagaagagaguaccuggcucuggggaggaaggggcagacuucugagccgcugucccugcag
mmu-mir-5116	26	0	0	26	-	blast	uuugauaggaaccccgccuga	-	auuuugauaggaaccccgccugaugcuuuuugguuuguuucaggcagggucuucauaucgaga
mmu-mir-3473f	805	0	0	805	-	blast	caaauaggacuggagagaug	-	ucugcuagagcagcauaugccccaaccacugagccaucucuccagccccuuuguuguuuucaauacauaaaauaagcaaauaggacuggagagauggcucagcgguuaagagcacugacugcucuuccagaggucu
mmu-mir-466l	1	0	0	1	-	blast	uauaaauacaugcacacauauu<br>uuguguguacauguacauguau	-	cuauguaugugcaugugugugguuuguguguacauguacauguauauauauauugauauacauauaaaauacaugcacacauauucaugcaugcacacgcacacauuaauggcacucuuagaucc
mmu-mir-449b	627	0	0	627	-	blast	aggcaguguuguuagcuggc	-	agacucggguaggcaguguuguuagcuggcugcguugggucaggccagcagccacagcuacccugccacuuccuucuggc
mmu-mir-3473c	8	0	0	8	-	blast	ucucuccagcccccauaauaag	-	cugcugagccaucucuccagcccccauaauaagucuuuuaaauguaaaauucauuauaggaguugggggagauggcucagua
mmu-mir-1192	1	0	0	1	-	blast	aaacaaacaaacagaccaaauu	-	ccuuccaaacaguaaaaacaaacaaacaaacagaccaaauuugucauuguuguuucgauuugcucuuuuuguuuguuugcuuuuguguuuugagacagggucuggcuuguauagcucaugc
mmu-mir-6946	22	0	0	22	-	blast	uuucuucucuucccuuucag<br>aaaagggagagaaagaaaugc	-	aaaagaaaagggagagaaagaaaugcaaggugggcaaagcuauuugacauuucuucucuucccuuucag
mmu-mir-344d-1	17925	17925	0	0	-	blast	gauauaaccacugccagacuga<br>agucaggcugcuggcuauacacca	-	aucagucaggcugcuggcuauacaccaggacuuaucugguccuggauauaaccacugccagacugaaag
mmu-mir-669i	55	55	0	0	-	blast	ugcauauacacacaugcauac<br>ugugcaugugugauaguugugugc	-	augugaugcccaccaauaaugugcaugugugauaguugugugcauauucauuucucuauaugacuaugcauauacacacaugcauacaaacucacauacaggaaugacagucauauauccuuuuga
mmu-mir-3472	10	0	0	10	-	blast	uaauagccagaagcuggaaggaacc	-	uucuuuccagcuucuggcuauuauaaauaaggcugcuccuuauuuauaauagccagaagcuggaaggaacc
mmu-mir-5098	215	0	0	215	-	blast	guuacauggugaagcccaguu	-	auuagccagaguuacauggugaagcccaguuuuguuuuguuuuguuuuuugagacaggguuucucuguguagcccuggcuguc
mmu-mir-1928	25	0	0	25	-	blast	agcuacauugccagcuc	-	aggauagagcuuugcgcauuguaggcaagugcucuaccacugagcuacauugccagcucuaacccca
mmu-mir-686	21	0	0	21	-	blast	auugcuucccagacggugaaga	-	gccacagcaggugcuuauauugcuucccagacggugaagaaaguaauagagaucaacccguaccuucugggcaccauggcugggggugcagcggauugcagcuucuggg
mmu-mir-669a-3	13262	13208	0	54	-	blast	acauaacauacacacacauguau<br>aguugugugugcauguucaugucu<br>aguugugugugcauguucaugucu	-	uuccuccauguaugugcaugugugauaguugugugugcauguucaugucuauauuugaauauacauaacauacacacacauguauaaacgcaagcacacauacacaga
mmu-mir-8101	54	0	0	54	-	blast	ggcgagcggagccggaggagcc	-	cuccgaauguuguuguugguggcggcggcgagcggagccggaggagccgccgcaaagauggaggagccgucgaggaggcgcugccgcugcuguugccgccgcugcugccgc
mmu-mir-5136	31	0	0	31	-	blast	auaugcgagggaacuacugg	-	gggccuuuccaagggugguuuucacgugagucuugauuuugucuuuauaugcgagggaacuacugggaacugggcc
mmu-mir-466k	852	409	0	34	-	blast	ugugugugugugugugugug<br>uguguguguacauguacauguga	-	auauaugugugugugugugugugugugguguacauguacaugugaguaugugugugugauauguuuguacaugugcaugugugugaguaugugaauauauguguauaugugugcaugugcaugugu
mmu-mir-713	40	0	0	40	-	blast	ugcacugaaggcacacagc	-	ggccuauccggaggcccugauugguuagugagacuugauugacaugagcaccucccuuaggguuagagugcacugaaggcacacagcauagggcuuccaagaucgggu
mmu-mir-8115	34	0	0	34	-	blast	ccgcaauagcaacaagggcucu	-	cccuggaacuggaacugcagguggguugggagccaccauggugguguugggaagugaacuugaguccuccgcaauagcaacaagggcucuuaacuacugagccaucucuccaccuucaaa
