# Supplementary material for: Identification of novel microRNAs in the embryonic mouse brain using deep sequencing
Source: Mol Cell Biochem. 2023 Apr 15;479(2):297–311. doi: 10.1007/s11010-023-04730-2 (PMC10890980; doi:10.1007/s11010-023-04730-2)
Supplement: Supplementary file 2 — miRDeep2 output and RNA secondary structure. Supplementary file2 (PDF 96 KB) [file 11010_2023_4730_MOESM2_ESM.pdf]

Provisional ID	:	gi_307603377_gb_CM000999.1__38372
Score total	:	28351.1
Score for star read(s)	:	3.9
Score for read counts	:	28349
Score for mfe	:	-3.2
Score for randfold	:	1.6
Score for cons. seed	:	
Total read count	:	55617
Mature read count	:	53812
Loop read count	:	1331
Star read count	:	474

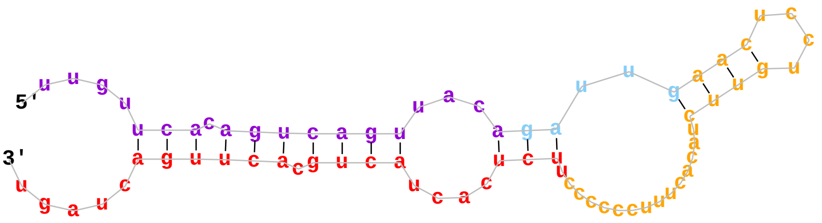

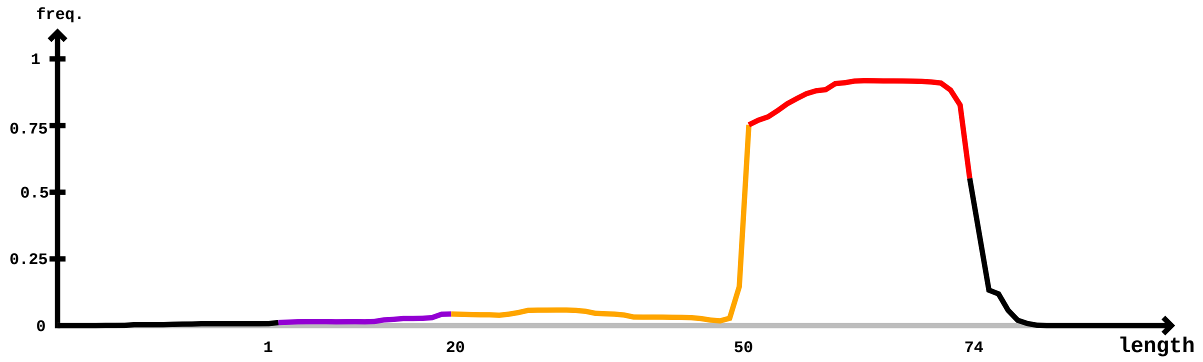

Star **Mature**

```
5'- guagugaguuaucucaauugauuguucacagucaguuacagauugaacuccuguucuacacuuucccccuucucacuacugcacuugacuagucuuuuuugaaacuuacaucu  -3'   obs
    guagugaguuaucucaauugauuguucacagucaguuacagauugaacuccuguucuacacuuucccccuucucacuacugcacuugacuagucuuuuuugaaacuuacaucu         exp
    ...(((((((.((..((..(((((.(((.(((((((...(((..((((....))))..............)))....)))).)))))).)))))..))..)))))))))....   reads   mm   sample
    ...gugaguuaucucaauuga............................................................................................   3       0    mmu
    ...gugaguuaucucaauugUuuguuc......................................................................................   1       1    mmu
    ...gugaguuaucucaauugauuguuc......................................................................................   26      0    mmu
    .....gaguuaucucaauugauuguuc......................................................................................   12      0    mmu
    ......aguuaucucaauugauuguucaca...................................................................................   160     0    mmu
    ......agGuaucucaauugauuguucaca...................................................................................   1       1    mmu
    .........uaucucaauugauuguucacaguc................................................................................   4       0    mmu
    ..........aucucaauugauuguucac....................................................................................   17      0    mmu
    ..........aucucaauugauuguucacagu.................................................................................   29      0    mmu
    ..........aucucaauugauugCucacaguc................................................................................   1       1    mmu
    ..........aucucaauugauuguucacaguc................................................................................   24      0    mmu
    ...........ucucaauugauuguucaca...................................................................................   10      0    mmu
    ...........ucucaauugauuguucacagu.................................................................................   26      0    mmu
    ...........ucucaauugauuguucacaguca...............................................................................   11      0    mmu
    ............cucaauugauuguucaca...................................................................................   1       0    mmu
    ............cucaauugauuCuucacaguca...............................................................................   1       1    mmu
    ............cucaauugauuguucacaguca...............................................................................   13      0    mmu
    ............cucaauugauuguucacagucG...............................................................................   1       1    mmu
    .............ucaauugauuguucacag..................................................................................   24      0    mmu
    .............ucaauugauuguCcacag..................................................................................   1       1    mmu
    .............ucaauugauuguucacagu.................................................................................   14      0    mmu
    .............ucaauugauuguCcacaguca...............................................................................   1       1    mmu
    .............ucaauugauuguucacaguca...............................................................................   37      0    mmu
    ....................auuguucacagucaguua...........................................................................   9       0    mmu
    ....................auuguucacagucaguuacagauu.....................................................................   6       0    mmu
    .....................uuguucacagucaguuac..........................................................................   24      0    mmu
    .....................uuguucacagucaguuaca.........................................................................   74      0    mmu
    .....................uuguucacagucagCuaca.........................................................................   1       1    mmu
    .....................uuguucacagucaguuacag........................................................................   37      0    mmu
    .....................uugCucacagucaguuacag........................................................................   1       1    mmu
    .....................uuguucacagucaguuacaga.......................................................................   34      0    mmu
    .....................uuguucacagucaguuacagauu.....................................................................   38      0    mmu
    .....................uuguucacagucaguuacagauug....................................................................   33      0    mmu
```

Star **Mature**

guagugaguuaucucaauugauuguucacagucaguuacagauugaacuccuguucuacacuuucccccuucucacuacugcacuugacuagucuuuuugaaacuuacaucu			
......................uguucacagucaguuaca..	15	0	mmu
......................uguucacagucaguCacaga..	1	1	mmu
......................uguucacagucaguuacaga..	13	0	mmu
......................uguucacagucaguuacagauu..	21	0	mmu
......................uguucacagucagCuacagauuga..	1	1	mmu
......................uguucacagucaguuacagauuga..	33	0	mmu
.......................guucacagucaguuacagauu..	32	0	mmu
.......................guucacagucaguuacagauug...	27	0	mmu
.......................guucacagucaguuacagauuga..	17	0	mmu
.......................guucacagucaguuacagauugaa...	19	0	mmu
........................uucacagucaguuacagauu..	24	0	mmu
.........................ucacagucaguuacagau...	2	0	mmu
............................cagucaguuacagauugaacuccu..	16	0	mmu
.............................agucaguuacagauugaacuccu..	10	0	mmu
.............................agucaguuacagauugaacuccug...	25	0	mmu
.............................agucaguuacGgauugaacuccug...	1	1	mmu
..............................gucaguuacagauugaacuccug...	22	0	mmu
..............................gucagCuacagauugaacuccugu..	1	1	mmu
..............................guGaguuacagauugaacuccugu..	1	1	mmu
..............................gucaguuacagauugaacuccugu..	117	0	mmu
..............................gucaguuacagauugaacuAcugu..	1	1	mmu
...............................ucaguuacagauugaacu...	12	0	mmu
...............................ucaguuacagauugaacuccu..	1	0	mmu
...............................ucaguuacagauugUacuccug...	1	1	mmu
...............................ucaguuacagauugaacuccug...	60	0	mmu
...............................ucaguuacagauugaacuccugu..	1	0	mmu
...............................ucaguuacagauugaacuccuguu...	13	0	mmu
................................caguuacagauugaacuccu..	7	0	mmu
................................cagCuacagauugaacuccug...	1	1	mmu
................................caguuacagauugaacuccug...	43	0	mmu
................................caguuacagauugaGcuccugu..	1	1	mmu
................................caguuacagauugaacuccugu..	197	0	mmu
................................caguuacagauugaacuccuguu...	104	0	mmu
................................cGguuacagauugaacuccuguu...	1	1	mmu
................................caguuacagauugaacuccuguuc..	66	0	mmu
.................................aguuacagauugaacuccCgu..	1	1	mmu
.................................aguuacagCuugaacuccugu..	1	1	mmu
.................................aguuacagauugGacuccugu..	1	1	mmu
.................................aguuacagauugaacuccugu..	81	0	mmu
.................................aguuacGgauugaacuccuguu...	1	1	mmu
.................................aguuacagauugaacuccuguu...	49	0	mmu
.................................aguuacagauugaacuccuguuc..	10	0	mmu
.................................aguuacaUauugaacuccuguucu...	7	1	mmu
..................................guuaAagauugaacuccu..	1	1	mmu
..................................guuacagauugaacuccu..	49	0	mmu
..................................guuacagauugaacuccug...	82	0	mmu
..................................guuacagauugaacuccuC...	1	1	mmu
..................................guuacagauugaacuccAgu..	1	1	mmu
..................................guuacagauugaacuccCgu..	1	1	mmu
..................................guuacagauugaacuccugu..	85	0	mmu
..................................guuacagauugaacuccuAu..	1	1	mmu
..................................guuacagauugaacuccuguucua..	35	0	mmu
....................................uacagauugaacuccuguucuac...	23	0	mmu
....................................uacagauugaacuccuguucuaca..	16	0	mmu
.....................................acagauugaacuccuguucu...	34	0	mmu
.....................................acagauugaacuccuguucC...	1	1	mmu
.....................................acagauugaacuccuguGcua..	1	1	mmu
.....................................acagGuugaacuccuguucua..	1	1	mmu
.....................................acagauugaacuccuguucCa..	1	1	mmu
.....................................acagauugaacuccuguucua..	57	0	mmu
.....................................acagauugaacuccuguucuac...	36	0	mmu
.....................................acagauugaacuccuguucuacac...	20	0	mmu
......................................cagauugaacuccuguuc..	96	0	mmu
......................................cagauugaacuccCguuc..	1	1	mmu
......................................cagauugaacuccugCuc..	1	1	mmu
......................................cagGuugaacuccuguuc..	1	1	mmu
......................................cagauugaacuccuguucu...	140	0	mmu
......................................cGgauugaacuccuguucu...	1	1	mmu
......................................cUgauugaacuccuguucu...	1	1	mmu
......................................cagauugaacuccuguucC...	1	1	mmu

Star **Mature**

guagugaguuaucucaauugauuguucacagucaguuacagauugaacuccuguucuacacuuucccccc uucucacuacugcacuugacuagucuuuuugaaacuuacaucu			
......................................cagauugaacuccuguuGu..	1	1	mmu
......................................cagGuugaacuccuguucua...	3	1	mmu
......................................cagauugaacuccuguuAua...	1	1	mmu
......................................cagauugaacuccuguucua...	425	0	mmu
......................................cagauugaacuccugCucua...	1	1	mmu
......................................cagauugaacucAuguucua...	2	1	mmu
......................................cagauugaacuccuguucuaca...	52	0	mmu
......................................cagauugaacuccuguucuacac..	12	0	mmu
......................................cagauugaacuccuguucuacacu...	24	0	mmu
....agauugaacuccuguucu..	46	0	mmu
....agauugaacucAuguucu..	1	1	mmu
....agauugaacuccuguucua...	37	0	mmu
....agauugaacuccuguucCa...	1	1	mmu
....gaacuccuguucuacacu...	19	0	mmu
....aacuccuguucuacacuu..	30	0	mmu
....aacuccuguucuacacuuu...	22	0	mmu
....aacuccuguucuacacuuuc..	26	0	mmu
....aacuccuguucCacacuuucc...	1	1	mmu
....aacuccuguucuacacuuucc...	56	0	mmu
....aacuccuguucuacacuuuccc..	87	0	mmu
....aacuccCguucuacacuuucccc...	1	1	mmu
....aacuccuguucuacacuuucccc...	49	0	mmu
....aacuccuguucuacacuuuccccc..	23	0	mmu
....Uacuccuguucuacacuuuccccc..	1	1	mmu
....acuccuguucuacacuuu...	55	0	mmu
....acuccuguucuacacuuA...	1	1	mmu
....acuccuguucuacacuuuc..	37	0	mmu
....acuccuguucuacaAuuuc..	1	1	mmu
....acuccuguucuacacuuucc...	65	0	mmu
....acuccuCuucuacacuuuccc..	1	1	mmu
....acuccuguucuacacuuuccc..	109	0	mmu
....acuccuguucuacacuuuccG..	1	1	mmu
....acuccuUuucuacacuuuccc..	1	1	mmu
....acuccuguucuacaUuuucccc...	1	1	mmu
....acuccuguucuacacuuucccc...	93	0	mmu
....acuccuguucuacacuuuUccc...	1	1	mmu
....acuccuguucuacacuuucccGc..	1	1	mmu
....acuccuguucuacacuuuccccc..	47	0	mmu
....cuccuguucuacacuuuc..	139	0	mmu
....cCccuguucuacacuuucc...	1	1	mmu
....cuccugAucuacacuuucc...	1	1	mmu
....cuccugCucuacacuuucc...	1	1	mmu
....cuccuguucuacacuuucc...	188	0	mmu
....cuccuguucuacacuuuccc..	103	0	mmu
....cuccuguucuacacuuucccc...	61	0	mmu
....cuccuguucuacacuuuccAc...	1	1	mmu
....cuccuguucuacacuuuccccccu..	12	0	mmu
....uccuguucuacacuuucc...	27	0	mmu
....uccuguucCacacuuucccc...	1	1	mmu
....uccuguucuacacuuucccc...	18	0	mmu
....ccuguucuacacuuuccc..	18	0	mmu
....cuguucuacacuuuccccccuu...	20	0	mmu
....uucuacacuuuccccccuucuca.....................................	36	0	mmu
....ucuacacuuuccccccuucuca.....................................	20	0	mmu
....Acuacacuuuccccccuucucac....................................	1	1	mmu
....ucuacacuuuccccccuucucac....................................	11	0	mmu
....ucuacacuuuccccccuucucacu...................................	16	0	mmu
....cuacacuuuccccccuucuca.....................................	20	0	mmu
....cuacaAuuuccccccuucucacu...................................	1	1	mmu
....cuacacuuuccccccuucucacu...................................	50	0	mmu
....uacacuuuccccccuucuc......................................	11	0	mmu
....uacacuuuccccccuucucac....................................	1	0	mmu
....uacacuuuccccccuucucacA...................................	14	1	mmu
....uacacuuuccccccuucucacu...................................	24	0	mmu
....uacacuuuccccccGucucacua..................................	1	1	mmu
....uacacuuuccccccuucucacua..................................	40	0	mmu
....uacaUuuuccccccuucucacua..................................	1	1	mmu
....acacuuuccccccuucuca.....................................	18	0	mmu
....acacuuuccccccuucucacu...................................	18	0	mmu
....cacuuuccccccuCcuca.....................................	1	1	mmu

	**Star** / **Mature**		
	guagugaguuaucucaauugauuguucacagucaguuacagauugaacuccuguucuacacuuuccccccuucucacuacugcacuugacuagucuuuuuugaaacuuacaucu		
	....cacuuuccccccuucuca......................................	12	0	mmu
	....Uacuuuccccccuucuca......................................	1	1	mmu
	....cacuuuccccccuucucacu....................................	20	0	mmu
	....cacuuuccAcccuucucacua...................................	1	1	mmu
	....cacuuuccccccuucucacua...................................	76	0	mmu
	....acuuuccccccuucucacua...................................	9	0	mmu
	....acuuuccccccuucucacuacu.................................	7	0	mmu
	....Gcuuuccccccuucucacuacug................................	19	1	mmu
	....cuuuccccccuucucacu....................................	9	0	mmu
	....cuuuccccccuucucacua...................................	18	0	mmu
	....cuuuccccccuucucacuacugc...............................	16	0	mmu
	....cuuuccccccuucucacuacugca..............................	31	0	mmu
	....uuuccccccuucucacuacu.................................	7	0	mmu
	....uuCccccccuucucacuacu.................................	1	1	mmu
	....uuuccccccuucucacuacug................................	20	0	mmu
	....uccccccuuUucacuacugcacu............................	1	1	mmu
	....uccccccuucucacuacugcacu............................	11	0	mmu
	....ccccccuucucacuacugc...............................	24	0	mmu
	....ccccccuucucacuacugca..............................	3	0	mmu
	....cccccCucucacuacugc...............................	1	1	mmu
	....cccuucucacuacugcacuugac........................	35	0	mmu
	....cccuucucacuacugcacuugGcu.......................	1	1	mmu
	....cccuucucacuacugcacuugaUu.......................	1	1	mmu
	....cccuucucacuacugcacuugacu.......................	108	0	mmu
	....cccuCcucacuacugcacuugacu.......................	1	1	mmu
	....ccuucucacuacugcacuuga.........................	26	0	mmu
	....ccuucucGcuacugcacuuga.........................	1	1	mmu
	....ccuucucacuacugcacuugac........................	66	0	mmu
	....ccuucucacuaUugcacuugac........................	1	1	mmu
	....ccGucucacuacugcacuugacu.......................	1	1	mmu
	....ccuucucUcuacugcacuugacu.......................	1	1	mmu
	....ccuucucacuacugcacuugacu.......................	290	0	mmu
	....ccuucucacuacugcacuugacua......................	412	0	mmu
	....ccuucucacuacugcacuugacAa......................	1	1	mmu
	....ccCucucacuacugcacuugacua......................	1	1	mmu
	....ccuucucacuacugcacuugUcua......................	1	1	mmu
	....ccuCcucacuacugcacuugacua......................	4	1	mmu
	....cuucucacuacugcaAuu...........................	22	1	mmu
	....cuucucacuacugcacuu...........................	14	0	mmu
	....cuucucacuacugcacuug..........................	41	0	mmu
	....cuucucacuGcugcacuug..........................	6	1	mmu
	....cuucucacuacugcacuugG.........................	2	1	mmu
	....cuucucacuacugcacuuga.........................	27	0	mmu
	....cuucucacuacugcaUuuga.........................	1	1	mmu
	....cuucucacuacugcacuugac........................	57	0	mmu
	....cuucucacuacugcacuuAac........................	1	1	mmu
	....cuucucacuacugcacuAgacu.......................	1	1	mmu
	....cuucucacuacCgcacuugacu.......................	1	1	mmu
	....cuucGcacuacugcacuugacu.......................	1	1	mmu
	....cuucucacuacugcacuugacu.......................	797	0	mmu
	....cuucucacuacugcacuugGcu.......................	3	1	mmu
	....cuGcucacuacugcacuugacu.......................	2	1	mmu
	....cuCcucacuacugcacuugacu.......................	1	1	mmu
	....cuucucGcuacugcacuugacua......................	1	1	mmu
	....cuucucacuacugcacCugacua......................	2	1	mmu
	....cuucucacuacugcacuugacua......................	1187	0	mmu
	....cuucucacuacugcCcuugacua......................	1	1	mmu
	....cuucucacuacCgcacuugacua......................	1	1	mmu
	....cuucAcacuacugcacuugacua......................	1	1	mmu
	....cuucucacuacugcacuugacuU......................	15	1	mmu
	....cuucucacuacugcacuugGcua......................	13	1	mmu
	....cuucucacuacugcacuCgacua......................	2	1	mmu
	....Uuucucacuacugcacuugacua......................	1	1	mmu
	....cuCcucacuacugcacuugacua......................	1	1	mmu
	....cuucucacuacugAacuugacua......................	1	1	mmu
	....cuucGcacuacugcacuugacuag.....................	2	1	mmu
	....cuucucacuacugcacuugUcuag.....................	1	1	mmu
	....cuucucacuacugcacuugacGag.....................	1	1	mmu
	....cuucucacuacugcacuuAacuag.....................	1	1	mmu
	....cuucucacCacugcacuugacuag.....................	2	1	mmu

Star Mature

guagugaguuaucucaauugauuguucacagucaguuacagauugaacuccuguucuacacuuucccccuucucacuacugcacuugacuagucuuuuugaaacuuacaucu			
....cuucucacuacugcaUuugacuag....................	1	1	mmu
....cuucucaUuacugcacuugacuag....................	1	1	mmu
....cuucucacuacugcacuCgacuag....................	1	1	mmu
....cuucCcacuacugcacuugacuag....................	31	1	mmu
....cuuGucacuacugcacuugacuag....................	2	1	mmu
....cuAcucacuacugcacuugacuag....................	1	1	mmu
....cCucucacuacugcacuugacuag....................	10	1	mmu
....cuucucacuacugcGcuugacuag....................	4	1	mmu
....cuucucacuacCgcacuugacuag....................	8	1	mmu
....cuucucacuacugcUcuugacuag....................	1	1	mmu
....cuucucacuacugcacuugacuag....................	4554	0	mmu
....cuucuUacuacugcacuugacuag....................	15	1	mmu
....cuucucacuacugcacuugGcuag....................	71	1	mmu
....cuucucacuacugcacCugacuag....................	3	1	mmu
....cuucucacuaUugcacuugacuag....................	1	1	mmu
....cuucucacuacugcacuugacuaU....................	23	1	mmu
....cuucucacuacugcacuAgacuag....................	2	1	mmu
....cuucucGcuacugcacuugacuag....................	3	1	mmu
....cuCcucacuacugcacuugacuag....................	2	1	mmu
....cuucucacuacuAcacuugacuag....................	25	1	mmu
....cuucucacuacGgcacuugacuag....................	1	1	mmu
....cuucucacuacugcacuugacuaA....................	131	1	mmu
....cuucucacuacugcacuGgacuag....................	1	1	mmu
....cuucucacuacugcacuugacuGg....................	2	1	mmu
....cuucucacuGcugcacuugacuag....................	4	1	mmu
....cuucucacuacugcacuugaUuag....................	3	1	mmu
....uucucacuacugcacuug.........................	8	0	mmu
....uucucacuacugcacuuga........................	18	0	mmu
....uucucacuacugcacuugac.......................	77	0	mmu
....uucuUacuacugcacuugac.......................	1	1	mmu
....uucucacuacugcacuugacA......................	7	1	mmu
....uucucacuacugcaGuugacu......................	2	1	mmu
....uucucacuacugcacuugGcu......................	2	1	mmu
....uucucacuacugcacuugacu......................	238	0	mmu
....uucucacuacugcacuuCacu......................	1	1	mmu
....Cucucacuacugcacuugacu......................	1	1	mmu
....uucucacuacugcacuCgacu......................	1	1	mmu
....uucucacuacugcacCugacua.....................	1	1	mmu
....uucuGacuacugcacuugacua.....................	1	1	mmu
....uucucacuacugcacuugacuG.....................	1	1	mmu
....Cucucacuacugcacuugacua.....................	1	1	mmu
....uCcucacuacugcacuugacua.....................	1	1	mmu
....uucucacuacugcacuugacua.....................	1243	0	mmu
....uucCcacuacugcacuugacua.....................	5	1	mmu
....uucucacuacugcacuuAacua.....................	9	1	mmu
....uucucacuacugUacuugacua.....................	1	1	mmu
....uucucacuacCgcacuugacua.....................	2	1	mmu
....uucucacuCcugcacuugacuag....................	1	1	mmu
....uucucacuacugcacuCgacuag....................	6	1	mmu
....uucucacuacugcacuuAacuag....................	1	1	mmu
....uucucacAacugcacuugacuag....................	1	1	mmu
....uucucacuacuCcacuugacuag....................	2	1	mmu
....uucAcacuacugcacuugacuag....................	1	1	mmu
....uucGcacuacugcacuugacuag....................	2	1	mmu
....uucucacuacugcacuugacGag....................	1	1	mmu
....uucucacuacugcGcuugacuag....................	11	1	mmu
....uucucacuacugcacuugacuaA....................	12	1	mmu
....uucucacuaGugcacuugacuag....................	1	1	mmu
....uucucacuacugcacuuUacuag....................	1	1	mmu
....uucucacuacugcacuugUcuag....................	1	1	mmu
....uucucacuacugcacuugacuaU....................	71	1	mmu
....Cucucacuacugcacuugacuag....................	6	1	mmu
....uucucacuGcugcacuugacuag....................	3	1	mmu
....uGcucacuacugcacuugacuag....................	1	1	mmu
....uuAucacuacugcacuugacuag....................	1	1	mmu
....uucucacuacugUacuugacuag....................	1	1	mmu
....uucucacuUcugcacuugacuag....................	2	1	mmu
....uucucacuacugcaGuugacuag....................	6	1	mmu
....uucucacuacuAcacuugacuag....................	7	1	mmu
....uucuUacuacugcacuugacuag....................	2	1	mmu

Star **Mature**

guagugaguuaucucaauugauuguucacagucaguuacagauugaacuccuguucuacacuuucccccuucucacuacugcacuugacuagucuuuuugaaacuuacaucu			
....uucucacuacuUcacuugacuag....................	1	1	mmu
....uucucacuacugcacuugacAag....................	3	1	mmu
....Gucucacuacugcacuugacuag....................	2	1	mmu
....uuGucacuacugcacuugacuag....................	2	1	mmu
....uucucacuacugcacuuCacuag....................	2	1	mmu
....uCcucacuacugcacuugacuag....................	14	1	mmu
....uucucacuacugcacuugGcuag....................	11	1	mmu
....uucucacuacugcacuugacuCg....................	1	1	mmu
....uucucacuacugcacuugacuGg....................	4	1	mmu
....uucCcacuacugcacuugacuag....................	10	1	mmu
....uucucacuacugcacuugacuag....................	10448	0	mmu
....uucucacuacugGacuugacuag....................	2	1	mmu
....uucucGcuacugcacuugacuag....................	9	1	mmu
....uucuAacuacugcacuugacuag....................	2	1	mmu
....uucucacCacugcacuugacuag....................	1	1	mmu
....uucucacuacCgcacuugacuag....................	9	1	mmu
....uucucacuacugcacCugacuag....................	2	1	mmu
....uucuGacuacugcacuugacuag....................	2	1	mmu
....uucAcacuacugcacuugacuagu...................	4	1	mmu
....uucucacuacugcacAugacuagu...................	1	1	mmu
....uucucacuaGugcacuugacuagu...................	1	1	mmu
....uucucCcuacugcacuugacuagu...................	1	1	mmu
....uucucacuacugcacuugacuaAu...................	72	1	mmu
....uucucacuacugGacuugacuagu...................	1	1	mmu
....uucucacuacugcacuugaGuagu...................	1	1	mmu
....uucuGacuacugcacuugacuagu...................	8	1	mmu
....uucucacuacugcacuugacGagu...................	1	1	mmu
....uucucacuacugcacuugGcuagu...................	22	1	mmu
....uucucacuacuUcacuugacuagu...................	1	1	mmu
....uucucacuacugcacuugaUuagu...................	3	1	mmu
....uucucacuacugcUcuugacuagu...................	2	1	mmu
....uucuUacuacugcacuugacuagu...................	8	1	mmu
....uucucacuacugcaAuugacuagu...................	2	1	mmu
....Aucucacuacugcacuugacuagu...................	2	1	mmu
....uucucacuacuAcacuugacuagu...................	8	1	mmu
....uucucacuacugAacuugacuagu...................	1	1	mmu
....uucucacuacuCcacuugacuagu...................	2	1	mmu
....uucucacuacugcacuugacuagu...................	17615	0	mmu
....uucGcacuacugcacuugacuagu...................	4	1	mmu
....uucucacuUcugcacuugacuagu...................	1	1	mmu
....uucucacuacCgcacuugacuagu...................	20	1	mmu
....uucucacuacugcacuugacuagG...................	11	1	mmu
....uCcucacuacugcacuugacuagu...................	27	1	mmu
....uucucacuacGgcacuugacuagu...................	1	1	mmu
....uucucacuacugcaGuugacuagu...................	14	1	mmu
....uucucacuGcugcacuugacuagu...................	2	1	mmu
....uuAucacuacugcacuugacuagu...................	3	1	mmu
....uuUucacuacugcacuugacuagu...................	9	1	mmu
....uucuAacuacugcacuugacuagu...................	7	1	mmu
....uucucGcuacugcacuugacuagu...................	18	1	mmu
....uucucacuacugcacuugacuagC...................	79	1	mmu
....uAcucacuacugcacuugacuagu...................	2	1	mmu
....uucucacuacugcacCugacuagu...................	17	1	mmu
....uucucacuacugcaUuugacuagu...................	7	1	mmu
....uucucacuacugcacuugacAagu...................	1	1	mmu
....uucucacuacugUacuugacuagu...................	19	1	mmu
....uucucUcuacugcacuugacuagu...................	5	1	mmu
....uucucacuacugcacuugacuUgu...................	1	1	mmu
....uucucacuacugcacuuUacuagu...................	2	1	mmu
....Cucucacuacugcacuugacuagu...................	16	1	mmu
....Gucucacuacugcacuugacuagu...................	1	1	mmu
....uucucacuacugcacuCgacuagu...................	10	1	mmu
....uuGucacuacugcacuugacuagu...................	3	1	mmu
....uucucacuacugcCcuugacuagu...................	2	1	mmu
....uucCcacuacugcacuugacuagu...................	21	1	mmu
....uucucacuacugcacuAgacuagu...................	1	1	mmu
....uucucacuacugcacuugacuaCu...................	6	1	mmu
....uucucacAacugcacuugacuagu...................	1	1	mmu
....uucucacuacugcacuugacuGgu...................	17	1	mmu
....uucucaUuacugcacuugacuagu...................	3	1	mmu

```
                                          Star                                              Mature
guagugaguuaucucaauugauuguucacagucaguuacagauugaacuccuguucuacacuuucccccuucucacuacugcacuugacuagucuuuuuugaaacuuacaucu
.....................................................................uucucacuacAgcacuugacuagu....................    2      1    mmu
.....................................................................uucucacuacugcGcuugacuagu....................    7      1    mmu
.....................................................................uucucacuacugcacuugacuagA....................    5101   1    mmu
.....................................................................uucucacuacugcacuugacuaUu....................    486    1    mmu
.....................................................................uucucacCacugcacuugacuagu....................    9      1    mmu
.....................................................................uGcucacuacugcacuugacuagu....................    1      1    mmu
.....................................................................uucucacuacugcacuuAacuagu....................    6      1    mmu
.....................................................................uucucacuaUugcacuugacuagu....................    2      1    mmu
......................................................................ucucacuacugcacuuga.........................    53     0    mmu
......................................................................ucucacuacugcacuugacu.......................    44     0    mmu
......................................................................ucucacuacugcacuugacua......................    110    0    mmu
......................................................................ucucacuacCgcacuugacuag.....................    1      1    mmu
......................................................................Ccucacuacugcacuugacuag.....................    1      1    mmu
......................................................................ucucacuacugcacuugacuag.....................    288    0    mmu
......................................................................ucucacuacugUacuugacuag.....................    1      1    mmu
......................................................................ucucacuacugcacuugGcuag.....................    1      1    mmu
......................................................................ucucacuacugcacuuAacuag.....................    1      1    mmu
......................................................................ucucacuacugcacuugacuaUu....................    27     1    mmu
......................................................................ucAcacuacugcacuugacuagu....................    2      1    mmu
......................................................................ucCcacuacugcacuugacuagu....................    2      1    mmu
......................................................................ucucacuacugcacuugacuagu....................    339    0    mmu
......................................................................ucucacuacugcacuGgacuagu....................    1      1    mmu
......................................................................ucucacuacugcacuugacuagA....................    99     1    mmu
......................................................................ucucacuacugcacuugacuaAu....................    15     1    mmu
......................................................................ucucacuacugcacuugacuaguU...................    11     1    mmu
......................................................................ucucacuacugcacuugacuaguc...................    60     0    mmu
......................................................................ucucacuacugcacuugUcuaguc...................    1      1    mmu
......................................................................ucGcacuacugcacuugacuaguc...................    1      1    mmu
......................................................................ucucacuacugcacuugacuaguA...................    1      1    mmu
.......................................................................cucacuacugcacuugacu.......................    34     0    mmu
.......................................................................cucacuacugcacuugacua......................    214    0    mmu
.......................................................................Aucacuacugcacuugacua......................    1      1    mmu
.......................................................................cucacuacugcacuugaGua......................    1      1    mmu
.......................................................................cucacuacugcacuugacuag.....................    92     0    mmu
.......................................................................Uucacuacugcacuugacuag.....................    45     1    mmu
.......................................................................cuUacuacugcacuugacuag.....................    1      1    mmu
.......................................................................cucacuacugcacuugacuagu....................    86     0    mmu
.......................................................................Uucacuacugcacuugacuagu....................    9      1    mmu
.......................................................................cucacuacugcacuugacuaguU...................    3      1    mmu
.......................................................................cucacuacugcacuugacuaguc...................    1      0    mmu
.......................................................................Uucacuacugcacuugacuaguc...................    1      1    mmu
.......................................................................cucacuacugcacuugacuagucu..................    272    0    mmu
.......................................................................cucacuacugcacCugacuagucu..................    1      1    mmu
.......................................................................Uucacuacugcacuugacuagucu..................    1      1    mmu
........................................................................ucacuacugcacuugacu.......................    20     0    mmu
........................................................................ucacuacugcacuugacua......................    11     0    mmu
........................................................................ucacuacugcacuugacuag.....................    55     0    mmu
........................................................................ucacuacugUacuugacuag.....................    1      1    mmu
........................................................................ucacuacugcacuugacuagu....................    43     0    mmu
........................................................................ucacuacugcacCugacuaguc...................    6      1    mmu
........................................................................ucacuacugcacuugacuaguc...................    34     0    mmu
........................................................................ucacuacugcacuugacuagucu..................    24     0    mmu
........................................................................uUacuacugcacuugacuagucu..................    12     1    mmu
........................................................................ucacuacugcacuugacuagucGu.................    2      1    mmu
........................................................................ucacuacugcacuugacuagucCu.................    1      1    mmu
........................................................................uUacuacugcacuugacuagucuu.................    45     1    mmu
........................................................................ucacuacugcacuCgacuagucuu.................    3      1    mmu
........................................................................ucacCacugcacuugacuagucuu.................    1      1    mmu
........................................................................ucacGacugcacuugacuagucuu.................    1      1    mmu
........................................................................ucacuacugcacuugacuaUucuu.................    11     1    mmu
........................................................................ucacuacugcacuugacuagAcuu.................    1      1    mmu
........................................................................ucacuacugcGcuugacuagucuu.................    1      1    mmu
........................................................................ucacuacugcacCugacuagucuu.................    1      1    mmu
........................................................................ucacuacugcacuugacuagucuu.................    783    0    mmu
........................................................................ucaGuacugcacuugacuagucuu.................    1      1    mmu
........................................................................ucacuacugcacuugacCagucuu.................    1      1    mmu
........................................................................Ccacuacugcacuugacuagucuu.................    5      1    mmu
........................................................................ucacuacugcacuugacuagucuA.................    314    1    mmu
.........................................................................cacuacugcacuugacua......................    81     0    mmu
.........................................................................cacuacugcGcuugacua......................    1      1    mmu
```

Star **Mature**

guagugaguuaucucaauugauuguucacagucaguuacagauugaacuccuguucuacacuuucccccuucucacuacugcacuugacuagucuuuuugaaacuuacaucu			
....cacuacugcacuugacuag....................	102	0	mmu
....Gacuacugcacuugacuag....................	1	1	mmu
....cacuacugcacuugacuagu...................	56	0	mmu
....cacuacugcacuugacuaUu...................	3	1	mmu
....cacuacugcacuugacuaguA..................	18	1	mmu
....cacuacugcacuugacuagucu.................	96	0	mmu
....cacuacugcacuugacuagucA.................	11	1	mmu
....cacuacuAcacuugacuagucuu................	1	1	mmu
....cGcuacugcacuugacuagucuu................	1	1	mmu
....cacAacugcacuugacuagucuu................	1	1	mmu
....cacuacugcacuugacuaUucuu................	19	1	mmu
....Aacuacugcacuugacuagucuu................	23	1	mmu
....cacuacugcacuugacuagCcuu................	13	1	mmu
....cacuacugcacuugacuagucuu................	480	0	mmu
....cacuacugcacuugacuagucuA................	183	1	mmu
....cacuacugcacuugacuagucuuu...............	208	0	mmu
....caUuacugcacuugacuagucuuu...............	1	1	mmu
....cacuacugcacuugacuagucuuC...............	17	1	mmu
....cacuacugcacuugacCagucuuu...............	2	1	mmu
....cGcuacugcacuugacuagucuuu...............	2	1	mmu
....cacuacugcacuugacuagucuuA...............	212	1	mmu
....acuacugcacuugacuag....................	155	0	mmu
....acAacugcacuugacuag....................	1	1	mmu
....acuacugcacuugacuagu...................	45	0	mmu
....acuacugcacuugacuaguc..................	16	0	mmu
....acuacugcacuugacuaUucu.................	38	1	mmu
....acuacugcacuugacuagucu.................	29	0	mmu
....acuCcugcacuugacuagucuu................	1	1	mmu
....acuacAgcacuugacuagucuu................	1	1	mmu
....acuacugcacuugacuagAcuu................	1	1	mmu
....acuacugcacuugUcuagucuu................	1	1	mmu
....acuacugcacuugacuagucuu................	365	0	mmu
....acuacugcacuugacuagucuA................	61	1	mmu
....Ccuacugcacuugacuagucuu................	31	1	mmu
....acuacugcacuugacuagucuAu...............	15	1	mmu
....acuacugcacuugacCagucuuu...............	1	1	mmu
....acuacugcacuugacuagucuuA...............	221	1	mmu
....acuacCgcacuugacuagucuuu...............	1	1	mmu
....acuacugcacuugacuagucuuu...............	144	0	mmu
....acuacugcacuugacuagucuuGu..............	10	1	mmu
....acuacugcacuugacuagucuAuu..............	25	1	mmu
....acuacugcacuugacuagucuuuu..............	7	0	mmu
....cuacugcacuuUacuagu...................	1	1	mmu
....cuacugcacuugacuagu...................	121	0	mmu
....cuacugcacCugacuagu...................	1	1	mmu
....cuacugcacuugacuaguc..................	16	0	mmu
....cuacugcacuugacuaguU..................	26	1	mmu
....cuacuCcacuugacuagucu.................	1	1	mmu
....cuacAgcacuugacuagucu.................	1	1	mmu
....cuacugcacuugacuagucA.................	18	1	mmu
....cuacuAcacuugacuagucu.................	1	1	mmu
....cuacugcacuugacuagucu.................	162	0	mmu
....cuacugcGcuugacuagucu.................	1	1	mmu
....cuacugcacuugacuagucCu................	1	1	mmu
....cuacugUacuugacuagucuu................	1	1	mmu
....cuacugcacuCgacuagucuu................	3	1	mmu
....cuacugcacuugacuagucuu................	412	0	mmu
....cuacuAcacuugacuagucuu................	1	1	mmu
....cuacugcacuugacuagucuA................	80	1	mmu
....cuGcugcacuugacuagucuu................	1	1	mmu
....cCacugcacuugacuagucuu................	1	1	mmu
....cuacugcacuugacuagucuuu...............	80	0	mmu
....cuacugcacuugacuagucuuA...............	212	1	mmu
....cuacugcacuugacuagucuuuu..............	37	0	mmu
....cuacugcacuugacGagucuuuu..............	1	1	mmu
....cuacugcacuugacuagucuuCu..............	9	1	mmu
....uacugcacuugacuaguc..................	47	0	mmu
....uacugcacuugacuaguG..................	1	1	mmu
....uacugcacuugacuaguU..................	67	1	mmu
....uacugcacuugacuagucu.................	69	0	mmu

Star **Mature**

```
guagugaguuaucucaauugauuguucacagucaguuacagauugaacuccuguucuacacuuucccccuucucacuacugcacuugacuagucuuuuuugaaacuuacaucu
............................................................................uacugcacuugacuagucuA.................  93   1  mmu
............................................................................uacugcacuugacuagucuu.................  212  0  mmu
............................................................................uacugcacuugacuagucuuu................  16   0  mmu
............................................................................uacugcacuugacuagucuuA................  122  1  mmu
............................................................................uacugcacuugacuagucuuuu...............  29   0  mmu
............................................................................uacugcacuugacuagucuuuuu..............  4    0  mmu
.............................................................................acugcacuuUacuagucu..................  45   1  mmu
.............................................................................acugcacuugacuagucu..................  65   0  mmu
.............................................................................acugcacuugGcuagucu..................  1    1  mmu
.............................................................................acAgcacuugacuagucuu.................  1    1  mmu
.............................................................................acugcacuugacuagucuA.................  65   1  mmu
.............................................................................acugcacuugacuaguUuu.................  19   1  mmu
.............................................................................acugcacuugacuagucuu.................  58   0  mmu
.............................................................................acugcGcuugacuagucuu.................  1    1  mmu
.............................................................................acugcacuugacuagucuuA................  82   1  mmu
.............................................................................acugcacuugacuagucuuu................  21   0  mmu
.............................................................................acugcacuugacuagucuuAu...............  3    1  mmu
.............................................................................acugcacuugacuagucuAuu...............  17   1  mmu
.............................................................................acugcacuugacuagucuuuAuu.............  23   1  mmu
..............................................................................cugcacuugacuagucuA.................  110  1  mmu
..............................................................................cugcacuugacuagucuu.................  219  0  mmu
..............................................................................cugcacuugacuagucAu.................  17   1  mmu
..............................................................................cugcacuugacuaguUuuu................  3    1  mmu
..............................................................................cAgcacuugacuagucuuu................  1    1  mmu
..............................................................................cugcacuugacuagucuuA................  331  1  mmu
..............................................................................cugcacuugacuagucuuu................  387  0  mmu
..............................................................................cugcacCugacuagucuuu................  1    1  mmu
..............................................................................cuUcacuugacuagucuuuu...............  13   1  mmu
..............................................................................cugcacuugacuagucuuuu...............  142  0  mmu
..............................................................................cugcacuugacuagucuuuA...............  65   1  mmu
..............................................................................cugGacuugacuagucuuuuu..............  1    1  mmu
..............................................................................cugcacuugacuagucuuuuu..............  201  0  mmu
...............................................................................ugcacuugacuagucuCu................  16   1  mmu
...............................................................................ugcacuugacuagucuuA................  80   1  mmu
...............................................................................ugcacuugacuagucuuu................  40   0  mmu
...............................................................................ugcacuugacuagucuuuA...............  19   1  mmu
...............................................................................ugcacuugacuagucuuuu...............  14   0  mmu
...............................................................................ugcacuugacuagucuuuuA..............  10   1  mmu
...............................................................................ugcacuugacuagucuuuuuu.............  11   0  mmu
...............................................................................ugcacuugacuagucuuuuuA.............  10   1  mmu
................................................................................gcacuugacuagGcuuuu...............  1    1  mmu
................................................................................gcacuugacuagucuuuu...............  296  0  mmu
................................................................................gcacuuAacuagucuuuu...............  1    1  mmu
................................................................................gcGcuugacuagucuuuu...............  10   1  mmu
................................................................................Acacuugacuagucuuuu...............  1    1  mmu
................................................................................gcacuugacuagucuuuA...............  37   1  mmu
................................................................................gcacuugacuagucuuuGu..............  34   1  mmu
.................................................................................cacuugacuagucuuuuu..............  5    0  mmu
.................................................................................cacuugacuagucuuuuA..............  108  1  mmu
.................................................................................cacuugacuagucuuuuuA.............  9    1  mmu
..................................................................................Ucuugacuagucuuuuuu.............  23   1  mmu
```

Provisional ID	:	gi_307603372_gb_CM001004.1__3793
Score total	:	9363.8
Score for star read(s)	:	3.9
Score for read counts	:	9356.9
Score for mfe	:	1.4
Score for randfold	:	1.6
Score for cons. seed	:	
Total read count	:	18365
Mature read count	:	18364
Loop read count	:	0
Star read count	:	1

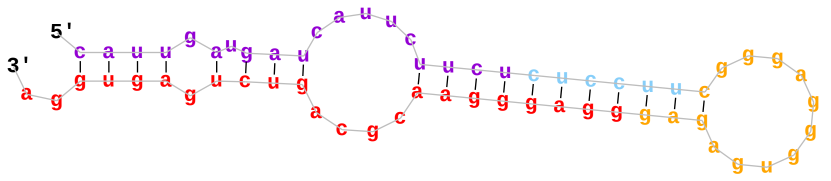

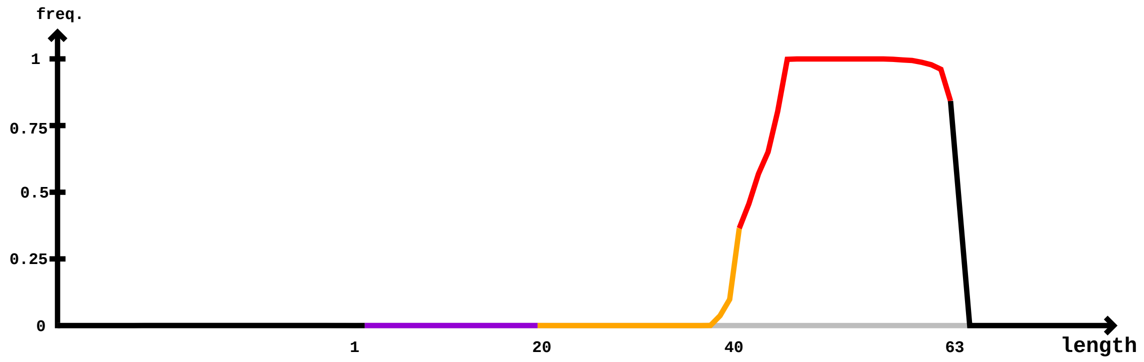

Star Mature

```
5'- aggugcugcuugauugcagcugccucuugccauugaugaucauucuucucuccuucgggagggugagagggagggaacgcagucugagugga uuugguuuauuaaaaa   -3'    obs
    aggugcugcuugauugcagcugccucuugccauugaugaucauucuucucuccuucgggagggugagagggagggaacgcagucugaguggauuugguuuauuaaaaa          exp
    (((((((((......))))).))))....(((((.(.(((.....(((((((((((..........)))))))))))....)))).))))).................    reads      mm      sample
    .......gcuugaCugcagcugccucu.................................................................................    3          1       mmu
    .......gcuugaCugcagcugccucuug...............................................................................    1          1       mmu
    ..............................cauugaugaucauucuucC...........................................................    1          1       mmu
    ..................................................................gagggagggaacgcagucuga.....................    5          0       mmu
    ..................................................................gagggagggaacgcagucugagug..................    3          0       mmu
    ...................................................................agggagggaacgcagucu.......................    20         0       mmu
    ...................................................................agggagggGacgcagucu.......................    2          1       mmu
    ...................................................................agggagggaacgcagucug......................    24         0       mmu
    ...................................................................agggagggaacgcagucuga.....................    13         0       mmu
    ...................................................................agggagggaacgcagucugag....................    23         0       mmu
    ...................................................................agggagggaacgcagucugagu...................    29         0       mmu
    ...................................................................agggagggaacgcagucugaguA..................    10         1       mmu
    ...................................................................agggGgggaacgcagucugagugg.................    1          1       mmu
    ...................................................................agggagggaacgcagucugagugg.................    551        0       mmu
    ...................................................................agggagggUacgcagucugagugg.................    1          1       mmu
    ....................................................................gggagggaacgcagucug......................    22         0       mmu
    ....................................................................gggagggaacgcagucugag....................    38         0       mmu
    ....................................................................gggagggaacgcagucugagu...................    34         0       mmu
    ....................................................................gggagggaacgcagucugagug..................    55         0       mmu
    ....................................................................gggagggaacgcagCcugagug..................    1          1       mmu
    ....................................................................gggagggaacgcagucCgagug..................    1          1       mmu
    ....................................................................gggagggaacgcagucugagugg.................    124        0       mmu
    ....................................................................gggagggaacgcagucugUgugga................    1          1       mmu
    ....................................................................gggagggaGcgcagucugagugga................    2          1       mmu
    ....................................................................gUgagggaacgcagucugagugga................    22         1       mmu
    ....................................................................gggaAggaacgcagucugagugga................    3          1       mmu
    ....................................................................gggagggaacgcagCcugagugga................    1          1       mmu
    ....................................................................gAgagggaacgcagucugagugga................    1          1       mmu
    ....................................................................gggagggaacgcagucugaguggG................    1          1       mmu
    ....................................................................Cggagggaacgcagucugagugga................    64         1       mmu
    ....................................................................gggagggaacgcagucugGgugga................    1          1       mmu
    ....................................................................gggagggaacgcagucCgagugga................    1          1       mmu
    ....................................................................gggagggaacgcagucugagugga................    751        0       mmu
```

Star **Mature**

```
aggugcugcuugauugcagcugccucuugccauugaugaucauucuucucuccuucgggagggugagagggagggaacgcagucugaguggauuugguuuauuaaaaa
....................................................................gggaggCaacgcagucugagugga................ 1 1 mmu
.....................................................................ggagggaacgcagucuga..................... 20 0 mmu
.....................................................................ggagggaacgcagucuUagu................... 1 1 mmu
.....................................................................ggagggaacgcagucugagu................... 41 0 mmu
.....................................................................ggagggaacgcagucugagug.................. 100 0 mmu
.....................................................................gUagggaacgcagucugagug.................. 5 1 mmu
.....................................................................ggagggaacgcaguAugagugg................. 1 1 mmu
.....................................................................ggagggGacgcagucugagugg................. 1 1 mmu
.....................................................................ggagggaacgcagucugUgugg................. 1 1 mmu
.....................................................................ggagggaacgcaguGugagugg................. 1 1 mmu
.....................................................................ggagggCacgcagucugagugg................. 1 1 mmu
.....................................................................ggGgggaacgcagucugagugg................. 2 1 mmu
.....................................................................ggagggaacgcagucugGgugg................. 1 1 mmu
.....................................................................ggagggaacgcagucugaguAg................. 1 1 mmu
.....................................................................ggagggaacgcagucugagugg................. 810 0 mmu
.....................................................................ggaCggaacgcagucugagugg................. 1 1 mmu
.....................................................................ggagggaacgcagAcugagugg................. 1 1 mmu
.....................................................................ggagggaaUgcagucugagugg................. 6 1 mmu
.....................................................................ggGgggaacgcagucugagugga................ 1 1 mmu
.....................................................................ggagCgaacgcagucugagugga................ 1 1 mmu
.....................................................................ggagggaacgcagucugaguggG............... 4 1 mmu
.....................................................................ggaggCaacgcagucugagugga................ 1 1 mmu
.....................................................................ggagggaacgcagucugagCgga................ 2 1 mmu
.....................................................................Ugagggaacgcagucugagugga................ 11 1 mmu
.....................................................................ggagggaGcgcagucugagugga................ 5 1 mmu
.....................................................................ggagggaacgcagucugagugga................ 3824 0 mmu
.....................................................................ggagggaacgcagucugaguAga................ 8 1 mmu
.....................................................................ggagggaacgcagucugGgugga................ 12 1 mmu
.....................................................................ggagggaacAcagucugagugga................ 2 1 mmu
.....................................................................gAagggaacgcagucugagugga................ 1 1 mmu
.....................................................................ggagggaacgcUgucugagugga................ 1 1 mmu
.....................................................................ggagggaacgcagucugagGgga................ 1 1 mmu
.....................................................................ggagggGacgcagucugagugga................ 7 1 mmu
.....................................................................ggagggaacgcGgucugagugga................ 2 1 mmu
.....................................................................Agagggaacgcagucugagugga................ 2 1 mmu
.....................................................................ggagggaacgcagucCgagugga................ 3 1 mmu
.....................................................................ggagggaacgcagCcugagugga................ 15 1 mmu
.....................................................................ggagUgaacgcagucugagugga................ 1 1 mmu
.....................................................................gCagggaacgcagucugagugga................ 1 1 mmu
......................................................................gagggaacgcagucugag.................... 63 0 mmu
......................................................................gagggaacgcagucugagu................... 36 0 mmu
......................................................................gagggaacgcagucugagug.................. 10 0 mmu
......................................................................gagggaacgcagucugagCgg................. 2 1 mmu
......................................................................gagggaacgcagucugagugg................. 352 0 mmu
......................................................................gagggaacgcagucugaAugg................. 1 1 mmu
......................................................................Aagggaacgcagucugagugg................. 1 1 mmu
......................................................................gagggGacgcagucugagugg................. 2 1 mmu
......................................................................gagggaacgcagucugaguggG................ 2 1 mmu
......................................................................gaUggaacgcagucugagugga................ 29 1 mmu
......................................................................gagggaGcgcagucugagugga................ 1 1 mmu
......................................................................gagggaacgcagCcugagugga................ 1 1 mmu
......................................................................gagggGacgcagucugagugga................ 1 1 mmu
......................................................................gagggaacgcagucugGgugga................ 1 1 mmu
......................................................................gagggaacgcagucugagCgga................ 1 1 mmu
......................................................................gagggaacgcagucugagugga................ 1177 0 mmu
......................................................................gagggaacgcagucugaUugga................ 1 1 mmu
......................................................................gagggaacgcagucugagGgga................ 1 1 mmu
......................................................................gagggaacgcagucugaAugga................ 1 1 mmu
......................................................................gGgggaacgcagucugagugga................ 1 1 mmu
......................................................................gagggaacgcagAcugagugga................ 1 1 mmu
.......................................................................agggaacgcagucugagu................... 28 0 mmu
.......................................................................Ggggaacgcagucugagu................... 1 1 mmu
.......................................................................agggaacgcagucugagug.................. 60 0 mmu
.......................................................................agggaacgcagucugagugg................. 167 0 mmu
.......................................................................agggaacgcagGcugagugg................. 1 1 mmu
.......................................................................agggaacgcagCcugagugga................ 3 1 mmu
.......................................................................aggAaacgcagucugagugga................ 1 1 mmu
.......................................................................agggaacgcagucugagugga................ 1773 0 mmu
.......................................................................agggaacgcagucCgagugga................ 2 1 mmu
.......................................................................Ugggaacgcagucugagugga................ 1 1 mmu
```

Star **Mature**

aggugcugcuugauugcagcugccucuugccauugaugaucauucuucucuccuucgggagggugagagggagggaacgcagucugagugga uuugguuuauuaaaaa			
....Ggggaacgcagucugagugga................	1	1	mmu
....agggGacgcagucugagugga................	2	1	mmu
....Cgggaacgcagucugagugga................	1	1	mmu
....agggaacgcagucugaguAga................	1	1	mmu
....aggCaacgcagucugagugga................	2	1	mmu
....aUggaacgcagucugagugga................	12	1	mmu
....agggaacgcagucugUgugga................	1	1	mmu
....agggaacgcaguAugagugga................	1	1	mmu
....agggaacgcagGcugagugga................	1	1	mmu
....agggaacAcagucugagugga................	1	1	mmu
....agggaacgcGgucugagugga................	3	1	mmu
....agggaacgcagucugagCgga................	1	1	mmu
....gggaacgcagAcugagug..................	1	1	mmu
....gggaacgcagucugagug..................	68	0	mmu
....gggaUcgcagucugagugg.................	1	1	mmu
....gggaacgcagucugagugg.................	107	0	mmu
....gggaacgcaAucugagugg.................	2	1	mmu
....gggaacgcaguUugagugga................	1	1	mmu
....Cggaacgcagucugagugga................	1	1	mmu
....gggaacgcagucugGgugga................	2	1	mmu
....gggaacgcagucugaguAga................	1	1	mmu
....gggaacgcagucugaguUga................	1	1	mmu
....Aggaacgcagucugagugga................	1	1	mmu
....gggaacgcagCcugagugga................	3	1	mmu
....gggaacgcagucugagugga................	1294	0	mmu
....gggaacgcagucugagCgga................	1	1	mmu
....gggaacgcaguAugagugga................	1	1	mmu
....Uggaacgcagucugagugga................	22	1	mmu
....ggaacgcagucugagugg.................	45	0	mmu
....ggaGcgcagucugagugga................	6	1	mmu
....ggaacgcagucugagCgga................	2	1	mmu
....ggaacgcagAcugagugga................	1	1	mmu
....ggaaGgcagucugagugga................	1	1	mmu
....ggaacgcaAucugagugga................	1	1	mmu
....ggaaUgcagucugagugga................	1	1	mmu
....ggaacgcagCcugagugga................	1	1	mmu
....gAaacgcagucugagugga................	1	1	mmu
....ggaacgUagucugagugga................	17	1	mmu
....ggaacgcagucugaguAga................	2	1	mmu
....ggGacgcagucugagugga................	2	1	mmu
....ggaacgcGgucugagugga................	2	1	mmu
....ggaacgcagucugCgugga................	1	1	mmu
....ggaUcgcagucugagugga................	1	1	mmu
....ggaacgcagucugaAugga................	5	1	mmu
....ggaacgcagucugagugga................	2699	0	mmu
....ggaacgcagucAgagugga................	1	1	mmu
....ggaacgcagucugGgugga................	4	1	mmu
....ggaacgcagucuAagugga................	2	1	mmu
....ggaacgcagucCgagugga................	1	1	mmu
....gaacgcaAucugagugga................	1	1	mmu
....gaacgcagucugaguggG................	2	1	mmu
....gaacgcagucugagugAa................	29	1	mmu
....gaaGgcagucugagugga................	1	1	mmu
....gaacgcagucuUagugga................	1	1	mmu
....gaacgcagucCgagugga................	2	1	mmu
....gaacgcagucGgagugga................	1	1	mmu
....gaacgcagucugagGgga................	1	1	mmu
....gaacgUagucugagugga................	1	1	mmu
....Aaacgcagucugagugga................	1	1	mmu
....gaacgcaguUugagugga................	1	1	mmu
....gaacgcagucuCagugga................	1	1	mmu
....gaGcgcagucugagugga................	1	1	mmu
....gaacgcagucugagugga................	3534	0	mmu
....gaaUgcagucugagugga................	1	1	mmu
....Uaacgcagucugagugga................	1	1	mmu
....gaacgcagucugGgugga................	2	1	mmu
....gaacgcagCcugagugga................	3	1	mmu
....gGacgcagucugagugga................	2	1	mmu
....gaacgcGgucugagugga................	2	1	mmu
....gaacUcagucugagugga................	1	1	mmu

```
                              Star                                    Mature
aggugcugcuugauugcagcugccucuugccauugaugaucauucuucucuccuucgggagggugagagggagggaacgcagucugaguggauuugguuuauuaaaaa
..........................................................................gaacAcagucugagugga................    2    1    mmu
...........................................................................aacgcagucugaguggaG...............    24   1    mmu
```

Provisional ID	:	gi_307603382_gb_CM000994.1__22531
Score total	:	3414.6
Score for star read(s)	:	3.9
Score for read counts	:	3412.9
Score for mfe	:	-3.7
Score for randfold	:	1.6
Score for cons. seed	:	
Total read count	:	6706
Mature read count	:	5133
Loop read count	:	230
Star read count	:	1343

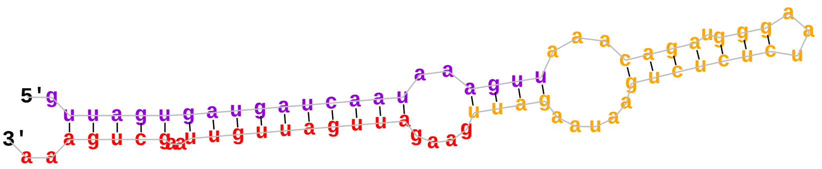

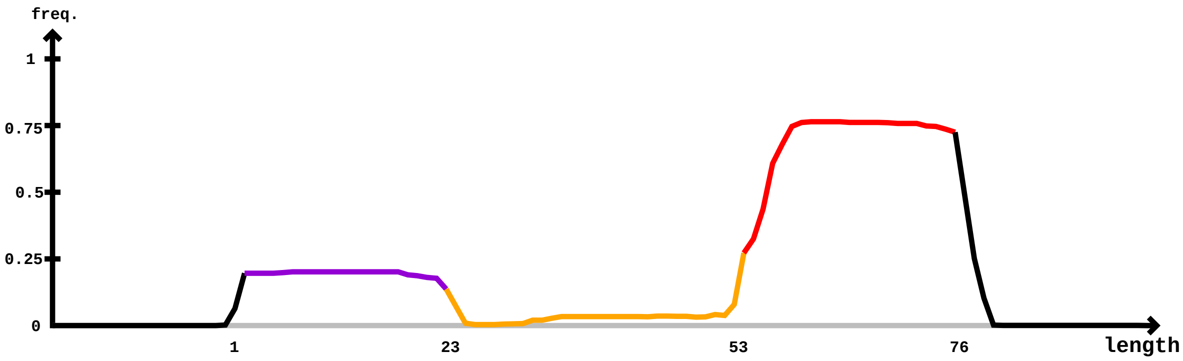

Star Mature

```
5'- uauaugaauuaagguacuguuagugaugaucaauaaaguuaaacagaugggaaucucucugaauaagauugaagauugauuguuaagcugaaacaguauuuaaggacaucagu   -3'   obs
    uauaugaauuaagguacuguuagugaugaucaauaaaguuaaacagaugggaaucucucugaauaagauugaagauugauuguuaagcugaaacaguauuuaaggacaucagu         exp
    ...........(((((((((((((((((((((((..((((...((((.(((...))))))).....))))....))))))))))..))))..)))))))))............   reads   mm   sample
    ................cuguuagugaugaucaauaaaguu.........................................................................   14   0   mmu
    .................uguuagugaugauUaaua..............................................................................   1   1   mmu
    .................uguuagugaugaucaaua..............................................................................   75   0   mmu
    .................uguuagugaugaucaauaa.............................................................................   24   0   mmu
    .................uguuagugaugaucaauaaa............................................................................   42   0   mmu
    .................uguuagugaGgaucaauaaa............................................................................   1   1   mmu
    .................uguuagugaugaCcaauaaagu..........................................................................   1   1   mmu
    .................uguuagugaugaucaauaaagu..........................................................................   76   0   mmu
    .................uguuagugGugaucaauaaaguu.........................................................................   1   1   mmu
    .................uguuagugaugaucaauaaaguu.........................................................................   169   0   mmu
    .................uguuagugaugaucGauaaaguu.........................................................................   1   1   mmu
    .................uguuagugaugaucaauaaaguua........................................................................   30   0   mmu
    .................uguuagugGugaucaauaaaguua........................................................................   2   1   mmu
    ..................guuagugaugaucaauaaag...........................................................................   27   0   mmu
    ..................guuagugaugaucaauaaagu..........................................................................   200   0   mmu
    ..................guCagugaugaucaauaaaguu.........................................................................   59   1   mmu
    ..................guuagugaugaCcaauaaaguu.........................................................................   1   1   mmu
    ..................guuagugaugaucaaCaaaguu.........................................................................   1   1   mmu
    ..................guuagugaugaucaauaaaguu.........................................................................   531   0   mmu
    ..................guuagCgaugaucaauaaaguu.........................................................................   2   1   mmu
    ..................guuagugaugaucaauaaUguu.........................................................................   1   1   mmu
    ..................guuaUugaugaucaauaaaguu.........................................................................   1   1   mmu
    ..................guuagugaugaucaauaaaguua........................................................................   45   0   mmu
    ..................guuagugaugaucaGuaaaguua........................................................................   1   1   mmu
    ..................guuagugaugaucaauaaaguuaa.......................................................................   35   0   mmu
    ......................gugaugaucaauaaaguu.........................................................................   16   0   mmu
    .......................ugaugaucaauaaaguuaaacaga..................................................................   20   0   mmu
    ......................................uuaaacagaugggaaucucucu.....................................................   5   0   mmu
    .............................................gaugggaaucucucugaa..................................................   13   0   mmu
    ..............................................augggaaucucucugaaua................................................   6   0   mmu
    ...............................................ugggaaucucucugaaua................................................   15   0   mmu
    ...............................................ugggaaucucucugaauaaga.............................................   12   0   mmu
    ................................................gggaaucucucugaauaaga.............................................   21   0   mmu
```

Star **Mature**

```
uauaugaauuaagguacuguuagugaugaucaauaaaguuaaacagaugggaaucucucugaauaagauugaagauugauuguuaagcugaaacaguauuuaaggacaucagu
................................................gggaaucucucugaauaagau............................................	41	0	mmu
................................................gggaaucucucugaauaagauu...........................................	24	0	mmu
................................................gggaaucucucAgaauaagauu...........................................	1	1	mmu
..................................................gaCucucucugaauaaga.............................................	1	1	mmu
..................................................gaaucucucugaauaaga.............................................	48	0	mmu
...................................................aaucucuGugaauaagauu...........................................	1	1	mmu
...................................................aaucucucugaauaagauu...........................................	42	0	mmu
.............................................................aauaagauugaagauugauu................................	18	0	mmu
...............................................................uaagauugaagauugauuguua............................	7	0	mmu
..................................................................gauugaagauugauuguuaagcu........................	7	0	mmu
...................................................................auugaagauugauuguuaa...........................	19	0	mmu
...................................................................auugaagauugauuguuaagcu........................	21	0	mmu
...................................................................auugaagauugauuguuaagcuga......................	19	0	mmu
....................................................................uugaagauugauuguuaagcu........................	20	0	mmu
....................................................................uugaagauugauuguuaagcug.......................	13	0	mmu
....................................................................uugaagauugauuguuaagcugaa.....................	26	0	mmu
.....................................................................ugaagauugauuguuaagcu........................	16	0	mmu
.....................................................................uUaagauugauuguuaagcugaaa....................	28	1	mmu
.....................................................................ugaagauugauuguuaagcugaaa....................	281	0	mmu
......................................................................gaagauugauuguuaagcuga......................	23	0	mmu
......................................................................gGagauugauuguuaagcugaa.....................	1	1	mmu
......................................................................gaagauugauuguuaagcugaa.....................	32	0	mmu
......................................................................gaagauugauuguuaagcugaGa....................	1	1	mmu
......................................................................gaagauugauuguuaagcugaaa....................	1257	0	mmu
......................................................................gaagauugauugCuaagcugaaa....................	1	1	mmu
......................................................................Uaagauugauuguuaagcugaaa....................	15	1	mmu
......................................................................gaagauugauuguuaagcugaaG....................	5	1	mmu
......................................................................gaUgauugauuguuaagcugaaa....................	1	1	mmu
......................................................................gaagauugauuguuaGgcugaaa....................	3	1	mmu
......................................................................gaagauugauuguuCagcugaaa....................	1	1	mmu
......................................................................gaagauugauuguuGagcugaaa....................	1	1	mmu
......................................................................gaagauugaCuguuaagcugaaac...................	1	1	mmu
......................................................................gaagauugauuguuaagcugaaac...................	44	0	mmu
.......................................................................aagauugauuguuaagcuga......................	27	0	mmu
.......................................................................aagauugauuguuaagcugaaa....................	236	0	mmu
.......................................................................aagauugauugCuaagcugaaa....................	1	1	mmu
.......................................................................aagauugauuguuaagcugaaaca..................	96	0	mmu
.......................................................................aagauugaAuguuaagcugaaaca..................	1	1	mmu
........................................................................agauugauuguuaagcugaa.....................	23	0	mmu
........................................................................agauugauuguuaagcugaGa....................	1	1	mmu
........................................................................agauugauuguCaagcugaaa....................	1	1	mmu
........................................................................agauGgauuguuaagcugaaa....................	1	1	mmu
........................................................................agauugauugCuaagcugaaa....................	1	1	mmu
........................................................................agauugauuguuaagcAgaaa....................	1	1	mmu
........................................................................agauugauuguuaGgcugaaa....................	1	1	mmu
........................................................................agauugUuuguuaagcugaaa....................	1	1	mmu
........................................................................agauugauuguuaagcugaaa....................	467	0	mmu
........................................................................agauugauuguuaagcugaaG....................	1	1	mmu
........................................................................agauugauuguuaagcugaaacG..................	2	1	mmu
........................................................................agauugauuguuaagcCgaaaca..................	1	1	mmu
........................................................................agauugauuguuaagcugaaGca..................	1	1	mmu
........................................................................agaCugauuguuaagcugaaaca..................	1	1	mmu
........................................................................agauugauuguuaagcugaaaca..................	136	0	mmu
........................................................................aUauugauuguuaagcugaaaca..................	22	1	mmu
........................................................................agaCugauuguuaagcugaaacag.................	1	1	mmu
........................................................................Ggauugauuguuaagcugaaacag.................	1	1	mmu
........................................................................agauugauuguuaagcugaaacag.................	101	0	mmu
........................................................................agauugauugCuaagcugaaacag.................	1	1	mmu
.........................................................................gauugauuguuaagcugaaa....................	456	0	mmu
.........................................................................gauugauuguuaagcugaCa....................	1	1	mmu
.........................................................................gauugauuAuuaagcugaaa....................	21	1	mmu
.........................................................................Uauugauuguuaagcugaaa....................	30	1	mmu
.........................................................................gauugauuguuaagcugaGa....................	1	1	mmu
.........................................................................gauugauuguuaagcCgaaa....................	1	1	mmu
.........................................................................gauuAauuguuaagcugaaa....................	1	1	mmu
.........................................................................gauugauugCuaagcugaaa....................	1	1	mmu
.........................................................................gauGgauuguuaagcugaaa....................	1	1	mmu
.........................................................................Cauugauuguuaagcugaaa....................	1	1	mmu
.........................................................................gauugauuguuaagcugGaaca..................	1	1	mmu
.........................................................................gauugauuguuaagcugaaaca..................	414	0	mmu
```

Star **Mature**

uauaugaauuaagguacuguuagugaugaucaauaaaguuaaacagaugggaaucucucugaauaagauugaagauugauuguuaagcugaaacaguauuuaaggacaucagu			
....gauugauuguuaagcugaGaca..................	1	1	mmu
....gauugauuguuaGgcugaaaca..................	2	1	mmu
....gauCgauuguuaagcugaaaca..................	1	1	mmu
....gauugauuguuaagcugaaacaA.................	10	1	mmu
....gauugauuguuaagcugaaacCg.................	7	1	mmu
....gauugauuguuaagcugaUacag.................	1	1	mmu
....gauugauuguuaagcugaaacaU.................	1	1	mmu
....gauugauuguuaagcugaaacag.................	217	0	mmu
....gauugauugCuaagcugaaacag.................	1	1	mmu
....gUuugauuguuaagcugaaacag.................	1	1	mmu
....auugauuguuaagcugaaa....................	64	0	mmu
....auugauuguuaagcugaaac...................	40	0	mmu
....auugauuguuaagcugaaaca..................	83	0	mmu
....auugauuguuaagcugaaacaA.................	2	1	mmu
....auugauuguuaagcugaaacag.................	284	0	mmu
....auugauuguuaagcugaaacaC.................	1	1	mmu
....auCgauuguuaagcugaaacag.................	1	1	mmu
....auugauuguuaagGugaaacag.................	1	1	mmu
....auugaCuguuaagcugaaacag.................	5	1	mmu
....auugauuguuaagcugaaacagu................	8	0	mmu
....uuAauuguuaagcugaaa....................	1	1	mmu
....uugauuguuaGgcugaaa....................	1	1	mmu
....uugauuguuaagUugaaa....................	1	1	mmu
....uugauuguuaagcuAaaa....................	2	1	mmu
....uugauuguuaagcugaaa....................	234	0	mmu
....uugauuguuaaAcugaaa....................	1	1	mmu
....uugauuguuaagcugaaac...................	13	0	mmu
....uugauuguuaagcugaaaca..................	147	0	mmu
....uugauuguuaagcugaaacag.................	56	0	mmu
....uugauuguuaagcuUaaacag.................	1	1	mmu
....ugauuguuaagcugaaac...................	15	0	mmu
....ugauuguuaagcugaaaca..................	85	0	mmu
....gauuguuaagcugaaaca..................	20	0	mmu
....acaguauuuaaggacaucag.	4	0	mmu

Provisional ID	:	gi_307603381_gb_CM000995.1__27908
Score total	:	369.8
Score for star read(s)	:	3.9
Score for read counts	:	361.6
Score for mfe	:	2.7
Score for randfold	:	1.6
Score for cons. seed	:	
Total read count	:	721
Mature read count	:	571
Loop read count	:	1
Star read count	:	149

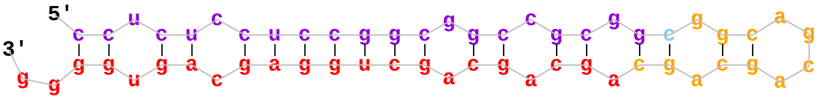

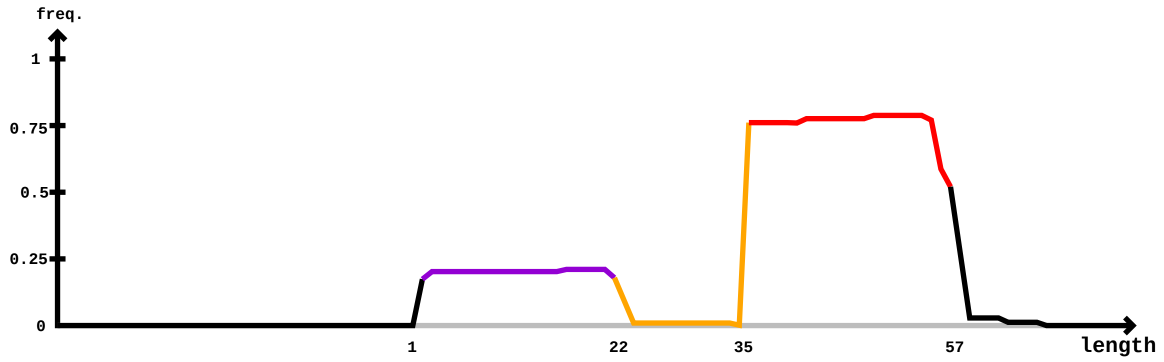


```
                                        Star                     Mature
5'- cgggccgggcagugacgcgccgcggcggugccagccccucuccuccggcggccgcggcggcagcagcagcagcagcagcuggagcaguggggcugucaccgccucccgcc  -3'   obs
    cgggccgggcagugacgcgccgcggcggugccagccccucuccuccggcggccgcggcggcagcagcagcagcagcagcuggagcaguggggcugucaccgccucccgcc         exp
    ((((.(((((......)).))).(((((((.(((((((.((.(((((((.((.((.((.((....)).)).)).)).))))))).)).))))))).))))))).))))..   reads   mm   sample
    ....................................ccucuccuccggcggccgGg......................................................   1       1    mmu
    ....................................ccucuccuccggcggccgcg......................................................   22      0    mmu
    ....................................ccucuccuccggcggccgcgg.....................................................   57      0    mmu
    ....................................ccucuccuccggcggccgcggc....................................................   48      0    mmu
    .....................................cucuccuccggcggccgcggc....................................................   21      0    mmu
    ...................................................ccgUggcggcagcagcag.........................................   6       1    mmu
    .........................................................cggcagcagcagcag......................................   1       0    mmu
    ......................................................................agcagcagcuggagcagug.....................   13      0    mmu
    ......................................................................agcagcagcuggagcagugg....................   135     0    mmu
    ......................................................................agcagcagcuggagcaguggA...................   13      1    mmu
    ......................................................................agcagcagcuggagcaguggg...................   36      0    mmu
    ......................................................................agcagcagcuggagcagugggU..................   361     1    mmu
    ......................................................................agcagcagcuggagcagugggg..................   1       0    mmu
    ............................................................................agcuggagcaguggggcuguc.............   12      0    mmu
    ...................................................................................gcaguggggcugucaccg.........   9       0    mmu
```

Provisional ID	:	gi_307603364_gb_CM001012.1__20539
Score total	:	260.8
Score for star read(s)	:	3.9
Score for read counts	:	253.5
Score for mfe	:	1.8
Score for randfold	:	1.6
Score for cons. seed	:	
Total read count	:	509
Mature read count	:	471
Loop read count	:	0
Star read count	:	38

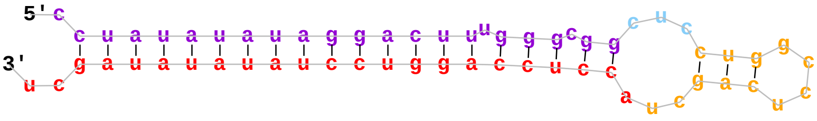

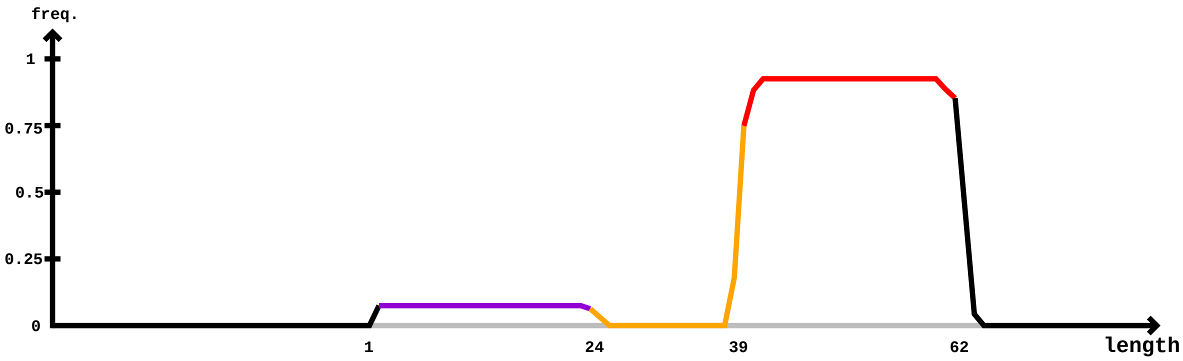

Star **Mature**

```
5'- ugucagcaaucucccagaucguggcgacugaaccuauauauaggacuuugggcggcuccuggccucagcuaccuccagguccuauauauagcuugggucuccauagccccgac  -3'   obs
    ugucagcaaucucccagaucguggcgacugaaccuauauauaggacuuugggcggcuccuggccucagcuaccuccagguccuauauauagcuugggucuccauagccccgac        exp
    .(((.((.((((...)))).((((.((((.((.((((((((((((((.(((.((...(((....)))...)))))))))))))))))))).)).)))).)))).))...)))   reads   mm   sample
    ................................ccuauauauaggacuuugggcg...........................................................   6       0    mmu
    ................................ccuauauauaggacuuugggcgg..........................................................   32      0    mmu
    .....................................................................uaccuccagguccuauauauag......................   20      0    mmu
    .....................................................................uaccuccagguccuauauauagc.....................   16      0    mmu
    .....................................................................uaccuccagguccuauaGauagc.....................   1       1    mmu
    .....................................................................uaccuccagguccuauauauagcu....................   54      0    mmu
    ......................................................................accuccagguccuauauauagcA....................   1       1    mmu
    ......................................................................Gccuccagguccuauauauagcu....................   1       1    mmu
    ......................................................................accuccagguccuaGauauagcu....................   1       1    mmu
    ......................................................................accuccaggAccuauauauagcu....................   1       1    mmu
    ......................................................................accuccagguccuauauauagcC....................   31      1    mmu
    ......................................................................accuccagguccuauauauagcu....................   217     0    mmu
    ......................................................................accuccagguccuauauauagcuu...................   37      0    mmu
    ......................................................................accuccagguccuauaCauagcuu...................   1       1    mmu
    .......................................................................ccuccagguccuauauauagcu....................   67      0    mmu
    .......................................................................Acuccagguccuauauauagcu....................   1       1    mmu
    ........................................................................cuccagguccuauauauagcuuC..................   1       1    mmu
    ........................................................................cuccagguccuauauauagcuuA..................   21      1    mmu
```

Provisional ID	:	gi_307603366_gb_CM001010.1__17021
Score total	:	249.6
Score for star read(s)	:	3.9
Score for read counts	:	242.3
Score for mfe	:	1.8
Score for randfold	:	1.6
Score for cons. seed	:	
Total read count	:	487
Mature read count	:	403
Loop read count	:	6
Star read count	:	78

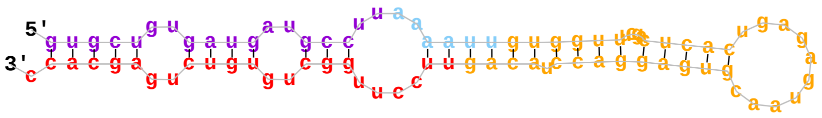

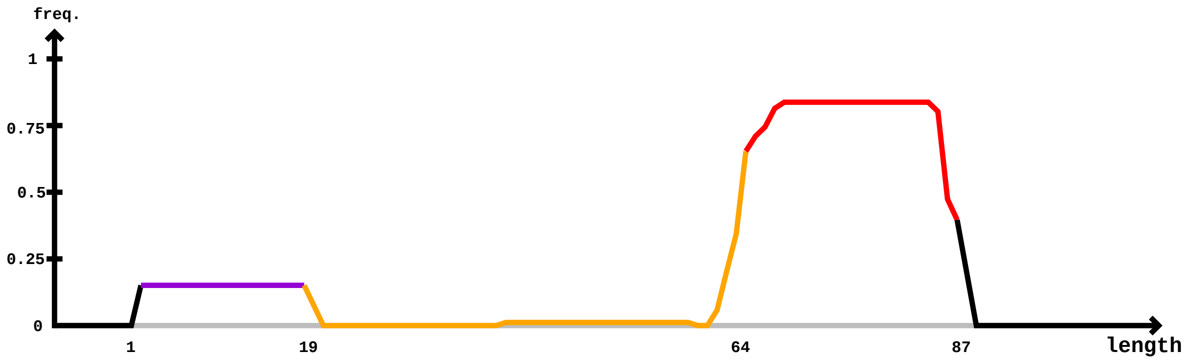

Star   Mature

```
5'- agcuguggugcugugaugaugccuuaaaauugugguuucgacucacugagaguaacgugaggaccuacaguuccuuggcugugucugagcaccagagcaccccuuuagaaaug   -3'    obs
    agcuguggugcugugaugaugccuuaaaauugugguuucgacucacugagaguaacgugaggaccuacaguuccuuggcugugucugagcaccagagcaccccuuuagaaaug          exp
    .(((.(((((((..((((..(((....((((((((((....(((((..........))))))))).))))))....)))..))))..))))))).)))................     reads     mm     sample
    .......gugcugugaugaugccuu........................................................................................     53        0      mmu
    .......gugcugugaugaugGcuu........................................................................................     1         1      mmu
    .......gugcugugaugaugccuua.......................................................................................     24        0      mmu
    .............................................cugagaguaacgugaggacc................................................     6         0      mmu
    ...................................................................caguuccuuggcugugucugagca......................     30        0      mmu
    ....................................................................aguuccuuggcugugucugagc.......................     18        0      mmu
    ....................................................................aguuccuuggcugugCcugagca......................     1         1      mmu
    ....................................................................aguuccuuggcugugucugagca......................     32        0      mmu
    ....................................................................aguuccuuggcugugucugagcac.....................     23        0      mmu
    ....................................................................aguGccuuggcugugucugagcac.....................     1         1      mmu
    .....................................................................gCuccuuggcugugucugagca......................     1         1      mmu
    .....................................................................guuccuuggcugugucugagca......................     70        0      mmu
    .....................................................................guAccuuggcugugucugagca......................     1         1      mmu
    .....................................................................guuccuuggcugugucugagGa......................     1         1      mmu
    ......................................................................uuccuuggcugugucugagca......................     9         0      mmu
    ......................................................................Cuccuuggcugugucugagca......................     1         1      mmu
    ......................................................................uuccuuggcugugucugagcac.....................     15        0      mmu
    ......................................................................uuccuuggcuguguAugagcac.....................     1         1      mmu
    ......................................................................uuccuuggcugugucugagcacc....................     132       0      mmu
    ......................................................................Cuccuuggcugugucugagcacc....................     1         1      mmu
    ......................................................................uuccuuggcugCgucugagcacc....................     1         1      mmu
    .......................................................................uccuuggcugugucugagca......................     11        0      mmu
    .......................................................................ucUuuggcugugucugagca......................     1         1      mmu
    .......................................................................uccuuggcugugucugagcacG....................     17        1      mmu
    ........................................................................ccuuggcugugucugagca......................     12        0      mmu
    ........................................................................ccuuggcugugucugagcacc....................     6         0      mmu
    .........................................................................cuuggcugugucugagcacc....................     34        0      mmu
    .........................................................................cuuggcugugucCgagcacc....................     1         1      mmu
    .........................................................................cCuggcugugucugagcacc....................     1         1      mmu
    ..........................................................................uuggcugugucugagcacc....................     12        0      mmu
```
