# Supplementary material for: Identification of novel microRNAs in the embryonic mouse brain using deep sequencing
Source: Mol Cell Biochem. 2023 Apr 15;479(2):297–311. doi: 10.1007/s11010-023-04730-2 (PMC10890980; doi:10.1007/s11010-023-04730-2)

Conservation Plot for Novel 11

i)

ii)

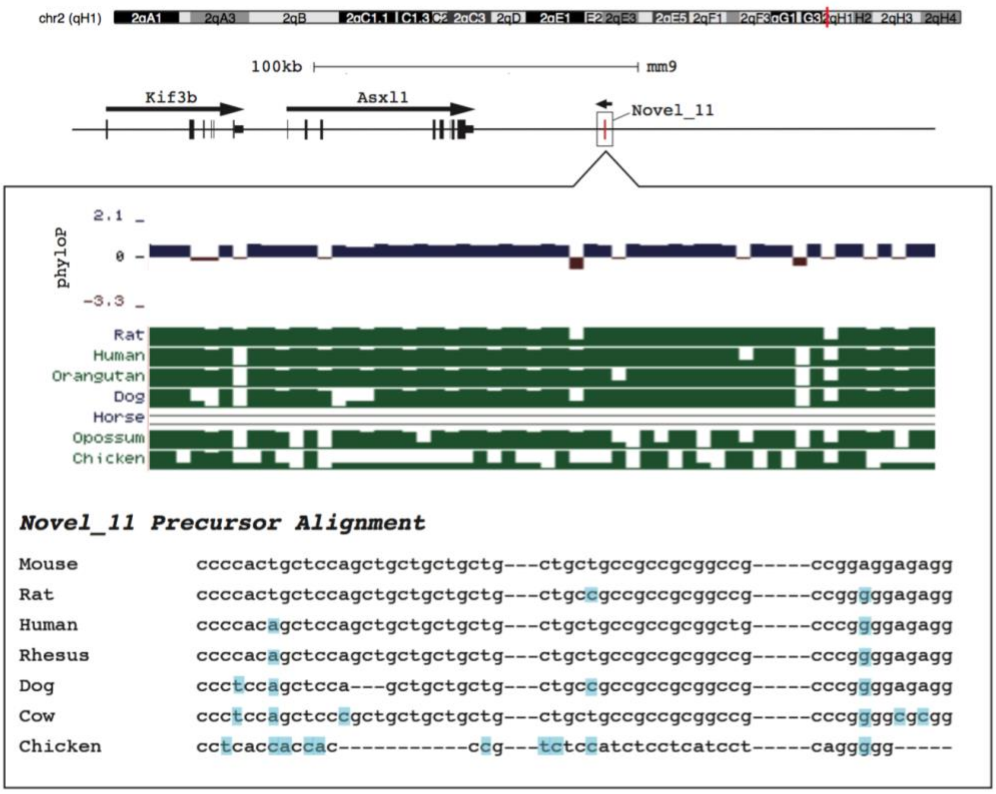

Conservation Plot for Novel 2

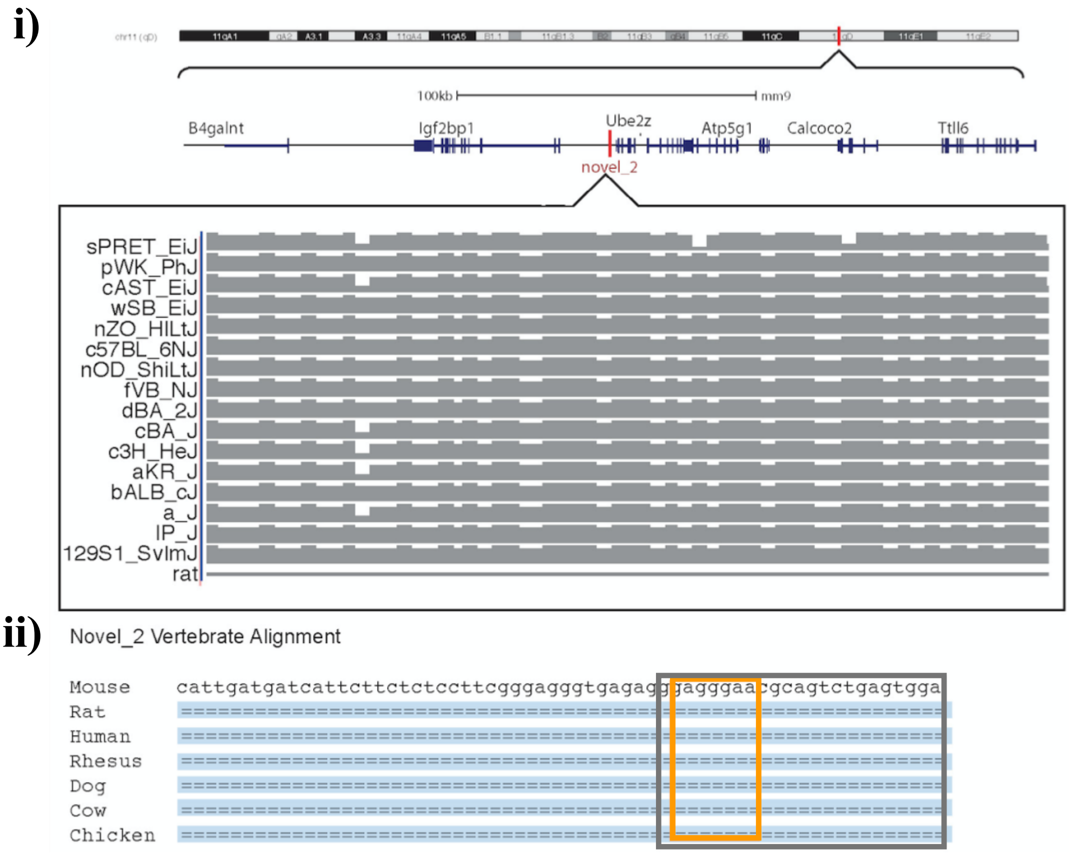

Supplement: Supplementary file 5 — gProfiler GO analysis for the target genes of six candidate miRNAs. Supplementary file5 (PDF 458 KB) [file 11010_2023_4730_MOESM5_ESM.pdf]
