# Supplementary material for: Identification of novel microRNAs in the embryonic mouse brain using deep sequencing
Source: Mol Cell Biochem. 2023 Apr 15;479(2):297–311. doi: 10.1007/s11010-023-04730-2 (PMC10890980; doi:10.1007/s11010-023-04730-2)
Supplement: Supplementary file 6 — Conservation analysis for candidate miRNAs. Supplementary file6 (PDF 40 KB) [file 11010_2023_4730_MOESM6_ESM.pdf]

Table S1: miRNA RT-qPCR primer sequences.

**miRNA**	**Forward primer sequence (5’-3’)**
cel-miR-39	TCACCGGGTGTAAATCAGCTTG
Novel_1	TTCTCACTACTGCACTTGACTAGT
Novel_2	GGAGGGAACGCAGTCTGAGTGGA
Novel_3	GAAGATTGATTGTTAAGCTGAAA
Novel_11	CAGCAGCTGGAGCAGTGGGGAAAAAA
Novel_16	CCTCCAGGTCCTATATATAGC
Novel_17	TTCCTTGGCTGTGTCTGA
